# Selective cell cycle arrest in glioblastoma cell lines by quantum molecular resonance alone or in combination with temozolomide

**DOI:** 10.1038/s41416-022-01865-9

**Published:** 2022-06-17

**Authors:** Daniela Catanzaro, Gloria Milani, Angela Bozza, Martina Bernardi, Katia Chieregato, Martina Menarin, Anna Merlo, Paola Celli, Romina Belli, Daniele Peroni, Alessandro Pozzato, Gianantonio Pozzato, Fabio Angelo Raneri, Lorenzo Volpin, Marco Ruggeri, Giuseppe Astori

**Affiliations:** 1 Advanced Cellular Therapy Laboratory, Hematology Unit, Vicenza Hospital, Vicenza, Italy; 2 CORIS, Consorzio per la Ricerca Sanitaria, Via N. Giustiniani, 2, 35128 Padova, Italy; 3 Genetic Unit, Vicenza Hospital, Vicenza, Italy; 4 Mass Spectrometry and Proteomics Facility, Department of Cellular, Computational and Integrative Biology, CIBIO University of Trento, Trento, Italy; 5 Telea Electronic Engineering srl, Sandrigo (VI), Italy; 6 Department of Neurosurgery, Vicenza Hospital, Vicenza, Italy; 7 Hematology Unit, Vicenza Hospital, Vicenza, Italy

**Keywords:** Cancer therapy, Mitotic spindle, Cancer therapy

## Abstract

**Background:**

Glioblastoma is the most aggressive form of brain cancer, characterised by high proliferation rates and cell invasiveness. Despite advances in surgery and radio-chemotherapy, patients continue to have poor prognoses, with a survival rate of 14–15 months. Thus, new therapeutic strategies are needed. Non-ionising electromagnetic fields represent an emerging option given the potential advantages of safety, low toxicity and the possibility to be combined with other therapies.

**Methods:**

Here, the anticancer activity of quantum molecular resonance (QMR) was investigated. For this purpose, three glioblastoma cell lines were tested, and the QMR effect was evaluated on cancer cell proliferation rate and aggressiveness. To clarify the QMR mechanism of action, the proteomic asset after stimulation was delineated. Mesenchymal stromal cells and astrocytes were used as healthy controls.

**Results:**

QMR affected cancer cell proliferation, inducing a significant arrest of cell cycle progression and reducing cancer tumorigenicity. These parameters were not altered in healthy control cells. Proteomic analysis suggested that QMR acts not only on DNA replication but also on the machinery involved in the mitotic spindle assembly and chromosome segregation. Moreover, in a combined therapy assessment, QMR significantly enhanced temozolomide efficacy.

**Conclusions:**

QMR technology appears to be a promising tool for glioblastoma treatment.

## Background

Due to its incomparable ability to infiltrate the surrounding parenchyma, glioblastoma (GBM) is among the most aggressive malignant tumours [1, 2]. Conventional therapy for GBM includes maximal surgical resection followed by radiotherapy and chemotherapy with temozolomide (TMZ), an oral alkylating anticancer agent [3–5]. Despite this multidisciplinary approach, GBM is characterised by high recurrence rates, drug resistance, and devastating neurological deterioration [6]. Most patients with GBM have a poor prognosis, with 14 months estimated survival at the time of diagnosis [7], and less than 5% of patients still alive at 5 years [8]. Thus, new therapeutic strategies directed at improving drug efficacy and reducing related adverse events are needed.

In this regard, non-ionising electromagnetic fields (EMFs) represent an emerging option given their potential advantages of safety and low toxicity, as well as the ability to combine their use with other therapies [9]. In recent years, several EMF technologies have been proposed, and their efficacy in the treatment of a wide variety of tumours when used alone [10–14] and in combination with chemotherapy [15–17] has been demonstrated. EMFs have different mechanisms of action (MoAs) depending on the frequency range, but they have the common final effect of reducing cancer cell proliferation, mainly by acting on mitotic spindle formation and stability [14, 18]. In 2011, the American Food and Drug Administration approved the use of Tumour Treating Fields (TTFields) in patients with recurrent glioblastoma, and promising results have led to the extension of this application to the treatment of newly diagnosed GBM in combination with TMZ [19, 20].

In this study, the in vitro anticancer activity of QMR was investigated. Unlike TTFields, which exploits intermediate-frequency (100–300 kHz) alternating electric fields [21], QMR is a non-ionising, low potency technology that uses high-frequency waves in the range of 4–64 MHz, thus probably acting with a different MoA. Currently, QMR technology is applied mainly in bipolar coagulators and electrosurgery devices [22–24]. For this kind of use, the molecular resonance generator works with a combination of four frequencies in the range of 4–16 MHz. QMR also has been applied to difficult-to-heal extremity wounds [25], in the treatment of post-surgical oedema and in physiotherapy [26]. To explain the efficacy observed in clinical practice, the MoAs of QMR were firstly investigated by Schiavon [27] and Dal Maschio [28] and more recently by our group [29], but nothing is known about its potential activity on cancer cells.

In contrast to other EMFs medical devices, QMR generates nanosecond pulses that might be able to penetrate the plasma membrane and interact with the inner organelles of cells [28].

In this study, the MoA of QMR was investigated in three different glioblastoma cell lines. QMR effect on the proliferation rate and cell cycle progression was firstly evaluated. Given the promising results, the capability of cancer cells to grow in a semi-solid medium was subsequently studied, highlighting QMR’s ability to reduce glioblastoma tumorigenicity. The molecular mechanism responsible for QMR efficacy was clarified in a thorough proteomic study. Mesenchymal stromal cells (MSCs) and astrocytes were in parallel irradiated to evaluate the effects of QMR on brain non-tumour cells; QMR was shown to be a cancer-selective approach. Lastly, as several studies have supported the combined use of TMZ and EMF to increase the efficacy of available treatments, the combinatory activity of QMR and TMZ on tumour cells at different concentrations was tested.

## Materials and methods

### Cell lines

The T98G (Sigma-Aldrich, St. Louis, MO, USA), A172 and U87MG (gifted by Prof. Massimo Dominici, Laboratory of Cellular Therapy, University Hospital of Modena and Reggio Emilia, Modena, Italy) glioblastoma cell lines were used in this work. A172 and T98G cells were cultured in Dulbecco’s modified Eagle medium (DMEM)/nutrient mixture F-12 GlutaMAX (Gibco, Thermo Fisher Scientific, Waltham, MA, USA) supplemented with 10% foetal bovine serum (FBS; Qualified Australian; Gibco, Thermo Fisher Scientific) and 1% penicillin/streptomycin (Sigma-Aldrich). U87MG cells were cultured in DMEM with GlutaMAX (Gibco, Thermo Fisher Scientific) supplemented with 10% FBS (Gibco, Thermo Fisher Scientific) and 1% penicillin/streptomycin (Sigma-Aldrich).

Adult human astrocytes (Cell Application, San Diego, CA, USA) and bone-marrow mesenchymal stromal cells were used as non-tumour control cells. MSCs were produced in our laboratory as previously described [29]. Briefly, MSCs were isolated from cells obtained from washouts of discarded bone-marrow collection bags and filters of healthy donors (Comitato Etico per le Sperimentazioni Cliniche Della Provincia di Vicenza authorisation no. 107/18, 12.02.2019). Cells were seeded at a density of 1 × 10^5^/cm^2^ in DMEM GlutaMAX (Gibco, Thermo Fisher Scientific) supplemented with 10% FBS (Gibco, Thermo Fisher Scientific) and 1% penicillin/streptomycin (Sigma-Aldrich). The cultures were incubated at 37 °C in a humidified atmosphere with 5% CO_2_. Human astrocytes were cultured in Human Astrocyte Growth Medium (Cell Application).

### QMR stimulation protocol

All cell lines were exposed to QMR using a generator prototype (Telea Electronic Engineering, Sandrigo, VI, Italy). This device generates alternating electric currents characterised by high-frequency (4–64 MHz) and low-intensity waves [29]. It was operated with the following parameters: power supply, 230 V, ~50/60 Hz; maximum power input, 250 VA; and power output, 5 W/400 Ω. QMR was applied using a pair of custom-made electrodes placed directly on the edge of a 100-mm Petri dish and connected to the QMR generator (Supplementary Fig. 1).

The transmission of electric fields to the culture medium generates heat according to the Joule–Lenz law [30]. The average temperature increase, calculated from three independent measurements taken with a data-logger probe (iLog; Escort, Scunthorpe, UK) placed in the culture medium, was 5 °C (Supplementary Fig. 2). Thus to ensure that the experimental temperature was 37 °C, the incubator was set to 32 °C with 5% CO_2_ and the laboratory temperature was maintained at 20–25 °C.

### Experimental setup

Depending on the cell line, a defined number of cells was seeded in 100-mm dishes (Greiner Bio-One, Frickenhausen, Germany) for stimulation at 60% confluence. The cells were then exposed to QMR for 24 h and analysed after 0–24–48 h from the end of stimulation (*t*_0_–*t*_24_–*t*_48_, respectively). In detail, the cells were washed with Dulbecco’s phosphate-buffered saline (D-PBS; Sigma-Aldrich) and detached with 1× TrypLE Select (Gibco, Thermo Fisher Scientific). Aliquots were used to investigate cell viability, cell cycles, karyotypes, and protein fingerprints. A cell aliquot was reseeded to evaluate cells' ability to grow in a semi-solid medium (soft agar assay), while cell morphology was monitored directly on the QMR-stimulated dish before and after stimulation (Supplementary Fig. 3).

The effect of combination therapy with QMR and TMZ (Sigma-Aldrich) was tested. The cells were treated for 144 h with 10–25 µM TMZ, administered together with or after 24 h QMR stimulation. Cell viability, apoptosis, and cell cycles were evaluated after 144 h.

### Trypan blue exclusion assay

After stimulation, the cells were harvested and suspended in trypan blue (Gibco, Thermo Fisher Scientific) at a 1:1 ratio in a medium solution. The cells were counted using a Burker hemocytometer (Paul Marienfeld GmbH & Co. KG, Lauda-Königshofen, Germany), and the number of viable cells was calculated using the following formula: [(cell number × 10,000 × D × V)/9], where D is the dilution factor and V represents the final volume.

### Cell cycle analysis

After stimulation, 5 × 10^5^ cells were harvested and centrifuged at 400×*g* for 6 min. The cells were washed with D-PBS and fixed/permeabilized with 80% acetone. After 1 h at 4 °C, the acetone was removed by centrifugation, washed (2×), and labelled with 1 µg/mL 7-AAD (Invitrogen) for 1 h at room temperature. The fluorescence of 2.5 × 10^4^ cells/sample was analysed using an FC500 flow cytometer (Beckman Coulter). To calculate the percentages of cells in different cell cycle phases, the EXPO 32 software (Coulter Systems, Fullerton, CA, USA) was used. Diploid cycles were considered and correction for cell clusters was performed.

### Annexin V/7-aminoactinomycin D staining

After stimulation, 1.5 × 10^5^ cells were harvested and centrifuged at 400×*g* for 6 min. After washing with binding buffer, the cells were labelled with annexin V/7-aminoactinomycin D (7-AAD) according to the manufacturer’s instructions (Invitrogen, Carlsbad, CA, USA). After dilution with binding buffer, the fluorescence of 2 × 10^4^ cells/sample was detected using an FC500 flow cytometer (Beckman Coulter, Brea, CA, USA). Cell populations were separated into four groups: viable cells (annexin V^–^/7-AAD^–^), cells in early apoptosis (annexin V^+^/7-AAD), cells in late apoptosis (annexin V^+^/7-AAD^+^) and necrotic cells (annexin V^−^/7-AAD^+^).

### Soft agar

The soft agar colony formation assay is used to monitor anchorage-independent growth, reflecting cell proliferation in a semi-solid culture medium, by optical counting of colonies [31]. As the rate of colony formation in soft agar varies among cell lines, the cell seeding density and the experiment end-date were optimised for each cell line. Cell suspensions were prepared in 0.4% agar/2x complete medium and overlaid on solidified 0.6% agar/2× complete medium. After 1 h at room temperature, 160 µl complete medium was added; an additional 100 µl was added every week until the end of the experiment. The plates were transferred to a 37 °C, 5% CO_2_ humidified incubator for 21–24 days before staining with 100 µM calcein (Sigma-Aldrich) for 30 min. Colonies of ≥50 cells were counted under an Axiovert 40 CFL inverted light microscope (Carl Zeiss).

### Karyotype analysis

To assess whether QMR exposure caused chromosomal alterations, astrocytes and MSCs were stimulated for 24 h and then subjected to G-Trypsin-Giemsa banding, following standard techniques with a resolution of 400 bands. Twenty metaphases were analysed and at least three metaphases were karyotyped. Unstimulated cells were used as a comparative control.

### Liquid chromatography/tandem mass spectrometry (LC/MS-MS)

#### Protein extraction

For LC-MS/MS sample preparation, A172 and MSC cells were harvested and centrifuged at 400×*g* for 6 min, washed with D-PBS, and lysed with ice-cold lysis buffer (Pierce™ RIPA buffer; Thermo Fisher Scientific) supplemented with a protease inhibitor cocktail (Cell Signaling Technology, Danvers, MA, USA). After 30 min on ice, the cell lysates were centrifuged at 14,000×*g* for 10 min at 4 °C and the supernatant protein content was determined by bicinchoninic acid colorimetric assay (Pierce™ BCA Protein Assay Kit; Thermo Fisher Scientific). Bovine serum albumin (Sigma-Aldrich) was used as the standard. In total, 50 μg of protein lysate was therefore subjected to acetone precipitation and the protein pellets were dissolved in 6 M urea and 100 mM ammonium bicarbonate (pH 8). The samples were reduced using 10 mM dithiothreitol for 1 h at room temperature and alkylated with 20 mM iodoacetamide in the dark for 30 min at room temperature. Subsequently, the proteins were digested with endopeptidase Lys-C (Promega, Madison, WI, USA) at an enzyme/protein ratio of 1:100 (w/w) for 3 h at room temperature. The proteins were then diluted four-fold in 50 mM ammonium bicarbonate and digested overnight with trypsin (Promega) at a ratio of 1:100 (w/w) at room temperature. Proteolysis was interrupted by the addition of 1% trifluoroacetic acid. The samples were then desalted using homemade stage tips (C18), vacuum dried, and resuspended in 0.1% formic acid for LC-MS/MS analysis.

#### LC-MS/MS analysis, database search and quantification

The samples were analysed using an Easy-nLC 1200 system coupled online with an Orbitrap Fusion Tribrid mass spectrometer (both from Thermo Fisher Scientific). A reverse-phase column (Acclaim PepMap RSLC C18 column, 2-µm particle size, 100-Å pore size, 75-µm i.d.; Thermo Fisher Scientific) with a two-component mobile phase system of 0.1% formic acid in water (buffer A) and 0.1% formic acid in acetonitrile (buffer B) was used to separate the digested peptides. The peptides were eluted using a 5–25% gradient over 52 min, followed by a 25–40% gradient over 8 min and a 40–98% gradient over 10 min, at a flow rate of 400 nL/min. The data-dependent acquisition method, based on full scans performed at 120,000 fwhm resolving power (at 200 *m/z*), the automatic gain control (AGC) target set at 1e6, and a 50-ms maximum injection time, was used. A mass range of 350–1100 *m/z* was surveyed for precursors, with the first mass set at 140 *m/z* for fragments. Full scans were followed by a set of high-energy collision dissociation (HCD) MS/MS scans over a 3-s cycle time, at 30% collision energy and detection in the ion trap with a maximum injection time of 150 ms. The AGC target was set at 5e3 and the dynamic exclusion time was set at 50 s. Raw data were searched using the Proteome Discoverer 2.2 software (Thermo Fisher Scientific). Peptide searches were performed using the human protein FASTA file (UniProt reviewed and downloaded July 2019). Proteins were identified using the MASCOT search engine (Matrix Science Inc., Boston, MA, USA) with a precursor mass tolerance of 10 ppm and product mass tolerance of 0.6 Da. Trypsin was chosen as the enzyme with three missed cleavages. Carbomidomethyl (C) as static modification and acetyl (protein N terminal) and oxidation (M) as the variable modification was incorporated in the search. Peak intensities were log2 transformed and data were normalised by the average of the protein abundance with each sample [32].

#### Bioinformatics analysis

The abundances of differentially expressed proteins across the experimental groups (at *t*_0_ and *t*_24_, respectively) were used to generate hierarchical clustergrams (using the correlation distance and complete linkage method) with the ClustVis web tool [33]. The gene ontology (GO) and Pathway annotation of protein IDs were performed using the comprehensive EnrichR gene set enrichment analysis web server (http://amp.pharm.mssm.edu/Enrichr/), applying Biological Processes and Reactome categorisation with the significance threshold set at *P* < 0.05. A protein interaction network was constructed using STRING interaction database, version 11.0 (https://string-db.org/) [34]. STRING analysis was performed by setting the species under investigation (Homo sapiens) with a medium confidence level (score 0.4); we retrieved interactions based on experimental and database knowledge, excluding all other prediction methods implemented in STRING.

### Western blot (WB)

Proteomic analysis of A172 and MSC cells was validated by western blot. At the aim, relevant proteins with a higher *P* value were selected by LC-MS raw data and analysed. The protein lysates extracted as above described were added with loading buffer 5× (lane marker reducing sample; Thermo Fisher Scientific) and denatured for 10 min at 95 °C. Equal amounts of protein (20 µg) were loaded on a 4–20% polyacrylamide gel (miniprotean TGX precast protein gels; Bio-rad) and electrophoretically separated in running buffer [25 mM Tris,192 mM glycine, 0.1% SDS, H_2_O q.b.], at a constant current of 100 V (Bio-rad Mini-PROTEAN^®^ Tetra System).

After electrophoresis, proteins were blotted onto a PVDF membrane (GE Healthcare, Chicago, IL, USA) in transfer buffer [25 mM Tris, 192 mM glycine, 20% methanol, water as required]. A current of 100 V for 105 min at 4 °C was applied. Non-specific binding sites were saturated using a TBST solution [20 mM TRIS, 140 mM NaCl, 0,1% Tween 20, water as required] added with 5% non-fat dried milk (Euroclone, Pero, MI, Italy). After washing with TBST, membranes were incubated overnight with the primary antibodies reported in Supplementary Table 1. Membranes were therefore exposed to HRP-conjugated anti-mouse secondary antibody (1:2000; Cell Signaling, Danvers, MA, USA) or anti-rabbit secondary antibody (1:1000; Invitrogen) for 1 h at room temperature.

Signals were visualised using Clarity Western ECL kit (Bio-rad) as per the manufacturer's instructions and analysed by Azure Imaging Systems (Azure Biosystems, Sierra Ct, Dublin, CA, USA). Anti-GAPDH was used as a housekeeping protein to normalise the integrated intensities.

### Bliss independence model

To determine the efficacy of QMR and temozolomide association therapy, Bliss Analysis was used. The combinatory effect was calculated as follows: Y_ab,P_ = Y_a_ + Y_b_ − Y_a_Y_b_ where Y_a_ and Y_b_ are the inhibitory effects of the elected treatments, respectively, used at doses a and b, while Y_ab,P_ is the predicted inhibitory percentage. This model is indeed based on the probability of obtaining a determined effect [35]. Thus, if the effective inhibitory percentage Y_ab,O_ is:

>Y_ab,P_ the combinatory effect is synergistic;

= Y_ab,P_ the effect of the two treatments is independent;

< Y_ab,P_ the combinatory effect is antagonistic.

### Statistical analysis

All data were analysed with GraphPad software (GraphPad Software, San Diego, CA, USA) and are expressed as means ± standard deviation (SD). The one-sample *t* test was used to analyse results expressed as ratios/percentages of controls. The unpaired Student’s *t* test was used for all other analyses. For LC-MS/MS analysis, statistical significance was assessed using a two-tails heteroscedastic *t* test. Significance was considered at *P* < 0.05.

## Results

### QMR reduces glioblastoma proliferation rate affecting cell cycle progression

The effect of QMR exposition on A172 glioblastoma cells was evaluated by analysing the cell morphology, proliferation rate, and cell cycle immediately after stimulation (*t*_0_) and 24 and 48 h thereafter (*t*_24_–*t*_48_). The use of this time course enabled estimation of the permanence of the QMR effect and the ability of injured cells to restore cell functions.

**Fig. 1:**
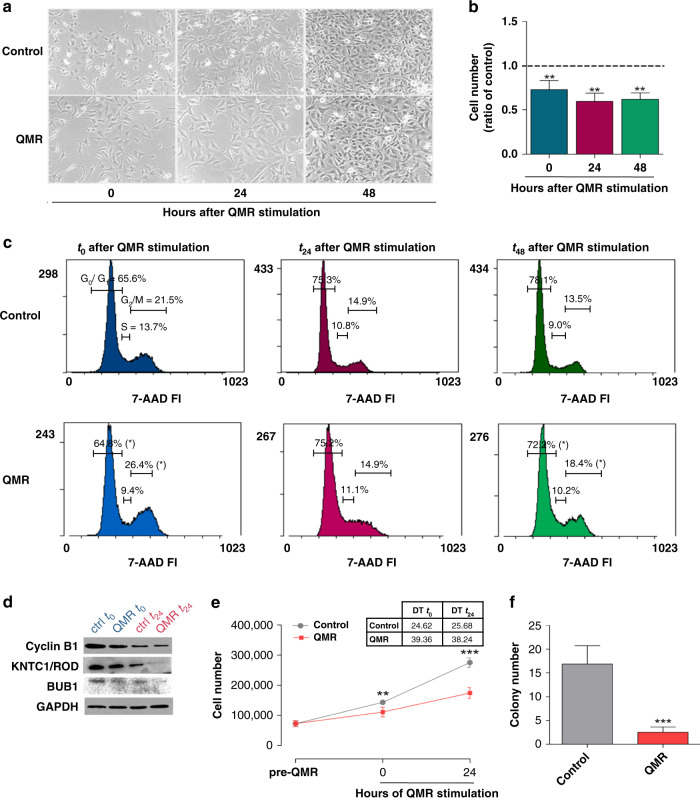
Effect of QMR stimulation on A172 glioblastoma cells. **a** Representative images of A172 at 0, 24 and 48 h after QMR exposure, acquired with an Axiovert 40 CFL inverted light microscope (Carl Zeiss, Oberkochen, Germany; ×10). **b** Cell viability was detected by trypan blue exclusion assay. Histograms represent the ratio of viable cells relative to that in unstimulated cells. **c** Cell cycle progression at 0, 24, and 48 h after QMR stimulation was monitored by flow cytometry. Representative cytograms report the percentage of cells in each cell cycle phase (G_0_–G_1_, S, G_2_–M). **d** Protein expression of cell cycle key regulators was evaluated by WB at *t*_0_–*t*_24_. GAPDH was used as a loading control. **e** A172 proliferation curves under basal conditions or after QMR treatment. Cells were counted before QMR and 0, 24 h after stimulation. *T*_48_ was not assessed because of technical/instrumental limits. Doubling times (DT) of control and stimulated cells are reported in the table. **f** A172 colonies were labelled with calcein and counted under an Axiovert 40 CFL inverted light microscope (Carl Zeiss, Oberkochen, Germany). Results are expressed as mean ± SD of at least four independent experiments. *P < 0.05, **P < 0.01, ***P < 0.001; QMR vs control.

As primary evidence of QMR activity, the morphology of stimulated A172 cells markedly differed from that of unstimulated cells. QMR-treated cells appeared swollen, more granulose, and lost the normal shape, indicating a cell injury effect that persisted over time likely due to QMR action on cell cycle progression (Fig. 1a).

The rate of cell proliferation was estimated by trypan blue assay. After 24 h of stimulation, QMR reduced tumour cell number by 28%. The percentage of viable cells had further decreased at 24 and 48 h (Fig. 1b), suggesting a QMR modulation of the cell cycle.

To further investigate this hypothesis, the A172 cell cycle was analysed by flow cytometry. QMR exposure significantly reduced the percentage of cells in the S phase with an associated increase of cells in the G_2_/M phase (Fig. 1c, *t*_0_). This effect persisted until 48 h after the end of QMR stimulation, indicating cell cycle arrest in the G_2_/M phase (Fig. 1c, *t*_48_).

The promotion of cell cycle progression is orchestrated by a considerable number of proteins. Among all, three key regulators with the higher *P* value in proteomic analysis (Supplementary Table 2) were selected to be confirmed by western blot. Representative images reported in Fig. 1d indicate an important decrease of cyclin B1 (coordinator of the G2/M transition), KNTC1/ROD and BUB1 (essential components of the mitotic checkpoint) protein content at both the considered timings.

QMR activity on the rate of cell division was corroborated by proliferation curves at three different time points (Fig. 1e). Results revealed a significant deceleration of QMR-stimulated cells at *t*_0_, still more evident at *t*_24_, with an almost twofold doubling time (DT; DT_t0_ control vs QMR = 24.62 vs 39.36, DT_t24_ control *vs* QMR = 25.68 vs 38.24).

The above-described effects of QMR technology finally result in the reduced ability of QMR-stimulated cells to grow in a semi-solid medium, an exclusive feature of cancer cells. Indeed, Fig. 1f depicts 85% fewer colonies after QMR stimulation than in control cells.

### QMR does not exhibit cytotoxic and genotoxic effects on non-tumour cells

**Fig. 2:**
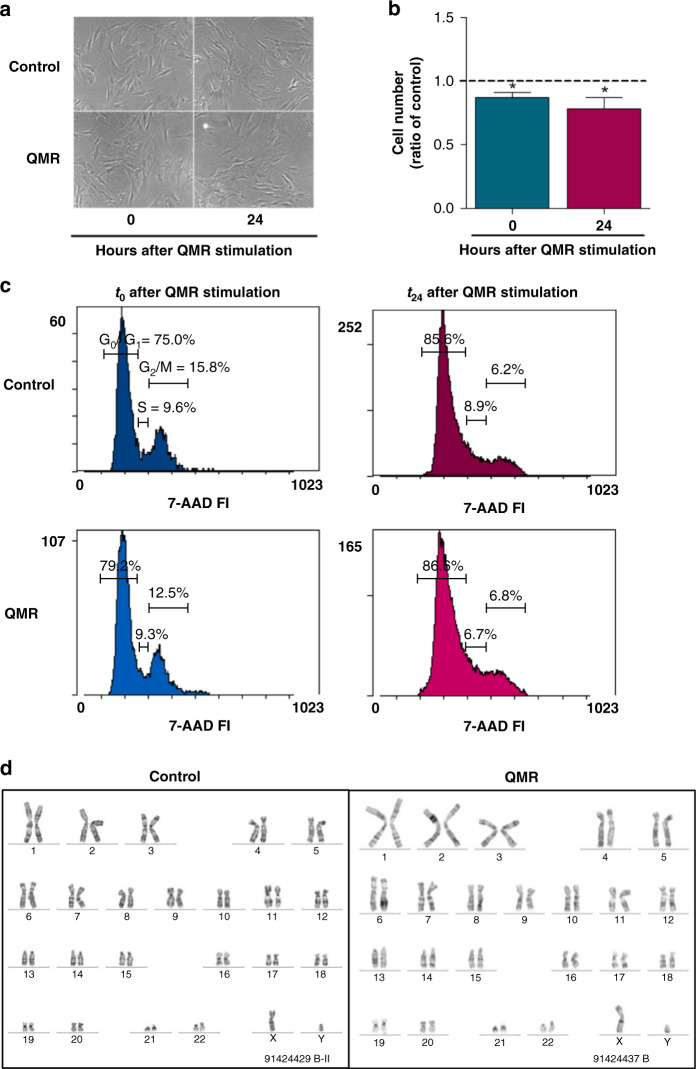
Effect of QMR stimulation on mesenchymal stromal cells. **a** Representative images of MSCs at 0 and 24 h after QMR exposure, acquired with an Axiovert 40 CFL inverted light microscope (Carl Zeiss, Oberkochen, Germany; ×10). **b** Cell viability was detected by trypan blue exclusion assay while the cell cycle (**c**) was analysed by flow cytometry at 0 and 24 h after QMR stimulation. Representative cytograms report the percentage of cells in each cell cycle phase (G_0_–G_1_, S, G_2_–M). **d** Demonstrative images of MSC karyotypes at baseline and after QMR exposure. Chromosomes in metaphase were G-banded using the G-Trypsin-Giemsa method. Results are expressed as mean ± SD of at least three independent experiments. *P < 0.05; QMR vs control.

To verify its safety, QMR treatment was applied to brain resident cells such as human adult astrocytes and mesenchymal stromal cells. MSCs are present in the major of human tissues including the perivascular niche of the adult brain, where potentially differentiate into mesodermal and neuroectodermal progeny [36]. The cell morphology, proliferation rate, cell cycle and MSC karyotype were examined at *t*_0_ and *t*_24_. Cultured MSCs showed a slight decrease in number after QMR stimulation (Fig. 2b), but this effect was not comparable to that observed on A172 glioblastoma cells (Fig. 1b); about 87 and 78% of MSCs viable cells were detected at *t*_0_ and *t*_24_, respectively. Moreover, stimulated cells showed no difference from controls in morphology or in the modulation of the cell cycle (Fig. 2a, c). The timing did not influence the final effect, as demonstrated by the near superimposability of results obtained at 0 and 24 h.

To further investigate the potential genotoxic effect of QMR on MSCs, the cells karyotypes after QMR treatment were examined. The stimulation did not affect the chromosome number or structure (Fig. 2d).

Due to the intrinsic difficulty of using primary astrocytes in our experimental model, only key experiments were performed on this cell line. As above, the severity of the QMR effect on cell morphology and viability (Supplementary Fig. 4a, b) was neither comparable to that shown on tumour cells nor differences in astrocytes karyotype after QMR treatment were observed (Supplementary Fig. 4c).

### QMR effects on the T98G and U87MG cell lines

To further verify the efficacy of QMR stimulation on more aggressive glioblastoma cell lines, key experiments were repeated with T98G and U87MG cells. The proliferation rate was reduced in both cell lines after QMR stimulation, but no major differences were observed in cell cycle progression and clonogenic ability (Supplementary Figs. 5 and 6).

**Fig. 3:**
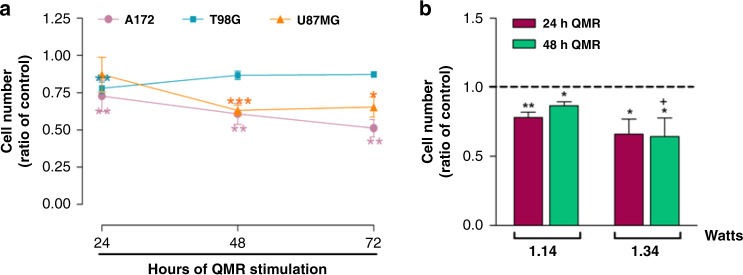
Effects of QMR on the A172, T98G and U87MG cell lines. **a** Cell viability was evaluated after 48 and 72 h of QMR stimulation of all cell lines. **b** The T98G cell line was treated with 15% greater power, and the proliferation rate was evaluated after 24 and 48 h of stimulation. The results are expressed as mean ± SD of two to four independent experiments. *P < 0.05, **P < 0.01, ***P < 0.001; QMR vs control. ^+^*P* < 0.05; 1.34 watts vs 1.14 watts.

Thus, cell viability was tested after 48 and 72 h of QMR stimulation of all cell lines. The proliferation rates of A172 and U87MG cells decreased significantly over time, with the U87MG cell line showing a 37% reduction in cell viability after 48 h that persisted over time (Fig. 3a). Unexpectedly, T98G cells displayed the opposite trend. To determine whether increased QMR potency would be more effective for the T98G cell line, the cells were treated with 15% increased power, and the proliferation rate was evaluated after 24 and 48 h stimulation. Cell viability was reduced by about 15–20% with the standard potency and about 35% after potency increase (Fig. 3b). Altogether, these data indicate the need to optimise QMR parameters for individual cell lines.

### QMR modulates proteomic fingerprint of A172 glioblastoma cell line

To better understand QMR MoA, the proteomic profiles of stimulated A172 and MSC cells were investigated at *t*_0_ and *t*_24_ by label-free quantitative proteomics. Unstimulated cells were used as comparative control (Supplementary Fig. 7).

**Fig. 4:**
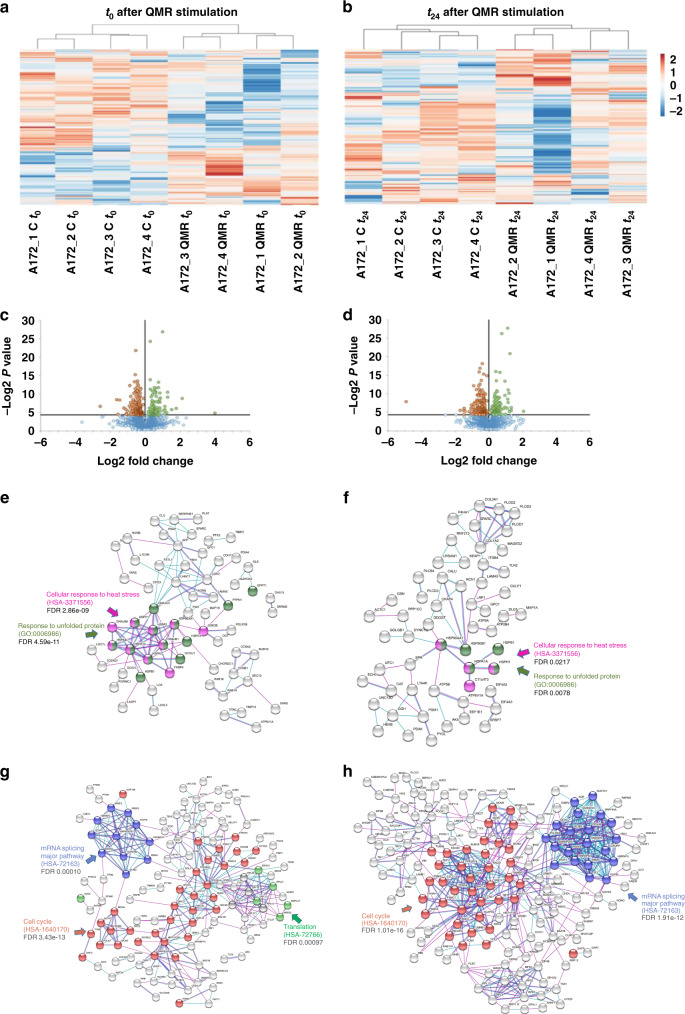
A172 proteomic profile after QMR stimulation. **a**, **b** Heatmaps of differential protein expression in A172 cells following QMR stimulation at *t*_0_ (**a**) and *t*_24_ (**b**). Red shading indicates upregulated proteins; blue shading indicates downregulated proteins. The threshold for significance was *P* < 0.05. **c**, **d** Volcano plots of differentially expressed proteins in QMR-stimulated vs unstimulated cells. Plots represent differential protein abundance in cells collected at 0 (**c**) and 24 h (**d**) after QMR exposure. The −log2 *P* value is plotted against the log2 fold change (QMR/control). The horizontal line represents the significance threshold in the logarithmic scale. **e**–**h** Networks of upregulated (**e**, **f**) and downregulated (**g**, **h**) proteins in QMR-stimulated A172 cells versus untreated cells at *t*_0_ (**e**–**g**) and *t*_24_ (**f**–**h**). Schematic views of known and predicted protein interactions according to the STRING database v. 11.0 (https://www.string-db.org). Only interactions with the medium confidence score (0.4) are shown. Each node represents a protein, and each line represents an interaction. The results derive from at least four independent experiments.

On A172 cells, a total of 5.104 proteins were successfully identified, of which 311 and 308 were significantly dysregulated at *t*_0_ and *t*_24_, respectively (Supplementary Table 2). The abundances of the differentially expressed proteins across the experimental groups were used for hierarchical clustering. The resulting heatmaps display two well-separated populations at both time points, demonstrating that QMR drastically interferes with the proteomic assets of cancer cells (Fig. 4a, b).

Proteins discriminating QMR-stimulated from unstimulated cells are visualised in Fig. 4c, d. At *t*_0_, 111 proteins in QMR-stimulated cells were significantly upregulated and 200 were downregulated (Fig. 4c). At *t*_24_, 103 upregulated and 205 downregulated proteins were detected (Fig. 4d). Only significant alterations are discussed below.

Gene ontology (GO) and pathway enrichment analyses were subsequently performed to identify the cellular processes most affected during QMR stimulation. Upregulated proteins were associated closely with the heat stress response, protein folding, and extracellular matrix (ECM) remodelling (Supplementary Fig. 8), suggesting that QMR act as a proteotoxic stimulus. In detail, QMR promoted a dramatic increase in the number of molecular chaperones (heat-shock proteins) involved in cytoprotective mechanisms that usually ensure proper protein folding and protein homoeostasis. Despite this intervention, exacerbated proteotoxic stimuli often lead to failure of the reparative response and, consequently, to cell death. The activation of the heat-shock pathway appeared to be drastic at the end of stimulation (Fig. 4e) and persisted over time (Fig. 4f). However, it was not sufficient to restore protein homoeostasis. Indeed, compared with untreated controls, QMR-stimulated cells showed prominent downregulation of key factors involved in protein translation, RNA processing and cell cycle-related pathways (Fig. 4g, h and Supplementary Fig. 9).

**Fig. 5:**
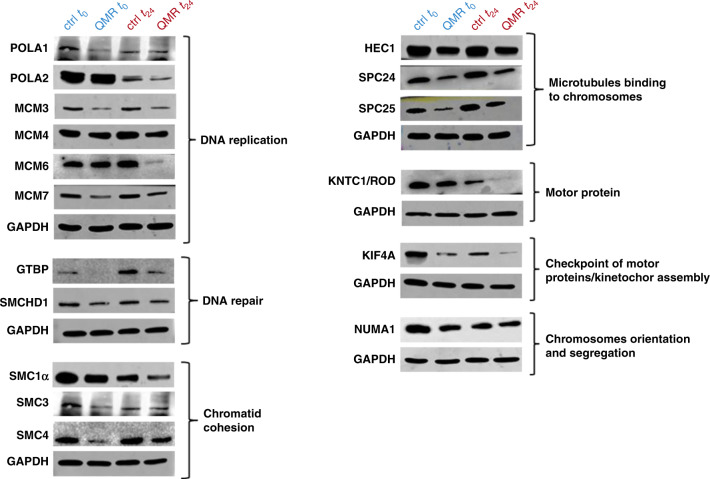
WB validation of proteomic analysis. Proteins with a higher *P* value were selected by proteomic raw data and confirmed by western blot assay. The expression of proteins involved in DNA replication, mitotic spindle assembly, and chromosome segregation was evaluated by WB at *t*_0_–*t*_24_ after QMR. GAPDH was used as a loading control.

Given cell cycle and proteomic results, further investigations on the mitotic machinery were performed. Key regulators of DNA replication and repair, mitotic spindle formation and stability, as well as chromosomes segregation were selected from proteomic raw data (Supplementary Table 2) and analysed by western blot. Representative images reported in Fig. 5 revealed a drastic interference of QMR treatment with all the main steps of the mitotic process. Indeed, stimulated cells presented lower levels of the DNA polymerases (POLA1-2) involved in the initiation of DNA replication, but also of the MCMs helicases responsible for DNA elongation. Injuries at this level could not be efficiently corrected since important factors implied in DNA repair (GTBP, SMCHD1) were also downregulated. Furthermore, QMR affected chromosomes cohesion (SMC1α-3–4), as well as microtubules binding to chromosomes, acting both on the microtubule-binding domain (Hec1) and centromere-binding domain (SPC24-25). This catastrophic condition inevitably led to aberrant chromosomes segregation and cell cycle arrest, as already demonstrated in Fig. 1.

### QMR effect on protein homoeostasis of MSC non-tumour cells

To clarify the different trends observed in healthy cells, quantitative proteomic analysis was then performed with MSCs (Supplementary Fig. 10 and Supplementary Table 3). The effect of QMR stimulation on MSCs was quite different if compared to tumour cells (Supplementary Fig. 10). Indeed, the activation of the heat-shock pathway was associated with ECM remodelling after QMR stimulation (Supplementary Fig. 10a, b). This alteration was almost completely restored after 24 h (Supplementary Fig. 10c, d) and thus did not interfere with the MSC proliferation rate. These results strongly suggest that QMR had a selective cytotoxic effect on cancer cells.

### QMR enhances the temozolomide effect in A172 glioblastoma cells

**Fig. 6:**
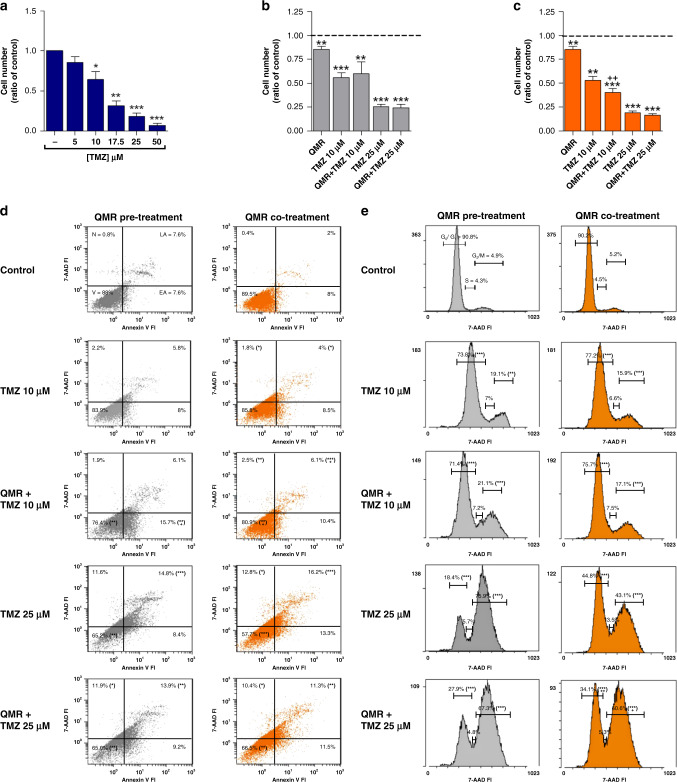
Effect of QMR and TMZ treatments on A172 cell viability, apoptosis and cell cycle. In total, 10–25 µM TMZ was administered for 144 h alone (**a**) or in combination with 24 h QMR (**b**–**e**). Grey histograms/dot plots represent TMZ administration after QMR stimulation, while orange histograms/dot plots are representative of the concomitant treatments. Cell viability (**a**–**c**) was evaluated by trypan blu exclusion assay, while apoptosis (**d**) and cell cycle (**e**) were monitored by flow cytometry. The results are expressed as mean ± SD of at least three independent experiments. *P < 0.05, **P < 0.01, ***P < 0.001; treatment vs control. ^+^*P* < 0.05, ^++^*P* < 0.01; combination of TMZ and QMR vs TMZ alone.

The main downsides of TMZ treatment are the onset of drug resistance mechanisms [37] and the severity of its side effects [38, 39]. Thus, new therapeutic strategies need to be identified to reduce TMZ dosage, thus improving patients’ quality of life. Here, association therapies were studied by treating cells with 10–25 µM TMZ administered together with or immediately after QMR exposure. Cell viability, apoptosis, and cell cycle were therefore analysed. TMZ concentrations were selected based on the half-maximal inhibitory concentration (Fig. 6a). Bliss analysis was performed to compare the efficacy of combined therapy with TMZ alone. Data revealed a different response to pre- or co-treatment strategy (Fig. 1b, c, respectively), Indeed, QMR and TMZ 10 µM co-administration resulted in a synergistic effect with a further 12% reduction of cell viability compared to TMZ alone (Fig. 6c). This data well correlates with apoptosis, where the combinatory therapy showed a reduction of viable cells associated with an increased rate of early apoptotic cells (Fig. 6d).

Concurrent exposure to QMR and TMZ at a final concentration of 25 µM triggered a greater alteration of cell cycle progression than did treatment with TMZ alone (Fig. 6e, co-treatment); the combination strategy induced a significant arrest of the cell cycle in the G_2_–M phase, reducing the percentage of G_0_–G_1_ cells (Fig. 6e, co-treatment). No major difference in the cell cycle was observed when TMZ was administered after QMR stimulation (Fig. 6e, pre-treatment).

## Discussion

GBM therapy includes maximal surgical resection followed by radiotherapy and chemotherapy with temozolomide [3–5]. Despite this multidisciplinary approach, glioblastoma patients continue to have a poor prognosis with a survival rate of 14–15 months [7]. This study was conducted to assess a promising technology for use alone or in combination with TMZ that could increase the success of glioblastoma therapy. Our efforts were directed at improving therapeutic efficacy and reduction of drug toxicity.

QMR treatment showed selective activity on glioblastoma cell lines, with a reduction of the cell proliferation rate associated with global proteomic perturbation that led to cell cycle arrest. Interestingly, only minor effects were detected in healthy mesenchymal and astrocyte cell lines, where the observed reduction of cell viability was not associated with cell cycle arrest or karyotype alteration.

Genetic material must be precisely duplicated in each cell division and equally distributed to daughter cells. Dysregulation at this level leads to chromosomal instability (CIN), defined as an increased rate of chromosomal changes [40]. CIN is considered a leading cause of tumour heterogeneity and instability. Oncogene-induced replication stress, breakage–fusion–bridge cycles induced by telomere dysfunction or translocation, and aberrant mitosis have been identified as possible causes of CIN [41–44]. Given their central roles in mitotic spindle assembly and chromosome segregation, centromeres and their associated kinetochores also may be implicated in the onset of CIN [45]. Their dysregulation results in chromosome missegregation, with consequent aneuploidy and micronucleus formation [46–48].

About 90% of solid tumours and many haematological malignancies display aneuploidy and DNA alterations, making CIN an essential hallmark of cancer cells that drives tumorigenesis and tumour progression [49, 50]. However, recent studies demonstrate that exacerbated CIN hampers cancer cell growth, presumably due to the excessive accumulation of CIN-induced genotoxic/proteotoxic stimuli [51, 52]. In this scenario, CIN exasperation represents an attractive opportunity for the selective targeting of cancer cells [53, 54]. In particular, Herman et al. [55] observed that healthy cells with robust kinetochore signalling easily survived the inhibition of the mitotic spindle checkpoint (BUB1B; the list of protein abbreviations is reported in Supplementary Table 4), whereas GBM cells with substantial chromosomal alterations did not [56]. They determined that the targeting of critical regulators of the cell division cascade leaves healthy cells with robust regulatory pathways largely unaffected, while compromised cells strongly resent any alteration.

In line with these observations, our data revealed that QMR-irradiated A172 glioblastoma cells underwent global proteomic perturbation that could affect tumour progression. In contrast, QMR-induced alterations were rapidly counteracted in healthy MSCs, resulting in a slight reduction in cell viability. In support of this evidence, A172 proteomic analysis revealed significant downregulation of several enzymes involved in the regulation of DNA replication (e.g., TYMS, DHFR, POLA1-2, MCMs, and ORC), chromatid cohesion (e.g., SMC1α, SMC3, SMC4), and post-replication DNA repair (e.g., GTBP, SMCHD1, RADs, MSH6, XRCC1 and DDB1). On the other hand, a pronounced reduction of proteins involved in the spindle assembly machinery and chromosome segregation was observed. Major effects on the proteins controlling kinetochore–microtubule (KT-MT) attachment stability were detected. Kinetochores are multiprotein complexes that act as microtubule-binding sites on each chromatid [57] and orchestrate chromosome movements and segregation, as part of the spindle assembly checkpoint signalling pathway [58]. The levels of centromere and kinetochore proteins are tightly regulated, and both depletion and overexpression of these proteins could result in chromosome abnormalities and cell death [59]. Although the kinetochore complex is composed of more than 100 proteins, the Mis12 complex, KNL1 and Ndc80 complex are considered the core attachment factors involved in KT-MT assembly [60, 61]. Intriguingly, QMR seems to work at this level, downregulating most components of the Ndc80 complex (NUF2, SPC24, SPC25, Hec1), as well as kinases involved in spindle checkpoint functions and chromosome segregation, such as BUB1B and KNTC1. Another possible cause of irregular spindle geometry is the failed migration of centrosomes around the nucleus, a process primarily driven by kinesin-5 and dynein [62]. These proteins are partly regulated by PCM1, NUMA1, DCTN3, KNTC1, PRC1, and KIF4A, all of which are downregulated in stimulated A172 cells. Chromosomes missegregation may result in aneuploidy and genomic instability, with subsequent onset of a process termed mitotic catastrophe [63]. In line with this assertion, the global perturbation detected in stimulated A172 cells was associated with the significant arrest of cell cycle progression in this study.

Emerging evidence suggests that cancer genomic instability often results in alarming levels of proteotoxic stress [64]. To avoid proteotoxicity, cells commonly activate the complex proteostasis network, which supervises protein synthesis, folding, and degradation, as well as conformational stability. Extrinsic and intrinsic factors may affect protein homoeostasis, generating proteotoxic stress [65]. To mitigate the misfolded protein burden, cells activate the adaptive heat-shock response [66]. However, whether proteostasis restoration fails, the permanence of proteotoxic stress can be deleterious, leading to cell death [65]. In this study, LC-MS results exposed a strong activation of the heat-shock pathway in QMR-irradiated A172 glioblastoma cells as an extreme attempt to revert protein injury. Nevertheless, the proliferation rate of stimulated cells was drastically reduced, highlighting the failure of this response. Equivalent activation in healthy MSCs was reverted efficiently, and protein alterations detected at the end of stimulation had been restored almost completely after 24 h. These data suggest that QMR has selective activity against cancer cells, making this technology an innovative option for GBM treatment.

The main objectives of this work were the understanding of QMR MoA and identifying the optimal QMR parameters for the selective targeting of glioblastoma. As already mentioned, conventional GBM therapy includes maximal surgical resection followed by radiotherapy and chemotherapy with TMZ. Unfortunately, TMZ treatment is characterised by the severity of its side effects and by the onset of drug resistance mechanisms. In this scenario, it appears essential the development of new therapeutic strategies to use alone or in combination with TMZ with the final goal of reducing drug dosage. Our assessment of the combined activity of QMR and TMZ demonstrated the ability of QMR to enhance TMZ efficacy. In particular, QMR and TMZ co-administration, with respect to pre-treatment strategy, resulted to be the best approach to possibly translate in clinical practice. Even if further investigations are needed to clarify QMR MoA and eventually confirm QMR efficacy in animal models, this study strongly suggests that QMR, alone or in combination with TMZ, might be a promising strategy to arrest glioblastoma progression.

## Supplementary information

Supplementary Figures-Tables

checklist

Supplementary Table 2

Supplementary Table 3

## Data Availability

Raw data generated at Mass Spectrometry and Proteomics Facility, Department of Cellular, Computational and Integrative Biology, CIBIO, Trento, supporting the findings of this study are available from RB and DP upon reasonable request. The other data supporting the findings of this study are available from the corresponding author (GA) upon reasonable request.
