# Supplementary material for: Selective cell cycle arrest in glioblastoma cell lines by quantum molecular resonance alone or in combination with temozolomide
Source: Br J Cancer. 2022 Jun 17;127(5):824–35. doi: 10.1038/s41416-022-01865-9 (PMC9427848; doi:10.1038/s41416-022-01865-9)
Supplement: Supplementary file 1 — Supplementary Figures-Tables [file 41416_2022_1865_MOESM1_ESM.docx]

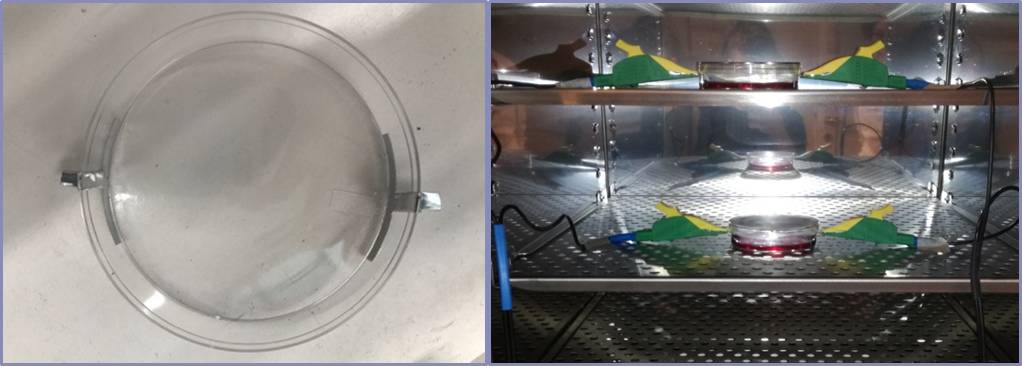
**Figure S1: Connection of custom-made electrodes (left) to the QMR generator prototype.**


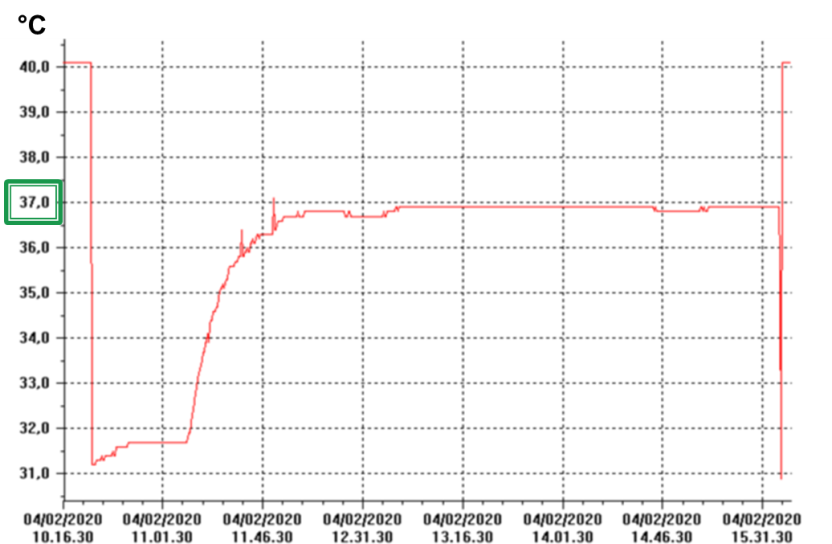


**Figure S2: Temperature increase during QMR stimulation.** To guarantee the appropriate cell growth conditions, the increase of temperature due to QMR irradiation was monitored by using a data-logger probe (iLog, Escort Scunthorpe, UK) placed inside the petri dish.


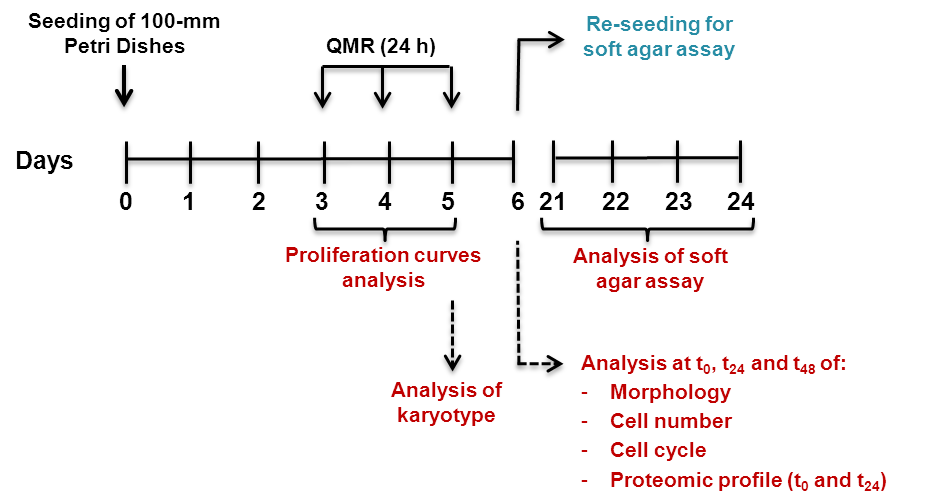


**Figure S3: Experimental setup**

**Table S1: Antibodies used in WB analysis**

| **Protein** | **Extensive name** | **Host Species** | **Datasheet MW (kDa)** | **Ab dilution** | **Manufacturing Company** |
| --- | --- | --- | --- | --- | --- |
| **ACTIN** | Actin | Mouse | 45 | 1:3000 | Abcam (AB170325) |
| **BUB1** | Mitotic checkpoint serine/threonine-protein kinase | Mouse | 122 | 1:250 | Santa Cruz (sc -47744) |
| **CYCLIN B1** | Cyclin B1 | Mouse | 60 | 1:250 | Santa Cruz (sc-245) |
| **GAPDH** | Glyceraldehyde-3-Phosphate Dehydrogenase | Mouse | 36 | 1:1000 | Cell Signaling (97166S) |
| **GTBP** | G/T mismatch-binding protein | Mouse | 160 | 1:100 | Santa Cruz (sc-271979) |
| **HEC1** | Highly expressed in cancer | Mouse | 76 | 1:1000 | Santa Cruz (sc-515550) |
| **KIF4A** | Chromosome-associated Kinesin | Rabbit | 140 | 1:1000 | Elabscience  (E-AB-63295-60) |
| **KNTC1/ROD** | Kinetochore-associated protein1 | Mouse | 240 | 1:250 | Santa Cruz (sc-81853) |
| **LAMIN B2** | Lamin B2 | Mouse | 67 | 1:500 | Santa Cruz (sc-56147) |
| **LAMININ γ1** | Laminin γ1 | Mouse | 215 | 1:500 | Santa Cruz (sc -17751) |
| **MCM3** | Mini-chromosome maintenance protein 3 | Mouse | 115 | 1:500 | Santa Cruz (sc -390480) |
| **MCM4** | Mini-chromosome maintenance protein 4 | Mouse | 100 | 1:500 | Santa Cruz (sc-48407) |
| **MCM6** | Mini-chromosome maintenance protein 6 | Mouse | 105 | 1:500 | Santa Cruz (sc-393618) |
| **MCM7** | Mini-chromosome maintenance protein 7 | Mouse | 88 | 1:500 | Santa Cruz (sc -9966) |
| **NUMA1** | Nuclear mitotic apparatus protein 1 | Mouse | 240 | 1:500 | Santa Cruz (sc-365532) |
| **POLA1** | DNA polymerase alpha catalytic subunit 1 | Mouse | 165 | 1:100 | Santa Cruz (sc-137021) |
| **POLA2** | DNA polymerase alpha catalytic subunit B | Mouse | 60-90 | 1:500 | Santa Cruz (sc-398255) |
| **SMC1α** | Structural maintenance of chromosomes 1α | Mouse | 155 | 1:500 | Santa Cruz (sc -393171) |
| **SMC3** | Structural maintenance of chromosomes 3 | Rabbit | 142-146 | 1:500 | ABclonal (A18402-20) |
| **SMC4** | Structural maintenance of chromosomes 4 | Rabbit | 180 | 1:500 | ABclonal (A3559-20) |
| **SMCHD1** | Structural Maintenance of Chromosomes Flexible Hinge Domain | Rabbit | 246 | 1:500 | Elabscience  (E-AB-61590-60) |
| **SPC24** | Kinetochore protein Spc24 | Rabbit | 22 | 1:1000 | Elabscience  (E-AB-66870-60) |
| **SPC25** | Kinetochore protein Spc25 | Rabbit | 26 | 1:500 | ABclonal (A10653-20) |

**
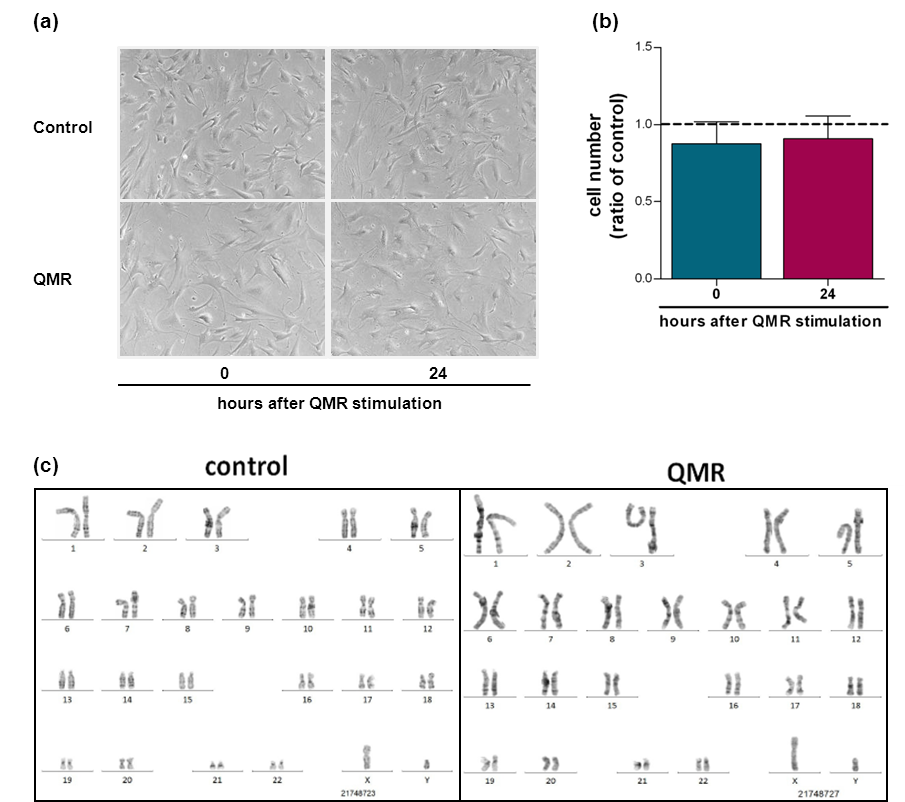
**

**Figure S4: Effect of QMR stimulation on healthy human astrocytes.** (**a**) Representative images of astrocytes at 0 and 24 h after QMR exposure, acquired with an Axiovert 40 CFL inverted light microscope (Carl Zeiss, Oberkochen, Germany; 10×). (**b**) Cell viability detected by trypan blue exclusion assay. (**c**) Representative images of astrocytes karyotypes at baseline and after QMR exposure. Chromosomes in metaphase were G-banded using the G-Trypsin-Giemsa method. Results are expressed as mean ± SD of at least four independent experiments.


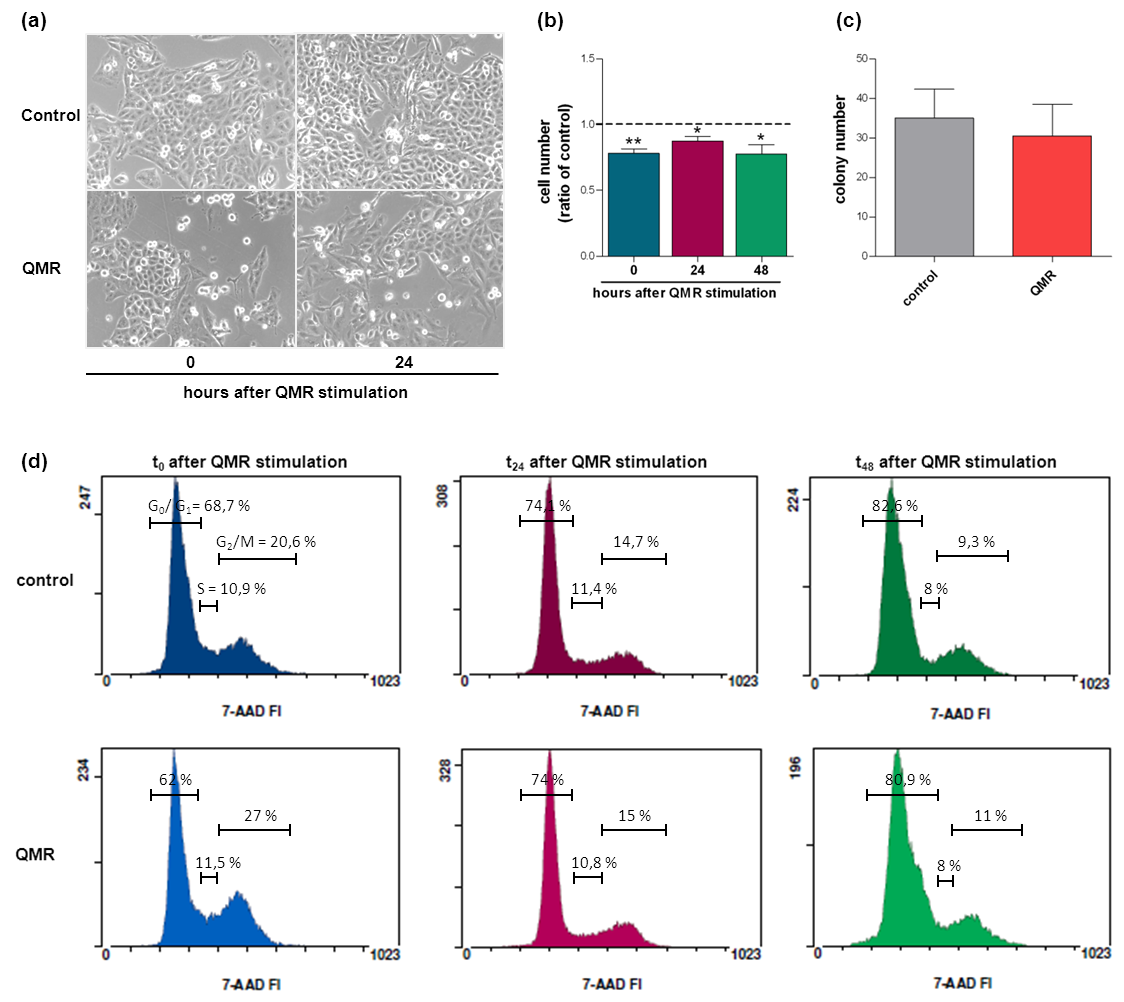


**Figure S5: Effect of QMR stimulation on T98G glioblastoma cells.** (**a**) Representative images of T98G at 0 and 24 h after QMR exposure, acquired with an Axiovert 40 CFL inverted light microscope (Carl Zeiss, Oberkochen, Germany; 10×). (**b**) Cell viability was detected by trypan blue exclusion assay, while T98G colonies **(c)** were labeled with calcein and counted under an inverted light microscope. Representative cell cycle cytograms (**d**) detected by flow cytometry at 0, 24 and 48 h after QMR stimulation. Results are expressed as mean ± SD of at least three independent experiments. *p<0.05, **p<0.01; QMR vs control.


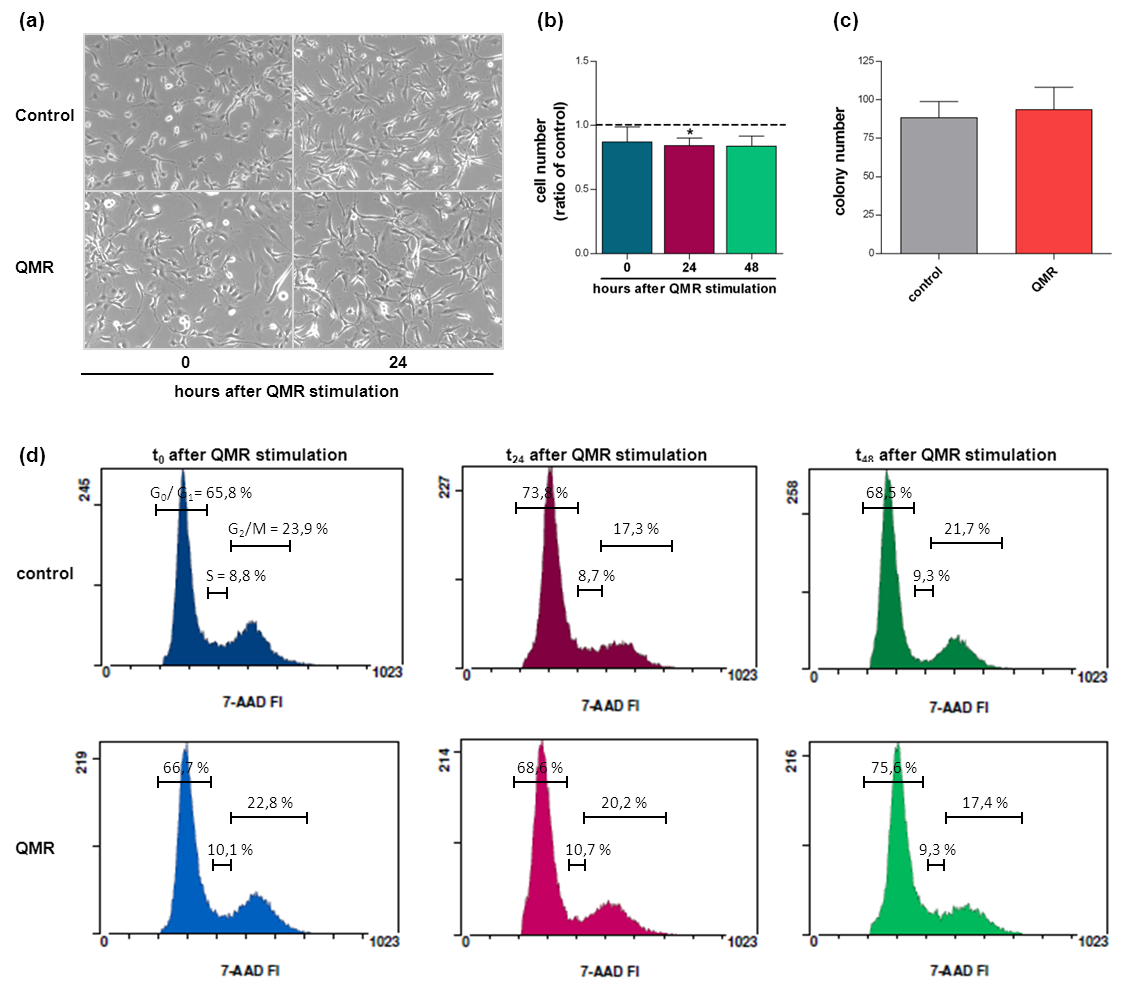


**Figure S6: Effect of QMR stimulation on U87MG glioblastoma cells.** (**a**) Representative images of U87MG at 0 and 24 h after QMR exposure, acquired with an Axiovert 40 CFL inverted light microscope (Carl Zeiss, Oberkochen, Germany; 10×). (**b**) Cell viability was detected by trypan blue exclusion assay, while T98G colonies **(c)** were labeled with calcein and counted under an inverted light microscope. Representative cell cycle cytograms (**d**) detected by flow cytometry at 0, 24 and 48 h after QMR stimulation. Results are expressed as mean ± SD of at least three independent experiments. *p < 0.05; QMR vs control.


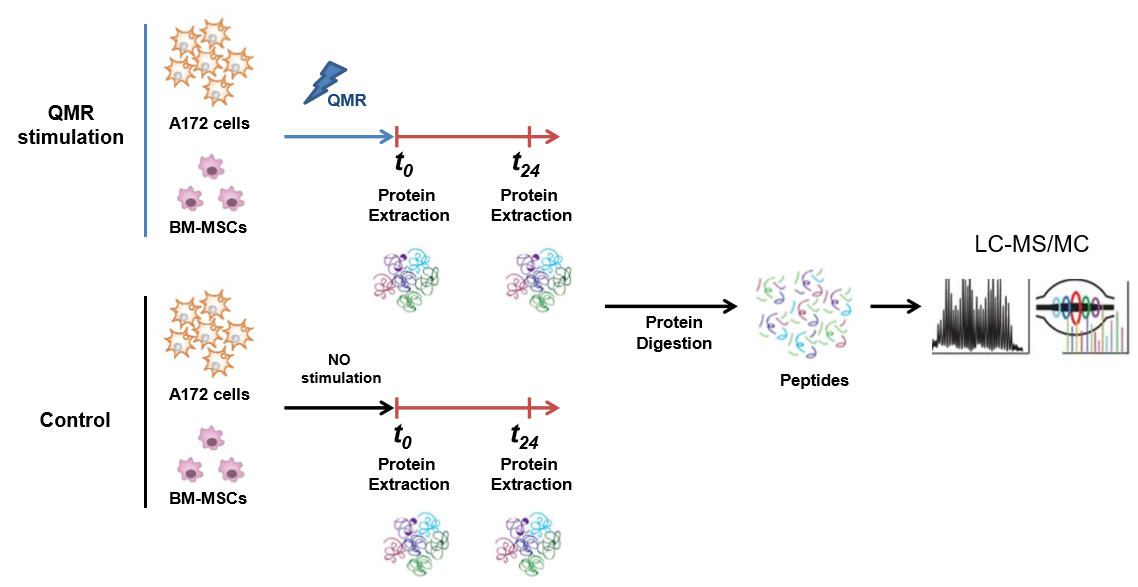


**Figure S7: Workflow for quantitative proteomic measurement of QMR-stimulated cells at t_0_ and t_24_.**

**
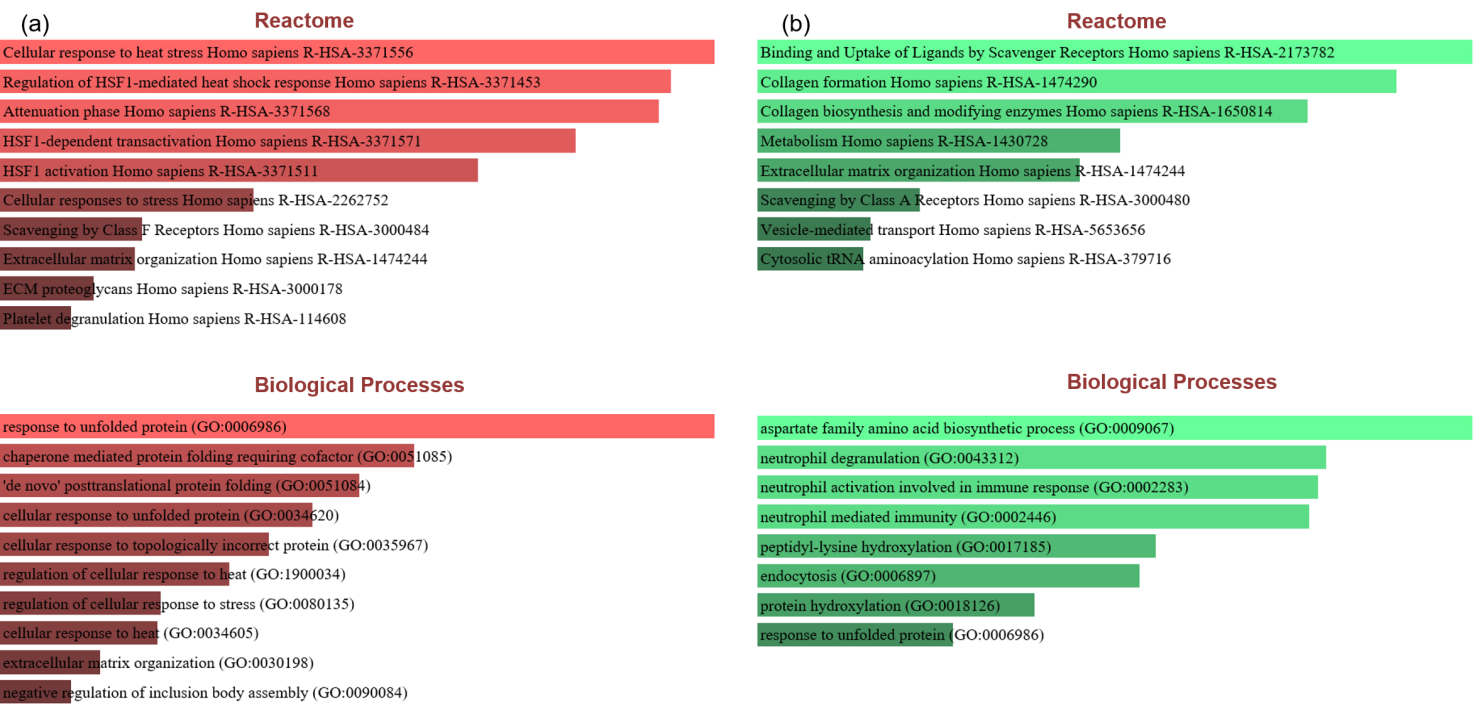
**

**Figure S8: Enrichment analysis performed on up-regulated proteins in QMR-stimulated A172 cells versus untreated cells at t_0_ (a) and t_24_ (b).** Analysis was performed with Reactome 2016 (upper panels) and GO Biological Process 2018 (lower panels) using the Enrichr online tool. The top 10 enriched protein sets are shown for each category (adj p-value &lt; 0.05). The length and the color gradient of bars (red and green for t_0_ and t_24_, respectively) indicate significance (−log10 p value).

**
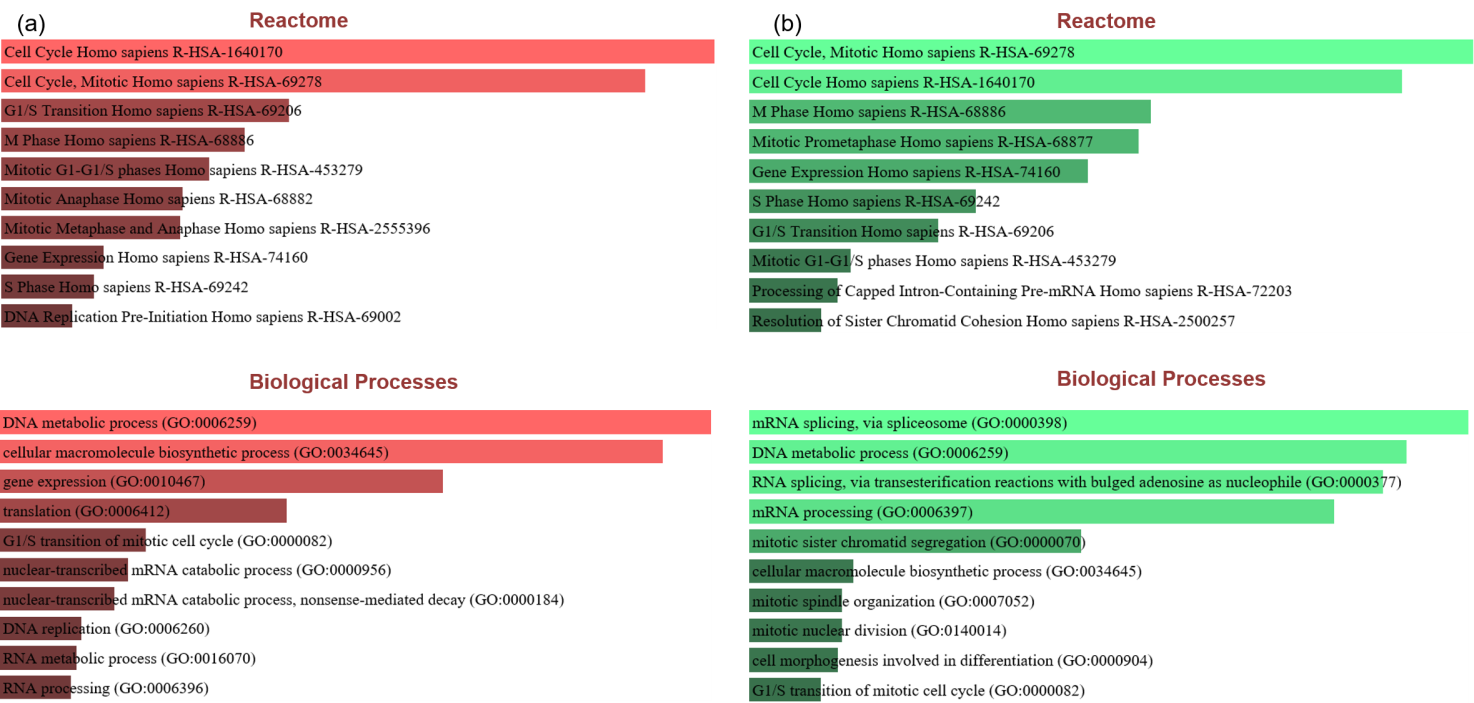
**

**Figure S9: Enrichment analysis performed on down-regulated proteins in QMR-stimulated A172 cells versus untreated cells at t_0_ (a) and t_24_ (b).** Analysis was performed with Reactome 2016 (upper panels) and GO Biological Process 2018 (lower panels) using the Enrichr online tool. The top 10 enriched protein sets are shown for each category (adj p-value &lt; 0.05). The length and the color gradient of bars (red and green for t_0_ and t_24_, respectively) indicate significance (−log10 p value).


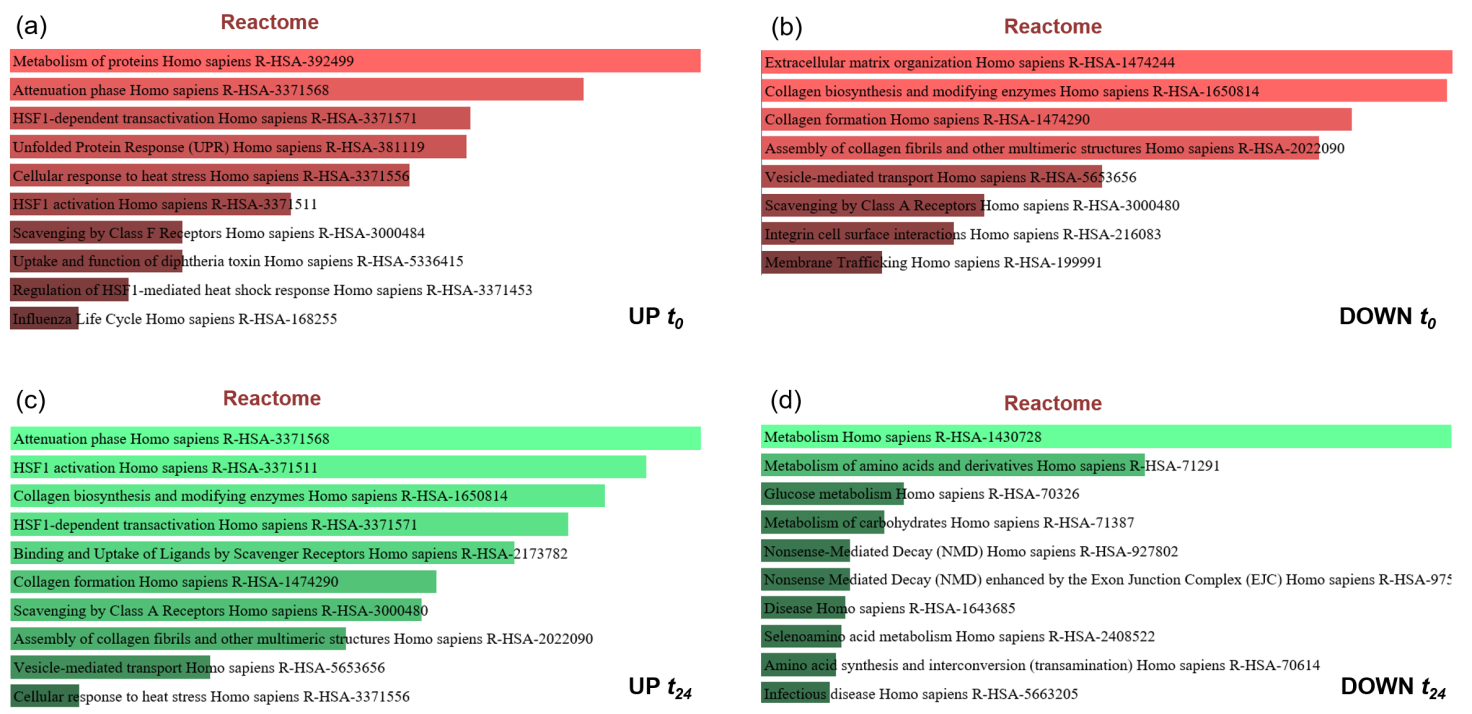


**Figure S10: Pathway analysis of differentially expressed proteins in QMR-stimulated MSCs versus untreated cells at t_0_ and t_24_.** Enrichment analysis was performed on up-regulated (a, c) and down-regulated (b, d) terms using Reactome 2016. The top 10 enriched protein sets are shown for each category (adj p-value &lt; 0.05). The length and the color gradient of bars indicate significance (−log10 p value).

**Table S4: Protein abbreviations**

| **Protein** | **Extensive name** |
| --- | --- |
| **BUB1B** | Mitotic checkpoint serine/threonine-protein kinase |
| **CHD3/4** | Chromodomain-helicase-DNA-binding protein 3/4 |
| **DCTN3** | Dynactin subunit 3 |
| **DDB1** | DNA damage-binding protein 1 |
| **DHX36** | ATP-dependent DNA/RNA helicase DHX36 |
| **DHFR** | Dihydrofolate reductase |
| **ESCO1** | Establishment of sister chromatid cohesion N-acetyltransferase 1 |
| **GTBP** | G/T mismatch-binding protein |
| **HEC1** | Highly expressed in cancer |
| **KIF4A** | Chromosome-associated kinesin KIF4A |
| **KNL1** | Kinetochore null protein 1 |
| **KNTC1** | Kinetochore-associated protein 1 |
| **MCMs** | minichromosome maintenance protein complex |
| **Mis12** | Protein MIS12 homolog |
| **MSH6** | mutS homolog 6 ([mismatch repair](https://en.wikipedia.org/wiki/DNA_mismatch_repair) protein) |
| **Ndc80** | Kinetochore protein NDC80 homolog |
| **NUF2** | Kinetochore protein Nuf2 |
| **NUMA1** | Nuclear mitotic apparatus protein 1 |
| **ORC** | origin recognition complex |
| **PCM1** | Pericentriolar material 1 |
| **POLA1/2** | DNA polymerase alpha catalytic subunit 1/B |
| **PRC1** | Protein Regulator of cytokinesis 1 |
| **RADs** | Cell cycle checkpoint protein |
| **SMCHD1** | Structural Maintenance of Chromosomes Flexible Hinge Domain |
| **SPC24/25** | Kinetochore protein Spc24/25 |
| **TYMS** | Thymidylate synthase |
| **XRCC1** | X-ray repair cross-complementing protein 1 (DNA repair protein) |
