# Supplementary material for: Selective cell cycle arrest in glioblastoma cell lines by quantum molecular resonance alone or in combination with temozolomide
Source: Br J Cancer. 2022 Jun 17;127(5):824–35. doi: 10.1038/s41416-022-01865-9 (PMC9427848; doi:10.1038/s41416-022-01865-9)
Supplement: Supplementary file 3 — Supplementary Table 2 [file 41416_2022_1865_MOESM3_ESM.pdf]

Supplementary Table 2: List of proteins identified as differentially expressed between QMR-stimulated A172 cells and untreated A172 cells.
For each protein, Master Protein Accessions number (Uniprot), Description,Prot. Fold change (QMR-stimulated cells/Untreated cells) and p-value are reported. Prot. Fold change are given as log2-fold changes, while p-value as -log2 (p-value). Statistical confidence was assessed when p-value < 0.05, corresponding to -log2 (p-value) of 4.32.

Master Protein Accessions	Description	Prot. Fold change (QMR/CON)	p-value
P0DMV8	Heat shock 70 kDa protein 1A OS=Homo sapiens OX=9606 GN=HSPA1A PE=1 SV=1	1,100507731	27,67607
Q92598	Heat shock protein 105 kDa OS=Homo sapiens OX=9606 GN=HSPH1 PE=1 SV=1	0,766016297	26,29133
P05067	Amyloid-beta precursor protein OS=Homo sapiens OX=9606 GN=APP PE=1 SV=3	1,238685396	20,88259
Q86VI3	Ras GTPase-activating-like protein IQGAP3 OS=Homo sapiens OX=9606 GN=IQGAP3 PE=1 SV=2	-0,409947188	18,13224
Q70J99	Protein unc-13 homolog D OS=Homo sapiens OX=9606 GN=UNC13D PE=1 SV=1	-0,526101103	16,8476
P07900	Heat shock protein HSP 90-alpha OS=Homo sapiens OX=9606 GN=HSP90AA1 PE=1 SV=5	0,408210295	15,97805
Q9UIG0	Tyrosine-protein kinase BAZ1B OS=Homo sapiens OX=9606 GN=BAZ1B PE=1 SV=2	-0,585779337	15,83447
P19022	Cadherin-2 OS=Homo sapiens OX=9606 GN=CDH2 PE=1 SV=4	0,654397699	15,78797
Q14980	Nuclear mitotic apparatus protein 1 OS=Homo sapiens OX=9606 GN=NUMA1 PE=1 SV=2	-0,324504926	15,34378
Q15149	Plectin OS=Homo sapiens OX=9606 GN=PLEC PE=1 SV=3	-0,145333337	14,86967
Q14573	Inositol 1,4,5-trisphosphate receptor type 3 OS=Homo sapiens OX=9606 GN=ITPR3 PE=1 SV=2	-0,46701753	13,58753
P11021	Endoplasmic reticulum chaperone BiP OS=Homo sapiens OX=9606 GN=HSPA5 PE=1 SV=2	0,424757819	13,48737
Q9UI42	Carboxypeptidase A4 OS=Homo sapiens OX=9606 GN=CPA4 PE=1 SV=2	0,943419525	13,35155
Q5UIP0	Telomere-associated protein RIF1 OS=Homo sapiens OX=9606 GN=RIF1 PE=1 SV=2	-0,529760454	12,56023
P05997	Collagen alpha-2(V) chain OS=Homo sapiens OX=9606 GN=COL5A2 PE=1 SV=3	-0,628981492	12,1749
O14578	Citron Rho-interacting kinase OS=Homo sapiens OX=9606 GN=CIT PE=1 SV=2	-0,922172648	12,1157
P38159	RNA-binding motif protein, X chromosome OS=Homo sapiens OX=9606 GN=RBMX PE=1 SV=3	-0,590758409	11,28239
Q8N6H7	ADP-ribosylation factor GTPase-activating protein 2 OS=Homo sapiens OX=9606 GN=ARFGAP2 PE=1 SV=1	-0,742148552	11,15986
Q9NZV1	Cysteine-rich motor neuron 1 protein OS=Homo sapiens OX=9606 GN=CRIM1 PE=1 SV=1	1,123502861	11,05439
Q03252	Lamin-B2 OS=Homo sapiens OX=9606 GN=LMNB2 PE=1 SV=4	-0,40085882	10,9707
Q16881	Thioredoxin reductase 1, cytoplasmic OS=Homo sapiens OX=9606 GN=TXNRD1 PE=1 SV=3	0,450778428	10,83938
O94925	Glutaminase kidney isoform, mitochondrial OS=Homo sapiens OX=9606 GN=GLS PE=1 SV=1	0,47776665	10,57246
Q8N3D4	EH domain-binding protein 1-like protein 1 OS=Homo sapiens OX=9606 GN=EHBP1L1 PE=1 SV=2	-0,520139427	10,5068
Q04727	Transducin-like enhancer protein 4 OS=Homo sapiens OX=9606 GN=TLE4 PE=1 SV=3	-0,571111414	10,3133
Q9NTJ3	Structural maintenance of chromosomes protein 4 OS=Homo sapiens OX=9606 GN=SMC4 PE=1 SV=2	-0,298907275	10,20581
Q96FV9	THO complex subunit 1 OS=Homo sapiens OX=9606 GN=THOC1 PE=1 SV=1	1,176746538	10,10459
Q9Y4L1	Hypoxia up-regulated protein 1 OS=Homo sapiens OX=9606 GN=HYOU1 PE=1 SV=1	0,355835752	9,96849
Q6P2Q9	Pre-mRNA-processing-splicing factor 8 OS=Homo sapiens OX=9606 GN=PRPF8 PE=1 SV=2	-0,213324703	9,87812
P26358	DNA (cytosine-5)-methyltransferase 1 OS=Homo sapiens OX=9606 GN=DNMT1 PE=1 SV=2	-0,384676926	9,800383
P31943	Heterogeneous nuclear ribonucleoprotein H OS=Homo sapiens OX=9606 GN=HNRNPH1 PE=1 SV=4	-0,526676355	9,774646
Q9Y4P3	Transducin beta-like protein 2 OS=Homo sapiens OX=9606 GN=TBL2 PE=1 SV=1	-0,693148944	9,676369
Q9NRZ9	Lymphoid-specific helicase OS=Homo sapiens OX=9606 GN=HELLS PE=1 SV=1	-0,650116748	9,61945
Q9UQE7	Structural maintenance of chromosomes protein 3 OS=Homo sapiens OX=9606 GN=SMC3 PE=1 SV=2	-0,33016746	9,569587
Q8IVT2	Mitotic interactor and substrate of PLK1 OS=Homo sapiens OX=9606 GN=MISP PE=1 SV=1	-0,581359229	9,494459
P26022	Pentraxin-related protein PTX3 OS=Homo sapiens OX=9606 GN=PTX3 PE=1 SV=3	0,947085739	9,489922
Q96JB5	CDK5 regulatory subunit-associated protein 3 OS=Homo sapiens OX=9606 GN=CDK5RAP3 PE=1 SV=2	-0,609344397	9,485461
P25205	DNA replication licensing factor MCM3 OS=Homo sapiens OX=9606 GN=MCM3 PE=1 SV=3	-0,327887062	9,47227
Q9H0D6	5'-3' exoribonuclease 2 OS=Homo sapiens OX=9606 GN=XRN2 PE=1 SV=1	-0,478846169	9,434629
O95466	Formin-like protein 1 OS=Homo sapiens OX=9606 GN=FMNL1 PE=1 SV=3	-0,462835121	9,392634
P13667	Protein disulfide-isomerase A4 OS=Homo sapiens OX=9606 GN=PDIA4 PE=1 SV=2	0,319879941	9,332209
P07384	Calpain-1 catalytic subunit OS=Homo sapiens OX=9606 GN=CAPN1 PE=1 SV=1	-0,333787959	9,080745
P35052	Glypican-1 OS=Homo sapiens OX=9606 GN=GPC1 PE=1 SV=2	0,496121937	9,054547
Q9UHD1	Cysteine and histidine-rich domain-containing protein 1 OS=Homo sapiens OX=9606 GN=CHORDC1 PE=1 SV=2	0,613412784	8,99118
Q96T51	RUN and FYVE domain-containing protein 1 OS=Homo sapiens OX=9606 GN=RUFY1 PE=1 SV=2	0,420934982	8,910553
P11047	Laminin subunit gamma-1 OS=Homo sapiens OX=9606 GN=LAMC1 PE=1 SV=3	0,322761281	8,848569
P20700	Lamin-B1 OS=Homo sapiens OX=9606 GN=LMNB1 PE=1 SV=2	-0,298189085	8,763174
O95757	Heat shock 70 kDa protein 4L OS=Homo sapiens OX=9606 GN=HSPA4L PE=1 SV=3	0,540999649	8,693131
P49915	GMP synthase [glutamine-hydrolyzing] OS=Homo sapiens OX=9606 GN=GMPS PE=1 SV=1	-0,352366265	8,646388
Q9UBP9	PTB domain-containing engulfment adapter protein 1 OS=Homo sapiens OX=9606 GN=GULP1 PE=1 SV=1	1,109645531	8,583771
P07602	Prosaposin OS=Homo sapiens OX=9606 GN=PSAP PE=1 SV=2	0,460244073	8,583546
P52701	DNA mismatch repair protein Msh6 OS=Homo sapiens OX=9606 GN=MSH6 PE=1 SV=2	-0,306641242	8,392816
Q99426	Tubulin-folding cofactor B OS=Homo sapiens OX=9606 GN=TBCB PE=1 SV=2	-0,648051479	8,350125
Q15021	Condensin complex subunit 1 OS=Homo sapiens OX=9606 GN=NCAPD2 PE=1 SV=3	-0,284244895	8,321293
P33991	DNA replication licensing factor MCM4 OS=Homo sapiens OX=9606 GN=MCM4 PE=1 SV=5	-0,330567775	8,315138
P22626	Heterogeneous nuclear ribonucleoproteins A2/B1 OS=Homo sapiens OX=9606 GN=HNRNPA2B1 PE=1 SV=2	-0,421075673	8,272437
Q9Y266	Nuclear migration protein nudC OS=Homo sapiens OX=9606 GN=NUDC PE=1 SV=1	-0,446708947	8,251116
Q8N5S9	Calcium/calmodulin-dependent protein kinase kinase 1 OS=Homo sapiens OX=9606 GN=CAMKK1 PE=1 SV=2	-0,712383799	8,247057
Q14683	Structural maintenance of chromosomes protein 1A OS=Homo sapiens OX=9606 GN=SMC1A PE=1 SV=2	-0,324807975	8,105624
P31040	Succinate dehydrogenase [ubiquinone] flavoprotein subunit, mitochondrial OS=Homo sapiens OX=9606 GN=SDHA PE=1 S	-0,508817672	8,052184
Q8IVF2	Protein AHNAK2 OS=Homo sapiens OX=9606 GN=AHNAK2 PE=1 SV=2	-0,143105355	8,030647
P32004	Neural cell adhesion molecule L1 OS=Homo sapiens OX=9606 GN=L1CAM PE=1 SV=2	0,390273196	8,030518
Q9Y618	Nuclear receptor corepressor 2 OS=Homo sapiens OX=9606 GN=NCOR2 PE=1 SV=3	-0,466903993	7,98653
Q96DT5	Dynein heavy chain 11, axonemal OS=Homo sapiens OX=9606 GN=DNAH11 PE=1 SV=4	-0,66378416	7,912597
Q9Y6R4	Mitogen-activated protein kinase kinase kinase 4 OS=Homo sapiens OX=9606 GN=MAP3K4 PE=1 SV=2	-4,931300587	7,905637
P35555	Fibrillin-1 OS=Homo sapiens OX=9606 GN=FBN1 PE=1 SV=4	0,31127404	7,812346
Q12841	Follistatin-related protein 1 OS=Homo sapiens OX=9606 GN=FSTL1 PE=1 SV=1	0,495163343	7,780212
Q5SSJ5	Heterochromatin protein 1-binding protein 3 OS=Homo sapiens OX=9606 GN=HP1BP3 PE=1 SV=1	-0,605809655	7,729926
Q14181	DNA polymerase alpha subunit B OS=Homo sapiens OX=9606 GN=POLA2 PE=1 SV=2	-0,765037388	7,702432
O60814	Histone H2B type 1-K OS=Homo sapiens OX=9606 GN=HIST1H2BK PE=1 SV=3	-0,849255497	7,66532
P26373	60S ribosomal protein L13 OS=Homo sapiens OX=9606 GN=RPL13 PE=1 SV=4	-0,600692635	7,654261
Q9BXF6	Rab11 family-interacting protein 5 OS=Homo sapiens OX=9606 GN=RAB11FIP5 PE=1 SV=1	-0,46917322	7,651168
P33993	DNA replication licensing factor MCM7 OS=Homo sapiens OX=9606 GN=MCM7 PE=1 SV=4	-0,323126327	7,632501
O60264	SWI/SNF-related matrix-associated actin-dependent regulator of chromatin subfamily A member 5 OS=Homo sapiens OX=	-0,342771568	7,625586
P49757	Protein numb homolog OS=Homo sapiens OX=9606 GN=NUMB PE=1 SV=2	0,637834872	7,589886
Q86XP3	ATP-dependent RNA helicase DDX42 OS=Homo sapiens OX=9606 GN=DDX42 PE=1 SV=1	-0,394674035	7,57431
Q14839	Chromodomain-helicase-DNA-binding protein 4 OS=Homo sapiens OX=9606 GN=CHD4 PE=1 SV=2	-0,263528369	7,572811
P16035	Metalloproteinase inhibitor 2 OS=Homo sapiens OX=9606 GN=TIMP2 PE=1 SV=2	1,144000206	7,568129
P42892	Endothelin-converting enzyme 1 OS=Homo sapiens OX=9606 GN=ECE1 PE=1 SV=2	-0,462546009	7,511679
Q14320	Protein FAM50A OS=Homo sapiens OX=9606 GN=FAM50A PE=1 SV=2	0,589475059	7,488025
P31153	S-adenosylmethionine synthase isoform type-2 OS=Homo sapiens OX=9606 GN=MAT2A PE=1 SV=1	-0,42751927	7,449081
Q9Y3P9	Rab GTPase-activating protein 1 OS=Homo sapiens OX=9606 GN=RABGAP1 PE=1 SV=3	0,389270418	7,432667
O95361	Tripartite motif-containing protein 16 OS=Homo sapiens OX=9606 GN=TRIM16 PE=1 SV=3	0,400022992	7,41638
P04114	Apolipoprotein B-100 OS=Homo sapiens OX=9606 GN=APOB PE=1 SV=2	-0,816849389	7,380933
P46779	60S ribosomal protein L28 OS=Homo sapiens OX=9606 GN=RPL28 PE=1 SV=3	-0,671175268	7,37651

A6NHR9	Structural maintenance of chromosomes flexible hinge domain-containing protein 1 OS=Homo sapiens OX=9606 GN=SMC	-0,315285313	7,366322
Q4J6C6	Prolyl endopeptidase-like OS=Homo sapiens OX=9606 GN=PREPL PE=1 SV=1	0,620301065	7,354225
P20908	Collagen alpha-1(V) chain OS=Homo sapiens OX=9606 GN=COL5A1 PE=1 SV=3	-0,395341713	7,298596
P16949	Stathmin OS=Homo sapiens OX=9606 GN=STMN1 PE=1 SV=3	-0,904083096	7,173686
Q9UJY1	Heat shock protein beta-8 OS=Homo sapiens OX=9606 GN=HSPB8 PE=1 SV=1	0,710554028	7,133103
Q9NSD9	Phenylalanine--tRNA ligase beta subunit OS=Homo sapiens OX=9606 GN=FARSB PE=1 SV=3	-0,367435131	7,126147
O75534	Cold shock domain-containing protein E1 OS=Homo sapiens OX=9606 GN=CSDE1 PE=1 SV=2	-0,302248878	7,114836
P30405	Peptidyl-prolyl cis-trans isomerase F, mitochondrial OS=Homo sapiens OX=9606 GN=PPIF PE=1 SV=1	-1,037115513	6,98212
P52272	Heterogeneous nuclear ribonucleoprotein M OS=Homo sapiens OX=9606 GN=HNRNPM PE=1 SV=3	-0,238405771	6,878542
P00374	Dihydrofolate reductase OS=Homo sapiens OX=9606 GN=DHFR PE=1 SV=2	-0,685478459	6,866056
Q92900	Regulator of nonsense transcripts 1 OS=Homo sapiens OX=9606 GN=UPF1 PE=1 SV=2	0,26707213	6,841371
P04792	Heat shock protein beta-1 OS=Homo sapiens OX=9606 GN=HSPB1 PE=1 SV=2	0,388099715	6,830711
P54577	Tyrosine--tRNA ligase, cytoplasmic OS=Homo sapiens OX=9606 GN=YARS PE=1 SV=4	0,346036528	6,792228
P55735	Protein SEC13 homolog OS=Homo sapiens OX=9606 GN=SEC13 PE=1 SV=3	0,427295907	6,773318
Q96PK6	RNA-binding protein 14 OS=Homo sapiens OX=9606 GN=RBM14 PE=1 SV=2	-0,385174827	6,760901
P32322	Pyrroline-5-carboxylate reductase 1, mitochondrial OS=Homo sapiens OX=9606 GN=PYCR1 PE=1 SV=2	0,611417394	6,650592
P36551	Oxygen-dependent coproporphyrinogen-III oxidase, mitochondrial OS=Homo sapiens OX=9606 GN=CPOX PE=1 SV=3	0,568181193	6,646307
P05121	Plasminogen activator inhibitor 1 OS=Homo sapiens OX=9606 GN=SERPINE1 PE=1 SV=1	0,420056329	6,622644
Q14315	Filamin-C OS=Homo sapiens OX=9606 GN=FLNC PE=1 SV=3	0,182015141	6,602201
Q13217	DnaJ homolog subfamily C member 3 OS=Homo sapiens OX=9606 GN=DNAJC3 PE=1 SV=1	0,528566607	6,578384
Q96T88	E3 ubiquitin-protein ligase UHRF1 OS=Homo sapiens OX=9606 GN=UHRF1 PE=1 SV=1	-0,377354058	6,574862
Q6P179	Endoplasmic reticulum aminopeptidase 2 OS=Homo sapiens OX=9606 GN=ERAP2 PE=1 SV=2	-0,470308287	6,500147
O14777	Kinetochore protein NDC80 homolog OS=Homo sapiens OX=9606 GN=NDC80 PE=1 SV=1	-0,397119312	6,449328
P48741	#N/D	0,942310795	6,429107
O43143	Pre-mRNA-splicing factor ATP-dependent RNA helicase DHX15 OS=Homo sapiens OX=9606 GN=DHX15 PE=1 SV=2	0,324489304	6,418941
P49841	Glycogen synthase kinase-3 beta OS=Homo sapiens OX=9606 GN=GSK3B PE=1 SV=2	0,643950046	6,417271
P54652	#N/D	0,591202061	6,401597
O75935	Dynactin subunit 3 OS=Homo sapiens OX=9606 GN=DCTN3 PE=1 SV=1	-0,642088185	6,396969
Q6VY07	Phosphofurin acidic cluster sorting protein 1 OS=Homo sapiens OX=9606 GN=PACS1 PE=1 SV=2	-0,549644476	6,392322
Q9H0W8	Protein SMG9 OS=Homo sapiens OX=9606 GN=SMG9 PE=1 SV=1	-0,934562845	6,382462
O15294	UDP-N-acetylglucosamine--peptide N-acetylglucosaminyltransferase 110 kDa subunit OS=Homo sapiens OX=9606 GN=OG	0,322273888	6,357134
Q15035	Translocating chain-associated membrane protein 2 OS=Homo sapiens OX=9606 GN=TRAM2 PE=1 SV=1	1,332234709	6,306937
Q96AC1	Fermitin family homolog 2 OS=Homo sapiens OX=9606 GN=FERMT2 PE=1 SV=1	0,293374192	6,282414
P38606	V-type proton ATPase catalytic subunit A OS=Homo sapiens OX=9606 GN=ATP6V1A PE=1 SV=2	0,297198692	6,218693
Q96FZ2	Abasic site processing protein HMCES OS=Homo sapiens OX=9606 GN=HMCES PE=1 SV=1	-0,689862796	6,217497
Q14978	Nucleolar and coiled-body phosphoprotein 1 OS=Homo sapiens OX=9606 GN=NOLC1 PE=1 SV=2	-0,476300707	6,200439
Q14847	LIM and SH3 domain protein 1 OS=Homo sapiens OX=9606 GN=LASP1 PE=1 SV=2	0,38543669	6,196311
P26640	Valine--tRNA ligase OS=Homo sapiens OX=9606 GN=VARS PE=1 SV=4	-0,23270646	6,168296
Q9NUW8	Tyrosyl-DNA phosphodiesterase 1 OS=Homo sapiens OX=9606 GN=TDP1 PE=1 SV=2	-0,675001352	6,141814
Q8TAQ2	SWI/SNF complex subunit SMARCC2 OS=Homo sapiens OX=9606 GN=SMARCC2 PE=1 SV=1	-0,32918341	6,114339
Q9UDT6	CAP-Gly domain-containing linker protein 2 OS=Homo sapiens OX=9606 GN=CLIP2 PE=1 SV=1	0,567726633	6,110606
Q02790	Peptidyl-prolyl cis-trans isomerase FKBP4 OS=Homo sapiens OX=9606 GN=FKBP4 PE=1 SV=3	0,305385447	6,083424
P53794	Sodium/myo-inositol cotransporter OS=Homo sapiens OX=9606 GN=SLC5A3 PE=1 SV=2	1,091249868	6,079101
Q9BZ68	Putative FERM domain-containing protein FRMD8P1 OS=Homo sapiens OX=9606 GN=FRMD8P1 PE=5 SV=2	-1,477291437	6,044246
P07951	Tropomyosin beta chain OS=Homo sapiens OX=9606 GN=TPM2 PE=1 SV=1	-1,078945046	6,024314
Q8IY17	Neuropathy target esterase OS=Homo sapiens OX=9606 GN=PNPLA6 PE=1 SV=3	0,491256511	6,017629
P86791	Vacuolar fusion protein CCZ1 homolog OS=Homo sapiens OX=9606 GN=CCZ1 PE=1 SV=1	-1,480770545	6,007626
Q92922	SWI/SNF complex subunit SMARCC1 OS=Homo sapiens OX=9606 GN=SMARCC1 PE=1 SV=3	-0,558214582	5,992198
P62191	26S proteasome regulatory subunit 4 OS=Homo sapiens OX=9606 GN=PSMC1 PE=1 SV=1	-0,301803794	5,982247
P42167	Lamina-associated polypeptide 2, isoforms beta/gamma OS=Homo sapiens OX=9606 GN=TMPO PE=1 SV=2	-0,388289023	5,966849
P28300	Protein-lysine 6-oxidase OS=Homo sapiens OX=9606 GN=LOX PE=1 SV=2	0,661984814	5,952077
P23229	Integrin alpha-6 OS=Homo sapiens OX=9606 GN=ITGA6 PE=1 SV=5	-0,350316498	5,939486
Q9H9Y6	DNA-directed RNA polymerase I subunit RPA2 OS=Homo sapiens OX=9606 GN=POLR1B PE=1 SV=2	0,86161417	5,93218
P25685	DnaJ homolog subfamily B member 1 OS=Homo sapiens OX=9606 GN=DNAJB1 PE=1 SV=4	0,417109077	5,906861
P78357	Contactin-associated protein 1 OS=Homo sapiens OX=9606 GN=CNTNAP1 PE=1 SV=1	-0,514020436	5,901945
Q92616	eIF-2-alpha kinase activator GCN1 OS=Homo sapiens OX=9606 GN=GCN1 PE=1 SV=6	0,151670021	5,886434
Q8IWR1	Tripartite motif-containing protein 59 OS=Homo sapiens OX=9606 GN=TRIM59 PE=1 SV=1	-0,672833626	5,875547
Q99707	Methionine synthase OS=Homo sapiens OX=9606 GN=MTR PE=1 SV=2	-0,433695679	5,862973
Q8NB90	ATPase family protein 2 homolog OS=Homo sapiens OX=9606 GN=SPATA5 PE=1 SV=3	-0,508465911	5,825294
P11279	Lysosome-associated membrane glycoprotein 1 OS=Homo sapiens OX=9606 GN=LAMP1 PE=1 SV=3	-0,727821879	5,820399
Q8NBT2	Kinetochore protein Spc24 OS=Homo sapiens OX=9606 GN=SPC24 PE=1 SV=2	-0,725272087	5,799827
Q14444	Caprin-1 OS=Homo sapiens OX=9606 GN=CAPRIN1 PE=1 SV=2	-0,366058568	5,780468
Q9Y230	RuvB-like 2 OS=Homo sapiens OX=9606 GN=RUVBL2 PE=1 SV=3	-0,36566528	5,772082
Q15642	Cdc42-interacting protein 4 OS=Homo sapiens OX=9606 GN=TRIP10 PE=1 SV=3	0,427819513	5,761171
Q8WVY7	Ubiquitin-like domain-containing CTD phosphatase 1 OS=Homo sapiens OX=9606 GN=UBLCP1 PE=1 SV=2	-0,538365779	5,744315
P56945	Breast cancer anti-estrogen resistance protein 1 OS=Homo sapiens OX=9606 GN=BCAR1 PE=1 SV=2	-0,407950762	5,738392
P12236	ADP/ATP translocase 3 OS=Homo sapiens OX=9606 GN=SLC25A6 PE=1 SV=4	-0,31399347	5,722995
P17813	Endoglin OS=Homo sapiens OX=9606 GN=ENG PE=1 SV=2	0,445091799	5,694073
Q5JVS0	Intracellular hyaluronan-binding protein 4 OS=Homo sapiens OX=9606 GN=HABP4 PE=1 SV=1	-0,821341913	5,674844
Q9HBL8	NmrA-like family domain-containing protein 1 OS=Homo sapiens OX=9606 GN=NMRAL1 PE=1 SV=1	-1,002358059	5,673539
Q92597	Protein NDRG1 OS=Homo sapiens OX=9606 GN=NDRG1 PE=1 SV=1	0,707382197	5,654708
P46821	Microtubule-associated protein 1B OS=Homo sapiens OX=9606 GN=MAP1B PE=1 SV=2	0,146069597	5,603639
Q04637	Eukaryotic translation initiation factor 4 gamma 1 OS=Homo sapiens OX=9606 GN=EIF4G1 PE=1 SV=4	-0,210032015	5,589431
Q9H2U1	ATP-dependent DNA/RNA helicase DHX36 OS=Homo sapiens OX=9606 GN=DHX36 PE=1 SV=2	-0,437068434	5,564287
P50748	Kinetochore-associated protein 1 OS=Homo sapiens OX=9606 GN=KNTC1 PE=1 SV=1	-0,416354729	5,563045
Q99959	Plakophilin-2 OS=Homo sapiens OX=9606 GN=PKP2 PE=1 SV=2	0,489354203	5,561337
P46778	60S ribosomal protein L21 OS=Homo sapiens OX=9606 GN=RPL21 PE=1 SV=2	-0,566367453	5,5597
O00622	CCN family member 1 OS=Homo sapiens OX=9606 GN=CCN1 PE=1 SV=1	1,311211042	5,532306
Q969M7	NEDD8-conjugating enzyme UBE2F OS=Homo sapiens OX=9606 GN=UBE2F PE=1 SV=1	-1,519009522	5,521599
P55060	Exportin-2 OS=Homo sapiens OX=9606 GN=CSE1L PE=1 SV=3	-0,191120719	5,516734
P06454	Prothymosin alpha OS=Homo sapiens OX=9606 GN=PTMA PE=1 SV=2	-0,687817523	5,494465
Q7L014	Probable ATP-dependent RNA helicase DDX46 OS=Homo sapiens OX=9606 GN=DDX46 PE=1 SV=2	-0,266493454	5,470879
P18031	Tyrosine-protein phosphatase non-receptor type 1 OS=Homo sapiens OX=9606 GN=PTPN1 PE=1 SV=1	0,376859931	5,453479
P30046	D-dopachrome decarboxylase OS=Homo sapiens OX=9606 GN=DDT PE=1 SV=3	-0,786808931	5,449156
O95365	Zinc finger and BTB domain-containing protein 7A OS=Homo sapiens OX=9606 GN=ZBTB7A PE=1 SV=1	-1,716323563	5,446129
Q7Z5L9	Interferon regulatory factor 2-binding protein 2 OS=Homo sapiens OX=9606 GN=IRF2BP2 PE=1 SV=2	-0,681144918	5,439452
P04181	Ornithine aminotransferase, mitochondrial OS=Homo sapiens OX=9606 GN=OAT PE=1 SV=1	0,479997159	5,432043
O14776	Transcription elongation regulator 1 OS=Homo sapiens OX=9606 GN=TCERG1 PE=1 SV=2	0,28765388	5,412681
O15355	Protein phosphatase 1G OS=Homo sapiens OX=9606 GN=PPM1G PE=1 SV=1	-0,388011807	5,373811
O75208	Ubiquinone biosynthesis protein COQ9, mitochondrial OS=Homo sapiens OX=9606 GN=COQ9 PE=1 SV=1	-0,762044136	5,356462
Q9NVH6	Trimethyllysine dioxygenase, mitochondrial OS=Homo sapiens OX=9606 GN=TMLHE PE=1 SV=1	0,124505619	5,348471

O15013	Rho guanine nucleotide exchange factor 10 OS=Homo sapiens OX=9606 GN=ARHGEF10 PE=1 SV=4	0,38593372	5,336009
Q13435	Splicing factor 3B subunit 2 OS=Homo sapiens OX=9606 GN=SF3B2 PE=1 SV=2	-0,260989017	5,309081
O00233	26S proteasome non-ATPase regulatory subunit 9 OS=Homo sapiens OX=9606 GN=PSMD9 PE=1 SV=3	-0,595116393	5,302369
P61587	Rho-related GTP-binding protein RhoE OS=Homo sapiens OX=9606 GN=RND3 PE=1 SV=1	1,227113298	5,278166
O15230	Laminin subunit alpha-5 OS=Homo sapiens OX=9606 GN=LAMA5 PE=1 SV=8	0,660984538	5,27214
P51648	Aldehyde dehydrogenase family 3 member A2 OS=Homo sapiens OX=9606 GN=ALDH3A2 PE=1 SV=1	0,418532551	5,265371
Q27J81	Inverted formin-2 OS=Homo sapiens OX=9606 GN=INF2 PE=1 SV=2	-0,214531418	5,26145
O95905	Protein ecdysoneless homolog OS=Homo sapiens OX=9606 GN=ECD PE=1 SV=1	-0,658821379	5,254092
Q14690	Protein RRP5 homolog OS=Homo sapiens OX=9606 GN=PDCD11 PE=1 SV=3	-0,39817058	5,250394
Q8WXI7	Mucin-16 OS=Homo sapiens OX=9606 GN=MUC16 PE=1 SV=3	-1,432335195	5,244283
Q9HD33	39S ribosomal protein L47, mitochondrial OS=Homo sapiens OX=9606 GN=MRPL47 PE=1 SV=2	-0,657497648	5,243038
Q9NVI1	Fanconi anemia group I protein OS=Homo sapiens OX=9606 GN=FANCI PE=1 SV=4	-0,340171697	5,233762
O95864	Acyl-CoA 6-desaturase OS=Homo sapiens OX=9606 GN=FADS2 PE=1 SV=1	-0,536602813	5,225358
Q6PGP7	Tetratricopeptide repeat protein 37 OS=Homo sapiens OX=9606 GN=TTC37 PE=1 SV=1	-0,396567645	5,222941
P62820	Ras-related protein Rab-1A OS=Homo sapiens OX=9606 GN=RAB1A PE=1 SV=3	0,39625911	5,217659
Q96QD8	Sodium-coupled neutral amino acid transporter 2 OS=Homo sapiens OX=9606 GN=SLC38A2 PE=1 SV=2	2,04726514	5,212591
P12277	Creatine kinase B-type OS=Homo sapiens OX=9606 GN=CKB PE=1 SV=1	-0,584725335	5,199907
Q9Y520	Protein PRRC2C OS=Homo sapiens OX=9606 GN=PRRC2C PE=1 SV=4	-0,251978962	5,186192
Q5SRE5	Nucleoporin NUP188 homolog OS=Homo sapiens OX=9606 GN=NUP188 PE=1 SV=1	-0,308220848	5,17948
P25440	Bromodomain-containing protein 2 OS=Homo sapiens OX=9606 GN=BRD2 PE=1 SV=2	1,205993753	5,166198
O60610	Protein diaphanous homolog 1 OS=Homo sapiens OX=9606 GN=DIAPH1 PE=1 SV=2	-0,227079123	5,157093
Q9NQ29	Putative RNA-binding protein Luc7-like 1 OS=Homo sapiens OX=9606 GN=LUC7L PE=1 SV=1	0,458947815	5,14127
Q7Z3K3	Pogo transposable element with ZNF domain OS=Homo sapiens OX=9606 GN=POGZ PE=1 SV=2	-0,432861734	5,140879
Q12873	Chromodomain-helicase-DNA-binding protein 3 OS=Homo sapiens OX=9606 GN=CHD3 PE=1 SV=3	-0,290724099	5,13701
P30153	Serine/threonine-protein phosphatase 2A 65 kDa regulatory subunit A alpha isoform OS=Homo sapiens OX=9606 GN=PPF	-0,277227956	5,136861
Q9HCC0	Methylcrotonoyl-CoA carboxylase beta chain, mitochondrial OS=Homo sapiens OX=9606 GN=MCCC2 PE=1 SV=1	-0,432341073	5,133092
Q09666	Neuroblast differentiation-associated protein AHNAK OS=Homo sapiens OX=9606 GN=AHNAK PE=1 SV=2	-0,074972772	5,126681
P20962	Parathymosin OS=Homo sapiens OX=9606 GN=PTMS PE=1 SV=2	-1,176525417	5,118787
Q9H6S3	Epidermal growth factor receptor kinase substrate 8-like protein 2 OS=Homo sapiens OX=9606 GN=EPS8L2 PE=1 SV=2	-0,270050764	5,107236
P08195	4F2 cell-surface antigen heavy chain OS=Homo sapiens OX=9606 GN=SLC3A2 PE=1 SV=3	0,288942782	5,092879
O95453	Poly(A)-specific ribonuclease PARN OS=Homo sapiens OX=9606 GN=PARN PE=1 SV=1	-0,639001701	5,087875
O94973	AP-2 complex subunit alpha-2 OS=Homo sapiens OX=9606 GN=AP2A2 PE=1 SV=2	-0,296203324	5,08778
Q9HBM1	Kinetochore protein Spc25 OS=Homo sapiens OX=9606 GN=SPC25 PE=1 SV=1	-0,573048891	5,084342
Q9BT25	HAUS augmin-like complex subunit 8 OS=Homo sapiens OX=9606 GN=HAUS8 PE=1 SV=3	-0,880562644	5,07901
P63244	Receptor of activated protein C kinase 1 OS=Homo sapiens OX=9606 GN=RACK1 PE=1 SV=3	-0,312607445	5,07766
P55290	Cadherin-13 OS=Homo sapiens OX=9606 GN=CDH13 PE=1 SV=1	0,522804858	5,069621
Q13155	Aminoacyl tRNA synthase complex-interacting multifunctional protein 2 OS=Homo sapiens OX=9606 GN=AIMP2 PE=1 SV=	-0,483954744	5,060895
Q9Y4K0	Lysyl oxidase homolog 2 OS=Homo sapiens OX=9606 GN=LOXL2 PE=1 SV=1	0,343372186	5,059587
Q86UX7	Fermitin family homolog 3 OS=Homo sapiens OX=9606 GN=FERMT3 PE=1 SV=1	-0,521374896	5,053458
P14635	G2/mitotic-specific cyclin-B1 OS=Homo sapiens OX=9606 GN=CCNB1 PE=1 SV=1	0,569221056	5,046362
O00468	Agrin OS=Homo sapiens OX=9606 GN=AGRN PE=1 SV=6	0,56752152	5,029485
O00154	Cytosolic acyl coenzyme A thioester hydrolase OS=Homo sapiens OX=9606 GN=ACOT7 PE=1 SV=3	0,450792563	5,028717
Q6NYC8	Phostensin OS=Homo sapiens OX=9606 GN=PPP1R18 PE=1 SV=1	-0,384935672	5,024309
P16520	Guanine nucleotide-binding protein G(I)/G(S)/G(T) subunit beta-3 OS=Homo sapiens OX=9606 GN=GNB3 PE=1 SV=1	-0,385969313	5,003753
P06737	Glycogen phosphorylase, liver form OS=Homo sapiens OX=9606 GN=PYGL PE=1 SV=4	0,300450821	4,999036
P50454	Serpin H1 OS=Homo sapiens OX=9606 GN=SERPINH1 PE=1 SV=2	0,27828048	4,998192
Q13724	Mannosyl-oligosaccharide glucosidase OS=Homo sapiens OX=9606 GN=MOGS PE=1 SV=5	0,265590957	4,987722
P31350	Ribonucleoside-diphosphate reductase subunit M2 OS=Homo sapiens OX=9606 GN=RRM2 PE=1 SV=1	-0,421655403	4,97348
Q01581	Hydroxymethylglutaryl-CoA synthase, cytoplasmic OS=Homo sapiens OX=9606 GN=HMGCS1 PE=1 SV=2	-0,399004641	4,952243
Q01130	Serine/arginine-rich splicing factor 2 OS=Homo sapiens OX=9606 GN=SRSF2 PE=1 SV=4	-0,710740752	4,935646
Q9Y6M1	Insulin-like growth factor 2 mRNA-binding protein 2 OS=Homo sapiens OX=9606 GN=IGF2BP2 PE=1 SV=2	0,419111434	4,935542
P00533	Epidermal growth factor receptor OS=Homo sapiens OX=9606 GN=EGFR PE=1 SV=2	-0,289772068	4,934144
Q9H7D0	Dedicator of cytokinesis protein 5 OS=Homo sapiens OX=9606 GN=DOCK5 PE=1 SV=3	-0,363475587	4,930204
Q3V6T2	Girdin OS=Homo sapiens OX=9606 GN=CCDC88A PE=1 SV=2	-0,705911078	4,90229
Q8IWB7	WD repeat and FYVE domain-containing protein 1 OS=Homo sapiens OX=9606 GN=WDFY1 PE=1 SV=1	0,507518575	4,896644
Q9BQG0	Myb-binding protein 1A OS=Homo sapiens OX=9606 GN=MYBBP1A PE=1 SV=2	-0,218822676	4,885599
Q16850	Lanosterol 14-alpha demethylase OS=Homo sapiens OX=9606 GN=CYP51A1 PE=1 SV=3	-0,842260004	4,882202
P09486	SPARC OS=Homo sapiens OX=9606 GN=SPARC PE=1 SV=1	0,505350931	4,872083
Q9NZN4	EH domain-containing protein 2 OS=Homo sapiens OX=9606 GN=EHD2 PE=1 SV=2	-0,287072304	4,870044
Q14566	DNA replication licensing factor MCM6 OS=Homo sapiens OX=9606 GN=MCM6 PE=1 SV=1	-0,224631238	4,851272
O75475	PC4 and SFRS1-interacting protein OS=Homo sapiens OX=9606 GN=PSIP1 PE=1 SV=1	-0,392632082	4,850545
P49023	Paxillin OS=Homo sapiens OX=9606 GN=PXN PE=1 SV=3	0,286193086	4,84922
Q14558	Phosphoribosyl pyrophosphate synthase-associated protein 1 OS=Homo sapiens OX=9606 GN=PRPSAP1 PE=1 SV=2	-0,467017212	4,847605
Q8N0X7	Spartin OS=Homo sapiens OX=9606 GN=SPART PE=1 SV=1	0,344951411	4,846367
P42330	Aldo-keto reductase family 1 member C3 OS=Homo sapiens OX=9606 GN=AKR1C3 PE=1 SV=4	0,546980457	4,824829
Q9NP72	Ras-related protein Rab-18 OS=Homo sapiens OX=9606 GN=RAB18 PE=1 SV=1	0,546933209	4,824356
P51114	Fragile X mental retardation syndrome-related protein 1 OS=Homo sapiens OX=9606 GN=FXR1 PE=1 SV=3	0,248478971	4,811335
Q8TCS8	Polyribonucleotide nucleotidyltransferase 1, mitochondrial OS=Homo sapiens OX=9606 GN=PNPT1 PE=1 SV=2	-0,341838425	4,78788
Q9NV70	Exocyst complex component 1 OS=Homo sapiens OX=9606 GN=EXOC1 PE=1 SV=4	-0,369000885	4,754056
O60566	Mitotic checkpoint serine/threonine-protein kinase BUB1 beta OS=Homo sapiens OX=9606 GN=BUB1B PE=1 SV=3	0,494479359	4,748803
P61160	Actin-related protein 2 OS=Homo sapiens OX=9606 GN=ACTR2 PE=1 SV=1	-0,352886117	4,740793
Q63HR2	#N/D	-1,037592145	4,732433
Q86UU1	Pleckstrin homology-like domain family B member 1 OS=Homo sapiens OX=9606 GN=PHLDB1 PE=1 SV=1	0,296141802	4,712006
P84098	60S ribosomal protein L19 OS=Homo sapiens OX=9606 GN=RPL19 PE=1 SV=1	-0,535664248	4,711591
O75828	Carbonyl reductase [NADPH] 3 OS=Homo sapiens OX=9606 GN=CBR3 PE=1 SV=3	-0,403551303	4,70409
Q15554	Telomeric repeat-binding factor 2 OS=Homo sapiens OX=9606 GN=TERF2 PE=1 SV=3	-1,043346342	4,694031
P23634	Plasma membrane calcium-transporting ATPase 4 OS=Homo sapiens OX=9606 GN=ATP2B4 PE=1 SV=2	0,295234615	4,692071
Q9UN86	Ras GTPase-activating protein-binding protein 2 OS=Homo sapiens OX=9606 GN=G3BP2 PE=1 SV=2	0,454468329	4,689609
Q96T60	Bifunctional polynucleotide phosphatase/kinase OS=Homo sapiens OX=9606 GN=PNKP PE=1 SV=1	-0,589834571	4,66926
P23921	Ribonucleoside-diphosphate reductase large subunit OS=Homo sapiens OX=9606 GN=RRM1 PE=1 SV=1	-0,225861431	4,661472
P06703	Protein S100-A6 OS=Homo sapiens OX=9606 GN=S100A6 PE=1 SV=1	-0,36289126	4,651074
Q13200	26S proteasome non-ATPase regulatory subunit 2 OS=Homo sapiens OX=9606 GN=PSMD2 PE=1 SV=3	-0,171919661	4,640752
Q93008	Probable ubiquitin carboxyl-terminal hydrolase FAF-X OS=Homo sapiens OX=9606 GN=USP9X PE=1 SV=3	0,159341526	4,625079
Q8N766	ER membrane protein complex subunit 1 OS=Homo sapiens OX=9606 GN=EMC1 PE=1 SV=1	-0,36116698	4,622078
P00403	Cytochrome c oxidase subunit 2 OS=Homo sapiens OX=9606 GN=MT-CO2 PE=1 SV=1	-0,792828449	4,62099
Q16531	DNA damage-binding protein 1 OS=Homo sapiens OX=9606 GN=DDB1 PE=1 SV=1	-0,197996232	4,586259
Q9UQ35	Serine/arginine repetitive matrix protein 2 OS=Homo sapiens OX=9606 GN=SRRM2 PE=1 SV=2	0,235640788	4,584979
P43686	26S proteasome regulatory subunit 6B OS=Homo sapiens OX=9606 GN=PSMC4 PE=1 SV=2	-0,255613539	4,579262
Q8NBF6	Late secretory pathway protein AVL9 homolog OS=Homo sapiens OX=9606 GN=AVL9 PE=1 SV=1	-0,784280687	4,574987
O76021	Ribosomal L1 domain-containing protein 1 OS=Homo sapiens OX=9606 GN=RSL1D1 PE=1 SV=3	-0,318286751	4,56647
P61254	60S ribosomal protein L26 OS=Homo sapiens OX=9606 GN=RPL26 PE=1 SV=1	-0,416623277	4,558535

P10809	60 kDa heat shock protein, mitochondrial OS=Homo sapiens OX=9606 GN=HSPD1 PE=1 SV=2	0,197027433	4,553496
O00264	Membrane-associated progesterone receptor component 1 OS=Homo sapiens OX=9606 GN=PGRMC1 PE=1 SV=3	0,655501262	4,548914
O75190	DnaJ homolog subfamily B member 6 OS=Homo sapiens OX=9606 GN=DNAJB6 PE=1 SV=2	0,519021959	4,544499
Q9BTY7	Protein HGH1 homolog OS=Homo sapiens OX=9606 GN=HGH1 PE=1 SV=1	-0,778424378	4,543325
Q8TCE6	Protein FAM45A OS=Homo sapiens OX=9606 GN=FAM45A PE=2 SV=1	-0,476157721	4,540752
Q9NYF8	Bcl-2-associated transcription factor 1 OS=Homo sapiens OX=9606 GN=BCLAF1 PE=1 SV=2	-0,316851734	4,53861
Q9Y399	28S ribosomal protein S2, mitochondrial OS=Homo sapiens OX=9606 GN=MRPS2 PE=1 SV=1	-0,573734656	4,530386
P40855	Peroxisomal biogenesis factor 19 OS=Homo sapiens OX=9606 GN=PEX19 PE=1 SV=1	-0,355683226	4,530073
Q06210	Glutamine--fructose-6-phosphate aminotransferase [isomerizing] 1 OS=Homo sapiens OX=9606 GN=GFPT1 PE=1 SV=3	0,237507266	4,510753
Q13362	Serine/threonine-protein phosphatase 2A 56 kDa regulatory subunit gamma isoform OS=Homo sapiens OX=9606 GN=PPF	-0,57059318	4,503193
O00203	AP-3 complex subunit beta-1 OS=Homo sapiens OX=9606 GN=AP3B1 PE=1 SV=3	-0,224351427	4,50295
Q9HBG6	Intraflagellar transport protein 122 homolog OS=Homo sapiens OX=9606 GN=IFT122 PE=1 SV=2	-0,460904433	4,496014
P14866	Heterogeneous nuclear ribonucleoprotein L OS=Homo sapiens OX=9606 GN=HNRNPL PE=1 SV=2	-0,264355147	4,495274
P41240	Tyrosine-protein kinase CSK OS=Homo sapiens OX=9606 GN=CSK PE=1 SV=1	-0,36913477	4,478067
Q3YEC7	Rab-like protein 6 OS=Homo sapiens OX=9606 GN=RABL6 PE=1 SV=2	-0,47044284	4,475809
Q96KB5	Lymphokine-activated killer T-cell-originated protein kinase OS=Homo sapiens OX=9606 GN=PBK PE=1 SV=3	-0,410585019	4,475788
P46783	40S ribosomal protein S10 OS=Homo sapiens OX=9606 GN=RPS10 PE=1 SV=1	-0,387981106	4,473699
Q13243	Serine/arginine-rich splicing factor 5 OS=Homo sapiens OX=9606 GN=SRSF5 PE=1 SV=1	-0,765111053	4,470917
Q9BUP3	Oxidoreductase HTATIP2 OS=Homo sapiens OX=9606 GN=HTATIP2 PE=1 SV=2	0,983468289	4,467731
Q68E01	Integrator complex subunit 3 OS=Homo sapiens OX=9606 GN=INTS3 PE=1 SV=1	-0,43650907	4,463725
Q04917	14-3-3 protein eta OS=Homo sapiens OX=9606 GN=YWHAH PE=1 SV=4	-0,367794137	4,456948
O00116	Alkyldihydroxyacetonephosphate synthase, peroxisomal OS=Homo sapiens OX=9606 GN=AGPS PE=1 SV=1	0,302123622	4,452807
Q6DKI1	60S ribosomal protein L7-like 1 OS=Homo sapiens OX=9606 GN=RPL7L1 PE=1 SV=2	-0,641809521	4,451327
O43913	Origin recognition complex subunit 5 OS=Homo sapiens OX=9606 GN=ORC5 PE=1 SV=1	-0,980833317	4,450809
P55145	Mesencephalic astrocyte-derived neurotrophic factor OS=Homo sapiens OX=9606 GN=MANF PE=1 SV=3	0,760922613	4,448012
Q9Y4G6	Talin-2 OS=Homo sapiens OX=9606 GN=TLN2 PE=1 SV=4	-0,312159248	4,447791
P00750	Tissue-type plasminogen activator OS=Homo sapiens OX=9606 GN=PLAT PE=1 SV=1	0,508529725	4,438855
Q8IY33	MICAL-like protein 2 OS=Homo sapiens OX=9606 GN=MICALL2 PE=1 SV=1	-0,803753689	4,434377
P11388	DNA topoisomerase 2-alpha OS=Homo sapiens OX=9606 GN=TOP2A PE=1 SV=3	0,204591009	4,430424
Q15019	Septin-2 OS=Homo sapiens OX=9606 GN=SEPTIN2 PE=1 SV=1	-0,268051873	4,425384
P10909	Clusterin OS=Homo sapiens OX=9606 GN=CLU PE=1 SV=1	0,973174276	4,422861
Q5JPE7	Nodal modulator 2 OS=Homo sapiens OX=9606 GN=NOMO2 PE=1 SV=1	0,221208329	4,41259
Q6PJG6	BRCA1-associated ATM activator 1 OS=Homo sapiens OX=9606 GN=BRAT1 PE=1 SV=2	-0,274000524	4,398179
P46013	Proliferation marker protein Ki-67 OS=Homo sapiens OX=9606 GN=MKI67 PE=1 SV=2	-0,281146047	4,385464
Q08117	TLE family member 5 OS=Homo sapiens OX=9606 GN=TLE5 PE=1 SV=4	-0,748903333	4,381954
P04818	Thymidylate synthase OS=Homo sapiens OX=9606 GN=TYMS PE=1 SV=3	-0,403443199	4,378093
Q9GZP8	Immortalization up-regulated protein OS=Homo sapiens OX=9606 GN=IMUP PE=1 SV=1	-1,506531561	4,375642
Q9HA64	Ketosamine-3-kinase OS=Homo sapiens OX=9606 GN=FN3KRP PE=1 SV=2	-0,501886384	4,37186
O15347	High mobility group protein B3 OS=Homo sapiens OX=9606 GN=HMGB3 PE=1 SV=4	-0,554393494	4,362499
P49591	Serine--tRNA ligase, cytoplasmic OS=Homo sapiens OX=9606 GN=SARS PE=1 SV=3	0,27957549	4,351643
P02786	Transferrin receptor protein 1 OS=Homo sapiens OX=9606 GN=TFRC PE=1 SV=2	0,427446671	4,34992
P42858	Huntingtin OS=Homo sapiens OX=9606 GN=HTT PE=1 SV=2	-0,552778804	4,348434
Q8NFF5	FAD synthase OS=Homo sapiens OX=9606 GN=FLAD1 PE=1 SV=1	-0,36036451	4,3402
O15067	Phosphoribosylformylglycinamidine synthase OS=Homo sapiens OX=9606 GN=PFAS PE=1 SV=4	-0,235525092	4,331958
Q13131	5'-AMP-activated protein kinase catalytic subunit alpha-1 OS=Homo sapiens OX=9606 GN=PRKAA1 PE=1 SV=4	-0,496756586	4,320078
Q9NUU7	ATP-dependent RNA helicase DDX19A OS=Homo sapiens OX=9606 GN=DDX19A PE=1 SV=1	0,737520025	4,318938
Q15185	Prostaglandin E synthase 3 OS=Homo sapiens OX=9606 GN=PTGES3 PE=1 SV=1	0,496053262	4,312976
P21333	Filamin-A OS=Homo sapiens OX=9606 GN=FLNA PE=1 SV=4	-0,093600684	4,293507
Q9NPD8	Ubiquitin-conjugating enzyme E2 T OS=Homo sapiens OX=9606 GN=UBE2T PE=1 SV=1	-0,546272219	4,291684
O15066	Kinesin-like protein KIF3B OS=Homo sapiens OX=9606 GN=KIF3B PE=1 SV=1	0,493299602	4,28516
Q9Y5J9	Mitochondrial import inner membrane translocase subunit Tim8 B OS=Homo sapiens OX=9606 GN=TIMM8B PE=1 SV=1	-0,352556474	4,285153
Q9Y448	Small kinetochore-associated protein OS=Homo sapiens OX=9606 GN=KNSTRN PE=1 SV=2	-0,934002552	4,277633
Q14240	Eukaryotic initiation factor 4A-II OS=Homo sapiens OX=9606 GN=EIF4A2 PE=1 SV=2	0,617202282	4,274285
P30520	Adenylosuccinate synthetase isozyme 2 OS=Homo sapiens OX=9606 GN=ADSS PE=1 SV=3	-0,3255019	4,257788
Q9BRQ8	Apoptosis-inducing factor 2 OS=Homo sapiens OX=9606 GN=AIFM2 PE=1 SV=1	-0,72575754	4,253362
P0DPD5	#N/D	-0,488649397	4,252128
Q5T6F2	Ubiquitin-associated protein 2 OS=Homo sapiens OX=9606 GN=UBAP2 PE=1 SV=1	-0,613667771	4,248685
P30040	Endoplasmic reticulum resident protein 29 OS=Homo sapiens OX=9606 GN=ERP29 PE=1 SV=4	0,450453531	4,248609
P62857	40S ribosomal protein S28 OS=Homo sapiens OX=9606 GN=RPS28 PE=1 SV=1	-0,925446661	4,245395
P09651	Heterogeneous nuclear ribonucleoprotein A1 OS=Homo sapiens OX=9606 GN=HNRNPA1 PE=1 SV=5	-0,301554131	4,24419
Q14011	Cold-inducible RNA-binding protein OS=Homo sapiens OX=9606 GN=CIRBP PE=1 SV=1	-0,72390883	4,243013
Q53EZ4	Centrosomal protein of 55 kDa OS=Homo sapiens OX=9606 GN=CEP55 PE=1 SV=3	-0,338350081	4,241504
P01024	#N/D	0,72352328	4,240853
P29692	Elongation factor 1-delta OS=Homo sapiens OX=9606 GN=EEF1D PE=1 SV=5	-0,259902741	4,238784
Q86X55	Histone-arginine methyltransferase CARM1 OS=Homo sapiens OX=9606 GN=CARM1 PE=1 SV=3	-0,353564297	4,233794
Q9H078	Caseinolytic peptidase B protein homolog OS=Homo sapiens OX=9606 GN=CLPB PE=1 SV=1	0,392797879	4,23287
O94874	E3 UFM1-protein ligase 1 OS=Homo sapiens OX=9606 GN=UFL1 PE=1 SV=2	-0,259522556	4,230139
P20585	DNA mismatch repair protein Msh3 OS=Homo sapiens OX=9606 GN=MSH3 PE=1 SV=4	-0,721301023	4,228395
Q16512	Serine/threonine-protein kinase N1 OS=Homo sapiens OX=9606 GN=PKN1 PE=1 SV=2	-0,417212957	4,221607
Q4VC31	Coiled-coil domain-containing protein 58 OS=Homo sapiens OX=9606 GN=CCDC58 PE=1 SV=1	0,719282568	4,217064
Q9P227	#N/D	-1,051192694	4,206867
Q9C035	Tripartite motif-containing protein 5 OS=Homo sapiens OX=9606 GN=TRIM5 PE=1 SV=1	-1,974823825	4,205203
P13797	Plastin-3 OS=Homo sapiens OX=9606 GN=PLS3 PE=1 SV=4	0,289352771	4,197807
Q9NVS2	39S ribosomal protein S18a, mitochondrial OS=Homo sapiens OX=9606 GN=MRPS18A PE=1 SV=1	-2,58643406	4,196247
O60884	DnaJ homolog subfamily A member 2 OS=Homo sapiens OX=9606 GN=DNAJA2 PE=1 SV=1	0,321676943	4,191192
Q93009	Ubiquitin carboxyl-terminal hydrolase 7 OS=Homo sapiens OX=9606 GN=USP7 PE=1 SV=2	-0,196695336	4,18512
P09543	2',3'-cyclic-nucleotide 3'-phosphodiesterase OS=Homo sapiens OX=9606 GN=CNP PE=1 SV=2	-0,257231823	4,178161
Q9NY93	Probable ATP-dependent RNA helicase DDX56 OS=Homo sapiens OX=9606 GN=DDX56 PE=1 SV=1	-0,388498206	4,174364
Q9UBP0	Spastin OS=Homo sapiens OX=9606 GN=SPAST PE=1 SV=1	0,906489881	4,173293
Q9NWV4	CXXC motif containing zinc binding protein OS=Homo sapiens OX=9606 GN=CZIB PE=1 SV=1	-0,710521952	4,167727
Q13616	Cullin-1 OS=Homo sapiens OX=9606 GN=CUL1 PE=1 SV=2	-0,27875059	4,162181
P60983	Glia maturation factor beta OS=Homo sapiens OX=9606 GN=GMFB PE=1 SV=2	-0,600005954	4,149343
P17844	Probable ATP-dependent RNA helicase DDX5 OS=Homo sapiens OX=9606 GN=DDX5 PE=1 SV=1	-0,233105833	4,145183
Q9Y5B8	Nucleoside diphosphate kinase 7 OS=Homo sapiens OX=9606 GN=NME7 PE=1 SV=1	0,705964584	4,141959
Q96FJ0	AMSH-like protease OS=Homo sapiens OX=9606 GN=STAMBPL1 PE=1 SV=2	0,705926518	4,141744
Q6PCE3	Glucose 1,6-bisphosphate synthase OS=Homo sapiens OX=9606 GN=PGM2L1 PE=1 SV=3	-0,347647337	4,14158
O95167	NADH dehydrogenase [ubiquinone] 1 alpha subcomplex subunit 3 OS=Homo sapiens OX=9606 GN=NDUFA3 PE=1 SV=1	-0,466274578	4,141378
O60502	Protein O-GlcNAcase OS=Homo sapiens OX=9606 GN=OGA PE=1 SV=2	-0,232439196	4,128437
Q13177	Serine/threonine-protein kinase PAK 2 OS=Homo sapiens OX=9606 GN=PAK2 PE=1 SV=3	-0,261495665	4,121948
P51398	28S ribosomal protein S29, mitochondrial OS=Homo sapiens OX=9606 GN=DAP3 PE=1 SV=1	-0,439125312	4,119904
Q2M389	WASH complex subunit 4 OS=Homo sapiens OX=9606 GN=WASHC4 PE=1 SV=2	0,276473432	4,115539

Q9UNH7	Sorting nexin-6 OS=Homo sapiens OX=9606 GN=SNX6 PE=1 SV=1	-0,317274032	4,11482
Q15029	116 kDa U5 small nuclear ribonucleoprotein component OS=Homo sapiens OX=9606 GN=EFTUD2 PE=1 SV=1	-0,191440751	4,110654
Q9UMS0	NFU1 iron-sulfur cluster scaffold homolog, mitochondrial OS=Homo sapiens OX=9606 GN=NFU1 PE=1 SV=2	-0,698594024	4,100137
Q96KG9	N-terminal kinase-like protein OS=Homo sapiens OX=9606 GN=SCYL1 PE=1 SV=1	0,30444121	4,097637
Q6EMK4	Vasorin OS=Homo sapiens OX=9606 GN=VASN PE=1 SV=1	0,696862429	4,090285
Q8IZ07	Ankyrin repeat domain-containing protein 13A OS=Homo sapiens OX=9606 GN=ANKRD13A PE=1 SV=3	-0,344181505	4,087735
Q8NC51	Plasminogen activator inhibitor 1 RNA-binding protein OS=Homo sapiens OX=9606 GN=SERBP1 PE=1 SV=2	-0,235707282	4,084117
P30101	Protein disulfide-isomerase A3 OS=Homo sapiens OX=9606 GN=PDIA3 PE=1 SV=4	0,2058249	4,078582
Q03154	Aminoacylase-1 OS=Homo sapiens OX=9606 GN=ACY1 PE=1 SV=1	-0,405615316	4,076519
Q8IXI2	Mitochondrial Rho GTPase 1 OS=Homo sapiens OX=9606 GN=RHOT1 PE=1 SV=2	0,880474736	4,072812
P35237	Serpin B6 OS=Homo sapiens OX=9606 GN=SERPINB6 PE=1 SV=3	0,282824536	4,069004
Q9NTX5	Ethylmalonyl-CoA decarboxylase OS=Homo sapiens OX=9606 GN=ECHDC1 PE=1 SV=2	0,471504668	4,064673
P55809	Succinyl-CoA:3-ketoacid coenzyme A transferase 1, mitochondrial OS=Homo sapiens OX=9606 GN=OXCT1 PE=1 SV=1	-0,434179932	4,063748
O94905	Erlin-2 OS=Homo sapiens OX=9606 GN=ERLIN2 PE=1 SV=1	-0,291992282	4,062683
P02511	Alpha-crystallin B chain OS=Homo sapiens OX=9606 GN=CRYAB PE=1 SV=2	1,334848021	4,054287
P01889	HLA class I histocompatibility antigen, B-7 alpha chain OS=Homo sapiens OX=9606 GN=HLA-B PE=1 SV=3	-0,358776465	4,045753
P33992	DNA replication licensing factor MCM5 OS=Homo sapiens OX=9606 GN=MCM5 PE=1 SV=5	-0,204492597	4,040833
Q9Y5J5	Pleckstrin homology-like domain family A member 3 OS=Homo sapiens OX=9606 GN=PHLDA3 PE=1 SV=1	0,418871418	4,036328
Q9HD15	Steroid receptor RNA activator 1 OS=Homo sapiens OX=9606 GN=SRA1 PE=1 SV=1	-0,686549862	4,031404
O60749	Sorting nexin-2 OS=Homo sapiens OX=9606 GN=SNX2 PE=1 SV=2	-0,264480681	4,030458
P18669	Phosphoglycerate mutase 1 OS=Homo sapiens OX=9606 GN=PGAM1 PE=1 SV=2	0,300444231	4,02536
Q9BVJ7	Dual specificity protein phosphatase 23 OS=Homo sapiens OX=9606 GN=DUSP23 PE=1 SV=1	-0,515627856	4,022951
Q2NL82	Pre-rRNA-processing protein TSR1 homolog OS=Homo sapiens OX=9606 GN=TSR1 PE=1 SV=1	-0,289862292	4,022529
O15031	Plexin-B2 OS=Homo sapiens OX=9606 GN=PLXNB2 PE=1 SV=3	0,311895894	4,021963
Q9BV44	THUMP domain-containing protein 3 OS=Homo sapiens OX=9606 GN=THUMPD3 PE=1 SV=1	-0,514615682	4,014038
Q9H2P0	Activity-dependent neuroprotector homeobox protein OS=Homo sapiens OX=9606 GN=ADNP PE=1 SV=1	-0,339338876	4,012716
P02545	Prelamin-A/C OS=Homo sapiens OX=9606 GN=LMNA PE=1 SV=1	-0,157271193	4,010732
Q9Y2R4	Probable ATP-dependent RNA helicase DDX52 OS=Homo sapiens OX=9606 GN=DDX52 PE=1 SV=3	0,864630302	4,010732
Q15738	Sterol-4-alpha-carboxylate 3-dehydrogenase, decarboxylating OS=Homo sapiens OX=9606 GN=NSDHL PE=1 SV=2	-0,299161433	4,002231
Q6YHU6	Thyroid adenoma-associated protein OS=Homo sapiens OX=9606 GN=THADA PE=1 SV=1	-0,513203005	4,001594
P11413	Glucose-6-phosphate 1-dehydrogenase OS=Homo sapiens OX=9606 GN=G6PD PE=1 SV=4	0,196481492	3,998316
Q9C0H2	Protein tweety homolog 3 OS=Homo sapiens OX=9606 GN=TTYH3 PE=1 SV=3	-0,679742087	3,992339
Q14195	Dihydropyrimidinase-related protein 3 OS=Homo sapiens OX=9606 GN=DPYSL3 PE=1 SV=1	-0,231885988	3,991218
O14786	Neuropilin-1 OS=Homo sapiens OX=9606 GN=NRP1 PE=1 SV=3	-0,427627911	3,98939
Q13442	28 kDa heat- and acid-stable phosphoprotein OS=Homo sapiens OX=9606 GN=PDAP1 PE=1 SV=1	-0,574661088	3,96346
Q9Y5Q8	General transcription factor 3C polypeptide 5 OS=Homo sapiens OX=9606 GN=GTF3C5 PE=1 SV=2	-0,352940877	3,961026
Q9NVP2	Histone chaperone ASF1B OS=Homo sapiens OX=9606 GN=ASF1B PE=1 SV=1	-0,850517764	3,954868
Q96A49	Synapse-associated protein 1 OS=Homo sapiens OX=9606 GN=SYAP1 PE=1 SV=1	-0,371775646	3,947757
O43175	D-3-phosphoglycerate dehydrogenase OS=Homo sapiens OX=9606 GN=PHGDH PE=1 SV=4	0,285837785	3,946946
Q9BWU0	Kanadaptin OS=Homo sapiens OX=9606 GN=SLC4A1AP PE=1 SV=1	0,371459485	3,943488
Q9Y3E7	Charged multivesicular body protein 3 OS=Homo sapiens OX=9606 GN=CHMP3 PE=1 SV=3	-0,570818882	3,935107
O95229	ZW10 interactor OS=Homo sapiens OX=9606 GN=ZWINT PE=1 SV=2	-0,845244647	3,933855
P06400	Retinoblastoma-associated protein OS=Homo sapiens OX=9606 GN=RB1 PE=1 SV=2	-0,319170434	3,926643
P18887	DNA repair protein XRCC1 OS=Homo sapiens OX=9606 GN=XRCC1 PE=1 SV=2	-0,504504229	3,924878
P08174	Complement decay-accelerating factor OS=Homo sapiens OX=9606 GN=CD55 PE=1 SV=4	0,504490325	3,924756
Q8N543	Prolyl 3-hydroxylase OGFOD1 OS=Homo sapiens OX=9606 GN=OGFOD1 PE=1 SV=1	-0,667724157	3,923
P16401	Histone H1.5 OS=Homo sapiens OX=9606 GN=HIST1H1B PE=1 SV=3	-0,369731239	3,920165
Q6NYC1	Bifunctional arginine demethylase and lysyl-hydroxylase JMJD6 OS=Homo sapiens OX=9606 GN=JMJD6 PE=1 SV=1	0,457152395	3,919257
Q9UJ70	N-acetyl-D-glucosamine kinase OS=Homo sapiens OX=9606 GN=NAGK PE=1 SV=4	-0,275003784	3,91615
Q13868	Exosome complex component RRP4 OS=Homo sapiens OX=9606 GN=EXOSC2 PE=1 SV=2	-0,665266662	3,908763
Q15393	Splicing factor 3B subunit 3 OS=Homo sapiens OX=9606 GN=SF3B3 PE=1 SV=4	-0,17969189	3,906259
Q9UKF6	Cleavage and polyadenylation specificity factor subunit 3 OS=Homo sapiens OX=9606 GN=CPSF3 PE=1 SV=1	-0,391834187	3,904676
Q8TCG1	Protein CIP2A OS=Homo sapiens OX=9606 GN=CIP2A PE=1 SV=2	-0,274235454	3,90122
P49736	DNA replication licensing factor MCM2 OS=Homo sapiens OX=9606 GN=MCM2 PE=1 SV=4	-0,199329861	3,895776
P42704	Leucine-rich PPR motif-containing protein, mitochondrial OS=Homo sapiens OX=9606 GN=LRPPRC PE=1 SV=3	-0,136989977	3,883386
Q8NEM7	Transcription factor SPT20 homolog OS=Homo sapiens OX=9606 GN=SUPT20H PE=1 SV=2	0,619320513	3,879276
Q14004	Cyclin-dependent kinase 13 OS=Homo sapiens OX=9606 GN=CDK13 PE=1 SV=2	0,436993371	3,875745
P24941	Cyclin-dependent kinase 2 OS=Homo sapiens OX=9606 GN=CDK2 PE=1 SV=2	-0,29199582	3,873644
Q15154	Pericentriolar material 1 protein OS=Homo sapiens OX=9606 GN=PCM1 PE=1 SV=5	-0,231988696	3,869575
Q8TC07	TBC1 domain family member 15 OS=Homo sapiens OX=9606 GN=TBC1D15 PE=1 SV=2	0,657105401	3,861338
P61978	Heterogeneous nuclear ribonucleoprotein K OS=Homo sapiens OX=9606 GN=HNRNPK PE=1 SV=1	-0,216985474	3,856978
E7ETH6	Zinc finger protein 587B OS=Homo sapiens OX=9606 GN=ZNF587B PE=1 SV=1	1,679656127	3,848891
Q9HCD5	Nuclear receptor coactivator 5 OS=Homo sapiens OX=9606 GN=NCOA5 PE=1 SV=2	0,495641715	3,84657
Q8NEZ5	F-box only protein 22 OS=Homo sapiens OX=9606 GN=FBXO22 PE=1 SV=1	0,558851487	3,846519
Q6NVY1	3-hydroxyisobutyryl-CoA hydrolase, mitochondrial OS=Homo sapiens OX=9606 GN=HIBCH PE=1 SV=2	0,414163755	3,836782
P17612	cAMP-dependent protein kinase catalytic subunit alpha OS=Homo sapiens OX=9606 GN=PRKACA PE=1 SV=2	-0,363081536	3,830606
P14735	Insulin-degrading enzyme OS=Homo sapiens OX=9606 GN=IDE PE=1 SV=4	-0,220275178	3,826954
Q9NX40	OCIA domain-containing protein 1 OS=Homo sapiens OX=9606 GN=OCIAD1 PE=1 SV=1	-0,447896457	3,825434
P31942	Heterogeneous nuclear ribonucleoprotein H3 OS=Homo sapiens OX=9606 GN=HNRNPH3 PE=1 SV=2	-0,362693825	3,825393
O95235	Kinesin-like protein KIF20A OS=Homo sapiens OX=9606 GN=KIF20A PE=1 SV=1	-0,385381945	3,824468
Q9H4A4	Aminopeptidase B OS=Homo sapiens OX=9606 GN=RNPEP PE=1 SV=2	-0,207400368	3,822265
P04406	Glyceraldehyde-3-phosphate dehydrogenase OS=Homo sapiens OX=9606 GN=GAPDH PE=1 SV=3	0,219879562	3,817281
Q3ZCQ8	Mitochondrial import inner membrane translocase subunit TIM50 OS=Homo sapiens OX=9606 GN=TIMM50 PE=1 SV=2	-0,412274689	3,815396
P56192	Methionine--tRNA ligase, cytoplasmic OS=Homo sapiens OX=9606 GN=MARS PE=1 SV=2	0,224419285	3,811782
P28074	Proteasome subunit beta type-5 OS=Homo sapiens OX=9606 GN=PSMB5 PE=1 SV=3	0,361604895	3,810758
Q9Y3B4	Splicing factor 3B subunit 6 OS=Homo sapiens OX=9606 GN=SF3B6 PE=1 SV=1	-0,647976169	3,808031
O75665	Oral-facial-digital syndrome 1 protein OS=Homo sapiens OX=9606 GN=OFD1 PE=1 SV=1	-0,385283084	3,797737
Q14CX7	N-alpha-acetyltransferase 25, NatB auxiliary subunit OS=Homo sapiens OX=9606 GN=NAA25 PE=1 SV=1	-0,340928119	3,787567
Q9NUQ6	SPATS2-like protein OS=Homo sapiens OX=9606 GN=SPATS2L PE=1 SV=2	-0,245923663	3,78565
Q6P6B7	Ankyrin repeat domain-containing protein 16 OS=Homo sapiens OX=9606 GN=ANKRD16 PE=1 SV=1	-0,963439432	3,778834
P52895	Aldo-keto reductase family 1 member C2 OS=Homo sapiens OX=9606 GN=AKR1C2 PE=1 SV=3	0,297448108	3,774979
Q14141	Septin-6 OS=Homo sapiens OX=9606 GN=SEPTIN6 PE=1 SV=4	-0,642090419	3,773519
Q13740	CD166 antigen OS=Homo sapiens OX=9606 GN=ALCAM PE=1 SV=2	0,251671044	3,763673
P51532	Transcription activator BRG1 OS=Homo sapiens OX=9606 GN=SMARCA4 PE=1 SV=2	-0,266935476	3,76018
Q9H5Q4	Dimethyladenosine transferase 2, mitochondrial OS=Homo sapiens OX=9606 GN=TFB2M PE=1 SV=1	-0,801339244	3,755923
Q9H0V9	VIP36-like protein OS=Homo sapiens OX=9606 GN=LMAN2L PE=1 SV=1	0,485250602	3,754583
Q9H3S7	Tyrosine-protein phosphatase non-receptor type 23 OS=Homo sapiens OX=9606 GN=PTPN23 PE=1 SV=1	-0,251142565	3,752785
O00291	Huntingtin-interacting protein 1 OS=Homo sapiens OX=9606 GN=HIP1 PE=1 SV=5	-0,544375263	3,738818
Q9NRS6	Sorting nexin-15 OS=Homo sapiens OX=9606 GN=SNX15 PE=1 SV=1	0,518402039	3,737951
Q8IVD9	NudC domain-containing protein 3 OS=Homo sapiens OX=9606 GN=NUDCD3 PE=1 SV=3	-0,30707993	3,730868
Q9H5Z1	Probable ATP-dependent RNA helicase DHX35 OS=Homo sapiens OX=9606 GN=DHX35 PE=1 SV=2	0,639800937	3,730613

P49593	Protein phosphatase 1F OS=Homo sapiens OX=9606 GN=PPM1F PE=1 SV=3	-0,482499135	3,730198
Q12959	Disks large homolog 1 OS=Homo sapiens OX=9606 GN=DLG1 PE=1 SV=2	-0,294651314	3,727604
P13984	General transcription factor IIF subunit 2 OS=Homo sapiens OX=9606 GN=GTF2F2 PE=1 SV=2	-0,437596658	3,721029
Q9UBS4	DnaJ homolog subfamily B member 11 OS=Homo sapiens OX=9606 GN=DNAJB11 PE=1 SV=1	0,35446782	3,715044
Q7KZF4	Staphylococcal nuclease domain-containing protein 1 OS=Homo sapiens OX=9606 GN=SND1 PE=1 SV=1	-0,162591549	3,706629
Q08AF3	Schlafen family member 5 OS=Homo sapiens OX=9606 GN=SLFN5 PE=1 SV=1	-1,485594066	3,701498
Q9Y375	Complex I intermediate-associated protein 30, mitochondrial OS=Homo sapiens OX=9606 GN=NDUFAF1 PE=1 SV=2	0,921744574	3,696641
Q5VT79	Annexin A8-like protein 1 OS=Homo sapiens OX=9606 GN=ANXA8L1 PE=2 SV=2	-0,304494023	3,689289
O95433	Activator of 90 kDa heat shock protein ATPase homolog 1 OS=Homo sapiens OX=9606 GN=AHSA1 PE=1 SV=1	0,2717651	3,685702
Q08257	Quinone oxidoreductase OS=Homo sapiens OX=9606 GN=CRYZ PE=1 SV=1	0,536941252	3,683289
Q9H488	GDP-fucose protein O-fucosyltransferase 1 OS=Homo sapiens OX=9606 GN=POFUT1 PE=1 SV=1	-0,400329714	3,680351
P48449	Lanosterol synthase OS=Homo sapiens OX=9606 GN=LSS PE=1 SV=1	-0,262542593	3,676022
P57740	Nuclear pore complex protein Nup107 OS=Homo sapiens OX=9606 GN=NUP107 PE=1 SV=1	-0,27043263	3,66122
O95104	SR-related and CTD-associated factor 4 OS=Homo sapiens OX=9606 GN=SCAF4 PE=1 SV=3	-0,533948373	3,660894
O15270	Serine palmitoyltransferase 2 OS=Homo sapiens OX=9606 GN=SPTLC2 PE=1 SV=1	0,622828117	3,659796
O00148	ATP-dependent RNA helicase DDX39A OS=Homo sapiens OX=9606 GN=DDX39A PE=1 SV=2	-0,239911101	3,658204
P28799	Progranulin OS=Homo sapiens OX=9606 GN=GRN PE=1 SV=2	0,349943271	3,654559
P35612	Beta-adducin OS=Homo sapiens OX=9606 GN=ADD2 PE=1 SV=3	-0,253166379	3,646994
P02458	#N/D	-0,723909918	3,639558
Q9BSY4	Coiled-coil-helix-coiled-coil-helix domain-containing protein 5 OS=Homo sapiens OX=9606 GN=CHCHD5 PE=1 SV=1	-1,021058742	3,636257
Q9H0A0	RNA cytidine acetyltransferase OS=Homo sapiens OX=9606 GN=NAT10 PE=1 SV=2	-0,208077385	3,635652
Q8N8A6	ATP-dependent RNA helicase DDX51 OS=Homo sapiens OX=9606 GN=DDX51 PE=1 SV=3	-1,058108236	3,630664
Q8TDQ7	Glucosamine-6-phosphate isomerase 2 OS=Homo sapiens OX=9606 GN=GNPDA2 PE=1 SV=1	-0,771158521	3,630483
Q14684	Ribosomal RNA processing protein 1 homolog B OS=Homo sapiens OX=9606 GN=RRP1B PE=1 SV=3	-0,314275894	3,628879
Q96IZ0	PRKC apoptosis WT1 regulator protein OS=Homo sapiens OX=9606 GN=PAWR PE=1 SV=1	0,395463692	3,625439
P27797	Calreticulin OS=Homo sapiens OX=9606 GN=CALR PE=1 SV=1	0,211859997	3,623034
Q658P3	Metalloreductase STEAP3 OS=Homo sapiens OX=9606 GN=STEAP3 PE=1 SV=2	-0,470240417	3,62143
O00221	NF-kappa-B inhibitor epsilon OS=Homo sapiens OX=9606 GN=NFKBIE PE=1 SV=3	0,428351314	3,618599
Q9Y4X5	E3 ubiquitin-protein ligase ARIH1 OS=Homo sapiens OX=9606 GN=ARIH1 PE=1 SV=2	0,321121643	3,617751
O95372	Acyl-protein thioesterase 2 OS=Homo sapiens OX=9606 GN=LYPLA2 PE=1 SV=1	0,368361553	3,613787
Q96AY3	Peptidyl-prolyl cis-trans isomerase FKBP10 OS=Homo sapiens OX=9606 GN=FKBP10 PE=1 SV=1	-0,244270147	3,61209
O15069	NAC-alpha domain-containing protein 1 OS=Homo sapiens OX=9606 GN=NACAD PE=1 SV=3	1,122360909	3,605777
O00339	Matrilin-2 OS=Homo sapiens OX=9606 GN=MATN2 PE=1 SV=4	1,208610471	3,603807
Q9NZQ3	NCK-interacting protein with SH3 domain OS=Homo sapiens OX=9606 GN=NCKIPSD PE=1 SV=1	-0,966938342	3,603159
P62753	40S ribosomal protein S6 OS=Homo sapiens OX=9606 GN=RPS6 PE=1 SV=1	0,39321788	3,600118
P28331	NADH-ubiquinone oxidoreductase 75 kDa subunit, mitochondrial OS=Homo sapiens OX=9606 GN=NDUFS1 PE=1 SV=3	-0,220294254	3,599613
P49792	E3 SUMO-protein ligase RanBP2 OS=Homo sapiens OX=9606 GN=RANBP2 PE=1 SV=2	-0,155559464	3,596516
Q9BVQ7	Spermatogenesis-associated protein 5-like protein 1 OS=Homo sapiens OX=9606 GN=SPATA5L1 PE=1 SV=2	0,252552427	3,59274
P36776	Lon protease homolog, mitochondrial OS=Homo sapiens OX=9606 GN=LONP1 PE=1 SV=2	0,202262753	3,591547
P27695	DNA-(apurinic or apyrimidinic site) lyase OS=Homo sapiens OX=9606 GN=APEX1 PE=1 SV=2	-0,32716271	3,590529
Q9ULX3	RNA-binding protein NOB1 OS=Homo sapiens OX=9606 GN=NOB1 PE=1 SV=1	-0,524227529	3,587998
P51858	Hepatoma-derived growth factor OS=Homo sapiens OX=9606 GN=HDGF PE=1 SV=1	-0,266191335	3,583599
Q92995	Ubiquitin carboxyl-terminal hydrolase 13 OS=Homo sapiens OX=9606 GN=USP13 PE=1 SV=2	-0,609964148	3,583196
Q8NCF5	NFATC2-interacting protein OS=Homo sapiens OX=9606 GN=NFATC2IP PE=1 SV=1	-1,123707664	3,581545
Q96P11	Probable 28S rRNA (cytosine-C(5))-methyltransferase OS=Homo sapiens OX=9606 GN=NSUN5 PE=1 SV=2	-0,818597997	3,578628
Q9Y4C2	TRPM8 channel-associated factor 1 OS=Homo sapiens OX=9606 GN=TCAF1 PE=1 SV=3	-0,326194223	3,576741
P61513	60S ribosomal protein L37a OS=Homo sapiens OX=9606 GN=RPL37A PE=1 SV=2	-0,608206659	3,572691
P13489	Ribonuclease inhibitor OS=Homo sapiens OX=9606 GN=RNH1 PE=1 SV=2	0,21870971	3,563544
O95239	Chromosome-associated kinesin KIF4A OS=Homo sapiens OX=9606 GN=KIF4A PE=1 SV=3	-0,223676661	3,55921
O43592	Exportin-T OS=Homo sapiens OX=9606 GN=XPOT PE=1 SV=2	0,223440588	3,553989
Q4G0J3	La-related protein 7 OS=Homo sapiens OX=9606 GN=LARP7 PE=1 SV=1	-0,518837414	3,547479
O95347	Structural maintenance of chromosomes protein 2 OS=Homo sapiens OX=9606 GN=SMC2 PE=1 SV=2	-0,163406143	3,539331
Q69YN2	CWF19-like protein 1 OS=Homo sapiens OX=9606 GN=CWF19L1 PE=1 SV=2	-0,460839362	3,537899
P19387	DNA-directed RNA polymerase II subunit RPB3 OS=Homo sapiens OX=9606 GN=POLR2C PE=1 SV=2	-0,516110059	3,526951
Q86U44	N6-adenosine-methyltransferase catalytic subunit OS=Homo sapiens OX=9606 GN=METTL3 PE=1 SV=2	0,314655247	3,522415
P49418	Amphiphysin OS=Homo sapiens OX=9606 GN=AMPH PE=1 SV=1	0,339848304	3,520187
Q96II8	Leucine-rich repeat and calponin homology domain-containing protein 3 OS=Homo sapiens OX=9606 GN=LRCH3 PE=1 SV=	0,417368156	3,516122
Q13619	Cullin-4A OS=Homo sapiens OX=9606 GN=CUL4A PE=1 SV=3	0,281941742	3,51421
P49903	Selenide, water dikinase 1 OS=Homo sapiens OX=9606 GN=SEPHS1 PE=1 SV=2	0,383480555	3,490513
O94822	E3 ubiquitin-protein ligase listerin OS=Homo sapiens OX=9606 GN=LTN1 PE=1 SV=6	0,226005024	3,490276
O43488	Aflatoxin B1 aldehyde reductase member 2 OS=Homo sapiens OX=9606 GN=AKR7A2 PE=1 SV=3	-0,45502426	3,486191
A0JNW5	UHRF1-binding protein 1-like OS=Homo sapiens OX=9606 GN=UHRF1BP1L PE=1 SV=2	0,72015227	3,482057
Q96EK7	Constitutive coactivator of peroxisome proliferator-activated receptor gamma OS=Homo sapiens OX=9606 GN=FAM120B	-0,876818331	3,475683
Q02218	2-oxoglutarate dehydrogenase, mitochondrial OS=Homo sapiens OX=9606 GN=OGDH PE=1 SV=3	-0,190334706	3,473596
P17096	High mobility group protein HMG-I/HMG-Y OS=Homo sapiens OX=9606 GN=HMGA1 PE=1 SV=3	-0,73257828	3,466357
Q16537	Serine/threonine-protein phosphatase 2A 56 kDa regulatory subunit epsilon isoform OS=Homo sapiens OX=9606 GN=PPP	-0,452135868	3,460497
O95352	Ubiquitin-like modifier-activating enzyme ATG7 OS=Homo sapiens OX=9606 GN=ATG7 PE=1 SV=1	-0,355751072	3,45867
Q15477	Helicase SKI2W OS=Homo sapiens OX=9606 GN=SKIV2L PE=1 SV=3	-0,411471821	3,456464
Q16270	Insulin-like growth factor-binding protein 7 OS=Homo sapiens OX=9606 GN=IGFBP7 PE=1 SV=1	0,451336784	3,453388
Q14966	Zinc finger protein 638 OS=Homo sapiens OX=9606 GN=ZNF638 PE=1 SV=2	0,506261065	3,452683
Q68CZ2	Tensin-3 OS=Homo sapiens OX=9606 GN=TNS3 PE=1 SV=2	-0,213684714	3,44998
Q9UPY8	Microtubule-associated protein RP/EB family member 3 OS=Homo sapiens OX=9606 GN=MAPRE3 PE=1 SV=1	0,379772909	3,448862
Q8TCZ2	CD99 antigen-like protein 2 OS=Homo sapiens OX=9606 GN=CD99L2 PE=1 SV=1	-0,587595836	3,448791
Q9GZM5	Protein YIPF3 OS=Homo sapiens OX=9606 GN=YIPF3 PE=1 SV=1	-1,050625083	3,439369
O95302	Peptidyl-prolyl cis-trans isomerase FKBP9 OS=Homo sapiens OX=9606 GN=FKBP9 PE=1 SV=2	-0,249583813	3,431022
Q2TAL8	Glutamine-rich protein 1 OS=Homo sapiens OX=9606 GN=QRICH1 PE=1 SV=1	-0,332975527	3,429194
O95340	Bifunctional 3'-phosphoadenosine 5'-phosphosulfate synthase 2 OS=Homo sapiens OX=9606 GN=PAPSS2 PE=1 SV=2	0,191962037	3,426627
Q9NZ08	Endoplasmic reticulum aminopeptidase 1 OS=Homo sapiens OX=9606 GN=ERAP1 PE=1 SV=3	-0,28792836	3,425555
P62910	60S ribosomal protein L32 OS=Homo sapiens OX=9606 GN=RPL32 PE=1 SV=2	-0,352522437	3,419107
P26368	Splicing factor U2AF 65 kDa subunit OS=Homo sapiens OX=9606 GN=U2AF2 PE=1 SV=4	-0,275899379	3,413893
O43299	AP-5 complex subunit zeta-1 OS=Homo sapiens OX=9606 GN=AP5Z1 PE=1 SV=2	-0,535499064	3,410555
Q9UDY8	Mucosa-associated lymphoid tissue lymphoma translocation protein 1 OS=Homo sapiens OX=9606 GN=MALT1 PE=1 SV=1	0,350844165	3,398567
P78347	General transcription factor II-I OS=Homo sapiens OX=9606 GN=GTF2I PE=1 SV=2	-0,158820656	3,398139
P37802	Transgelin-2 OS=Homo sapiens OX=9606 GN=TAGLN2 PE=1 SV=3	0,211366574	3,398016
P46379	Large proline-rich protein BAG6 OS=Homo sapiens OX=9606 GN=BAG6 PE=1 SV=2	-0,221652743	3,397624
O43824	Putative GTP-binding protein 6 OS=Homo sapiens OX=9606 GN=GTPBP6 PE=2 SV=4	-0,73510046	3,391686
P40692	DNA mismatch repair protein Mlh1 OS=Homo sapiens OX=9606 GN=MLH1 PE=1 SV=1	-0,330124814	3,391574
P78417	Glutathione S-transferase omega-1 OS=Homo sapiens OX=9606 GN=GSTO1 PE=1 SV=2	-0,273122619	3,368048
Q9UKV5	E3 ubiquitin-protein ligase AMFR OS=Homo sapiens OX=9606 GN=AMFR PE=1 SV=2	-0,889774622	3,365167
P18754	Regulator of chromosome condensation OS=Homo sapiens OX=9606 GN=RCC1 PE=1 SV=1	-0,440523578	3,357154
P68032	Actin, alpha cardiac muscle 1 OS=Homo sapiens OX=9606 GN=ACTC1 PE=1 SV=1	0,707111736	3,355673

Q86V21	Acetoacetyl-CoA synthetase OS=Homo sapiens OX=9606 GN=AACS PE=1 SV=1	-0,347167659	3,353636
Q8IWZ3	Ankyrin repeat and KH domain-containing protein 1 OS=Homo sapiens OX=9606 GN=ANKHD1 PE=1 SV=1	-0,271612778	3,343189
P29372	DNA-3-methyladenine glycosylase OS=Homo sapiens OX=9606 GN=MPG PE=1 SV=3	-0,570190422	3,343178
Q86Y56	Dynein assembly factor 5, axonemal OS=Homo sapiens OX=9606 GN=DNAAF5 PE=1 SV=4	-0,208901172	3,343049
Q32P28	Prolyl 3-hydroxylase 1 OS=Homo sapiens OX=9606 GN=P3H1 PE=1 SV=2	0,230613638	3,337544
Q6P9B6	MTOR-associated protein MEAK7 OS=Homo sapiens OX=9606 GN=MEAK7 PE=1 SV=2	0,437737314	3,332349
Q86Y37	CDK2-associated and cullin domain-containing protein 1 OS=Homo sapiens OX=9606 GN=CACUL1 PE=1 SV=1	-0,568254265	3,331375
O94763	Unconventional prefoldin RPB5 interactor 1 OS=Homo sapiens OX=9606 GN=URI1 PE=1 SV=3	-0,568009216	3,329881
Q04206	Transcription factor p65 OS=Homo sapiens OX=9606 GN=RELA PE=1 SV=2	-0,344267136	3,318252
Q9Y5Z7	Host cell factor 2 OS=Homo sapiens OX=9606 GN=HCFC2 PE=1 SV=1	1,014502957	3,316483
Q5VZM2	Ras-related GTP-binding protein B OS=Homo sapiens OX=9606 GN=RRAGB PE=1 SV=1	0,301922209	3,315759
Q53GL7	Protein mono-ADP-ribosyltransferase PARP10 OS=Homo sapiens OX=9606 GN=PARP10 PE=1 SV=2	0,998796518	3,313811
O94979	Protein transport protein Sec31A OS=Homo sapiens OX=9606 GN=SEC31A PE=1 SV=3	0,172160184	3,312131
Q9Y277	Voltage-dependent anion-selective channel protein 3 OS=Homo sapiens OX=9606 GN=VDAC3 PE=1 SV=1	0,307405562	3,311372
Q9UIQ6	Leucyl-cystinyl aminopeptidase OS=Homo sapiens OX=9606 GN=LNPEP PE=1 SV=3	-0,242989869	3,308299
P13473	Lysosome-associated membrane glycoprotein 2 OS=Homo sapiens OX=9606 GN=LAMP2 PE=1 SV=2	0,694459175	3,299985
Q00577	Transcriptional activator protein Pur-alpha OS=Homo sapiens OX=9606 GN=PURA PE=1 SV=2	-0,395707016	3,297208
Q9UHB6	LIM domain and actin-binding protein 1 OS=Homo sapiens OX=9606 GN=LIMA1 PE=1 SV=1	0,211218269	3,287185
Q13439	Golgin subfamily A member 4 OS=Homo sapiens OX=9606 GN=GOLGA4 PE=1 SV=1	0,201790332	3,285551
P13010	X-ray repair cross-complementing protein 5 OS=Homo sapiens OX=9606 GN=XRCC5 PE=1 SV=3	-0,159227904	3,285383
Q9BTE3	Mini-chromosome maintenance complex-binding protein OS=Homo sapiens OX=9606 GN=MCMBP PE=1 SV=2	-0,258347523	3,284931
P78346	Ribonuclease P protein subunit p30 OS=Homo sapiens OX=9606 GN=RPP30 PE=1 SV=1	-0,432295383	3,283899
Q86WI1	Fibrocystin-L OS=Homo sapiens OX=9606 GN=PKHD1L1 PE=2 SV=2	0,82660843	3,279395
P82650	28S ribosomal protein S22, mitochondrial OS=Homo sapiens OX=9606 GN=MRPS22 PE=1 SV=1	-0,483036246	3,276764
P14854	Cytochrome c oxidase subunit 6B1 OS=Homo sapiens OX=9606 GN=COX6B1 PE=1 SV=2	0,687480377	3,269072
Q9NUL3	Double-stranded RNA-binding protein Staufen homolog 2 OS=Homo sapiens OX=9606 GN=STAU2 PE=1 SV=2	0,555633735	3,254186
Q5JSZ5	Protein PRRC2B OS=Homo sapiens OX=9606 GN=PRRC2B PE=1 SV=2	-0,683689377	3,25222
O15397	Importin-8 OS=Homo sapiens OX=9606 GN=IPO8 PE=1 SV=2	-0,266036731	3,251807
P26006	Integrin alpha-3 OS=Homo sapiens OX=9606 GN=ITGA3 PE=1 SV=5	-0,204748732	3,251171
Q9Y512	Sorting and assembly machinery component 50 homolog OS=Homo sapiens OX=9606 GN=SAMM50 PE=1 SV=3	0,554298041	3,245991
P16615	Sarcoplasmic/endoplasmic reticulum calcium ATPase 2 OS=Homo sapiens OX=9606 GN=ATP2A2 PE=1 SV=1	0,199867371	3,242097
P26639	Threonine--tRNA ligase, cytoplasmic OS=Homo sapiens OX=9606 GN=TARS PE=1 SV=3	0,166943691	3,24066
Q9Y570	Protein phosphatase methylesterase 1 OS=Homo sapiens OX=9606 GN=PPME1 PE=1 SV=3	0,231779667	3,232329
P68371	Tubulin beta-4B chain OS=Homo sapiens OX=9606 GN=TUBB4B PE=1 SV=1	-0,313519694	3,22434
Q9UFC0	Leucine-rich repeat and WD repeat-containing protein 1 OS=Homo sapiens OX=9606 GN=LRWD1 PE=1 SV=2	0,550512882	3,222741
O94808	Glutamine--fructose-6-phosphate aminotransferase [isomerizing] 2 OS=Homo sapiens OX=9606 GN=GFPT2 PE=1 SV=3	-0,264242646	3,222551
Q8NBU5	ATPase family AAA domain-containing protein 1 OS=Homo sapiens OX=9606 GN=ATAD1 PE=1 SV=1	-0,475717357	3,221123
Q8N128	Protein FAM177A1 OS=Homo sapiens OX=9606 GN=FAM177A1 PE=1 SV=1	0,897635448	3,217496
Q9H853	Putative tubulin-like protein alpha-4B OS=Homo sapiens OX=9606 GN=TUBA4B PE=5 SV=2	1,878710037	3,215191
P61221	ATP-binding cassette sub-family E member 1 OS=Homo sapiens OX=9606 GN=ABCE1 PE=1 SV=1	-0,212891942	3,213657
Q15910	Histone-lysine N-methyltransferase EZH2 OS=Homo sapiens OX=9606 GN=EZH2 PE=1 SV=2	-0,674307341	3,210336
Q96RL7	Vacuolar protein sorting-associated protein 13A OS=Homo sapiens OX=9606 GN=VPS13A PE=1 SV=2	0,274097912	3,209089
O95865	N(G),N(G)-dimethylarginine dimethylaminohydrolase 2 OS=Homo sapiens OX=9606 GN=DDAH2 PE=1 SV=1	0,299511845	3,201169
Q86X02	Cerebellar degeneration-related protein 2-like OS=Homo sapiens OX=9606 GN=CDR2L PE=1 SV=2	-0,72634613	3,198286
Q9Y6W5	Wiskott-Aldrich syndrome protein family member 2 OS=Homo sapiens OX=9606 GN=WASF2 PE=1 SV=3	0,315370285	3,192745
P37235	Hippocalcin-like protein 1 OS=Homo sapiens OX=9606 GN=HPCAL1 PE=1 SV=3	-0,385187804	3,191205
P06865	Beta-hexosaminidase subunit alpha OS=Homo sapiens OX=9606 GN=HEXA PE=1 SV=2	-0,421732448	3,189853
P31948	Stress-induced-phosphoprotein 1 OS=Homo sapiens OX=9606 GN=STIP1 PE=1 SV=1	0,153854807	3,188743
P52292	Importin subunit alpha-1 OS=Homo sapiens OX=9606 GN=KPNA2 PE=1 SV=1	-0,23642679	3,187503
Q08209	Serine/threonine-protein phosphatase 2B catalytic subunit alpha isoform OS=Homo sapiens OX=9606 GN=PPP3CA PE=1 S	-0,470227656	3,179333
Q8IVH2	Forkhead box protein P4 OS=Homo sapiens OX=9606 GN=FOXP4 PE=1 SV=1	-0,834677688	3,173035
Q15942	Zyxin OS=Homo sapiens OX=9606 GN=ZYX PE=1 SV=1	-0,243190221	3,171075
Q14562	ATP-dependent RNA helicase DHX8 OS=Homo sapiens OX=9606 GN=DHX8 PE=1 SV=1	-0,419533954	3,170282
Q9BPX3	Condensin complex subunit 3 OS=Homo sapiens OX=9606 GN=NCAPG PE=1 SV=1	-0,181491651	3,167389
P35610	Sterol O-acyltransferase 1 OS=Homo sapiens OX=9606 GN=SOAT1 PE=1 SV=3	0,354491084	3,166325
Q9BQ61	Telomerase RNA component interacting RNase OS=Homo sapiens OX=9606 GN=TRIR PE=1 SV=1	-0,541350863	3,166305
Q9NPL8	Complex I assembly factor TIMMDC1, mitochondrial OS=Homo sapiens OX=9606 GN=TIMMDC1 PE=1 SV=2	-0,939542652	3,164405
Q6L8Q7	2',5'-phosphodiesterase 12 OS=Homo sapiens OX=9606 GN=PDE12 PE=1 SV=2	0,540133719	3,158791
P50225	Sulfotransferase 1A1 OS=Homo sapiens OX=9606 GN=SULT1A1 PE=1 SV=3	-0,417778089	3,154652
Q07065	Cytoskeleton-associated protein 4 OS=Homo sapiens OX=9606 GN=CKAP4 PE=1 SV=2	0,163723126	3,152821
P62277	40S ribosomal protein S13 OS=Homo sapiens OX=9606 GN=RPS13 PE=1 SV=2	-0,330189254	3,147361
P52298	Nuclear cap-binding protein subunit 2 OS=Homo sapiens OX=9606 GN=NCBP2 PE=1 SV=1	0,46581513	3,14571
P43155	Carnitine O-acetyltransferase OS=Homo sapiens OX=9606 GN=CRAT PE=1 SV=5	-0,295338921	3,143242
Q9NNX1	Tuftelin OS=Homo sapiens OX=9606 GN=TUFT1 PE=1 SV=1	-0,537193404	3,140623
Q9BRJ6	Uncharacterized protein C7orf50 OS=Homo sapiens OX=9606 GN=C7orf50 PE=1 SV=1	-0,536760883	3,137949
P61289	Proteasome activator complex subunit 3 OS=Homo sapiens OX=9606 GN=PSME3 PE=1 SV=1	-0,294539151	3,132167
Q9Y617	Phosphoserine aminotransferase OS=Homo sapiens OX=9606 GN=PSAT1 PE=1 SV=2	0,226307389	3,128756
P61225	Ras-related protein Rap-2b OS=Homo sapiens OX=9606 GN=RAP2B PE=1 SV=1	-0,925559189	3,128193
Q9Y5K6	CD2-associated protein OS=Homo sapiens OX=9606 GN=CD2AP PE=1 SV=1	0,463182426	3,125636
Q8TEX9	Importin-4 OS=Homo sapiens OX=9606 GN=IPO4 PE=1 SV=2	-0,208531648	3,123385
P28838	Cytosol aminopeptidase OS=Homo sapiens OX=9606 GN=LAP3 PE=1 SV=3	-0,268224726	3,118264
P51153	Ras-related protein Rab-13 OS=Homo sapiens OX=9606 GN=RAB13 PE=1 SV=1	-0,65329505	3,115613
P23588	Eukaryotic translation initiation factor 4B OS=Homo sapiens OX=9606 GN=EIF4B PE=1 SV=2	-0,208085855	3,114205
Q14332	Frizzled-2 OS=Homo sapiens OX=9606 GN=FZD2 PE=1 SV=1	-0,651533031	3,107612
O43390	Heterogeneous nuclear ribonucleoprotein R OS=Homo sapiens OX=9606 GN=HNRNPR PE=1 SV=1	-0,207702493	3,106317
O95373	Importin-7 OS=Homo sapiens OX=9606 GN=IPO7 PE=1 SV=1	0,155076874	3,104294
P02751	Fibronectin OS=Homo sapiens OX=9606 GN=FN1 PE=1 SV=4	0,30801516	3,102474
Q0VDF9	Heat shock 70 kDa protein 14 OS=Homo sapiens OX=9606 GN=HSPA14 PE=1 SV=1	-0,247415938	3,099548
Q6P587	Acylpyruvase FAHD1, mitochondrial OS=Homo sapiens OX=9606 GN=FAHD1 PE=1 SV=2	-0,649609333	3,098867
Q9H1E5	Thioredoxin-related transmembrane protein 4 OS=Homo sapiens OX=9606 GN=TMX4 PE=1 SV=1	-0,913839645	3,097554
P48060	Glioma pathogenesis-related protein 1 OS=Homo sapiens OX=9606 GN=GLIPR1 PE=1 SV=3	1,298500916	3,096635
P51580	Thiopurine S-methyltransferase OS=Homo sapiens OX=9606 GN=TPMT PE=1 SV=1	-0,529731671	3,094419
Q96PZ0	Pseudouridylate synthase 7 homolog OS=Homo sapiens OX=9606 GN=PUS7 PE=1 SV=2	-0,912545464	3,094154
O75380	NADH dehydrogenase [ubiquinone] iron-sulfur protein 6, mitochondrial OS=Homo sapiens OX=9606 GN=NDUFS6 PE=1 SV	-0,648403437	3,093379
Q9NYV4	Cyclin-dependent kinase 12 OS=Homo sapiens OX=9606 GN=CDK12 PE=1 SV=2	-0,386066211	3,092857
P07996	Thrombospondin-1 OS=Homo sapiens OX=9606 GN=THBS1 PE=1 SV=2	0,231201351	3,092317
O95166	Gamma-aminobutyric acid receptor-associated protein OS=Homo sapiens OX=9606 GN=GABARAP PE=1 SV=1	0,399037738	3,091759
Q8WY22	BRI3-binding protein OS=Homo sapiens OX=9606 GN=BRI3BP PE=1 SV=1	-0,462295067	3,091495
P30260	Cell division cycle protein 27 homolog OS=Homo sapiens OX=9606 GN=CDC27 PE=1 SV=2	0,375164542	3,09045
O14950	Myosin regulatory light chain 12B OS=Homo sapiens OX=9606 GN=MYL12B PE=1 SV=2	0,306926342	3,088367
Q9Y5K5	Ubiquitin carboxyl-terminal hydrolase isozyme L5 OS=Homo sapiens OX=9606 GN=UCHL5 PE=1 SV=3	-0,277835409	3,086327

P78330	Phosphoserine phosphatase OS=Homo sapiens OX=9606 GN=PSPH PE=1 SV=2	0,457257588	3,080428
Q9NUJ3	T-complex protein 11-like protein 1 OS=Homo sapiens OX=9606 GN=TCP11L1 PE=1 SV=1	0,346559242	3,078301
Q7L2E3	ATP-dependent RNA helicase DHX30 OS=Homo sapiens OX=9606 GN=DHX30 PE=1 SV=1	-0,181130317	3,077096
Q9Y2S7	Polymerase delta-interacting protein 2 OS=Homo sapiens OX=9606 GN=POLDIP2 PE=1 SV=1	-0,373824708	3,077003
Q9H7B2	Ribosome production factor 2 homolog OS=Homo sapiens OX=9606 GN=RPF2 PE=1 SV=2	-0,526848938	3,076531
Q02750	Dual specificity mitogen-activated protein kinase kinase 1 OS=Homo sapiens OX=9606 GN=MAP2K1 PE=1 SV=2	0,346349394	3,075976
Q9BQ39	ATP-dependent RNA helicase DDX50 OS=Homo sapiens OX=9606 GN=DDX50 PE=1 SV=1	-0,255170252	3,075719
Q9Y679	Ancient ubiquitous protein 1 OS=Homo sapiens OX=9606 GN=AUP1 PE=1 SV=2	-0,456215393	3,072471
P42566	Epidermal growth factor receptor substrate 15 OS=Homo sapiens OX=9606 GN=EPS15 PE=1 SV=2	0,276837988	3,071773
Q92558	Wiskott-Aldrich syndrome protein family member 1 OS=Homo sapiens OX=9606 GN=WASF1 PE=1 SV=1	-1,213011166	3,07138
Q13043	Serine/threonine-protein kinase 4 OS=Homo sapiens OX=9606 GN=STK4 PE=1 SV=2	-0,323568205	3,067511
Q9H4L5	Oxysterol-binding protein-related protein 3 OS=Homo sapiens OX=9606 GN=OSBPL3 PE=1 SV=1	0,407880882	3,066579
Q9C0B0	RING finger protein unkempt homolog OS=Homo sapiens OX=9606 GN=UNK PE=1 SV=2	0,642258692	3,065353
Q9NYL9	Tropomodulin-3 OS=Homo sapiens OX=9606 GN=TMOD3 PE=1 SV=1	0,237065498	3,063759
A5PLN9	Trafficking protein particle complex subunit 13 OS=Homo sapiens OX=9606 GN=TRAPPC13 PE=1 SV=2	-0,900918704	3,063465
P78527	DNA-dependent protein kinase catalytic subunit OS=Homo sapiens OX=9606 GN=PRKDC PE=1 SV=3	-0,070240109	3,061061
O94855	Protein transport protein Sec24D OS=Homo sapiens OX=9606 GN=SEC24D PE=1 SV=2	0,288819441	3,053218
P57764	Gasdermin-D OS=Homo sapiens OX=9606 GN=GSDMD PE=1 SV=1	-0,371311267	3,051791
Q03164	Histone-lysine N-methyltransferase 2A OS=Homo sapiens OX=9606 GN=KMT2A PE=1 SV=5	-0,602367036	3,051233
Q7Z3E5	LisH domain-containing protein ARMC9 OS=Homo sapiens OX=9606 GN=ARMC9 PE=1 SV=3	0,406109165	3,050819
P52732	Kinesin-like protein KIF11 OS=Homo sapiens OX=9606 GN=KIF11 PE=1 SV=2	-0,236152045	3,047849
Q9NR09	Baculoviral IAP repeat-containing protein 6 OS=Homo sapiens OX=9606 GN=BIRC6 PE=1 SV=2	-0,141759429	3,047036
O43395	U4/U6 small nuclear ribonucleoprotein Prp3 OS=Homo sapiens OX=9606 GN=PRPF3 PE=1 SV=2	-0,263582504	3,046961
P60981	Destrin OS=Homo sapiens OX=9606 GN=DSTN PE=1 SV=3	0,274963792	3,044471
Q9Y2B9	cAMP-dependent protein kinase inhibitor gamma OS=Homo sapiens OX=9606 GN=PKIG PE=2 SV=1	-0,906199019	3,042702
Q9UJS0	Calcium-binding mitochondrial carrier protein Aralar2 OS=Homo sapiens OX=9606 GN=SLC25A13 PE=1 SV=2	-0,303363742	3,042303
Q9H9J2	39S ribosomal protein L44, mitochondrial OS=Homo sapiens OX=9606 GN=MRPL44 PE=1 SV=1	-0,521100585	3,0408
Q16186	Proteasomal ubiquitin receptor ADRM1 OS=Homo sapiens OX=9606 GN=ADRM1 PE=1 SV=2	0,342970601	3,038587
Q8TB61	Adenosine 3'-phospho 5'-phosphosulfate transporter 1 OS=Homo sapiens OX=9606 GN=SLC35B2 PE=1 SV=1	-0,369906604	3,03771
Q9UJZ1	Stomatin-like protein 2, mitochondrial OS=Homo sapiens OX=9606 GN=STOML2 PE=1 SV=1	-0,342754486	3,036198
Q01658	Protein Dr1 OS=Homo sapiens OX=9606 GN=DR1 PE=1 SV=1	-0,890465835	3,035647
P61758	Prefoldin subunit 3 OS=Homo sapiens OX=9606 GN=VBP1 PE=1 SV=4	-0,342570643	3,034165
Q9NPA0	ER membrane protein complex subunit 7 OS=Homo sapiens OX=9606 GN=EMC7 PE=1 SV=1	-0,888882567	3,031414
O43324	Eukaryotic translation elongation factor 1 epsilon-1 OS=Homo sapiens OX=9606 GN=EEF1E1 PE=1 SV=1	-0,369110455	3,029731
Q15274	Nicotinate-nucleotide pyrophosphorylase [carboxylating] OS=Homo sapiens OX=9606 GN=QPRT PE=1 SV=3	-0,368968816	3,028312
P25686	DnaJ homolog subfamily B member 2 OS=Homo sapiens OX=9606 GN=DNAJB2 PE=1 SV=3	0,886719251	3,025623
Q96G03	Phosphoglucomutase-2 OS=Homo sapiens OX=9606 GN=PGM2 PE=1 SV=4	-0,301889948	3,023288
P62750	60S ribosomal protein L23a OS=Homo sapiens OX=9606 GN=RPL23A PE=1 SV=1	-0,319573765	3,019512
O75643	U5 small nuclear ribonucleoprotein 200 kDa helicase OS=Homo sapiens OX=9606 GN=SNRNP200 PE=1 SV=2	-0,099729302	3,018067
Q9H3R5	Centromere protein H OS=Homo sapiens OX=9606 GN=CENPH PE=1 SV=1	-0,882535234	3,014396
P40123	Adenylyl cyclase-associated protein 2 OS=Homo sapiens OX=9606 GN=CAP2 PE=1 SV=1	0,251107328	3,010582
P62937	Peptidyl-prolyl cis-trans isomerase A OS=Homo sapiens OX=9606 GN=PPIA PE=1 SV=2	0,261135519	3,009551
Q92636	Protein FAN OS=Homo sapiens OX=9606 GN=NSMAF PE=1 SV=2	-0,875074109	3,009485
Q9BV20	Methylthioribose-1-phosphate isomerase OS=Homo sapiens OX=9606 GN=MRI1 PE=1 SV=1	0,629243183	3,005634
Q96HA8	Protein N-terminal glutamine amidohydrolase OS=Homo sapiens OX=9606 GN=WDYHV1 PE=1 SV=2	-0,838666044	3,005621
Q7L273	BTB/POZ domain-containing protein KCTD9 OS=Homo sapiens OX=9606 GN=KCTD9 PE=1 SV=1	0,628639587	3,002853
Q14527	Helicase-like transcription factor OS=Homo sapiens OX=9606 GN=HLTF PE=1 SV=2	-0,202629419	3,002585
P52630	Signal transducer and activator of transcription 2 OS=Homo sapiens OX=9606 GN=STAT2 PE=1 SV=1	-0,39955864	2,992575
P35232	Prohibitin OS=Homo sapiens OX=9606 GN=PHB PE=1 SV=1	-0,240936041	2,991137
P11362	Fibroblast growth factor receptor 1 OS=Homo sapiens OX=9606 GN=FGFR1 PE=1 SV=3	-0,497175241	2,990732
Q15758	Neutral amino acid transporter B(0) OS=Homo sapiens OX=9606 GN=SLC1A5 PE=1 SV=2	0,398469253	2,982892
Q8TAT6	Nuclear protein localization protein 4 homolog OS=Homo sapiens OX=9606 GN=NPLOC4 PE=1 SV=3	-0,239967299	2,975027
Q9UPY6	#N/D	-0,310362092	2,974361
Q9Y5L0	Transportin-3 OS=Homo sapiens OX=9606 GN=TNPO3 PE=1 SV=3	-0,224652685	2,974301
Q9Y6G9	Cytoplasmic dynein 1 light intermediate chain 1 OS=Homo sapiens OX=9606 GN=DYNC1LI1 PE=1 SV=3	0,224597953	2,973321
Q8WUH2	Transforming growth factor-beta receptor-associated protein 1 OS=Homo sapiens OX=9606 GN=TGFBRAP1 PE=1 SV=1	-0,576806293	2,970113
Q99755	Phosphatidylinositol 4-phosphate 5-kinase type-1 alpha OS=Homo sapiens OX=9606 GN=PIP5K1A PE=1 SV=1	0,620656753	2,965974
O60346	PH domain leucine-rich repeat-containing protein phosphatase 1 OS=Homo sapiens OX=9606 GN=PHLPP1 PE=1 SV=3	2,05760342	2,962642
Q9NPJ6	Mediator of RNA polymerase II transcription subunit 4 OS=Homo sapiens OX=9606 GN=MED4 PE=1 SV=1	-0,460594774	2,96014
P82664	28S ribosomal protein S10, mitochondrial OS=Homo sapiens OX=9606 GN=MRPS10 PE=1 SV=2	-1,382663149	2,957577
Q58FF6	Putative heat shock protein HSP 90-beta 4 OS=Homo sapiens OX=9606 GN=HSP90AB4P PE=5 SV=1	0,360810928	2,957394
Q96EK5	KIF1-binding protein OS=Homo sapiens OX=9606 GN=KIF1BP PE=1 SV=1	0,22314337	2,947305
Q8WXA3	RUN and FYVE domain-containing protein 2 OS=Homo sapiens OX=9606 GN=RUFY2 PE=1 SV=3	-0,334459602	2,944697
P28161	Glutathione S-transferase Mu 2 OS=Homo sapiens OX=9606 GN=GSTM2 PE=1 SV=2	0,438874691	2,939913
Q9BW83	Intraflagellar transport protein 27 homolog OS=Homo sapiens OX=9606 GN=IFT27 PE=1 SV=1	0,29074853	2,939447
P61106	Ras-related protein Rab-14 OS=Homo sapiens OX=9606 GN=RAB14 PE=1 SV=4	0,222591047	2,937446
Q8N2K0	Lysophosphatidylserine lipase ABHD12 OS=Homo sapiens OX=9606 GN=ABHD12 PE=1 SV=2	-0,280365165	2,937378
O43707	Alpha-actinin-4 OS=Homo sapiens OX=9606 GN=ACTN4 PE=1 SV=2	0,136334288	2,931656
P39023	60S ribosomal protein L3 OS=Homo sapiens OX=9606 GN=RPL3 PE=1 SV=2	-0,204224164	2,931601
P85037	Forkhead box protein K1 OS=Homo sapiens OX=9606 GN=FOXK1 PE=1 SV=1	0,852064593	2,931563
Q5GLZ8	Probable E3 ubiquitin-protein ligase HERC4 OS=Homo sapiens OX=9606 GN=HERC4 PE=1 SV=1	-0,294432973	2,927465
Q5VU43	Myomegalin OS=Homo sapiens OX=9606 GN=PDE4DIP PE=1 SV=3	0,893888277	2,926692
Q03135	Caveolin-1 OS=Homo sapiens OX=9606 GN=CAV1 PE=1 SV=4	-0,436637302	2,92279
Q9Y237	Peptidyl-prolyl cis-trans isomerase NIMA-interacting 4 OS=Homo sapiens OX=9606 GN=PIN4 PE=1 SV=1	-0,502007642	2,921561
Q99832	T-complex protein 1 subunit eta OS=Homo sapiens OX=9606 GN=CCT7 PE=1 SV=2	0,152746838	2,920205
O00401	Neural Wiskott-Aldrich syndrome protein OS=Homo sapiens OX=9606 GN=WASL PE=1 SV=2	0,39133139	2,919484
P09669	Cytochrome c oxidase subunit 6C OS=Homo sapiens OX=9606 GN=COX6C PE=1 SV=2	-0,845412433	2,913226
Q9NYB0	Telomeric repeat-binding factor 2-interacting protein 1 OS=Homo sapiens OX=9606 GN=TERF2IP PE=1 SV=1	0,608936301	2,911503
Q9Y508	E3 ubiquitin-protein ligase RNF114 OS=Homo sapiens OX=9606 GN=RNF114 PE=1 SV=1	0,435103056	2,911046
O00170	AH receptor-interacting protein OS=Homo sapiens OX=9606 GN=AIP PE=1 SV=2	-0,278332899	2,909689
Q96C01	Protein FAM136A OS=Homo sapiens OX=9606 GN=FAM136A PE=1 SV=1	-0,434499344	2,906425
Q14152	Eukaryotic translation initiation factor 3 subunit A OS=Homo sapiens OX=9606 GN=EIF3A PE=1 SV=1	-0,10949151	2,905349
Q02543	60S ribosomal protein L18a OS=Homo sapiens OX=9606 GN=RPL18A PE=1 SV=2	-0,292656155	2,90473
Q9UK39	Nocturnin OS=Homo sapiens OX=9606 GN=NOCT PE=1 SV=2	0,660127615	2,902811
P08648	Integrin alpha-5 OS=Homo sapiens OX=9606 GN=ITGA5 PE=1 SV=2	0,235454483	2,900319
P63167	Dynein light chain 1, cytoplasmic OS=Homo sapiens OX=9606 GN=DYNLL1 PE=1 SV=1	-0,837750654	2,891992
P12110	Collagen alpha-2(VI) chain OS=Homo sapiens OX=9606 GN=COL6A2 PE=1 SV=4	0,604439181	2,890501
Q9UKD2	mRNA turnover protein 4 homolog OS=Homo sapiens OX=9606 GN=MRTO4 PE=1 SV=2	-0,354636613	2,885056
P82094	TATA element modulatory factor OS=Homo sapiens OX=9606 GN=TMF1 PE=1 SV=2	0,354486126	2,883556
Q7Z2T5	TRMT1-like protein OS=Homo sapiens OX=9606 GN=TRMT1L PE=1 SV=2	0,431460512	2,883158
Q6PCB7	Long-chain fatty acid transport protein 1 OS=Homo sapiens OX=9606 GN=SLC27A1 PE=1 SV=1	-0,495627903	2,881535

P61006	Ras-related protein Rab-8A OS=Homo sapiens OX=9606 GN=RAB8A PE=1 SV=1	0,495195408	2,878819
P49406	39S ribosomal protein L19, mitochondrial OS=Homo sapiens OX=9606 GN=MRPL19 PE=1 SV=2	-0,386304333	2,874866
Q9NX62	Inositol monophosphatase 3 OS=Homo sapiens OX=9606 GN=IMPAD1 PE=1 SV=1	-0,43015352	2,873149
Q969Z0	FAST kinase domain-containing protein 4 OS=Homo sapiens OX=9606 GN=TBRG4 PE=1 SV=1	-0,251343342	2,861104
Q15435	Protein phosphatase 1 regulatory subunit 7 OS=Homo sapiens OX=9606 GN=PPP1R7 PE=1 SV=1	0,250909493	2,854574
Q8TD16	Protein bicaudal D homolog 2 OS=Homo sapiens OX=9606 GN=BICD2 PE=1 SV=1	-0,273993216	2,850772
Q8TEQ6	Gem-associated protein 5 OS=Homo sapiens OX=9606 GN=GEMIN5 PE=1 SV=3	-0,165217685	2,849563
Q13148	TAR DNA-binding protein 43 OS=Homo sapiens OX=9606 GN=TARDBP PE=1 SV=1	-0,305274368	2,84883
P27144	Adenylate kinase 4, mitochondrial OS=Homo sapiens OX=9606 GN=AK4 PE=1 SV=1	0,6652595	2,84395
P49321	Nuclear autoantigenic sperm protein OS=Homo sapiens OX=9606 GN=NASP PE=1 SV=2	-0,199715461	2,843654
Q9Y316	Protein MEMO1 OS=Homo sapiens OX=9606 GN=MEMO1 PE=1 SV=1	0,287804438	2,842846
Q96FS4	Signal-induced proliferation-associated protein 1 OS=Homo sapiens OX=9606 GN=SIPA1 PE=1 SV=1	-0,483680968	2,84224
Q9UKN8	General transcription factor 3C polypeptide 4 OS=Homo sapiens OX=9606 GN=GTF3C4 PE=1 SV=2	-0,260719645	2,839005
P50851	Lipopolysaccharide-responsive and beige-like anchor protein OS=Homo sapiens OX=9606 GN=LRBA PE=1 SV=4	-1,882818381	2,838321
P17066	#N/D	0,816684931	2,832972
O14641	Segment polarity protein dishevelled homolog DVL-2 OS=Homo sapiens OX=9606 GN=DVL2 PE=1 SV=1	-0,424265138	2,828046
Q9Y6K5	2'-5'-oligoadenylate synthase 3 OS=Homo sapiens OX=9606 GN=OAS3 PE=1 SV=3	-0,380547847	2,82382
P49005	DNA polymerase delta subunit 2 OS=Homo sapiens OX=9606 GN=POLD2 PE=1 SV=1	-0,380494783	2,82335
Q8WZ82	Esterase OVCA2 OS=Homo sapiens OX=9606 GN=OVCA2 PE=1 SV=1	-0,323176342	2,820921
P33897	ATP-binding cassette sub-family D member 1 OS=Homo sapiens OX=9606 GN=ABCD1 PE=1 SV=2	-0,302909636	2,820787
P29084	Transcription initiation factor IIE subunit beta OS=Homo sapiens OX=9606 GN=GTF2E2 PE=1 SV=1	-0,348165259	2,820624
P46934	E3 ubiquitin-protein ligase NEDD4 OS=Homo sapiens OX=9606 GN=NEDD4 PE=1 SV=4	-0,379419063	2,813817
Q99733	Nucleosome assembly protein 1-like 4 OS=Homo sapiens OX=9606 GN=NAP1L4 PE=1 SV=1	0,271177605	2,812703
P07814	Bifunctional glutamate/proline--tRNA ligase OS=Homo sapiens OX=9606 GN=EPRS PE=1 SV=5	0,105437007	2,812553
Q9BW27	Nuclear pore complex protein Nup85 OS=Homo sapiens OX=9606 GN=NUP85 PE=1 SV=1	-0,258156577	2,802429
P32189	Glycerol kinase OS=Homo sapiens OX=9606 GN=GK PE=1 SV=3	-0,805696261	2,801809
Q07352	mRNA decay activator protein ZFP36L1 OS=Homo sapiens OX=9606 GN=ZFP36L1 PE=1 SV=1	0,585118095	2,799632
Q3KQV9	UDP-N-acetylhexosamine pyrophosphorylase-like protein 1 OS=Homo sapiens OX=9606 GN=UAP1L1 PE=1 SV=2	0,202707479	2,798789
P09001	39S ribosomal protein L3, mitochondrial OS=Homo sapiens OX=9606 GN=MRPL3 PE=1 SV=1	-0,482373349	2,798107
P27816	Microtubule-associated protein 4 OS=Homo sapiens OX=9606 GN=MAP4 PE=1 SV=3	-0,131822024	2,796169
P12814	Alpha-actinin-1 OS=Homo sapiens OX=9606 GN=ACTN1 PE=1 SV=2	0,113615398	2,795415
Q9BYN0	Sulfiredoxin-1 OS=Homo sapiens OX=9606 GN=SRXN1 PE=1 SV=2	0,584097543	2,794804
Q9UPX0	Protein turtle homolog B OS=Homo sapiens OX=9606 GN=IGSF9B PE=2 SV=2	-0,483549404	2,791603
P52306	Rap1 GTPase-GDP dissociation stimulator 1 OS=Homo sapiens OX=9606 GN=RAP1GDS1 PE=1 SV=3	-0,207900955	2,788419
P02795	Metallothionein-2 OS=Homo sapiens OX=9606 GN=MT2A PE=1 SV=1	-0,800796581	2,787831
Q4G0F5	Vacuolar protein sorting-associated protein 26B OS=Homo sapiens OX=9606 GN=VPS26B PE=1 SV=2	-0,344466757	2,783878
Q9GZZ1	N-alpha-acetyltransferase 50 OS=Homo sapiens OX=9606 GN=NAA50 PE=1 SV=1	0,319384321	2,779515
P19388	DNA-directed RNA polymerases I, II, and III subunit RPABC1 OS=Homo sapiens OX=9606 GN=POLR2E PE=1 SV=4	-0,34398485	2,779094
P12270	Nucleoprotein TPR OS=Homo sapiens OX=9606 GN=TPR PE=1 SV=3	-0,111235101	2,77836
Q86VN1	Vacuolar protein-sorting-associated protein 36 OS=Homo sapiens OX=9606 GN=VPS36 PE=1 SV=1	-0,479086542	2,777364
Q63HN8	E3 ubiquitin-protein ligase RNF213 OS=Homo sapiens OX=9606 GN=RNF213 PE=1 SV=3	-0,131043188	2,773044
Q9BSH4	Translational activator of cytochrome c oxidase 1 OS=Homo sapiens OX=9606 GN=TACO1 PE=1 SV=1	-0,416755165	2,770501
Q9UBF8	Phosphatidylinositol 4-kinase beta OS=Homo sapiens OX=9606 GN=PI4KB PE=1 SV=1	-0,476579213	2,761527
P07355	Annexin A2 OS=Homo sapiens OX=9606 GN=ANXA2 PE=1 SV=2	0,155960083	2,757842
Q9H2W6	39S ribosomal protein L46, mitochondrial OS=Homo sapiens OX=9606 GN=MRPL46 PE=1 SV=1	-0,789240672	2,754655
P14923	Junction plakoglobin OS=Homo sapiens OX=9606 GN=JUP PE=1 SV=3	-0,153279489	2,75344
P11172	Uridine 5'-monophosphate synthase OS=Homo sapiens OX=9606 GN=UMPS PE=1 SV=1	-0,226309657	2,75068
Q15063	Periostin OS=Homo sapiens OX=9606 GN=POSTN PE=1 SV=2	1,766258401	2,749247
Q9NYB9	Abl interactor 2 OS=Homo sapiens OX=9606 GN=ABI2 PE=1 SV=1	-0,474259015	2,746862
P21589	5'-nucleotidase OS=Homo sapiens OX=9606 GN=NT5E PE=1 SV=1	0,189840015	2,746476
O14974	Protein phosphatase 1 regulatory subunit 12A OS=Homo sapiens OX=9606 GN=PPP1R12A PE=1 SV=1	0,170104297	2,746201
Q9UHD9	Ubiquilin-2 OS=Homo sapiens OX=9606 GN=UBQLN2 PE=1 SV=2	-0,371715364	2,745611
P30443	HLA class I histocompatibility antigen, A-1 alpha chain OS=Homo sapiens OX=9606 GN=HLA-A PE=1 SV=1	-0,315881622	2,741359
Q96A26	Protein FAM162A OS=Homo sapiens OX=9606 GN=FAM162A PE=1 SV=2	-1,704035168	2,738725
Q9H2D6	TRIO and F-actin-binding protein OS=Homo sapiens OX=9606 GN=TRIOBP PE=1 SV=3	0,357754275	2,737477
Q13459	Unconventional myosin-IXb OS=Homo sapiens OX=9606 GN=MYO9B PE=1 SV=3	-0,189181294	2,733499
P11766	Alcohol dehydrogenase class-3 OS=Homo sapiens OX=9606 GN=ADH5 PE=1 SV=4	0,253143318	2,731248
Q9P273	Teneurin-3 OS=Homo sapiens OX=9606 GN=TENM3 PE=2 SV=3	0,180370393	2,730152
Q14974	Importin subunit beta-1 OS=Homo sapiens OX=9606 GN=KPNB1 PE=1 SV=2	-0,137745976	2,729603
Q96RS6	NudC domain-containing protein 1 OS=Homo sapiens OX=9606 GN=NUDCD1 PE=1 SV=2	0,278713587	2,727694
Q9BTW9	Tubulin-specific chaperone D OS=Homo sapiens OX=9606 GN=TBCD PE=1 SV=2	-0,162924967	2,726521
P23743	Diacylglycerol kinase alpha OS=Homo sapiens OX=9606 GN=DGKA PE=1 SV=3	-0,242282893	2,725601
Q9BQ70	Transcription factor 25 OS=Homo sapiens OX=9606 GN=TCF25 PE=1 SV=1	-0,278522642	2,725287
Q8IWB9	Testis-expressed protein 2 OS=Homo sapiens OX=9606 GN=TEX2 PE=1 SV=2	-0,470681603	2,724231
Q9NUP9	Protein lin-7 homolog C OS=Homo sapiens OX=9606 GN=LIN7C PE=1 SV=1	-0,568036782	2,71845
P04632	Calpain small subunit 1 OS=Homo sapiens OX=9606 GN=CAPNS1 PE=1 SV=1	-0,241542713	2,714613
Q8N3P4	Vacuolar protein sorting-associated protein 8 homolog OS=Homo sapiens OX=9606 GN=VPS8 PE=1 SV=3	-0,506414593	2,713385
P25705	ATP synthase subunit alpha, mitochondrial OS=Homo sapiens OX=9606 GN=ATP5F1A PE=1 SV=1	-0,162302195	2,712161
Q9NXR7	BRISC and BRCA1-A complex member 2 OS=Homo sapiens OX=9606 GN=BABAM2 PE=1 SV=2	0,468490507	2,710358
Q9H0J9	Protein mono-ADP-ribosyltransferase PARP12 OS=Homo sapiens OX=9606 GN=PARP12 PE=1 SV=1	0,170023844	2,709318
P62487	DNA-directed RNA polymerase II subunit RPB7 OS=Homo sapiens OX=9606 GN=POLR2G PE=1 SV=1	0,773584678	2,709242
P11498	Pyruvate carboxylase, mitochondrial OS=Homo sapiens OX=9606 GN=PC PE=1 SV=2	0,367497763	2,70832
Q8IWR0	Zinc finger CCCH domain-containing protein 7A OS=Homo sapiens OX=9606 GN=ZC3H7A PE=1 SV=1	-0,565511246	2,706381
Q99439	Calponin-2 OS=Homo sapiens OX=9606 GN=CNN2 PE=1 SV=4	0,263124615	2,704517
Q9BZX2	Uridine-cytidine kinase 2 OS=Homo sapiens OX=9606 GN=UCK2 PE=1 SV=1	-0,770468884	2,700139
Q6WCQ1	Myosin phosphatase Rho-interacting protein OS=Homo sapiens OX=9606 GN=MPRIP PE=1 SV=3	-0,156170557	2,699813
Q9P265	Disco-interacting protein 2 homolog B OS=Homo sapiens OX=9606 GN=DIP2B PE=1 SV=3	0,240404621	2,697741
Q12788	Transducin beta-like protein 3 OS=Homo sapiens OX=9606 GN=TBL3 PE=1 SV=2	0,275969638	2,69315
P39019	40S ribosomal protein S19 OS=Homo sapiens OX=9606 GN=RPS19 PE=1 SV=2	-0,262160193	2,691631
Q9UHI6	Probable ATP-dependent RNA helicase DDX20 OS=Homo sapiens OX=9606 GN=DDX20 PE=1 SV=2	0,335013124	2,690229
Q13573	SNW domain-containing protein 1 OS=Homo sapiens OX=9606 GN=SNW1 PE=1 SV=1	-0,334697254	2,687107
Q8IZL8	Proline-, glutamic acid- and leucine-rich protein 1 OS=Homo sapiens OX=9606 GN=PELP1 PE=1 SV=2	-0,249876493	2,685126
Q9BXS5	AP-1 complex subunit mu-1 OS=Homo sapiens OX=9606 GN=AP1M1 PE=1 SV=3	0,222052623	2,681842
Q92541	RNA polymerase-associated protein RTF1 homolog OS=Homo sapiens OX=9606 GN=RTF1 PE=1 SV=4	-0,291056036	2,68105
Q9H0G5	Nuclear speckle splicing regulatory protein 1 OS=Homo sapiens OX=9606 GN=NSRP1 PE=1 SV=1	-0,763860594	2,680762
Q13505	Metaxin-1 OS=Homo sapiens OX=9606 GN=MTX1 PE=1 SV=3	-0,46377089	2,680449
Q04828	Aldo-keto reductase family 1 member C1 OS=Homo sapiens OX=9606 GN=AKR1C1 PE=1 SV=1	0,404772565	2,678663
P11441	Ubiquitin-like protein 4A OS=Homo sapiens OX=9606 GN=UBL4A PE=1 SV=1	-0,404527376	2,676784
Q9BW72	HIG1 domain family member 2A, mitochondrial OS=Homo sapiens OX=9606 GN=HIGD2A PE=1 SV=1	-0,761302732	2,673235
P42226	Signal transducer and activator of transcription 6 OS=Homo sapiens OX=9606 GN=STAT6 PE=1 SV=1	-0,311689391	2,672545
Q32MZ4	Leucine-rich repeat flightless-interacting protein 1 OS=Homo sapiens OX=9606 GN=LRRFIP1 PE=1 SV=2	0,177555536	2,67208

Q8N201	Integrator complex subunit 1 OS=Homo sapiens OX=9606 GN=INTS1 PE=1 SV=2	-0,332965204	2,669997
Q460N5	Protein mono-ADP-ribosyltransferase PARP14 OS=Homo sapiens OX=9606 GN=PARP14 PE=1 SV=3	-0,461760024	2,667694
Q9UBX3	Mitochondrial dicarboxylate carrier OS=Homo sapiens OX=9606 GN=SLC25A10 PE=1 SV=2	-0,362876881	2,667508
O14656	Torsin-1A OS=Homo sapiens OX=9606 GN=TOR1A PE=1 SV=1	-0,403048387	2,665448
Q96EX3	WD repeat-containing protein 34 OS=Homo sapiens OX=9606 GN=WDR34 PE=1 SV=2	-1,661084162	2,664057
P19474	E3 ubiquitin-protein ligase TRIM21 OS=Homo sapiens OX=9606 GN=TRIM21 PE=1 SV=1	0,461081956	2,663392
O75368	SH3 domain-binding glutamic acid-rich-like protein OS=Homo sapiens OX=9606 GN=SH3BGRL PE=1 SV=1	-0,461027908	2,663049
Q5K651	Sterile alpha motif domain-containing protein 9 OS=Homo sapiens OX=9606 GN=SAMD9 PE=1 SV=1	0,190259598	2,662182
Q9Y2X3	Nucleolar protein 58 OS=Homo sapiens OX=9606 GN=NOP58 PE=1 SV=1	-0,213509798	2,661732
Q6PL24	Protein TMED8 OS=Homo sapiens OX=9606 GN=TMED8 PE=1 SV=1	-0,30831721	2,651634
P54132	#N/D	0,217379931	2,650123
Q9Y394	Dehydrogenase/reductase SDR family member 7 OS=Homo sapiens OX=9606 GN=DHRS7 PE=1 SV=1	0,458379312	2,646236
Q92522	Histone H1x OS=Homo sapiens OX=9606 GN=H1FX PE=1 SV=1	-0,329838064	2,639145
Q16540	39S ribosomal protein L23, mitochondrial OS=Homo sapiens OX=9606 GN=MRPL23 PE=1 SV=1	-0,550608294	2,634821
P49917	DNA ligase 4 OS=Homo sapiens OX=9606 GN=LIG4 PE=1 SV=2	-0,747756124	2,633129
Q86YP4	Transcriptional repressor p66-alpha OS=Homo sapiens OX=9606 GN=GATAD2A PE=1 SV=1	-0,286917454	2,632606
P61313	60S ribosomal protein L15 OS=Homo sapiens OX=9606 GN=RPL15 PE=1 SV=2	-0,286908208	2,632498
Q68CQ7	Glycosyltransferase 8 domain-containing protein 1 OS=Homo sapiens OX=9606 GN=GLT8D1 PE=1 SV=2	-0,52391875	2,627244
P15924	Desmoplakin OS=Homo sapiens OX=9606 GN=DSP PE=1 SV=3	-0,153030443	2,625225
Q09028	Histone-binding protein RBBP4 OS=Homo sapiens OX=9606 GN=RBBP4 PE=1 SV=3	0,304997528	2,623379
Q14693	Phosphatidate phosphatase LPIN1 OS=Homo sapiens OX=9606 GN=LPIN1 PE=1 SV=2	-0,744399499	2,623128
Q8WXH0	Nesprin-2 OS=Homo sapiens OX=9606 GN=SYNE2 PE=1 SV=3	-0,743927499	2,621719
Q9UNF1	Melanoma-associated antigen D2 OS=Homo sapiens OX=9606 GN=MAGED2 PE=1 SV=2	0,158247382	2,619357
P29317	Ephrin type-A receptor 2 OS=Homo sapiens OX=9606 GN=EPHA2 PE=1 SV=2	0,187959114	2,618652
Q9NP81	Serine--tRNA ligase, mitochondrial OS=Homo sapiens OX=9606 GN=SARS2 PE=1 SV=1	-0,304559614	2,618651
P16930	Fumarylacetoacetase OS=Homo sapiens OX=9606 GN=FAH PE=1 SV=2	-0,357323807	2,618532
Q14789	Golgin subfamily B member 1 OS=Homo sapiens OX=9606 GN=GOLGB1 PE=1 SV=2	0,120250035	2,615283
Q96T76	MMS19 nucleotide excision repair protein homolog OS=Homo sapiens OX=9606 GN=MMS19 PE=1 SV=2	0,174764787	2,614946
Q9H6R4	Nucleolar protein 6 OS=Homo sapiens OX=9606 GN=NOL6 PE=1 SV=2	-0,256360506	2,614471
Q9Y6M7	Sodium bicarbonate cotransporter 3 OS=Homo sapiens OX=9606 GN=SLC4A7 PE=1 SV=2	0,545694247	2,611098
Q9H0E2	Toll-interacting protein OS=Homo sapiens OX=9606 GN=TOLLIP PE=1 SV=1	0,326847697	2,609688
Q8IX90	Spindle and kinetochore-associated protein 3 OS=Homo sapiens OX=9606 GN=SKA3 PE=1 SV=2	-0,452295637	2,607575
Q86UQ4	ATP-binding cassette sub-family A member 13 OS=Homo sapiens OX=9606 GN=ABCA13 PE=2 SV=3	-0,490919566	2,606218
Q8TDZ2	[F-actin]-monooxygenase MICAL1 OS=Homo sapiens OX=9606 GN=MICAL1 PE=1 SV=2	-0,217246229	2,604763
Q6P2E9	Enhancer of mRNA-decapping protein 4 OS=Homo sapiens OX=9606 GN=EDC4 PE=1 SV=1	0,20964343	2,597444
P18858	DNA ligase 1 OS=Homo sapiens OX=9606 GN=LIG1 PE=1 SV=1	-0,558575494	2,597356
Q9GZL7	Ribosome biogenesis protein WDR12 OS=Homo sapiens OX=9606 GN=WDR12 PE=1 SV=2	-0,302510771	2,596554
O15050	TPR and ankyrin repeat-containing protein 1 OS=Homo sapiens OX=9606 GN=TRANK1 PE=2 SV=4	-0,924068442	2,592256
P57678	Gem-associated protein 4 OS=Homo sapiens OX=9606 GN=GEMIN4 PE=1 SV=2	-0,325057366	2,592075
Q53TN4	Cytochrome b reductase 1 OS=Homo sapiens OX=9606 GN=CYBRD1 PE=1 SV=1	0,73374616	2,591215
P42166	Lamina-associated polypeptide 2, isoform alpha OS=Homo sapiens OX=9606 GN=TMPO PE=1 SV=2	-0,216380092	2,590946
Q96HC4	PDZ and LIM domain protein 5 OS=Homo sapiens OX=9606 GN=PDLIM5 PE=1 SV=5	0,30192493	2,590241
P14625	Endoplasmin OS=Homo sapiens OX=9606 GN=HSP90B1 PE=1 SV=1	0,124758175	2,58926
Q9BSJ2	Gamma-tubulin complex component 2 OS=Homo sapiens OX=9606 GN=TUBGCP2 PE=1 SV=2	-0,301590757	2,586642
Q92968	Peroxisomal membrane protein PEX13 OS=Homo sapiens OX=9606 GN=PEX13 PE=1 SV=2	-0,773826349	2,584328
P43246	DNA mismatch repair protein Msh2 OS=Homo sapiens OX=9606 GN=MSH2 PE=1 SV=1	-0,146337155	2,582546
P43304	Glycerol-3-phosphate dehydrogenase, mitochondrial OS=Homo sapiens OX=9606 GN=GPD2 PE=1 SV=3	-0,162620642	2,581791
Q96MG7	Non-structural maintenance of chromosomes element 3 homolog OS=Homo sapiens OX=9606 GN=NSMCE3 PE=1 SV=1	-0,759995654	2,579362
P46937	Transcriptional coactivator YAP1 OS=Homo sapiens OX=9606 GN=YAP1 PE=1 SV=2	0,300523609	2,575154
Q9Y697	Cysteine desulfurase, mitochondrial OS=Homo sapiens OX=9606 GN=NFS1 PE=1 SV=3	0,727298515	2,571775
P09874	Poly [ADP-ribose] polymerase 1 OS=Homo sapiens OX=9606 GN=PARP1 PE=1 SV=4	-0,116360918	2,571014
P49643	DNA primase large subunit OS=Homo sapiens OX=9606 GN=PRIM2 PE=1 SV=2	-0,300127879	2,570896
Q96EP5	DAZ-associated protein 1 OS=Homo sapiens OX=9606 GN=DAZAP1 PE=1 SV=1	-0,300038282	2,569932
O96008	Mitochondrial import receptor subunit TOM40 homolog OS=Homo sapiens OX=9606 GN=TOMM40 PE=1 SV=1	-0,351425675	2,566602
Q9NZM5	Ribosome biogenesis protein NOP53 OS=Homo sapiens OX=9606 GN=NOP53 PE=1 SV=2	-0,536504286	2,566569
P98179	RNA-binding protein 3 OS=Homo sapiens OX=9606 GN=RBM3 PE=1 SV=1	-0,389509122	2,561707
Q8NBJ5	Procollagen galactosyltransferase 1 OS=Homo sapiens OX=9606 GN=COLGALT1 PE=1 SV=1	0,161411697	2,555581
P62829	60S ribosomal protein L23 OS=Homo sapiens OX=9606 GN=RPL23 PE=1 SV=1	-0,298531969	2,553738
P11940	Polyadenylate-binding protein 1 OS=Homo sapiens OX=9606 GN=PABPC1 PE=1 SV=2	-0,194675484	2,551891
O60825	6-phosphofructo-2-kinase/fructose-2,6-bisphosphatase 2 OS=Homo sapiens OX=9606 GN=PFKFB2 PE=1 SV=2	0,839131029	2,551874
Q8NFV4	Protein ABHD11 OS=Homo sapiens OX=9606 GN=ABHD11 PE=1 SV=1	-0,443397839	2,550934
Q92845	Kinesin-associated protein 3 OS=Homo sapiens OX=9606 GN=KIFAP3 PE=1 SV=2	0,387748782	2,548225
P47755	F-actin-capping protein subunit alpha-2 OS=Homo sapiens OX=9606 GN=CAPZA2 PE=1 SV=3	0,264162097	2,545682
Q96JM3	Chromosome alignment-maintaining phosphoprotein 1 OS=Homo sapiens OX=9606 GN=CHAMP1 PE=1 SV=2	-0,297608564	2,543821
P15151	Poliovirus receptor OS=Homo sapiens OX=9606 GN=PVR PE=1 SV=2	0,717550322	2,542202
O14618	Copper chaperone for superoxide dismutase OS=Homo sapiens OX=9606 GN=CCS PE=1 SV=1	0,44201512	2,542122
P29401	Transketolase OS=Homo sapiens OX=9606 GN=TKT PE=1 SV=3	0,136245371	2,54126
Q8IVG5	Sterile alpha motif domain-containing protein 9-like OS=Homo sapiens OX=9606 GN=SAMD9L PE=1 SV=2	-0,348447307	2,540416
O75962	Triple functional domain protein OS=Homo sapiens OX=9606 GN=TRIO PE=1 SV=2	-0,129129406	2,540259
Q562R1	Beta-actin-like protein 2 OS=Homo sapiens OX=9606 GN=ACTBL2 PE=1 SV=2	0,53064784	2,538082
Q13444	Disintegrin and metalloproteinase domain-containing protein 15 OS=Homo sapiens OX=9606 GN=ADAM15 PE=1 SV=4	-0,71492628	2,534204
P10599	Thioredoxin OS=Homo sapiens OX=9606 GN=TXN PE=1 SV=3	-0,440498998	2,532457
P52434	DNA-directed RNA polymerases I, II, and III subunit RPABC3 OS=Homo sapiens OX=9606 GN=POLR2H PE=1 SV=4	0,385544552	2,531345
Q02809	Procollagen-lysine,2-oxoglutarate 5-dioxygenase 1 OS=Homo sapiens OX=9606 GN=PLOD1 PE=1 SV=2	0,174478952	2,529625
Q9P0P0	E3 ubiquitin-protein ligase RNF181 OS=Homo sapiens OX=9606 GN=RNF181 PE=1 SV=1	-0,439806981	2,528045
Q92783	Signal transducing adapter molecule 1 OS=Homo sapiens OX=9606 GN=STAM PE=1 SV=3	0,262715135	2,527747
Q8IWZ8	SURP and G-patch domain-containing protein 1 OS=Homo sapiens OX=9606 GN=SUGP1 PE=1 SV=2	-0,384288234	2,521726
P09601	Heme oxygenase 1 OS=Homo sapiens OX=9606 GN=HMOX1 PE=1 SV=1	0,527215794	2,521348
P49427	Ubiquitin-conjugating enzyme E2 R1 OS=Homo sapiens OX=9606 GN=CDC34 PE=1 SV=2	-0,438652424	2,520682
Q9NXG6	Transmembrane prolyl 4-hydroxylase OS=Homo sapiens OX=9606 GN=P4HTM PE=1 SV=2	0,82769391	2,51921
O75348	V-type proton ATPase subunit G 1 OS=Homo sapiens OX=9606 GN=ATP6V1G1 PE=1 SV=3	-0,526378601	2,517261
Q13637	Ras-related protein Rab-32 OS=Homo sapiens OX=9606 GN=RAB32 PE=1 SV=3	0,294765113	2,513326
Q9UNW1	Multiple inositol polyphosphate phosphatase 1 OS=Homo sapiens OX=9606 GN=MINPP1 PE=1 SV=1	0,525396944	2,512468
O00165	HCLS1-associated protein X-1 OS=Homo sapiens OX=9606 GN=HAX1 PE=1 SV=2	-0,382804018	2,510363
P62280	40S ribosomal protein S11 OS=Homo sapiens OX=9606 GN=RPS11 PE=1 SV=3	-0,218977028	2,509937
P43121	Cell surface glycoprotein MUC18 OS=Homo sapiens OX=9606 GN=MCAM PE=1 SV=2	0,107987252	2,509413
Q9UJU6	Drebrin-like protein OS=Homo sapiens OX=9606 GN=DBNL PE=1 SV=1	-0,186471594	2,50011
O00470	Homeobox protein Meis1 OS=Homo sapiens OX=9606 GN=MEIS1 PE=1 SV=1	-0,605949704	2,499608
P17707	S-adenosylmethionine decarboxylase proenzyme OS=Homo sapiens OX=9606 GN=AMD1 PE=1 SV=2	1,473472737	2,499
Q9UDY2	Tight junction protein ZO-2 OS=Homo sapiens OX=9606 GN=TJP2 PE=1 SV=2	0,147576939	2,497567
Q9UHQ4	B-cell receptor-associated protein 29 OS=Homo sapiens OX=9606 GN=BCAP29 PE=1 SV=2	-0,381130578	2,497554

Q12765	Secernin-1 OS=Homo sapiens OX=9606 GN=SCRN1 PE=1 SV=2	0,210188062	2,492833
Q9NY61	Protein AATF OS=Homo sapiens OX=9606 GN=AATF PE=1 SV=1	-0,69970724	2,487503
O43847	Nardilysin OS=Homo sapiens OX=9606 GN=NRDC PE=1 SV=3	-0,185700003	2,4862
Q92544	Transmembrane 9 superfamily member 4 OS=Homo sapiens OX=9606 GN=TM9SF4 PE=1 SV=2	0,274302591	2,486115
P54725	UV excision repair protein RAD23 homolog A OS=Homo sapiens OX=9606 GN=RAD23A PE=1 SV=1	-0,259341421	2,486048
Q9UDY4	DnaJ homolog subfamily B member 4 OS=Homo sapiens OX=9606 GN=DNAJB4 PE=1 SV=1	0,433189775	2,485823
A4UGR9	Xin actin-binding repeat-containing protein 2 OS=Homo sapiens OX=9606 GN=XIRP2 PE=1 SV=2	-0,581346016	2,485025
Q02818	Nucleobindin-1 OS=Homo sapiens OX=9606 GN=NUCB1 PE=1 SV=4	-0,340890856	2,474102
Q9BX40	Protein LSM14 homolog B OS=Homo sapiens OX=9606 GN=LSM14B PE=1 SV=1	0,517522074	2,473928
O14662	Syntaxin-16 OS=Homo sapiens OX=9606 GN=STX16 PE=1 SV=3	-0,517465643	2,473652
Q16527	Cysteine and glycine-rich protein 2 OS=Homo sapiens OX=9606 GN=CSRP2 PE=1 SV=3	0,517327486	2,472974
Q9GZU8	PSME3-interacting protein OS=Homo sapiens OX=9606 GN=FAM192A PE=1 SV=1	-0,430436965	2,468243
P21926	CD9 antigen OS=Homo sapiens OX=9606 GN=CD9 PE=1 SV=4	0,430163239	2,466495
Q9H479	Fructosamine-3-kinase OS=Homo sapiens OX=9606 GN=FN3K PE=1 SV=1	0,51558726	2,464437
Q13405	39S ribosomal protein L49, mitochondrial OS=Homo sapiens OX=9606 GN=MRPL49 PE=1 SV=1	-0,515192939	2,462501
Q8N8S7	Protein enabled homolog OS=Homo sapiens OX=9606 GN=ENAH PE=1 SV=2	0,179400703	2,458881
P48729	Casein kinase I isoform alpha OS=Homo sapiens OX=9606 GN=CSNK1A1 PE=1 SV=2	-0,28962849	2,458418
P67809	Nuclease-sensitive element-binding protein 1 OS=Homo sapiens OX=9606 GN=YBX1 PE=1 SV=3	-0,271694899	2,456062
Q99613	Eukaryotic translation initiation factor 3 subunit C OS=Homo sapiens OX=9606 GN=EIF3C PE=1 SV=1	-0,153776499	2,455599
Q14123	Calcium/calmodulin-dependent 3',5'-cyclic nucleotide phosphodiesterase 1C OS=Homo sapiens OX=9606 GN=PDE1C PE=1	0,145735878	2,455013
P14209	CD99 antigen OS=Homo sapiens OX=9606 GN=CD99 PE=1 SV=1	-0,513239405	2,452907
Q05682	Caldesmon OS=Homo sapiens OX=9606 GN=CALD1 PE=1 SV=3	0,174478282	2,449449
Q9Y2W1	Thyroid hormone receptor-associated protein 3 OS=Homo sapiens OX=9606 GN=THRAP3 PE=1 SV=2	-0,188526057	2,444295
Q9BY32	Inosine triphosphate pyrophosphatase OS=Homo sapiens OX=9606 GN=ITPA PE=1 SV=2	-0,309931438	2,443987
P13726	Tissue factor OS=Homo sapiens OX=9606 GN=F3 PE=1 SV=1	0,511105776	2,442418
Q6NUQ4	Transmembrane protein 214 OS=Homo sapiens OX=9606 GN=TMEM214 PE=1 SV=2	0,214466469	2,44184
Q8WVM7	Cohesin subunit SA-1 OS=Homo sapiens OX=9606 GN=STAG1 PE=1 SV=3	-0,222351229	2,434177
Q5EBL4	RILP-like protein 1 OS=Homo sapiens OX=9606 GN=RILPL1 PE=1 SV=1	-0,799208641	2,431999
P06733	Alpha-enolase OS=Homo sapiens OX=9606 GN=ENO1 PE=1 SV=2	-0,169452289	2,43146
Q9UNF0	Protein kinase C and casein kinase substrate in neurons protein 2 OS=Homo sapiens OX=9606 GN=PACSIN2 PE=1 SV=2	0,268757737	2,42231
P49748	Very long-chain specific acyl-CoA dehydrogenase, mitochondrial OS=Homo sapiens OX=9606 GN=ACADVL PE=1 SV=1	-0,139644165	2,421769
Q9UJX6	Anaphase-promoting complex subunit 2 OS=Homo sapiens OX=9606 GN=ANAPC2 PE=1 SV=1	1,12769009	2,420202
P53007	Tricarboxylate transport protein, mitochondrial OS=Homo sapiens OX=9606 GN=SLC25A1 PE=1 SV=2	-0,307367979	2,419025
Q9NR30	Nucleolar RNA helicase 2 OS=Homo sapiens OX=9606 GN=DDX21 PE=1 SV=5	-0,149074195	2,413879
O00743	Serine/threonine-protein phosphatase 6 catalytic subunit OS=Homo sapiens OX=9606 GN=PPP6C PE=1 SV=1	-0,240690109	2,408919
Q9UDR5	Alpha-aminoadipic semialdehyde synthase, mitochondrial OS=Homo sapiens OX=9606 GN=AASS PE=1 SV=1	0,333135899	2,406245
Q96RN5	Mediator of RNA polymerase II transcription subunit 15 OS=Homo sapiens OX=9606 GN=MED15 PE=1 SV=2	0,673408106	2,405526
O75886	Signal transducing adapter molecule 2 OS=Homo sapiens OX=9606 GN=STAM2 PE=1 SV=1	0,332867779	2,403902
Q14739	Delta(14)-sterol reductase OS=Homo sapiens OX=9606 GN=LBR PE=1 SV=2	-0,305716534	2,402966
P28066	Proteasome subunit alpha type-5 OS=Homo sapiens OX=9606 GN=PSMA5 PE=1 SV=3	-0,252571541	2,402887
Q9UPV0	Centrosomal protein of 164 kDa OS=Homo sapiens OX=9606 GN=CEP164 PE=1 SV=3	0,889528261	2,399847
Q9UMY4	Sorting nexin-12 OS=Homo sapiens OX=9606 GN=SNX12 PE=1 SV=3	-0,332349974	2,39938
Q8WWN8	Arf-GAP with Rho-GAP domain, ANK repeat and PH domain-containing protein 3 OS=Homo sapiens OX=9606 GN=ARAP3 F	-0,75161237	2,396819
Q6NUK1	Calcium-binding mitochondrial carrier protein SCaMC-1 OS=Homo sapiens OX=9606 GN=SLC25A24 PE=1 SV=2	-0,180640088	2,395635
Q96T37	RNA-binding protein 15 OS=Homo sapiens OX=9606 GN=RBM15 PE=1 SV=2	0,331736303	2,394021
Q9H3Q1	Cdc42 effector protein 4 OS=Homo sapiens OX=9606 GN=CDC42EP4 PE=1 SV=1	-1,168061018	2,393582
Q9NW08	DNA-directed RNA polymerase III subunit RPC2 OS=Homo sapiens OX=9606 GN=POLR3B PE=1 SV=2	0,723890027	2,393527
P24752	Acetyl-CoA acetyltransferase, mitochondrial OS=Homo sapiens OX=9606 GN=ACAT1 PE=1 SV=1	-0,180438429	2,392049
O95299	NADH dehydrogenase [ubiquinone] 1 alpha subcomplex subunit 10, mitochondrial OS=Homo sapiens OX=9606 GN=NDUF	-0,265826981	2,388737
P08243	Asparagine synthetase [glutamine-hydrolyzing] OS=Homo sapiens OX=9606 GN=ASNS PE=1 SV=4	0,251358331	2,388058
P35658	Nuclear pore complex protein Nup214 OS=Homo sapiens OX=9606 GN=NUP214 PE=1 SV=2	0,180204878	2,387898
Q7L804	Rab11 family-interacting protein 2 OS=Homo sapiens OX=9606 GN=RAB11FIP2 PE=1 SV=1	-1,344366819	2,387302
P15559	NAD(P)H dehydrogenase [quinone] 1 OS=Homo sapiens OX=9606 GN=NQO1 PE=1 SV=1	0,238936267	2,38619
Q14498	RNA-binding protein 39 OS=Homo sapiens OX=9606 GN=RBM39 PE=1 SV=2	0,185158097	2,386024
O95816	BAG family molecular chaperone regulator 2 OS=Homo sapiens OX=9606 GN=BAG2 PE=1 SV=1	0,251181196	2,385895
Q9BRF8	Serine/threonine-protein phosphatase CPPED1 OS=Homo sapiens OX=9606 GN=CPPED1 PE=1 SV=3	0,22819452	2,384529
Q9UJX3	Anaphase-promoting complex subunit 7 OS=Homo sapiens OX=9606 GN=ANAPC7 PE=1 SV=4	-0,228000546	2,381885
P04062	Lysosomal acid glucosylceramidase OS=Homo sapiens OX=9606 GN=GBA PE=1 SV=3	-0,227977631	2,381573
Q8WXF1	Paraspeckle component 1 OS=Homo sapiens OX=9606 GN=PSPC1 PE=1 SV=1	-0,190307446	2,379974
Q8N806	Putative E3 ubiquitin-protein ligase UBR7 OS=Homo sapiens OX=9606 GN=UBR7 PE=1 SV=2	-0,416236243	2,377432
Q8IY37	Probable ATP-dependent RNA helicase DHX37 OS=Homo sapiens OX=9606 GN=DHX37 PE=1 SV=1	0,664084942	2,376074
Q96PZ2	Protein FAM111A OS=Homo sapiens OX=9606 GN=FAM111A PE=1 SV=2	-0,497245912	2,374026
Q12789	General transcription factor 3C polypeptide 1 OS=Homo sapiens OX=9606 GN=GTF3C1 PE=1 SV=4	-0,1627131	2,373313
P82663	28S ribosomal protein S25, mitochondrial OS=Homo sapiens OX=9606 GN=MRPS25 PE=1 SV=1	-0,663016565	2,372686
Q8N5F7	NF-kappa-B-activating protein OS=Homo sapiens OX=9606 GN=NKAP PE=1 SV=1	0,719288885	2,370865
Q1KMD3	Heterogeneous nuclear ribonucleoprotein U-like protein 2 OS=Homo sapiens OX=9606 GN=HNRNPUL2 PE=1 SV=1	-0,162543765	2,369977
Q13557	Calcium/calmodulin-dependent protein kinase type II subunit delta OS=Homo sapiens OX=9606 GN=CAMK2D PE=1 SV=3	0,302301842	2,369817
Q9NRR8	CDC42 small effector protein 1 OS=Homo sapiens OX=9606 GN=CDC42SE1 PE=1 SV=1	1,332978389	2,362488
O43809	Cleavage and polyadenylation specificity factor subunit 5 OS=Homo sapiens OX=9606 GN=NUDT21 PE=1 SV=1	-0,236991272	2,361052
Q5T8P6	RNA-binding protein 26 OS=Homo sapiens OX=9606 GN=RBM26 PE=1 SV=3	0,413607152	2,3606
O43264	Centromere/kinetochore protein zw10 homolog OS=Homo sapiens OX=9606 GN=ZW10 PE=1 SV=3	-0,217159949	2,359857
Q9UBT2	SUMO-activating enzyme subunit 2 OS=Homo sapiens OX=9606 GN=UBA2 PE=1 SV=2	-0,16971146	2,359706
Q92901	60S ribosomal protein L3-like OS=Homo sapiens OX=9606 GN=RPL3L PE=2 SV=3	1,408662318	2,358711
Q9BTE7	DCN1-like protein 5 OS=Homo sapiens OX=9606 GN=DCUN1D5 PE=1 SV=1	-0,465762637	2,356995
P11717	Cation-independent mannose-6-phosphate receptor OS=Homo sapiens OX=9606 GN=IGF2R PE=1 SV=3	0,094268317	2,35536
O75367	Core histone macro-H2A.1 OS=Homo sapiens OX=9606 GN=H2AFY PE=1 SV=4	0,208639775	2,354715
Q4G0N4	NAD kinase 2, mitochondrial OS=Homo sapiens OX=9606 GN=NADK2 PE=1 SV=2	0,787610999	2,353117
Q9UPQ0	LIM and calponin homology domains-containing protein 1 OS=Homo sapiens OX=9606 GN=LIMCH1 PE=1 SV=4	0,412345022	2,352517
Q4KMP7	TBC1 domain family member 10B OS=Homo sapiens OX=9606 GN=TBC1D10B PE=1 SV=3	-0,326965361	2,352408
Q8TEA8	D-aminoacyl-tRNA deacylase 1 OS=Homo sapiens OX=9606 GN=DTD1 PE=1 SV=2	-0,412316532	2,352335
Q9P2N5	RNA-binding protein 27 OS=Homo sapiens OX=9606 GN=RBM27 PE=1 SV=2	-0,262511989	2,350891
P41236	Protein phosphatase inhibitor 2 OS=Homo sapiens OX=9606 GN=PPP1R2 PE=1 SV=2	-0,492576535	2,350887
Q8ND04	Protein SMG8 OS=Homo sapiens OX=9606 GN=SMG8 PE=1 SV=1	-0,492449415	2,350256
P22059	Oxysterol-binding protein 1 OS=Homo sapiens OX=9606 GN=OSBP PE=1 SV=1	0,225661985	2,350071
Q99816	Tumor susceptibility gene 101 protein OS=Homo sapiens OX=9606 GN=TSG101 PE=1 SV=2	-0,236134878	2,350006
Q9H900	Protein zwilch homolog OS=Homo sapiens OX=9606 GN=ZWILCH PE=1 SV=2	-0,411936584	2,349901
Q96EA4	Protein Spindly OS=Homo sapiens OX=9606 GN=SPDL1 PE=1 SV=2	-0,165192189	2,349343
O96000	NADH dehydrogenase [ubiquinone] 1 beta subcomplex subunit 10 OS=Homo sapiens OX=9606 GN=NDUFB10 PE=1 SV=3	-0,326409438	2,347565
P10586	Receptor-type tyrosine-protein phosphatase F OS=Homo sapiens OX=9606 GN=PTPRF PE=1 SV=2	0,411370412	2,346275
P49411	Elongation factor Tu, mitochondrial OS=Homo sapiens OX=9606 GN=TUFM PE=1 SV=2	-0,157766977	2,34397
Q15262	Receptor-type tyrosine-protein phosphatase kappa OS=Homo sapiens OX=9606 GN=PTPRK PE=1 SV=2	0,561794855	2,34338

P60604	Ubiquitin-conjugating enzyme E2 G2 OS=Homo sapiens OX=9606 GN=UBE2G2 PE=1 SV=1	-0,490993739	2,343032
Q8NFH4	Nucleoporin Nup37 OS=Homo sapiens OX=9606 GN=NUP37 PE=1 SV=1	-0,299470929	2,342394
P20742	Pregnancy zone protein OS=Homo sapiens OX=9606 GN=PZP PE=1 SV=4	0,473400885	2,339815
P67870	Casein kinase II subunit beta OS=Homo sapiens OX=9606 GN=CSNK2B PE=1 SV=1	-0,299145207	2,339242
Q9HBD1	Roquin-2 OS=Homo sapiens OX=9606 GN=RC3H2 PE=1 SV=2	0,850914102	2,33666
Q9Y248	DNA replication complex GINS protein PSF2 OS=Homo sapiens OX=9606 GN=GINS2 PE=1 SV=1	-0,409583732	2,33483
Q9NXR5	Ankyrin repeat domain-containing protein 10 OS=Homo sapiens OX=9606 GN=ANKRD10 PE=1 SV=2	1,305732268	2,334548
Q96FV2	Secernin-2 OS=Homo sapiens OX=9606 GN=SCRN2 PE=1 SV=3	-0,324734886	2,332984
B1ANS9	WD repeat-containing protein 64 OS=Homo sapiens OX=9606 GN=WDR64 PE=2 SV=1	0,209731012	2,330509
O43665	Regulator of G-protein signaling 10 OS=Homo sapiens OX=9606 GN=RGS10 PE=1 SV=3	-0,359084076	2,329064
P49454	Centromere protein F OS=Homo sapiens OX=9606 GN=CENPF PE=1 SV=3	0,359054862	2,328841
Q15370	Elongin-B OS=Homo sapiens OX=9606 GN=ELOB PE=1 SV=1	-0,324161016	2,327989
Q05209	Tyrosine-protein phosphatase non-receptor type 12 OS=Homo sapiens OX=9606 GN=PTPN12 PE=1 SV=3	0,223918688	2,326434
Q9UGV2	Protein NDRG3 OS=Homo sapiens OX=9606 GN=NDRG3 PE=1 SV=2	0,323943296	2,326095
Q9UBU8	Mortality factor 4-like protein 1 OS=Homo sapiens OX=9606 GN=MORF4L1 PE=1 SV=2	-0,323942718	2,32609
P27361	Mitogen-activated protein kinase 3 OS=Homo sapiens OX=9606 GN=MAPK3 PE=1 SV=4	0,223819024	2,325085
Q10713	Mitochondrial-processing peptidase subunit alpha OS=Homo sapiens OX=9606 GN=PMPCA PE=1 SV=2	-0,223718408	2,323723
Q9NUD5	Zinc finger CCHC domain-containing protein 3 OS=Homo sapiens OX=9606 GN=ZCCHC3 PE=1 SV=2	-0,687586326	2,32181
Q5VU36	Spermatogenesis-associated protein 31A5 OS=Homo sapiens OX=9606 GN=SPATA31A5 PE=3 SV=1	0,645934743	2,318146
O75717	WD repeat and HMG-box DNA-binding protein 1 OS=Homo sapiens OX=9606 GN=WDHD1 PE=1 SV=1	-0,322836294	2,316466
Q06033	Inter-alpha-trypsin inhibitor heavy chain H3 OS=Homo sapiens OX=9606 GN=ITIH3 PE=1 SV=2	-0,48554591	2,315956
Q9H3N1	Thioredoxin-related transmembrane protein 1 OS=Homo sapiens OX=9606 GN=TMX1 PE=1 SV=1	-0,485218703	2,314328
O95336	6-phosphogluconolactonase OS=Homo sapiens OX=9606 GN=PGLS PE=1 SV=2	0,223017245	2,314238
Q96GG9	DCN1-like protein 1 OS=Homo sapiens OX=9606 GN=DCUN1D1 PE=1 SV=1	0,322274583	2,311582
P48163	NADP-dependent malic enzyme OS=Homo sapiens OX=9606 GN=ME1 PE=1 SV=1	0,175743207	2,309079
Q9BXM0	Periaxin OS=Homo sapiens OX=9606 GN=PRX PE=1 SV=2	-1,288705589	2,308216
Q96DB5	Regulator of microtubule dynamics protein 1 OS=Homo sapiens OX=9606 GN=RMDN1 PE=1 SV=1	-0,295531635	2,304324
P11908	Ribose-phosphate pyrophosphokinase 2 OS=Homo sapiens OX=9606 GN=PRPS2 PE=1 SV=2	-0,213212986	2,303806
Q9ULX6	A-kinase anchor protein 8-like OS=Homo sapiens OX=9606 GN=AKAP8L PE=1 SV=4	0,295433319	2,303375
O14562	Ubiquitin domain-containing protein UBFD1 OS=Homo sapiens OX=9606 GN=UBFD1 PE=1 SV=2	0,355598864	2,302487
Q7Z2K8	G protein-regulated inducer of neurite outgrowth 1 OS=Homo sapiens OX=9606 GN=GPRIN1 PE=1 SV=2	-0,482765956	2,302115
O75608	Acyl-protein thioesterase 1 OS=Homo sapiens OX=9606 GN=LYPLA1 PE=1 SV=1	-0,321159008	2,301886
P09884	DNA polymerase alpha catalytic subunit OS=Homo sapiens OX=9606 GN=POLA1 PE=1 SV=2	-0,212925219	2,299735
Q96RE7	Nucleus accumbens-associated protein 1 OS=Homo sapiens OX=9606 GN=NACC1 PE=1 SV=1	-0,274430056	2,297378
Q9P0S9	Transmembrane protein 14C OS=Homo sapiens OX=9606 GN=TMEM14C PE=1 SV=1	-0,481503722	2,295826
O75051	Plexin-A2 OS=Homo sapiens OX=9606 GN=PLXNA2 PE=1 SV=4	0,403411398	2,295276
Q8NBM8	Prenylcysteine oxidase-like OS=Homo sapiens OX=9606 GN=PCYOX1L PE=1 SV=2	-0,638652375	2,294683
P84101	Small EDRK-rich factor 2 OS=Homo sapiens OX=9606 GN=SERF2 PE=1 SV=1	-1,955256071	2,292356
Q9Y314	Nitric oxide synthase-interacting protein OS=Homo sapiens OX=9606 GN=NOSIP PE=1 SV=1	0,221177536	2,289404
Q92600	CCR4-NOT transcription complex subunit 9 OS=Homo sapiens OX=9606 GN=CNOT9 PE=1 SV=1	0,35372907	2,288236
Q13418	Integrin-linked protein kinase OS=Homo sapiens OX=9606 GN=ILK PE=1 SV=2	-0,174543596	2,288041
O15027	Protein transport protein Sec16A OS=Homo sapiens OX=9606 GN=SEC16A PE=1 SV=4	0,122770673	2,287033
Q92466	DNA damage-binding protein 2 OS=Homo sapiens OX=9606 GN=DDB2 PE=1 SV=1	-0,231213823	2,286804
Q02952	A-kinase anchor protein 12 OS=Homo sapiens OX=9606 GN=AKAP12 PE=1 SV=4	-0,102785507	2,285059
Q53HC9	EARP and GARP complex-interacting protein 1 OS=Homo sapiens OX=9606 GN=EIPR1 PE=1 SV=2	-0,293393161	2,283703
Q15428	Splicing factor 3A subunit 2 OS=Homo sapiens OX=9606 GN=SF3A2 PE=1 SV=2	-0,230917312	2,28301
Q9NVM6	DnaJ homolog subfamily C member 17 OS=Homo sapiens OX=9606 GN=DNAJC17 PE=1 SV=1	-0,63473166	2,281999
Q9BXP5	Serrate RNA effector molecule homolog OS=Homo sapiens OX=9606 GN=SRRT PE=1 SV=1	-0,174093552	2,280165
Q9BVC5	Ashwin OS=Homo sapiens OX=9606 GN=C2orf49 PE=1 SV=1	-1,253973124	2,280036
Q5TAQ9	DDB1- and CUL4-associated factor 8 OS=Homo sapiens OX=9606 GN=DCAF8 PE=1 SV=1	-0,478073809	2,278717
Q9UNN5	FAS-associated factor 1 OS=Homo sapiens OX=9606 GN=FAF1 PE=1 SV=2	-0,256038451	2,277391
Q96R06	Sperm-associated antigen 5 OS=Homo sapiens OX=9606 GN=SPAG5 PE=1 SV=2	-0,255832028	2,275056
O94966	Ubiquitin carboxyl-terminal hydrolase 19 OS=Homo sapiens OX=9606 GN=USP19 PE=1 SV=2	-0,477239783	2,274553
Q9P2J8	Zinc finger protein 624 OS=Homo sapiens OX=9606 GN=ZNF624 PE=1 SV=3	1,03230344	2,273127
Q15050	Ribosome biogenesis regulatory protein homolog OS=Homo sapiens OX=9606 GN=RRS1 PE=1 SV=2	-0,399365484	2,269337
P46734	Dual specificity mitogen-activated protein kinase kinase 3 OS=Homo sapiens OX=9606 GN=MAP2K3 PE=1 SV=2	0,219435609	2,265961
Q12931	Heat shock protein 75 kDa, mitochondrial OS=Homo sapiens OX=9606 GN=TRAP1 PE=1 SV=3	-0,173083968	2,262531
Q8WZA9	Immunity-related GTPase family Q protein OS=Homo sapiens OX=9606 GN=IRGQ PE=1 SV=1	0,241010063	2,262517
P23368	NAD-dependent malic enzyme, mitochondrial OS=Homo sapiens OX=9606 GN=ME2 PE=1 SV=1	-0,209936329	2,257578
Q6ZNJ1	Neurobeachin-like protein 2 OS=Homo sapiens OX=9606 GN=NBEAL2 PE=1 SV=2	0,189452671	2,257392
P48378	DNA-binding protein RFX2 OS=Homo sapiens OX=9606 GN=RFX2 PE=1 SV=2	1,999442345	2,255753
Q7LG56	Ribonucleoside-diphosphate reductase subunit M2 B OS=Homo sapiens OX=9606 GN=RRM2B PE=1 SV=1	0,473372126	2,255225
Q9H814	Phosphorylated adapter RNA export protein OS=Homo sapiens OX=9606 GN=PHAX PE=1 SV=1	0,290202424	2,252996
O14744	Protein arginine N-methyltransferase 5 OS=Homo sapiens OX=9606 GN=PRMT5 PE=1 SV=4	0,177349997	2,252751
Q12830	Nucleosome-remodeling factor subunit BPTF OS=Homo sapiens OX=9606 GN=BPTF PE=1 SV=3	0,571718203	2,251156
Q96CV9	Optineurin OS=Homo sapiens OX=9606 GN=OPTN PE=1 SV=3	-0,182350701	2,248541
Q86WR0	Coiled-coil domain-containing protein 25 OS=Homo sapiens OX=9606 GN=CCDC25 PE=1 SV=2	-0,471735969	2,24704
Q7L7X3	Serine/threonine-protein kinase TAO1 OS=Homo sapiens OX=9606 GN=TAOK1 PE=1 SV=1	0,348091475	2,245307
A6NCE7	Microtubule-associated proteins 1A/1B light chain 3 beta 2 OS=Homo sapiens OX=9606 GN=MAP1LC3B2 PE=2 SV=1	0,494270886	2,24496
O95833	Chloride intracellular channel protein 3 OS=Homo sapiens OX=9606 GN=CLIC3 PE=1 SV=2	0,19386784	2,239979
Q96C36	Pyrroline-5-carboxylate reductase 2 OS=Homo sapiens OX=9606 GN=PYCR2 PE=1 SV=1	-0,227433554	2,238571
Q9NPG1	Frizzled-3 OS=Homo sapiens OX=9606 GN=FZD3 PE=1 SV=1	0,270112408	2,237779
Q5T4B2	Inactive glycosyltransferase 25 family member 3 OS=Homo sapiens OX=9606 GN=CERCAM PE=1 SV=1	-0,383028549	2,237696
Q5VW36	Focadhesin OS=Homo sapiens OX=9606 GN=FOCAD PE=1 SV=1	-0,268578719	2,235981
Q9UN37	Vacuolar protein sorting-associated protein 4A OS=Homo sapiens OX=9606 GN=VPS4A PE=1 SV=1	-0,268564743	2,235835
P82914	28S ribosomal protein S15, mitochondrial OS=Homo sapiens OX=9606 GN=MRPS15 PE=1 SV=1	-0,620229033	2,23476
Q8IX12	Cell division cycle and apoptosis regulator protein 1 OS=Homo sapiens OX=9606 GN=CCAR1 PE=1 SV=2	0,193505454	2,234462
Q9H223	EH domain-containing protein 4 OS=Homo sapiens OX=9606 GN=EHD4 PE=1 SV=1	0,176252718	2,234228
Q96KQ7	Histone-lysine N-methyltransferase EHMT2 OS=Homo sapiens OX=9606 GN=EHMT2 PE=1 SV=3	-0,619535051	2,232487
A1L020	RNA-binding protein MEX3A OS=Homo sapiens OX=9606 GN=MEX3A PE=1 SV=1	-0,505114792	2,231399
Q9NX47	E3 ubiquitin-protein ligase MARCH5 OS=Homo sapiens OX=9606 GN=MARCH5 PE=1 SV=1	-1,209232395	2,231322
Q15637	Splicing factor 1 OS=Homo sapiens OX=9606 GN=SF1 PE=1 SV=4	-0,208054684	2,231158
O43819	Protein SCO2 homolog, mitochondrial OS=Homo sapiens OX=9606 GN=SCO2 PE=1 SV=3	-0,47573305	2,2256
O75521	Enoyl-CoA delta isomerase 2, mitochondrial OS=Homo sapiens OX=9606 GN=ECI2 PE=1 SV=4	0,216278225	2,223645
Q96A65	Exocyst complex component 4 OS=Homo sapiens OX=9606 GN=EXOC4 PE=1 SV=1	0,154899259	2,221157
Q9UKM9	RNA-binding protein Raly OS=Homo sapiens OX=9606 GN=RALY PE=1 SV=1	-0,225999392	2,220347
P17174	Aspartate aminotransferase, cytoplasmic OS=Homo sapiens OX=9606 GN=GOT1 PE=1 SV=3	0,175338414	2,218833
O60216	Double-strand-break repair protein rad21 homolog OS=Homo sapiens OX=9606 GN=RAD21 PE=1 SV=2	-0,225824593	2,218128
P28070	Proteasome subunit beta type-4 OS=Homo sapiens OX=9606 GN=PSMB4 PE=1 SV=4	-0,286473432	2,217203
P51610	Host cell factor 1 OS=Homo sapiens OX=9606 GN=HCFC1 PE=1 SV=2	-0,128833016	2,216552
O15126	Secretory carrier-associated membrane protein 1 OS=Homo sapiens OX=9606 GN=SCAMP1 PE=1 SV=2	-0,344243239	2,216037

Q9UKV3	Apoptotic chromatin condensation inducer in the nucleus OS=Homo sapiens OX=9606 GN=ACIN1 PE=1 SV=2	0,175168622	2,215978
Q7L775	EPM2A-interacting protein 1 OS=Homo sapiens OX=9606 GN=EPM2AIP1 PE=1 SV=1	-0,390490633	2,212415
Q8WTS6	Histone-lysine N-methyltransferase SETD7 OS=Homo sapiens OX=9606 GN=SETD7 PE=1 SV=1	0,464592189	2,211238
P17301	Integrin alpha-2 OS=Homo sapiens OX=9606 GN=ITGA2 PE=1 SV=1	0,265944753	2,208459
Q13042	Cell division cycle protein 16 homolog OS=Homo sapiens OX=9606 GN=CDC16 PE=1 SV=2	0,265631131	2,205187
Q9BVP2	Guanine nucleotide-binding protein-like 3 OS=Homo sapiens OX=9606 GN=GNL3 PE=1 SV=2	-0,342800018	2,205068
P06493	Cyclin-dependent kinase 1 OS=Homo sapiens OX=9606 GN=CDK1 PE=1 SV=3	-0,19140364	2,202546
P55265	Double-stranded RNA-specific adenosine deaminase OS=Homo sapiens OX=9606 GN=ADAR PE=1 SV=4	-0,122624858	2,200132
Q9Y536	#N/D	0,462299614	2,199727
Q8WUA2	Peptidyl-prolyl cis-trans isomerase-like 4 OS=Homo sapiens OX=9606 GN=PPIL4 PE=1 SV=1	0,30929257	2,199086
P61086	Ubiquitin-conjugating enzyme E2 K OS=Homo sapiens OX=9606 GN=UBE2K PE=1 SV=3	-0,214260625	2,196725
Q86XI2	Condensin-2 complex subunit G2 OS=Homo sapiens OX=9606 GN=NCAPG2 PE=1 SV=1	-0,264795812	2,196476
Q9Y6A5	Transforming acidic coiled-coil-containing protein 3 OS=Homo sapiens OX=9606 GN=TACC3 PE=1 SV=1	-0,284245917	2,195872
Q9Y2R5	28S ribosomal protein S17, mitochondrial OS=Homo sapiens OX=9606 GN=MRPS17 PE=1 SV=1	-0,460314877	2,189754
P40429	60S ribosomal protein L13a OS=Homo sapiens OX=9606 GN=RPL13A PE=1 SV=2	-0,23474577	2,187368
Q05932	Folylpolyglutamate synthase, mitochondrial OS=Homo sapiens OX=9606 GN=FPGS PE=1 SV=3	-0,34040026	2,186838
Q9NXE8	Pre-mRNA-splicing factor CWC25 homolog OS=Homo sapiens OX=9606 GN=CWC25 PE=1 SV=1	0,622392391	2,186063
Q13263	Transcription intermediary factor 1-beta OS=Homo sapiens OX=9606 GN=TRIM28 PE=1 SV=5	-0,127472585	2,18491
P49458	Signal recognition particle 9 kDa protein OS=Homo sapiens OX=9606 GN=SRP9 PE=1 SV=2	-0,339882629	2,182907
Q13144	Translation initiation factor eIF-2B subunit epsilon OS=Homo sapiens OX=9606 GN=EIF2B5 PE=1 SV=3	-0,263490334	2,182879
P00441	Superoxide dismutase [Cu-Zn] OS=Homo sapiens OX=9606 GN=SOD1 PE=1 SV=2	0,339521605	2,180166
O00469	Procollagen-lysine,2-oxoglutarate 5-dioxygenase 2 OS=Homo sapiens OX=9606 GN=PLOD2 PE=1 SV=2	0,178128659	2,179805
P00505	Aspartate aminotransferase, mitochondrial OS=Homo sapiens OX=9606 GN=GOT2 PE=1 SV=3	0,173009729	2,179786
P22304	Iduronate 2-sulfatase OS=Homo sapiens OX=9606 GN=IDS PE=1 SV=1	-0,728057704	2,17828
Q969G3	SWI/SNF-related matrix-associated actin-dependent regulator of chromatin subfamily E member 1 OS=Homo sapiens OX=	-0,247013715	2,17585
Q96KM6	Zinc finger protein 512B OS=Homo sapiens OX=9606 GN=ZNF512B PE=1 SV=1	-0,597341401	2,173153
Q76M96	Coiled-coil domain-containing protein 80 OS=Homo sapiens OX=9606 GN=CCDC80 PE=1 SV=1	-0,306254485	2,172872
Q7Z4H3	HD domain-containing protein 2 OS=Homo sapiens OX=9606 GN=HDDC2 PE=1 SV=1	-0,384083164	2,171305
P49589	Cysteine--tRNA ligase, cytoplasmic OS=Homo sapiens OX=9606 GN=CARS PE=1 SV=3	0,14307542	2,17023
Q9H0U3	Magnesium transporter protein 1 OS=Homo sapiens OX=9606 GN=MAGT1 PE=1 SV=1	-0,455684912	2,16646
Q8NFW8	N-acylneuraminate cytidylyltransferase OS=Homo sapiens OX=9606 GN=CMAS PE=1 SV=2	0,337493658	2,164774
P82933	28S ribosomal protein S9, mitochondrial OS=Homo sapiens OX=9606 GN=MRPS9 PE=1 SV=2	-0,26169667	2,164226
Q6P996	Pyridoxal-dependent decarboxylase domain-containing protein 1 OS=Homo sapiens OX=9606 GN=PDXDC1 PE=1 SV=2	0,163021861	2,162919
Q96AE4	Far upstream element-binding protein 1 OS=Homo sapiens OX=9606 GN=FUBP1 PE=1 SV=3	-0,142686095	2,162299
Q969T9	WW domain-binding protein 2 OS=Homo sapiens OX=9606 GN=WBP2 PE=1 SV=1	0,304641236	2,15897
Q99700	Ataxin-2 OS=Homo sapiens OX=9606 GN=ATXN2 PE=1 SV=2	0,280236157	2,15757
Q96A33	Coiled-coil domain-containing protein 47 OS=Homo sapiens OX=9606 GN=CCDC47 PE=1 SV=1	0,202622811	2,155412
Q8N1F7	Nuclear pore complex protein Nup93 OS=Homo sapiens OX=9606 GN=NUP93 PE=1 SV=2	-0,13469049	2,155145
O00483	Cytochrome c oxidase subunit NDUFA4 OS=Homo sapiens OX=9606 GN=NDUFA4 PE=1 SV=1	-0,453141897	2,153648
Q9ULH7	Myocardin-related transcription factor B OS=Homo sapiens OX=9606 GN=MRTFB PE=1 SV=3	-0,178513727	2,152359
Q15084	Protein disulfide-isomerase A6 OS=Homo sapiens OX=9606 GN=PDIA6 PE=1 SV=1	0,176317954	2,150542
Q9HAU6	Putative translationally-controlled tumor protein-like protein TPT1P8 OS=Homo sapiens OX=9606 GN=TPT1P8 PE=5 SV=2	-1,136732322	2,149007
Q71RC2	La-related protein 4 OS=Homo sapiens OX=9606 GN=LARP4 PE=1 SV=3	0,260130874	2,147971
Q9NR19	Acetyl-coenzyme A synthetase, cytoplasmic OS=Homo sapiens OX=9606 GN=ACSS2 PE=1 SV=1	-0,451990548	2,147844
Q14353	Guanidinoacetate N-methyltransferase OS=Homo sapiens OX=9606 GN=GAMT PE=1 SV=1	-0,279190928	2,147606
O96005	Cleft lip and palate transmembrane protein 1 OS=Homo sapiens OX=9606 GN=CLPTM1 PE=1 SV=1	-0,334704687	2,143622
O43765	Small glutamine-rich tetratricopeptide repeat-containing protein alpha OS=Homo sapiens OX=9606 GN=SGTA PE=1 SV=1	0,219839217	2,142534
Q8IV48	3'-5' exoribonuclease 1 OS=Homo sapiens OX=9606 GN=ERI1 PE=1 SV=3	-0,334514619	2,142181
O43426	Synaptojanin-1 OS=Homo sapiens OX=9606 GN=SYNJ1 PE=1 SV=2	0,334488854	2,141985
Q9NQH7	#N/D	0,298193494	2,141611
P50613	Cyclin-dependent kinase 7 OS=Homo sapiens OX=9606 GN=CDK7 PE=1 SV=1	-0,450594011	2,140801
P23246	Splicing factor, proline- and glutamine-rich OS=Homo sapiens OX=9606 GN=SFPQ PE=1 SV=2	-0,147431212	2,13981
Q9Y5X2	Sorting nexin-8 OS=Homo sapiens OX=9606 GN=SNX8 PE=1 SV=1	-0,302089108	2,137005
P12235	ADP/ATP translocase 1 OS=Homo sapiens OX=9606 GN=SLC25A4 PE=1 SV=4	0,378654973	2,136474
Q9H0X9	Oxysterol-binding protein-related protein 5 OS=Homo sapiens OX=9606 GN=OSBPL5 PE=1 SV=1	-0,301829497	2,134772
Q8TB72	Pumilio homolog 2 OS=Homo sapiens OX=9606 GN=PUM2 PE=1 SV=2	-0,378388725	2,134766
Q86TP1	Exopolyphosphatase PRUNE1 OS=Homo sapiens OX=9606 GN=PRUNE1 PE=1 SV=2	-0,58511596	2,134654
O15381	Nuclear valosin-containing protein-like OS=Homo sapiens OX=9606 GN=NVL PE=1 SV=1	-0,589314113	2,132385
Q9GZV4	#N/D	0,333205234	2,132257
O00754	Lysosomal alpha-mannosidase OS=Homo sapiens OX=9606 GN=MAN2B1 PE=1 SV=3	-0,219003052	2,132031
O75152	Zinc finger CCCH domain-containing protein 11A OS=Homo sapiens OX=9606 GN=ZC3H11A PE=1 SV=3	0,30143575	2,131387
P11216	Glycogen phosphorylase, brain form OS=Homo sapiens OX=9606 GN=PYGB PE=1 SV=5	0,115002294	2,130153
P04040	Catalase OS=Homo sapiens OX=9606 GN=CAT PE=1 SV=3	0,193289829	2,129898
Q15022	Polycomb protein SUZ12 OS=Homo sapiens OX=9606 GN=SUZ12 PE=1 SV=3	-0,448290367	2,129175
Q9UHD8	Septin-9 OS=Homo sapiens OX=9606 GN=SEPTIN9 PE=1 SV=2	-0,126988796	2,128609
Q969V3	Nicalin OS=Homo sapiens OX=9606 GN=NCLN PE=1 SV=2	-0,21839439	2,124395
Q8NF91	Nesprin-1 OS=Homo sapiens OX=9606 GN=SYNE1 PE=1 SV=4	-0,218075193	2,120393
Q96GX5	Serine/threonine-protein kinase greatwall OS=Homo sapiens OX=9606 GN=MASTL PE=1 SV=1	-0,446323762	2,119243
Q9Y6Y8	SEC23-interacting protein OS=Homo sapiens OX=9606 GN=SEC23IP PE=1 SV=1	-0,164671219	2,117413
Q96CS2	HAUS augmin-like complex subunit 1 OS=Homo sapiens OX=9606 GN=HAUS1 PE=1 SV=1	-0,584813699	2,117291
P53677	AP-3 complex subunit mu-2 OS=Homo sapiens OX=9606 GN=AP3M2 PE=2 SV=1	-0,444503764	2,110045
O00471	Exocyst complex component 5 OS=Homo sapiens OX=9606 GN=EXOC5 PE=1 SV=1	0,275223995	2,109871
Q9H4A5	Golgi phosphoprotein 3-like OS=Homo sapiens OX=9606 GN=GOLPH3L PE=1 SV=1	-0,444167638	2,108345
Q9UBP6	tRNA (guanine-N(7)-)-methyltransferase OS=Homo sapiens OX=9606 GN=METTL1 PE=1 SV=1	-0,404462003	2,103862
Q01081	Splicing factor U2AF 35 kDa subunit OS=Homo sapiens OX=9606 GN=U2AF1 PE=1 SV=3	-0,255700869	2,102129
Q9Y2X7	ARF GTPase-activating protein GIT1 OS=Homo sapiens OX=9606 GN=GIT1 PE=1 SV=2	-0,191281252	2,100999
P39060	Collagen alpha-1(XVIII) chain OS=Homo sapiens OX=9606 GN=COL18A1 PE=1 SV=5	0,373038387	2,100435
O14979	Heterogeneous nuclear ribonucleoprotein D-like OS=Homo sapiens OX=9606 GN=HNRNPDL PE=1 SV=3	-0,240229472	2,100258
P26038	Moesin OS=Homo sapiens OX=9606 GN=MSN PE=1 SV=3	0,115192161	2,09749
Q9H9E3	Conserved oligomeric Golgi complex subunit 4 OS=Homo sapiens OX=9606 GN=COG4 PE=1 SV=3	-0,328521302	2,096792
O96007	Molybdopterin synthase catalytic subunit OS=Homo sapiens OX=9606 GN=MOCS2 PE=1 SV=1	-0,441478831	2,094745
Q9Y3A5	Ribosome maturation protein SBDS OS=Homo sapiens OX=9606 GN=SBDS PE=1 SV=4	0,190630614	2,091664
P05413	Fatty acid-binding protein, heart OS=Homo sapiens OX=9606 GN=FABP3 PE=1 SV=4	0,440170766	2,088124
P27658	Collagen alpha-1(VIII) chain OS=Homo sapiens OX=9606 GN=COL8A1 PE=1 SV=2	1,105693099	2,087431
Q9UBP4	Dickkopf-related protein 3 OS=Homo sapiens OX=9606 GN=DKK3 PE=1 SV=2	0,439945451	2,086983
Q8WVD3	E3 ubiquitin-protein ligase RNF138 OS=Homo sapiens OX=9606 GN=RNF138 PE=1 SV=1	-0,286937761	2,08582
Q6NZI2	Caveolae-associated protein 1 OS=Homo sapiens OX=9606 GN=CAVIN1 PE=1 SV=1	0,190151347	2,084796
Q9H9B1	Histone-lysine N-methyltransferase EHMT1 OS=Homo sapiens OX=9606 GN=EHMT1 PE=1 SV=4	-0,370402391	2,083523
P18859	ATP synthase-coupling factor 6, mitochondrial OS=Homo sapiens OX=9606 GN=ATP5PF PE=1 SV=1	-0,574027895	2,080921
Q8IVL0	Neuron navigator 3 OS=Homo sapiens OX=9606 GN=NAV3 PE=1 SV=3	0,427155431	2,079955
Q8TCF1	AN1-type zinc finger protein 1 OS=Homo sapiens OX=9606 GN=ZFAND1 PE=1 SV=1	-1,076268105	2,076906

Q9P107	GEM-interacting protein OS=Homo sapiens OX=9606 GN=GMIP PE=1 SV=2	0,349953938	2,076547
Q96I25	Splicing factor 45 OS=Homo sapiens OX=9606 GN=RBM17 PE=1 SV=1	0,271662229	2,0761
Q8IX01	SURP and G-patch domain-containing protein 2 OS=Homo sapiens OX=9606 GN=SUGP2 PE=1 SV=2	0,18296685	2,075909
P19623	Spermidine synthase OS=Homo sapiens OX=9606 GN=SRM PE=1 SV=1	-0,252907965	2,073341
Q6ZXV5	Protein O-mannosyl-transferase TMTC3 OS=Homo sapiens OX=9606 GN=TMTC3 PE=1 SV=2	0,325416575	2,073315
Q92973	Transportin-1 OS=Homo sapiens OX=9606 GN=TNPO1 PE=1 SV=2	0,122506537	2,070869
Q9P035	Very-long-chain (3R)-3-hydroxyacyl-CoA dehydratase 3 OS=Homo sapiens OX=9606 GN=HACD3 PE=1 SV=2	0,270972713	2,069575
Q9UBS8	E3 ubiquitin-protein ligase RNF14 OS=Homo sapiens OX=9606 GN=RNF14 PE=1 SV=1	-1,712154018	2,069263
P01112	GTPase HRas OS=Homo sapiens OX=9606 GN=HRAS PE=1 SV=1	0,569915049	2,06698
Q9P015	39S ribosomal protein L15, mitochondrial OS=Homo sapiens OX=9606 GN=MRPL15 PE=1 SV=1	-0,324523533	2,066567
Q8N392	Rho GTPase-activating protein 18 OS=Homo sapiens OX=9606 GN=ARHGAP18 PE=1 SV=3	-0,150099672	2,066543
P49642	DNA primase small subunit OS=Homo sapiens OX=9606 GN=PRIM1 PE=1 SV=1	-0,367474864	2,064742
Q9UG63	ATP-binding cassette sub-family F member 2 OS=Homo sapiens OX=9606 GN=ABCF2 PE=1 SV=2	0,170939431	2,064395
Q05397	Focal adhesion kinase 1 OS=Homo sapiens OX=9606 GN=PTK2 PE=1 SV=2	-0,251851352	2,062473
P24386	#N/D	-0,234173474	2,061704
O60508	Pre-mRNA-processing factor 17 OS=Homo sapiens OX=9606 GN=CDC40 PE=1 SV=1	0,83955549	2,061116
P60510	Serine/threonine-protein phosphatase 4 catalytic subunit OS=Homo sapiens OX=9606 GN=PPP4C PE=1 SV=1	0,323782303	2,060968
Q9H2V7	Protein spinster homolog 1 OS=Homo sapiens OX=9606 GN=SPNS1 PE=1 SV=1	-1,27199761	2,06075
Q6ICB0	Desumoylating isopeptidase 1 OS=Homo sapiens OX=9606 GN=DESI1 PE=1 SV=1	-1,063061661	2,060713
Q9UI15	Transgelin-3 OS=Homo sapiens OX=9606 GN=TAGLN3 PE=1 SV=2	0,567800892	2,059798
Q8IZ83	Aldehyde dehydrogenase family 16 member A1 OS=Homo sapiens OX=9606 GN=ALDH16A1 PE=1 SV=2	-0,236516015	2,05916
Q92797	Symplekin OS=Homo sapiens OX=9606 GN=SYMPK PE=1 SV=2	-0,188357363	2,05915
O43237	Cytoplasmic dynein 1 light intermediate chain 2 OS=Homo sapiens OX=9606 GN=DYNC1LI2 PE=1 SV=1	0,223911905	2,058981
O60826	Coiled-coil domain-containing protein 22 OS=Homo sapiens OX=9606 GN=CCDC22 PE=1 SV=1	-0,251348923	2,05731
O14519	Cyclin-dependent kinase 2-associated protein 1 OS=Homo sapiens OX=9606 GN=CDK2AP1 PE=1 SV=1	-0,433868114	2,056182
A8CG34	Nuclear envelope pore membrane protein POM 121C OS=Homo sapiens OX=9606 GN=POM121C PE=1 SV=3	-0,322820327	2,053704
Q9NRW1	Ras-related protein Rab-6B OS=Homo sapiens OX=9606 GN=RAB6B PE=1 SV=1	0,3651404	2,049768
Q2TB90	Putative hexokinase HKDC1 OS=Homo sapiens OX=9606 GN=HKDC1 PE=1 SV=3	0,667144525	2,046961
Q9BRT3	Migration and invasion enhancer 1 OS=Homo sapiens OX=9606 GN=MIEN1 PE=1 SV=1	-0,563380112	2,044746
Q9NWH9	SAFB-like transcription modulator OS=Homo sapiens OX=9606 GN=SLTM PE=1 SV=2	-0,211921916	2,043666
Q9Y5U2	Protein TSSC4 OS=Homo sapiens OX=9606 GN=TSSC4 PE=1 SV=3	-0,561693324	2,038991
P40121	Macrophage-capping protein OS=Homo sapiens OX=9606 GN=CAPG PE=1 SV=2	-0,186857762	2,037788
P16278	Beta-galactosidase OS=Homo sapiens OX=9606 GN=GLB1 PE=1 SV=2	0,221853895	2,034826
P36871	Phosphoglucomutase-1 OS=Homo sapiens OX=9606 GN=PGM1 PE=1 SV=3	0,141922067	2,033739
Q8TCG2	Phosphatidylinositol 4-kinase type 2-beta OS=Homo sapiens OX=9606 GN=PI4K2B PE=1 SV=1	-1,040731397	2,032952
Q7Z422	SUZ domain-containing protein 1 OS=Homo sapiens OX=9606 GN=SZRD1 PE=1 SV=1	0,429202238	2,032495
Q9P0U1	Mitochondrial import receptor subunit TOM7 homolog OS=Homo sapiens OX=9606 GN=TOMM7 PE=1 SV=1	-1,04027274	2,032377
O43566	Regulator of G-protein signaling 14 OS=Homo sapiens OX=9606 GN=RGS14 PE=1 SV=4	-0,625218093	2,031753
Q14692	Ribosome biogenesis protein BMS1 homolog OS=Homo sapiens OX=9606 GN=BMS1 PE=1 SV=1	-0,362322915	2,031698
Q9Y3C8	Ubiquitin-fold modifier-conjugating enzyme 1 OS=Homo sapiens OX=9606 GN=UFC1 PE=1 SV=3	0,289740794	2,031213
O60306	RNA helicase aquarius OS=Homo sapiens OX=9606 GN=AQR PE=1 SV=4	0,179941849	2,031088
Q6STE5	#N/D	-0,36213059	2,030464
Q96FC9	ATP-dependent DNA helicase DDX11 OS=Homo sapiens OX=9606 GN=DDX11 PE=1 SV=1	0,181130206	2,027333
O15530	3-phosphoinositide-dependent protein kinase 1 OS=Homo sapiens OX=9606 GN=PDPK1 PE=1 SV=1	0,42800972	2,026436
Q86Y33	Cell division cycle protein 20 homolog B OS=Homo sapiens OX=9606 GN=CDC20B PE=1 SV=3	-1,202495009	2,026333
Q9HCP0	Casein kinase I isoform gamma-1 OS=Homo sapiens OX=9606 GN=CSNK1G1 PE=1 SV=1	0,244231441	2,026063
Q9Y2Y0	ADP-ribosylation factor-like protein 2-binding protein OS=Homo sapiens OX=9606 GN=ARL2BP PE=1 SV=1	-0,248185245	2,024865
P30613	Pyruvate kinase PKLR OS=Homo sapiens OX=9606 GN=PKLR PE=1 SV=2	0,467845462	2,024819
Q9NSI6	Bromodomain and WD repeat-containing protein 1 OS=Homo sapiens OX=9606 GN=BRWD1 PE=1 SV=4	-0,082832332	2,023386
Q14126	Desmoglein-2 OS=Homo sapiens OX=9606 GN=DSG2 PE=1 SV=2	0,168325663	2,022953
Q99611	Selenide, water dikinase 2 OS=Homo sapiens OX=9606 GN=SEPHS2 PE=1 SV=3	-0,746901709	2,022847
P10301	Ras-related protein R-Ras OS=Homo sapiens OX=9606 GN=RRAS PE=1 SV=1	-0,265984766	2,022491
O15085	Rho guanine nucleotide exchange factor 11 OS=Homo sapiens OX=9606 GN=ARHGEF11 PE=1 SV=1	-0,399515953	2,021939
P16435	NADPH--cytochrome P450 reductase OS=Homo sapiens OX=9606 GN=POR PE=1 SV=2	0,158953237	2,020664
Q06481	Amyloid-like protein 2 OS=Homo sapiens OX=9606 GN=APLP2 PE=1 SV=2	0,555956544	2,019366
Q5VZ89	DENN domain-containing protein 4C OS=Homo sapiens OX=9606 GN=DENND4C PE=1 SV=3	0,318201108	2,018859
A5YKK6	CCR4-NOT transcription complex subunit 1 OS=Homo sapiens OX=9606 GN=CNOT1 PE=1 SV=2	0,124040072	2,018603
P80217	Interferon-induced 35 kDa protein OS=Homo sapiens OX=9606 GN=IFI35 PE=1 SV=5	-0,247472515	2,017572
Q8NBN3	Transmembrane protein 87A OS=Homo sapiens OX=9606 GN=TMEM87A PE=1 SV=3	0,368036103	2,016674
Q9HB07	UPF0160 protein MYG1, mitochondrial OS=Homo sapiens OX=9606 GN=C12orf10 PE=1 SV=2	-0,23257554	2,015771
P98194	Calcium-transporting ATPase type 2C member 1 OS=Homo sapiens OX=9606 GN=ATP2C1 PE=1 SV=3	0,317704724	2,015118
O00161	Synaptosomal-associated protein 23 OS=Homo sapiens OX=9606 GN=SNAP23 PE=1 SV=1	0,247167637	2,014455
Q99985	Semaphorin-3C OS=Homo sapiens OX=9606 GN=SEMA3C PE=2 SV=2	-0,172977542	2,013551
Q96DI7	U5 small nuclear ribonucleoprotein 40 kDa protein OS=Homo sapiens OX=9606 GN=SNRNP40 PE=1 SV=1	-0,21996099	2,012675
P04843	Dolichyl-diphosphooligosaccharide--protein glycosyltransferase subunit 1 OS=Homo sapiens OX=9606 GN=RPN1 PE=1 SV=	0,132665408	2,012161
Q00587	Cdc42 effector protein 1 OS=Homo sapiens OX=9606 GN=CDC42EP1 PE=1 SV=1	0,375726329	2,01065
O95817	BAG family molecular chaperone regulator 3 OS=Homo sapiens OX=9606 GN=BAG3 PE=1 SV=3	0,137768082	2,009264
Q13426	DNA repair protein XRCC4 OS=Homo sapiens OX=9606 GN=XRCC4 PE=1 SV=2	-0,552544456	2,007657
P05026	Sodium/potassium-transporting ATPase subunit beta-1 OS=Homo sapiens OX=9606 GN=ATP1B1 PE=1 SV=1	0,316669383	2,007318
O75431	Metaxin-2 OS=Homo sapiens OX=9606 GN=MTX2 PE=1 SV=1	-0,246425174	2,006866
Q9NYK5	39S ribosomal protein L39, mitochondrial OS=Homo sapiens OX=9606 GN=MRPL39 PE=1 SV=3	-0,286866909	2,006713
Q7Z3J2	VPS35 endosomal protein sorting factor-like OS=Homo sapiens OX=9606 GN=VPS35L PE=1 SV=2	-0,167163676	2,004619
E9PAV3	Nascent polypeptide-associated complex subunit alpha, muscle-specific form OS=Homo sapiens OX=9606 GN=NACA PE=1	-0,316232291	2,004026
Q8TD55	Pleckstrin homology domain-containing family O member 2 OS=Homo sapiens OX=9606 GN=PLEKHO2 PE=1 SV=1	-0,551201048	2,00304
Q8N6R0	eEF1A lysine and N-terminal methyltransferase OS=Homo sapiens OX=9606 GN=EEF1AKNMT PE=1 SV=1	-0,263598837	2,000045
P11142	Heat shock cognate 71 kDa protein OS=Homo sapiens OX=9606 GN=HSPA8 PE=1 SV=1	-0,132049169	1,999706
Q14157	Ubiquitin-associated protein 2-like OS=Homo sapiens OX=9606 GN=UBAP2L PE=1 SV=2	-0,153642334	1,99941
Q15020	Squamous cell carcinoma antigen recognized by T-cells 3 OS=Homo sapiens OX=9606 GN=SART3 PE=1 SV=1	0,191168517	1,998296
P61326	Protein mago nashi homolog OS=Homo sapiens OX=9606 GN=MAGOH PE=1 SV=1	-0,285836841	1,997944
Q15024	Exosome complex component RRP42 OS=Homo sapiens OX=9606 GN=EXOSC7 PE=1 SV=3	0,549448522	1,99701
Q13427	Peptidyl-prolyl cis-trans isomerase G OS=Homo sapiens OX=9606 GN=PPIG PE=1 SV=2	0,315143288	1,995827
P60520	Gamma-aminobutyric acid receptor-associated protein-like 2 OS=Homo sapiens OX=9606 GN=GABARAPL2 PE=1 SV=1	0,356624267	1,995161
P15880	40S ribosomal protein S2 OS=Homo sapiens OX=9606 GN=RPS2 PE=1 SV=2	-0,177408389	1,99379
Q08J23	tRNA (cytosine(34)-C(5))-methyltransferase OS=Homo sapiens OX=9606 GN=NSUN2 PE=1 SV=2	-0,146094509	1,99366
O94967	WD repeat-containing protein 47 OS=Homo sapiens OX=9606 GN=WDR47 PE=1 SV=1	0,370828155	1,993635
Q9NT99	Leucine-rich repeat-containing protein 4B OS=Homo sapiens OX=9606 GN=LRRC4B PE=2 SV=3	-0,339832968	1,992486
O60739	Eukaryotic translation initiation factor 1b OS=Homo sapiens OX=9606 GN=EIF1B PE=1 SV=2	1,008806486	1,992409
O14737	Programmed cell death protein 5 OS=Homo sapiens OX=9606 GN=PDCD5 PE=1 SV=3	0,262410985	1,988889
Q03013	Glutathione S-transferase Mu 4 OS=Homo sapiens OX=9606 GN=GSTM4 PE=1 SV=3	-0,42046177	1,988035
Q9BTT0	Acidic leucine-rich nuclear phosphoprotein 32 family member E OS=Homo sapiens OX=9606 GN=ANP32E PE=1 SV=1	-0,314087458	1,987881
Q96SU4	Oxysterol-binding protein-related protein 9 OS=Homo sapiens OX=9606 GN=OSBPL9 PE=1 SV=2	-0,217825891	1,987769

O43670	BUB3-interacting and GLEBS motif-containing protein ZNF207 OS=Homo sapiens OX=9606 GN=ZNF207 PE=1 SV=1	-0,420388872	1,987664
Q6Y288	Beta-1,3-glucosyltransferase OS=Homo sapiens OX=9606 GN=B3GLCT PE=1 SV=2	-1,004046153	1,986276
P51808	Dynein light chain Tctex-type 3 OS=Homo sapiens OX=9606 GN=DYNLT3 PE=1 SV=1	0,546312502	1,986201
Q9UI30	Multifunctional methyltransferase subunit TRM112-like protein OS=Homo sapiens OX=9606 GN=TRMT112 PE=1 SV=1	0,419394915	1,982601
Q05519	Serine/arginine-rich splicing factor 11 OS=Homo sapiens OX=9606 GN=SRSF11 PE=1 SV=1	-0,283837401	1,980939
O76024	Wolframin OS=Homo sapiens OX=9606 GN=WFS1 PE=1 SV=2	-0,197806194	1,98078
Q13123	Protein Red OS=Homo sapiens OX=9606 GN=IK PE=1 SV=3	-0,261474527	1,980103
P33316	Deoxyuridine 5'-triphosphate nucleotidohydrolase, mitochondrial OS=Homo sapiens OX=9606 GN=DUT PE=1 SV=4	-0,312959006	1,979393
P82909	28S ribosomal protein S36, mitochondrial OS=Homo sapiens OX=9606 GN=MRPS36 PE=1 SV=2	-0,54400619	1,978238
Q9BT23	LIM domain-containing protein 2 OS=Homo sapiens OX=9606 GN=LIMD2 PE=1 SV=1	0,311374753	1,978121
P13674	Prolyl 4-hydroxylase subunit alpha-1 OS=Homo sapiens OX=9606 GN=P4HA1 PE=1 SV=2	0,152271831	1,975891
Q92785	Zinc finger protein ubi-d4 OS=Homo sapiens OX=9606 GN=DPF2 PE=1 SV=2	-0,353507256	1,975183
P05204	Non-histone chromosomal protein HMG-17 OS=Homo sapiens OX=9606 GN=HMGN2 PE=1 SV=3	-0,994557849	1,97398
O00186	Syntaxin-binding protein 3 OS=Homo sapiens OX=9606 GN=STXBP3 PE=1 SV=2	0,228729775	1,973649
Q13057	Bifunctional coenzyme A synthase OS=Homo sapiens OX=9606 GN=COASY PE=1 SV=4	-0,216566785	1,97312
O94906	Pre-mRNA-processing factor 6 OS=Homo sapiens OX=9606 GN=PRPF6 PE=1 SV=1	-0,133144371	1,971251
O00142	Thymidine kinase 2, mitochondrial OS=Homo sapiens OX=9606 GN=TK2 PE=1 SV=4	-0,690093357	1,964489
Q99808	Equilibrative nucleoside transporter 1 OS=Homo sapiens OX=9606 GN=SLC29A1 PE=1 SV=3	-0,415776918	1,964159
Q00613	Heat shock factor protein 1 OS=Homo sapiens OX=9606 GN=HSF1 PE=1 SV=1	-0,986396781	1,963328
Q86XZ4	Spermatogenesis-associated serine-rich protein 2 OS=Homo sapiens OX=9606 GN=SPATS2 PE=1 SV=1	0,351488811	1,962251
Q7L576	Cytoplasmic FMR1-interacting protein 1 OS=Homo sapiens OX=9606 GN=CYFIP1 PE=1 SV=1	-0,14111856	1,96168
O95551	Tyrosyl-DNA phosphodiesterase 2 OS=Homo sapiens OX=9606 GN=TDP2 PE=1 SV=1	0,608817314	1,959842
Q07960	Rho GTPase-activating protein 1 OS=Homo sapiens OX=9606 GN=ARHGAP1 PE=1 SV=1	-0,151309331	1,959431
Q99460	26S proteasome non-ATPase regulatory subunit 1 OS=Homo sapiens OX=9606 GN=PSMD1 PE=1 SV=2	0,110981677	1,959393
Q7L1Q6	Basic leucine zipper and W2 domain-containing protein 1 OS=Homo sapiens OX=9606 GN=BZW1 PE=1 SV=1	-0,144132718	1,958297
Q99519	Sialidase-1 OS=Homo sapiens OX=9606 GN=NEU1 PE=1 SV=1	0,5382211	1,958208
P31939	Bifunctional purine biosynthesis protein PURH OS=Homo sapiens OX=9606 GN=ATIC PE=1 SV=3	-0,119254814	1,956532
P30530	Tyrosine-protein kinase receptor UFO OS=Homo sapiens OX=9606 GN=AXL PE=1 SV=4	-0,174859568	1,95649
P40938	Replication factor C subunit 3 OS=Homo sapiens OX=9606 GN=RFC3 PE=1 SV=2	0,280891046	1,955926
Q9NZC9	SWI/SNF-related matrix-associated actin-dependent regulator of chromatin subfamily A-like protein 1 OS=Homo sapiens (	-0,537407863	1,955386
A1L0T0	Acetolactate synthase-like protein OS=Homo sapiens OX=9606 GN=ILVBL PE=1 SV=2	-0,21496106	1,954481
Q8NEM2	SHC SH2 domain-binding protein 1 OS=Homo sapiens OX=9606 GN=SHCBP1 PE=1 SV=3	-0,258337769	1,95073
P16220	Cyclic AMP-responsive element-binding protein 1 OS=Homo sapiens OX=9606 GN=CREB1 PE=1 SV=2	-0,412487215	1,947376
Q9Y315	Deoxyribose-phosphate aldolase OS=Homo sapiens OX=9606 GN=DERA PE=1 SV=2	-0,349138725	1,947197
Q9H270	Vacuolar protein sorting-associated protein 11 homolog OS=Homo sapiens OX=9606 GN=VPS11 PE=1 SV=1	0,534882071	1,946612
Q13363	C-terminal-binding protein 1 OS=Homo sapiens OX=9606 GN=CTBP1 PE=1 SV=2	-0,257856702	1,946234
Q5W111	SPRY domain-containing protein 7 OS=Homo sapiens OX=9606 GN=SPRYD7 PE=1 SV=2	0,257781347	1,945529
Q96CN4	EVI5-like protein OS=Homo sapiens OX=9606 GN=EVI5L PE=1 SV=1	-0,527164681	1,943521
Q13257	Mitotic spindle assembly checkpoint protein MAD2A OS=Homo sapiens OX=9606 GN=MAD2L1 PE=1 SV=1	-0,308093723	1,942843
Q8NEZ2	Vacuolar protein sorting-associated protein 37A OS=Homo sapiens OX=9606 GN=VPS37A PE=1 SV=1	0,278494311	1,935618
Q12768	WASH complex subunit 5 OS=Homo sapiens OX=9606 GN=WASHC5 PE=1 SV=1	-0,149833318	1,934282
Q96A35	39S ribosomal protein L24, mitochondrial OS=Homo sapiens OX=9606 GN=MRPL24 PE=1 SV=1	-0,409885211	1,934092
Q9NWY4	Histone PARylation factor 1 OS=Homo sapiens OX=9606 GN=HPF1 PE=1 SV=2	-0,278312778	1,934082
Q86Y82	Syntaxin-12 OS=Homo sapiens OX=9606 GN=STX12 PE=1 SV=1	0,3468623	1,932619
A6NDG6	Glycerol-3-phosphate phosphatase OS=Homo sapiens OX=9606 GN=PGP PE=1 SV=1	0,213019317	1,932006
Q14997	Proteasome activator complex subunit 4 OS=Homo sapiens OX=9606 GN=PSME4 PE=1 SV=2	-0,212912567	1,930772
O15084	Serine/threonine-protein phosphatase 6 regulatory ankyrin repeat subunit A OS=Homo sapiens OX=9606 GN=ANKRD28 P	-0,346498622	1,93029
Q96FX7	tRNA (adenine(58)-N(1))-methyltransferase catalytic subunit TRMT61A OS=Homo sapiens OX=9606 GN=TRMT61A PE=1 S'	-0,961109744	1,929874
Q8NFH5	Nucleoporin NUP35 OS=Homo sapiens OX=9606 GN=NUP35 PE=1 SV=1	0,306312448	1,929481
Q96BN8	Ubiquitin thioesterase otulin OS=Homo sapiens OX=9606 GN=OTULIN PE=1 SV=3	0,34632744	1,929194
P56385	ATP synthase subunit e, mitochondrial OS=Homo sapiens OX=9606 GN=ATP5ME PE=1 SV=2	0,529758233	1,928767
Q9NQL2	Ras-related GTP-binding protein D OS=Homo sapiens OX=9606 GN=RRAGD PE=1 SV=1	-0,528806743	1,925447
Q9Y5J1	U3 small nucleolar RNA-associated protein 18 homolog OS=Homo sapiens OX=9606 GN=UTP18 PE=1 SV=3	0,866534527	1,924611
Q567U6	Coiled-coil domain-containing protein 93 OS=Homo sapiens OX=9606 GN=CCDC93 PE=1 SV=2	0,277123467	1,924019
Q5T5U3	Rho GTPase-activating protein 21 OS=Homo sapiens OX=9606 GN=ARHGAP21 PE=1 SV=2	0,284455845	1,922459
Q08AD1	Calmodulin-regulated spectrin-associated protein 2 OS=Homo sapiens OX=9606 GN=CAMSAP2 PE=1 SV=3	0,193269551	1,922405
P49116	Nuclear receptor subfamily 2 group C member 2 OS=Homo sapiens OX=9606 GN=NR2C2 PE=1 SV=1	0,904300754	1,921143
Q8IWE2	Protein NOXP20 OS=Homo sapiens OX=9606 GN=FAM114A1 PE=1 SV=2	0,276598901	1,919584
Q15645	Pachytene checkpoint protein 2 homolog OS=Homo sapiens OX=9606 GN=TRIP13 PE=1 SV=2	-0,185310879	1,919353
Q92621	Nuclear pore complex protein Nup205 OS=Homo sapiens OX=9606 GN=NUP205 PE=1 SV=3	-0,117417892	1,916458
Q68CQ4	Digestive organ expansion factor homolog OS=Homo sapiens OX=9606 GN=DIEXF PE=1 SV=2	0,950240408	1,91528
P16402	Histone H1.3 OS=Homo sapiens OX=9606 GN=HIST1H1D PE=1 SV=2	-0,25453765	1,915268
Q9C0C4	Semaphorin-4C OS=Homo sapiens OX=9606 GN=SEMA4C PE=1 SV=2	-0,95003711	1,915006
Q68D10	Protein SPT2 homolog OS=Homo sapiens OX=9606 GN=SPTY2D1 PE=1 SV=3	0,393498326	1,91472
Q10570	Cleavage and polyadenylation specificity factor subunit 1 OS=Homo sapiens OX=9606 GN=CPSF1 PE=1 SV=2	0,211494533	1,914406
Q13938	Calcyphosin OS=Homo sapiens OX=9606 GN=CAPS PE=1 SV=2	-0,525558423	1,914095
P68104	Elongation factor 1-alpha 1 OS=Homo sapiens OX=9606 GN=EEF1A1 PE=1 SV=1	0,161373519	1,914088
Q02388	Collagen alpha-1(VII) chain OS=Homo sapiens OX=9606 GN=COL7A1 PE=1 SV=2	-0,254180695	1,911943
Q96I99	Succinate--CoA ligase [GDP-forming] subunit beta, mitochondrial OS=Homo sapiens OX=9606 GN=SUCLG2 PE=1 SV=2	-0,161175491	1,911016
Q92520	Protein FAM3C OS=Homo sapiens OX=9606 GN=FAM3C PE=1 SV=1	-0,405302305	1,910673
Q8IZ69	tRNA (uracil-5-)-methyltransferase homolog A OS=Homo sapiens OX=9606 GN=TRMT2A PE=1 SV=2	-0,946766104	1,910588
Q9NP77	RNA polymerase II subunit A C-terminal domain phosphatase SSU72 OS=Homo sapiens OX=9606 GN=SSU72 PE=1 SV=1	0,303720617	1,910059
O15182	Centrin-3 OS=Homo sapiens OX=9606 GN=CETN3 PE=1 SV=2	-0,524401033	1,910045
Q969U7	Proteasome assembly chaperone 2 OS=Homo sapiens OX=9606 GN=PSMG2 PE=1 SV=1	-0,3034555	1,908074
Q9H3F6	BTB/POZ domain-containing adapter for CUL3-mediated RhoA degradation protein 3 OS=Homo sapiens OX=9606 GN=KCT	0,943424778	1,906062
P52565	Rho GDP-dissociation inhibitor 1 OS=Homo sapiens OX=9606 GN=ARHGDIA PE=1 SV=3	-0,18431442	1,906021
A0FGR8	Extended synaptotagmin-2 OS=Homo sapiens OX=9606 GN=ESYT2 PE=1 SV=1	-0,184144486	1,90375
Q9UPT8	Zinc finger CCCH domain-containing protein 4 OS=Homo sapiens OX=9606 GN=ZC3H4 PE=1 SV=3	-0,253241351	1,903202
Q16658	Fascin OS=Homo sapiens OX=9606 GN=FSCN1 PE=1 SV=3	-0,134941011	1,902118
Q8IXM6	Nurim OS=Homo sapiens OX=9606 GN=NRM PE=1 SV=1	0,343588612	1,902009
Q8NBJ4	Golgi membrane protein 1 OS=Homo sapiens OX=9606 GN=GOLM1 PE=1 SV=1	0,222070789	1,901249
Q07866	Kinesin light chain 1 OS=Homo sapiens OX=9606 GN=KLC1 PE=1 SV=2	-0,151704161	1,900242
Q13151	Heterogeneous nuclear ribonucleoprotein A0 OS=Homo sapiens OX=9606 GN=HNRNPA0 PE=1 SV=1	-0,252899582	1,900023
Q8TED1	Probable glutathione peroxidase 8 OS=Homo sapiens OX=9606 GN=GPX8 PE=1 SV=2	0,252735514	1,898497
Q13084	39S ribosomal protein L28, mitochondrial OS=Homo sapiens OX=9606 GN=MRPL28 PE=1 SV=4	-0,402766177	1,897702
P46087	Probable 28S rRNA (cytosine(4447)-C(5))-methyltransferase OS=Homo sapiens OX=9606 GN=NOP2 PE=1 SV=2	-0,209964211	1,896787
Q9NP61	ADP-ribosylation factor GTPase-activating protein 3 OS=Homo sapiens OX=9606 GN=ARFGAP3 PE=1 SV=1	0,520118963	1,895032
Q00973	Beta-1,4 N-acetylgalactosaminyltransferase 1 OS=Homo sapiens OX=9606 GN=B4GALNT1 PE=1 SV=2	-0,519464822	1,892735
O00625	Pirin OS=Homo sapiens OX=9606 GN=PIR PE=1 SV=1	0,273170724	1,890641
P56545	C-terminal-binding protein 2 OS=Homo sapiens OX=9606 GN=CTBP2 PE=1 SV=1	-0,221050633	1,890219
P25788	Proteasome subunit alpha type-3 OS=Homo sapiens OX=9606 GN=PSMA3 PE=1 SV=2	-0,220907001	1,888667

Q9C0B7	Transport and Golgi organization protein 6 homolog OS=Homo sapiens OX=9606 GN=TANGO6 PE=1 SV=2	-0,449718476	1,887828
Q92769	Histone deacetylase 2 OS=Homo sapiens OX=9606 GN=HDAC2 PE=1 SV=2	-0,220711863	1,886559
O94804	Serine/threonine-protein kinase 10 OS=Homo sapiens OX=9606 GN=STK10 PE=1 SV=1	0,234518423	1,886089
Q9H0H5	Rac GTPase-activating protein 1 OS=Homo sapiens OX=9606 GN=RACGAP1 PE=1 SV=1	-0,272436902	1,884456
A3KN83	Protein strawberry notch homolog 1 OS=Homo sapiens OX=9606 GN=SBNO1 PE=1 SV=1	-0,516724955	1,883104
P12109	Collagen alpha-1(VI) chain OS=Homo sapiens OX=9606 GN=COL6A1 PE=1 SV=3	0,198773684	1,882423
P62993	Growth factor receptor-bound protein 2 OS=Homo sapiens OX=9606 GN=GRB2 PE=1 SV=1	0,182463083	1,881326
Q9Y2K3	Myosin-15 OS=Homo sapiens OX=9606 GN=MYH15 PE=1 SV=5	0,924553667	1,880265
Q9UPT9	Ubiquitin carboxyl-terminal hydrolase 22 OS=Homo sapiens OX=9606 GN=USP22 PE=1 SV=2	-0,922769563	1,877805
Q15008	26S proteasome non-ATPase regulatory subunit 6 OS=Homo sapiens OX=9606 GN=PSMD6 PE=1 SV=1	-0,146470677	1,877405
Q0JRZ9	F-BAR domain only protein 2 OS=Homo sapiens OX=9606 GN=FCHO2 PE=1 SV=2	0,271283783	1,874743
P01137	Transforming growth factor beta-1 proprotein OS=Homo sapiens OX=9606 GN=TGFB1 PE=1 SV=2	-0,397875821	1,872671
Q9Y5Z4	Heme-binding protein 2 OS=Homo sapiens OX=9606 GN=HEBP2 PE=1 SV=1	-0,298645806	1,872101
Q02878	60S ribosomal protein L6 OS=Homo sapiens OX=9606 GN=RPL6 PE=1 SV=3	-0,149956475	1,871587
Q5JSH3	WD repeat-containing protein 44 OS=Homo sapiens OX=9606 GN=WDR44 PE=1 SV=1	0,433608397	1,871475
Q10471	Polypeptide N-acetylgalactosaminyltransferase 2 OS=Homo sapiens OX=9606 GN=GALNT2 PE=1 SV=1	-0,146080506	1,870843
P22061	Protein-L-isoaspartate(D-aspartate) O-methyltransferase OS=Homo sapiens OX=9606 GN=PCMT1 PE=1 SV=4	-0,181577539	1,869548
Q01804	OTU domain-containing protein 4 OS=Homo sapiens OX=9606 GN=OTUD4 PE=1 SV=4	-0,270643093	1,869351
P17535	Transcription factor jun-D OS=Homo sapiens OX=9606 GN=JUND PE=1 SV=3	0,170254442	1,866673
P43307	Translocon-associated protein subunit alpha OS=Homo sapiens OX=9606 GN=SSR1 PE=1 SV=3	0,336527444	1,866496
O15056	Synaptojanin-2 OS=Homo sapiens OX=9606 GN=SYNJ2 PE=1 SV=3	-0,511788964	1,865709
Q9BRT2	Ubiquinol-cytochrome-c reductase complex assembly factor 2 OS=Homo sapiens OX=9606 GN=UQCC2 PE=1 SV=1	0,913947085	1,865586
Q99442	Translocation protein SEC62 OS=Homo sapiens OX=9606 GN=SEC62 PE=1 SV=1	0,336309164	1,865101
Q96GP6	Scavenger receptor class F member 2 OS=Homo sapiens OX=9606 GN=SCARF2 PE=1 SV=5	-0,396384384	1,865032
Q8IYU8	Calcium uptake protein 2, mitochondrial OS=Homo sapiens OX=9606 GN=MICU2 PE=1 SV=2	-0,511583477	1,864984
Q99436	Proteasome subunit beta type-7 OS=Homo sapiens OX=9606 GN=PSMB7 PE=1 SV=1	-0,218362331	1,86123
Q9Y4A5	Transformation/transcription domain-associated protein OS=Homo sapiens OX=9606 GN=TRRAP PE=1 SV=3	-0,196704351	1,857392
Q8TDB6	E3 ubiquitin-protein ligase DTX3L OS=Homo sapiens OX=9606 GN=DTX3L PE=1 SV=1	-0,231633164	1,857089
Q9UBV2	Protein sel-1 homolog 1 OS=Homo sapiens OX=9606 GN=SEL1L PE=1 SV=3	0,33504239	1,857004
Q00169	Phosphatidylinositol transfer protein alpha isoform OS=Homo sapiens OX=9606 GN=PITPNA PE=1 SV=2	-0,231431005	1,855061
Q12882	Dihydropyrimidine dehydrogenase [NADP(+)] OS=Homo sapiens OX=9606 GN=DPYD PE=1 SV=2	-0,508310877	1,853419
Q13576	Ras GTPase-activating-like protein IQGAP2 OS=Homo sapiens OX=9606 GN=IQGAP2 PE=1 SV=4	-0,393510072	1,850303
O00330	Pyruvate dehydrogenase protein X component, mitochondrial OS=Homo sapiens OX=9606 GN=PDHX PE=1 SV=3	-0,506982392	1,848718
Q15334	Lethal(2) giant larvae protein homolog 1 OS=Homo sapiens OX=9606 GN=LLGL1 PE=1 SV=3	-0,247329095	1,848374
Q99575	Ribonucleases P/MRP protein subunit POP1 OS=Homo sapiens OX=9606 GN=POP1 PE=1 SV=2	-0,247156788	1,846781
Q9BYW2	Histone-lysine N-methyltransferase SETD2 OS=Homo sapiens OX=9606 GN=SETD2 PE=1 SV=3	-0,310174041	1,84558
Q9Y3B9	RRP15-like protein OS=Homo sapiens OX=9606 GN=RRP15 PE=1 SV=2	-0,899428705	1,845279
O43639	Cytoplasmic protein NCK2 OS=Homo sapiens OX=9606 GN=NCK2 PE=1 SV=2	0,343465927	1,844487
O15379	Histone deacetylase 3 OS=Homo sapiens OX=9606 GN=HDAC3 PE=1 SV=2	1,230408478	1,842139
Q6NSJ5	Volume-regulated anion channel subunit LRRC8E OS=Homo sapiens OX=9606 GN=LRRC8E PE=1 SV=2	0,332336328	1,839715
Q7Z4Q2	HEAT repeat-containing protein 3 OS=Homo sapiens OX=9606 GN=HEATR3 PE=1 SV=2	-0,246278174	1,838664
Q96K17	Transcription factor BTF3 homolog 4 OS=Homo sapiens OX=9606 GN=BTF3L4 PE=1 SV=1	-0,294138696	1,838471
Q8NCA5	Protein FAM98A OS=Homo sapiens OX=9606 GN=FAM98A PE=1 SV=2	-0,216203311	1,838033
Q96EL3	39S ribosomal protein L53, mitochondrial OS=Homo sapiens OX=9606 GN=MRPL53 PE=1 SV=1	-0,893159302	1,836432
Q9Y2H0	Disks large-associated protein 4 OS=Homo sapiens OX=9606 GN=DLGAP4 PE=1 SV=3	-0,194949558	1,83624
Q99584	Protein S100-A13 OS=Homo sapiens OX=9606 GN=S100A13 PE=1 SV=1	-0,502886746	1,834198
Q9C0E8	Endoplasmic reticulum junction formation protein lunapark OS=Homo sapiens OX=9606 GN=LNPK PE=1 SV=2	-0,266351445	1,8333
Q9UKV8	Protein argonaute-2 OS=Homo sapiens OX=9606 GN=AGO2 PE=1 SV=3	0,389921743	1,831904
Q9BZL1	Ubiquitin-like protein 5 OS=Homo sapiens OX=9606 GN=UBL5 PE=1 SV=1	-0,330928851	1,830726
Q9NVR2	Integrator complex subunit 10 OS=Homo sapiens OX=9606 GN=INTS10 PE=1 SV=2	0,888361076	1,82963
Q01433	AMP deaminase 2 OS=Homo sapiens OX=9606 GN=AMPD2 PE=1 SV=2	0,194361068	1,829162
Q9ULT8	E3 ubiquitin-protein ligase HECTD1 OS=Homo sapiens OX=9606 GN=HECTD1 PE=1 SV=3	0,123333255	1,826579
Q15678	Tyrosine-protein phosphatase non-receptor type 14 OS=Homo sapiens OX=9606 GN=PTPN14 PE=1 SV=2	-0,20361589	1,824171
P53396	ATP-citrate synthase OS=Homo sapiens OX=9606 GN=ACLY PE=1 SV=3	-0,092305102	1,823897
O15523	ATP-dependent RNA helicase DDX3Y OS=Homo sapiens OX=9606 GN=DDX3Y PE=1 SV=2	0,388234342	1,823247
Q8NFH3	Nucleoporin Nup43 OS=Homo sapiens OX=9606 GN=NUP43 PE=1 SV=1	0,388122159	1,822672
P62269	40S ribosomal protein S18 OS=Homo sapiens OX=9606 GN=RPS18 PE=1 SV=3	-0,214678137	1,821691
P15374	Ubiquitin carboxyl-terminal hydrolase isozyme L3 OS=Homo sapiens OX=9606 GN=UCHL3 PE=1 SV=1	-0,387744674	1,820735
Q9P2R7	Succinate--CoA ligase [ADP-forming] subunit beta, mitochondrial OS=Homo sapiens OX=9606 GN=SUCLA2 PE=1 SV=3	0,203254814	1,820064
A6NDU8	UPF0600 protein C5orf51 OS=Homo sapiens OX=9606 GN=C5orf51 PE=1 SV=1	-0,264583232	1,818484
P32780	General transcription factor IIH subunit 1 OS=Homo sapiens OX=9606 GN=GTF2H1 PE=1 SV=1	-0,498384366	1,818194
P11233	Ras-related protein Ral-A OS=Homo sapiens OX=9606 GN=RALA PE=1 SV=1	-0,243998226	1,817636
Q9NXH8	Torsin-4A OS=Homo sapiens OX=9606 GN=TOR4A PE=1 SV=2	-0,227656489	1,817294
P35611	Alpha-adducin OS=Homo sapiens OX=9606 GN=ADD1 PE=1 SV=2	-0,127699011	1,817224
P09525	Annexin A4 OS=Homo sapiens OX=9606 GN=ANXA4 PE=1 SV=4	-0,133105435	1,81611
Q5VSL9	Striatin-interacting protein 1 OS=Homo sapiens OX=9606 GN=STRIP1 PE=1 SV=1	-0,328551517	1,815549
Q96CW1	AP-2 complex subunit mu OS=Homo sapiens OX=9606 GN=AP2M1 PE=1 SV=2	-0,13908314	1,811705
Q14139	Ubiquitin conjugation factor E4 A OS=Homo sapiens OX=9606 GN=UBE4A PE=1 SV=2	-0,496317492	1,810832
O76080	AN1-type zinc finger protein 5 OS=Homo sapiens OX=9606 GN=ZFAND5 PE=1 SV=1	-0,495758641	1,80884
Q15345	Leucine-rich repeat-containing protein 41 OS=Homo sapiens OX=9606 GN=LRRC41 PE=1 SV=3	0,385403714	1,80872
P82930	28S ribosomal protein S34, mitochondrial OS=Homo sapiens OX=9606 GN=MRPS34 PE=1 SV=2	-0,494025073	1,802656
Q8IYB7	DIS3-like exonuclease 2 OS=Homo sapiens OX=9606 GN=DIS3L2 PE=1 SV=4	0,493530104	1,800889
P16383	GC-rich sequence DNA-binding factor 2 OS=Homo sapiens OX=9606 GN=GCFC2 PE=1 SV=2	-0,867941653	1,800365
Q15366	Poly(rC)-binding protein 2 OS=Homo sapiens OX=9606 GN=PCBP2 PE=1 SV=1	-0,191923336	1,799927
P41440	Folate transporter 1 OS=Homo sapiens OX=9606 GN=SLC19A1 PE=1 SV=3	-0,867576827	1,799837
Q15382	GTP-binding protein Rheb OS=Homo sapiens OX=9606 GN=RHEB PE=1 SV=1	-0,225818553	1,798972
Q9HD26	Golgi-associated PDZ and coiled-coil motif-containing protein OS=Homo sapiens OX=9606 GN=GOPC PE=1 SV=1	0,288799357	1,798735
Q7Z7G8	Vacuolar protein sorting-associated protein 13B OS=Homo sapiens OX=9606 GN=VPS13B PE=1 SV=2	0,400235012	1,798472
Q8NF37	Lysophosphatidylcholine acyltransferase 1 OS=Homo sapiens OX=9606 GN=LPCAT1 PE=1 SV=2	0,3257974	1,797977
P09936	Ubiquitin carboxyl-terminal hydrolase isozyme L1 OS=Homo sapiens OX=9606 GN=UCHL1 PE=1 SV=2	0,176050707	1,796556
Q12996	Cleavage stimulation factor subunit 3 OS=Homo sapiens OX=9606 GN=CSTF3 PE=1 SV=1	-0,241695358	1,796449
Q8N5C6	S1 RNA-binding domain-containing protein 1 OS=Homo sapiens OX=9606 GN=SRBD1 PE=1 SV=2	-0,382990939	1,796332
P14678	Small nuclear ribonucleoprotein-associated proteins B and B' OS=Homo sapiens OX=9606 GN=SNRPB PE=1 SV=2	-0,225498903	1,79579
Q8NHH9	Atlastin-2 OS=Homo sapiens OX=9606 GN=ATL2 PE=1 SV=2	-0,325382092	1,795328
Q96EB1	Elongator complex protein 4 OS=Homo sapiens OX=9606 GN=ELP4 PE=1 SV=2	-0,864171825	1,794905
P62258	14-3-3 protein epsilon OS=Homo sapiens OX=9606 GN=YWHAE PE=1 SV=1	-0,141510082	1,794567
O43286	Beta-1,4-galactosyltransferase 5 OS=Homo sapiens OX=9606 GN=B4GALT5 PE=1 SV=1	-0,268616635	1,793714
Q96EK6	Glucosamine 6-phosphate N-acetyltransferase OS=Homo sapiens OX=9606 GN=GNPNAT1 PE=1 SV=1	-0,491013018	1,791895
Q9Y5Q0	Fatty acid desaturase 3 OS=Homo sapiens OX=9606 GN=FADS3 PE=1 SV=1	0,381860134	1,790524
O75663	TIP41-like protein OS=Homo sapiens OX=9606 GN=TIPRL PE=1 SV=2	0,200637311	1,79037
Q13158	FAS-associated death domain protein OS=Homo sapiens OX=9606 GN=FADD PE=1 SV=1	-0,3245048	1,789733

Q14344	Guanine nucleotide-binding protein subunit alpha-13 OS=Homo sapiens OX=9606 GN=GNA13 PE=1 SV=2	0,224889983	1,789733
Q96HS1	Serine/threonine-protein phosphatase PGAM5, mitochondrial OS=Homo sapiens OX=9606 GN=PGAM5 PE=1 SV=2	0,260804502	1,786894
Q96RT1	Erbin OS=Homo sapiens OX=9606 GN=ERBIN PE=1 SV=2	-0,144707803	1,786421
Q9NRW7	Vacuolar protein sorting-associated protein 45 OS=Homo sapiens OX=9606 GN=VPS45 PE=1 SV=1	-0,153049068	1,78638
P82675	28S ribosomal protein S5, mitochondrial OS=Homo sapiens OX=9606 GN=MRPS5 PE=1 SV=2	-0,48945617	1,786325
Q4L180	Filamin A-interacting protein 1-like OS=Homo sapiens OX=9606 GN=FILIP1L PE=1 SV=2	0,323925547	1,78604
P07196	Neurofilament light polypeptide OS=Homo sapiens OX=9606 GN=NEFL PE=1 SV=3	-0,152872007	1,783696
Q8WW12	PEST proteolytic signal-containing nuclear protein OS=Homo sapiens OX=9606 GN=PCNP PE=1 SV=2	-0,240270845	1,78337
Q86W50	RNA N6-adenosine-methyltransferase METTL16 OS=Homo sapiens OX=9606 GN=METTL16 PE=1 SV=2	-0,380250885	1,782257
P50213	Isocitrate dehydrogenase [NAD] subunit alpha, mitochondrial OS=Homo sapiens OX=9606 GN=IDH3A PE=1 SV=1	-0,199587803	1,778501
Q96F07	Cytoplasmic FMR1-interacting protein 2 OS=Homo sapiens OX=9606 GN=CYFIP2 PE=1 SV=2	-0,162402748	1,777471
Q9HCU5	Prolactin regulatory element-binding protein OS=Homo sapiens OX=9606 GN=PREB PE=1 SV=2	-0,259656899	1,777321
Q9BQ52	Zinc phosphodiesterase ELAC protein 2 OS=Homo sapiens OX=9606 GN=ELAC2 PE=1 SV=2	-0,137053946	1,777199
Q969N2	GPI transamidase component PIG-T OS=Homo sapiens OX=9606 GN=PIGT PE=1 SV=1	0,322175449	1,774884
P78314	SH3 domain-binding protein 2 OS=Homo sapiens OX=9606 GN=SH3BP2 PE=1 SV=2	1,828897807	1,774581
Q9UKI8	Serine/threonine-protein kinase tousled-like 1 OS=Homo sapiens OX=9606 GN=TLK1 PE=1 SV=2	-0,718602132	1,773347
Q8N335	Glycerol-3-phosphate dehydrogenase 1-like protein OS=Homo sapiens OX=9606 GN=GPD1L PE=1 SV=1	0,378099661	1,771203
Q92733	Proline-rich protein PRCC OS=Homo sapiens OX=9606 GN=PRCC PE=1 SV=1	0,485060165	1,77057
P40926	Malate dehydrogenase, mitochondrial OS=Homo sapiens OX=9606 GN=MDH2 PE=1 SV=3	0,156720994	1,770518
O43181	NADH dehydrogenase [ubiquinone] iron-sulfur protein 4, mitochondrial OS=Homo sapiens OX=9606 GN=NDUFS4 PE=1 SV	-0,377738968	1,76935
Q9Y2G3	Probable phospholipid-transporting ATPase IF OS=Homo sapiens OX=9606 GN=ATP11B PE=1 SV=2	0,132380264	1,768807
Q86W42	THO complex subunit 6 homolog OS=Homo sapiens OX=9606 GN=THOC6 PE=1 SV=1	0,845663161	1,767841
Q8TDX7	Serine/threonine-protein kinase Nek7 OS=Homo sapiens OX=9606 GN=NEK7 PE=1 SV=1	-0,161629736	1,766543
P20290	Transcription factor BTF3 OS=Homo sapiens OX=9606 GN=BTF3 PE=1 SV=1	0,222346413	1,764482
Q9P013	Spliceosome-associated protein CWC15 homolog OS=Homo sapiens OX=9606 GN=CWC15 PE=1 SV=2	-0,843233025	1,764255
P48454	Serine/threonine-protein phosphatase 2B catalytic subunit gamma isoform OS=Homo sapiens OX=9606 GN=PPP3CC PE=1	0,933264771	1,763988
Q13601	KRR1 small subunit processome component homolog OS=Homo sapiens OX=9606 GN=KRR1 PE=1 SV=4	-0,37653886	1,763181
Q9ULS6	Potassium voltage-gated channel subfamily S member 2 OS=Homo sapiens OX=9606 GN=KCNS2 PE=1 SV=2	0,842312855	1,762895
O43805	Sjoegren syndrome nuclear autoantigen 1 OS=Homo sapiens OX=9606 GN=SSNA1 PE=1 SV=2	-0,320231134	1,762497
Q8WXI9	Transcriptional repressor p66-beta OS=Homo sapiens OX=9606 GN=GATAD2B PE=1 SV=1	-0,198143707	1,762206
O14733	Dual specificity mitogen-activated protein kinase kinase 7 OS=Homo sapiens OX=9606 GN=MAP2K7 PE=1 SV=2	0,405147763	1,762019
P16989	Y-box-binding protein 3 OS=Homo sapiens OX=9606 GN=YBX3 PE=1 SV=4	-0,188695934	1,76143
P11277	Spectrin beta chain, erythrocytic OS=Homo sapiens OX=9606 GN=SPTB PE=1 SV=5	0,283714618	1,761006
Q9UGR2	Zinc finger CCCH domain-containing protein 7B OS=Homo sapiens OX=9606 GN=ZC3H7B PE=1 SV=2	-0,283714245	1,761003
Q9NZN8	CCR4-NOT transcription complex subunit 2 OS=Homo sapiens OX=9606 GN=CNOT2 PE=1 SV=1	-0,482076554	1,759852
Q8IY18	Structural maintenance of chromosomes protein 5 OS=Homo sapiens OX=9606 GN=SMC5 PE=1 SV=2	0,838315041	1,756974
Q93050	V-type proton ATPase 116 kDa subunit a isoform 1 OS=Homo sapiens OX=9606 GN=ATP6V0A1 PE=1 SV=3	-0,481069215	1,75623
Q9BX68	Histidine triad nucleotide-binding protein 2, mitochondrial OS=Homo sapiens OX=9606 GN=HINT2 PE=1 SV=1	-0,318894173	1,753982
P49006	MARCKS-related protein OS=Homo sapiens OX=9606 GN=MARCKSL1 PE=1 SV=2	-0,374682429	1,753637
Q9H0S4	Probable ATP-dependent RNA helicase DDX47 OS=Homo sapiens OX=9606 GN=DDX47 PE=1 SV=1	0,221125608	1,752394
Q8N5V2	Ephexin-1 OS=Homo sapiens OX=9606 GN=NGEF PE=1 SV=2	-0,221083653	1,751979
Q9BWS9	Chitinase domain-containing protein 1 OS=Homo sapiens OX=9606 GN=CHID1 PE=1 SV=1	0,374336947	1,751861
Q9Y2Q9	28S ribosomal protein S28, mitochondrial OS=Homo sapiens OX=9606 GN=MRPS28 PE=1 SV=1	-0,479738835	1,751442
Q8TDD1	ATP-dependent RNA helicase DDX54 OS=Homo sapiens OX=9606 GN=DDX54 PE=1 SV=2	-0,282184944	1,749678
P11234	Ras-related protein Ral-B OS=Homo sapiens OX=9606 GN=RALB PE=1 SV=1	0,318051105	1,748614
P49821	NADH dehydrogenase [ubiquinone] flavoprotein 1, mitochondrial OS=Homo sapiens OX=9606 GN=NDUFV1 PE=1 SV=4	-0,207717353	1,747593
P53621	Coatomer subunit alpha OS=Homo sapiens OX=9606 GN=COPA PE=1 SV=2	-0,083460243	1,746703
Q9H9Q2	COP9 signalosome complex subunit 7b OS=Homo sapiens OX=9606 GN=COPS7B PE=1 SV=1	-0,281663039	1,745815
Q5TCZ1	SH3 and PX domain-containing protein 2A OS=Homo sapiens OX=9606 GN=SH3PXD2A PE=1 SV=1	-0,830774214	1,745749
O15427	Monocarboxylate transporter 4 OS=Homo sapiens OX=9606 GN=SLC16A3 PE=1 SV=1	-0,281128994	1,741864
Q15043	Zinc transporter ZIP14 OS=Homo sapiens OX=9606 GN=SLC39A14 PE=1 SV=3	0,372253459	1,741146
Q5T200	Zinc finger CCCH domain-containing protein 13 OS=Homo sapiens OX=9606 GN=ZC3H13 PE=1 SV=1	-0,371978799	1,739733
Q07954	Prolow-density lipoprotein receptor-related protein 1 OS=Homo sapiens OX=9606 GN=LRP1 PE=1 SV=2	-0,064647953	1,738769
O15231	Zinc finger protein 185 OS=Homo sapiens OX=9606 GN=ZNF185 PE=1 SV=3	0,316265221	1,737248
Q9NS87	Kinesin-like protein KIF15 OS=Homo sapiens OX=9606 GN=KIF15 PE=1 SV=1	-0,475365724	1,735677
Q9P258	Protein RCC2 OS=Homo sapiens OX=9606 GN=RCC2 PE=1 SV=2	0,149608718	1,734456
Q86US8	Telomerase-binding protein EST1A OS=Homo sapiens OX=9606 GN=SMG6 PE=1 SV=2	0,823002062	1,734101
Q14137	Ribosome biogenesis protein BOP1 OS=Homo sapiens OX=9606 GN=BOP1 PE=1 SV=2	-0,370853932	1,733947
Q9BVI4	Nucleolar complex protein 4 homolog OS=Homo sapiens OX=9606 GN=NOC4L PE=1 SV=1	-0,219130774	1,732685
O75787	Renin receptor OS=Homo sapiens OX=9606 GN=ATP6AP2 PE=1 SV=2	0,254282323	1,732614
Q9H0R4	Haloacid dehalogenase-like hydrolase domain-containing protein 2 OS=Homo sapiens OX=9606 GN=HDHD2 PE=1 SV=1	-0,821667196	1,732093
Q9H1E3	Nuclear ubiquitous casein and cyclin-dependent kinase substrate 1 OS=Homo sapiens OX=9606 GN=NUCKS1 PE=1 SV=1	-0,473954668	1,730582
P46976	Glycogenin-1 OS=Homo sapiens OX=9606 GN=GYG1 PE=1 SV=4	0,253872736	1,729215
Q01831	DNA repair protein complementing XP-C cells OS=Homo sapiens OX=9606 GN=XPC PE=1 SV=4	-0,473262504	1,728081
Q99523	Sortilin OS=Homo sapiens OX=9606 GN=SORT1 PE=1 SV=3	-0,314675012	1,727132
Q96ST3	Paired amphipathic helix protein Sin3a OS=Homo sapiens OX=9606 GN=SIN3A PE=1 SV=2	-0,253353242	1,724907
P49770	Translation initiation factor eIF-2B subunit beta OS=Homo sapiens OX=9606 GN=EIF2B2 PE=1 SV=3	0,314320927	1,72488
Q8NBK3	Formylglycine-generating enzyme OS=Homo sapiens OX=9606 GN=SUMF1 PE=1 SV=3	-0,749551479	1,722226
Q9HCM4	Band 4.1-like protein 5 OS=Homo sapiens OX=9606 GN=EPB41L5 PE=1 SV=3	0,471490588	1,721673
Q5T3I0	G patch domain-containing protein 4 OS=Homo sapiens OX=9606 GN=GPATCH4 PE=1 SV=2	-0,814705099	1,721578
Q9Y639	Neuroplastin OS=Homo sapiens OX=9606 GN=NPTN PE=1 SV=2	0,313658178	1,720666
Q6VN20	Ran-binding protein 10 OS=Homo sapiens OX=9606 GN=RANBP10 PE=1 SV=1	0,36818081	1,720194
Q9UBJ2	ATP-binding cassette sub-family D member 2 OS=Homo sapiens OX=9606 GN=ABCD2 PE=1 SV=1	1,230379434	1,719559
Q8N5K1	CDGSH iron-sulfur domain-containing protein 2 OS=Homo sapiens OX=9606 GN=CISD2 PE=1 SV=1	0,470894848	1,719518
Q5JSL3	Dedicator of cytokinesis protein 11 OS=Homo sapiens OX=9606 GN=DOCK11 PE=1 SV=2	0,888463548	1,718862
Q9HC35	Echinoderm microtubule-associated protein-like 4 OS=Homo sapiens OX=9606 GN=EML4 PE=1 SV=3	-0,194232482	1,718281
Q8N163	Cell cycle and apoptosis regulator protein 2 OS=Homo sapiens OX=9606 GN=CCAR2 PE=1 SV=2	-0,124889594	1,718157
Q9UBU9	Nuclear RNA export factor 1 OS=Homo sapiens OX=9606 GN=NXF1 PE=1 SV=1	0,20469036	1,715623
O43294	Transforming growth factor beta-1-induced transcript 1 protein OS=Homo sapiens OX=9606 GN=TGFB1I1 PE=1 SV=2	-0,277342495	1,713886
O43752	Syntaxin-6 OS=Homo sapiens OX=9606 GN=STX6 PE=1 SV=1	0,36690636	1,713635
O60488	Long-chain-fatty-acid--CoA ligase 4 OS=Homo sapiens OX=9606 GN=ACSL4 PE=1 SV=2	0,157819515	1,712998
P14543	Nidogen-1 OS=Homo sapiens OX=9606 GN=NID1 PE=1 SV=3	0,808257969	1,711784
Q8WUI4	Histone deacetylase 7 OS=Homo sapiens OX=9606 GN=HDAC7 PE=1 SV=2	-0,312242453	1,711666
P78406	mRNA export factor OS=Homo sapiens OX=9606 GN=RAE1 PE=1 SV=1	-0,193499716	1,710086
O94903	Pyridoxal phosphate homeostasis protein OS=Homo sapiens OX=9606 GN=PLPBP PE=1 SV=1	0,366187752	1,709937
Q99829	Copine-1 OS=Homo sapiens OX=9606 GN=CPNE1 PE=1 SV=1	0,157532978	1,708993
Q96JH8	Ras-associating and dilute domain-containing protein OS=Homo sapiens OX=9606 GN=RADIL PE=1 SV=5	0,522099811	1,708799
O43615	Mitochondrial import inner membrane translocase subunit TIM44 OS=Homo sapiens OX=9606 GN=TIMM44 PE=1 SV=2	0,184137691	1,70747
Q9UGL1	#N/D	-0,678356373	1,706712
P08708	40S ribosomal protein S17 OS=Homo sapiens OX=9606 GN=RPS17 PE=1 SV=2	-0,231843355	1,706429
Q9BZE1	39S ribosomal protein L37, mitochondrial OS=Homo sapiens OX=9606 GN=MRPL37 PE=1 SV=2	-0,216319673	1,705005

P05114	Non-histone chromosomal protein HMG-14 OS=Homo sapiens OX=9606 GN=HMGN1 PE=1 SV=3	-0,250853408	1,704204
Q86SF2	N-acetylgalactosaminyltransferase 7 OS=Homo sapiens OX=9606 GN=GALNT7 PE=1 SV=1	-0,25078721	1,703657
Q9H7L9	Sin3 histone deacetylase corepressor complex component SDS3 OS=Homo sapiens OX=9606 GN=SUDS3 PE=1 SV=2	0,135730322	1,703017
Q9UNS1	Protein timeless homolog OS=Homo sapiens OX=9606 GN=TIMELESS PE=1 SV=2	-0,466310178	1,702904
O14879	Interferon-induced protein with tetratricopeptide repeats 3 OS=Homo sapiens OX=9606 GN=IFIT3 PE=1 SV=1	0,425364853	1,700921
Q9NZL4	Hsp70-binding protein 1 OS=Homo sapiens OX=9606 GN=HSPBP1 PE=1 SV=2	0,156869572	1,699731
Q7L5Y9	E3 ubiquitin-protein transferase MAEA OS=Homo sapiens OX=9606 GN=MAEA PE=1 SV=1	-0,364007081	1,698712
Q96PD2	Discoidin, CUB and LCCL domain-containing protein 2 OS=Homo sapiens OX=9606 GN=DCBLD2 PE=1 SV=1	-0,151771153	1,698559
Q9H0P0	Cytosolic 5'-nucleotidase 3A OS=Homo sapiens OX=9606 GN=NT5C3A PE=1 SV=3	0,465103673	1,698524
P14314	Glucosidase 2 subunit beta OS=Homo sapiens OX=9606 GN=PRKCSH PE=1 SV=2	-0,142982145	1,697689
P42574	Caspase-3 OS=Homo sapiens OX=9606 GN=CASP3 PE=1 SV=2	-0,275090696	1,697279
Q13506	NGFI-A-binding protein 1 OS=Homo sapiens OX=9606 GN=NAB1 PE=1 SV=2	0,798697359	1,697155
Q9UBC2	Epidermal growth factor receptor substrate 15-like 1 OS=Homo sapiens OX=9606 GN=EPS15L1 PE=1 SV=1	-0,126294274	1,6955
P0DP25	Calmodulin-3 OS=Homo sapiens OX=9606 GN=CALM3 PE=1 SV=1	-0,182843131	1,692233
Q93100	Phosphorylase b kinase regulatory subunit beta OS=Homo sapiens OX=9606 GN=PHKB PE=1 SV=3	-0,462544796	1,689226
Q9UI09	NADH dehydrogenase [ubiquinone] 1 alpha subcomplex subunit 12 OS=Homo sapiens OX=9606 GN=NDUFA12 PE=1 SV=1	-0,792611768	1,687777
Q93074	Mediator of RNA polymerase II transcription subunit 12 OS=Homo sapiens OX=9606 GN=MED12 PE=1 SV=4	-0,461955156	1,687081
Q9Y6A4	Cilia- and flagella-associated protein 20 OS=Homo sapiens OX=9606 GN=CFAP20 PE=1 SV=1	-0,361547488	1,686049
Q9Y4E8	Ubiquitin carboxyl-terminal hydrolase 15 OS=Homo sapiens OX=9606 GN=USP15 PE=1 SV=3	0,201497119	1,682067
Q9NR46	Endophilin-B2 OS=Homo sapiens OX=9606 GN=SH3GLB2 PE=1 SV=1	-0,173779928	1,677628
Q9BQE5	Apolipoprotein L2 OS=Homo sapiens OX=9606 GN=APOL2 PE=1 SV=1	-0,228664826	1,677606
Q9HAS0	Protein Njmu-R1 OS=Homo sapiens OX=9606 GN=C17orf75 PE=1 SV=2	-0,306847114	1,677404
P32969	60S ribosomal protein L9 OS=Homo sapiens OX=9606 GN=RPL9 PE=1 SV=1	-0,213445188	1,676814
P19367	Hexokinase-1 OS=Homo sapiens OX=9606 GN=HK1 PE=1 SV=3	-0,092600976	1,676511
Q9Y2R9	28S ribosomal protein S7, mitochondrial OS=Homo sapiens OX=9606 GN=MRPS7 PE=1 SV=2	0,247481131	1,676353
Q16576	Histone-binding protein RBBP7 OS=Homo sapiens OX=9606 GN=RBBP7 PE=1 SV=1	0,160529904	1,674367
O43432	Eukaryotic translation initiation factor 4 gamma 3 OS=Homo sapiens OX=9606 GN=EIF4G3 PE=1 SV=2	0,190175206	1,673042
O60762	Dolichol-phosphate mannosyltransferase subunit 1 OS=Homo sapiens OX=9606 GN=DPM1 PE=1 SV=1	0,228142452	1,67288
P04080	Cystatin-B OS=Homo sapiens OX=9606 GN=CSTB PE=1 SV=2	0,306028306	1,67221
Q9Y2B0	Protein canopy homolog 2 OS=Homo sapiens OX=9606 GN=CNPY2 PE=1 SV=1	0,227987063	1,671475
Q8IZU2	WD repeat-containing protein 17 OS=Homo sapiens OX=9606 GN=WDR17 PE=2 SV=2	0,781908839	1,67116
O60220	Mitochondrial import inner membrane translocase subunit Tim8 A OS=Homo sapiens OX=9606 GN=TIMM8A PE=1 SV=1	0,780259267	1,668585
Q99590	Protein SCAF11 OS=Homo sapiens OX=9606 GN=SCAF11 PE=1 SV=2	0,35784192	1,666965
Q7L5N1	COP9 signalosome complex subunit 6 OS=Homo sapiens OX=9606 GN=COPS6 PE=1 SV=1	-0,200031961	1,666729
Q02241	Kinesin-like protein KIF23 OS=Homo sapiens OX=9606 GN=KIF23 PE=1 SV=3	0,227408546	1,666245
Q96AB3	Isochorismatase domain-containing protein 2 OS=Homo sapiens OX=9606 GN=ISOC2 PE=1 SV=1	-0,304589728	1,663087
Q9H6U6	Breast carcinoma-amplified sequence 3 OS=Homo sapiens OX=9606 GN=BCAS3 PE=1 SV=3	0,774888389	1,660173
Q9UPN7	Serine/threonine-protein phosphatase 6 regulatory subunit 1 OS=Homo sapiens OX=9606 GN=PPP6R1 PE=1 SV=5	-0,172181635	1,658
O00443	Phosphatidylinositol 4-phosphate 3-kinase C2 domain-containing subunit alpha OS=Homo sapiens OX=9606 GN=PIK3C2A	-0,172122031	1,657269
O43747	AP-1 complex subunit gamma-1 OS=Homo sapiens OX=9606 GN=AP1G1 PE=1 SV=5	-0,121436616	1,656488
Q15233	Non-POU domain-containing octamer-binding protein OS=Homo sapiens OX=9606 GN=NONO PE=1 SV=4	-0,133058722	1,656401
Q7KZ85	Transcription elongation factor SPT6 OS=Homo sapiens OX=9606 GN=SUPT6H PE=1 SV=2	0,106747472	1,654962
Q86TI2	Dipeptidyl peptidase 9 OS=Homo sapiens OX=9606 GN=DPP9 PE=1 SV=3	-0,126753012	1,654815
Q9Y3C0	WASH complex subunit 3 OS=Homo sapiens OX=9606 GN=WASHC3 PE=1 SV=1	0,303248988	1,654588
Q00534	Cyclin-dependent kinase 6 OS=Homo sapiens OX=9606 GN=CDK6 PE=1 SV=1	-0,158984381	1,653736
Q13867	Bleomycin hydrolase OS=Homo sapiens OX=9606 GN=BLMH PE=1 SV=1	0,1441241	1,652727
Q8IYD1	Eukaryotic peptide chain release factor GTP-binding subunit ERF3B OS=Homo sapiens OX=9606 GN=GSPT2 PE=1 SV=2	-0,419875683	1,651647
O43402	ER membrane protein complex subunit 8 OS=Homo sapiens OX=9606 GN=EMC8 PE=1 SV=1	-0,452167858	1,651381
Q9BS26	Endoplasmic reticulum resident protein 44 OS=Homo sapiens OX=9606 GN=ERP44 PE=1 SV=1	0,153370714	1,651148
Q9P0J0	NADH dehydrogenase [ubiquinone] 1 alpha subcomplex subunit 13 OS=Homo sapiens OX=9606 GN=NDUFA13 PE=1 SV=3	-0,354721591	1,650892
O43493	Trans-Golgi network integral membrane protein 2 OS=Homo sapiens OX=9606 GN=TGOLN2 PE=1 SV=3	0,198428352	1,649984
Q15717	ELAV-like protein 1 OS=Homo sapiens OX=9606 GN=ELAVL1 PE=1 SV=2	0,18805416	1,649526
O14964	Hepatocyte growth factor-regulated tyrosine kinase substrate OS=Homo sapiens OX=9606 GN=HGS PE=1 SV=1	-0,143892169	1,6493
Q96EY7	Pentatricopeptide repeat domain-containing protein 3, mitochondrial OS=Homo sapiens OX=9606 GN=PTCD3 PE=1 SV=3	0,210391056	1,646989
Q9BPZ3	Polyadenylate-binding protein-interacting protein 2 OS=Homo sapiens OX=9606 GN=PAIP2 PE=1 SV=1	-0,733562066	1,645932
Q92665	28S ribosomal protein S31, mitochondrial OS=Homo sapiens OX=9606 GN=MRPS31 PE=1 SV=3	-0,763997129	1,642989
P53004	Biliverdin reductase A OS=Homo sapiens OX=9606 GN=BLVRA PE=1 SV=2	-0,139377812	1,64257
Q5T280	Putative methyltransferase C9orf114 OS=Homo sapiens OX=9606 GN=SPOUT1 PE=1 SV=3	-0,763573526	1,642317
O14763	Tumor necrosis factor receptor superfamily member 10B OS=Homo sapiens OX=9606 GN=TNFRSF10B PE=1 SV=2	0,224537959	1,64035
P20929	Nebulin OS=Homo sapiens OX=9606 GN=NEB PE=1 SV=5	0,144413955	1,638528
Q15437	Protein transport protein Sec23B OS=Homo sapiens OX=9606 GN=SEC23B PE=1 SV=2	0,131838072	1,636757
P60842	Eukaryotic initiation factor 4A-I OS=Homo sapiens OX=9606 GN=EIF4A1 PE=1 SV=1	-0,105762911	1,635054
Q96CW5	Gamma-tubulin complex component 3 OS=Homo sapiens OX=9606 GN=TUBGCP3 PE=1 SV=2	-0,351545608	1,634529
P18124	60S ribosomal protein L7 OS=Homo sapiens OX=9606 GN=RPL7 PE=1 SV=1	-0,152149136	1,634292
P37268	Squalene synthase OS=Homo sapiens OX=9606 GN=FDFT1 PE=1 SV=1	-0,208930251	1,63277
Q9BXR0	Queuine tRNA-ribosyltransferase catalytic subunit 1 OS=Homo sapiens OX=9606 GN=QTRT1 PE=1 SV=3	-0,299773652	1,632578
Q9UHB9	Signal recognition particle subunit SRP68 OS=Homo sapiens OX=9606 GN=SRP68 PE=1 SV=2	-0,147149424	1,63225
P52907	F-actin-capping protein subunit alpha-1 OS=Homo sapiens OX=9606 GN=CAPZA1 PE=1 SV=3	0,186469852	1,632022
O60701	UDP-glucose 6-dehydrogenase OS=Homo sapiens OX=9606 GN=UGDH PE=1 SV=1	0,117607138	1,631717
Q92620	Pre-mRNA-splicing factor ATP-dependent RNA helicase PRP16 OS=Homo sapiens OX=9606 GN=DHX38 PE=1 SV=2	0,196492316	1,629828
Q5VTL8	Pre-mRNA-splicing factor 38B OS=Homo sapiens OX=9606 GN=PRPF38B PE=1 SV=1	0,241821847	1,629816
Q9BZ23	#N/D	0,495524	1,628365
P24928	DNA-directed RNA polymerase II subunit RPB1 OS=Homo sapiens OX=9606 GN=POLR2A PE=1 SV=2	-0,162821005	1,6255
Q04760	Lactoylglutathione lyase OS=Homo sapiens OX=9606 GN=GLO1 PE=1 SV=4	-0,208140508	1,625097
P29966	Myristoylated alanine-rich C-kinase substrate OS=Homo sapiens OX=9606 GN=MARCKS PE=1 SV=4	0,169299772	1,622782
Q9ULC4	Malignant T-cell-amplified sequence 1 OS=Homo sapiens OX=9606 GN=MCTS1 PE=1 SV=1	0,222580077	1,622742
Q9HBK9	Arsenite methyltransferase OS=Homo sapiens OX=9606 GN=AS3MT PE=1 SV=3	-0,168901208	1,61793
Q96QC0	Serine/threonine-protein phosphatase 1 regulatory subunit 10 OS=Homo sapiens OX=9606 GN=PPP1R10 PE=1 SV=1	-0,264253846	1,617707
Q9H2M9	Rab3 GTPase-activating protein non-catalytic subunit OS=Homo sapiens OX=9606 GN=RAB3GAP2 PE=1 SV=1	0,119231099	1,61751
Q7Z7K0	COX assembly mitochondrial protein homolog OS=Homo sapiens OX=9606 GN=CMC1 PE=1 SV=1	-0,442684122	1,616605
Q6NZ67	Mitotic-spindle organizing protein 2B OS=Homo sapiens OX=9606 GN=MZT2B PE=1 SV=1	-0,747457293	1,616564
Q92979	Ribosomal RNA small subunit methyltransferase NEP1 OS=Homo sapiens OX=9606 GN=EMG1 PE=1 SV=4	-0,240200788	1,616533
Q06495	Sodium-dependent phosphate transport protein 2A OS=Homo sapiens OX=9606 GN=SLC34A1 PE=1 SV=1	0,513429938	1,616427
Q9H299	SH3 domain-binding glutamic acid-rich-like protein 3 OS=Homo sapiens OX=9606 GN=SH3BGRL3 PE=1 SV=1	-0,264078328	1,616423
Q6YHK3	CD109 antigen OS=Homo sapiens OX=9606 GN=CD109 PE=1 SV=2	-0,184530579	1,610667
Q9BY43	Charged multivesicular body protein 4a OS=Homo sapiens OX=9606 GN=CHMP4A PE=1 SV=3	0,29621184	1,610048
P35659	Protein DEK OS=Homo sapiens OX=9606 GN=DEK PE=1 SV=1	-0,168249854	1,610008
Q8IVM0	Coiled-coil domain-containing protein 50 OS=Homo sapiens OX=9606 GN=CCDC50 PE=1 SV=1	-0,184465681	1,609953
Q9H6D7	HAUS augmin-like complex subunit 4 OS=Homo sapiens OX=9606 GN=HAUS4 PE=1 SV=1	-0,439934936	1,606491
Q9H7D7	WD repeat-containing protein 26 OS=Homo sapiens OX=9606 GN=WDR26 PE=1 SV=3	-0,220742121	1,606251
P62314	Small nuclear ribonucleoprotein Sm D1 OS=Homo sapiens OX=9606 GN=SNRPD1 PE=1 SV=1	0,345554606	1,603656

Q03001	Dystonin OS=Homo sapiens OX=9606 GN=DST PE=1 SV=4	0,043671108	1,600825
Q07666	KH domain-containing, RNA-binding, signal transduction-associated protein 1 OS=Homo sapiens OX=9606 GN=KHDRBS1 P	-0,205570917	1,60019
Q9UBE0	SUMO-activating enzyme subunit 1 OS=Homo sapiens OX=9606 GN=SAE1 PE=1 SV=1	-0,144860187	1,599723
Q9NY27	Serine/threonine-protein phosphatase 4 regulatory subunit 2 OS=Homo sapiens OX=9606 GN=PPP4R2 PE=1 SV=3	-0,2945727	1,599691
P62244	40S ribosomal protein S15a OS=Homo sapiens OX=9606 GN=RPS15A PE=1 SV=2	-0,238095979	1,59932
O14828	Secretory carrier-associated membrane protein 3 OS=Homo sapiens OX=9606 GN=SCAMP3 PE=1 SV=3	0,219889635	1,598615
Q9H0L4	Cleavage stimulation factor subunit 2 tau variant OS=Homo sapiens OX=9606 GN=CSTF2T PE=1 SV=1	-0,437536524	1,597656
Q8WWM7	Ataxin-2-like protein OS=Homo sapiens OX=9606 GN=ATXN2L PE=1 SV=2	-0,11803571	1,596519
Q8N5A5	Zinc finger CCCH-type with G patch domain-containing protein OS=Homo sapiens OX=9606 GN=ZGPAT PE=1 SV=3	-0,735061888	1,596496
Q9Y5S9	RNA-binding protein 8A OS=Homo sapiens OX=9606 GN=RBM8A PE=1 SV=1	-0,193209256	1,595799
Q96J01	THO complex subunit 3 OS=Homo sapiens OX=9606 GN=THOC3 PE=1 SV=1	-0,261129362	1,594877
P06576	ATP synthase subunit beta, mitochondrial OS=Homo sapiens OX=9606 GN=ATP5F1B PE=1 SV=3	-0,117935861	1,59477
Q9Y2Z4	Tyrosine--tRNA ligase, mitochondrial OS=Homo sapiens OX=9606 GN=YARS2 PE=1 SV=2	-0,343770433	1,594461
Q7Z5K2	Wings apart-like protein homolog OS=Homo sapiens OX=9606 GN=WAPL PE=1 SV=1	0,293618431	1,593664
P17480	Nucleolar transcription factor 1 OS=Homo sapiens OX=9606 GN=UBTF PE=1 SV=1	-0,154325525	1,592033
Q9BT40	Inositol polyphosphate 5-phosphatase K OS=Homo sapiens OX=9606 GN=INPP5K PE=1 SV=3	-0,435655832	1,59072
Q9BV86	N-terminal Xaa-Pro-Lys N-methyltransferase 1 OS=Homo sapiens OX=9606 GN=NTMT1 PE=1 SV=3	0,34288564	1,589901
Q9NYU2	UDP-glucose:glycoprotein glucosyltransferase 1 OS=Homo sapiens OX=9606 GN=UGGT1 PE=1 SV=3	-0,08686651	1,589514
P11117	Lysosomal acid phosphatase OS=Homo sapiens OX=9606 GN=ACP2 PE=1 SV=3	-0,260387746	1,589465
O75122	CLIP-associating protein 2 OS=Homo sapiens OX=9606 GN=CLASP2 PE=1 SV=3	0,166358166	1,587069
Q15056	Eukaryotic translation initiation factor 4H OS=Homo sapiens OX=9606 GN=EIF4H PE=1 SV=5	-0,148705161	1,587068
Q9UKA4	A-kinase anchor protein 11 OS=Homo sapiens OX=9606 GN=AKAP11 PE=1 SV=1	0,259955272	1,586311
O75691	Small subunit processome component 20 homolog OS=Homo sapiens OX=9606 GN=UTP20 PE=1 SV=3	-0,153814405	1,585308
Q9Y496	Kinesin-like protein KIF3A OS=Homo sapiens OX=9606 GN=KIF3A PE=1 SV=4	-0,236264183	1,584369
P01023	Alpha-2-macroglobulin OS=Homo sapiens OX=9606 GN=A2M PE=1 SV=3	0,259609878	1,583793
Q00535	Cyclin-dependent-like kinase 5 OS=Homo sapiens OX=9606 GN=CDK5 PE=1 SV=3	-0,259511588	1,583076
O00767	Acyl-CoA desaturase OS=Homo sapiens OX=9606 GN=SCD PE=1 SV=2	-0,726703765	1,582835
Q7Z3B4	Nucleoporin p54 OS=Homo sapiens OX=9606 GN=NUP54 PE=1 SV=2	0,191791728	1,581166
Q00796	Sorbitol dehydrogenase OS=Homo sapiens OX=9606 GN=SORD PE=1 SV=4	-0,191744693	1,580681
Q8NEF9	Serum response factor-binding protein 1 OS=Homo sapiens OX=9606 GN=SRFBP1 PE=1 SV=1	0,725084831	1,580176
Q9H5V9	UPF0428 protein CXorf56 OS=Homo sapiens OX=9606 GN=CXorf56 PE=1 SV=1	0,72485052	1,579791
O75391	Sperm-associated antigen 7 OS=Homo sapiens OX=9606 GN=SPAG7 PE=1 SV=2	0,259041748	1,579652
Q9Y4B6	DDB1- and CUL4-associated factor 1 OS=Homo sapiens OX=9606 GN=DCAF1 PE=1 SV=3	-0,217759667	1,579574
Q05655	Protein kinase C delta type OS=Homo sapiens OX=9606 GN=PRKCD PE=1 SV=2	-0,258548244	1,576056
P08397	Porphobilinogen deaminase OS=Homo sapiens OX=9606 GN=HMBS PE=1 SV=2	0,290767907	1,575674
Q8TCT9	Minor histocompatibility antigen H13 OS=Homo sapiens OX=9606 GN=HM13 PE=1 SV=1	0,217242419	1,574958
Q9NQA3	WAS protein family homolog 6 OS=Homo sapiens OX=9606 GN=WASH6P PE=1 SV=3	-0,431040368	1,573669
Q9Y2G5	GDP-fucose protein O-fucosyltransferase 2 OS=Homo sapiens OX=9606 GN=POFUT2 PE=1 SV=3	0,258219822	1,573664
Q8TER5	Rho guanine nucleotide exchange factor 40 OS=Homo sapiens OX=9606 GN=ARHGEF40 PE=1 SV=3	0,33973158	1,573645
Q13526	Peptidyl-prolyl cis-trans isomerase NIMA-interacting 1 OS=Homo sapiens OX=9606 GN=PIN1 PE=1 SV=1	-0,202783827	1,573286
Q9Y2W6	Tudor and KH domain-containing protein OS=Homo sapiens OX=9606 GN=TDRKH PE=1 SV=2	-0,339253544	1,571181
Q9HCS7	Pre-mRNA-splicing factor SYF1 OS=Homo sapiens OX=9606 GN=XAB2 PE=1 SV=2	-0,202493759	1,570492
Q13423	NAD(P) transhydrogenase, mitochondrial OS=Homo sapiens OX=9606 GN=NNT PE=1 SV=3	0,142770778	1,57022
Q13033	Striatin-3 OS=Homo sapiens OX=9606 GN=STRN3 PE=1 SV=3	0,338907246	1,569396
Q9HC98	Serine/threonine-protein kinase Nek6 OS=Homo sapiens OX=9606 GN=NEK6 PE=1 SV=2	0,682581429	1,569368
Q6ZS17	Rho family-interacting cell polarization regulator 1 OS=Homo sapiens OX=9606 GN=RIPOR1 PE=1 SV=1	0,202370756	1,569308
Q9H977	WD repeat-containing protein 54 OS=Homo sapiens OX=9606 GN=WDR54 PE=1 SV=1	-0,429709208	1,568745
P20340	Ras-related protein Rab-6A OS=Homo sapiens OX=9606 GN=RAB6A PE=1 SV=3	-0,33829551	1,566243
Q99490	Arf-GAP with GTPase, ANK repeat and PH domain-containing protein 2 OS=Homo sapiens OX=9606 GN=AGAP2 PE=1 SV=2	-0,480792806	1,566088
Q15269	Periodic tryptophan protein 2 homolog OS=Homo sapiens OX=9606 GN=PWP2 PE=2 SV=2	0,338072492	1,565094
Q9Y6K9	NF-kappa-B essential modulator OS=Homo sapiens OX=9606 GN=IKBKG PE=1 SV=2	0,180278328	1,564118
Q9UHX1	Poly(U)-binding-splicing factor PUF60 OS=Homo sapiens OX=9606 GN=PUF60 PE=1 SV=1	-0,134132741	1,563444
P36542	ATP synthase subunit gamma, mitochondrial OS=Homo sapiens OX=9606 GN=ATP5F1C PE=1 SV=1	-0,201676402	1,562627
P10636	Microtubule-associated protein tau OS=Homo sapiens OX=9606 GN=MAPT PE=1 SV=5	0,33747729	1,562026
Q8NHP8	Putative phospholipase B-like 2 OS=Homo sapiens OX=9606 GN=PLBD2 PE=1 SV=2	-0,233249641	1,559828
Q6PD74	Alpha- and gamma-adaptin-binding protein p34 OS=Homo sapiens OX=9606 GN=AAGAB PE=1 SV=1	-0,427235767	1,559585
Q00403	Transcription initiation factor IIB OS=Homo sapiens OX=9606 GN=GTF2B PE=1 SV=1	0,712462905	1,559313
P23193	Transcription elongation factor A protein 1 OS=Homo sapiens OX=9606 GN=TCEA1 PE=1 SV=2	-0,179638252	1,557144
Q9UL12	Sarcosine dehydrogenase, mitochondrial OS=Homo sapiens OX=9606 GN=SARDH PE=1 SV=1	0,336445424	1,556708
P33981	Dual specificity protein kinase TTK OS=Homo sapiens OX=9606 GN=TTK PE=1 SV=2	-0,426405651	1,556508
P51148	Ras-related protein Rab-5C OS=Homo sapiens OX=9606 GN=RAB5C PE=1 SV=2	0,189385005	1,556404
P21964	Catechol O-methyltransferase OS=Homo sapiens OX=9606 GN=COMT PE=1 SV=2	0,163775141	1,555905
Q9BZQ8	Protein Niban OS=Homo sapiens OX=9606 GN=FAM129A PE=1 SV=1	-0,151546018	1,55557
Q9H254	Spectrin beta chain, non-erythrocytic 4 OS=Homo sapiens OX=9606 GN=SPTBN4 PE=1 SV=2	-0,425795865	1,554247
Q96JP5	E3 ubiquitin-protein ligase ZFP91 OS=Homo sapiens OX=9606 GN=ZFP91 PE=1 SV=1	0,708855579	1,553305
P19320	#N/D	-0,707374012	1,55315
Q96RK0	Protein capicua homolog OS=Homo sapiens OX=9606 GN=CIC PE=1 SV=2	-0,707601626	1,551212
Q6ZT12	E3 ubiquitin-protein ligase UBR3 OS=Homo sapiens OX=9606 GN=UBR3 PE=2 SV=2	-0,424253968	1,548528
Q9Y5B6	PAX3- and PAX7-binding protein 1 OS=Homo sapiens OX=9606 GN=PAXBP1 PE=1 SV=2	0,424163719	1,548193
O60287	Nucleolar pre-ribosomal-associated protein 1 OS=Homo sapiens OX=9606 GN=URB1 PE=1 SV=4	-0,200173619	1,548193
Q8IWT6	Volume-regulated anion channel subunit LRRC8A OS=Homo sapiens OX=9606 GN=LRRC8A PE=1 SV=1	0,214203778	1,547904
Q6P3W7	SCY1-like protein 2 OS=Homo sapiens OX=9606 GN=SCYL2 PE=1 SV=1	-0,178660799	1,546511
Q9NTK5	Obg-like ATPase 1 OS=Homo sapiens OX=9606 GN=OLA1 PE=1 SV=2	-0,141061976	1,546222
Q01970	1-phosphatidylinositol 4,5-bisphosphate phosphodiesterase beta-3 OS=Homo sapiens OX=9606 GN=PLCB3 PE=1 SV=2	-0,110621684	1,546062
Q9HAV0	Guanine nucleotide-binding protein subunit beta-4 OS=Homo sapiens OX=9606 GN=GNB4 PE=1 SV=3	-0,162849475	1,544782
P52747	Zinc finger protein 143 OS=Homo sapiens OX=9606 GN=ZNF143 PE=1 SV=2	-0,703489316	1,54433
Q9H583	HEAT repeat-containing protein 1 OS=Homo sapiens OX=9606 GN=HEATR1 PE=1 SV=3	-0,120154226	1,544272
O00629	Importin subunit alpha-3 OS=Homo sapiens OX=9606 GN=KPNA4 PE=1 SV=1	0,156372514	1,544046
Q07812	Apoptosis regulator BAX OS=Homo sapiens OX=9606 GN=BAX PE=1 SV=1	0,25414121	1,544007
P50750	Cyclin-dependent kinase 9 OS=Homo sapiens OX=9606 GN=CDK9 PE=1 SV=3	-0,285637203	1,543348
P49662	Caspase-4 OS=Homo sapiens OX=9606 GN=CASP4 PE=1 SV=1	0,247729781	1,541953
Q15003	Condensin complex subunit 2 OS=Homo sapiens OX=9606 GN=NCAPH PE=1 SV=3	-0,150489908	1,541785
Q587J7	Putative ATP-dependent RNA helicase TDRD12 OS=Homo sapiens OX=9606 GN=TDRD12 PE=2 SV=2	0,435569705	1,541037
P30154	Serine/threonine-protein phosphatase 2A 65 kDa regulatory subunit A beta isoform OS=Homo sapiens OX=9606 GN=PPP2	0,156105389	1,540699
Q8N668	COMM domain-containing protein 1 OS=Homo sapiens OX=9606 GN=COMMD1 PE=1 SV=1	-0,700795628	1,539808
Q9Y2U5	Mitogen-activated protein kinase kinase kinase 2 OS=Homo sapiens OX=9606 GN=MAP3K2 PE=1 SV=2	-0,699588474	1,537778
Q9Y3D8	Adenylate kinase isoenzyme 6 OS=Homo sapiens OX=9606 GN=AK6 PE=1 SV=1	0,420809921	1,535736
O75970	Multiple PDZ domain protein OS=Homo sapiens OX=9606 GN=MPDZ PE=1 SV=2	0,187272464	1,534755
Q6ZWT7	Lysophospholipid acyltransferase 2 OS=Homo sapiens OX=9606 GN=MBOAT2 PE=2 SV=2	0,697792509	1,534754
O14617	AP-3 complex subunit delta-1 OS=Homo sapiens OX=9606 GN=AP3D1 PE=1 SV=1	-0,104106763	1,533913
Q8NEW0	Zinc transporter 7 OS=Homo sapiens OX=9606 GN=SLC30A7 PE=2 SV=1	0,420224178	1,533558

Q9NSC5	Homer protein homolog 3 OS=Homo sapiens OX=9606 GN=HOMER3 PE=1 SV=2	-0,229934499	1,53293
Q15363	Transmembrane emp24 domain-containing protein 2 OS=Homo sapiens OX=9606 GN=TMED2 PE=1 SV=1	-0,21246949	1,532511
Q8WYA0	Intraflagellar transport protein 81 homolog OS=Homo sapiens OX=9606 GN=IFT81 PE=1 SV=1	-0,696449542	1,532488
Q8IXB1	DnaJ homolog subfamily C member 10 OS=Homo sapiens OX=9606 GN=DNAJC10 PE=1 SV=2	-0,177340928	1,532186
P49137	MAP kinase-activated protein kinase 2 OS=Homo sapiens OX=9606 GN=MAPKAPK2 PE=1 SV=1	-0,252304282	1,530681
O60831	PRA1 family protein 2 OS=Homo sapiens OX=9606 GN=PRAF2 PE=1 SV=1	-0,33131112	1,530248
Q9UPN3	Microtubule-actin cross-linking factor 1, isoforms 1/2/3/5 OS=Homo sapiens OX=9606 GN=MACF1 PE=1 SV=4	0,050435001	1,528677
Q96DH6	RNA-binding protein Musashi homolog 2 OS=Homo sapiens OX=9606 GN=MSI2 PE=1 SV=1	-0,198097178	1,528304
P16333	Cytoplasmic protein NCK1 OS=Homo sapiens OX=9606 GN=NCK1 PE=1 SV=1	-0,229327071	1,528012
P52594	Arf-GAP domain and FG repeat-containing protein 1 OS=Homo sapiens OX=9606 GN=AGFG1 PE=1 SV=2	-0,211906936	1,527526
P09619	Platelet-derived growth factor receptor beta OS=Homo sapiens OX=9606 GN=PDGFRB PE=1 SV=1	-0,251860845	1,527467
O75312	Zinc finger protein ZPR1 OS=Homo sapiens OX=9606 GN=ZPR1 PE=1 SV=1	0,251768452	1,526798
Q8WZ42	Titin OS=Homo sapiens OX=9606 GN=TTN PE=1 SV=4	0,418322597	1,526485
Q13492	Phosphatidylinositol-binding clathrin assembly protein OS=Homo sapiens OX=9606 GN=PICALM PE=1 SV=2	0,128034045	1,524889
Q14008	Cytoskeleton-associated protein 5 OS=Homo sapiens OX=9606 GN=CKAP5 PE=1 SV=3	0,066239966	1,524632
A2RRP1	Neuroblastoma-amplified sequence OS=Homo sapiens OX=9606 GN=NBAS PE=1 SV=2	-0,282279597	1,522233
Q9Y5M8	Signal recognition particle receptor subunit beta OS=Homo sapiens OX=9606 GN=SRPRB PE=1 SV=3	0,17624771	1,520349
P0DPB6	DNA-directed RNA polymerases I and III subunit RPAC2 OS=Homo sapiens OX=9606 GN=POLR1D PE=1 SV=1	0,688829616	1,519583
Q9P2E7	Protocadherin-10 OS=Homo sapiens OX=9606 GN=PCDH10 PE=2 SV=2	-0,328868085	1,517659
Q9UH62	Armadillo repeat-containing X-linked protein 3 OS=Homo sapiens OX=9606 GN=ARMCX3 PE=1 SV=1	0,686784292	1,516103
Q9Y4K1	Beta/gamma crystallin domain-containing protein 1 OS=Homo sapiens OX=9606 GN=CRYBG1 PE=1 SV=3	-0,227777132	1,515478
Q16637	Survival motor neuron protein OS=Homo sapiens OX=9606 GN=SMN1 PE=1 SV=1	0,686199904	1,515107
P22681	E3 ubiquitin-protein ligase CBL OS=Homo sapiens OX=9606 GN=CBL PE=1 SV=2	-0,169074512	1,514815
P69905	Hemoglobin subunit alpha OS=Homo sapiens OX=9606 GN=HBA1 PE=1 SV=2	1,141719873	1,514584
P08579	U2 small nuclear ribonucleoprotein B'' OS=Homo sapiens OX=9606 GN=SNRPB2 PE=1 SV=1	-0,281049324	1,514504
O96033	Molybdopterin synthase sulfur carrier subunit OS=Homo sapiens OX=9606 GN=MOCS2 PE=1 SV=1	0,28104171	1,514456
Q9NZW5	MAGUK p55 subfamily member 6 OS=Homo sapiens OX=9606 GN=MPP6 PE=1 SV=2	-0,414867058	1,513614
P14618	Pyruvate kinase PKM OS=Homo sapiens OX=9606 GN=PKM PE=1 SV=4	0,085626316	1,51351
Q8IV38	Ankyrin repeat and MYND domain-containing protein 2 OS=Homo sapiens OX=9606 GN=ANKMY2 PE=1 SV=1	-0,328042842	1,513407
O00192	Armadillo repeat protein deleted in velo-cardio-facial syndrome OS=Homo sapiens OX=9606 GN=ARVCF PE=1 SV=1	-0,467004134	1,512143
P55036	26S proteasome non-ATPase regulatory subunit 4 OS=Homo sapiens OX=9606 GN=PSMD4 PE=1 SV=1	-0,167065704	1,509769
Q86X83	COMM domain-containing protein 2 OS=Homo sapiens OX=9606 GN=COMMD2 PE=1 SV=2	-0,280135728	1,508767
P30519	Heme oxygenase 2 OS=Homo sapiens OX=9606 GN=HMOX2 PE=1 SV=2	-0,153499175	1,508165
Q14146	Unhealthy ribosome biogenesis protein 2 homolog OS=Homo sapiens OX=9606 GN=URB2 PE=1 SV=2	0,91438367	1,507313
P61204	ADP-ribosylation factor 3 OS=Homo sapiens OX=9606 GN=ARF3 PE=1 SV=2	-0,175006664	1,506943
P04424	Argininosuccinate lyase OS=Homo sapiens OX=9606 GN=ASL PE=1 SV=4	-0,681137226	1,50646
Q9NXG2	THUMP domain-containing protein 1 OS=Homo sapiens OX=9606 GN=THUMPD1 PE=1 SV=2	0,17495768	1,506415
P35580	Myosin-10 OS=Homo sapiens OX=9606 GN=MYH10 PE=1 SV=3	-0,060675233	1,505444
Q6P1X5	Transcription initiation factor TFIID subunit 2 OS=Homo sapiens OX=9606 GN=TAF2 PE=1 SV=3	-0,16542685	1,505126
P04439	HLA class I histocompatibility antigen, A-3 alpha chain OS=Homo sapiens OX=9606 GN=HLA-A PE=1 SV=2	-0,279506542	1,504818
Q9H2J4	Phosducin-like protein 3 OS=Homo sapiens OX=9606 GN=PDCL3 PE=1 SV=1	-0,15941201	1,503686
Q8WUM4	Programmed cell death 6-interacting protein OS=Homo sapiens OX=9606 GN=PDCD6IP PE=1 SV=1	-0,08517327	1,503132
Q9BRG1	Vacuolar protein-sorting-associated protein 25 OS=Homo sapiens OX=9606 GN=VPS25 PE=1 SV=1	-0,226244319	1,503103
Q9NYH9	U3 small nucleolar RNA-associated protein 6 homolog OS=Homo sapiens OX=9606 GN=UTP6 PE=2 SV=2	-0,678505781	1,501949
Q92604	Acyl-CoA:lysophosphatidylglycerol acyltransferase 1 OS=Homo sapiens OX=9606 GN=LPGAT1 PE=1 SV=1	-0,208987003	1,501712
Q8N129	Protein canopy homolog 4 OS=Homo sapiens OX=9606 GN=CNPY4 PE=2 SV=1	-0,278947344	1,501309
Q9UL26	Ras-related protein Rab-22A OS=Homo sapiens OX=9606 GN=RAB22A PE=1 SV=2	-0,678052615	1,501171
P18206	Vinculin OS=Homo sapiens OX=9606 GN=VCL PE=1 SV=4	0,068396579	1,50083
P33527	Multidrug resistance-associated protein 1 OS=Homo sapiens OX=9606 GN=ABCC1 PE=1 SV=3	-0,183890791	1,50027
P61247	40S ribosomal protein S3a OS=Homo sapiens OX=9606 GN=RPS3A PE=1 SV=2	-0,120213704	1,499735
Q15004	PCNA-associated factor OS=Homo sapiens OX=9606 GN=PCLAF PE=1 SV=1	-0,41095257	1,499008
Q9H6K5	Proline-rich protein 36 OS=Homo sapiens OX=9606 GN=PRR36 PE=1 SV=2	-0,676525554	1,498547
Q8TBX8	Phosphatidylinositol 5-phosphate 4-kinase type-2 gamma OS=Homo sapiens OX=9606 GN=PIP4K2C PE=1 SV=3	0,325134943	1,498425
P20248	Cyclin-A2 OS=Homo sapiens OX=9606 GN=CCNA2 PE=1 SV=2	0,32498721	1,497664
Q9UNL2	Translocon-associated protein subunit gamma OS=Homo sapiens OX=9606 GN=SSR3 PE=1 SV=1	0,330163447	1,497594
Q12802	A-kinase anchor protein 13 OS=Homo sapiens OX=9606 GN=AKAP13 PE=1 SV=2	0,194872025	1,497542
P06756	Integrin alpha-V OS=Homo sapiens OX=9606 GN=ITGAV PE=1 SV=2	0,183481752	1,496113
Q15397	Pumilio homolog 3 OS=Homo sapiens OX=9606 GN=PUM3 PE=1 SV=3	-0,194571986	1,494688
P47897	Glutamine--tRNA ligase OS=Homo sapiens OX=9606 GN=QARS PE=1 SV=1	-0,084783471	1,494224
Q9NTM9	Copper homeostasis protein cutC homolog OS=Homo sapiens OX=9606 GN=CUTC PE=1 SV=1	-0,673286017	1,492969
P54136	Arginine--tRNA ligase, cytoplasmic OS=Homo sapiens OX=9606 GN=RARS PE=1 SV=2	-0,095355382	1,488526
Q9Y303	N-acetylglucosamine-6-phosphate deacetylase OS=Homo sapiens OX=9606 GN=AMDHD2 PE=1 SV=2	-0,224423392	1,48843
Q13107	Ubiquitin carboxyl-terminal hydrolase 4 OS=Homo sapiens OX=9606 GN=USP4 PE=1 SV=3	0,246461409	1,488425
Q16563	Synaptophysin-like protein 1 OS=Homo sapiens OX=9606 GN=SYPL1 PE=1 SV=1	0,276700158	1,487218
O60684	Importin subunit alpha-7 OS=Homo sapiens OX=9606 GN=KPNA6 PE=1 SV=1	-0,146171636	1,485818
Q53GQ0	Very-long-chain 3-oxoacyl-CoA reductase OS=Homo sapiens OX=9606 GN=HSD17B12 PE=1 SV=2	-0,157884875	1,485535
O00255	Menin OS=Homo sapiens OX=9606 GN=MEN1 PE=1 SV=4	-0,406801118	1,485438
P35916	Vascular endothelial growth factor receptor 3 OS=Homo sapiens OX=9606 GN=FLT4 PE=1 SV=3	0,455197939	1,484973
Q5BJF2	Sigma intracellular receptor 2 OS=Homo sapiens OX=9606 GN=TMEM97 PE=1 SV=1	-0,66856586	1,484809
P42766	60S ribosomal protein L35 OS=Homo sapiens OX=9606 GN=RPL35 PE=1 SV=2	-0,322377932	1,484223
Q9BXW9	Fanconi anemia group D2 protein OS=Homo sapiens OX=9606 GN=FANCD2 PE=1 SV=2	-0,193425678	1,483798
Q9BTV5	Fibronectin type III and SPRY domain-containing protein 1 OS=Homo sapiens OX=9606 GN=FSD1 PE=1 SV=1	-0,245560394	1,481927
P14621	Acylphosphatase-2 OS=Homo sapiens OX=9606 GN=ACYP2 PE=1 SV=2	-0,666289832	1,480862
Q5T0F9	Coiled-coil and C2 domain-containing protein 1B OS=Homo sapiens OX=9606 GN=CC2D1B PE=1 SV=1	-0,321319817	1,478773
P78316	Nucleolar protein 14 OS=Homo sapiens OX=9606 GN=NOP14 PE=1 SV=3	-0,245097086	1,478588
Q96AQ6	Pre-B-cell leukemia transcription factor-interacting protein 1 OS=Homo sapiens OX=9606 GN=PBXIP1 PE=1 SV=1	0,157273263	1,478283
Q96RQ3	Methylcrotonoyl-CoA carboxylase subunit alpha, mitochondrial OS=Homo sapiens OX=9606 GN=MCCC1 PE=1 SV=3	-0,405358674	1,478091
Q9NQS7	Inner centromere protein OS=Homo sapiens OX=9606 GN=INCENP PE=1 SV=3	-0,801235632	1,477744
Q5M775	Cytospin-B OS=Homo sapiens OX=9606 GN=SPECC1 PE=1 SV=1	-0,405096894	1,477111
Q8N2G8	GH3 domain-containing protein OS=Homo sapiens OX=9606 GN=GHDC PE=1 SV=2	-0,253119477	1,476841
Q14596	Next to BRCA1 gene 1 protein OS=Homo sapiens OX=9606 GN=NBR1 PE=1 SV=3	0,624719735	1,476694
P28065	Proteasome subunit beta type-9 OS=Homo sapiens OX=9606 GN=PSMB9 PE=1 SV=2	-0,404861357	1,476229
A4D1E9	GTP-binding protein 10 OS=Homo sapiens OX=9606 GN=GTPBP10 PE=1 SV=1	-0,404763121	1,475861
Q9BZG1	Ras-related protein Rab-34 OS=Homo sapiens OX=9606 GN=RAB34 PE=1 SV=1	-0,181287319	1,473865
Q9NS69	Mitochondrial import receptor subunit TOM22 homolog OS=Homo sapiens OX=9606 GN=TOMM22 PE=1 SV=3	0,274522511	1,473577
P16083	Ribosyldihydronicotinamide dehydrogenase [quinone] OS=Homo sapiens OX=9606 GN=NQO2 PE=1 SV=5	0,320142297	1,472709
Q15018	BRISC complex subunit Abraxas 2 OS=Homo sapiens OX=9606 GN=ABRAXAS2 PE=1 SV=2	-0,320087299	1,472426
O00411	DNA-directed RNA polymerase, mitochondrial OS=Homo sapiens OX=9606 GN=POLRMT PE=1 SV=2	0,403683423	1,471817
P55268	Laminin subunit beta-2 OS=Homo sapiens OX=9606 GN=LAMB2 PE=1 SV=2	0,660883252	1,471451
Q2PPJ7	#N/D	-1,147803643	1,470981
P57723	Poly(rC)-binding protein 4 OS=Homo sapiens OX=9606 GN=PCBP4 PE=2 SV=1	-0,243785536	1,469141

P09972	Fructose-bisphosphate aldolase C OS=Homo sapiens OX=9606 GN=ALDOC PE=1 SV=2	0,142003327	1,467946
Q9Y3B3	Transmembrane emp24 domain-containing protein 7 OS=Homo sapiens OX=9606 GN=TMED7 PE=1 SV=2	0,243455758	1,466768
P43034	Platelet-activating factor acetylhydrolase IB subunit alpha OS=Homo sapiens OX=9606 GN=PAFAH1B1 PE=1 SV=2	-0,12416265	1,466159
Q96QG7	Myotubularin-related protein 9 OS=Homo sapiens OX=9606 GN=MTMR9 PE=1 SV=1	-0,657242287	1,465086
P00367	Glutamate dehydrogenase 1, mitochondrial OS=Homo sapiens OX=9606 GN=GLUD1 PE=1 SV=2	-0,124046874	1,464415
P46776	60S ribosomal protein L27a OS=Homo sapiens OX=9606 GN=RPL27A PE=1 SV=2	-0,272978364	1,463914
Q5VT25	Serine/threonine-protein kinase MRCK alpha OS=Homo sapiens OX=9606 GN=CDC42BPA PE=1 SV=1	-0,221277411	1,463152
Q9BPX6	Calcium uptake protein 1, mitochondrial OS=Homo sapiens OX=9606 GN=MICU1 PE=1 SV=1	0,318278276	1,46311
Q9UKG1	DCC-interacting protein 13-alpha OS=Homo sapiens OX=9606 GN=APPL1 PE=1 SV=1	-0,318197756	1,462696
O94887	FERM, ARHGEF and pleckstrin domain-containing protein 2 OS=Homo sapiens OX=9606 GN=FARP2 PE=1 SV=3	0,655440828	1,461929
O43252	Bifunctional 3'-phosphoadenosine 5'-phosphosulfate synthase 1 OS=Homo sapiens OX=9606 GN=PAPSS1 PE=1 SV=2	-0,134963565	1,461552
Q9Y490	Talin-1 OS=Homo sapiens OX=9606 GN=TLN1 PE=1 SV=3	0,05079805	1,460091
Q9P2K5	Myelin expression factor 2 OS=Homo sapiens OX=9606 GN=MYEF2 PE=1 SV=3	0,155729865	1,460032
Q99729	Heterogeneous nuclear ribonucleoprotein A/B OS=Homo sapiens OX=9606 GN=HNRNPAB PE=1 SV=2	-0,220880461	1,459968
O43660	Pleiotropic regulator 1 OS=Homo sapiens OX=9606 GN=PLRG1 PE=1 SV=1	-0,179684645	1,457673
Q6DN12	Multiple C2 and transmembrane domain-containing protein 2 OS=Homo sapiens OX=9606 GN=MCTP2 PE=1 SV=3	-0,652515516	1,456791
O14531	Dihydropyrimidinase-related protein 4 OS=Homo sapiens OX=9606 GN=DPYSL4 PE=1 SV=2	-0,203872716	1,456749
Q99973	Telomerase protein component 1 OS=Homo sapiens OX=9606 GN=TEP1 PE=1 SV=2	0,175848992	1,456031
Q96PU8	Protein quaking OS=Homo sapiens OX=9606 GN=QKI PE=1 SV=1	-0,24172482	1,454321
P49257	Protein ERGIC-53 OS=Homo sapiens OX=9606 GN=LMAN1 PE=1 SV=2	0,162079824	1,453379
Q96AX1	Vacuolar protein sorting-associated protein 33A OS=Homo sapiens OX=9606 GN=VPS33A PE=1 SV=1	0,219776053	1,45112
Q9UKX7	Nuclear pore complex protein Nup50 OS=Homo sapiens OX=9606 GN=NUP50 PE=1 SV=2	-0,161824819	1,450511
P18077	60S ribosomal protein L35a OS=Homo sapiens OX=9606 GN=RPL35A PE=1 SV=2	-0,203141962	1,450351
P07093	Glia-derived nexin OS=Homo sapiens OX=9606 GN=SERPINE2 PE=1 SV=1	-0,189760371	1,449114
Q96GC5	39S ribosomal protein L48, mitochondrial OS=Homo sapiens OX=9606 GN=MRPL48 PE=1 SV=2	-0,648028863	1,448882
Q8WU76	Sec1 family domain-containing protein 2 OS=Homo sapiens OX=9606 GN=SCFD2 PE=1 SV=2	-0,513422543	1,447857
P18084	Integrin beta-5 OS=Homo sapiens OX=9606 GN=ITGB5 PE=1 SV=1	-0,315165677	1,447086
Q7Z460	CLIP-associating protein 1 OS=Homo sapiens OX=9606 GN=CLASP1 PE=1 SV=1	0,094681844	1,446077
Q6P2C8	Mediator of RNA polymerase II transcription subunit 27 OS=Homo sapiens OX=9606 GN=MED27 PE=1 SV=1	-0,646249271	1,445736
Q9H330	Transmembrane protein 245 OS=Homo sapiens OX=9606 GN=TMEM245 PE=1 SV=3	-0,270058295	1,445661
Q9NRW3	DNA dC->dU-editing enzyme APOBEC-3C OS=Homo sapiens OX=9606 GN=APOBEC3C PE=1 SV=2	0,240353161	1,44447
Q9NRG9	Aladin OS=Homo sapiens OX=9606 GN=AAAS PE=1 SV=1	-0,240342743	1,444396
Q9Y678	Coatomer subunit gamma-1 OS=Homo sapiens OX=9606 GN=COPG1 PE=1 SV=1	0,07805525	1,443866
Q9NRV9	Heme-binding protein 1 OS=Homo sapiens OX=9606 GN=HEBP1 PE=1 SV=1	0,202227757	1,442356
P49721	Proteasome subunit beta type-2 OS=Homo sapiens OX=9606 GN=PSMB2 PE=1 SV=1	-0,154166116	1,44161
P57772	Selenocysteine-specific elongation factor OS=Homo sapiens OX=9606 GN=EEFSEC PE=1 SV=4	-0,3952722	1,440244
Q9UPN4	Centrosomal protein of 131 kDa OS=Homo sapiens OX=9606 GN=CEP131 PE=1 SV=3	0,64214546	1,438461
O14976	Cyclin-G-associated kinase OS=Homo sapiens OX=9606 GN=GAK PE=1 SV=2	-0,201723926	1,437954
Q9NUM4	Transmembrane protein 106B OS=Homo sapiens OX=9606 GN=TMEM106B PE=1 SV=2	-0,394660705	1,437944
P25490	Transcriptional repressor protein YY1 OS=Homo sapiens OX=9606 GN=YY1 PE=1 SV=2	0,313237074	1,43716
P36915	Guanine nucleotide-binding protein-like 1 OS=Homo sapiens OX=9606 GN=GNL1 PE=1 SV=2	-0,2686768	1,437035
Q9Y5P6	Mannose-1-phosphate guanyltransferase beta OS=Homo sapiens OX=9606 GN=GMPPB PE=1 SV=2	0,313203292	1,436986
Q13618	Cullin-3 OS=Homo sapiens OX=9606 GN=CUL3 PE=1 SV=2	0,129186041	1,436774
P39656	Dolichyl-diphosphooligosaccharide--protein glycosyltransferase 48 kDa subunit OS=Homo sapiens OX=9606 GN=DDOST P	0,168367999	1,4358
Q86WJ1	Chromodomain-helicase-DNA-binding protein 1-like OS=Homo sapiens OX=9606 GN=CHD1L PE=1 SV=3	-0,640534767	1,435597
Q9BT09	Protein canopy homolog 3 OS=Homo sapiens OX=9606 GN=CNPY3 PE=1 SV=1	0,201257087	1,433878
Q9BV38	WD repeat-containing protein 18 OS=Homo sapiens OX=9606 GN=WDR18 PE=1 SV=2	-0,201157342	1,433008
Q6ZMK1	Cysteine and histidine-rich protein 1 OS=Homo sapiens OX=9606 GN=CYHR1 PE=1 SV=2	-0,638279094	1,43158
Q92503	SEC14-like protein 1 OS=Homo sapiens OX=9606 GN=SEC14L1 PE=1 SV=2	-0,312136409	1,431496
P28676	Grancalcin OS=Homo sapiens OX=9606 GN=GCA PE=1 SV=2	-0,238460544	1,430898
P84243	Histone H3.3 OS=Homo sapiens OX=9606 GN=H3F3A PE=1 SV=2	-0,311737661	1,429445
Q96BH1	E3 ubiquitin-protein ligase RNF25 OS=Homo sapiens OX=9606 GN=RNF25 PE=1 SV=1	-0,311609274	1,428784
P12956	X-ray repair cross-complementing protein 6 OS=Homo sapiens OX=9606 GN=XRCC6 PE=1 SV=2	-0,090938736	1,427471
A0AVT1	Ubiquitin-like modifier-activating enzyme 6 OS=Homo sapiens OX=9606 GN=UBA6 PE=1 SV=1	0,108149369	1,426561
O14735	CDP-diacylglycerol--inositol 3-phosphatidyltransferase OS=Homo sapiens OX=9606 GN=CDIPT PE=1 SV=1	-0,391615989	1,426484
O43674	NADH dehydrogenase [ubiquinone] 1 beta subcomplex subunit 5, mitochondrial OS=Homo sapiens OX=9606 GN=NDUFB5	-0,391093617	1,424517
Q7Z4H8	Protein O-glucosyltransferase 3 OS=Homo sapiens OX=9606 GN=POGLUT3 PE=1 SV=2	-0,152612342	1,423374
Q3MHD2	Protein LSM12 homolog OS=Homo sapiens OX=9606 GN=LSM12 PE=1 SV=2	-0,390540465	1,422433
P56182	Ribosomal RNA processing protein 1 homolog A OS=Homo sapiens OX=9606 GN=RRP1 PE=1 SV=1	-0,237089926	1,421084
Q13630	GDP-L-fucose synthase OS=Homo sapiens OX=9606 GN=TSTA3 PE=1 SV=1	-0,152414284	1,421054
O00178	GTP-binding protein 1 OS=Homo sapiens OX=9606 GN=GTPBP1 PE=1 SV=3	0,632274664	1,420844
Q16836	Hydroxyacyl-coenzyme A dehydrogenase, mitochondrial OS=Homo sapiens OX=9606 GN=HADH PE=1 SV=3	-0,186684249	1,420167
O95628	CCR4-NOT transcription complex subunit 4 OS=Homo sapiens OX=9606 GN=CNOT4 PE=1 SV=3	-0,306451978	1,419751
Q15545	Transcription initiation factor TFIID subunit 7 OS=Homo sapiens OX=9606 GN=TAF7 PE=1 SV=1	0,389767554	1,41952
Q8NAV1	Pre-mRNA-splicing factor 38A OS=Homo sapiens OX=9606 GN=PRPF38A PE=1 SV=1	0,309589019	1,418391
O43379	WD repeat-containing protein 62 OS=Homo sapiens OX=9606 GN=WDR62 PE=1 SV=4	-0,265683711	1,418369
P15529	Membrane cofactor protein OS=Homo sapiens OX=9606 GN=CD46 PE=1 SV=3	-0,309259307	1,416695
Q8NCN5	Pyruvate dehydrogenase phosphatase regulatory subunit, mitochondrial OS=Homo sapiens OX=9606 GN=PDPR PE=1 SV=	0,388997262	1,416617
Q6N063	2-oxoglutarate and iron-dependent oxygenase domain-containing protein 2 OS=Homo sapiens OX=9606 GN=OGFOD2 PE=	-0,571143284	1,41626
Q14192	Four and a half LIM domains protein 2 OS=Homo sapiens OX=9606 GN=FHL2 PE=1 SV=3	0,236369613	1,415931
P49441	Inositol polyphosphate 1-phosphatase OS=Homo sapiens OX=9606 GN=INPP1 PE=1 SV=1	0,308864915	1,414666
P24534	Elongation factor 1-beta OS=Homo sapiens OX=9606 GN=EEF1B2 PE=1 SV=3	-0,15857006	1,414059
Q15648	Mediator of RNA polymerase II transcription subunit 1 OS=Homo sapiens OX=9606 GN=MED1 PE=1 SV=4	-0,235971198	1,413082
Q12974	Protein tyrosine phosphatase type IVA 2 OS=Homo sapiens OX=9606 GN=PTP4A2 PE=1 SV=1	-0,627792351	1,41279
Q6PI48	Aspartate--tRNA ligase, mitochondrial OS=Homo sapiens OX=9606 GN=DARS2 PE=1 SV=1	-0,198676734	1,411401
Q96MW5	Conserved oligomeric Golgi complex subunit 8 OS=Homo sapiens OX=9606 GN=COG8 PE=1 SV=2	0,626936068	1,411248
Q86WX3	Active regulator of SIRT1 OS=Homo sapiens OX=9606 GN=RPS19BP1 PE=1 SV=1	0,125812761	1,411135
O14730	Serine/threonine-protein kinase RIO3 OS=Homo sapiens OX=9606 GN=RIOK3 PE=1 SV=2	-0,626659731	1,41075
Q9UH65	Switch-associated protein 70 OS=Homo sapiens OX=9606 GN=SWAP70 PE=1 SV=1	0,117408553	1,410586
Q9BTC8	Metastasis-associated protein MTA3 OS=Homo sapiens OX=9606 GN=MTA3 PE=1 SV=2	-0,198449229	1,409423
Q6ZTR5	Cilia- and flagella-associated protein 47 OS=Homo sapiens OX=9606 GN=CFAP47 PE=2 SV=5	0,314418398	1,408117
Q86TB3	Alpha-protein kinase 2 OS=Homo sapiens OX=9606 GN=ALPK2 PE=2 SV=3	0,623975338	1,405904
Q70UQ0	Inhibitor of nuclear factor kappa-B kinase-interacting protein OS=Homo sapiens OX=9606 GN=IKBIP PE=1 SV=1	0,151110564	1,405813
Q8WWQ0	PH-interacting protein OS=Homo sapiens OX=9606 GN=PHIP PE=1 SV=2	-0,09158223	1,405447
P13591	Neural cell adhesion molecule 1 OS=Homo sapiens OX=9606 GN=NCAM1 PE=1 SV=3	0,385636406	1,403937
Q12866	Tyrosine-protein kinase Mer OS=Homo sapiens OX=9606 GN=MERTK PE=1 SV=2	0,741698214	1,401925
Q9NV06	DDB1- and CUL4-associated factor 13 OS=Homo sapiens OX=9606 GN=DCAF13 PE=1 SV=2	-0,621759796	1,401896
Q5HYI7	Metaxin-3 OS=Homo sapiens OX=9606 GN=MTX3 PE=1 SV=2	-0,880018908	1,401727
Q8NCX0	Coiled-coil domain-containing protein 150 OS=Homo sapiens OX=9606 GN=CCDC150 PE=1 SV=2	0,447675557	1,40087
Q9Y3Z3	Deoxynucleoside triphosphate triphosphohydrolase SAMHD1 OS=Homo sapiens OX=9606 GN=SAMHD1 PE=1 SV=2	0,165049862	1,400606
O43301	Heat shock 70 kDa protein 12A OS=Homo sapiens OX=9606 GN=HSPA12A PE=1 SV=2	0,213383197	1,400127

O43172	U4/U6 small nuclear ribonucleoprotein Prp4 OS=Homo sapiens OX=9606 GN=PRPF4 PE=1 SV=2	0,13943883	1,399848
Q8NHG7	Small VCP/p97-interacting protein OS=Homo sapiens OX=9606 GN=SVIP PE=1 SV=1	-0,069323807	1,398348
P31327	Carbamoyl-phosphate synthase [ammonia], mitochondrial OS=Homo sapiens OX=9606 GN=CPS1 PE=1 SV=2	-0,383883486	1,397318
P26885	Peptidyl-prolyl cis-trans isomerase FKBP2 OS=Homo sapiens OX=9606 GN=FKBP2 PE=1 SV=2	0,383865674	1,39725
Q02447	Transcription factor Sp3 OS=Homo sapiens OX=9606 GN=SP3 PE=1 SV=3	-0,619170791	1,397201
O94760	N(G),N(G)-dimethylarginine dimethylaminohydrolase 1 OS=Homo sapiens OX=9606 GN=DDAH1 PE=1 SV=3	0,233641604	1,396446
Q16587	Zinc finger protein 74 OS=Homo sapiens OX=9606 GN=ZNF74 PE=1 SV=3	-0,618648498	1,396253
P22234	Multifunctional protein ADE2 OS=Homo sapiens OX=9606 GN=PAICS PE=1 SV=3	-0,119396641	1,394905
Q8WUZ0	B-cell CLL/lymphoma 7 protein family member C OS=Homo sapiens OX=9606 GN=BCL7C PE=1 SV=3	-0,617699873	1,394529
Q6AI08	HEAT repeat-containing protein 6 OS=Homo sapiens OX=9606 GN=HEATR6 PE=1 SV=1	0,261643968	1,393226
A8MXV4	Nucleoside diphosphate-linked moiety X motif 19 OS=Homo sapiens OX=9606 GN=NUDT19 PE=1 SV=1	-0,261563837	1,392728
Q8WX92	Negative elongation factor B OS=Homo sapiens OX=9606 GN=NELFB PE=1 SV=1	-0,19639346	1,391582
Q5VYK3	Proteasome adapter and scaffold protein ECM29 OS=Homo sapiens OX=9606 GN=ECPAS PE=1 SV=2	-0,085449045	1,391173
Q16543	Hsp90 co-chaperone Cdc37 OS=Homo sapiens OX=9606 GN=CDC37 PE=1 SV=1	-0,138606551	1,389331
P56524	Histone deacetylase 4 OS=Homo sapiens OX=9606 GN=HDAC4 PE=1 SV=3	-0,434589554	1,388586
Q9BZQ6	ER degradation-enhancing alpha-mannosidase-like protein 3 OS=Homo sapiens OX=9606 GN=EDEM3 PE=1 SV=2	-0,447078773	1,388557
Q14318	Peptidyl-prolyl cis-trans isomerase FKBP8 OS=Homo sapiens OX=9606 GN=FKBP8 PE=1 SV=2	-0,18320797	1,387636
P00568	Adenylate kinase isoenzyme 1 OS=Homo sapiens OX=9606 GN=AK1 PE=1 SV=3	-0,149527382	1,387371
Q13029	PR domain zinc finger protein 2 OS=Homo sapiens OX=9606 GN=PRDM2 PE=1 SV=3	-0,969676006	1,386804
O75155	Cullin-associated NEDD8-dissociated protein 2 OS=Homo sapiens OX=9606 GN=CAND2 PE=1 SV=3	-0,498531209	1,385601
Q9UIW2	Plexin-A1 OS=Homo sapiens OX=9606 GN=PLXNA1 PE=1 SV=3	-0,380731546	1,385403
Q13409	Cytoplasmic dynein 1 intermediate chain 2 OS=Homo sapiens OX=9606 GN=DYNC1I2 PE=1 SV=3	0,143486138	1,385159
P21283	V-type proton ATPase subunit C 1 OS=Homo sapiens OX=9606 GN=ATP6V1C1 PE=1 SV=4	-0,172439146	1,385078
Q6IA69	Glutamine-dependent NAD(+) synthetase OS=Homo sapiens OX=9606 GN=NADSYN1 PE=1 SV=3	-0,380633663	1,385033
O75323	Protein NipSnap homolog 2 OS=Homo sapiens OX=9606 GN=NIPSNAP2 PE=1 SV=1	-0,260175184	1,384099
O75533	Splicing factor 3B subunit 1 OS=Homo sapiens OX=9606 GN=SF3B1 PE=1 SV=3	-0,07106877	1,382376
Q15631	Translin OS=Homo sapiens OX=9606 GN=TSN PE=1 SV=1	0,172149211	1,382194
Q13464	Rho-associated protein kinase 1 OS=Homo sapiens OX=9606 GN=ROCK1 PE=1 SV=1	0,121740154	1,381658
Q14671	Pumilio homolog 1 OS=Homo sapiens OX=9606 GN=PUM1 PE=1 SV=3	0,195239089	1,381588
O43768	Alpha-endosulfine OS=Homo sapiens OX=9606 GN=ENSA PE=1 SV=1	-0,182438391	1,38046
Q6UWP7	Lysocardiolipin acyltransferase 1 OS=Homo sapiens OX=9606 GN=LCLAT1 PE=1 SV=1	-0,302162294	1,380209
Q13206	Probable ATP-dependent RNA helicase DDX10 OS=Homo sapiens OX=9606 GN=DDX10 PE=1 SV=2	0,15661782	1,377904
O75044	SLIT-ROBO Rho GTPase-activating protein 2 OS=Homo sapiens OX=9606 GN=SRGAP2 PE=1 SV=3	-0,182114579	1,377444
Q9NRR5	Ubiquilin-4 OS=Homo sapiens OX=9606 GN=UBQLN4 PE=1 SV=2	0,194574896	1,375845
P18440	Arylamine N-acetyltransferase 1 OS=Homo sapiens OX=9606 GN=NAT1 PE=1 SV=2	0,750585726	1,375675
P40939	Trifunctional enzyme subunit alpha, mitochondrial OS=Homo sapiens OX=9606 GN=HADHA PE=1 SV=2	-0,092475519	1,37484
Q8ND90	Paraneoplastic antigen Ma1 OS=Homo sapiens OX=9606 GN=PNMA1 PE=1 SV=2	-0,377854011	1,374513
Q9Y5B9	FACT complex subunit SPT16 OS=Homo sapiens OX=9606 GN=SUPT16H PE=1 SV=1	-0,097271807	1,374507
Q6ZS11	Ras and Rab interactor-like protein OS=Homo sapiens OX=9606 GN=RINL PE=2 SV=2	-0,605725119	1,372633
Q9H993	Damage-control phosphatase ARMT1 OS=Homo sapiens OX=9606 GN=ARMT1 PE=1 SV=1	-0,181564387	1,372323
Q13330	Metastasis-associated protein MTA1 OS=Homo sapiens OX=9606 GN=MTA1 PE=1 SV=2	-0,377085341	1,371602
P68036	Ubiquitin-conjugating enzyme E2 L3 OS=Homo sapiens OX=9606 GN=UBE2L3 PE=1 SV=1	-0,230149732	1,371578
O00541	Pescadillo homolog OS=Homo sapiens OX=9606 GN=PES1 PE=1 SV=1	-0,154725093	1,371344
Q9BTE6	Alanyl-tRNA editing protein Aarsd1 OS=Homo sapiens OX=9606 GN=AARSD1 PE=1 SV=2	-0,258117358	1,371325
O43678	NADH dehydrogenase [ubiquinone] 1 alpha subcomplex subunit 2 OS=Homo sapiens OX=9606 GN=NDUFA2 PE=1 SV=3	-0,376993671	1,371255
Q16644	MAP kinase-activated protein kinase 3 OS=Homo sapiens OX=9606 GN=MAPKAPK3 PE=1 SV=1	-0,768035105	1,369722
Q9NRR4	Ribonuclease 3 OS=Homo sapiens OX=9606 GN=DROSHA PE=1 SV=2	-0,232549624	1,369607
Q14197	Peptidyl-tRNA hydrolase ICT1, mitochondrial OS=Homo sapiens OX=9606 GN=MRPL58 PE=1 SV=1	-0,603807437	1,369103
Q9UPZ3	Hermansky-Pudlak syndrome 5 protein OS=Homo sapiens OX=9606 GN=HPS5 PE=1 SV=2	-0,603108421	1,367815
Q9NXK8	F-box/LRR-repeat protein 12 OS=Homo sapiens OX=9606 GN=FBXL12 PE=1 SV=1	-0,602897746	1,367426
Q9H2K8	Serine/threonine-protein kinase TAO3 OS=Homo sapiens OX=9606 GN=TAOK3 PE=1 SV=2	0,60260352	1,366884
Q9NVX2	Notchless protein homolog 1 OS=Homo sapiens OX=9606 GN=NLE1 PE=1 SV=4	-0,144915709	1,366326
Q9NQG5	Regulation of nuclear pre-mRNA domain-containing protein 1B OS=Homo sapiens OX=9606 GN=RPRD1B PE=1 SV=1	0,22939714	1,366228
Q9Y287	Integral membrane protein 2B OS=Homo sapiens OX=9606 GN=ITM2B PE=1 SV=1	0,601948039	1,365674
Q14694	Ubiquitin carboxyl-terminal hydrolase 10 OS=Homo sapiens OX=9606 GN=USP10 PE=1 SV=2	-0,124086825	1,36532
P52179	Myomesin-1 OS=Homo sapiens OX=9606 GN=MYOM1 PE=1 SV=2	-0,601585804	1,365006
P53597	Succinate--CoA ligase [ADP/GDP-forming] subunit alpha, mitochondrial OS=Homo sapiens OX=9606 GN=SUCLG1 PE=1 SV=	-0,193208118	1,364046
O14949	Cytochrome b-c1 complex subunit 8 OS=Homo sapiens OX=9606 GN=UQCRQ PE=1 SV=4	-0,298996032	1,363945
Q9BS16	Centromere protein K OS=Homo sapiens OX=9606 GN=CENPK PE=1 SV=1	-0,600546777	1,363086
Q6SJ93	Protein FAM111B OS=Homo sapiens OX=9606 GN=FAM111B PE=1 SV=1	-0,599527161	1,361201
P16157	Ankyrin-1 OS=Homo sapiens OX=9606 GN=ANK1 PE=1 SV=3	0,136352604	1,360972
P78344	Eukaryotic translation initiation factor 4 gamma 2 OS=Homo sapiens OX=9606 GN=EIF4G2 PE=1 SV=1	0,087600221	1,360699
Q9BRR6	ADP-dependent glucokinase OS=Homo sapiens OX=9606 GN=ADPGK PE=1 SV=1	0,208391968	1,360589
Q9NWZ5	Uridine-cytidine kinase-like 1 OS=Homo sapiens OX=9606 GN=UCKL1 PE=1 SV=2	-0,348854237	1,360081
P41743	Protein kinase C iota type OS=Homo sapiens OX=9606 GN=PRKCI PE=1 SV=2	0,180235722	1,359976
Q13685	Angio-associated migratory cell protein OS=Homo sapiens OX=9606 GN=AAMP PE=1 SV=2	-0,256198503	1,359427
P61586	Transforming protein RhoA OS=Homo sapiens OX=9606 GN=RHOA PE=1 SV=1	0,208185252	1,358957
P08236	Beta-glucuronidase OS=Homo sapiens OX=9606 GN=GUSB PE=1 SV=2	-0,598313476	1,358955
Q12849	G-rich sequence factor 1 OS=Homo sapiens OX=9606 GN=GRSF1 PE=1 SV=3	-0,192578763	1,358621
Q9NSE4	Isoleucine--tRNA ligase, mitochondrial OS=Homo sapiens OX=9606 GN=IARS2 PE=1 SV=2	-0,091616733	1,358566
Q9BT88	Synaptotagmin-11 OS=Homo sapiens OX=9606 GN=SYT11 PE=1 SV=2	0,597690414	1,3578
Q15147	1-phosphatidylinositol 4,5-bisphosphate phosphodiesterase beta-4 OS=Homo sapiens OX=9606 GN=PLCB4 PE=1 SV=3	0,131468902	1,357421
Q9BUI4	DNA-directed RNA polymerase III subunit RPC3 OS=Homo sapiens OX=9606 GN=POLR3C PE=1 SV=1	0,595022072	1,356829
Q8NHQ1	Centrosomal protein of 70 kDa OS=Homo sapiens OX=9606 GN=CEP70 PE=1 SV=2	-0,368586004	1,356662
Q14145	Kelch-like ECH-associated protein 1 OS=Homo sapiens OX=9606 GN=KEAP1 PE=1 SV=2	0,081996301	1,356111
Q13509	Tubulin beta-3 chain OS=Homo sapiens OX=9606 GN=TUBB3 PE=1 SV=2	-0,135946103	1,355877
P23284	Peptidyl-prolyl cis-trans isomerase B OS=Homo sapiens OX=9606 GN=PPIB PE=1 SV=2	0,141001	1,355125
Q7Z6Z7	E3 ubiquitin-protein ligase HUWE1 OS=Homo sapiens OX=9606 GN=HUWE1 PE=1 SV=3	-0,055272456	1,355001
Q96KP4	Cytosolic non-specific dipeptidase OS=Homo sapiens OX=9606 GN=CNDP2 PE=1 SV=2	0,116691585	1,354981
Q92629	Delta-sarcoglycan OS=Homo sapiens OX=9606 GN=SGCD PE=1 SV=2	-1,187119759	1,354808
O75616	GTPase Era, mitochondrial OS=Homo sapiens OX=9606 GN=ERAL1 PE=1 SV=2	-0,386156349	1,354748
Q13823	Nucleolar GTP-binding protein 2 OS=Homo sapiens OX=9606 GN=GNL2 PE=1 SV=1	-0,255320795	1,35399
O75586	Mediator of RNA polymerase II transcription subunit 6 OS=Homo sapiens OX=9606 GN=MED6 PE=1 SV=2	0,595415782	1,35358
Q9NRX2	39S ribosomal protein L17, mitochondrial OS=Homo sapiens OX=9606 GN=MRPL17 PE=1 SV=1	-0,296807079	1,352707
P68400	Casein kinase II subunit alpha OS=Homo sapiens OX=9606 GN=CSNK2A1 PE=1 SV=1	0,146420746	1,351393
Q2TAA2	Isoamyl acetate-hydrolyzing esterase 1 homolog OS=Homo sapiens OX=9606 GN=IAH1 PE=1 SV=1	0,594127377	1,351186
P61764	Syntaxin-binding protein 1 OS=Homo sapiens OX=9606 GN=STXBP1 PE=1 SV=1	0,152897132	1,351169
Q9H0B6	Kinesin light chain 2 OS=Homo sapiens OX=9606 GN=KLC2 PE=1 SV=1	-0,146311718	1,350136
Q9H0H0	Integrator complex subunit 2 OS=Homo sapiens OX=9606 GN=INTS2 PE=1 SV=2	0,370765405	1,347638
Q96GD4	Aurora kinase B OS=Homo sapiens OX=9606 GN=AURKB PE=1 SV=3	-0,295792216	1,347498
Q7Z478	ATP-dependent RNA helicase DHX29 OS=Homo sapiens OX=9606 GN=DHX29 PE=1 SV=2	-0,107855131	1,345241

P08758	Annexin A5 OS=Homo sapiens OX=9606 GN=ANXA5 PE=1 SV=2	0,105467042	1,344828
P48637	Glutathione synthetase OS=Homo sapiens OX=9606 GN=GSS PE=1 SV=1	0,113069545	1,344778
Q9Y2V7	Conserved oligomeric Golgi complex subunit 6 OS=Homo sapiens OX=9606 GN=COG6 PE=1 SV=2	0,286391542	1,343764
Q16555	Dihydropyrimidinase-related protein 2 OS=Homo sapiens OX=9606 GN=DPYSL2 PE=1 SV=1	0,097216972	1,342177
Q8NHP6	Motile sperm domain-containing protein 2 OS=Homo sapiens OX=9606 GN=MOSPD2 PE=1 SV=1	0,190612868	1,341709
Q6AWC8	Putative uncharacterized protein LOC100129027 OS=Homo sapiens OX=9606 PE=5 SV=1	0,486142055	1,34103
Q96GM8	Target of EGR1 protein 1 OS=Homo sapiens OX=9606 GN=TOE1 PE=1 SV=1	-0,368997311	1,340924
Q14914	Prostaglandin reductase 1 OS=Homo sapiens OX=9606 GN=PTGR1 PE=1 SV=2	0,588453842	1,340608
P35249	Replication factor C subunit 4 OS=Homo sapiens OX=9606 GN=RFC4 PE=1 SV=2	-0,190482296	1,340587
Q8N3T6	Transmembrane protein 132C OS=Homo sapiens OX=9606 GN=TMEM132C PE=2 SV=3	-0,900642479	1,340517
Q16718	NADH dehydrogenase [ubiquinone] 1 alpha subcomplex subunit 5 OS=Homo sapiens OX=9606 GN=NDUFA5 PE=1 SV=3	0,253135833	1,340468
Q9BTD8	RNA-binding protein 42 OS=Homo sapiens OX=9606 GN=RBM42 PE=1 SV=1	0,36872923	1,339905
P30837	Aldehyde dehydrogenase X, mitochondrial OS=Homo sapiens OX=9606 GN=ALDH1B1 PE=1 SV=3	-0,205737242	1,339661
Q96A72	Protein mago nashi homolog 2 OS=Homo sapiens OX=9606 GN=MAGOHB PE=1 SV=1	-1,085290337	1,339422
Q9BYD2	39S ribosomal protein L9, mitochondrial OS=Homo sapiens OX=9606 GN=MRPL9 PE=1 SV=2	-0,368564629	1,33928
Q6P1L8	39S ribosomal protein L14, mitochondrial OS=Homo sapiens OX=9606 GN=MRPL14 PE=1 SV=1	-0,252780489	1,33827
Q8WWI5	Choline transporter-like protein 1 OS=Homo sapiens OX=9606 GN=SLC44A1 PE=1 SV=1	-0,587006283	1,3379
Q9NVE5	Ubiquitin carboxyl-terminal hydrolase 40 OS=Homo sapiens OX=9606 GN=USP40 PE=1 SV=3	-0,586919016	1,337736
Q96DV4	39S ribosomal protein L38, mitochondrial OS=Homo sapiens OX=9606 GN=MRPL38 PE=1 SV=2	-0,225236369	1,336726
Q8TES7	Fas-binding factor 1 OS=Homo sapiens OX=9606 GN=FBF1 PE=1 SV=2	-0,586374918	1,336717
Q5CZC0	Fibrous sheath-interacting protein 2 OS=Homo sapiens OX=9606 GN=FSIP2 PE=2 SV=4	-0,173058506	1,335478
Q86WB0	Nuclear-interacting partner of ALK OS=Homo sapiens OX=9606 GN=ZC3HC1 PE=1 SV=1	0,252304937	1,33533
Q12874	Splicing factor 3A subunit 3 OS=Homo sapiens OX=9606 GN=SF3A3 PE=1 SV=1	0,129696969	1,334457
Q9NUY8	TBC1 domain family member 23 OS=Homo sapiens OX=9606 GN=TBC1D23 PE=1 SV=3	0,224910247	1,334418
P63165	Small ubiquitin-related modifier 1 OS=Homo sapiens OX=9606 GN=SUMO1 PE=1 SV=1	-0,251882683	1,332721
O75792	Ribonuclease H2 subunit A OS=Homo sapiens OX=9606 GN=RNASEH2A PE=1 SV=2	-0,292885409	1,332585
Q6ZRY4	RNA-binding protein with multiple splicing 2 OS=Homo sapiens OX=9606 GN=RBPMS2 PE=1 SV=1	-0,583672917	1,331649
Q8IYS2	Uncharacterized protein KIAA2013 OS=Homo sapiens OX=9606 GN=KIAA2013 PE=1 SV=1	-0,583447808	1,331226
P78536	Disintegrin and metalloproteinase domain-containing protein 17 OS=Homo sapiens OX=9606 GN=ADAM17 PE=1 SV=1	0,292606051	1,331152
Q9BUE0	Mediator of RNA polymerase II transcription subunit 18 OS=Homo sapiens OX=9606 GN=MED18 PE=1 SV=1	0,548854219	1,330393
Q9BTM9	Ubiquitin-related modifier 1 OS=Homo sapiens OX=9606 GN=URM1 PE=1 SV=1	-0,366224172	1,330385
O75369	Filamin-B OS=Homo sapiens OX=9606 GN=FLNB PE=1 SV=2	-0,044373755	1,328843
Q7L0J3	Synaptic vesicle glycoprotein 2A OS=Homo sapiens OX=9606 GN=SV2A PE=1 SV=1	-0,581595672	1,327744
Q03518	Antigen peptide transporter 1 OS=Homo sapiens OX=9606 GN=TAP1 PE=1 SV=2	0,223951683	1,327641
Q9BZE4	Nucleolar GTP-binding protein 1 OS=Homo sapiens OX=9606 GN=GTPBP4 PE=1 SV=3	-0,114790254	1,327144
Q9NRX1	RNA-binding protein PNO1 OS=Homo sapiens OX=9606 GN=PNO1 PE=1 SV=1	-0,250903804	1,326673
Q5T160	Probable arginine--tRNA ligase, mitochondrial OS=Homo sapiens OX=9606 GN=RARS2 PE=1 SV=1	0,8411369	1,326669
P49753	Acyl-coenzyme A thioesterase 2, mitochondrial OS=Homo sapiens OX=9606 GN=ACOT2 PE=1 SV=6	-0,580685542	1,326031
Q9NRX4	14 kDa phosphohistidine phosphatase OS=Homo sapiens OX=9606 GN=PHPT1 PE=1 SV=1	0,203968153	1,325753
Q09161	Nuclear cap-binding protein subunit 1 OS=Homo sapiens OX=9606 GN=NCBP1 PE=1 SV=1	0,109111253	1,325641
P35221	Catenin alpha-1 OS=Homo sapiens OX=9606 GN=CTNNA1 PE=1 SV=1	0,077216473	1,325053
P43490	Nicotinamide phosphoribosyltransferase OS=Homo sapiens OX=9606 GN=NAMPT PE=1 SV=1	0,12882647	1,323219
P55854	Small ubiquitin-related modifier 3 OS=Homo sapiens OX=9606 GN=SUMO3 PE=1 SV=2	-0,578939845	1,32274
O95825	Quinone oxidoreductase-like protein 1 OS=Homo sapiens OX=9606 GN=CRYZL1 PE=1 SV=2	0,203371666	1,321071
Q5VIR6	Vacuolar protein sorting-associated protein 53 homolog OS=Homo sapiens OX=9606 GN=VPS53 PE=1 SV=1	0,203340376	1,320826
Q9Y305	Acyl-coenzyme A thioesterase 9, mitochondrial OS=Homo sapiens OX=9606 GN=ACOT9 PE=1 SV=2	-0,111432686	1,320219
Q9NVU0	DNA-directed RNA polymerase III subunit RPC5 OS=Homo sapiens OX=9606 GN=POLR3E PE=1 SV=1	-0,107584089	1,319995
Q9NV96	Cell cycle control protein 50A OS=Homo sapiens OX=9606 GN=TMEM30A PE=1 SV=1	-0,362996189	1,318105
Q9H4A3	Serine/threonine-protein kinase WNK1 OS=Homo sapiens OX=9606 GN=WNK1 PE=1 SV=2	-0,362894774	1,317719
Q86WA8	Lon protease homolog 2, peroxisomal OS=Homo sapiens OX=9606 GN=LONP2 PE=1 SV=1	-0,362808877	1,317392
P49773	Histidine triad nucleotide-binding protein 1 OS=Homo sapiens OX=9606 GN=HINT1 PE=1 SV=2	0,17559048	1,317034
O94856	Neurofascin OS=Homo sapiens OX=9606 GN=NFASC PE=1 SV=4	0,36267882	1,316897
Q9NS91	E3 ubiquitin-protein ligase RAD18 OS=Homo sapiens OX=9606 GN=RAD18 PE=1 SV=2	-0,128333858	1,316873
Q00610	Clathrin heavy chain 1 OS=Homo sapiens OX=9606 GN=CLTC PE=1 SV=5	-0,051958612	1,316775
Q86YS6	Ras-related protein Rab-43 OS=Homo sapiens OX=9606 GN=RAB43 PE=1 SV=1	-0,24926972	1,316587
Q9H3P7	Golgi resident protein GCP60 OS=Homo sapiens OX=9606 GN=ACBD3 PE=1 SV=4	0,137639595	1,314817
P63104	14-3-3 protein zeta/delta OS=Homo sapiens OX=9606 GN=YWHAZ PE=1 SV=1	0,105898332	1,314423
Q13510	Acid ceramidase OS=Homo sapiens OX=9606 GN=ASAH1 PE=1 SV=5	-0,187305659	1,313373
Q8IUE6	Histone H2A type 2-B OS=Homo sapiens OX=9606 GN=HIST2H2AB PE=1 SV=3	-0,289088462	1,313119
Q6NXR4	TELO2-interacting protein 2 OS=Homo sapiens OX=9606 GN=TTI2 PE=1 SV=1	-0,573578122	1,312599
Q5H9R7	Serine/threonine-protein phosphatase 6 regulatory subunit 3 OS=Homo sapiens OX=9606 GN=PPP6R3 PE=1 SV=2	-0,105711278	1,31149
P62633	Cellular nucleic acid-binding protein OS=Homo sapiens OX=9606 GN=CNBP PE=1 SV=1	0,202006075	1,310366
Q16775	Hydroxyacylglutathione hydrolase, mitochondrial OS=Homo sapiens OX=9606 GN=HAGH PE=1 SV=2	-0,156199639	1,307946
O75251	NADH dehydrogenase [ubiquinone] iron-sulfur protein 7, mitochondrial OS=Homo sapiens OX=9606 GN=NDUFS7 PE=1 SV	-0,288042231	1,307758
O00303	Eukaryotic translation initiation factor 3 subunit F OS=Homo sapiens OX=9606 GN=EIF3F PE=1 SV=1	0,136711912	1,303758
O00267	Transcription elongation factor SPT5 OS=Homo sapiens OX=9606 GN=SUPT5H PE=1 SV=1	-0,098662883	1,302645
O15162	Phospholipid scramblase 1 OS=Homo sapiens OX=9606 GN=PLSCR1 PE=1 SV=1	-0,568167868	1,302313
O43837	Isocitrate dehydrogenase [NAD] subunit beta, mitochondrial OS=Homo sapiens OX=9606 GN=IDH3B PE=1 SV=2	0,173930633	1,301777
P0DPH7	Tubulin alpha-3C chain OS=Homo sapiens OX=9606 GN=TUBA3C PE=1 SV=1	0,091577473	1,301646
Q9BSC4	Nucleolar protein 10 OS=Homo sapiens OX=9606 GN=NOL10 PE=1 SV=1	-0,567560824	1,301155
Q9BQA9	Cytochrome b-245 chaperone 1 OS=Homo sapiens OX=9606 GN=CYBC1 PE=1 SV=1	0,286669906	1,300728
Q8N6Y2	Leucine-rich repeat-containing protein 17 OS=Homo sapiens OX=9606 GN=LRRC17 PE=2 SV=1	0,116043165	1,300557
Q6NUM9	All-trans-retinol 13,14-reductase OS=Homo sapiens OX=9606 GN=RETSAT PE=1 SV=2	-0,16381881	1,300028
Q9BUP0	EF-hand domain-containing protein D1 OS=Homo sapiens OX=9606 GN=EFHD1 PE=1 SV=1	-0,358057913	1,299295
P43897	Elongation factor Ts, mitochondrial OS=Homo sapiens OX=9606 GN=TSFM PE=1 SV=2	-0,185652743	1,299266
P26641	Elongation factor 1-gamma OS=Homo sapiens OX=9606 GN=EEF1G PE=1 SV=3	-0,09473651	1,298884
Q9ULE0	Protein WWC3 OS=Homo sapiens OX=9606 GN=WWC3 PE=1 SV=3	-1,278818677	1,298727
O15533	Tapasin OS=Homo sapiens OX=9606 GN=TAPBP PE=1 SV=1	-0,566219054	1,298595
Q9HAU5	Regulator of nonsense transcripts 2 OS=Homo sapiens OX=9606 GN=UPF2 PE=1 SV=1	0,357771118	1,298201
O75569	Interferon-inducible double-stranded RNA-dependent protein kinase activator A OS=Homo sapiens OX=9606 GN=PRKRA F	0,286110844	1,297865
Q53EP0	Fibronectin type III domain-containing protein 3B OS=Homo sapiens OX=9606 GN=FNDC3B PE=1 SV=2	0,185452035	1,297556
O75747	Phosphatidylinositol 4-phosphate 3-kinase C2 domain-containing subunit gamma OS=Homo sapiens OX=9606 GN=PIK3C2	-0,280604009	1,296487
Q04323	UBX domain-containing protein 1 OS=Homo sapiens OX=9606 GN=UBXN1 PE=1 SV=2	0,173346356	1,296417
O95232	Luc7-like protein 3 OS=Homo sapiens OX=9606 GN=LUC7L3 PE=1 SV=2	0,147695038	1,294231
O14966	Ras-related protein Rab-7L1 OS=Homo sapiens OX=9606 GN=RAB29 PE=1 SV=1	0,564049018	1,293582
Q14061	Cytochrome c oxidase copper chaperone OS=Homo sapiens OX=9606 GN=COX17 PE=1 SV=2	0,194684132	1,292696
Q9Y3A3	MOB-like protein phocein OS=Homo sapiens OX=9606 GN=MOB4 PE=1 SV=1	0,285024433	1,292302
Q12923	Tyrosine-protein phosphatase non-receptor type 13 OS=Homo sapiens OX=9606 GN=PTPN13 PE=1 SV=2	-0,223175659	1,29156
Q96AJ9	Vesicle transport through interaction with t-SNAREs homolog 1A OS=Homo sapiens OX=9606 GN=VTI1A PE=1 SV=2	0,506455471	1,290819
Q9NVU7	Protein SDA1 homolog OS=Homo sapiens OX=9606 GN=SDAD1 PE=1 SV=3	-0,283873899	1,286413
A6NHX0	Cytosolic arginine sensor for mTORC1 subunit 2 OS=Homo sapiens OX=9606 GN=CASTOR2 PE=1 SV=3	-0,559465801	1,285657

P40222	Alpha-taxilin OS=Homo sapiens OX=9606 GN=TXLNA PE=1 SV=3	-0,154022134	1,285422
Q9BXB4	Oxysterol-binding protein-related protein 11 OS=Homo sapiens OX=9606 GN=OSBPL11 PE=1 SV=2	0,162296686	1,285163
Q9NVI7	ATPase family AAA domain-containing protein 3A OS=Homo sapiens OX=9606 GN=ATAD3A PE=1 SV=2	0,162213639	1,284353
Q9Y4F1	FERM, ARHGEF and pleckstrin domain-containing protein 1 OS=Homo sapiens OX=9606 GN=FARP1 PE=1 SV=1	-0,140486498	1,283451
Q9NVV4	Poly(A) RNA polymerase, mitochondrial OS=Homo sapiens OX=9606 GN=MTPAP PE=1 SV=1	0,158543015	1,283107
O75113	NEDD4-binding protein 1 OS=Homo sapiens OX=9606 GN=N4BP1 PE=1 SV=4	-0,835318811	1,282766
P07858	Cathepsin B OS=Homo sapiens OX=9606 GN=CTSB PE=1 SV=3	0,171763629	1,281927
A6NKD9	Coiled-coil domain-containing protein 85C OS=Homo sapiens OX=9606 GN=CCDC85C PE=1 SV=1	-0,282644341	1,280121
O75822	Eukaryotic translation initiation factor 3 subunit J OS=Homo sapiens OX=9606 GN=EIF3J PE=1 SV=2	0,161748816	1,279824
Q5T5C0	Syntaxin-binding protein 5 OS=Homo sapiens OX=9606 GN=STXBP5 PE=1 SV=1	-0,176419286	1,278624
P07339	Cathepsin D OS=Homo sapiens OX=9606 GN=CTSD PE=1 SV=1	0,121376362	1,278446
O95793	Double-stranded RNA-binding protein Staufen homolog 1 OS=Homo sapiens OX=9606 GN=STAU1 PE=1 SV=2	-0,103584159	1,278279
Q641Q2	WASH complex subunit 2A OS=Homo sapiens OX=9606 GN=WASHC2A PE=1 SV=3	-0,899984753	1,277394
Q12834	Cell division cycle protein 20 homolog OS=Homo sapiens OX=9606 GN=CDC20 PE=1 SV=2	-0,281952122	1,27658
Q9HC07	Transmembrane protein 165 OS=Homo sapiens OX=9606 GN=TMEM165 PE=1 SV=1	-0,554545041	1,276177
P49750	YLP motif-containing protein 1 OS=Homo sapiens OX=9606 GN=YLPM1 PE=1 SV=4	-0,197414259	1,274512
O60427	Acyl-CoA (8-3)-desaturase OS=Homo sapiens OX=9606 GN=FADS1 PE=1 SV=3	-0,281413134	1,273823
O43920	NADH dehydrogenase [ubiquinone] iron-sulfur protein 5 OS=Homo sapiens OX=9606 GN=NDUFS5 PE=1 SV=3	0,281370418	1,273605
O00115	Deoxyribonuclease-2-alpha OS=Homo sapiens OX=9606 GN=DNASE2 PE=1 SV=2	-0,281199953	1,272733
Q6DN90	IQ motif and SEC7 domain-containing protein 1 OS=Homo sapiens OX=9606 GN=IQSEC1 PE=1 SV=1	-0,552743804	1,272696
Q8N6T3	ADP-ribosylation factor GTPase-activating protein 1 OS=Homo sapiens OX=9606 GN=ARFGAP1 PE=1 SV=2	0,160989381	1,272432
A6NIH7	Protein unc-119 homolog B OS=Homo sapiens OX=9606 GN=UNC119B PE=1 SV=1	-0,350974367	1,272262
Q7Z434	Mitochondrial antiviral-signaling protein OS=Homo sapiens OX=9606 GN=MAVS PE=1 SV=2	0,16094652	1,272015
O75182	Paired amphipathic helix protein Sin3b OS=Homo sapiens OX=9606 GN=SIN3B PE=1 SV=2	0,281015035	1,271787
O00425	Insulin-like growth factor 2 mRNA-binding protein 3 OS=Homo sapiens OX=9606 GN=IGF2BP3 PE=1 SV=2	0,124795992	1,271573
Q13671	Ras and Rab interactor 1 OS=Homo sapiens OX=9606 GN=RIN1 PE=1 SV=4	-0,280917067	1,271286
Q9H845	Acyl-CoA dehydrogenase family member 9, mitochondrial OS=Homo sapiens OX=9606 GN=ACAD9 PE=1 SV=1	-0,110922247	1,271088
Q9GZT6	Coiled-coil domain-containing protein 90B, mitochondrial OS=Homo sapiens OX=9606 GN=CCDC90B PE=1 SV=2	-0,55186612	1,270997
Q15257	Serine/threonine-protein phosphatase 2A activator OS=Homo sapiens OX=9606 GN=PTPA PE=1 SV=3	-0,152529751	1,270048
P05455	Lupus La protein OS=Homo sapiens OX=9606 GN=SSB PE=1 SV=2	0,110797794	1,269297
Q96CT7	Coiled-coil domain-containing protein 124 OS=Homo sapiens OX=9606 GN=CCDC124 PE=1 SV=1	0,196543814	1,26774
O15269	Serine palmitoyltransferase 1 OS=Homo sapiens OX=9606 GN=SPTLC1 PE=1 SV=1	0,196438108	1,266918
Q7Z392	Trafficking protein particle complex subunit 11 OS=Homo sapiens OX=9606 GN=TRAPPC11 PE=1 SV=2	-1,115786828	1,265767
Q70E73	Ras-associated and pleckstrin homology domains-containing protein 1 OS=Homo sapiens OX=9606 GN=RAPH1 PE=1 SV=3	0,169849443	1,264458
Q13617	Cullin-2 OS=Homo sapiens OX=9606 GN=CUL2 PE=1 SV=2	-0,110419716	1,263862
Q8IWC1	MAP7 domain-containing protein 3 OS=Homo sapiens OX=9606 GN=MAP7D3 PE=1 SV=2	0,823887904	1,263724
Q9BSD7	Cancer-related nucleoside-triphosphatase OS=Homo sapiens OX=9606 GN=NTPCR PE=1 SV=1	-0,181402863	1,263166
O43159	Ribosomal RNA-processing protein 8 OS=Homo sapiens OX=9606 GN=RRP8 PE=1 SV=2	-0,347642576	1,259528
Q9H446	RWD domain-containing protein 1 OS=Homo sapiens OX=9606 GN=RWDD1 PE=1 SV=1	0,545756195	1,259134
Q92804	TATA-binding protein-associated factor 2N OS=Homo sapiens OX=9606 GN=TAF15 PE=1 SV=1	0,23989584	1,258943
Q9BVJ6	U3 small nucleolar RNA-associated protein 14 homolog A OS=Homo sapiens OX=9606 GN=UTP14A PE=1 SV=1	-0,195357774	1,258526
Q9BZL6	#N/D	0,193776621	1,258417
Q8NB46	Serine/threonine-protein phosphatase 6 regulatory ankyrin repeat subunit C OS=Homo sapiens OX=9606 GN=ANKRD52 P	-0,159502923	1,257999
Q9ULV0	Unconventional myosin-Vb OS=Homo sapiens OX=9606 GN=MYO5B PE=1 SV=3	-0,347128658	1,257563
P53611	Geranylgeranyl transferase type-2 subunit beta OS=Homo sapiens OX=9606 GN=RABGGTB PE=1 SV=2	-0,346719899	1,255999
O94851	[F-actin]-monooxygenase MICAL2 OS=Homo sapiens OX=9606 GN=MICAL2 PE=1 SV=1	-0,138039195	1,255732
P17931	Galectin-3 OS=Homo sapiens OX=9606 GN=LGALS3 PE=1 SV=5	-0,194991943	1,255687
Q92963	GTP-binding protein Rit1 OS=Homo sapiens OX=9606 GN=RIT1 PE=1 SV=1	0,543660745	1,25505
Q14493	Histone RNA hairpin-binding protein OS=Homo sapiens OX=9606 GN=SLBP PE=1 SV=1	0,531671394	1,254195
O75150	E3 ubiquitin-protein ligase BRE1B OS=Homo sapiens OX=9606 GN=RNF40 PE=1 SV=5	-0,123403105	1,253873
O75718	Cartilage-associated protein OS=Homo sapiens OX=9606 GN=CRTAP PE=1 SV=1	0,127674537	1,253472
Q8TAE8	Growth arrest and DNA damage-inducible proteins-interacting protein 1 OS=Homo sapiens OX=9606 GN=GADD45GIP1 PE	-0,213247865	1,252408
O15127	Secretory carrier-associated membrane protein 2 OS=Homo sapiens OX=9606 GN=SCAMP2 PE=1 SV=2	0,542162952	1,252125
Q9NVN8	Guanine nucleotide-binding protein-like 3-like protein OS=Homo sapiens OX=9606 GN=GNL3L PE=1 SV=1	0,277045831	1,2515
P02792	Ferritin light chain OS=Homo sapiens OX=9606 GN=FTL PE=1 SV=2	-0,213063115	1,251117
Q96C86	m7GpppX diphosphatase OS=Homo sapiens OX=9606 GN=DCPS PE=1 SV=2	-0,212939931	1,250256
Q6PKG0	La-related protein 1 OS=Homo sapiens OX=9606 GN=LARP1 PE=1 SV=2	-0,088511843	1,246808
Q96C90	Protein phosphatase 1 regulatory subunit 14B OS=Homo sapiens OX=9606 GN=PPP1R14B PE=1 SV=3	0,275834851	1,245316
Q13424	Alpha-1-syntrophin OS=Homo sapiens OX=9606 GN=SNTA1 PE=1 SV=1	-0,538517643	1,244991
P48507	Glutamate--cysteine ligase regulatory subunit OS=Homo sapiens OX=9606 GN=GCLM PE=1 SV=1	0,275623683	1,244237
Q9NVH2	Integrator complex subunit 7 OS=Homo sapiens OX=9606 GN=INTS7 PE=1 SV=1	-0,538000919	1,243977
Q9BRT9	DNA replication complex GINS protein SLD5 OS=Homo sapiens OX=9606 GN=GINS4 PE=1 SV=1	-0,537893423	1,243766
P43378	Tyrosine-protein phosphatase non-receptor type 9 OS=Homo sapiens OX=9606 GN=PTPN9 PE=1 SV=1	-0,211891696	1,242936
Q9Y221	60S ribosome subunit biogenesis protein NIP7 homolog OS=Homo sapiens OX=9606 GN=NIP7 PE=1 SV=1	0,343254344	1,242738
O14646	Chromodomain-helicase-DNA-binding protein 1 OS=Homo sapiens OX=9606 GN=CHD1 PE=1 SV=2	0,275315838	1,242666
Q9UKG9	Peroxisomal carnitine O-octanoyltransferase OS=Homo sapiens OX=9606 GN=CROT PE=1 SV=2	0,34322976	1,242644
Q8NDT2	Putative RNA-binding protein 15B OS=Homo sapiens OX=9606 GN=RBM15B PE=1 SV=3	-0,343155818	1,242361
Q14202	Zinc finger MYM-type protein 3 OS=Homo sapiens OX=9606 GN=ZMYM3 PE=1 SV=2	-0,274962876	1,240864
Q96B26	Exosome complex component RRP43 OS=Homo sapiens OX=9606 GN=EXOSC8 PE=1 SV=1	0,211585457	1,2408
P19525	Interferon-induced, double-stranded RNA-activated protein kinase OS=Homo sapiens OX=9606 GN=EIF2AK2 PE=1 SV=2	-0,114976075	1,24057
Q08380	Galectin-3-binding protein OS=Homo sapiens OX=9606 GN=LGALS3BP PE=1 SV=1	0,136641098	1,239977
Q05086	Ubiquitin-protein ligase E3A OS=Homo sapiens OX=9606 GN=UBE3A PE=1 SV=4	0,178629258	1,239739
Q8TF01	Arginine/serine-rich protein PNISR OS=Homo sapiens OX=9606 GN=PNISR PE=1 SV=2	0,539105678	1,238966
O60568	Multifunctional procollagen lysine hydroxylase and glycosyltransferase LH3 OS=Homo sapiens OX=9606 GN=PLOD3 PE=1	0,094795242	1,238571
P12268	Inosine-5'-monophosphate dehydrogenase 2 OS=Homo sapiens OX=9606 GN=IMPDH2 PE=1 SV=2	0,094794971	1,238567
O75223	Gamma-glutamylcyclotransferase OS=Homo sapiens OX=9606 GN=GGCT PE=1 SV=1	-0,178428954	1,238051
Q9Y6D5	Brefeldin A-inhibited guanine nucleotide-exchange protein 2 OS=Homo sapiens OX=9606 GN=ARFGEF2 PE=1 SV=3	-0,211057539	1,237118
P31749	RAC-alpha serine/threonine-protein kinase OS=Homo sapiens OX=9606 GN=AKT1 PE=1 SV=2	-0,235967988	1,234903
Q9BSJ8	Extended synaptotagmin-1 OS=Homo sapiens OX=9606 GN=ESYT1 PE=1 SV=1	-0,075755686	1,234108
Q9HA77	Probable cysteine--tRNA ligase, mitochondrial OS=Homo sapiens OX=9606 GN=CARS2 PE=1 SV=1	0,340643885	1,232741
Q8NBX0	Saccharopine dehydrogenase-like oxidoreductase OS=Homo sapiens OX=9606 GN=SCCPDH PE=1 SV=1	0,14189653	1,231603
O60942	mRNA-capping enzyme OS=Homo sapiens OX=9606 GN=RNGTT PE=1 SV=1	0,340334981	1,231558
P08582	Melanotransferrin OS=Homo sapiens OX=9606 GN=MELTF PE=1 SV=2	-0,177585952	1,230954
Q99615	DnaJ homolog subfamily C member 7 OS=Homo sapiens OX=9606 GN=DNAJC7 PE=1 SV=2	0,100516558	1,230875
P05496	ATP synthase F(0) complex subunit C1, mitochondrial OS=Homo sapiens OX=9606 GN=ATP5MC1 PE=1 SV=2	-0,530870454	1,229943
Q96P47	Arf-GAP with GTPase, ANK repeat and PH domain-containing protein 3 OS=Homo sapiens OX=9606 GN=AGAP3 PE=1 SV=2	-0,530723519	1,229652
Q9Y295	Developmentally-regulated GTP-binding protein 1 OS=Homo sapiens OX=9606 GN=DRG1 PE=1 SV=1	-0,125704467	1,229444
Q92543	Sorting nexin-19 OS=Homo sapiens OX=9606 GN=SNX19 PE=1 SV=2	0,350623458	1,229219
Q9UII2	ATPase inhibitor, mitochondrial OS=Homo sapiens OX=9606 GN=ATP5IF1 PE=1 SV=1	-0,339606586	1,228767
Q13948	#N/D	-0,209817844	1,22848
P46781	40S ribosomal protein S9 OS=Homo sapiens OX=9606 GN=RPS9 PE=1 SV=3	-0,114073486	1,228417

Q9NTZ6	RNA-binding protein 12 OS=Homo sapiens OX=9606 GN=RBM12 PE=1 SV=1	-0,156430916	1,22831
Q17RY0	Cytoplasmic polyadenylation element-binding protein 4 OS=Homo sapiens OX=9606 GN=CPEB4 PE=1 SV=1	0,339142652	1,226989
Q9UNI6	Dual specificity protein phosphatase 12 OS=Homo sapiens OX=9606 GN=DUSP12 PE=1 SV=1	-0,272155826	1,22654
Q8IXM3	39S ribosomal protein L41, mitochondrial OS=Homo sapiens OX=9606 GN=MRPL41 PE=1 SV=1	-0,529005915	1,226257
Q9UHD2	Serine/threonine-protein kinase TBK1 OS=Homo sapiens OX=9606 GN=TBK1 PE=1 SV=1	-0,191066075	1,22531
Q9H501	ESF1 homolog OS=Homo sapiens OX=9606 GN=ESF1 PE=1 SV=1	0,271787427	1,224661
O95470	Sphingosine-1-phosphate lyase 1 OS=Homo sapiens OX=9606 GN=SGPL1 PE=1 SV=3	-0,176819324	1,224509
Q9NRZ7	1-acyl-sn-glycerol-3-phosphate acyltransferase gamma OS=Homo sapiens OX=9606 GN=AGPAT3 PE=1 SV=1	-0,271704527	1,224238
Q15121	Astrocytic phosphoprotein PEA-15 OS=Homo sapiens OX=9606 GN=PEA15 PE=1 SV=2	0,190781356	1,223113
Q2KHT3	Protein CLEC16A OS=Homo sapiens OX=9606 GN=CLEC16A PE=1 SV=2	0,265267604	1,222758
O43719	HIV Tat-specific factor 1 OS=Homo sapiens OX=9606 GN=HTATSF1 PE=1 SV=1	-0,526907391	1,222101
Q96S59	Ran-binding protein 9 OS=Homo sapiens OX=9606 GN=RANBP9 PE=1 SV=1	-0,271264003	1,221992
Q52LJ0	Protein FAM98B OS=Homo sapiens OX=9606 GN=FAM98B PE=1 SV=2	-0,165088147	1,221277
Q96T58	Msx2-interacting protein OS=Homo sapiens OX=9606 GN=SPEN PE=1 SV=1	0,86358371	1,22119
Q4KMQ1	Taperin OS=Homo sapiens OX=9606 GN=TPRN PE=1 SV=2	-0,18297193	1,218976
Q9HAV4	Exportin-5 OS=Homo sapiens OX=9606 GN=XPO5 PE=1 SV=1	-0,085462674	1,21858
O95870	Phosphatidylserine lipase ABHD16A OS=Homo sapiens OX=9606 GN=ABHD16A PE=1 SV=3	0,270454919	1,217867
Q96PE3	Type I inositol 3,4-bisphosphate 4-phosphatase OS=Homo sapiens OX=9606 GN=INPP4A PE=1 SV=1	0,653070901	1,21737
P06746	DNA polymerase beta OS=Homo sapiens OX=9606 GN=POLB PE=1 SV=3	-0,23308045	1,217275
O60870	DNA/RNA-binding protein KIN17 OS=Homo sapiens OX=9606 GN=KIN PE=1 SV=2	0,183295027	1,21725
P62249	40S ribosomal protein S16 OS=Homo sapiens OX=9606 GN=RPS16 PE=1 SV=2	-0,164592339	1,216802
Q96S82	Ubiquitin-like protein 7 OS=Homo sapiens OX=9606 GN=UBL7 PE=1 SV=2	0,523952738	1,216236
Q15291	Retinoblastoma-binding protein 5 OS=Homo sapiens OX=9606 GN=RBBP5 PE=1 SV=2	0,207776997	1,214286
P17812	CTP synthase 1 OS=Homo sapiens OX=9606 GN=CTPS1 PE=1 SV=2	-0,106911364	1,213781
Q99459	Cell division cycle 5-like protein OS=Homo sapiens OX=9606 GN=CDC5L PE=1 SV=2	0,112939418	1,213199
P45974	Ubiquitin carboxyl-terminal hydrolase 5 OS=Homo sapiens OX=9606 GN=USP5 PE=1 SV=2	-0,081112037	1,211215
Q96C19	EF-hand domain-containing protein D2 OS=Homo sapiens OX=9606 GN=EFHD2 PE=1 SV=1	-0,174895645	1,208373
Q6DD88	Atlastin-3 OS=Homo sapiens OX=9606 GN=ATL3 PE=1 SV=1	-0,109370217	1,207566
P63261	Actin, cytoplasmic 2 OS=Homo sapiens OX=9606 GN=ACTG1 PE=1 SV=1	0,333905712	1,206907
O14802	DNA-directed RNA polymerase III subunit RPC1 OS=Homo sapiens OX=9606 GN=POLR3A PE=1 SV=2	0,653245191	1,206839
Q9UMX0	Ubiquilin-1 OS=Homo sapiens OX=9606 GN=UBQLN1 PE=1 SV=2	0,123705637	1,205208
Q06587	E3 ubiquitin-protein ligase RING1 OS=Homo sapiens OX=9606 GN=RING1 PE=1 SV=2	-0,333291611	1,20455
Q16891	MICOS complex subunit MIC60 OS=Homo sapiens OX=9606 GN=IMMT PE=1 SV=1	-0,0819955	1,204412
P27708	CAD protein OS=Homo sapiens OX=9606 GN=CAD PE=1 SV=3	-0,056738052	1,20428
O00458	Interferon-related developmental regulator 1 OS=Homo sapiens OX=9606 GN=IFRD1 PE=1 SV=4	0,517555519	1,20348
Q99497	Protein/nucleic acid deglycase DJ-1 OS=Homo sapiens OX=9606 GN=PARK7 PE=1 SV=2	-0,123446977	1,202082
Q8N0U8	Vitamin K epoxide reductase complex subunit 1-like protein 1 OS=Homo sapiens OX=9606 GN=VKORC1L1 PE=1 SV=2	0,332264024	1,200606
O14657	Torsin-1B OS=Homo sapiens OX=9606 GN=TOR1B PE=1 SV=2	0,230107763	1,199168
Q96L91	E1A-binding protein p400 OS=Homo sapiens OX=9606 GN=EP400 PE=1 SV=4	-0,515316305	1,198997
Q9BQQ3	Golgi reassembly-stacking protein 1 OS=Homo sapiens OX=9606 GN=GORASP1 PE=1 SV=3	-0,515246666	1,198858
O15120	1-acyl-sn-glycerol-3-phosphate acyltransferase beta OS=Homo sapiens OX=9606 GN=AGPAT2 PE=1 SV=1	0,746299429	1,197692
P60660	Myosin light polypeptide 6 OS=Homo sapiens OX=9606 GN=MYL6 PE=1 SV=2	-0,153066183	1,196013
Q8N142	Adenylosuccinate synthetase isozyme 1 OS=Homo sapiens OX=9606 GN=ADSSL1 PE=1 SV=1	0,056508191	1,193906
P55072	Transitional endoplasmic reticulum ATPase OS=Homo sapiens OX=9606 GN=VCP PE=1 SV=4	0,059019696	1,193156
Q16401	26S proteasome non-ATPase regulatory subunit 5 OS=Homo sapiens OX=9606 GN=PSMD5 PE=1 SV=3	-0,105440493	1,192975
Q13614	Myotubularin-related protein 2 OS=Homo sapiens OX=9606 GN=MTMR2 PE=1 SV=4	0,511931727	1,192203
Q15843	NEDD8 OS=Homo sapiens OX=9606 GN=NEDD8 PE=1 SV=1	0,265333426	1,191783
Q9Y4K3	TNF receptor-associated factor 6 OS=Homo sapiens OX=9606 GN=TRAF6 PE=1 SV=1	-0,514395243	1,191365
P51659	Peroxisomal multifunctional enzyme type 2 OS=Homo sapiens OX=9606 GN=HSD17B4 PE=1 SV=3	-0,09010966	1,191034
Q14571	Inositol 1,4,5-trisphosphate receptor type 2 OS=Homo sapiens OX=9606 GN=ITPR2 PE=1 SV=2	0,259603593	1,190767
P42694	Probable helicase with zinc finger domain OS=Homo sapiens OX=9606 GN=HELZ PE=1 SV=2	0,30753306	1,188602
Q14012	Calcium/calmodulin-dependent protein kinase type 1 OS=Homo sapiens OX=9606 GN=CAMK1 PE=1 SV=1	-0,204023505	1,188265
Q7Z6E9	E3 ubiquitin-protein ligase RBBP6 OS=Homo sapiens OX=9606 GN=RBBP6 PE=1 SV=1	0,509855495	1,188025
P0CG08	Golgi pH regulator B OS=Homo sapiens OX=9606 GN=GPR89B PE=1 SV=1	0,32885481	1,187515
Q9BZK7	F-box-like/WD repeat-containing protein TBL1XR1 OS=Homo sapiens OX=9606 GN=TBL1XR1 PE=1 SV=1	-0,122234147	1,187459
Q16594	Transcription initiation factor TFIID subunit 9 OS=Homo sapiens OX=9606 GN=TAF9 PE=1 SV=1	-0,509567193	1,187444
Q9NZL9	Methionine adenosyltransferase 2 subunit beta OS=Homo sapiens OX=9606 GN=MAT2B PE=1 SV=1	0,131888257	1,186853
O75818	Ribonuclease P protein subunit p40 OS=Homo sapiens OX=9606 GN=RPP40 PE=1 SV=3	-0,203564688	1,185092
P46060	Ran GTPase-activating protein 1 OS=Homo sapiens OX=9606 GN=RANGAP1 PE=1 SV=1	-0,082221977	1,184864
Q13098	COP9 signalosome complex subunit 1 OS=Homo sapiens OX=9606 GN=GPS1 PE=1 SV=4	-0,11404002	1,182808
P98164	Low-density lipoprotein receptor-related protein 2 OS=Homo sapiens OX=9606 GN=LRP2 PE=1 SV=3	1,608761181	1,182766
P36957	Dihydrolipoyllysine-residue succinyltransferase component of 2-oxoglutarate dehydrogenase complex, mitochondrial OS=	-0,160704714	1,181866
O43818	U3 small nucleolar RNA-interacting protein 2 OS=Homo sapiens OX=9606 GN=RRP9 PE=1 SV=1	0,263175645	1,180808
Q13610	Periodic tryptophan protein 1 homolog OS=Homo sapiens OX=9606 GN=PWP1 PE=1 SV=1	-0,185231971	1,180478
O14972	Vacuolar protein sorting-associated protein 26C OS=Homo sapiens OX=9606 GN=VPS26C PE=1 SV=1	-0,326847773	1,179804
O95749	Geranylgeranyl pyrophosphate synthase OS=Homo sapiens OX=9606 GN=GGPS1 PE=1 SV=1	-0,262855465	1,17918
Q96DE0	U8 snoRNA-decapping enzyme OS=Homo sapiens OX=9606 GN=NUDT16 PE=1 SV=2	-0,180059937	1,179054
Q9BYD1	39S ribosomal protein L13, mitochondrial OS=Homo sapiens OX=9606 GN=MRPL13 PE=1 SV=1	-0,262829606	1,179048
Q9Y5V3	Melanoma-associated antigen D1 OS=Homo sapiens OX=9606 GN=MAGED1 PE=1 SV=3	-0,171372856	1,178958
P98175	RNA-binding protein 10 OS=Homo sapiens OX=9606 GN=RBM10 PE=1 SV=3	0,151237343	1,178556
O60888	Protein CutA OS=Homo sapiens OX=9606 GN=CUTA PE=1 SV=2	0,202609982	1,178495
Q8IUI8	Cytokine receptor-like factor 3 OS=Homo sapiens OX=9606 GN=CRLF3 PE=1 SV=2	0,326446644	1,178263
Q9C0I1	Myotubularin-related protein 12 OS=Homo sapiens OX=9606 GN=MTMR12 PE=1 SV=2	0,87747731	1,178189
P30740	Leukocyte elastase inhibitor OS=Homo sapiens OX=9606 GN=SERPINB1 PE=1 SV=1	-0,171220567	1,177691
P53814	Smoothelin OS=Homo sapiens OX=9606 GN=SMTN PE=1 SV=7	0,18463933	1,175944
Q96AV8	Transcription factor E2F7 OS=Homo sapiens OX=9606 GN=E2F7 PE=1 SV=3	-0,313070863	1,175229
Q9NQZ2	Something about silencing protein 10 OS=Homo sapiens OX=9606 GN=UTP3 PE=1 SV=1	-0,225799306	1,173
P13987	CD59 glycoprotein OS=Homo sapiens OX=9606 GN=CD59 PE=1 SV=1	0,261534458	1,172466
Q9H910	Jupiter microtubule associated homolog 2 OS=Homo sapiens OX=9606 GN=JPT2 PE=1 SV=1	-0,150529668	1,17182
O14745	Na(+)/H(+) exchange regulatory cofactor NHE-RF1 OS=Homo sapiens OX=9606 GN=SLC9A3R1 PE=1 SV=4	0,106738451	1,171488
Q9NVS9	Pyridoxine-5'-phosphate oxidase OS=Homo sapiens OX=9606 GN=PNPO PE=1 SV=1	-0,170448027	1,171265
P53999	Activated RNA polymerase II transcriptional coactivator p15 OS=Homo sapiens OX=9606 GN=SUB1 PE=1 SV=3	0,183917737	1,17043
Q8NDI1	EH domain-binding protein 1 OS=Homo sapiens OX=9606 GN=EHBP1 PE=1 SV=3	-0,201325581	1,169632
Q8N138	ORM1-like protein 3 OS=Homo sapiens OX=9606 GN=ORMDL3 PE=1 SV=1	0,500759497	1,169623
Q9BRZ2	E3 ubiquitin-protein ligase TRIM56 OS=Homo sapiens OX=9606 GN=TRIM56 PE=1 SV=3	0,260960074	1,169548
O14908	PDZ domain-containing protein GIPC1 OS=Homo sapiens OX=9606 GN=GIPC1 PE=1 SV=2	-0,116631568	1,168907
Q96PU4	E3 ubiquitin-protein ligase UHRF2 OS=Homo sapiens OX=9606 GN=UHRF2 PE=1 SV=1	-0,500329249	1,168749
Q92870	Amyloid-beta A4 precursor protein-binding family B member 2 OS=Homo sapiens OX=9606 GN=APBB2 PE=1 SV=3	-0,375747886	1,168478
Q69YN4	Protein virilizer homolog OS=Homo sapiens OX=9606 GN=VIRMA PE=1 SV=2	-0,22484166	1,167196
Q9NZZ3	Charged multivesicular body protein 5 OS=Homo sapiens OX=9606 GN=CHMP5 PE=1 SV=1	-0,224610948	1,165798
Q9BW62	Katanin p60 ATPase-containing subunit A-like 1 OS=Homo sapiens OX=9606 GN=KATNAL1 PE=1 SV=1	0,169732946	1,165326

Q99986	Serine/threonine-protein kinase VRK1 OS=Homo sapiens OX=9606 GN=VRK1 PE=1 SV=1	0,231646615	1,164176
Q9H497	Torsin-3A OS=Homo sapiens OX=9606 GN=TOR3A PE=1 SV=1	-0,497906469	1,163819
P42696	RNA-binding protein 34 OS=Homo sapiens OX=9606 GN=RBM34 PE=1 SV=2	0,497638715	1,163273
P09211	Glutathione S-transferase P OS=Homo sapiens OX=9606 GN=GSTP1 PE=1 SV=2	0,135438163	1,162902
Q9BST9	Rhotekin OS=Homo sapiens OX=9606 GN=RTKN PE=1 SV=2	-0,278838833	1,161624
O60506	Heterogeneous nuclear ribonucleoprotein Q OS=Homo sapiens OX=9606 GN=SYNCRIP PE=1 SV=2	-0,112331845	1,16087
Q9H8S9	MOB kinase activator 1A OS=Homo sapiens OX=9606 GN=MOB1A PE=1 SV=4	-0,158286778	1,160271
Q9UJY4	ADP-ribosylation factor-binding protein GGA2 OS=Homo sapiens OX=9606 GN=GGA2 PE=1 SV=3	0,182573625	1,160175
P22695	Cytochrome b-c1 complex subunit 2, mitochondrial OS=Homo sapiens OX=9606 GN=UQCRC2 PE=1 SV=3	-0,129478129	1,160173
Q9ULR0	Pre-mRNA-splicing factor ISY1 homolog OS=Homo sapiens OX=9606 GN=ISY1 PE=1 SV=3	-0,321652901	1,159831
O75955	Flotillin-1 OS=Homo sapiens OX=9606 GN=FLOT1 PE=1 SV=3	-0,135116029	1,159505
Q3SXM5	Inactive hydroxysteroid dehydrogenase-like protein 1 OS=Homo sapiens OX=9606 GN=HSDL1 PE=1 SV=3	0,182470176	1,159386
O00151	PDZ and LIM domain protein 1 OS=Homo sapiens OX=9606 GN=PDLIM1 PE=1 SV=4	0,129397296	1,159282
Q86W25	NACHT, LRR and PYD domains-containing protein 13 OS=Homo sapiens OX=9606 GN=NLRP13 PE=2 SV=2	-0,208851056	1,158071
Q7L8L6	FAST kinase domain-containing protein 5, mitochondrial OS=Homo sapiens OX=9606 GN=FASTKD5 PE=1 SV=1	-0,258571011	1,157417
P15408	Fos-related antigen 2 OS=Homo sapiens OX=9606 GN=FOSL2 PE=1 SV=1	0,494707474	1,157292
Q9BV73	Centrosome-associated protein CEP250 OS=Homo sapiens OX=9606 GN=CEP250 PE=1 SV=2	-0,293739481	1,157035
P13073	Cytochrome c oxidase subunit 4 isoform 1, mitochondrial OS=Homo sapiens OX=9606 GN=COX4I1 PE=1 SV=1	-0,181998215	1,155791
Q92692	Nectin-2 OS=Homo sapiens OX=9606 GN=NECTIN2 PE=1 SV=1	0,320460206	1,155243
P53041	Serine/threonine-protein phosphatase 5 OS=Homo sapiens OX=9606 GN=PPP5C PE=1 SV=1	-0,128963738	1,154502
P11532	Dystrophin OS=Homo sapiens OX=9606 GN=DMD PE=1 SV=3	-0,222717125	1,154336
Q16740	ATP-dependent Clp protease proteolytic subunit, mitochondrial OS=Homo sapiens OX=9606 GN=CLPP PE=1 SV=1	0,181725904	1,153717
Q9UJX5	Anaphase-promoting complex subunit 4 OS=Homo sapiens OX=9606 GN=ANAPC4 PE=1 SV=2	-0,319898639	1,153082
P15121	Aldo-keto reductase family 1 member B1 OS=Homo sapiens OX=9606 GN=AKR1B1 PE=1 SV=3	0,128788897	1,152576
P31689	DnaJ homolog subfamily A member 1 OS=Homo sapiens OX=9606 GN=DNAJA1 PE=1 SV=2	0,119305035	1,152363
Q6NXE6	Armadillo repeat-containing protein 6 OS=Homo sapiens OX=9606 GN=ARMC6 PE=1 SV=2	-0,168130531	1,152043
Q6PL18	ATPase family AAA domain-containing protein 2 OS=Homo sapiens OX=9606 GN=ATAD2 PE=1 SV=1	-0,222164785	1,150997
Q9BWH6	RNA polymerase II-associated protein 1 OS=Homo sapiens OX=9606 GN=RPAP1 PE=1 SV=3	0,319309079	1,150813
O43663	Protein regulator of cytokinesis 1 OS=Homo sapiens OX=9606 GN=PRC1 PE=1 SV=2	0,222066847	1,150405
P12004	Proliferating cell nuclear antigen OS=Homo sapiens OX=9606 GN=PCNA PE=1 SV=1	-0,119055947	1,149393
P84077	ADP-ribosylation factor 1 OS=Homo sapiens OX=9606 GN=ARF1 PE=1 SV=2	0,221858616	1,149146
Q9BWM7	Sideroflexin-3 OS=Homo sapiens OX=9606 GN=SFXN3 PE=1 SV=3	0,123459683	1,148854
Q9UBR2	Cathepsin Z OS=Homo sapiens OX=9606 GN=CTSZ PE=1 SV=1	-0,167723461	1,148674
P19338	Nucleolin OS=Homo sapiens OX=9606 GN=NCL PE=1 SV=3	-0,08435336	1,148377
Q6UB35	Monofunctional C1-tetrahydrofolate synthase, mitochondrial OS=Homo sapiens OX=9606 GN=MTHFD1L PE=1 SV=1	-0,198154331	1,147804
Q01167	Forkhead box protein K2 OS=Homo sapiens OX=9606 GN=FOXK2 PE=1 SV=3	-0,489227811	1,146068
P27707	Deoxycytidine kinase OS=Homo sapiens OX=9606 GN=DCK PE=1 SV=1	-0,221128085	1,144733
Q6P1N0	Coiled-coil and C2 domain-containing protein 1A OS=Homo sapiens OX=9606 GN=CC2D1A PE=1 SV=1	-0,111015819	1,144053
P04004	Vitronectin OS=Homo sapiens OX=9606 GN=VTN PE=1 SV=1	0,853583928	1,14396
Q9HB71	Calcyclin-binding protein OS=Homo sapiens OX=9606 GN=CACYBP PE=1 SV=2	0,127992207	1,143812
Q9UIF8	Bromodomain adjacent to zinc finger domain protein 2B OS=Homo sapiens OX=9606 GN=BAZ2B PE=1 SV=3	0,294244217	1,143786
Q56P03	E2F-associated phosphoprotein OS=Homo sapiens OX=9606 GN=EAPP PE=1 SV=4	-0,488023243	1,143592
O15228	Dihydroxyacetone phosphate acyltransferase OS=Homo sapiens OX=9606 GN=GNPAT PE=1 SV=1	-0,118562165	1,143512
P00558	Phosphoglycerate kinase 1 OS=Homo sapiens OX=9606 GN=PGK1 PE=1 SV=3	-0,090750283	1,143418
A0MZ66	Shootin-1 OS=Homo sapiens OX=9606 GN=SHTN1 PE=1 SV=4	0,255802367	1,143373
Q13501	Sequestosome-1 OS=Homo sapiens OX=9606 GN=SQSTM1 PE=1 SV=1	-0,110873178	1,142234
Q86Y79	Probable peptidyl-tRNA hydrolase OS=Homo sapiens OX=9606 GN=PTRH1 PE=1 SV=1	-0,487268234	1,14204
Q5JVF3	PCI domain-containing protein 2 OS=Homo sapiens OX=9606 GN=PCID2 PE=1 SV=2	-0,118424631	1,141875
P82932	28S ribosomal protein S6, mitochondrial OS=Homo sapiens OX=9606 GN=MRPS6 PE=1 SV=3	0,220616379	1,141643
Q9UL63	Muskelin OS=Homo sapiens OX=9606 GN=MKLN1 PE=1 SV=2	-0,486682802	1,140835
Q9NVD7	Alpha-parvin OS=Homo sapiens OX=9606 GN=PARVA PE=1 SV=1	-0,133322031	1,140636
Q8IWB1	Inositol 1,4,5-trisphosphate receptor-interacting protein OS=Homo sapiens OX=9606 GN=ITPRIP PE=1 SV=1	0,255248229	1,140564
Q5VWZ2	Lysophospholipase-like protein 1 OS=Homo sapiens OX=9606 GN=LYPLAL1 PE=1 SV=3	-0,254768141	1,138131
Q14938	Nuclear factor 1 X-type OS=Homo sapiens OX=9606 GN=NFIX PE=1 SV=2	0,315555532	1,136364
Q9UBB6	Neurochondrin OS=Homo sapiens OX=9606 GN=NCDN PE=1 SV=1	-0,315549335	1,136341
Q9NVZ3	Adaptin ear-binding coat-associated protein 2 OS=Homo sapiens OX=9606 GN=NECAP2 PE=1 SV=1	0,219723297	1,136254
Q15047	Histone-lysine N-methyltransferase SETDB1 OS=Homo sapiens OX=9606 GN=SETDB1 PE=1 SV=1	-0,483727029	1,134742
O75494	Serine/arginine-rich splicing factor 10 OS=Homo sapiens OX=9606 GN=SRSF10 PE=1 SV=1	-0,254060155	1,134544
P20042	Eukaryotic translation initiation factor 2 subunit 2 OS=Homo sapiens OX=9606 GN=EIF2S2 PE=1 SV=2	-0,106803549	1,131887
O95487	Protein transport protein Sec24B OS=Homo sapiens OX=9606 GN=SEC24B PE=1 SV=2	-0,126896932	1,131795
Q8NBA8	DTW domain-containing protein 2 OS=Homo sapiens OX=9606 GN=DTWD2 PE=1 SV=1	-0,471542364	1,130357
Q15139	Serine/threonine-protein kinase D1 OS=Homo sapiens OX=9606 GN=PRKD1 PE=1 SV=2	-0,265519624	1,129934
O96013	Serine/threonine-protein kinase PAK 4 OS=Homo sapiens OX=9606 GN=PAK4 PE=1 SV=1	-0,165283033	1,12853
O75351	Vacuolar protein sorting-associated protein 4B OS=Homo sapiens OX=9606 GN=VPS4B PE=1 SV=2	0,138464278	1,127721
Q06136	3-ketodihydrosphingosine reductase OS=Homo sapiens OX=9606 GN=KDSR PE=1 SV=1	0,313275428	1,127583
P58546	Myotrophin OS=Homo sapiens OX=9606 GN=MTPN PE=1 SV=2	-0,178215133	1,127061
P07910	Heterogeneous nuclear ribonucleoproteins C1/C2 OS=Homo sapiens OX=9606 GN=HNRNPC PE=1 SV=4	0,117146038	1,126695
P50749	Ras association domain-containing protein 2 OS=Homo sapiens OX=9606 GN=RASSF2 PE=1 SV=1	-0,312721773	1,12545
Q14116	Interleukin-18 OS=Homo sapiens OX=9606 GN=IL18 PE=1 SV=1	0,217901571	1,125273
Q8TCJ2	Dolichyl-diphosphooligosaccharide--protein glycosyltransferase subunit STT3B OS=Homo sapiens OX=9606 GN=STT3B PE=	-0,131714502	1,123803
E9PRG8	Uncharacterized protein C11orf98 OS=Homo sapiens OX=9606 GN=C11orf98 PE=4 SV=2	-0,477956972	1,1228
O60841	Eukaryotic translation initiation factor 5B OS=Homo sapiens OX=9606 GN=EIF5B PE=1 SV=4	0,077511004	1,122571
Q9P2W9	Syntaxin-18 OS=Homo sapiens OX=9606 GN=STX18 PE=1 SV=1	-0,476917295	1,120641
Q9UMZ2	Synergin gamma OS=Homo sapiens OX=9606 GN=SYNRG PE=1 SV=2	-0,217048323	1,120136
Q05639	Elongation factor 1-alpha 2 OS=Homo sapiens OX=9606 GN=EEF1A2 PE=1 SV=1	0,093153221	1,119451
O15254	Peroxisomal acyl-coenzyme A oxidase 3 OS=Homo sapiens OX=9606 GN=ACOX3 PE=1 SV=2	-0,476138058	1,119022
Q9BZC7	ATP-binding cassette sub-family A member 2 OS=Homo sapiens OX=9606 GN=ABCA2 PE=1 SV=4	-0,475699946	1,118111
Q16610	Extracellular matrix protein 1 OS=Homo sapiens OX=9606 GN=ECM1 PE=1 SV=2	0,100079988	1,118105
Q6NZY4	Zinc finger CCHC domain-containing protein 8 OS=Homo sapiens OX=9606 GN=ZCCHC8 PE=1 SV=2	-0,250596994	1,117013
P08581	Hepatocyte growth factor receptor OS=Homo sapiens OX=9606 GN=MET PE=1 SV=4	-0,474274866	1,115146
P09104	Gamma-enolase OS=Homo sapiens OX=9606 GN=ENO2 PE=1 SV=3	0,137140197	1,114566
O15143	Actin-related protein 2/3 complex subunit 1B OS=Homo sapiens OX=9606 GN=ARPC1B PE=1 SV=3	-0,11610929	1,11443
P51692	Signal transducer and activator of transcription 5B OS=Homo sapiens OX=9606 GN=STAT5B PE=1 SV=2	-0,17653778	1,114375
P67812	Signal peptidase complex catalytic subunit SEC11A OS=Homo sapiens OX=9606 GN=SEC11A PE=1 SV=1	0,193175468	1,113703
Q9UNX3	60S ribosomal protein L26-like 1 OS=Homo sapiens OX=9606 GN=RPL26L1 PE=1 SV=1	-0,151022942	1,11276
Q53EL6	Programmed cell death protein 4 OS=Homo sapiens OX=9606 GN=PDCD4 PE=1 SV=2	0,473064059	1,112623
Q9UNY4	Transcription termination factor 2 OS=Homo sapiens OX=9606 GN=TTF2 PE=1 SV=2	-0,472822781	1,11212
Q86YN1	Dolichyldiphosphatase 1 OS=Homo sapiens OX=9606 GN=DOLPP1 PE=2 SV=1	-0,326147991	1,112063
Q9BYD3	39S ribosomal protein L4, mitochondrial OS=Homo sapiens OX=9606 GN=MRPL4 PE=1 SV=1	0,192829778	1,111343
Q92820	Gamma-glutamyl hydrolase OS=Homo sapiens OX=9606 GN=GGH PE=1 SV=2	-0,192561267	1,109511
O60563	Cyclin-T1 OS=Homo sapiens OX=9606 GN=CCNT1 PE=1 SV=1	0,308523951	1,109275

O43865	S-adenosylhomocysteine hydrolase-like protein 1 OS=Homo sapiens OX=9606 GN=AHCYL1 PE=1 SV=2	0,162872103	1,108715
Q9H936	Mitochondrial glutamate carrier 1 OS=Homo sapiens OX=9606 GN=SLC25A22 PE=1 SV=1	-0,162804165	1,108158
Q96K21	Abscission/NoCut checkpoint regulator OS=Homo sapiens OX=9606 GN=ZFYVE19 PE=1 SV=3	-0,470659896	1,107606
O75882	Attractin OS=Homo sapiens OX=9606 GN=ATRN PE=1 SV=2	0,364081889	1,107071
Q6DKK2	Tetratricopeptide repeat protein 19, mitochondrial OS=Homo sapiens OX=9606 GN=TTC19 PE=1 SV=4	-0,377169052	1,1065
Q5SWX8	Protein odr-4 homolog OS=Homo sapiens OX=9606 GN=ODR4 PE=1 SV=1	0,469988646	1,106204
P12429	Annexin A3 OS=Homo sapiens OX=9606 GN=ANXA3 PE=1 SV=3	0,108002909	1,105832
Q9HDC9	Adipocyte plasma membrane-associated protein OS=Homo sapiens OX=9606 GN=APMAP PE=1 SV=2	-0,115323858	1,105164
Q9GZP4	PITH domain-containing protein 1 OS=Homo sapiens OX=9606 GN=PITHD1 PE=1 SV=1	-0,307356122	1,104773
P33764	Protein S100-A3 OS=Homo sapiens OX=9606 GN=S100A3 PE=1 SV=1	0,469199812	1,104554
Q9Y4D7	Plexin-D1 OS=Homo sapiens OX=9606 GN=PLXND1 PE=1 SV=3	0,111366416	1,104097
Q9UBM7	7-dehydrocholesterol reductase OS=Homo sapiens OX=9606 GN=DHCR7 PE=1 SV=1	0,191704415	1,103667
Q49AR2	UPF0489 protein C5orf22 OS=Homo sapiens OX=9606 GN=C5orf22 PE=1 SV=2	-0,468716881	1,103543
Q13609	Deoxyribonuclease gamma OS=Homo sapiens OX=9606 GN=DNASE1L3 PE=1 SV=1	-1,433977102	1,103146
Q86W92	Liprin-beta-1 OS=Homo sapiens OX=9606 GN=PPFIBP1 PE=1 SV=2	-0,081641327	1,102611
P35080	Profilin-2 OS=Homo sapiens OX=9606 GN=PFN2 PE=1 SV=3	0,191375877	1,101429
P0DN79	Cystathionine beta-synthase-like protein OS=Homo sapiens OX=9606 GN=CBSL PE=1 SV=1	0,306485193	1,101416
Q8N584	Tetratricopeptide repeat protein 39C OS=Homo sapiens OX=9606 GN=TTC39C PE=2 SV=2	0,467258692	1,10049
Q07817	Bcl-2-like protein 1 OS=Homo sapiens OX=9606 GN=BCL2L1 PE=1 SV=1	0,306153826	1,100138
P08240	Signal recognition particle receptor subunit alpha OS=Homo sapiens OX=9606 GN=SRPRA PE=1 SV=2	-0,096291956	1,100049
O95810	Caveolae-associated protein 2 OS=Homo sapiens OX=9606 GN=CAVIN2 PE=1 SV=3	-0,110976658	1,099344
Q96FN4	Copine-2 OS=Homo sapiens OX=9606 GN=CPNE2 PE=1 SV=3	-0,305869533	1,099042
Q96FZ7	Charged multivesicular body protein 6 OS=Homo sapiens OX=9606 GN=CHMP6 PE=1 SV=3	0,305845156	1,098948
P22392	Nucleoside diphosphate kinase B OS=Homo sapiens OX=9606 GN=NME2 PE=1 SV=1	-0,246868769	1,098171
Q9NQX3	Gephyrin OS=Homo sapiens OX=9606 GN=GPHN PE=1 SV=1	0,213366061	1,098012
Q9NYS0	NF-kappa-B inhibitor-interacting Ras-like protein 1 OS=Homo sapiens OX=9606 GN=NKIRAS1 PE=1 SV=1	0,46595081	1,097747
P23786	Carnitine O-palmitoyltransferase 2, mitochondrial OS=Homo sapiens OX=9606 GN=CPT2 PE=1 SV=2	-0,174281281	1,097361
O95989	Diphosphoinositol polyphosphate phosphohydrolase 1 OS=Homo sapiens OX=9606 GN=NUDT3 PE=1 SV=1	0,608009499	1,097251
P07954	Fumarate hydratase, mitochondrial OS=Homo sapiens OX=9606 GN=FH PE=1 SV=3	-0,089589704	1,096177
Q96D71	RalBP1-associated Eps domain-containing protein 1 OS=Homo sapiens OX=9606 GN=REPS1 PE=1 SV=3	-0,213010295	1,095879
O60645	Exocyst complex component 3 OS=Homo sapiens OX=9606 GN=EXOC3 PE=1 SV=3	0,19049985	1,095463
Q14790	Caspase-8 OS=Homo sapiens OX=9606 GN=CASP8 PE=1 SV=1	-0,123562746	1,095437
Q13112	Chromatin assembly factor 1 subunit B OS=Homo sapiens OX=9606 GN=CHAF1B PE=1 SV=1	-0,304655273	1,09436
Q8TB22	Spermatogenesis-associated protein 20 OS=Homo sapiens OX=9606 GN=SPATA20 PE=2 SV=3	-0,464271403	1,094221
Q9UKM7	Endoplasmic reticulum mannosyl-oligosaccharide 1,2-alpha-mannosidase OS=Homo sapiens OX=9606 GN=MAN1B1 PE=1	0,142289145	1,094143
P61966	AP-1 complex subunit sigma-1A OS=Homo sapiens OX=9606 GN=AP1S1 PE=1 SV=1	-0,245907578	1,093318
Q8WVJ2	NudC domain-containing protein 2 OS=Homo sapiens OX=9606 GN=NUDCD2 PE=1 SV=1	-0,212580643	1,093303
Q9HD20	Manganese-transporting ATPase 13A1 OS=Homo sapiens OX=9606 GN=ATP13A1 PE=1 SV=2	-0,095811896	1,093275
Q9Y5L4	Mitochondrial import inner membrane translocase subunit Tim13 OS=Homo sapiens OX=9606 GN=TIMM13 PE=1 SV=1	-0,245873751	1,093148
O95249	Golgi SNAP receptor complex member 1 OS=Homo sapiens OX=9606 GN=GOSR1 PE=1 SV=1	0,190011836	1,092143
Q8IVL5	Prolyl 3-hydroxylase 2 OS=Homo sapiens OX=9606 GN=P3H2 PE=1 SV=1	0,280199533	1,090529
Q8NC56	LEM domain-containing protein 2 OS=Homo sapiens OX=9606 GN=LEMD2 PE=1 SV=1	0,160603584	1,09015
Q14166	Tubulin--tyrosine ligase-like protein 12 OS=Homo sapiens OX=9606 GN=TTLL12 PE=1 SV=2	0,118268543	1,089767
Q9NWB6	Arginine and glutamate-rich protein 1 OS=Homo sapiens OX=9606 GN=ARGLU1 PE=1 SV=1	0,303462549	1,08976
Q92552	28S ribosomal protein S27, mitochondrial OS=Homo sapiens OX=9606 GN=MRPS27 PE=1 SV=3	-0,461995651	1,089434
P40227	T-complex protein 1 subunit zeta OS=Homo sapiens OX=9606 GN=CCT6A PE=1 SV=3	0,088977292	1,086913
Q06609	DNA repair protein RAD51 homolog 1 OS=Homo sapiens OX=9606 GN=RAD51 PE=1 SV=1	0,263781136	1,086501
P53582	Methionine aminopeptidase 1 OS=Homo sapiens OX=9606 GN=METAP1 PE=1 SV=2	0,160145678	1,086411
Q96H55	Unconventional myosin-XIX OS=Homo sapiens OX=9606 GN=MYO19 PE=1 SV=2	-0,460352388	1,085971
O00299	Chloride intracellular channel protein 1 OS=Homo sapiens OX=9606 GN=CLIC1 PE=1 SV=4	0,095169591	1,084233
Q9Y623	Myosin-4 OS=Homo sapiens OX=9606 GN=MYH4 PE=2 SV=2	-0,933729096	1,084059
Q8NA72	Centrosomal protein POC5 OS=Homo sapiens OX=9606 GN=POC5 PE=1 SV=2	-0,459355028	1,083867
Q8WWV3	Reticulon-4-interacting protein 1, mitochondrial OS=Homo sapiens OX=9606 GN=RTN4IP1 PE=1 SV=2	-0,459135991	1,083404
O60499	Syntaxin-10 OS=Homo sapiens OX=9606 GN=STX10 PE=1 SV=1	-0,459084133	1,083295
Q9NRN9	Methyltransferase-like protein 5 OS=Homo sapiens OX=9606 GN=METTL5 PE=1 SV=1	0,243844243	1,082909
Q14258	E3 ubiquitin/ISG15 ligase TRIM25 OS=Homo sapiens OX=9606 GN=TRIM25 PE=1 SV=2	-0,088606579	1,081317
O75844	CAAX prenyl protease 1 homolog OS=Homo sapiens OX=9606 GN=ZMPSTE24 PE=1 SV=2	0,122242439	1,081134
P27482	Calmodulin-like protein 3 OS=Homo sapiens OX=9606 GN=CALML3 PE=1 SV=2	0,243447635	1,08091
O75525	#N/D	-0,281408809	1,079792
Q86XL3	Ankyrin repeat and LEM domain-containing protein 2 OS=Homo sapiens OX=9606 GN=ANKLE2 PE=1 SV=4	-0,10267159	1,078107
Q3KQU3	MAP7 domain-containing protein 1 OS=Homo sapiens OX=9606 GN=MAP7D1 PE=1 SV=1	-0,11713619	1,076999
O75352	Mannose-P-dolichol utilization defect 1 protein OS=Homo sapiens OX=9606 GN=MPDU1 PE=1 SV=2	-0,242525252	1,076261
O43156	TELO2-interacting protein 1 homolog OS=Homo sapiens OX=9606 GN=TTI1 PE=1 SV=3	0,14876019	1,076194
Q9BUJ2	Heterogeneous nuclear ribonucleoprotein U-like protein 1 OS=Homo sapiens OX=9606 GN=HNRNPUL1 PE=1 SV=2	-0,084617936	1,075046
Q99719	Septin-5 OS=Homo sapiens OX=9606 GN=SEPTIN5 PE=1 SV=1	0,542497921	1,073579
Q9Y241	HIG1 domain family member 1A, mitochondrial OS=Homo sapiens OX=9606 GN=HIGD1A PE=1 SV=1	0,454233702	1,073032
Q9NZM1	Myoferlin OS=Homo sapiens OX=9606 GN=MYOF PE=1 SV=1	-0,038825913	1,07192
Q86V81	THO complex subunit 4 OS=Homo sapiens OX=9606 GN=ALYREF PE=1 SV=3	-0,132751909	1,071271
O00499	Myc box-dependent-interacting protein 1 OS=Homo sapiens OX=9606 GN=BIN1 PE=1 SV=1	-0,105248035	1,071226
O43396	Thioredoxin-like protein 1 OS=Homo sapiens OX=9606 GN=TXNL1 PE=1 SV=3	-0,126641571	1,071147
P61812	Transforming growth factor beta-2 proprotein OS=Homo sapiens OX=9606 GN=TGFB2 PE=1 SV=1	0,241493284	1,071062
Q8WUP2	Filamin-binding LIM protein 1 OS=Homo sapiens OX=9606 GN=FBLIM1 PE=1 SV=2	-0,170685191	1,070369
Q9ULA0	Aspartyl aminopeptidase OS=Homo sapiens OX=9606 GN=DNPEP PE=1 SV=1	-0,148065066	1,070123
Q99988	Growth/differentiation factor 15 OS=Homo sapiens OX=9606 GN=GDF15 PE=1 SV=3	-0,239704387	1,069891
P08559	Pyruvate dehydrogenase E1 component subunit alpha, somatic form, mitochondrial OS=Homo sapiens OX=9606 GN=PDH	-0,096514674	1,069142
Q8IUF8	Ribosomal oxygenase 2 OS=Homo sapiens OX=9606 GN=RIOX2 PE=1 SV=1	-0,297920033	1,068379
O14602	Eukaryotic translation initiation factor 1A, Y-chromosomal OS=Homo sapiens OX=9606 GN=EIF1AY PE=1 SV=4	0,174987601	1,067848
Q9NWB7	Intraflagellar transport protein 57 homolog OS=Homo sapiens OX=9606 GN=IFT57 PE=1 SV=1	0,675258551	1,067845
O00567	Nucleolar protein 56 OS=Homo sapiens OX=9606 GN=NOP56 PE=1 SV=4	-0,108354838	1,067525
Q66K14	TBC1 domain family member 9B OS=Homo sapiens OX=9606 GN=TBC1D9B PE=1 SV=3	-0,120937677	1,067051
Q15041	ADP-ribosylation factor-like protein 6-interacting protein 1 OS=Homo sapiens OX=9606 GN=ARL6IP1 PE=1 SV=2	-0,451336842	1,066881
Q15643	Thyroid receptor-interacting protein 11 OS=Homo sapiens OX=9606 GN=TRIP11 PE=1 SV=3	0,157637019	1,065987
Q96KR1	Zinc finger RNA-binding protein OS=Homo sapiens OX=9606 GN=ZFR PE=1 SV=2	-0,126020813	1,064752
P09661	U2 small nuclear ribonucleoprotein A' OS=Homo sapiens OX=9606 GN=SNRPA1 PE=1 SV=2	-0,157394059	1,064014
Q13416	Origin recognition complex subunit 2 OS=Homo sapiens OX=9606 GN=ORC2 PE=1 SV=2	-0,185780581	1,063443
Q9BYX2	TBC1 domain family member 2A OS=Homo sapiens OX=9606 GN=TBC1D2 PE=1 SV=3	0,089319332	1,062762
Q96I59	Probable asparagine--tRNA ligase, mitochondrial OS=Homo sapiens OX=9606 GN=NARS2 PE=1 SV=3	-0,29642242	1,0626
P35998	26S proteasome regulatory subunit 7 OS=Homo sapiens OX=9606 GN=PSMC2 PE=1 SV=3	-0,07921634	1,062193
P00338	L-lactate dehydrogenase A chain OS=Homo sapiens OX=9606 GN=LDHA PE=1 SV=2	0,095954585	1,061511
Q9UNZ2	NSFL1 cofactor p47 OS=Homo sapiens OX=9606 GN=NSFL1C PE=1 SV=2	-0,098483734	1,06054
Q9UHY8	Fasciculation and elongation protein zeta-2 OS=Homo sapiens OX=9606 GN=FEZ2 PE=1 SV=2	-0,29558597	1,059371

P10253	Lysosomal alpha-glucosidase OS=Homo sapiens OX=9606 GN=GAA PE=1 SV=4	-0,104109715	1,057023
Q13247	Serine/arginine-rich splicing factor 6 OS=Homo sapiens OX=9606 GN=SRSF6 PE=1 SV=2	-0,115347695	1,056921
Q7Z7F7	39S ribosomal protein L55, mitochondrial OS=Homo sapiens OX=9606 GN=MRPL55 PE=1 SV=1	-0,294853754	1,056545
Q15746	Myosin light chain kinase, smooth muscle OS=Homo sapiens OX=9606 GN=MYLK PE=1 SV=4	-0,09811499	1,055662
O60232	Protein ZNRD2 OS=Homo sapiens OX=9606 GN=ZNRD2 PE=1 SV=1	-0,094926708	1,055084
Q9NZ01	Very-long-chain enoyl-CoA reductase OS=Homo sapiens OX=9606 GN=TECR PE=1 SV=1	-0,138044257	1,05469
P03905	NADH-ubiquinone oxidoreductase chain 4 OS=Homo sapiens OX=9606 GN=MT-ND4 PE=1 SV=1	-0,238108545	1,05403
Q9Y6H1	Coiled-coil-helix-coiled-coil-helix domain-containing protein 2 OS=Homo sapiens OX=9606 GN=CHCHD2 PE=1 SV=1	-0,237899551	1,05298
Q13309	S-phase kinase-associated protein 2 OS=Homo sapiens OX=9606 GN=SKP2 PE=1 SV=2	-0,412038012	1,052058
P55010	Eukaryotic translation initiation factor 5 OS=Homo sapiens OX=9606 GN=EIF5 PE=1 SV=2	0,130699863	1,051186
Q14956	Transmembrane glycoprotein NMB OS=Homo sapiens OX=9606 GN=GPNMB PE=1 SV=2	0,443690219	1,050567
Q9BU76	Multiple myeloma tumor-associated protein 2 OS=Homo sapiens OX=9606 GN=MMTAG2 PE=1 SV=1	-0,293032006	1,049514
Q15058	Kinesin-like protein KIF14 OS=Homo sapiens OX=9606 GN=KIF14 PE=1 SV=1	-0,237189754	1,049412
P13716	Delta-aminolevulinic acid dehydratase OS=Homo sapiens OX=9606 GN=ALAD PE=1 SV=1	0,292961005	1,04924
Q9NZT2	Opioid growth factor receptor OS=Homo sapiens OX=9606 GN=OGFR PE=1 SV=3	-0,110477748	1,0485
Q9Y6C9	Mitochondrial carrier homolog 2 OS=Homo sapiens OX=9606 GN=MTCH2 PE=1 SV=1	-0,130403285	1,048292
Q6UVK1	Chondroitin sulfate proteoglycan 4 OS=Homo sapiens OX=9606 GN=CSPG4 PE=1 SV=2	-0,050769187	1,047886
Q13308	Inactive tyrosine-protein kinase 7 OS=Homo sapiens OX=9606 GN=PTK7 PE=1 SV=2	-0,086328664	1,04713
Q86YH6	Decaprenyl-diphosphate synthase subunit 2 OS=Homo sapiens OX=9606 GN=PDSS2 PE=1 SV=2	0,442016533	1,046981
Q86SX6	Glutaredoxin-related protein 5, mitochondrial OS=Homo sapiens OX=9606 GN=GLRX5 PE=1 SV=2	-1,184727832	1,046387
P54819	Adenylate kinase 2, mitochondrial OS=Homo sapiens OX=9606 GN=AK2 PE=1 SV=2	0,130202303	1,046332
P50452	Serpin B8 OS=Homo sapiens OX=9606 GN=SERPINB8 PE=1 SV=2	-0,204618356	1,045753
P49207	60S ribosomal protein L34 OS=Homo sapiens OX=9606 GN=RPL34 PE=1 SV=3	-0,183155422	1,045715
Q9GZT3	SRA stem-loop-interacting RNA-binding protein, mitochondrial OS=Homo sapiens OX=9606 GN=SLIRP PE=1 SV=1	-0,291771354	1,044648
P68431	Histone H3.1 OS=Homo sapiens OX=9606 GN=HIST1H3A PE=1 SV=2	-0,440667705	1,044088
P35573	Glycogen debranching enzyme OS=Homo sapiens OX=9606 GN=AGL PE=1 SV=3	-0,182758965	1,043043
Q7Z2Z2	Elongation factor-like GTPase 1 OS=Homo sapiens OX=9606 GN=EFL1 PE=1 SV=2	-0,291268857	1,042708
Q14331	Protein FRG1 OS=Homo sapiens OX=9606 GN=FRG1 PE=1 SV=1	-0,154686158	1,042083
Q2M2I8	AP2-associated protein kinase 1 OS=Homo sapiens OX=9606 GN=AAK1 PE=1 SV=3	0,136436704	1,039849
O75976	Carboxypeptidase D OS=Homo sapiens OX=9606 GN=CPD PE=1 SV=2	0,23524203	1,03963
Q13895	Bystin OS=Homo sapiens OX=9606 GN=BYSL PE=1 SV=3	-0,166555915	1,039565
Q9Y446	Plakophilin-3 OS=Homo sapiens OX=9606 GN=PKP3 PE=1 SV=1	-0,438286874	1,038972
P22087	rRNA 2'-O-methyltransferase fibrillarin OS=Homo sapiens OX=9606 GN=FBL PE=1 SV=2	-0,136217792	1,037832
Q9BXL7	Caspase recruitment domain-containing protein 11 OS=Homo sapiens OX=9606 GN=CARD11 PE=1 SV=3	-0,437170022	1,036569
Q58WW2	DDB1- and CUL4-associated factor 6 OS=Homo sapiens OX=9606 GN=DCAF6 PE=1 SV=1	-0,293577315	1,036278
Q9BYE7	Polycomb group RING finger protein 6 OS=Homo sapiens OX=9606 GN=PCGF6 PE=1 SV=2	-0,436996464	1,036195
Q9BXJ9	N-alpha-acetyltransferase 15, NatA auxiliary subunit OS=Homo sapiens OX=9606 GN=NAA15 PE=1 SV=1	-0,074853335	1,03322
P31483	Nucleolysin TIA-1 isoform p40 OS=Homo sapiens OX=9606 GN=TIA1 PE=1 SV=3	-0,435233136	1,032395
O00487	26S proteasome non-ATPase regulatory subunit 14 OS=Homo sapiens OX=9606 GN=PSMD14 PE=1 SV=1	-0,128749805	1,032196
P18846	Cyclic AMP-dependent transcription factor ATF-1 OS=Homo sapiens OX=9606 GN=ATF1 PE=1 SV=2	-0,082426015	1,032131
Q29RF7	Sister chromatid cohesion protein PDS5 homolog A OS=Homo sapiens OX=9606 GN=PDS5A PE=1 SV=1	-0,109017337	1,031594
Q9Y5A7	NEDD8 ultimate buster 1 OS=Homo sapiens OX=9606 GN=NUB1 PE=1 SV=2	-0,202123962	1,030931
Q12769	Nuclear pore complex protein Nup160 OS=Homo sapiens OX=9606 GN=NUP160 PE=1 SV=3	-0,091342315	1,030831
Q15714	TSC22 domain family protein 1 OS=Homo sapiens OX=9606 GN=TSC22D1 PE=1 SV=3	0,434866479	1,030664
Q86X76	Deaminated glutathione amidase OS=Homo sapiens OX=9606 GN=NIT1 PE=1 SV=2	0,233224768	1,029509
P35222	Catenin beta-1 OS=Homo sapiens OX=9606 GN=CTNNB1 PE=1 SV=1	0,077186465	1,028724
P35442	Thrombospondin-2 OS=Homo sapiens OX=9606 GN=THBS2 PE=1 SV=2	0,433424449	1,02849
Q8WVQ1	Soluble calcium-activated nucleotidase 1 OS=Homo sapiens OX=9606 GN=CANT1 PE=1 SV=1	-0,471833459	1,027895
Q96BJ3	Axin interactor, dorsalization-associated protein OS=Homo sapiens OX=9606 GN=AIDA PE=1 SV=1	-0,128288194	1,027714
Q9P253	Vacuolar protein sorting-associated protein 18 homolog OS=Homo sapiens OX=9606 GN=VPS18 PE=1 SV=2	-0,180334596	1,026735
Q7Z7E8	Ubiquitin-conjugating enzyme E2 Q1 OS=Homo sapiens OX=9606 GN=UBE2Q1 PE=1 SV=1	-0,133892891	1,025751
Q5MIZ7	Serine/threonine-protein phosphatase 4 regulatory subunit 3B OS=Homo sapiens OX=9606 GN=PPP4R3B PE=1 SV=2	0,431904268	1,025204
Q9UL15	BAG family molecular chaperone regulator 5 OS=Homo sapiens OX=9606 GN=BAG5 PE=1 SV=1	-0,286520799	1,024377
P37198	Nuclear pore glycoprotein p62 OS=Homo sapiens OX=9606 GN=NUP62 PE=1 SV=3	-0,152396843	1,02363
Q15126	Phosphomevalonate kinase OS=Homo sapiens OX=9606 GN=PMVK PE=1 SV=3	-0,127696337	1,021976
Q9BXU1	Serine/threonine-protein kinase 31 OS=Homo sapiens OX=9606 GN=STK31 PE=2 SV=2	0,058143287	1,021974
Q04721	Neurogenic locus notch homolog protein 2 OS=Homo sapiens OX=9606 GN=NOTCH2 PE=1 SV=3	-0,231683501	1,021784
P22314	Ubiquitin-like modifier-activating enzyme 1 OS=Homo sapiens OX=9606 GN=UBA1 PE=1 SV=3	-0,056927672	1,020527
Q86XN8	RNA-binding protein MEX3D OS=Homo sapiens OX=9606 GN=MEX3D PE=1 SV=3	-0,429717207	1,020469
O14925	Mitochondrial import inner membrane translocase subunit Tim23 OS=Homo sapiens OX=9606 GN=TIMM23 PE=1 SV=1	-0,429619974	1,020258
P78560	Death domain-containing protein CRADD OS=Homo sapiens OX=9606 GN=CRADD PE=1 SV=1	-0,285211315	1,01932
Q9NQY0	Bridging integrator 3 OS=Homo sapiens OX=9606 GN=BIN3 PE=1 SV=1	-0,285091555	1,018858
Q9Y6D6	Brefeldin A-inhibited guanine nucleotide-exchange protein 1 OS=Homo sapiens OX=9606 GN=ARFGEF1 PE=1 SV=2	0,15441478	1,018134
Q96N11	Uncharacterized protein C7orf26 OS=Homo sapiens OX=9606 GN=C7orf26 PE=2 SV=1	-0,140733453	1,018125
P0C0S5	Histone H2A.Z OS=Homo sapiens OX=9606 GN=H2AFZ PE=1 SV=2	-0,230912911	1,017924
Q9HBH5	Retinol dehydrogenase 14 OS=Homo sapiens OX=9606 GN=RDH14 PE=1 SV=1	-0,199877035	1,017611
Q13287	N-myc-interactor OS=Homo sapiens OX=9606 GN=NMI PE=1 SV=2	0,199871959	1,017581
Q92759	General transcription factor IIH subunit 4 OS=Homo sapiens OX=9606 GN=GTF2H4 PE=1 SV=1	0,230779884	1,017258
Q32P41	tRNA (guanine(37)-N1)-methyltransferase OS=Homo sapiens OX=9606 GN=TRMT5 PE=1 SV=2	-0,284519138	1,016648
Q9UL03	Integrator complex subunit 6 OS=Homo sapiens OX=9606 GN=INTS6 PE=1 SV=1	-0,427791723	1,016292
Q9NQC3	Reticulon-4 OS=Homo sapiens OX=9606 GN=RTN4 PE=1 SV=2	-0,141819297	1,015971
Q9NRF8	CTP synthase 2 OS=Homo sapiens OX=9606 GN=CTPS2 PE=1 SV=1	0,133831714	1,015918
Q96ND0	Protein FAM210A OS=Homo sapiens OX=9606 GN=FAM210A PE=1 SV=2	0,427348007	1,015328
P09110	3-ketoacyl-CoA thiolase, peroxisomal OS=Homo sapiens OX=9606 GN=ACAA1 PE=1 SV=2	0,151302124	1,014834
P62906	60S ribosomal protein L10a OS=Homo sapiens OX=9606 GN=RPL10A PE=1 SV=2	-0,133651615	1,014269
Q96Q15	Serine/threonine-protein kinase SMG1 OS=Homo sapiens OX=9606 GN=SMG1 PE=1 SV=3	0,230040424	1,013556
Q8WUH6	Transmembrane protein 263 OS=Homo sapiens OX=9606 GN=TMEM263 PE=1 SV=1	-0,283689269	1,013443
Q9UL25	Ras-related protein Rab-21 OS=Homo sapiens OX=9606 GN=RAB21 PE=1 SV=3	-0,141511436	1,01332
P15170	Eukaryotic peptide chain release factor GTP-binding subunit ERF3A OS=Homo sapiens OX=9606 GN=GSPT1 PE=1 SV=1	-0,100569442	1,013209
Q8TAF3	WD repeat-containing protein 48 OS=Homo sapiens OX=9606 GN=WDR48 PE=1 SV=1	0,198911589	1,011897
Q9UQB8	Brain-specific angiogenesis inhibitor 1-associated protein 2 OS=Homo sapiens OX=9606 GN=BAIAP2 PE=1 SV=1	0,080469039	1,010502
Q969Q6	Serine/threonine-protein phosphatase 2A regulatory subunit B'' subunit gamma OS=Homo sapiens OX=9606 GN=PPP2R3(	-0,424757974	1,009697
Q13404	Ubiquitin-conjugating enzyme E2 variant 1 OS=Homo sapiens OX=9606 GN=UBE2V1 PE=1 SV=2	-0,150634185	1,009476
O00422	Histone deacetylase complex subunit SAP18 OS=Homo sapiens OX=9606 GN=SAP18 PE=1 SV=1	0,162390316	1,0087
Q13951	Core-binding factor subunit beta OS=Homo sapiens OX=9606 GN=CBFB PE=1 SV=2	-0,177485553	1,007638
P50542	Peroxisomal targeting signal 1 receptor OS=Homo sapiens OX=9606 GN=PEX5 PE=1 SV=3	-0,423761706	1,007527
P51617	Interleukin-1 receptor-associated kinase 1 OS=Homo sapiens OX=9606 GN=IRAK1 PE=1 SV=2	-0,177447856	1,007385
O95202	Mitochondrial proton/calcium exchanger protein OS=Homo sapiens OX=9606 GN=LETM1 PE=1 SV=1	0,106908808	1,007325
Q06830	Peroxiredoxin-1 OS=Homo sapiens OX=9606 GN=PRDX1 PE=1 SV=1	0,106898706	1,007209
Q9Y4I1	Unconventional myosin-Va OS=Homo sapiens OX=9606 GN=MYO5A PE=1 SV=2	-0,177220301	1,005863
O94955	Rho-related BTB domain-containing protein 3 OS=Homo sapiens OX=9606 GN=RHOBTB3 PE=1 SV=2	0,281566347	1,005246

P02794	Ferritin heavy chain OS=Homo sapiens OX=9606 GN=FTH1 PE=1 SV=2	0,161800292	1,004345
Q14019	Coactosin-like protein OS=Homo sapiens OX=9606 GN=COTL1 PE=1 SV=3	-0,14996866	1,004145
P23381	Tryptophan--tRNA ligase, cytoplasmic OS=Homo sapiens OX=9606 GN=WARS PE=1 SV=2	-0,09980057	1,003765
Q13425	Beta-2-syntrophin OS=Homo sapiens OX=9606 GN=SNTB2 PE=1 SV=1	-0,176841567	1,003331
Q96B49	Mitochondrial import receptor subunit TOM6 homolog OS=Homo sapiens OX=9606 GN=TOMM6 PE=1 SV=1	0,421298956	1,002156
P40763	Signal transducer and activator of transcription 3 OS=Homo sapiens OX=9606 GN=STAT3 PE=1 SV=2	0,069637449	1,00202
Q9NRY6	Phospholipid scramblase 3 OS=Homo sapiens OX=9606 GN=PLSCR3 PE=1 SV=2	-0,421118064	1,001761
Q9UIA9	Exportin-7 OS=Homo sapiens OX=9606 GN=XPO7 PE=1 SV=3	-0,083232764	1,001209
O14880	Microsomal glutathione S-transferase 3 OS=Homo sapiens OX=9606 GN=MGST3 PE=1 SV=1	0,280372861	1,000637
Q13011	Delta(3,5)-Delta(2,4)-dienoyl-CoA isomerase, mitochondrial OS=Homo sapiens OX=9606 GN=ECH1 PE=1 SV=2	0,139910462	0,999564
Q12846	Syntaxin-4 OS=Homo sapiens OX=9606 GN=STX4 PE=1 SV=2	-0,196701196	0,998836
P39748	Flap endonuclease 1 OS=Homo sapiens OX=9606 GN=FEN1 PE=1 SV=1	-0,119557325	0,998802
O15118	NPC intracellular cholesterol transporter 1 OS=Homo sapiens OX=9606 GN=NPC1 PE=1 SV=2	0,176099351	0,998373
P13861	cAMP-dependent protein kinase type II-alpha regulatory subunit OS=Homo sapiens OX=9606 GN=PRKAR2A PE=1 SV=2	-0,106074545	0,997769
O60282	Kinesin heavy chain isoform 5C OS=Homo sapiens OX=9606 GN=KIF5C PE=1 SV=1	-0,149139436	0,997512
Q9C0J8	pre-mRNA 3' end processing protein WDR33 OS=Homo sapiens OX=9606 GN=WDR33 PE=1 SV=2	0,344905312	0,996589
Q96FW1	Ubiquitin thioesterase OTUB1 OS=Homo sapiens OX=9606 GN=OTUB1 PE=1 SV=2	-0,119272227	0,99592
Q9NXC5	GATOR complex protein MIOS OS=Homo sapiens OX=9606 GN=MIOS PE=1 SV=2	-0,417453574	0,993747
O94964	Protein SOGA1 OS=Homo sapiens OX=9606 GN=SOGA1 PE=1 SV=2	-0,148564101	0,992916
Q99496	E3 ubiquitin-protein ligase RING2 OS=Homo sapiens OX=9606 GN=RNF2 PE=1 SV=1	-0,195662047	0,992706
Q96L93	Kinesin-like protein KIF16B OS=Homo sapiens OX=9606 GN=KIF16B PE=1 SV=2	0,069367577	0,992117
P04908	Histone H2A type 1-B/E OS=Homo sapiens OX=9606 GN=HIST1H2AB PE=1 SV=2	0,225748203	0,992098
P61981	14-3-3 protein gamma OS=Homo sapiens OX=9606 GN=YWHAG PE=1 SV=2	0,109490249	0,991925
Q8NEY1	Neuron navigator 1 OS=Homo sapiens OX=9606 GN=NAV1 PE=1 SV=2	0,148359837	0,991285
Q99848	Probable rRNA-processing protein EBP2 OS=Homo sapiens OX=9606 GN=EBNA1BP2 PE=1 SV=2	-0,138807127	0,990112
P50897	Palmitoyl-protein thioesterase 1 OS=Homo sapiens OX=9606 GN=PPT1 PE=1 SV=1	0,174769104	0,989498
O43683	Mitotic checkpoint serine/threonine-protein kinase BUB1 OS=Homo sapiens OX=9606 GN=BUB1 PE=1 SV=1	0,441369408	0,988816
O60763	General vesicular transport factor p115 OS=Homo sapiens OX=9606 GN=USO1 PE=1 SV=2	0,092950652	0,98806
Q96IX5	ATP synthase membrane subunit DAPIT, mitochondrial OS=Homo sapiens OX=9606 GN=ATP5MD PE=1 SV=1	0,414772187	0,987866
Q99798	Aconitate hydratase, mitochondrial OS=Homo sapiens OX=9606 GN=ACO2 PE=1 SV=2	-0,080547599	0,986954
O60869	Endothelial differentiation-related factor 1 OS=Homo sapiens OX=9606 GN=EDF1 PE=1 SV=1	0,138423097	0,986828
Q86VX2	COMM domain-containing protein 7 OS=Homo sapiens OX=9606 GN=COMMD7 PE=1 SV=2	0,414298553	0,986826
P18615	Negative elongation factor E OS=Homo sapiens OX=9606 GN=NELFE PE=1 SV=3	0,130618819	0,986605
Q9NWT1	p21-activated protein kinase-interacting protein 1 OS=Homo sapiens OX=9606 GN=PAK1IP1 PE=1 SV=2	-0,414092377	0,986373
Q6ZSZ5	Rho guanine nucleotide exchange factor 18 OS=Homo sapiens OX=9606 GN=ARHGEF18 PE=1 SV=4	0,276560424	0,985916
P09960	Leukotriene A-4 hydrolase OS=Homo sapiens OX=9606 GN=LTA4H PE=1 SV=2	0,0759159	0,985906
P48556	26S proteasome non-ATPase regulatory subunit 8 OS=Homo sapiens OX=9606 GN=PSMD8 PE=1 SV=2	-0,11330695	0,985744
P19784	Casein kinase II subunit alpha' OS=Homo sapiens OX=9606 GN=CSNK2A2 PE=1 SV=1	-0,174163123	0,985461
Q9BUR5	MICOS complex subunit MIC26 OS=Homo sapiens OX=9606 GN=APOO PE=1 SV=1	-0,276220636	0,984603
P45877	Peptidyl-prolyl cis-trans isomerase C OS=Homo sapiens OX=9606 GN=PPIC PE=1 SV=1	0,275841686	0,98314
Q8NBZ7	UDP-glucuronic acid decarboxylase 1 OS=Homo sapiens OX=9606 GN=UXS1 PE=1 SV=1	-0,626897699	0,982756
Q16204	Coiled-coil domain-containing protein 6 OS=Homo sapiens OX=9606 GN=CCDC6 PE=1 SV=2	0,117889484	0,981974
P20839	Inosine-5'-monophosphate dehydrogenase 1 OS=Homo sapiens OX=9606 GN=IMPDH1 PE=1 SV=2	0,123533796	0,981863
P13693	Translationally-controlled tumor protein OS=Homo sapiens OX=9606 GN=TPT1 PE=1 SV=1	0,137700099	0,980651
Q969X6	U3 small nucleolar RNA-associated protein 4 homolog OS=Homo sapiens OX=9606 GN=UTP4 PE=1 SV=1	-0,411464747	0,980594
Q14203	Dynactin subunit 1 OS=Homo sapiens OX=9606 GN=DCTN1 PE=1 SV=3	0,059961863	0,979984
Q5W0Z9	Palmitoyltransferase ZDHHC20 OS=Homo sapiens OX=9606 GN=ZDHHC20 PE=1 SV=1	0,063404288	0,978993
Q92696	Geranylgeranyl transferase type-2 subunit alpha OS=Homo sapiens OX=9606 GN=RABGGTA PE=1 SV=2	0,274759729	0,978963
Q8WW01	tRNA-splicing endonuclease subunit Sen15 OS=Homo sapiens OX=9606 GN=TSEN15 PE=1 SV=1	-0,410548807	0,978576
P53609	Geranylgeranyl transferase type-1 subunit beta OS=Homo sapiens OX=9606 GN=PGGT1B PE=1 SV=2	-0,410228607	0,977871
Q9NR12	PDZ and LIM domain protein 7 OS=Homo sapiens OX=9606 GN=PDLIM7 PE=1 SV=1	-0,097655991	0,977561
Q12972	Nuclear inhibitor of protein phosphatase 1 OS=Homo sapiens OX=9606 GN=PPP1R8 PE=1 SV=2	-0,273998509	0,976023
P04844	Dolichyl-diphosphooligosaccharide--protein glycosyltransferase subunit 2 OS=Homo sapiens OX=9606 GN=RPN2 PE=1 SV=	0,089579832	0,975824
Q8IYE0	Coiled-coil domain-containing protein 146 OS=Homo sapiens OX=9606 GN=CCDC146 PE=1 SV=2	0,514639249	0,973905
Q14108	Lysosome membrane protein 2 OS=Homo sapiens OX=9606 GN=SCARB2 PE=1 SV=2	-0,129151174	0,973289
Q15125	3-beta-hydroxysteroid-Delta(8),Delta(7)-isomerase OS=Homo sapiens OX=9606 GN=EBP PE=1 SV=3	0,273078085	0,972469
O75695	Protein XRP2 OS=Homo sapiens OX=9606 GN=RP2 PE=1 SV=4	0,157449531	0,972367
Q96TA1	Niban-like protein 1 OS=Homo sapiens OX=9606 GN=FAM129B PE=1 SV=3	-0,064200434	0,972366
P39880	Homeobox protein cut-like 1 OS=Homo sapiens OX=9606 GN=CUX1 PE=1 SV=3	-0,407693928	0,972278
Q9UBW8	COP9 signalosome complex subunit 7a OS=Homo sapiens OX=9606 GN=COPS7A PE=1 SV=1	-0,221566028	0,971245
Q9P287	BRCA2 and CDKN1A-interacting protein OS=Homo sapiens OX=9606 GN=BCCIP PE=1 SV=1	-0,221470705	0,97077
O95861	3'(2'),5'-bisphosphate nucleotidase 1 OS=Homo sapiens OX=9606 GN=BPNT1 PE=1 SV=1	0,128759187	0,969741
Q0ZGT2	Nexilin OS=Homo sapiens OX=9606 GN=NEXN PE=1 SV=1	0,406451987	0,969533
Q9H4L4	Sentrin-specific protease 3 OS=Homo sapiens OX=9606 GN=SENP3 PE=1 SV=2	-0,22078545	0,967358
Q969S3	Zinc finger protein 622 OS=Homo sapiens OX=9606 GN=ZNF622 PE=1 SV=1	0,405411074	0,967231
O95456	Proteasome assembly chaperone 1 OS=Homo sapiens OX=9606 GN=PSMG1 PE=1 SV=1	-0,145246461	0,966514
P40925	Malate dehydrogenase, cytoplasmic OS=Homo sapiens OX=9606 GN=MDH1 PE=1 SV=4	-0,103318796	0,966387
Q9H8Y8	Golgi reassembly-stacking protein 2 OS=Homo sapiens OX=9606 GN=GORASP2 PE=1 SV=3	0,135805971	0,964517
P25786	Proteasome subunit alpha type-1 OS=Homo sapiens OX=9606 GN=PSMA1 PE=1 SV=1	-0,09105754	0,96361
Q16799	Reticulon-1 OS=Homo sapiens OX=9606 GN=RTN1 PE=1 SV=1	-0,403615718	0,963254
Q12905	Interleukin enhancer-binding factor 2 OS=Homo sapiens OX=9606 GN=ILF2 PE=1 SV=2	0,096362325	0,96185
O60493	Sorting nexin-3 OS=Homo sapiens OX=9606 GN=SNX3 PE=1 SV=3	-0,135441728	0,961422
O15258	Protein RER1 OS=Homo sapiens OX=9606 GN=RER1 PE=1 SV=1	0,206075577	0,95934
Q9BW71	HIRA-interacting protein 3 OS=Homo sapiens OX=9606 GN=HIRIP3 PE=1 SV=3	0,26901576	0,956786
Q92905	COP9 signalosome complex subunit 5 OS=Homo sapiens OX=9606 GN=COPS5 PE=1 SV=4	0,120873721	0,956455
Q9H173	Nucleotide exchange factor SIL1 OS=Homo sapiens OX=9606 GN=SIL1 PE=1 SV=1	0,189486158	0,956411
P42575	Caspase-2 OS=Homo sapiens OX=9606 GN=CASP2 PE=1 SV=2	-0,169488253	0,954432
Q96J02	E3 ubiquitin-protein ligase Itchy homolog OS=Homo sapiens OX=9606 GN=ITCH PE=1 SV=2	-0,218101063	0,954009
Q5QJE6	Deoxynucleotidyltransferase terminal-interacting protein 2 OS=Homo sapiens OX=9606 GN=DNTTIP2 PE=1 SV=2	-0,218096861	0,953988
P51570	Galactokinase OS=Homo sapiens OX=9606 GN=GALK1 PE=1 SV=1	-0,092828401	0,953187
P53384	Cytosolic Fe-S cluster assembly factor NUBP1 OS=Homo sapiens OX=9606 GN=NUBP1 PE=1 SV=2	0,143346412	0,951471
Q9P2G1	Ankyrin repeat and IBR domain-containing protein 1 OS=Homo sapiens OX=9606 GN=ANKIB1 PE=1 SV=3	0,397683665	0,950073
Q9UNE7	E3 ubiquitin-protein ligase CHIP OS=Homo sapiens OX=9606 GN=STUB1 PE=1 SV=2	0,126577291	0,950052
P34896	Serine hydroxymethyltransferase, cytosolic OS=Homo sapiens OX=9606 GN=SHMT1 PE=1 SV=1	-0,217295905	0,950009
P05141	ADP/ATP translocase 2 OS=Homo sapiens OX=9606 GN=SLC25A5 PE=1 SV=7	-0,143004412	0,948769
Q9UMX5	Neudesin OS=Homo sapiens OX=9606 GN=NENF PE=1 SV=1	-0,188134757	0,948501
Q12888	TP53-binding protein 1 OS=Homo sapiens OX=9606 GN=TP53BP1 PE=1 SV=2	-0,089865513	0,948307
Q9UFN0	Protein NipSnap homolog 3A OS=Homo sapiens OX=9606 GN=NIPSNAP3A PE=1 SV=2	-0,142745494	0,946725
P62495	Eukaryotic peptide chain release factor subunit 1 OS=Homo sapiens OX=9606 GN=ETF1 PE=1 SV=3	-0,083138301	0,946397
Q99943	1-acyl-sn-glycerol-3-phosphate acyltransferase alpha OS=Homo sapiens OX=9606 GN=AGPAT1 PE=1 SV=2	0,395823615	0,945926
P49189	4-trimethylaminobutyraldehyde dehydrogenase OS=Homo sapiens OX=9606 GN=ALDH9A1 PE=1 SV=3	-0,105278464	0,945915

P49790	Nuclear pore complex protein Nup153 OS=Homo sapiens OX=9606 GN=NUP153 PE=1 SV=2	-0,098110501	0,945852
Q9BUK6	Protein misato homolog 1 OS=Homo sapiens OX=9606 GN=MSTO1 PE=1 SV=1	-0,119756347	0,945836
Q9NR45	Sialic acid synthase OS=Homo sapiens OX=9606 GN=NANS PE=1 SV=2	-0,105248802	0,945593
P63151	Serine/threonine-protein phosphatase 2A 55 kDa regulatory subunit B alpha isoform OS=Homo sapiens OX=9606 GN=PPF	0,098001552	0,944581
Q9NQX4	Unconventional myosin-Vc OS=Homo sapiens OX=9606 GN=MYO5C PE=1 SV=2	0,179251694	0,944393
A9UHW6	MIF4G domain-containing protein OS=Homo sapiens OX=9606 GN=MIF4GD PE=1 SV=1	-0,39507622	0,944259
O75964	ATP synthase subunit g, mitochondrial OS=Homo sapiens OX=9606 GN=ATP5MG PE=1 SV=3	-0,265636909	0,943744
Q8IXS6	Paralemmin-2 OS=Homo sapiens OX=9606 GN=PALM2 PE=1 SV=3	-0,394696859	0,943412
Q1ED39	Lysine-rich nucleolar protein 1 OS=Homo sapiens OX=9606 GN=KNOP1 PE=1 SV=1	-0,265492372	0,943186
Q08431	Lactadherin OS=Homo sapiens OX=9606 GN=MFGE8 PE=1 SV=3	0,265324789	0,942539
Q13393	Phospholipase D1 OS=Homo sapiens OX=9606 GN=PLD1 PE=1 SV=1	-0,565167681	0,942251
O95219	Sorting nexin-4 OS=Homo sapiens OX=9606 GN=SNX4 PE=1 SV=1	-0,12568302	0,942012
Q9Y4W2	Ribosomal biogenesis protein LAS1L OS=Homo sapiens OX=9606 GN=LAS1L PE=1 SV=2	-0,125663385	0,941836
P54753	Ephrin type-B receptor 3 OS=Homo sapiens OX=9606 GN=EPHB3 PE=1 SV=2	0,609669337	0,940351
Q9Y5J7	Mitochondrial import inner membrane translocase subunit Tim9 OS=Homo sapiens OX=9606 GN=TIMM9 PE=1 SV=1	0,393163915	0,939986
O75147	Obscurin-like protein 1 OS=Homo sapiens OX=9606 GN=OBSL1 PE=1 SV=4	-0,141881819	0,939914
P49588	Alanine--tRNA ligase, cytoplasmic OS=Homo sapiens OX=9606 GN=AARS PE=1 SV=2	0,060044099	0,939398
P78371	T-complex protein 1 subunit beta OS=Homo sapiens OX=9606 GN=CCT2 PE=1 SV=4	-0,068111367	0,938903
P35754	Glutaredoxin-1 OS=Homo sapiens OX=9606 GN=GLRX PE=1 SV=2	0,16713434	0,938887
Q8IWV7	E3 ubiquitin-protein ligase UBR1 OS=Homo sapiens OX=9606 GN=UBR1 PE=1 SV=1	0,113533713	0,938393
P42695	Condensin-2 complex subunit D3 OS=Homo sapiens OX=9606 GN=NCAPD3 PE=1 SV=2	-0,152725467	0,937914
P54760	Ephrin type-B receptor 4 OS=Homo sapiens OX=9606 GN=EPHB4 PE=1 SV=2	0,214630002	0,936781
Q9H3U1	Protein unc-45 homolog A OS=Homo sapiens OX=9606 GN=UNC45A PE=1 SV=1	0,070205459	0,935732
P04350	Tubulin beta-4A chain OS=Homo sapiens OX=9606 GN=TUBB4A PE=1 SV=2	0,113181758	0,934895
P17252	Protein kinase C alpha type OS=Homo sapiens OX=9606 GN=PRKCA PE=1 SV=4	-0,104235678	0,934618
Q9UP83	Conserved oligomeric Golgi complex subunit 5 OS=Homo sapiens OX=9606 GN=COG5 PE=1 SV=3	0,263253652	0,934547
Q9P000	COMM domain-containing protein 9 OS=Homo sapiens OX=9606 GN=COMMD9 PE=1 SV=2	0,124806558	0,93415
P62834	Ras-related protein Rap-1A OS=Homo sapiens OX=9606 GN=RAP1A PE=1 SV=1	-0,390227601	0,933413
P61916	NPC intracellular cholesterol transporter 2 OS=Homo sapiens OX=9606 GN=NPC2 PE=1 SV=1	0,165990321	0,931351
Q96ME1	F-box/LRR-repeat protein 18 OS=Homo sapiens OX=9606 GN=FBXL18 PE=1 SV=2	-0,10389704	0,930958
P41567	Eukaryotic translation initiation factor 1 OS=Homo sapiens OX=9606 GN=EIF1 PE=1 SV=1	-0,140735712	0,930894
Q96HE9	Proline-rich protein 11 OS=Homo sapiens OX=9606 GN=PRR11 PE=1 SV=1	-0,350360198	0,930004
P07686	Beta-hexosaminidase subunit beta OS=Homo sapiens OX=9606 GN=HEXB PE=1 SV=3	-0,090938254	0,929717
Q9P016	Thymocyte nuclear protein 1 OS=Homo sapiens OX=9606 GN=THYN1 PE=1 SV=1	0,261774213	0,928838
Q9UET6	Putative tRNA (cytidine(32)/guanosine(34)-2'-O)-methyltransferase OS=Homo sapiens OX=9606 GN=FTSJ1 PE=1 SV=2	-0,123070233	0,928734
P35637	RNA-binding protein FUS OS=Homo sapiens OX=9606 GN=FUS PE=1 SV=1	-0,13157566	0,928727
P10768	S-formylglutathione hydrolase OS=Homo sapiens OX=9606 GN=ESD PE=1 SV=2	-0,112542239	0,928548
Q9C0C9	(E3-independent) E2 ubiquitin-conjugating enzyme OS=Homo sapiens OX=9606 GN=UBE2O PE=1 SV=3	0,107771706	0,927899
Q8IXQ6	Protein mono-ADP-ribosyltransferase PARP9 OS=Homo sapiens OX=9606 GN=PARP9 PE=1 SV=2	0,212800432	0,927717
Q9UBT7	Alpha-catulin OS=Homo sapiens OX=9606 GN=CTNNAL1 PE=1 SV=2	0,387522041	0,927342
Q3B726	DNA-directed RNA polymerase I subunit RPA43 OS=Homo sapiens OX=9606 GN=TWISTNB PE=1 SV=1	-0,387497885	0,927288
Q9NQW7	Xaa-Pro aminopeptidase 1 OS=Homo sapiens OX=9606 GN=XPNPEP1 PE=1 SV=3	-0,107680756	0,926957
Q9H8V3	Protein ECT2 OS=Homo sapiens OX=9606 GN=ECT2 PE=1 SV=4	0,212591516	0,926683
Q14376	UDP-glucose 4-epimerase OS=Homo sapiens OX=9606 GN=GALE PE=1 SV=2	-0,151157546	0,926542
C9JLW8	Mapk-regulated corepressor-interacting protein 1 OS=Homo sapiens OX=9606 GN=MCRIP1 PE=1 SV=1	-0,261074455	0,926138
O75746	Calcium-binding mitochondrial carrier protein Aralar1 OS=Homo sapiens OX=9606 GN=SLC25A12 PE=1 SV=2	-0,103443731	0,926064
Q96K76	Ubiquitin carboxyl-terminal hydrolase 47 OS=Homo sapiens OX=9606 GN=USP47 PE=1 SV=3	0,08575334	0,925421
Q8N3V7	Synaptopodin OS=Homo sapiens OX=9606 GN=SYNPO PE=1 SV=2	0,150944469	0,924999
P52788	Spermine synthase OS=Homo sapiens OX=9606 GN=SMS PE=1 SV=2	-0,090545591	0,924862
Q9P2J5	Leucine--tRNA ligase, cytoplasmic OS=Homo sapiens OX=9606 GN=LARS PE=1 SV=2	-0,053056005	0,924803
Q9BTE1	Dynactin subunit 5 OS=Homo sapiens OX=9606 GN=DCTN5 PE=1 SV=1	-0,212205148	0,924771
Q13620	Cullin-4B OS=Homo sapiens OX=9606 GN=CUL4B PE=1 SV=4	0,077891055	0,923448
P80723	Brain acid soluble protein 1 OS=Homo sapiens OX=9606 GN=BASP1 PE=1 SV=2	-0,103168466	0,923095
Q15691	Microtubule-associated protein RP/EB family member 1 OS=Homo sapiens OX=9606 GN=MAPRE1 PE=1 SV=3	0,093025792	0,921669
Q16222	UDP-N-acetylhexosamine pyrophosphorylase OS=Homo sapiens OX=9606 GN=UAP1 PE=1 SV=3	-0,074621702	0,921624
Q8WWH5	Probable tRNA pseudouridine synthase 1 OS=Homo sapiens OX=9606 GN=TRUB1 PE=1 SV=1	-0,183475983	0,921323
O00461	Golgi integral membrane protein 4 OS=Homo sapiens OX=9606 GN=GOLIM4 PE=1 SV=1	-0,139488981	0,921106
Q9BQE4	Selenoprotein S OS=Homo sapiens OX=9606 GN=SELENOS PE=1 SV=3	0,384032409	0,919493
Q9Y5N6	Origin recognition complex subunit 6 OS=Homo sapiens OX=9606 GN=ORC6 PE=1 SV=1	0,383767812	0,918897
P40616	ADP-ribosylation factor-like protein 1 OS=Homo sapiens OX=9606 GN=ARL1 PE=1 SV=1	0,183048005	0,918833
Q9Y6M4	Casein kinase I isoform gamma-3 OS=Homo sapiens OX=9606 GN=CSNK1G3 PE=1 SV=2	-0,38363172	0,91859
Q8TF30	WASP homolog-associated protein with actin, membranes and microtubules OS=Homo sapiens OX=9606 GN=WHAMM PI	-0,150363818	0,918581
P83916	Chromobox protein homolog 1 OS=Homo sapiens OX=9606 GN=CBX1 PE=1 SV=1	0,182999446	0,918551
Q9H410	Kinetochore-associated protein DSN1 homolog OS=Homo sapiens OX=9606 GN=DSN1 PE=1 SV=2	0,382059379	0,918549
Q08945	FACT complex subunit SSRP1 OS=Homo sapiens OX=9606 GN=SSRP1 PE=1 SV=1	0,089951895	0,917536
Q7Z3E2	Coiled-coil domain-containing protein 186 OS=Homo sapiens OX=9606 GN=CCDC186 PE=1 SV=2	0,382984079	0,91713
Q96I15	Selenocysteine lyase OS=Homo sapiens OX=9606 GN=SCLY PE=1 SV=4	-0,382872953	0,91688
Q5FWF5	N-acetyltransferase ESCO1 OS=Homo sapiens OX=9606 GN=ESCO1 PE=1 SV=3	-0,951205597	0,916817
Q01085	Nucleolysin TIAR OS=Homo sapiens OX=9606 GN=TIAL1 PE=1 SV=1	-0,122828474	0,916468
Q9H6Z4	Ran-binding protein 3 OS=Homo sapiens OX=9606 GN=RANBP3 PE=1 SV=1	-0,09883191	0,9159
P27348	14-3-3 protein theta OS=Homo sapiens OX=9606 GN=YWHAQ PE=1 SV=1	-0,102441208	0,915265
Q9NRF2	SH2B adapter protein 1 OS=Homo sapiens OX=9606 GN=SH2B1 PE=1 SV=3	0,196733823	0,915048
A6NKF1	SAC3 domain-containing protein 1 OS=Homo sapiens OX=9606 GN=SAC3D1 PE=1 SV=2	-0,381720166	0,914279
P36404	ADP-ribosylation factor-like protein 2 OS=Homo sapiens OX=9606 GN=ARL2 PE=1 SV=4	-0,163320499	0,913813
Q92879	CUGBP Elav-like family member 1 OS=Homo sapiens OX=9606 GN=CELF1 PE=1 SV=2	-0,138402688	0,912598
Q00013	55 kDa erythrocyte membrane protein OS=Homo sapiens OX=9606 GN=MPP1 PE=1 SV=2	-0,149170113	0,912172
Q8IWA4	Mitofusin-1 OS=Homo sapiens OX=9606 GN=MFN1 PE=1 SV=3	-0,16305034	0,912043
Q9UK99	F-box only protein 3 OS=Homo sapiens OX=9606 GN=FBXO3 PE=1 SV=3	-0,380433116	0,911373
Q9P2K8	eIF-2-alpha kinase GCN2 OS=Homo sapiens OX=9606 GN=EIF2AK4 PE=1 SV=3	-0,276932082	0,911188
P49585	Choline-phosphate cytidylyltransferase A OS=Homo sapiens OX=9606 GN=PCYT1A PE=1 SV=2	-0,110752793	0,91085
P51991	Heterogeneous nuclear ribonucleoprotein A3 OS=Homo sapiens OX=9606 GN=HNRNPA3 PE=1 SV=2	-0,086905607	0,910615
O75530	Polycomb protein EED OS=Homo sapiens OX=9606 GN=EED PE=1 SV=2	-0,162773153	0,910226
Q6PJT7	Zinc finger CCCH domain-containing protein 14 OS=Homo sapiens OX=9606 GN=ZC3H14 PE=1 SV=1	0,148770723	0,909291
Q6NTF9	Rhomboid domain-containing protein 2 OS=Homo sapiens OX=9606 GN=RHBDD2 PE=2 SV=2	0,379285933	0,90878
Q8WUQ7	Cactin OS=Homo sapiens OX=9606 GN=CACTIN PE=1 SV=3	-0,379274766	0,908755
P42773	Cyclin-dependent kinase 4 inhibitor C OS=Homo sapiens OX=9606 GN=CDKN2C PE=1 SV=1	0,208936238	0,908611
P63279	SUMO-conjugating enzyme UBC9 OS=Homo sapiens OX=9606 GN=UBE2I PE=1 SV=1	0,129150903	0,908366
Q13428	Treacle protein OS=Homo sapiens OX=9606 GN=TCOF1 PE=1 SV=3	0,075201665	0,907811
Q9BZJ0	Crooked neck-like protein 1 OS=Homo sapiens OX=9606 GN=CRNKL1 PE=1 SV=4	-0,162169899	0,906277
P51452	Dual specificity protein phosphatase 3 OS=Homo sapiens OX=9606 GN=DUSP3 PE=1 SV=1	0,148298071	0,905883
P12694	2-oxoisovalerate dehydrogenase subunit alpha, mitochondrial OS=Homo sapiens OX=9606 GN=BCKDHA PE=1 SV=2	0,37792206	0,905695

P32298	#N/D	-1,208209776	0,905134
O75880	Protein SCO1 homolog, mitochondrial OS=Homo sapiens OX=9606 GN=SCO1 PE=1 SV=1	-0,377646053	0,90507
P53990	IST1 homolog OS=Homo sapiens OX=9606 GN=IST1 PE=1 SV=1	0,161871057	0,904321
Q9BWF3	RNA-binding protein 4 OS=Homo sapiens OX=9606 GN=RBM4 PE=1 SV=1	-0,109941773	0,902859
Q05707	Collagen alpha-1(XIV) chain OS=Homo sapiens OX=9606 GN=COL14A1 PE=1 SV=3	0,601029394	0,902795
Q9Y227	Ectonucleoside triphosphate diphosphohydrolase 4 OS=Homo sapiens OX=9606 GN=ENTPD4 PE=1 SV=1	-0,376549017	0,902585
O75815	Breast cancer anti-estrogen resistance protein 3 OS=Homo sapiens OX=9606 GN=BCAR3 PE=1 SV=1	-0,18022555	0,902445
Q13542	Eukaryotic translation initiation factor 4E-binding protein 2 OS=Homo sapiens OX=9606 GN=EIF4EBP2 PE=1 SV=1	-0,136830228	0,90244
P46108	Adapter molecule crk OS=Homo sapiens OX=9606 GN=CRK PE=1 SV=2	0,105294821	0,902327
O43504	Ragulator complex protein LAMTOR5 OS=Homo sapiens OX=9606 GN=LAMTOR5 PE=1 SV=1	-0,254854839	0,902151
Q92805	Golgin subfamily A member 1 OS=Homo sapiens OX=9606 GN=GOLGA1 PE=1 SV=3	-0,161414526	0,901336
Q9P2T1	GMP reductase 2 OS=Homo sapiens OX=9606 GN=GMPR2 PE=1 SV=1	0,254619307	0,901243
Q96S44	EKC/KEOPS complex subunit TP53RK OS=Homo sapiens OX=9606 GN=TP53RK PE=1 SV=2	0,375451256	0,900096
P27694	Replication protein A 70 kDa DNA-binding subunit OS=Homo sapiens OX=9606 GN=RPA1 PE=1 SV=2	-0,077939889	0,899878
P48739	Phosphatidylinositol transfer protein beta isoform OS=Homo sapiens OX=9606 GN=PITPNB PE=1 SV=2	0,114642161	0,897632
P52948	Nuclear pore complex protein Nup98-Nup96 OS=Homo sapiens OX=9606 GN=NUP98 PE=1 SV=4	-0,074413707	0,896311
Q96LD4	E3 ubiquitin-protein ligase TRIM47 OS=Homo sapiens OX=9606 GN=TRIM47 PE=1 SV=2	-0,109242182	0,89598
P63313	Thymosin beta-10 OS=Homo sapiens OX=9606 GN=TMSB10 PE=1 SV=2	0,253243184	0,895938
P52435	DNA-directed RNA polymerase II subunit RPB11-a OS=Homo sapiens OX=9606 GN=POLR2J PE=1 SV=1	0,206300106	0,895607
Q9BVL2	Nucleoporin p58/p45 OS=Homo sapiens OX=9606 GN=NUP58 PE=1 SV=1	-0,179034331	0,895545
Q9BRT6	Protein LLP homolog OS=Homo sapiens OX=9606 GN=LLPH PE=1 SV=1	-0,325771511	0,895485
Q13586	Stromal interaction molecule 1 OS=Homo sapiens OX=9606 GN=STIM1 PE=1 SV=3	-0,17899819	0,895336
O43374	Ras GTPase-activating protein 4 OS=Homo sapiens OX=9606 GN=RASA4 PE=2 SV=2	0,503614385	0,895265
Q15811	Intersectin-1 OS=Homo sapiens OX=9606 GN=ITSN1 PE=1 SV=3	0,120311311	0,894093
Q7Z4G1	COMM domain-containing protein 6 OS=Homo sapiens OX=9606 GN=COMMD6 PE=1 SV=1	0,252686686	0,893793
P50502	Hsc70-interacting protein OS=Homo sapiens OX=9606 GN=ST13 PE=1 SV=2	0,120090288	0,892135
Q15181	Inorganic pyrophosphatase OS=Homo sapiens OX=9606 GN=PPA1 PE=1 SV=2	0,093212937	0,889187
Q9NW13	RNA-binding protein 28 OS=Homo sapiens OX=9606 GN=RBM28 PE=1 SV=3	0,126786479	0,88862
Q9UL46	Proteasome activator complex subunit 2 OS=Homo sapiens OX=9606 GN=PSME2 PE=1 SV=4	0,096351346	0,888314
Q5JTH9	RRP12-like protein OS=Homo sapiens OX=9606 GN=RRP12 PE=1 SV=2	-0,073798367	0,88736
Q13049	E3 ubiquitin-protein ligase TRIM32 OS=Homo sapiens OX=9606 GN=TRIM32 PE=1 SV=2	-0,204525519	0,886866
O76003	Glutaredoxin-3 OS=Homo sapiens OX=9606 GN=GLRX3 PE=1 SV=2	0,108252899	0,886278
Q9HAV7	GrpE protein homolog 1, mitochondrial OS=Homo sapiens OX=9606 GN=GRPEL1 PE=1 SV=2	-0,126411584	0,885499
Q9BY42	Replication termination factor 2 OS=Homo sapiens OX=9606 GN=RTF2 PE=1 SV=3	0,177053682	0,884092
Q9Y243	RAC-gamma serine/threonine-protein kinase OS=Homo sapiens OX=9606 GN=AKT3 PE=1 SV=1	-0,176983895	0,883689
O00462	Beta-mannosidase OS=Homo sapiens OX=9606 GN=MANBA PE=1 SV=3	-0,248313723	0,883037
Q9NW64	Pre-mRNA-splicing factor RBM22 OS=Homo sapiens OX=9606 GN=RBM22 PE=1 SV=1	-0,203597448	0,8823
Q16739	Ceramide glucosyltransferase OS=Homo sapiens OX=9606 GN=UGCG PE=1 SV=1	0,367426596	0,881838
Q8N568	Serine/threonine-protein kinase DCLK2 OS=Homo sapiens OX=9606 GN=DCLK2 PE=1 SV=4	-0,0803308	0,881757
Q9GZT8	NIF3-like protein 1 OS=Homo sapiens OX=9606 GN=NIF3L1 PE=1 SV=2	0,125883516	0,881108
Q9H3H3	UPF0696 protein C11orf68 OS=Homo sapiens OX=9606 GN=C11orf68 PE=1 SV=3	0,134273103	0,880428
P10321	HLA class I histocompatibility antigen, Cw-7 alpha chain OS=Homo sapiens OX=9606 GN=HLA-C PE=1 SV=3	0,249124391	0,880067
Q709C8	Vacuolar protein sorting-associated protein 13C OS=Homo sapiens OX=9606 GN=VPS13C PE=1 SV=1	0,082233461	0,879744
O60725	Protein-S-isoprenylcysteine O-methyltransferase OS=Homo sapiens OX=9606 GN=ICMT PE=1 SV=1	0,366310695	0,87929
Q9UJX2	Cell division cycle protein 23 homolog OS=Homo sapiens OX=9606 GN=CDC23 PE=1 SV=3	0,112672578	0,879244
Q9NV88	Integrator complex subunit 9 OS=Homo sapiens OX=9606 GN=INTS9 PE=1 SV=2	0,202939782	0,879066
Q92556	Engulfment and cell motility protein 1 OS=Homo sapiens OX=9606 GN=ELMO1 PE=1 SV=2	0,366080708	0,878764
P09914	Interferon-induced protein with tetratricopeptide repeats 1 OS=Homo sapiens OX=9606 GN=IFIT1 PE=1 SV=2	-0,08994917	0,878722
Q9BT73	Proteasome assembly chaperone 3 OS=Homo sapiens OX=9606 GN=PSMG3 PE=1 SV=1	-0,176081239	0,878479
P17900	Ganglioside GM2 activator OS=Homo sapiens OX=9606 GN=GM2A PE=1 SV=4	0,357962329	0,878323
Q9BW61	DET1- and DDB1-associated protein 1 OS=Homo sapiens OX=9606 GN=DDA1 PE=1 SV=1	-0,365782986	0,878084
Q8N3F8	MICAL-like protein 1 OS=Homo sapiens OX=9606 GN=MICALL1 PE=1 SV=2	0,248457438	0,877498
Q00325	Phosphate carrier protein, mitochondrial OS=Homo sapiens OX=9606 GN=SLC25A3 PE=1 SV=2	0,107325546	0,877208
Q9P0T7	Transmembrane protein 9 OS=Homo sapiens OX=9606 GN=TMEM9 PE=1 SV=1	-0,365283352	0,876942
Q9UHG3	Prenylcysteine oxidase 1 OS=Homo sapiens OX=9606 GN=PCYOX1 PE=1 SV=3	0,102811107	0,876878
O00429	Dynamin-1-like protein OS=Homo sapiens OX=9606 GN=DNM1L PE=1 SV=2	0,062384126	0,876323
P05161	Ubiquitin-like protein ISG15 OS=Homo sapiens OX=9606 GN=ISG15 PE=1 SV=5	0,247697332	0,87457
Q5T1B0	Axonemal dynein light chain domain-containing protein 1 OS=Homo sapiens OX=9606 GN=AXDND1 PE=2 SV=1	-0,140260069	0,87455
P26440	Isovaleryl-CoA dehydrogenase, mitochondrial OS=Homo sapiens OX=9606 GN=IVD PE=1 SV=2	-0,364034344	0,874085
P22670	MHC class II regulatory factor RFX1 OS=Homo sapiens OX=9606 GN=RFX1 PE=1 SV=2	0,20189899	0,873952
P13612	Integrin alpha-4 OS=Homo sapiens OX=9606 GN=ITGA4 PE=1 SV=3	-1,055244813	0,873646
Q8IYB1	Protein MB21D2 OS=Homo sapiens OX=9606 GN=MB21D2 PE=1 SV=3	-0,106807349	0,872151
Q6PIU2	Neutral cholesterol ester hydrolase 1 OS=Homo sapiens OX=9606 GN=NCEH1 PE=1 SV=3	-0,117795166	0,871868
P41208	Centrin-2 OS=Homo sapiens OX=9606 GN=CETN2 PE=1 SV=1	-0,156851123	0,871608
O43427	Acidic fibroblast growth factor intracellular-binding protein OS=Homo sapiens OX=9606 GN=FIBP PE=1 SV=3	0,362930484	0,871558
P19404	NADH dehydrogenase [ubiquinone] flavoprotein 2, mitochondrial OS=Homo sapiens OX=9606 GN=NDUFV2 PE=1 SV=2	-0,156806001	0,871315
P20645	Cation-dependent mannose-6-phosphate receptor OS=Homo sapiens OX=9606 GN=M6PR PE=1 SV=1	0,201208393	0,87056
P50548	ETS domain-containing transcription factor ERF OS=Homo sapiens OX=9606 GN=ERF PE=1 SV=2	0,323600547	0,870216
O75410	Transforming acidic coiled-coil-containing protein 1 OS=Homo sapiens OX=9606 GN=TACC1 PE=1 SV=2	-0,324509076	0,869926
Q92608	Dedicator of cytokinesis protein 2 OS=Homo sapiens OX=9606 GN=DOCK2 PE=1 SV=2	-0,156570729	0,869788
Q8TCT8	Signal peptide peptidase-like 2A OS=Homo sapiens OX=9606 GN=SPPL2A PE=1 SV=2	-0,36209557	0,869645
Q7L4I2	Arginine/serine-rich coiled-coil protein 2 OS=Homo sapiens OX=9606 GN=RSRC2 PE=1 SV=1	0,362063236	0,869571
O75794	Cell division cycle protein 123 homolog OS=Homo sapiens OX=9606 GN=CDC123 PE=1 SV=1	-0,132860631	0,869488
P53992	Protein transport protein Sec24C OS=Homo sapiens OX=9606 GN=SEC24C PE=1 SV=3	-0,075669496	0,868345
P0C7P4	#N/D	-0,13270444	0,86828
Q6ZT07	TBC1 domain family member 9 OS=Homo sapiens OX=9606 GN=TBC1D9 PE=2 SV=2	-0,360590486	0,866193
Q8TF66	Leucine-rich repeat-containing protein 15 OS=Homo sapiens OX=9606 GN=LRRC15 PE=2 SV=2	0,091059034	0,864576
P48735	Isocitrate dehydrogenase [NADP], mitochondrial OS=Homo sapiens OX=9606 GN=IDH2 PE=1 SV=2	-0,085590321	0,864219
Q96HP0	#N/D	0,359426385	0,863521
Q7Z6K3	Protein prenyltransferase alpha subunit repeat-containing protein 1 OS=Homo sapiens OX=9606 GN=PTAR1 PE=1 SV=2	-0,244817648	0,863482
Q8WXG9	Adhesion G-protein coupled receptor V1 OS=Homo sapiens OX=9606 GN=ADGRV1 PE=1 SV=2	0,547658917	0,863468
Q9ULJ6	Zinc finger MIZ domain-containing protein 1 OS=Homo sapiens OX=9606 GN=ZMIZ1 PE=1 SV=3	0,921311666	0,863032
Q6IA86	Elongator complex protein 2 OS=Homo sapiens OX=9606 GN=ELP2 PE=1 SV=2	-0,155179025	0,860768
P61081	NEDD8-conjugating enzyme Ubc12 OS=Homo sapiens OX=9606 GN=UBE2M PE=1 SV=1	0,123394365	0,860481
Q9BQ24	Zinc finger FYVE domain-containing protein 21 OS=Homo sapiens OX=9606 GN=ZFYVE21 PE=1 SV=1	-0,223509649	0,859979
Q15424	Scaffold attachment factor B1 OS=Homo sapiens OX=9606 GN=SAFB PE=1 SV=4	0,0767511	0,859225
Q6KC79	Nipped-B-like protein OS=Homo sapiens OX=9606 GN=NIPBL PE=1 SV=2	-0,19869351	0,858225
P34932	Heat shock 70 kDa protein 4 OS=Homo sapiens OX=9606 GN=HSPA4 PE=1 SV=4	-0,049843486	0,857294
Q96GA7	Serine dehydratase-like OS=Homo sapiens OX=9606 GN=SDSL PE=1 SV=1	0,122942229	0,856747
P16150	Leukosialin OS=Homo sapiens OX=9606 GN=SPN PE=1 SV=1	0,356398488	0,85656
P63098	Calcineurin subunit B type 1 OS=Homo sapiens OX=9606 GN=PPP3R1 PE=1 SV=2	0,155074807	0,855621

Q8TBA6	Golgin subfamily A member 5 OS=Homo sapiens OX=9606 GN=GOLGA5 PE=1 SV=3	0,242681829	0,855262
P07942	Laminin subunit beta-1 OS=Homo sapiens OX=9606 GN=LAMB1 PE=1 SV=2	0,064046302	0,854469
P51798	H(+)/Cl(-) exchange transporter 7 OS=Homo sapiens OX=9606 GN=CLCN7 PE=1 SV=2	0,354538856	0,852276
P26378	ELAV-like protein 4 OS=Homo sapiens OX=9606 GN=ELAVL4 PE=1 SV=2	0,241618437	0,851171
Q8IWY9	Codanin-1 OS=Homo sapiens OX=9606 GN=CDAN1 PE=1 SV=4	-0,353902181	0,850809
Q01995	Transgelin OS=Homo sapiens OX=9606 GN=TAGLN PE=1 SV=4	0,089829292	0,85061
Q99567	Nuclear pore complex protein Nup88 OS=Homo sapiens OX=9606 GN=NUP88 PE=1 SV=2	0,1219099	0,848236
Q9Y6R0	Numb-like protein OS=Homo sapiens OX=9606 GN=NUMBL PE=1 SV=1	-0,196539063	0,847678
P04920	Anion exchange protein 2 OS=Homo sapiens OX=9606 GN=SLC4A2 PE=1 SV=4	-0,352349792	0,847227
Q8WUD4	Coiled-coil domain-containing protein 12 OS=Homo sapiens OX=9606 GN=CCDC12 PE=1 SV=1	0,240559526	0,847098
P46109	Crk-like protein OS=Homo sapiens OX=9606 GN=CRKL PE=1 SV=1	-0,121751723	0,846934
P62266	40S ribosomal protein S23 OS=Homo sapiens OX=9606 GN=RPS23 PE=1 SV=3	-0,170593261	0,846925
P08238	Heat shock protein HSP 90-beta OS=Homo sapiens OX=9606 GN=HSP90AB1 PE=1 SV=4	0,060604448	0,846254
P46063	ATP-dependent DNA helicase Q1 OS=Homo sapiens OX=9606 GN=RECQL PE=1 SV=3	0,068142018	0,845438
Q9UIV1	CCR4-NOT transcription complex subunit 7 OS=Homo sapiens OX=9606 GN=CNOT7 PE=1 SV=3	-0,24008416	0,84527
P30536	Translocator protein OS=Homo sapiens OX=9606 GN=TSPO PE=1 SV=3	-0,195947961	0,844787
Q56VL3	OCIA domain-containing protein 2 OS=Homo sapiens OX=9606 GN=OCIAD2 PE=1 SV=1	-0,114606051	0,843904
Q69YQ0	Cytospin-A OS=Homo sapiens OX=9606 GN=SPECC1L PE=1 SV=2	0,239418771	0,842711
Q92688	Acidic leucine-rich nuclear phosphoprotein 32 family member B OS=Homo sapiens OX=9606 GN=ANP32B PE=1 SV=1	-0,152326013	0,842336
Q9Y4R8	Telomere length regulation protein TEL2 homolog OS=Homo sapiens OX=9606 GN=TELO2 PE=1 SV=2	-0,103703381	0,842018
Q9H5X1	Cytosolic iron-sulfur assembly component 2A OS=Homo sapiens OX=9606 GN=CIAO2A PE=1 SV=1	0,350016077	0,841834
P45973	Chromobox protein homolog 5 OS=Homo sapiens OX=9606 GN=CBX5 PE=1 SV=1	0,152239333	0,841778
Q12981	Vesicle transport protein SEC20 OS=Homo sapiens OX=9606 GN=BNIP1 PE=1 SV=3	0,312105074	0,840142
Q9HCG8	Pre-mRNA-splicing factor CWC22 homolog OS=Homo sapiens OX=9606 GN=CWC22 PE=1 SV=3	0,194765736	0,839009
Q9NUQ9	Protein FAM49B OS=Homo sapiens OX=9606 GN=FAM49B PE=1 SV=1	0,103382747	0,838921
P49815	Tuberin OS=Homo sapiens OX=9606 GN=TSC2 PE=1 SV=2	-0,138855118	0,838421
Q96S66	Chloride channel CLIC-like protein 1 OS=Homo sapiens OX=9606 GN=CLCC1 PE=1 SV=1	0,15149762	0,837
Q9UK58	Cyclin-L1 OS=Homo sapiens OX=9606 GN=CCNL1 PE=1 SV=1	0,3477193	0,836518
P29353	SHC-transforming protein 1 OS=Homo sapiens OX=9606 GN=SHC1 PE=1 SV=4	0,108023152	0,836232
P09429	High mobility group protein B1 OS=Homo sapiens OX=9606 GN=HMGB1 PE=1 SV=3	-0,11370086	0,836009
P30876	DNA-directed RNA polymerase II subunit RPB2 OS=Homo sapiens OX=9606 GN=POLR2B PE=1 SV=1	-0,113680774	0,835834
Q03426	Mevalonate kinase OS=Homo sapiens OX=9606 GN=MVK PE=1 SV=1	-0,347072547	0,835019
Q8N8R5	UPF0565 protein C2orf69 OS=Homo sapiens OX=9606 GN=C2orf69 PE=1 SV=1	-0,347037109	0,834937
Q8N4H5	Mitochondrial import receptor subunit TOM5 homolog OS=Homo sapiens OX=9606 GN=TOMM5 PE=1 SV=1	-0,34693868	0,834709
Q9Y676	28S ribosomal protein S18b, mitochondrial OS=Homo sapiens OX=9606 GN=MRPS18B PE=1 SV=1	-0,346424569	0,833517
P55884	Eukaryotic translation initiation factor 3 subunit B OS=Homo sapiens OX=9606 GN=EIF3B PE=1 SV=3	-0,060701466	0,832479
Q99757	Thioredoxin, mitochondrial OS=Homo sapiens OX=9606 GN=TXN2 PE=1 SV=2	0,145703108	0,83245
P62070	Ras-related protein R-Ras2 OS=Homo sapiens OX=9606 GN=RRAS2 PE=1 SV=1	0,127990561	0,832017
Q9NSI2	Protein FAM207A OS=Homo sapiens OX=9606 GN=FAM207A PE=1 SV=2	-0,503179605	0,831068
Q8WVX9	Fatty acyl-CoA reductase 1 OS=Homo sapiens OX=9606 GN=FAR1 PE=1 SV=1	0,167748145	0,83065
Q00653	Nuclear factor NF-kappa-B p100 subunit OS=Homo sapiens OX=9606 GN=NFKB2 PE=1 SV=4	-0,344726183	0,829576
P05166	Propionyl-CoA carboxylase beta chain, mitochondrial OS=Homo sapiens OX=9606 GN=PCCB PE=1 SV=3	-0,23597476	0,829473
Q15404	Ras suppressor protein 1 OS=Homo sapiens OX=9606 GN=RSU1 PE=1 SV=3	0,094236234	0,828202
P29083	General transcription factor IIE subunit 1 OS=Homo sapiens OX=9606 GN=GTF2E1 PE=1 SV=2	-0,19243704	0,827646
Q15025	TNFAIP3-interacting protein 1 OS=Homo sapiens OX=9606 GN=TNIP1 PE=1 SV=2	-0,343885545	0,827624
Q9H3K6	BolA-like protein 2 OS=Homo sapiens OX=9606 GN=BOLA2 PE=1 SV=1	-0,150027138	0,827546
Q15650	Activating signal cointegrator 1 OS=Homo sapiens OX=9606 GN=TRIP4 PE=1 SV=4	0,343767518	0,82735
P08572	Collagen alpha-2(IV) chain OS=Homo sapiens OX=9606 GN=COL4A2 PE=1 SV=4	0,343732681	0,827269
Q6ZNC4	Zinc finger protein 704 OS=Homo sapiens OX=9606 GN=ZNF704 PE=1 SV=1	0,174605589	0,827083
Q9Y6W3	Calpain-7 OS=Homo sapiens OX=9606 GN=CAPN7 PE=1 SV=1	-0,192265705	0,82681
Q9NR31	GTP-binding protein SAR1a OS=Homo sapiens OX=9606 GN=SAR1A PE=1 SV=1	0,119298417	0,826801
Q96HY7	Probable 2-oxoglutarate dehydrogenase E1 component DHKTD1, mitochondrial OS=Homo sapiens OX=9606 GN=DHTKD1	-0,343400282	0,826497
Q9Y4E1	WASH complex subunit 2C OS=Homo sapiens OX=9606 GN=WASHC2C PE=1 SV=4	0,090688404	0,82621
O75175	CCR4-NOT transcription complex subunit 3 OS=Homo sapiens OX=9606 GN=CNOT3 PE=1 SV=1	-0,149768001	0,825882
O95983	Methyl-CpG-binding domain protein 3 OS=Homo sapiens OX=9606 GN=MBD3 PE=1 SV=1	0,137072895	0,825821
Q9UIJ7	GTP:AMP phosphotransferase AK3, mitochondrial OS=Homo sapiens OX=9606 GN=AK3 PE=1 SV=4	-0,137060128	0,825731
Q9GZQ3	COMM domain-containing protein 5 OS=Homo sapiens OX=9606 GN=COMMD5 PE=1 SV=1	0,234933612	0,825473
P31946	14-3-3 protein beta/alpha OS=Homo sapiens OX=9606 GN=YWHAB PE=1 SV=3	0,112432975	0,824981
Q9Y2H2	Phosphatidylinositide phosphatase SAC2 OS=Homo sapiens OX=9606 GN=INPP5F PE=1 SV=3	-0,14961113	0,824875
P18827	Syndecan-1 OS=Homo sapiens OX=9606 GN=SDC1 PE=1 SV=3	-0,234773837	0,824859
Q13496	Myotubularin OS=Homo sapiens OX=9606 GN=MTM1 PE=1 SV=2	-0,301226463	0,824821
Q96HA7	Tonsoku-like protein OS=Homo sapiens OX=9606 GN=TONSL PE=1 SV=2	0,29949346	0,824307
P62805	Histone H4 OS=Homo sapiens OX=9606 GN=HIST1H4A PE=1 SV=2	0,112293951	0,823774
Q9Y2A7	Nck-associated protein 1 OS=Homo sapiens OX=9606 GN=NCKAP1 PE=1 SV=1	-0,062120699	0,823684
Q8WUX9	Charged multivesicular body protein 7 OS=Homo sapiens OX=9606 GN=CHMP7 PE=1 SV=1	0,118882626	0,8234
Q8WV41	Sorting nexin-33 OS=Homo sapiens OX=9606 GN=SNX33 PE=1 SV=1	-0,191542307	0,823285
P33176	Kinesin-1 heavy chain OS=Homo sapiens OX=9606 GN=KIF5B PE=1 SV=1	-0,048141217	0,822142
P06396	Gelsolin OS=Homo sapiens OX=9606 GN=GSN PE=1 SV=1	0,07220263	0,820814
O96011	Peroxisomal membrane protein 11B OS=Homo sapiens OX=9606 GN=PEX11B PE=1 SV=1	-0,340532472	0,819825
P61201	COP9 signalosome complex subunit 2 OS=Homo sapiens OX=9606 GN=COPS2 PE=1 SV=1	0,101304006	0,818916
Q4V339	COBW domain-containing protein 6 OS=Homo sapiens OX=9606 GN=CBWD6 PE=3 SV=1	-0,339984691	0,818549
Q96EE3	Nucleoporin SEH1 OS=Homo sapiens OX=9606 GN=SEH1L PE=1 SV=3	0,09685239	0,816623
O75131	Copine-3 OS=Homo sapiens OX=9606 GN=CPNE3 PE=1 SV=1	-0,081627836	0,816558
P35241	Radixin OS=Homo sapiens OX=9606 GN=RDX PE=1 SV=1	-0,059683093	0,815749
Q96H20	Vacuolar-sorting protein SNF8 OS=Homo sapiens OX=9606 GN=SNF8 PE=1 SV=1	-0,23218136	0,814904
Q9NPA8	Transcription and mRNA export factor ENY2 OS=Homo sapiens OX=9606 GN=ENY2 PE=1 SV=1	-0,189804152	0,814824
P48723	Heat shock 70 kDa protein 13 OS=Homo sapiens OX=9606 GN=HSPA13 PE=1 SV=1	0,135508658	0,814797
Q9P0J1	[Pyruvate dehydrogenase [acetyl-transferring]]-phosphatase 1, mitochondrial OS=Homo sapiens OX=9606 GN=PDP1 PE=1	-0,338322906	0,814676
Q6IAA8	Ragulator complex protein LAMTOR1 OS=Homo sapiens OX=9606 GN=LAMTOR1 PE=1 SV=2	-0,135393381	0,813986
Q96CM8	Acyl-CoA synthetase family member 2, mitochondrial OS=Homo sapiens OX=9606 GN=ACSF2 PE=1 SV=2	0,135269736	0,813116
Q8IWJ2	GRIP and coiled-coil domain-containing protein 2 OS=Homo sapiens OX=9606 GN=GCC2 PE=1 SV=4	0,164470153	0,811971
Q8TEU7	Rap guanine nucleotide exchange factor 6 OS=Homo sapiens OX=9606 GN=RAPGEF6 PE=1 SV=2	0,466872854	0,811674
O15511	Actin-related protein 2/3 complex subunit 5 OS=Homo sapiens OX=9606 GN=ARPC5 PE=1 SV=3	0,147544662	0,811635
O75390	Citrate synthase, mitochondrial OS=Homo sapiens OX=9606 GN=CS PE=1 SV=2	-0,081199811	0,811454
Q8IX04	Ubiquitin-conjugating enzyme E2 variant 3 OS=Homo sapiens OX=9606 GN=UEVLD PE=1 SV=2	-0,125286639	0,811382
Q13111	Chromatin assembly factor 1 subunit A OS=Homo sapiens OX=9606 GN=CHAF1A PE=1 SV=3	-0,188945662	0,810649
Q14C86	GTPase-activating protein and VPS9 domain-containing protein 1 OS=Homo sapiens OX=9606 GN=GAPVD1 PE=1 SV=2	0,064568147	0,810483
Q86X10	Ral GTPase-activating protein subunit beta OS=Homo sapiens OX=9606 GN=RALGAPB PE=1 SV=1	0,336429362	0,810257
Q9NRY2	SOSS complex subunit C OS=Homo sapiens OX=9606 GN=INIP PE=1 SV=1	-0,336114213	0,80952
Q9ULV3	Cip1-interacting zinc finger protein OS=Homo sapiens OX=9606 GN=CIZ1 PE=1 SV=2	-0,230777373	0,809516
Q6DHV7	Adenosine deaminase-like protein OS=Homo sapiens OX=9606 GN=ADAL PE=2 SV=2	0,322463992	0,809379

Q8TF05	Serine/threonine-protein phosphatase 4 regulatory subunit 1 OS=Homo sapiens OX=9606 GN=PPP4R1 PE=1 SV=1	0,080996486	0,809032
Q9Y6E0	Serine/threonine-protein kinase 24 OS=Homo sapiens OX=9606 GN=STK24 PE=1 SV=1	-0,124925069	0,808632
Q96HQ2	CDKN2AIP N-terminal-like protein OS=Homo sapiens OX=9606 GN=CDKN2AIPNL PE=1 SV=1	-0,335564982	0,808237
Q7Z3C6	Autophagy-related protein 9A OS=Homo sapiens OX=9606 GN=ATG9A PE=1 SV=3	0,134486778	0,807614
Q9UJW0	Dynactin subunit 4 OS=Homo sapiens OX=9606 GN=DCTN4 PE=1 SV=1	-0,100034629	0,806761
Q15532	Protein SSXT OS=Homo sapiens OX=9606 GN=SS18 PE=1 SV=3	-0,334890672	0,806661
P00390	Glutathione reductase, mitochondrial OS=Homo sapiens OX=9606 GN=GSR PE=1 SV=2	0,083234908	0,806582
P38571	Lysosomal acid lipase/cholesteryl ester hydrolase OS=Homo sapiens OX=9606 GN=LIPA PE=1 SV=2	-0,187956288	0,805842
Q9NVE7	Pantothenate kinase 4 OS=Homo sapiens OX=9606 GN=PANK4 PE=1 SV=1	-0,334511042	0,805773
P55212	Caspase-6 OS=Homo sapiens OX=9606 GN=CASP6 PE=1 SV=2	-0,134164493	0,805351
Q9Y5S2	Serine/threonine-protein kinase MRCK beta OS=Homo sapiens OX=9606 GN=CDC42BPB PE=1 SV=2	0,116468873	0,803727
Q9Y3Y2	Chromatin target of PRMT1 protein OS=Homo sapiens OX=9606 GN=CHTOP PE=1 SV=2	0,187417913	0,803228
O76031	ATP-dependent Clp protease ATP-binding subunit clpX-like, mitochondrial OS=Homo sapiens OX=9606 GN=CLPX PE=1 SV=	0,099649326	0,803081
Q7LBC6	Lysine-specific demethylase 3B OS=Homo sapiens OX=9606 GN=KDM3B PE=1 SV=2	-0,091789234	0,802691
Q8NB37	Glutamine amidotransferase-like class 1 domain-containing protein 1 OS=Homo sapiens OX=9606 GN=GATD1 PE=1 SV=1	0,134441214	0,801402
Q9H4G0	Band 4.1-like protein 1 OS=Homo sapiens OX=9606 GN=EPB41L1 PE=1 SV=2	0,116136527	0,801027
P55210	Caspase-7 OS=Homo sapiens OX=9606 GN=CASP7 PE=1 SV=1	0,22856249	0,801019
Q9Y5K8	V-type proton ATPase subunit D OS=Homo sapiens OX=9606 GN=ATP6V1D PE=1 SV=1	-0,186942439	0,80092
Q9ULG6	Cell cycle progression protein 1 OS=Homo sapiens OX=9606 GN=CCPG1 PE=1 SV=3	0,34703378	0,800112
P30086	Phosphatidylethanolamine-binding protein 1 OS=Homo sapiens OX=9606 GN=PEBP1 PE=1 SV=3	0,104040716	0,799833
Q712K3	Ubiquitin-conjugating enzyme E2 R2 OS=Homo sapiens OX=9606 GN=UBE2R2 PE=1 SV=1	-0,162300065	0,799649
O00764	Pyridoxal kinase OS=Homo sapiens OX=9606 GN=PDXK PE=1 SV=1	-0,080204375	0,799617
P62310	U6 snRNA-associated Sm-like protein LSm3 OS=Homo sapiens OX=9606 GN=LSM3 PE=1 SV=2	-0,228136414	0,799385
O95070	Protein YIF1A OS=Homo sapiens OX=9606 GN=YIF1A PE=1 SV=2	-0,331743422	0,799295
Q9NVF7	F-box only protein 28 OS=Homo sapiens OX=9606 GN=FBXO28 PE=1 SV=1	-0,331701316	0,799196
Q9NYJ8	TGF-beta-activated kinase 1 and MAP3K7-binding protein 2 OS=Homo sapiens OX=9606 GN=TAB2 PE=1 SV=1	-0,339397895	0,799055
P52597	Heterogeneous nuclear ribonucleoprotein F OS=Homo sapiens OX=9606 GN=HNRNPF PE=1 SV=3	-0,088132514	0,798579
P17405	Sphingomyelin phosphodiesterase OS=Homo sapiens OX=9606 GN=SMPD1 PE=1 SV=5	-0,123566253	0,798317
Q15819	Ubiquitin-conjugating enzyme E2 variant 2 OS=Homo sapiens OX=9606 GN=UBE2V2 PE=1 SV=4	-0,33083594	0,797168
O95571	Persulfide dioxygenase ETHE1, mitochondrial OS=Homo sapiens OX=9606 GN=ETHE1 PE=1 SV=2	-0,161811601	0,79688
Q9ULP9	TBC1 domain family member 24 OS=Homo sapiens OX=9606 GN=TBC1D24 PE=1 SV=2	-0,052687978	0,796477
P51948	CDK-activating kinase assembly factor MAT1 OS=Homo sapiens OX=9606 GN=MNAT1 PE=1 SV=1	-0,330093391	0,795426
P17858	ATP-dependent 6-phosphofructokinase, liver type OS=Homo sapiens OX=9606 GN=PFKL PE=1 SV=6	-0,082260652	0,795361
P63208	S-phase kinase-associated protein 1 OS=Homo sapiens OX=9606 GN=SKP1 PE=1 SV=2	0,098837201	0,795338
Q96MU7	YTH domain-containing protein 1 OS=Homo sapiens OX=9606 GN=YTHDC1 PE=1 SV=3	0,329952336	0,795096
Q96J92	#N/D	0,186327628	0,794696
Q6N069	N-alpha-acetyltransferase 16, NatA auxiliary subunit OS=Homo sapiens OX=9606 GN=NAA16 PE=1 SV=2	0,249271815	0,79447
P02452	Collagen alpha-1(I) chain OS=Homo sapiens OX=9606 GN=COL1A1 PE=1 SV=5	0,226795215	0,794243
Q9UBL3	Set1/Ash2 histone methyltransferase complex subunit ASH2 OS=Homo sapiens OX=9606 GN=ASH2L PE=1 SV=1	-0,103377468	0,793811
Q5JS54	Proteasome assembly chaperone 4 OS=Homo sapiens OX=9606 GN=PSMG4 PE=1 SV=2	-0,226650828	0,793689
Q15750	TGF-beta-activated kinase 1 and MAP3K7-binding protein 1 OS=Homo sapiens OX=9606 GN=TAB1 PE=1 SV=1	-0,328437805	0,791541
Q07002	Cyclin-dependent kinase 18 OS=Homo sapiens OX=9606 GN=CDK18 PE=1 SV=4	0,225991081	0,791161
Q96L92	Sorting nexin-27 OS=Homo sapiens OX=9606 GN=SNX27 PE=1 SV=2	0,094298298	0,791142
P50281	Matrix metalloproteinase-14 OS=Homo sapiens OX=9606 GN=MMP14 PE=1 SV=3	-0,114715732	0,78951
Q9H3Z4	DnaJ homolog subfamily C member 5 OS=Homo sapiens OX=9606 GN=DNAJC5 PE=1 SV=1	-0,326872238	0,787862
P62995	Transformer-2 protein homolog beta OS=Homo sapiens OX=9606 GN=TRA2B PE=1 SV=1	-0,114505967	0,787813
Q7Z7H5	Transmembrane emp24 domain-containing protein 4 OS=Homo sapiens OX=9606 GN=TMED4 PE=1 SV=1	-0,15997283	0,786473
P78559	Microtubule-associated protein 1A OS=Homo sapiens OX=9606 GN=MAP1A PE=1 SV=6	0,040685666	0,78569
Q9Y281	Cofilin-2 OS=Homo sapiens OX=9606 GN=CFL2 PE=1 SV=1	-0,131315434	0,785411
P13796	Plastin-2 OS=Homo sapiens OX=9606 GN=LCP1 PE=1 SV=6	0,159744199	0,785181
P22307	Non-specific lipid-transfer protein OS=Homo sapiens OX=9606 GN=SCP2 PE=1 SV=2	-0,102298154	0,784035
Q9BQC3	2-(3-amino-3-carboxypropyl)histidine synthase subunit 2 OS=Homo sapiens OX=9606 GN=DPH2 PE=1 SV=1	-0,190854507	0,782617
O76094	Signal recognition particle subunit SRP72 OS=Homo sapiens OX=9606 GN=SRP72 PE=1 SV=3	0,067866513	0,782553
O00505	Importin subunit alpha-4 OS=Homo sapiens OX=9606 GN=KPNA3 PE=1 SV=2	0,102128743	0,782504
Q8WWY3	U4/U6 small nuclear ribonucleoprotein Prp31 OS=Homo sapiens OX=9606 GN=PRPF31 PE=1 SV=2	0,089828187	0,782398
Q6UXN9	WD repeat-containing protein 82 OS=Homo sapiens OX=9606 GN=WDR82 PE=1 SV=1	0,097456325	0,782216
O00400	Acetyl-coenzyme A transporter 1 OS=Homo sapiens OX=9606 GN=SLC33A1 PE=1 SV=1	-0,459804733	0,780131
Q9NT62	Ubiquitin-like-conjugating enzyme ATG3 OS=Homo sapiens OX=9606 GN=ATG3 PE=1 SV=1	-0,121085437	0,779563
P46939	Utrophin OS=Homo sapiens OX=9606 GN=UTRN PE=1 SV=2	-0,046003992	0,778612
Q9C0A0	#N/D	-0,153466512	0,778154
Q5T1M5	FK506-binding protein 15 OS=Homo sapiens OX=9606 GN=FKBP15 PE=1 SV=2	0,092887821	0,777161
Q99797	Mitochondrial intermediate peptidase OS=Homo sapiens OX=9606 GN=MIPEP PE=1 SV=2	-0,18197569	0,776872
Q0VDG4	Secernin-3 OS=Homo sapiens OX=9606 GN=SCRN3 PE=1 SV=1	-0,181804796	0,776046
P15311	Ezrin OS=Homo sapiens OX=9606 GN=EZR PE=1 SV=4	-0,05814289	0,775945
Q9NRL2	Bromodomain adjacent to zinc finger domain protein 1A OS=Homo sapiens OX=9606 GN=BAZ1A PE=1 SV=2	-0,17548709	0,775398
Q9Y2Q5	Ragulator complex protein LAMTOR2 OS=Homo sapiens OX=9606 GN=LAMTOR2 PE=1 SV=1	0,321576281	0,77539
Q9UNP9	Peptidyl-prolyl cis-trans isomerase E OS=Homo sapiens OX=9606 GN=PPIE PE=1 SV=1	-0,181646063	0,775279
Q01196	Runt-related transcription factor 1 OS=Homo sapiens OX=9606 GN=RUNX1 PE=1 SV=3	0,15787304	0,774619
Q86WQ0	Nuclear receptor 2C2-associated protein OS=Homo sapiens OX=9606 GN=NR2C2AP PE=1 SV=1	0,22159894	0,77434
P42126	Enoyl-CoA delta isomerase 1, mitochondrial OS=Homo sapiens OX=9606 GN=ECI1 PE=1 SV=1	-0,119779636	0,769733
Q9Y6E2	Basic leucine zipper and W2 domain-containing protein 2 OS=Homo sapiens OX=9606 GN=BZW2 PE=1 SV=1	-0,088537378	0,769114
P30044	Peroxiredoxin-5, mitochondrial OS=Homo sapiens OX=9606 GN=PRDX5 PE=1 SV=4	-0,105914115	0,768862
Q9H773	dCTP pyrophosphatase 1 OS=Homo sapiens OX=9606 GN=DCTPP1 PE=1 SV=1	0,100611571	0,76882
P30419	Glycylpeptide N-tetradecanoyltransferase 1 OS=Homo sapiens OX=9606 GN=NMT1 PE=1 SV=2	0,088468008	0,768402
Q15599	Na(+)/H(+) exchange regulatory cofactor NHE-RF2 OS=Homo sapiens OX=9606 GN=SLC9A3R2 PE=1 SV=2	0,095971763	0,768171
Q9H7N4	Splicing factor, arginine/serine-rich 19 OS=Homo sapiens OX=9606 GN=SCAF1 PE=1 SV=3	-0,180141328	0,768017
Q69YH5	Cell division cycle-associated protein 2 OS=Homo sapiens OX=9606 GN=CDCA2 PE=1 SV=2	0,21992518	0,767936
Q9Y3E5	Peptidyl-tRNA hydrolase 2, mitochondrial OS=Homo sapiens OX=9606 GN=PTRH2 PE=1 SV=1	0,180094217	0,76779
Q92530	Proteasome inhibitor PI31 subunit OS=Homo sapiens OX=9606 GN=PSMF1 PE=1 SV=2	-0,156608453	0,767496
Q8IVW4	Cyclin-dependent kinase-like 3 OS=Homo sapiens OX=9606 GN=CDKL3 PE=1 SV=1	-0,279691477	0,767466
Q14746	Conserved oligomeric Golgi complex subunit 2 OS=Homo sapiens OX=9606 GN=COG2 PE=1 SV=1	0,156483209	0,766792
Q9NY65	#N/D	-0,121771682	0,766626
Q9NX46	ADP-ribose glycohydrolase ARH3 OS=Homo sapiens OX=9606 GN=ADPRHL2 PE=1 SV=1	0,179825589	0,766494
P51572	B-cell receptor-associated protein 31 OS=Homo sapiens OX=9606 GN=BCAP31 PE=1 SV=3	0,091753711	0,765965
P62424	60S ribosomal protein L7a OS=Homo sapiens OX=9606 GN=RPL7A PE=1 SV=2	-0,085024673	0,765317
Q8NFA0	Ubiquitin carboxyl-terminal hydrolase 32 OS=Homo sapiens OX=9606 GN=USP32 PE=1 SV=1	-0,317142204	0,764914
Q8WWC4	m-AAA protease-interacting protein 1, mitochondrial OS=Homo sapiens OX=9606 GN=MAIP1 PE=1 SV=1	-0,21910106	0,764784
Q16539	Mitogen-activated protein kinase 14 OS=Homo sapiens OX=9606 GN=MAPK14 PE=1 SV=3	-0,11163151	0,764647
Q12907	Vesicular integral-membrane protein VIP36 OS=Homo sapiens OX=9606 GN=LMAN2 PE=1 SV=1	-0,105414171	0,764598
Q6P5R6	60S ribosomal protein L22-like 1 OS=Homo sapiens OX=9606 GN=RPL22L1 PE=1 SV=2	-0,31683005	0,764175
P48059	LIM and senescent cell antigen-like-containing domain protein 1 OS=Homo sapiens OX=9606 GN=LIMS1 PE=1 SV=4	0,139981287	0,763561

P36639	7,8-dihydro-8-oxoguanine triphosphatase OS=Homo sapiens OX=9606 GN=NUDT1 PE=1 SV=3	-0,139965961	0,763464
Q01082	Spectrin beta chain, non-erythrocytic 1 OS=Homo sapiens OX=9606 GN=SPTBN1 PE=1 SV=2	-0,028408216	0,762054
Q9HCH3	Copine-5 OS=Homo sapiens OX=9606 GN=CPNE5 PE=1 SV=2	-0,363979352	0,761685
Q92575	UBX domain-containing protein 4 OS=Homo sapiens OX=9606 GN=UBXN4 PE=1 SV=2	0,155515248	0,761349
Q92538	Golgi-specific brefeldin A-resistance guanine nucleotide exchange factor 1 OS=Homo sapiens OX=9606 GN=GBF1 PE=1 SV	-0,067781272	0,761286
P14373	Zinc finger protein RFP OS=Homo sapiens OX=9606 GN=TRIM27 PE=1 SV=1	0,357122993	0,761238
O95400	CD2 antigen cytoplasmic tail-binding protein 2 OS=Homo sapiens OX=9606 GN=CD2BP2 PE=1 SV=1	-0,118469493	0,7599
Q01780	Exosome component 10 OS=Homo sapiens OX=9606 GN=EXOSC10 PE=1 SV=2	-0,087503937	0,758522
P50443	Sulfate transporter OS=Homo sapiens OX=9606 GN=SLC26A2 PE=1 SV=2	0,314441879	0,758519
Q16762	Thiosulfate sulfurtransferase OS=Homo sapiens OX=9606 GN=TST PE=1 SV=4	-0,217299937	0,757899
P50583	Bis(5'-nucleosyl)-tetraphosphatase [asymmetrical] OS=Homo sapiens OX=9606 GN=NUDT2 PE=1 SV=3	-0,177995619	0,757678
P83876	Thioredoxin-like protein 4A OS=Homo sapiens OX=9606 GN=TXNL4A PE=1 SV=1	0,177835109	0,756906
Q9BRB3	Phosphatidylinositol N-acetylglucosaminyltransferase subunit Q OS=Homo sapiens OX=9606 GN=PIGQ PE=1 SV=3	0,313727743	0,756826
Q96GA3	Protein LTV1 homolog OS=Homo sapiens OX=9606 GN=LTV1 PE=1 SV=1	-0,154682576	0,756672
Q6P1M0	Long-chain fatty acid transport protein 4 OS=Homo sapiens OX=9606 GN=SLC27A4 PE=1 SV=1	0,216977052	0,756665
P07711	Cathepsin L1 OS=Homo sapiens OX=9606 GN=CTSL PE=1 SV=2	0,216649292	0,755412
Q96TC7	Regulator of microtubule dynamics protein 3 OS=Homo sapiens OX=9606 GN=RMDN3 PE=1 SV=2	0,216592447	0,755195
P61224	Ras-related protein Rap-1b OS=Homo sapiens OX=9606 GN=RAP1B PE=1 SV=1	0,126932318	0,754951
Q9UKI2	Cdc42 effector protein 3 OS=Homo sapiens OX=9606 GN=CDC42EP3 PE=1 SV=1	-0,154342418	0,754763
P62851	40S ribosomal protein S25 OS=Homo sapiens OX=9606 GN=RPS25 PE=1 SV=1	-0,312714793	0,754423
Q9UJF2	Ras GTPase-activating protein nGAP OS=Homo sapiens OX=9606 GN=RASAL2 PE=1 SV=2	0,154214256	0,754044
Q9H0E9	Bromodomain-containing protein 8 OS=Homo sapiens OX=9606 GN=BRD8 PE=1 SV=2	-0,312490576	0,753891
Q8NHU6	Tudor domain-containing protein 7 OS=Homo sapiens OX=9606 GN=TDRD7 PE=1 SV=2	0,110253205	0,753597
P61923	Coatomer subunit zeta-1 OS=Homo sapiens OX=9606 GN=COPZ1 PE=1 SV=1	0,126725275	0,753519
Q9UH99	SUN domain-containing protein 2 OS=Homo sapiens OX=9606 GN=SUN2 PE=1 SV=3	-0,098869259	0,75318
Q9P0K7	Ankycorbin OS=Homo sapiens OX=9606 GN=RAI14 PE=1 SV=2	-0,090295039	0,751626
O60331	Phosphatidylinositol 4-phosphate 5-kinase type-1 gamma OS=Homo sapiens OX=9606 GN=PIP5K1C PE=1 SV=2	0,215445097	0,750812
O43896	Kinesin-like protein KIF1C OS=Homo sapiens OX=9606 GN=KIF1C PE=1 SV=3	-0,215444955	0,750812
Q9Y3B7	39S ribosomal protein L11, mitochondrial OS=Homo sapiens OX=9606 GN=MRPL11 PE=1 SV=1	-0,215329183	0,750369
Q9NQT4	Exosome complex component RRP46 OS=Homo sapiens OX=9606 GN=EXOSC5 PE=1 SV=1	-0,215306873	0,750284
Q969X5	Endoplasmic reticulum-Golgi intermediate compartment protein 1 OS=Homo sapiens OX=9606 GN=ERGIC1 PE=1 SV=1	0,137855273	0,750157
O60518	Ran-binding protein 6 OS=Homo sapiens OX=9606 GN=RANBP6 PE=1 SV=2	-0,109630301	0,748616
Q9NVH1	DnaJ homolog subfamily C member 11 OS=Homo sapiens OX=9606 GN=DNAJC11 PE=1 SV=2	0,214702458	0,747976
Q5VYS8	Terminal uridylyltransferase 7 OS=Homo sapiens OX=9606 GN=TUT7 PE=1 SV=1	-0,309823172	0,747556
Q9BQL6	Fermitin family homolog 1 OS=Homo sapiens OX=9606 GN=FERMT1 PE=1 SV=1	0,159477025	0,747133
Q15785	Mitochondrial import receptor subunit TOM34 OS=Homo sapiens OX=9606 GN=TOMM34 PE=1 SV=2	0,083255921	0,746552
Q8IXQ5	Kelch-like protein 7 OS=Homo sapiens OX=9606 GN=KLHL7 PE=1 SV=2	0,309347759	0,746425
Q9H792	Inactive tyrosine-protein kinase PEAK1 OS=Homo sapiens OX=9606 GN=PEAK1 PE=1 SV=4	0,309126261	0,745898
P62873	Guanine nucleotide-binding protein G(I)/G(S)/G(T) subunit beta-1 OS=Homo sapiens OX=9606 GN=GNB1 PE=1 SV=3	0,11657288	0,745716
Q9UKL0	REST corepressor 1 OS=Homo sapiens OX=9606 GN=RCOR1 PE=1 SV=2	-0,214026252	0,745395
Q6IAN0	Dehydrogenase/reductase SDR family member 7B OS=Homo sapiens OX=9606 GN=DHRS7B PE=1 SV=2	0,213955948	0,745126
Q08378	Golgin subfamily A member 3 OS=Homo sapiens OX=9606 GN=GOLGA3 PE=1 SV=2	0,097946316	0,744928
Q0PNE2	Elongator complex protein 6 OS=Homo sapiens OX=9606 GN=ELP6 PE=1 SV=1	0,213450415	0,743197
P83731	60S ribosomal protein L24 OS=Homo sapiens OX=9606 GN=RPL24 PE=1 SV=1	-0,136720357	0,743022
O94888	UBX domain-containing protein 7 OS=Homo sapiens OX=9606 GN=UBXN7 PE=1 SV=2	-0,116192973	0,742883
P46926	Glucosamine-6-phosphate isomerase 1 OS=Homo sapiens OX=9606 GN=GNPDA1 PE=1 SV=1	-0,093219281	0,7423
Q9Y5X3	Sorting nexin-5 OS=Homo sapiens OX=9606 GN=SNX5 PE=1 SV=1	-0,080059999	0,741871
Q9UBQ6	Exostosin-like 2 OS=Homo sapiens OX=9606 GN=EXTL2 PE=1 SV=1	-0,360079211	0,741307
Q9UQ16	Dynamin-3 OS=Homo sapiens OX=9606 GN=DNM3 PE=1 SV=4	-0,499717673	0,741181
P30041	Peroxiredoxin-6 OS=Homo sapiens OX=9606 GN=PRDX6 PE=1 SV=3	0,064808048	0,740875
Q9Y312	Protein AAR2 homolog OS=Homo sapiens OX=9606 GN=AAR2 PE=1 SV=2	0,124856187	0,740617
P15291	Beta-1,4-galactosyltransferase 1 OS=Homo sapiens OX=9606 GN=B4GALT1 PE=1 SV=5	-0,212612105	0,739998
P49959	Double-strand break repair protein MRE11 OS=Homo sapiens OX=9606 GN=MRE11 PE=1 SV=3	0,115430411	0,737202
P62263	40S ribosomal protein S14 OS=Homo sapiens OX=9606 GN=RPS14 PE=1 SV=3	-0,124338092	0,737049
Q9Y5P4	Collagen type IV alpha-3-binding protein OS=Homo sapiens OX=9606 GN=COL4A3BP PE=1 SV=1	0,11531863	0,73637
O95071	E3 ubiquitin-protein ligase UBR5 OS=Homo sapiens OX=9606 GN=UBR5 PE=1 SV=2	-0,11525456	0,735894
Q9UBQ0	Vacuolar protein sorting-associated protein 29 OS=Homo sapiens OX=9606 GN=VPS29 PE=1 SV=1	-0,107985926	0,735504
O95831	Apoptosis-inducing factor 1, mitochondrial OS=Homo sapiens OX=9606 GN=AIFM1 PE=1 SV=1	0,107937043	0,735115
Q53GA4	Pleckstrin homology-like domain family A member 2 OS=Homo sapiens OX=9606 GN=PHLDA2 PE=1 SV=2	-0,304539057	0,734974
Q8TB52	F-box only protein 30 OS=Homo sapiens OX=9606 GN=FBXO30 PE=1 SV=3	0,15062138	0,733941
O94776	Metastasis-associated protein MTA2 OS=Homo sapiens OX=9606 GN=MTA2 PE=1 SV=1	-0,06286236	0,732607
P13804	Electron transfer flavoprotein subunit alpha, mitochondrial OS=Homo sapiens OX=9606 GN=ETFA PE=1 SV=1	0,084895454	0,731952
Q9Y3A6	Transmembrane emp24 domain-containing protein 5 OS=Homo sapiens OX=9606 GN=TMED5 PE=1 SV=1	0,840553122	0,731736
Q9Y6G5	COMM domain-containing protein 10 OS=Homo sapiens OX=9606 GN=COMMD10 PE=1 SV=1	-0,210226157	0,730899
P78318	Immunoglobulin-binding protein 1 OS=Homo sapiens OX=9606 GN=IGBP1 PE=1 SV=1	-0,123411152	0,730675
P52209	6-phosphogluconate dehydrogenase, decarboxylating OS=Homo sapiens OX=9606 GN=PGD PE=1 SV=3	-0,060233872	0,729836
P22102	Trifunctional purine biosynthetic protein adenosine-3 OS=Homo sapiens OX=9606 GN=GART PE=1 SV=1	-0,051268006	0,72965
P40426	Pre-B-cell leukemia transcription factor 3 OS=Homo sapiens OX=9606 GN=PBX3 PE=1 SV=1	-0,302245272	0,7295
Q9UID3	Vacuolar protein sorting-associated protein 51 homolog OS=Homo sapiens OX=9606 GN=VPS51 PE=1 SV=2	-0,209752662	0,729094
Q8N1G0	Zinc finger protein 687 OS=Homo sapiens OX=9606 GN=ZNF687 PE=1 SV=1	0,301787316	0,728406
Q13595	Transformer-2 protein homolog alpha OS=Homo sapiens OX=9606 GN=TRA2A PE=1 SV=1	-0,171827092	0,728073
P31146	Coronin-1A OS=Homo sapiens OX=9606 GN=CORO1A PE=1 SV=4	-0,123012716	0,727939
Q96S52	GPI transamidase component PIG-S OS=Homo sapiens OX=9606 GN=PIGS PE=1 SV=3	0,101080191	0,727879
Q9Y606	tRNA pseudouridine synthase A OS=Homo sapiens OX=9606 GN=PUS1 PE=1 SV=3	0,301098411	0,72676
Q96S97	Myeloid-associated differentiation marker OS=Homo sapiens OX=9606 GN=MYADM PE=1 SV=2	0,300685606	0,725773
O43303	Centriolar coiled-coil protein of 110 kDa OS=Homo sapiens OX=9606 GN=CCP110 PE=1 SV=3	0,15889652	0,72543
P31947	14-3-3 protein sigma OS=Homo sapiens OX=9606 GN=SFN PE=1 SV=1	0,106514791	0,723819
P04156	Major prion protein OS=Homo sapiens OX=9606 GN=PRNP PE=1 SV=1	0,208244991	0,72335
Q9Y223	Bifunctional UDP-N-acetylglucosamine 2-epimerase/N-acetylmannosamine kinase OS=Homo sapiens OX=9606 GN=GNE P	-0,087312551	0,722521
Q9BT78	COP9 signalosome complex subunit 4 OS=Homo sapiens OX=9606 GN=COPS4 PE=1 SV=1	-0,06969635	0,722408
Q8IY22	C-Maf-inducing protein OS=Homo sapiens OX=9606 GN=CMIP PE=1 SV=3	0,207478989	0,720433
Q9NTJ5	Phosphatidylinositide phosphatase SAC1 OS=Homo sapiens OX=9606 GN=SACM1L PE=1 SV=2	0,095183152	0,720354
P57088	Transmembrane protein 33 OS=Homo sapiens OX=9606 GN=TMEM33 PE=1 SV=2	0,147989965	0,719281
Q8TED0	U3 small nucleolar RNA-associated protein 15 homolog OS=Homo sapiens OX=9606 GN=UTP15 PE=1 SV=3	0,207030999	0,718727
O14530	Thioredoxin domain-containing protein 9 OS=Homo sapiens OX=9606 GN=TXNDC9 PE=1 SV=2	0,105866639	0,718685
Q00688	Peptidyl-prolyl cis-trans isomerase FKBP3 OS=Homo sapiens OX=9606 GN=FKBP3 PE=1 SV=1	-0,11293162	0,718658
Q9NZ45	CDGSH iron-sulfur domain-containing protein 1 OS=Homo sapiens OX=9606 GN=CISD1 PE=1 SV=1	0,297330063	0,717744
P10644	cAMP-dependent protein kinase type I-alpha regulatory subunit OS=Homo sapiens OX=9606 GN=PRKAR1A PE=1 SV=1	0,08048299	0,717374
P98170	E3 ubiquitin-protein ligase XIAP OS=Homo sapiens OX=9606 GN=XIAP PE=1 SV=2	0,297082456	0,717151
P41214	Eukaryotic translation initiation factor 2D OS=Homo sapiens OX=9606 GN=EIF2D PE=1 SV=3	-0,099744988	0,716654
Q9UNS2	COP9 signalosome complex subunit 3 OS=Homo sapiens OX=9606 GN=COPS3 PE=1 SV=3	0,080349579	0,715977

P04216	Thy-1 membrane glycoprotein OS=Homo sapiens OX=9606 GN=THY1 PE=1 SV=2	0,169233949	0,715681
P03886	NADH-ubiquinone oxidoreductase chain 1 OS=Homo sapiens OX=9606 GN=MT-ND1 PE=1 SV=1	-0,296014094	0,714591
Q5JRA6	Transport and Golgi organization protein 1 homolog OS=Homo sapiens OX=9606 GN=MIA3 PE=1 SV=1	-0,121028533	0,714348
P78362	SRSF protein kinase 2 OS=Homo sapiens OX=9606 GN=SRPK2 PE=1 SV=3	0,077462302	0,71362
P55263	Adenosine kinase OS=Homo sapiens OX=9606 GN=ADK PE=1 SV=2	-0,086368099	0,713364
P23528	Cofilin-1 OS=Homo sapiens OX=9606 GN=CFL1 PE=1 SV=3	0,074875382	0,71167
Q9NVP1	ATP-dependent RNA helicase DDX18 OS=Homo sapiens OX=9606 GN=DDX18 PE=1 SV=2	-0,074856945	0,711464
O75817	Ribonuclease P protein subunit p20 OS=Homo sapiens OX=9606 GN=POP7 PE=1 SV=2	0,430362536	0,71061
O43617	Trafficking protein particle complex subunit 3 OS=Homo sapiens OX=9606 GN=TRAPPC3 PE=1 SV=1	-0,13150029	0,710386
Q96BD8	Spindle and kinetochore-associated protein 1 OS=Homo sapiens OX=9606 GN=SKA1 PE=1 SV=1	-0,287909001	0,710053
Q01518	Adenylyl cyclase-associated protein 1 OS=Homo sapiens OX=9606 GN=CAP1 PE=1 SV=5	-0,058777706	0,709057
Q9H9B4	Sideroflexin-1 OS=Homo sapiens OX=9606 GN=SFXN1 PE=1 SV=4	-0,082617501	0,708946
P49069	Calcium signal-modulating cyclophilin ligand OS=Homo sapiens OX=9606 GN=CAMLG PE=1 SV=1	0,15232214	0,708647
Q15102	Platelet-activating factor acetylhydrolase IB subunit gamma OS=Homo sapiens OX=9606 GN=PAFAH1B3 PE=1 SV=1	0,093847238	0,708545
Q99627	COP9 signalosome complex subunit 8 OS=Homo sapiens OX=9606 GN=COPS8 PE=1 SV=1	-0,146055347	0,708538
Q9BVC6	Transmembrane protein 109 OS=Homo sapiens OX=9606 GN=TMEM109 PE=1 SV=1	-0,204335278	0,70847
O15382	Branched-chain-amino-acid aminotransferase, mitochondrial OS=Homo sapiens OX=9606 GN=BCAT2 PE=1 SV=2	-0,167703958	0,708384
Q5TGL8	PX domain-containing protein 1 OS=Homo sapiens OX=9606 GN=PXDC1 PE=2 SV=3	-0,293423862	0,708378
Q96G23	Ceramide synthase 2 OS=Homo sapiens OX=9606 GN=CERS2 PE=1 SV=1	0,111528546	0,708292
P59998	Actin-related protein 2/3 complex subunit 4 OS=Homo sapiens OX=9606 GN=ARPC4 PE=1 SV=3	-0,120095452	0,707975
P63220	40S ribosomal protein S21 OS=Homo sapiens OX=9606 GN=RPS21 PE=1 SV=1	-0,145861572	0,707463
O00273	DNA fragmentation factor subunit alpha OS=Homo sapiens OX=9606 GN=DFFA PE=1 SV=1	0,292989923	0,707336
Q5TC12	ATP synthase mitochondrial F1 complex assembly factor 1 OS=Homo sapiens OX=9606 GN=ATPAF1 PE=1 SV=1	-0,203831962	0,706556
Q9H4M9	EH domain-containing protein 1 OS=Homo sapiens OX=9606 GN=EHD1 PE=1 SV=2	0,05371014	0,706346
P50570	Dynamin-2 OS=Homo sapiens OX=9606 GN=DNM2 PE=1 SV=2	0,059730991	0,706332
O95819	Mitogen-activated protein kinase kinase kinase kinase 4 OS=Homo sapiens OX=9606 GN=MAP4K4 PE=1 SV=2	-0,085625788	0,706188
Q9BUL8	Programmed cell death protein 10 OS=Homo sapiens OX=9606 GN=PDCD10 PE=1 SV=1	-0,11121802	0,706002
O95139	NADH dehydrogenase [ubiquinone] 1 beta subcomplex subunit 6 OS=Homo sapiens OX=9606 GN=NDUFB6 PE=1 SV=3	-0,203619358	0,705748
Q9BW85	Splicing factor YJU2 OS=Homo sapiens OX=9606 GN=YJU2 PE=1 SV=1	-0,166985128	0,70496
Q9NX55	Huntingtin-interacting protein K OS=Homo sapiens OX=9606 GN=HYPK PE=1 SV=2	-0,291963019	0,704869
Q9H061	Transmembrane protein 126A OS=Homo sapiens OX=9606 GN=TMEM126A PE=1 SV=1	-0,291886825	0,704686
P36959	#N/D	0,042904925	0,704597
Q9Y2E5	Epididymis-specific alpha-mannosidase OS=Homo sapiens OX=9606 GN=MAN2B2 PE=1 SV=4	-0,179566926	0,704327
Q9NX63	MICOS complex subunit MIC19 OS=Homo sapiens OX=9606 GN=CHCHD3 PE=1 SV=1	-0,10399253	0,703887
Q8N3X1	Formin-binding protein 4 OS=Homo sapiens OX=9606 GN=FNBP4 PE=1 SV=3	0,291425553	0,703578
Q9Y5T5	Ubiquitin carboxyl-terminal hydrolase 16 OS=Homo sapiens OX=9606 GN=USP16 PE=1 SV=1	-0,119338992	0,702818
P62491	Ras-related protein Rab-11A OS=Homo sapiens OX=9606 GN=RAB11A PE=1 SV=3	-0,081981397	0,702554
P49916	DNA ligase 3 OS=Homo sapiens OX=9606 GN=LIG3 PE=1 SV=2	-0,11074491	0,702517
Q96HE7	ERO1-like protein alpha OS=Homo sapiens OX=9606 GN=ERO1A PE=1 SV=2	0,093108472	0,702035
Q8WUY1	Protein THEM6 OS=Homo sapiens OX=9606 GN=THEM6 PE=1 SV=2	-0,144814682	0,701664
Q9HBL0	Tensin-1 OS=Homo sapiens OX=9606 GN=TNS1 PE=1 SV=2	-0,119146601	0,701508
Q9NPE3	H/ACA ribonucleoprotein complex subunit 3 OS=Homo sapiens OX=9606 GN=NOP10 PE=1 SV=1	-0,290294782	0,700859
Q9BYG3	MKI67 FHA domain-interacting nucleolar phosphoprotein OS=Homo sapiens OX=9606 GN=NIFK PE=1 SV=1	-0,110473185	0,700517
Q15014	Mortality factor 4-like protein 2 OS=Homo sapiens OX=9606 GN=MORF4L2 PE=1 SV=1	-0,290072077	0,700324
O96028	Histone-lysine N-methyltransferase NSD2 OS=Homo sapiens OX=9606 GN=NSD2 PE=1 SV=1	-0,289885399	0,699875
P06744	Glucose-6-phosphate isomerase OS=Homo sapiens OX=9606 GN=GPI PE=1 SV=4	-0,059262201	0,699812
Q13596	Sorting nexin-1 OS=Homo sapiens OX=9606 GN=SNX1 PE=1 SV=3	0,088500237	0,698461
P62917	60S ribosomal protein L8 OS=Homo sapiens OX=9606 GN=RPL8 PE=1 SV=2	0,078642156	0,698167
Q86SR1	Polypeptide N-acetylgalactosaminyltransferase 10 OS=Homo sapiens OX=9606 GN=GALNT10 PE=1 SV=2	-0,441131565	0,698138
O75940	Survival of motor neuron-related-splicing factor 30 OS=Homo sapiens OX=9606 GN=SMNDC1 PE=1 SV=1	0,103124995	0,697062
Q96AT9	Ribulose-phosphate 3-epimerase OS=Homo sapiens OX=9606 GN=RPE PE=1 SV=1	0,165171548	0,696332
Q9C0G0	Zinc finger protein 407 OS=Homo sapiens OX=9606 GN=ZNF407 PE=1 SV=2	-0,491556613	0,696079
O95478	Ribosome biogenesis protein NSA2 homolog OS=Homo sapiens OX=9606 GN=NSA2 PE=1 SV=1	-0,209877644	0,695803
Q99447	Ethanolamine-phosphate cytidylyltransferase OS=Homo sapiens OX=9606 GN=PCYT2 PE=1 SV=1	-0,078395603	0,695604
P10155	60 kDa SS-A/Ro ribonucleoprotein OS=Homo sapiens OX=9606 GN=RO60 PE=1 SV=2	0,073328697	0,69445
Q8ND56	Protein LSM14 homolog A OS=Homo sapiens OX=9606 GN=LSM14A PE=1 SV=3	-0,143474099	0,69425
Q969E4	Transcription elongation factor A protein-like 3 OS=Homo sapiens OX=9606 GN=TCEAL3 PE=1 SV=1	-0,200490335	0,693861
Q9P0V3	SH3 domain-binding protein 4 OS=Homo sapiens OX=9606 GN=SH3BP4 PE=1 SV=1	0,200256625	0,692974
Q9UNM6	26S proteasome non-ATPase regulatory subunit 13 OS=Homo sapiens OX=9606 GN=PSMD13 PE=1 SV=2	-0,06123457	0,692956
P62875	DNA-directed RNA polymerases I, II, and III subunit RPABC5 OS=Homo sapiens OX=9606 GN=POLR2L PE=1 SV=1	0,200129693	0,692492
Q5RI15	Cytochrome c oxidase assembly protein COX20, mitochondrial OS=Homo sapiens OX=9606 GN=COX20 PE=1 SV=2	0,199731243	0,69098
P35250	Replication factor C subunit 2 OS=Homo sapiens OX=9606 GN=RFC2 PE=1 SV=3	-0,087663147	0,690753
Q5SY16	Polynucleotide 5'-hydroxyl-kinase NOL9 OS=Homo sapiens OX=9606 GN=NOL9 PE=1 SV=1	-0,14283043	0,690696
Q8TA86	Retinitis pigmentosa 9 protein OS=Homo sapiens OX=9606 GN=RP9 PE=1 SV=2	0,286070542	0,690689
Q9Y657	Spindlin-1 OS=Homo sapiens OX=9606 GN=SPIN1 PE=1 SV=3	0,14257341	0,689278
Q8TBC4	NEDD8-activating enzyme E1 catalytic subunit OS=Homo sapiens OX=9606 GN=UBA3 PE=1 SV=2	-0,087478907	0,689059
P38919	Eukaryotic initiation factor 4A-III OS=Homo sapiens OX=9606 GN=EIF4A3 PE=1 SV=4	-0,070670836	0,688629
Q13485	Mothers against decapentaplegic homolog 4 OS=Homo sapiens OX=9606 GN=SMAD4 PE=1 SV=1	-0,142374056	0,688178
P34949	Mannose-6-phosphate isomerase OS=Homo sapiens OX=9606 GN=MPI PE=1 SV=2	-0,163421599	0,688022
Q9Y6Y0	Influenza virus NS1A-binding protein OS=Homo sapiens OX=9606 GN=IVNS1ABP PE=1 SV=3	0,127866918	0,687848
Q8IVU3	Probable E3 ubiquitin-protein ligase HERC6 OS=Homo sapiens OX=9606 GN=HERC6 PE=1 SV=2	-0,103698195	0,687683
Q9Y6A9	Signal peptidase complex subunit 1 OS=Homo sapiens OX=9606 GN=SPCS1 PE=1 SV=4	0,198853264	0,687648
Q8NFZ8	Cell adhesion molecule 4 OS=Homo sapiens OX=9606 GN=CADM4 PE=1 SV=1	-0,117090598	0,687538
P0CG30	Glutathione S-transferase theta-2B OS=Homo sapiens OX=9606 GN=GSTT2B PE=1 SV=1	-0,284754013	0,687515
O95602	DNA-directed RNA polymerase I subunit RPA1 OS=Homo sapiens OX=9606 GN=POLR1A PE=1 SV=2	-0,087221955	0,686699
Q8NHV4	Protein NEDD1 OS=Homo sapiens OX=9606 GN=NEDD1 PE=1 SV=1	0,127618503	0,686313
Q9UQ53	Alpha-1,3-mannosyl-glycoprotein 4-beta-N-acetylglucosaminyltransferase B OS=Homo sapiens OX=9606 GN=MGAT4B PE	-0,198458641	0,686151
O75934	Pre-mRNA-splicing factor SPF27 OS=Homo sapiens OX=9606 GN=BCAS2 PE=1 SV=1	0,116862354	0,685991
Q13185	Chromobox protein homolog 3 OS=Homo sapiens OX=9606 GN=CBX3 PE=1 SV=4	-0,108475307	0,685853
P63000	Ras-related C3 botulinum toxin substrate 1 OS=Homo sapiens OX=9606 GN=RAC1 PE=1 SV=1	0,141883211	0,685472
P51809	Vesicle-associated membrane protein 7 OS=Homo sapiens OX=9606 GN=VAMP7 PE=1 SV=3	0,091196093	0,685249
P07947	Tyrosine-protein kinase Yes OS=Homo sapiens OX=9606 GN=YES1 PE=1 SV=3	-0,127305429	0,684379
Q9NQC7	Ubiquitin carboxyl-terminal hydrolase CYLD OS=Homo sapiens OX=9606 GN=CYLD PE=1 SV=1	0,28329752	0,684
O00193	Small acidic protein OS=Homo sapiens OX=9606 GN=SMAP PE=1 SV=1	0,162560848	0,68394
O43676	NADH dehydrogenase [ubiquinone] 1 beta subcomplex subunit 3 OS=Homo sapiens OX=9606 GN=NDUFB3 PE=1 SV=3	-0,162436594	0,683351
P45984	Mitogen-activated protein kinase 9 OS=Homo sapiens OX=9606 GN=MAPK9 PE=1 SV=2	0,141118678	0,68126
P11802	Cyclin-dependent kinase 4 OS=Homo sapiens OX=9606 GN=CDK4 PE=1 SV=2	-0,161969055	0,681136
Q9H857	5'-nucleotidase domain-containing protein 2 OS=Homo sapiens OX=9606 GN=NT5DC2 PE=1 SV=1	-0,281990493	0,680844
Q9Y2W2	WW domain-binding protein 11 OS=Homo sapiens OX=9606 GN=WBP11 PE=1 SV=1	0,090690512	0,680828
Q8TEM1	Nuclear pore membrane glycoprotein 210 OS=Homo sapiens OX=9606 GN=NUP210 PE=1 SV=3	0,126701564	0,680651
P61024	Cyclin-dependent kinases regulatory subunit 1 OS=Homo sapiens OX=9606 GN=CKS1B PE=1 SV=1	-0,161801082	0,68034

Q6P1Q9	Methyltransferase-like protein 2B OS=Homo sapiens OX=9606 GN=METTL2B PE=1 SV=3	0,281496059	0,679649
Q5JPI3	Uncharacterized protein C3orf38 OS=Homo sapiens OX=9606 GN=C3orf38 PE=1 SV=1	0,196731367	0,679603
Q9Y5A9	YTH domain-containing family protein 2 OS=Homo sapiens OX=9606 GN=YTHDF2 PE=1 SV=2	-0,107608292	0,67951
Q12904	Aminoacyl tRNA synthase complex-interacting multifunctional protein 1 OS=Homo sapiens OX=9606 GN=AIMP1 PE=1 SV=	0,08643222	0,679456
Q9BRK5	45 kDa calcium-binding protein OS=Homo sapiens OX=9606 GN=SDF4 PE=1 SV=1	-0,095233372	0,679033
O15439	Multidrug resistance-associated protein 4 OS=Homo sapiens OX=9606 GN=ABCC4 PE=1 SV=3	-0,107527831	0,678922
Q14738	Serine/threonine-protein phosphatase 2A 56 kDa regulatory subunit delta isoform OS=Homo sapiens OX=9606 GN=PPP2F	-0,074219693	0,678746
Q14678	KN motif and ankyrin repeat domain-containing protein 1 OS=Homo sapiens OX=9606 GN=KANK1 PE=1 SV=3	0,161135134	0,677187
P02461	Collagen alpha-1(III) chain OS=Homo sapiens OX=9606 GN=COL3A1 PE=1 SV=4	-0,055452995	0,676811
P49327	Fatty acid synthase OS=Homo sapiens OX=9606 GN=FASN PE=1 SV=3	-0,026409971	0,675948
O15305	Phosphomannomutase 2 OS=Homo sapiens OX=9606 GN=PMM2 PE=1 SV=1	-0,106962128	0,674792
O94817	Ubiquitin-like protein ATG12 OS=Homo sapiens OX=9606 GN=ATG12 PE=1 SV=1	0,279474006	0,674761
Q9Y3D9	28S ribosomal protein S23, mitochondrial OS=Homo sapiens OX=9606 GN=MRPS23 PE=1 SV=2	-0,195283859	0,674119
Q96QK1	Vacuolar protein sorting-associated protein 35 OS=Homo sapiens OX=9606 GN=VPS35 PE=1 SV=2	-0,04667016	0,674101
Q9H2U2	Inorganic pyrophosphatase 2, mitochondrial OS=Homo sapiens OX=9606 GN=PPA2 PE=1 SV=2	-0,125624126	0,67401
Q9H9A7	RecQ-mediated genome instability protein 1 OS=Homo sapiens OX=9606 GN=RMI1 PE=1 SV=3	-0,160431697	0,673859
Q99598	Translin-associated protein X OS=Homo sapiens OX=9606 GN=TSNAX PE=1 SV=1	-0,106829391	0,673823
Q96D46	60S ribosomal export protein NMD3 OS=Homo sapiens OX=9606 GN=NMD3 PE=1 SV=1	-0,100102638	0,673402
Q8N3R9	MAGUK p55 subfamily member 5 OS=Homo sapiens OX=9606 GN=MPP5 PE=1 SV=3	0,139647675	0,67317
Q9UGP8	Translocation protein SEC63 homolog OS=Homo sapiens OX=9606 GN=SEC63 PE=1 SV=2	0,089721484	0,672372
Q7L2J0	7SK snRNA methylphosphate capping enzyme OS=Homo sapiens OX=9606 GN=MEPCE PE=1 SV=1	-0,194803364	0,672299
Q9NZ56	Formin-2 OS=Homo sapiens OX=9606 GN=FMN2 PE=1 SV=4	0,278433056	0,672242
Q9UMX1	Suppressor of fused homolog OS=Homo sapiens OX=9606 GN=SUFU PE=1 SV=2	-0,194784594	0,672228
Q9UHB4	NADPH-dependent diflavin oxidoreductase 1 OS=Homo sapiens OX=9606 GN=NDOR1 PE=1 SV=1	-0,139475387	0,672224
Q9NRK6	ATP-binding cassette sub-family B member 10, mitochondrial OS=Homo sapiens OX=9606 GN=ABCB10 PE=1 SV=2	-0,160039573	0,672005
O75694	Nuclear pore complex protein Nup155 OS=Homo sapiens OX=9606 GN=NUP155 PE=1 SV=1	0,050662914	0,671757
Q9P270	SLAIN motif-containing protein 2 OS=Homo sapiens OX=9606 GN=SLAIN2 PE=1 SV=2	0,125252557	0,671723
O94766	Galactosylgalactosylxylosylprotein 3-beta-glucuronosyltransferase 3 OS=Homo sapiens OX=9606 GN=B3GAT3 PE=1 SV=2	0,278135253	0,671521
Q8IUC4	Rhophilin-2 OS=Homo sapiens OX=9606 GN=RHPN2 PE=1 SV=1	0,29707488	0,671385
Q9NZI8	Insulin-like growth factor 2 mRNA-binding protein 1 OS=Homo sapiens OX=9606 GN=IGF2BP1 PE=1 SV=2	0,076039016	0,671228
O43314	Inositol hexakisphosphate and diphosphoinositol-pentakisphosphate kinase 2 OS=Homo sapiens OX=9606 GN=PPIP5K2 PI	-0,139196454	0,670692
Q92599	Septin-8 OS=Homo sapiens OX=9606 GN=SEPTIN8 PE=1 SV=4	-0,081919671	0,670625
P30622	CAP-Gly domain-containing linker protein 1 OS=Homo sapiens OX=9606 GN=CLIP1 PE=1 SV=2	0,049092559	0,67058
Q63ZY3	KN motif and ankyrin repeat domain-containing protein 2 OS=Homo sapiens OX=9606 GN=KANK2 PE=1 SV=1	-0,075949434	0,670306
P08651	Nuclear factor 1 C-type OS=Homo sapiens OX=9606 GN=NFIC PE=1 SV=2	-0,114528669	0,670215
P61353	60S ribosomal protein L27 OS=Homo sapiens OX=9606 GN=RPL27 PE=1 SV=2	-0,139030524	0,669782
Q92572	AP-3 complex subunit sigma-1 OS=Homo sapiens OX=9606 GN=AP3S1 PE=1 SV=1	-0,106262421	0,66969
Q14919	Dr1-associated corepressor OS=Homo sapiens OX=9606 GN=DRAP1 PE=1 SV=3	-0,114426891	0,669529
Q8NHQ9	ATP-dependent RNA helicase DDX55 OS=Homo sapiens OX=9606 GN=DDX55 PE=1 SV=3	0,27718561	0,669222
Q9NQT5	Exosome complex component RRP40 OS=Homo sapiens OX=9606 GN=EXOSC3 PE=1 SV=3	-0,124761743	0,668704
Q9Y2Q3	Glutathione S-transferase kappa 1 OS=Homo sapiens OX=9606 GN=GSTK1 PE=1 SV=3	-0,106125613	0,668694
Q9Y3D3	28S ribosomal protein S16, mitochondrial OS=Homo sapiens OX=9606 GN=MRPS16 PE=1 SV=1	-0,159243615	0,668244
Q68D91	Metallo-beta-lactamase domain-containing protein 2 OS=Homo sapiens OX=9606 GN=MBLAC2 PE=1 SV=3	-0,27673359	0,668128
Q9BWE0	Replication initiator 1 OS=Homo sapiens OX=9606 GN=REPIN1 PE=1 SV=1	0,276648478	0,667921
P53367	Arfaptin-1 OS=Homo sapiens OX=9606 GN=ARFIP1 PE=1 SV=2	0,093744275	0,666721
Q99685	Monoglyceride lipase OS=Homo sapiens OX=9606 GN=MGLL PE=1 SV=2	0,099216117	0,666498
P60891	Ribose-phosphate pyrophosphokinase 1 OS=Homo sapiens OX=9606 GN=PRPS1 PE=1 SV=2	0,158779768	0,666053
Q96SY0	Integrator complex subunit 14 OS=Homo sapiens OX=9606 GN=INTS14 PE=1 SV=2	-0,275450097	0,665018
Q9H9T3	Elongator complex protein 3 OS=Homo sapiens OX=9606 GN=ELP3 PE=1 SV=2	-0,098953296	0,664454
Q96J84	Kin of IRRE-like protein 1 OS=Homo sapiens OX=9606 GN=KIRREL1 PE=1 SV=2	0,275196277	0,664402
Q8IY81	pre-rRNA 2'-O-ribose RNA methyltransferase FTSJ3 OS=Homo sapiens OX=9606 GN=FTSJ3 PE=1 SV=2	0,113652104	0,66431
Q99653	Calcineurin B homologous protein 1 OS=Homo sapiens OX=9606 GN=CHP1 PE=1 SV=3	0,274887743	0,663654
Q969T3	Sorting nexin-21 OS=Homo sapiens OX=9606 GN=SNX21 PE=2 SV=1	0,274755224	0,663333
Q14232	Translation initiation factor eIF-2B subunit alpha OS=Homo sapiens OX=9606 GN=EIF2B1 PE=1 SV=1	-0,137764076	0,662838
Q6Y7W6	GRB10-interacting GYF protein 2 OS=Homo sapiens OX=9606 GN=GIGYF2 PE=1 SV=1	0,063115813	0,662369
Q9BTX1	Nucleoporin NDC1 OS=Homo sapiens OX=9606 GN=NDC1 PE=1 SV=2	-0,274317982	0,662273
P08865	40S ribosomal protein SA OS=Homo sapiens OX=9606 GN=RPSA PE=1 SV=4	-0,068338572	0,662133
Q15417	Calponin-3 OS=Homo sapiens OX=9606 GN=CNN3 PE=1 SV=1	0,088517565	0,661902
P52888	Thimet oligopeptidase OS=Homo sapiens OX=9606 GN=THOP1 PE=1 SV=2	-0,05884885	0,661429
P25325	3-mercaptopyruvate sulfurtransferase OS=Homo sapiens OX=9606 GN=MPST PE=1 SV=3	-0,113053471	0,660283
Q9BZE9	Tether containing UBX domain for GLUT4 OS=Homo sapiens OX=9606 GN=ASPSCR1 PE=1 SV=1	0,104843406	0,659371
Q96E11	Ribosome-recycling factor, mitochondrial OS=Homo sapiens OX=9606 GN=MRRF PE=1 SV=1	0,137126204	0,659345
P17706	Tyrosine-protein phosphatase non-receptor type 2 OS=Homo sapiens OX=9606 GN=PTPN2 PE=1 SV=2	-0,272712284	0,658377
P54802	Alpha-N-acetylglucosaminidase OS=Homo sapiens OX=9606 GN=NAGLU PE=1 SV=2	0,139515161	0,657823
Q9Y421	Protein FAM32A OS=Homo sapiens OX=9606 GN=FAM32A PE=1 SV=2	-0,272393216	0,657603
O00506	Serine/threonine-protein kinase 25 OS=Homo sapiens OX=9606 GN=STK25 PE=1 SV=1	0,12280797	0,656715
P35269	General transcription factor IIF subunit 1 OS=Homo sapiens OX=9606 GN=GTF2F1 PE=1 SV=2	0,077367606	0,656624
P16152	Carbonyl reductase [NADPH] 1 OS=Homo sapiens OX=9606 GN=CBR1 PE=1 SV=3	0,083877321	0,65615
Q9Y3A4	Ribosomal RNA-processing protein 7 homolog A OS=Homo sapiens OX=9606 GN=RRP7A PE=1 SV=2	-0,190532386	0,656146
P47756	F-actin-capping protein subunit beta OS=Homo sapiens OX=9606 GN=CAPZB PE=1 SV=4	-0,074485154	0,655272
Q9H9C1	Spermatogenesis-defective protein 39 homolog OS=Homo sapiens OX=9606 GN=VIPAS39 PE=1 SV=1	0,297162264	0,654275
P62847	40S ribosomal protein S24 OS=Homo sapiens OX=9606 GN=RPS24 PE=1 SV=1	-0,156246189	0,654109
O15498	Synaptobrevin homolog YKT6 OS=Homo sapiens OX=9606 GN=YKT6 PE=1 SV=1	0,104096321	0,653953
Q9Y6X9	ATPase MORC2 OS=Homo sapiens OX=9606 GN=MORC2 PE=1 SV=2	-0,270836347	0,653822
Q86SQ0	Pleckstrin homology-like domain family B member 2 OS=Homo sapiens OX=9606 GN=PHLDB2 PE=1 SV=2	0,062414632	0,653787
P05165	Propionyl-CoA carboxylase alpha chain, mitochondrial OS=Homo sapiens OX=9606 GN=PCCA PE=1 SV=4	0,270764879	0,653648
Q15392	Delta(24)-sterol reductase OS=Homo sapiens OX=9606 GN=DHCR24 PE=1 SV=2	-0,122293051	0,653562
Q14624	Inter-alpha-trypsin inhibitor heavy chain H4 OS=Homo sapiens OX=9606 GN=ITIH4 PE=1 SV=4	0,18984296	0,653541
Q16666	Gamma-interferon-inducible protein 16 OS=Homo sapiens OX=9606 GN=IFI16 PE=1 SV=3	-0,060909108	0,653465
Q8WUA4	General transcription factor 3C polypeptide 2 OS=Homo sapiens OX=9606 GN=GTF3C2 PE=1 SV=2	-0,156083474	0,653343
Q53T59	HCLS1-binding protein 3 OS=Homo sapiens OX=9606 GN=HS1BP3 PE=1 SV=1	-0,156053544	0,653202
Q96ED9	Protein Hook homolog 2 OS=Homo sapiens OX=9606 GN=HOOK2 PE=1 SV=3	0,270560849	0,653153
L0R819	ASNSD1 upstream open reading frame protein OS=Homo sapiens OX=9606 GN=ASDURF PE=1 SV=1	-0,251908037	0,652799
Q13547	Histone deacetylase 1 OS=Homo sapiens OX=9606 GN=HDAC1 PE=1 SV=1	-0,087303876	0,651385
O75688	Protein phosphatase 1B OS=Homo sapiens OX=9606 GN=PPM1B PE=1 SV=1	-0,103680437	0,65094
O00399	Dynactin subunit 6 OS=Homo sapiens OX=9606 GN=DCTN6 PE=1 SV=1	0,188976984	0,650272
Q96IU4	Protein ABHD14B OS=Homo sapiens OX=9606 GN=ABHD14B PE=1 SV=1	-0,111514865	0,649958
Q9Y613	FH1/FH2 domain-containing protein 1 OS=Homo sapiens OX=9606 GN=FHOD1 PE=1 SV=3	-0,188811018	0,649645
Q7Z3U7	Protein MON2 homolog OS=Homo sapiens OX=9606 GN=MON2 PE=1 SV=3	-0,091657264	0,649553
Q96GQ7	Probable ATP-dependent RNA helicase DDX27 OS=Homo sapiens OX=9606 GN=DDX27 PE=1 SV=2	-0,103475221	0,649455
P28702	Retinoic acid receptor RXR-beta OS=Homo sapiens OX=9606 GN=RXRB PE=1 SV=2	0,155170543	0,649048

Q9P2E3	NFX1-type zinc finger-containing protein 1 OS=Homo sapiens OX=9606 GN=ZNFX1 PE=2 SV=2	-0,103407055	0,648962
Q9Y2X9	Zinc finger protein 281 OS=Homo sapiens OX=9606 GN=ZNF281 PE=1 SV=1	0,432512977	0,647455
Q12979	Active breakpoint cluster region-related protein OS=Homo sapiens OX=9606 GN=ABR PE=1 SV=2	-0,069045026	0,64731
Q7Z7L7	Protein zer-1 homolog OS=Homo sapiens OX=9606 GN=ZER1 PE=1 SV=1	0,183761514	0,647204
Q8WXF7	Atlastin-1 OS=Homo sapiens OX=9606 GN=ATL1 PE=1 SV=1	-0,134873838	0,64704
P61088	Ubiquitin-conjugating enzyme E2 N OS=Homo sapiens OX=9606 GN=UBE2N PE=1 SV=1	-0,096550283	0,645833
O75937	DnaJ homolog subfamily C member 8 OS=Homo sapiens OX=9606 GN=DNAJC8 PE=1 SV=2	0,091118278	0,645136
P02042	Hemoglobin subunit delta OS=Homo sapiens OX=9606 GN=HBD PE=1 SV=2	0,267246405	0,645094
Q9UBS9	SUN domain-containing ossification factor OS=Homo sapiens OX=9606 GN=SUCO PE=1 SV=1	0,267231537	0,645057
P12074	Cytochrome c oxidase subunit 6A1, mitochondrial OS=Homo sapiens OX=9606 GN=COX6A1 PE=1 SV=4	-0,210988114	0,644743
Q9Y5E1	Protocadherin beta-9 OS=Homo sapiens OX=9606 GN=PCDHB9 PE=2 SV=2	-0,644351785	0,644421
P35268	60S ribosomal protein L22 OS=Homo sapiens OX=9606 GN=RPL22 PE=1 SV=2	-0,15414634	0,644235
P13798	Acylamino-acid-releasing enzyme OS=Homo sapiens OX=9606 GN=APEH PE=1 SV=4	0,070968759	0,644221
O94900	Thymocyte selection-associated high mobility group box protein TOX OS=Homo sapiens OX=9606 GN=TOX PE=2 SV=3	-0,13434839	0,644176
P00846	ATP synthase subunit a OS=Homo sapiens OX=9606 GN=MT-ATP6 PE=1 SV=1	-0,266541315	0,643377
Q9NP84	Tumor necrosis factor receptor superfamily member 12A OS=Homo sapiens OX=9606 GN=TNFRSF12A PE=1 SV=1	0,589496294	0,643038
Q9UHW9	Solute carrier family 12 member 6 OS=Homo sapiens OX=9606 GN=SLC12A6 PE=1 SV=2	0,266181736	0,642502
P56377	AP-1 complex subunit sigma-2 OS=Homo sapiens OX=9606 GN=AP1S2 PE=1 SV=1	0,266119826	0,642351
P30566	Adenylosuccinate lyase OS=Homo sapiens OX=9606 GN=ADSL PE=1 SV=2	-0,075896403	0,642138
O43290	U4/U6.U5 tri-snRNP-associated protein 1 OS=Homo sapiens OX=9606 GN=SART1 PE=1 SV=1	-0,068549415	0,641909
Q9UI10	Translation initiation factor eIF-2B subunit delta OS=Homo sapiens OX=9606 GN=EIF2B4 PE=1 SV=2	-0,120333205	0,641591
Q9H8H0	Nucleolar protein 11 OS=Homo sapiens OX=9606 GN=NOL11 PE=1 SV=1	-0,186290089	0,641478
Q8IYT4	Katanin p60 ATPase-containing subunit A-like 2 OS=Homo sapiens OX=9606 GN=KATNAL2 PE=1 SV=3	-0,172368022	0,641416
Q01813	ATP-dependent 6-phosphofructokinase, platelet type OS=Homo sapiens OX=9606 GN=PFKP PE=1 SV=2	-0,051962223	0,640572
Q9Y4B5	Microtubule cross-linking factor 1 OS=Homo sapiens OX=9606 GN=MTCL1 PE=1 SV=5	-0,136514218	0,640481
Q9NX08	COMM domain-containing protein 8 OS=Homo sapiens OX=9606 GN=COMMD8 PE=1 SV=1	-0,133592424	0,640058
Q06787	Synaptic functional regulator FMR1 OS=Homo sapiens OX=9606 GN=FMR1 PE=1 SV=1	-0,095782959	0,639912
Q9H1A4	Anaphase-promoting complex subunit 1 OS=Homo sapiens OX=9606 GN=ANAPC1 PE=1 SV=1	-0,095765975	0,639781
Q9Y343	Sorting nexin-24 OS=Homo sapiens OX=9606 GN=SNX24 PE=1 SV=1	0,436251565	0,638543
P31949	Protein S100-A11 OS=Homo sapiens OX=9606 GN=S100A11 PE=1 SV=2	-0,109765002	0,638257
Q12851	Mitogen-activated protein kinase kinase kinase kinase 2 OS=Homo sapiens OX=9606 GN=MAP4K2 PE=1 SV=2	-0,264116317	0,637471
P41226	Ubiquitin-like modifier-activating enzyme 7 OS=Homo sapiens OX=9606 GN=UBA7 PE=1 SV=2	-0,152641158	0,637171
Q9P2B4	CTTNBP2 N-terminal-like protein OS=Homo sapiens OX=9606 GN=CTTNBP2NL PE=1 SV=2	-0,109587858	0,637074
P54764	Ephrin type-A receptor 4 OS=Homo sapiens OX=9606 GN=EPHA4 PE=1 SV=1	0,263130827	0,635069
Q9Y263	Phospholipase A-2-activating protein OS=Homo sapiens OX=9606 GN=PLAA PE=1 SV=2	-0,056812842	0,634817
Q8NEZ4	Histone-lysine N-methyltransferase 2C OS=Homo sapiens OX=9606 GN=KMT2C PE=1 SV=3	-0,53568727	0,63459
Q6UXH1	Cysteine-rich with EGF-like domain protein 2 OS=Homo sapiens OX=9606 GN=CRELD2 PE=1 SV=1	0,26288761	0,634476
O60828	Polyglutamine-binding protein 1 OS=Homo sapiens OX=9606 GN=PQBP1 PE=1 SV=1	0,152009641	0,634211
P20020	Plasma membrane calcium-transporting ATPase 1 OS=Homo sapiens OX=9606 GN=ATP2B1 PE=1 SV=4	0,085237852	0,633574
Q5H9U9	Probable ATP-dependent RNA helicase DDX60-like OS=Homo sapiens OX=9606 GN=DDX60L PE=2 SV=2	-0,262486463	0,633498
O75874	Isocitrate dehydrogenase [NADP] cytoplasmic OS=Homo sapiens OX=9606 GN=IDH1 PE=1 SV=2	-0,069937483	0,633361
Q7L0Y3	tRNA methyltransferase 10 homolog C OS=Homo sapiens OX=9606 GN=TRMT10C PE=1 SV=2	-0,094925252	0,633308
Q15436	Protein transport protein Sec23A OS=Homo sapiens OX=9606 GN=SEC23A PE=1 SV=2	-0,051450292	0,633225
Q8IYB3	Serine/arginine repetitive matrix protein 1 OS=Homo sapiens OX=9606 GN=SRRM1 PE=1 SV=2	0,094908541	0,633179
Q8WXX5	DnaJ homolog subfamily C member 9 OS=Homo sapiens OX=9606 GN=DNAJC9 PE=1 SV=1	0,089607901	0,632794
O94830	Phospholipase DDHD2 OS=Homo sapiens OX=9606 GN=DDHD2 PE=1 SV=2	-0,089583854	0,632598
Q8WZA0	Protein LZIC OS=Homo sapiens OX=9606 GN=LZIC PE=1 SV=1	0,184144056	0,632051
Q9C0C2	182 kDa tankyrase-1-binding protein OS=Homo sapiens OX=9606 GN=TNKS1BP1 PE=1 SV=4	-0,044143478	0,631621
O00410	Importin-5 OS=Homo sapiens OX=9606 GN=IPO5 PE=1 SV=4	-0,042005648	0,631576
O43633	Charged multivesicular body protein 2a OS=Homo sapiens OX=9606 GN=CHMP2A PE=1 SV=1	0,151340749	0,631078
P51784	Ubiquitin carboxyl-terminal hydrolase 11 OS=Homo sapiens OX=9606 GN=USP11 PE=1 SV=3	-0,089389642	0,631016
O00213	Amyloid-beta A4 precursor protein-binding family B member 1 OS=Homo sapiens OX=9606 GN=APBB1 PE=1 SV=2	-0,183574426	0,629906
P28482	Mitogen-activated protein kinase 1 OS=Homo sapiens OX=9606 GN=MAPK1 PE=1 SV=3	-0,063615678	0,629385
Q8IWA0	WD repeat-containing protein 75 OS=Homo sapiens OX=9606 GN=WDR75 PE=1 SV=1	-0,150860155	0,628828
O43149	Zinc finger ZZ-type and EF-hand domain-containing protein 1 OS=Homo sapiens OX=9606 GN=ZZEF1 PE=1 SV=6	0,094266688	0,628247
Q9H3Y8	Pancreatic progenitor cell differentiation and proliferation factor OS=Homo sapiens OX=9606 GN=PPDPF PE=1 SV=1	0,26022591	0,627982
Q5JWF2	Guanine nucleotide-binding protein G(s) subunit alpha isoforms XLas OS=Homo sapiens OX=9606 GN=GNAS PE=1 SV=2	0,074442911	0,627901
P62304	Small nuclear ribonucleoprotein E OS=Homo sapiens OX=9606 GN=SNRPE PE=1 SV=1	0,183039082	0,627891
P26374	Rab proteins geranylgeranyltransferase component A 2 OS=Homo sapiens OX=9606 GN=CHML PE=1 SV=2	0,272219733	0,627151
Q8N1G4	Leucine-rich repeat-containing protein 47 OS=Homo sapiens OX=9606 GN=LRRC47 PE=1 SV=1	-0,06172846	0,626776
O95155	Ubiquitin conjugation factor E4 B OS=Homo sapiens OX=9606 GN=UBE4B PE=1 SV=1	-0,088864338	0,626739
Q9NZJ7	Mitochondrial carrier homolog 1 OS=Homo sapiens OX=9606 GN=MTCH1 PE=1 SV=1	-0,18245461	0,625693
Q9NZB2	Constitutive coactivator of PPAR-gamma-like protein 1 OS=Homo sapiens OX=9606 GN=FAM120A PE=1 SV=2	-0,050922229	0,625668
Q9UHV9	Prefoldin subunit 2 OS=Homo sapiens OX=9606 GN=PFDN2 PE=1 SV=1	-0,117688228	0,625505
Q9GZN8	UPF0687 protein C20orf27 OS=Homo sapiens OX=9606 GN=C20orf27 PE=1 SV=3	0,130777711	0,624772
P10398	Serine/threonine-protein kinase A-Raf OS=Homo sapiens OX=9606 GN=ARAF PE=1 SV=2	-0,182183097	0,624671
Q99747	Gamma-soluble NSF attachment protein OS=Homo sapiens OX=9606 GN=NAPG PE=1 SV=1	0,084198403	0,624655
P28715	DNA repair protein complementing XP-G cells OS=Homo sapiens OX=9606 GN=ERCC5 PE=1 SV=3	-0,181988917	0,623941
O43491	Band 4.1-like protein 2 OS=Homo sapiens OX=9606 GN=EPB41L2 PE=1 SV=1	-0,052625773	0,623014
Q9NVM9	Integrator complex subunit 13 OS=Homo sapiens OX=9606 GN=INTS13 PE=1 SV=2	-0,181643812	0,622643
Q92974	Rho guanine nucleotide exchange factor 2 OS=Homo sapiens OX=9606 GN=ARHGEF2 PE=1 SV=4	-0,041986486	0,622522
O14980	Exportin-1 OS=Homo sapiens OX=9606 GN=XPO1 PE=1 SV=1	-0,038040028	0,622303
P22694	cAMP-dependent protein kinase catalytic subunit beta OS=Homo sapiens OX=9606 GN=PRKACB PE=1 SV=2	0,306559767	0,622271
Q15287	RNA-binding protein with serine-rich domain 1 OS=Homo sapiens OX=9606 GN=RNPS1 PE=1 SV=1	0,130236324	0,621839
P37837	Transaldolase OS=Homo sapiens OX=9606 GN=TALDO1 PE=1 SV=2	-0,066644998	0,621257
Q68EM7	Rho GTPase-activating protein 17 OS=Homo sapiens OX=9606 GN=ARHGAP17 PE=1 SV=1	0,062872753	0,620864
Q9BTV4	Transmembrane protein 43 OS=Homo sapiens OX=9606 GN=TMEM43 PE=1 SV=1	0,062826219	0,620331
Q96B36	Proline-rich AKT1 substrate 1 OS=Homo sapiens OX=9606 GN=AKT1S1 PE=1 SV=1	0,129911044	0,620078
Q9Y296	Trafficking protein particle complex subunit 4 OS=Homo sapiens OX=9606 GN=TRAPPC4 PE=1 SV=1	0,180621288	0,6188
Q3LXA3	Triokinase/FMN cyclase OS=Homo sapiens OX=9606 GN=TKFC PE=1 SV=2	-0,076381866	0,618318
P61619	Protein transport protein Sec61 subunit alpha isoform 1 OS=Homo sapiens OX=9606 GN=SEC61A1 PE=1 SV=2	0,087797304	0,618071
Q9Y653	Adhesion G-protein coupled receptor G1 OS=Homo sapiens OX=9606 GN=ADGRG1 PE=1 SV=2	0,256049289	0,617777
Q8IW35	Centrosomal protein of 97 kDa OS=Homo sapiens OX=9606 GN=CEP97 PE=1 SV=1	0,068413801	0,617396
O60292	Signal-induced proliferation-associated 1-like protein 3 OS=Homo sapiens OX=9606 GN=SIPA1L3 PE=1 SV=3	-0,129323534	0,6169
Q8NFG4	Folliculin OS=Homo sapiens OX=9606 GN=FLCN PE=1 SV=1	0,148125418	0,616051
O43772	Mitochondrial carnitine/acylcarnitine carrier protein OS=Homo sapiens OX=9606 GN=SLC25A20 PE=1 SV=1	-0,255308268	0,615964
Q13432	Protein unc-119 homolog A OS=Homo sapiens OX=9606 GN=UNC119 PE=1 SV=1	0,179819453	0,615788
Q9Y3I0	tRNA-splicing ligase RtcB homolog OS=Homo sapiens OX=9606 GN=RTCB PE=1 SV=1	-0,066075926	0,615117
O43293	Death-associated protein kinase 3 OS=Homo sapiens OX=9606 GN=DAPK3 PE=1 SV=1	0,179637903	0,615106
P62942	Peptidyl-prolyl cis-trans isomerase FKBP1A OS=Homo sapiens OX=9606 GN=FKBP1A PE=1 SV=2	0,128984539	0,615068
Q8NBL1	Protein O-glucosyltransferase 1 OS=Homo sapiens OX=9606 GN=POGLUT1 PE=1 SV=1	0,128954688	0,614907

Q12797	Aspartyl/asparaginyl beta-hydroxylase OS=Homo sapiens OX=9606 GN=ASPH PE=1 SV=3	-0,068166639	0,614815
Q9HCU8	DNA polymerase delta subunit 4 OS=Homo sapiens OX=9606 GN=POLD4 PE=1 SV=1	0,069181311	0,613991
O95394	Phosphoacetylglucosamine mutase OS=Homo sapiens OX=9606 GN=PGM3 PE=1 SV=1	-0,065858555	0,612775
Q9Y320	Thioredoxin-related transmembrane protein 2 OS=Homo sapiens OX=9606 GN=TMX2 PE=1 SV=1	0,105928382	0,612758
P62879	Guanine nucleotide-binding protein G(I)/G(S)/G(T) subunit beta-2 OS=Homo sapiens OX=9606 GN=GNB2 PE=1 SV=3	0,178917804	0,612402
Q9UBY8	Protein CLN8 OS=Homo sapiens OX=9606 GN=CLN8 PE=1 SV=3	0,253728371	0,612098
Q9UPN9	E3 ubiquitin-protein ligase TRIM33 OS=Homo sapiens OX=9606 GN=TRIM33 PE=1 SV=3	-0,253472108	0,611471
Q13191	E3 ubiquitin-protein ligase CBL-B OS=Homo sapiens OX=9606 GN=CBLB PE=1 SV=2	0,178456427	0,610671
Q9UK59	Lariat debranching enzyme OS=Homo sapiens OX=9606 GN=DBR1 PE=1 SV=2	-0,115211128	0,610514
Q13625	Apoptosis-stimulating of p53 protein 2 OS=Homo sapiens OX=9606 GN=TP53BP2 PE=1 SV=2	-0,098052602	0,610479
P48681	Nestin OS=Homo sapiens OX=9606 GN=NES PE=1 SV=2	-0,032995863	0,608237
Q9Y2H6	Fibronectin type-III domain-containing protein 3A OS=Homo sapiens OX=9606 GN=FNDC3A PE=1 SV=4	0,105119335	0,607409
P09622	Dihydrolipoyl dehydrogenase, mitochondrial OS=Homo sapiens OX=9606 GN=DLD PE=1 SV=2	-0,065342965	0,60723
Q86WV6	Stimulator of interferon genes protein OS=Homo sapiens OX=9606 GN=TMEM173 PE=1 SV=1	0,146124106	0,606727
O14672	Disintegrin and metalloproteinase domain-containing protein 10 OS=Homo sapiens OX=9606 GN=ADAM10 PE=1 SV=1	0,097504534	0,606568
Q14247	Src substrate cortactin OS=Homo sapiens OX=9606 GN=CTTN PE=1 SV=2	0,050349065	0,60477
Q13564	NEDD8-activating enzyme E1 regulatory subunit OS=Homo sapiens OX=9606 GN=NAE1 PE=1 SV=1	0,074897445	0,604468
P49419	Alpha-aminoadipic semialdehyde dehydrogenase OS=Homo sapiens OX=9606 GN=ALDH7A1 PE=1 SV=5	0,0630397	0,602895
Q96HR8	H/ACA ribonucleoprotein complex non-core subunit NAF1 OS=Homo sapiens OX=9606 GN=NAF1 PE=1 SV=2	-0,4049962	0,602863
Q14194	Dihydropyrimidinase-related protein 1 OS=Homo sapiens OX=9606 GN=CRMP1 PE=1 SV=1	0,061268896	0,602563
Q6IBW4	Condensin-2 complex subunit H2 OS=Homo sapiens OX=9606 GN=NCAPH2 PE=1 SV=1	-0,249684841	0,602192
Q9NYJ1	Cytochrome c oxidase assembly factor 4 homolog, mitochondrial OS=Homo sapiens OX=9606 GN=COA4 PE=1 SV=2	0,249644236	0,602092
O14981	TATA-binding protein-associated factor 172 OS=Homo sapiens OX=9606 GN=BTAF1 PE=1 SV=2	0,104237671	0,60159
Q9NZD2	Glycolipid transfer protein OS=Homo sapiens OX=9606 GN=GLTP PE=1 SV=3	0,249336567	0,601338
Q5T2S8	Armadillo repeat-containing protein 4 OS=Homo sapiens OX=9606 GN=ARMC4 PE=1 SV=1	-0,144842339	0,600766
Q15005	Signal peptidase complex subunit 2 OS=Homo sapiens OX=9606 GN=SPCS2 PE=1 SV=3	-0,113535539	0,600414
Q9NRM1	Enamelin OS=Homo sapiens OX=9606 GN=ENAM PE=1 SV=3	0,248920933	0,600319
O15042	U2 snRNP-associated SURP motif-containing protein OS=Homo sapiens OX=9606 GN=U2SURP PE=1 SV=2	-0,064683362	0,600153
Q99614	Tetratricopeptide repeat protein 1 OS=Homo sapiens OX=9606 GN=TTC1 PE=1 SV=1	-0,074401023	0,599852
Q676U5	Autophagy-related protein 16-1 OS=Homo sapiens OX=9606 GN=ATG16L1 PE=1 SV=2	-0,175402868	0,599222
Q13813	Spectrin alpha chain, non-erythrocytic 1 OS=Homo sapiens OX=9606 GN=SPTAN1 PE=1 SV=3	-0,021495466	0,598089
O75306	NADH dehydrogenase [ubiquinone] iron-sulfur protein 2, mitochondrial OS=Homo sapiens OX=9606 GN=NDUFS2 PE=1 SV	0,090283544	0,597832
Q92576	PHD finger protein 3 OS=Homo sapiens OX=9606 GN=PHF3 PE=1 SV=3	-0,243172121	0,597595
P09038	Fibroblast growth factor 2 OS=Homo sapiens OX=9606 GN=FGF2 PE=1 SV=3	-0,125732843	0,597542
O95169	NADH dehydrogenase [ubiquinone] 1 beta subcomplex subunit 8, mitochondrial OS=Homo sapiens OX=9606 GN=NDUFB8	-0,247513926	0,596867
Q8WVT3	Trafficking protein particle complex subunit 12 OS=Homo sapiens OX=9606 GN=TRAPPC12 PE=1 SV=3	-0,247347146	0,596458
Q96ST2	Protein IWS1 homolog OS=Homo sapiens OX=9606 GN=IWS1 PE=1 SV=2	0,112740421	0,595632
Q9H0A8	COMM domain-containing protein 4 OS=Homo sapiens OX=9606 GN=COMMD4 PE=1 SV=1	0,095923724	0,595319
Q53H12	Acylglycerol kinase, mitochondrial OS=Homo sapiens OX=9606 GN=AGK PE=1 SV=2	-0,112597532	0,594774
P10619	Lysosomal protective protein OS=Homo sapiens OX=9606 GN=CTSA PE=1 SV=2	0,089865006	0,594655
Q9NRA8	Eukaryotic translation initiation factor 4E transporter OS=Homo sapiens OX=9606 GN=EIF4ENIF1 PE=1 SV=2	-0,246491131	0,594357
Q86YR5	G-protein-signaling modulator 1 OS=Homo sapiens OX=9606 GN=GPSM1 PE=1 SV=2	-0,066086756	0,5932
Q9UEY8	Gamma-adducin OS=Homo sapiens OX=9606 GN=ADD3 PE=1 SV=1	0,09550564	0,592351
Q9NVC6	Mediator of RNA polymerase II transcription subunit 17 OS=Homo sapiens OX=9606 GN=MED17 PE=1 SV=2	-0,245658154	0,592311
Q8WWK9	Cytoskeleton-associated protein 2 OS=Homo sapiens OX=9606 GN=CKAP2 PE=1 SV=1	0,142867443	0,591601
P63010	AP-2 complex subunit beta OS=Homo sapiens OX=9606 GN=AP2B1 PE=1 SV=1	-0,039241788	0,591525
Q96FQ6	Protein S100-A16 OS=Homo sapiens OX=9606 GN=S100A16 PE=1 SV=1	-0,112023986	0,591331
Q86SZ2	Trafficking protein particle complex subunit 6B OS=Homo sapiens OX=9606 GN=TRAPPC6B PE=1 SV=1	0,245160008	0,591088
Q92540	Protein SMG7 OS=Homo sapiens OX=9606 GN=SMG7 PE=1 SV=2	-0,173026068	0,590325
P09496	Clathrin light chain A OS=Homo sapiens OX=9606 GN=CLTA PE=1 SV=1	-0,095112476	0,589564
Q9NXW2	DnaJ homolog subfamily B member 12 OS=Homo sapiens OX=9606 GN=DNAJB12 PE=1 SV=5	0,124182717	0,58922
Q9NZ09	Ubiquitin-associated protein 1 OS=Homo sapiens OX=9606 GN=UBAP1 PE=1 SV=1	0,123887095	0,587635
Q8WYP5	Protein ELYS OS=Homo sapiens OX=9606 GN=AHCTF1 PE=1 SV=3	0,056923323	0,58754
Q14680	Maternal embryonic leucine zipper kinase OS=Homo sapiens OX=9606 GN=MELK PE=1 SV=3	-0,300710925	0,586746
Q96EY4	Translation machinery-associated protein 16 OS=Homo sapiens OX=9606 GN=TMA16 PE=1 SV=2	-0,167809443	0,586669
P36969	Phospholipid hydroperoxide glutathione peroxidase OS=Homo sapiens OX=9606 GN=GPX4 PE=1 SV=3	-0,141651276	0,585967
Q9UIC8	Leucine carboxyl methyltransferase 1 OS=Homo sapiens OX=9606 GN=LCMT1 PE=1 SV=2	-0,111106718	0,585832
P05412	Transcription factor AP-1 OS=Homo sapiens OX=9606 GN=JUN PE=1 SV=2	-0,171805387	0,585762
Q9HC36	rRNA methyltransferase 3, mitochondrial OS=Homo sapiens OX=9606 GN=MRM3 PE=1 SV=2	-0,17160741	0,585022
Q8NBJ7	Inactive C-alpha-formylglycine-generating enzyme 2 OS=Homo sapiens OX=9606 GN=SUMF2 PE=1 SV=2	0,123376772	0,584901
Q8N4Q1	Mitochondrial intermembrane space import and assembly protein 40 OS=Homo sapiens OX=9606 GN=CHCHD4 PE=1 SV=1	-0,141040606	0,583142
O43464	Serine protease HTRA2, mitochondrial OS=Homo sapiens OX=9606 GN=HTRA2 PE=1 SV=2	0,123000387	0,582886
Q8N3C0	Activating signal cointegrator 1 complex subunit 3 OS=Homo sapiens OX=9606 GN=ASCC3 PE=1 SV=3	0,075702618	0,582881
P07203	Glutathione peroxidase 1 OS=Homo sapiens OX=9606 GN=GPX1 PE=1 SV=4	0,083424097	0,582831
Q9Y2T2	AP-3 complex subunit mu-1 OS=Homo sapiens OX=9606 GN=AP3M1 PE=1 SV=1	0,075599621	0,58197
Q9NQ88	Fructose-2,6-bisphosphatase TIGAR OS=Homo sapiens OX=9606 GN=TIGAR PE=1 SV=1	0,170689978	0,581594
Q99996	A-kinase anchor protein 9 OS=Homo sapiens OX=9606 GN=AKAP9 PE=1 SV=4	0,241080682	0,581061
Q9GZY8	Mitochondrial fission factor OS=Homo sapiens OX=9606 GN=MFF PE=1 SV=1	-0,110288235	0,580934
Q96SB3	Neurabin-2 OS=Homo sapiens OX=9606 GN=PPP1R9B PE=1 SV=3	-0,110054484	0,579537
P26572	Alpha-1,3-mannosyl-glycoprotein 2-beta-N-acetylglucosaminyltransferase OS=Homo sapiens OX=9606 GN=MGAT1 PE=1 S	-0,109986052	0,579128
P50135	Histamine N-methyltransferase OS=Homo sapiens OX=9606 GN=HNMT PE=1 SV=1	0,093588195	0,578781
Q13347	Eukaryotic translation initiation factor 3 subunit I OS=Homo sapiens OX=9606 GN=EIF3I PE=1 SV=1	-0,060792901	0,57818
Q9H7C9	Mth938 domain-containing protein OS=Homo sapiens OX=9606 GN=AAMDC PE=1 SV=1	-0,139847189	0,577626
Q6ZMG9	Ceramide synthase 6 OS=Homo sapiens OX=9606 GN=CERS6 PE=1 SV=1	-0,239677445	0,577609
Q96G46	tRNA-dihydrouridine(47) synthase [NAD(P)(+)]-like OS=Homo sapiens OX=9606 GN=DUS3L PE=1 SV=2	-0,139826624	0,577531
P08962	CD63 antigen OS=Homo sapiens OX=9606 GN=CD63 PE=1 SV=2	-0,239481796	0,577128
P02462	Collagen alpha-1(IV) chain OS=Homo sapiens OX=9606 GN=COL4A1 PE=1 SV=4	0,109600989	0,576827
P48960	CD97 antigen OS=Homo sapiens OX=9606 GN=CD97 PE=1 SV=4	-0,071907077	0,576784
Q9NQ55	Suppressor of SWI4 1 homolog OS=Homo sapiens OX=9606 GN=PPAN PE=2 SV=1	0,109574098	0,576667
P22090	40S ribosomal protein S4, Y isoform 1 OS=Homo sapiens OX=9606 GN=RPS4Y1 PE=1 SV=2	0,069131014	0,576513
Q969H8	Myeloid-derived growth factor OS=Homo sapiens OX=9606 GN=MYDGF PE=1 SV=1	-0,109535806	0,576438
P84095	Rho-related GTP-binding protein RhoG OS=Homo sapiens OX=9606 GN=RHOG PE=1 SV=1	0,109432537	0,575822
Q6IQ49	Replication stress response regulator SDE2 OS=Homo sapiens OX=9606 GN=SDE2 PE=1 SV=1	0,169004833	0,575305
Q9UGJ1	Gamma-tubulin complex component 4 OS=Homo sapiens OX=9606 GN=TUBGCP4 PE=1 SV=1	0,238535433	0,574798
Q8NBF2	NHL repeat-containing protein 2 OS=Homo sapiens OX=9606 GN=NHLRC2 PE=1 SV=1	0,074640296	0,573505
Q12933	TNF receptor-associated factor 2 OS=Homo sapiens OX=9606 GN=TRAF2 PE=1 SV=2	0,138830087	0,572931
Q99470	Stromal cell-derived factor 2 OS=Homo sapiens OX=9606 GN=SDF2 PE=1 SV=2	0,168284326	0,572617
Q9UBG0	C-type mannose receptor 2 OS=Homo sapiens OX=9606 GN=MRC2 PE=1 SV=2	0,048923798	0,572591
Q6P9B9	Integrator complex subunit 5 OS=Homo sapiens OX=9606 GN=INTS5 PE=1 SV=1	0,13872903	0,572465
P42677	40S ribosomal protein S27 OS=Homo sapiens OX=9606 GN=RPS27 PE=1 SV=3	-0,035202563	0,572059
Q96RU3	Formin-binding protein 1 OS=Homo sapiens OX=9606 GN=FNBP1 PE=1 SV=2	0,120895287	0,57164

Q96GX9	Methylthioribulose-1-phosphate dehydratase OS=Homo sapiens OX=9606 GN=APIP PE=1 SV=1	0,167907913	0,571214
Q7Z4V5	Hepatoma-derived growth factor-related protein 2 OS=Homo sapiens OX=9606 GN=HDGFL2 PE=1 SV=1	0,086746134	0,571097
Q15365	Poly(rC)-binding protein 1 OS=Homo sapiens OX=9606 GN=PCBP1 PE=1 SV=2	-0,081939191	0,570968
Q9BXK5	Bcl-2-like protein 13 OS=Homo sapiens OX=9606 GN=BCL2L13 PE=1 SV=1	0,167737629	0,570579
O75110	Probable phospholipid-transporting ATPase IIA OS=Homo sapiens OX=9606 GN=ATP9A PE=1 SV=3	-0,188609843	0,56977
Q9NPI6	mRNA-decapping enzyme 1A OS=Homo sapiens OX=9606 GN=DCP1A PE=1 SV=3	0,167506863	0,569719
O43583	Density-regulated protein OS=Homo sapiens OX=9606 GN=DENR PE=1 SV=2	0,120526909	0,569676
Q5JTZ9	Alanine--tRNA ligase, mitochondrial OS=Homo sapiens OX=9606 GN=AARS2 PE=1 SV=1	-0,092291797	0,569643
Q8N7X0	Androglobin OS=Homo sapiens OX=9606 GN=ADGB PE=2 SV=3	-0,23636727	0,569459
Q14696	LRP chaperone MESD OS=Homo sapiens OX=9606 GN=MESD PE=1 SV=2	0,099285448	0,569123
A6ZKI3	Retrotransposon Gag-like protein 8B OS=Homo sapiens OX=9606 GN=RTL8C PE=1 SV=1	-0,236139435	0,568897
Q92917	G-patch domain and KOW motifs-containing protein OS=Homo sapiens OX=9606 GN=GPKOW PE=1 SV=2	-0,074071243	0,568497
P07311	Acylphosphatase-1 OS=Homo sapiens OX=9606 GN=ACYP1 PE=1 SV=2	0,235964675	0,568467
P41250	Glycine--tRNA ligase OS=Homo sapiens OX=9606 GN=GARS PE=1 SV=3	0,046018487	0,567755
Q9UEW8	STE20/SPS1-related proline-alanine-rich protein kinase OS=Homo sapiens OX=9606 GN=STK39 PE=1 SV=3	0,108076855	0,56774
O60507	Protein-tyrosine sulfotransferase 1 OS=Homo sapiens OX=9606 GN=TPST1 PE=1 SV=1	-0,235662174	0,567721
Q9ULK4	Mediator of RNA polymerase II transcription subunit 23 OS=Homo sapiens OX=9606 GN=MED23 PE=1 SV=2	-0,086289676	0,567667
Q5T4S7	E3 ubiquitin-protein ligase UBR4 OS=Homo sapiens OX=9606 GN=UBR4 PE=1 SV=1	-0,023414563	0,567344
Q9Y2I1	Nischarin OS=Homo sapiens OX=9606 GN=NISCH PE=1 SV=3	0,235485186	0,567285
Q96BX8	MOB kinase activator 3A OS=Homo sapiens OX=9606 GN=MOB3A PE=1 SV=1	-0,235460849	0,567225
O94875	Sorbin and SH3 domain-containing protein 2 OS=Homo sapiens OX=9606 GN=SORBS2 PE=1 SV=3	0,235083062	0,566294
Q9NTI5	Sister chromatid cohesion protein PDS5 homolog B OS=Homo sapiens OX=9606 GN=PDS5B PE=1 SV=1	-0,051452848	0,566055
Q9H4A6	Golgi phosphoprotein 3 OS=Homo sapiens OX=9606 GN=GOLPH3 PE=1 SV=1	0,091758443	0,565893
Q6R327	Rapamycin-insensitive companion of mTOR OS=Homo sapiens OX=9606 GN=RICTOR PE=1 SV=1	0,41821066	0,565753
Q9BT22	Chitobiosyldiphosphodolichol beta-mannosyltransferase OS=Homo sapiens OX=9606 GN=ALG1 PE=1 SV=2	-0,234719798	0,565399
Q8IWI9	MAX gene-associated protein OS=Homo sapiens OX=9606 GN=MGA PE=1 SV=4	-0,087339487	0,564552
Q96SB4	SRSF protein kinase 1 OS=Homo sapiens OX=9606 GN=SRPK1 PE=1 SV=2	-0,107445441	0,563984
Q8N0T1	Ribosomal biogenesis factor OS=Homo sapiens OX=9606 GN=RBIS PE=1 SV=4	-0,047089917	0,563818
P51970	NADH dehydrogenase [ubiquinone] 1 alpha subcomplex subunit 8 OS=Homo sapiens OX=9606 GN=NDUFA8 PE=1 SV=3	-0,098393533	0,563314
Q15046	Lysine--tRNA ligase OS=Homo sapiens OX=9606 GN=KARS PE=1 SV=3	0,046474426	0,562904
P54578	Ubiquitin carboxyl-terminal hydrolase 14 OS=Homo sapiens OX=9606 GN=USP14 PE=1 SV=3	0,050129791	0,562742
Q99595	Mitochondrial import inner membrane translocase subunit Tim17-A OS=Homo sapiens OX=9606 GN=TIMM17A PE=1 SV=:	-0,233565943	0,562554
Q14160	Protein scribble homolog OS=Homo sapiens OX=9606 GN=SCRIB PE=1 SV=4	0,065244603	0,562311
O95163	Elongator complex protein 1 OS=Homo sapiens OX=9606 GN=ELP1 PE=1 SV=3	-0,053474291	0,56204
Q6ZW31	Rho GTPase-activating protein SYDE1 OS=Homo sapiens OX=9606 GN=SYDE1 PE=1 SV=1	0,13634126	0,561469
Q92925	SWI/SNF-related matrix-associated actin-dependent regulator of chromatin subfamily D member 2 OS=Homo sapiens OX=	-0,136332845	0,56143
P61962	DDB1- and CUL4-associated factor 7 OS=Homo sapiens OX=9606 GN=DCAF7 PE=1 SV=1	-0,098050016	0,56108
Q96HY6	DDRGK domain-containing protein 1 OS=Homo sapiens OX=9606 GN=DDRGK1 PE=1 SV=2	0,106886579	0,560663
Q9NRY5	Protein FAM114A2 OS=Homo sapiens OX=9606 GN=FAM114A2 PE=1 SV=4	0,091012689	0,560657
Q9BVK2	Probable dolichyl pyrophosphate Glc1Man9GlcNAc2 alpha-1,3-glucosyltransferase OS=Homo sapiens OX=9606 GN=ALG8	0,232765512	0,56058
P16298	Serine/threonine-protein phosphatase 2B catalytic subunit beta isoform OS=Homo sapiens OX=9606 GN=PPP3CB PE=1 SV	0,106802683	0,560164
P82979	SAP domain-containing ribonucleoprotein OS=Homo sapiens OX=9606 GN=SARNP PE=1 SV=3	0,09786584	0,559883
P40937	Replication factor C subunit 5 OS=Homo sapiens OX=9606 GN=RFC5 PE=1 SV=1	-0,064996761	0,559863
Q8N1B4	Vacuolar protein sorting-associated protein 52 homolog OS=Homo sapiens OX=9606 GN=VPS52 PE=1 SV=1	0,135859895	0,559256
Q13555	Calcium/calmodulin-dependent protein kinase type II subunit gamma OS=Homo sapiens OX=9606 GN=CAMK2G PE=1 SV=	-0,106560113	0,558724
Q86TB9	Protein PAT1 homolog 1 OS=Homo sapiens OX=9606 GN=PATL1 PE=1 SV=2	0,135665614	0,558363
Q01650	Large neutral amino acids transporter small subunit 1 OS=Homo sapiens OX=9606 GN=SLC7A5 PE=1 SV=2	0,135560864	0,557882
Q9GZR7	ATP-dependent RNA helicase DDX24 OS=Homo sapiens OX=9606 GN=DDX24 PE=1 SV=1	-0,076260403	0,557502
Q9NPI1	Bromodomain-containing protein 7 OS=Homo sapiens OX=9606 GN=BRD7 PE=1 SV=1	-0,231475041	0,557397
P0C221	Coiled-coil domain-containing protein 175 OS=Homo sapiens OX=9606 GN=CCDC175 PE=4 SV=2	0,092879739	0,557164
P83436	Conserved oligomeric Golgi complex subunit 7 OS=Homo sapiens OX=9606 GN=COG7 PE=1 SV=1	-0,164074334	0,556942
O15504	Nucleoporin-like protein 2 OS=Homo sapiens OX=9606 GN=NUPL2 PE=1 SV=1	0,231244187	0,556827
Q14257	Reticulocalbin-2 OS=Homo sapiens OX=9606 GN=RCN2 PE=1 SV=1	-0,069716168	0,556688
P13929	#N/D	0,11798785	0,556172
Q9BTT6	Leucine-rich repeat-containing protein 1 OS=Homo sapiens OX=9606 GN=LRRC1 PE=1 SV=1	0,230859108	0,555877
P28340	DNA polymerase delta catalytic subunit OS=Homo sapiens OX=9606 GN=POLD1 PE=1 SV=2	-0,064539114	0,55535
A6NHL2	Tubulin alpha chain-like 3 OS=Homo sapiens OX=9606 GN=TUBAL3 PE=1 SV=2	-0,163321119	0,554143
P55327	Tumor protein D52 OS=Homo sapiens OX=9606 GN=TPD52 PE=1 SV=2	-0,096851166	0,553297
O15514	DNA-directed RNA polymerase II subunit RPB4 OS=Homo sapiens OX=9606 GN=POLR2D PE=1 SV=1	-0,105628043	0,553198
P29144	Tripeptidyl-peptidase 2 OS=Homo sapiens OX=9606 GN=TPP2 PE=1 SV=4	-0,0435231	0,553043
Q6XZF7	Dynamin-binding protein OS=Homo sapiens OX=9606 GN=DNMBP PE=1 SV=1	0,089756777	0,551863
O60443	Gasdermin-E OS=Homo sapiens OX=9606 GN=GSDME PE=1 SV=2	-0,072127037	0,551458
Q01844	RNA-binding protein EWS OS=Homo sapiens OX=9606 GN=EWSR1 PE=1 SV=1	-0,117092426	0,551424
Q9UHA3	Probable ribosome biogenesis protein RLP24 OS=Homo sapiens OX=9606 GN=RSL24D1 PE=1 SV=1	-0,131498477	0,550429
A1X283	SH3 and PX domain-containing protein 2B OS=Homo sapiens OX=9606 GN=SH3PXD2B PE=1 SV=3	0,083901838	0,549791
Q9Y265	RuvB-like 1 OS=Homo sapiens OX=9606 GN=RUVBL1 PE=1 SV=1	-0,075335129	0,549785
O15484	Calpain-5 OS=Homo sapiens OX=9606 GN=CAPN5 PE=1 SV=2	-0,096283983	0,549623
O95140	Mitofusin-2 OS=Homo sapiens OX=9606 GN=MFN2 PE=1 SV=3	-0,133746595	0,549557
Q9Y3D6	Mitochondrial fission 1 protein OS=Homo sapiens OX=9606 GN=FIS1 PE=1 SV=2	-0,161899039	0,548861
Q96P48	Arf-GAP with Rho-GAP domain, ANK repeat and PH domain-containing protein 1 OS=Homo sapiens OX=9606 GN=ARAP1 F	-0,075203889	0,548692
O60613	Selenoprotein F OS=Homo sapiens OX=9606 GN=SELENOF PE=1 SV=4	0,22790242	0,548577
Q9NU22	Midasin OS=Homo sapiens OX=9606 GN=MDN1 PE=1 SV=2	0,161822229	0,548576
P48426	Phosphatidylinositol 5-phosphate 4-kinase type-2 alpha OS=Homo sapiens OX=9606 GN=PIP4K2A PE=1 SV=2	-0,096098148	0,54842
Q9ULH0	Kinase D-interacting substrate of 220 kDa OS=Homo sapiens OX=9606 GN=KIDINS220 PE=1 SV=3	0,133459563	0,548242
Q8N684	Cleavage and polyadenylation specificity factor subunit 7 OS=Homo sapiens OX=9606 GN=CPSF7 PE=1 SV=1	-0,083573687	0,547344
P54709	Sodium/potassium-transporting ATPase subunit beta-3 OS=Homo sapiens OX=9606 GN=ATP1B3 PE=1 SV=1	0,133093011	0,546563
Q5T2T1	MAGUK p55 subfamily member 7 OS=Homo sapiens OX=9606 GN=MPP7 PE=1 SV=1	0,078797932	0,546049
Q14151	Scaffold attachment factor B2 OS=Homo sapiens OX=9606 GN=SAFB2 PE=1 SV=1	0,104357321	0,54568
P20674	Cytochrome c oxidase subunit 5A, mitochondrial OS=Homo sapiens OX=9606 GN=COX5A PE=1 SV=2	-0,095636286	0,545433
Q99570	Phosphoinositide 3-kinase regulatory subunit 4 OS=Homo sapiens OX=9606 GN=PIK3R4 PE=1 SV=3	0,074801846	0,545347
O96019	Actin-like protein 6A OS=Homo sapiens OX=9606 GN=ACTL6A PE=1 SV=1	0,065778339	0,544598
Q7KZI7	Serine/threonine-protein kinase MARK2 OS=Homo sapiens OX=9606 GN=MARK2 PE=1 SV=2	0,081505575	0,544574
Q07020	60S ribosomal protein L18 OS=Homo sapiens OX=9606 GN=RPL18 PE=1 SV=2	-0,083139504	0,54411
P11177	Pyruvate dehydrogenase E1 component subunit beta, mitochondrial OS=Homo sapiens OX=9606 GN=PDHB PE=1 SV=3	0,068322013	0,543982
P29034	Protein S100-A2 OS=Homo sapiens OX=9606 GN=S100A2 PE=1 SV=3	-0,225955461	0,543767
Q8NDV7	Trinucleotide repeat-containing gene 6A protein OS=Homo sapiens OX=9606 GN=TNRC6A PE=1 SV=2	0,509231505	0,54338
Q13568	Interferon regulatory factor 5 OS=Homo sapiens OX=9606 GN=IRF5 PE=1 SV=2	-0,109195783	0,543307
Q6P1A2	Lysophospholipid acyltransferase 5 OS=Homo sapiens OX=9606 GN=LPCAT3 PE=1 SV=1	0,3420618	0,543302
P16070	CD44 antigen OS=Homo sapiens OX=9606 GN=CD44 PE=1 SV=3	-0,082975992	0,542893
Q86V48	Leucine zipper protein 1 OS=Homo sapiens OX=9606 GN=LUZP1 PE=1 SV=2	-0,051781351	0,541681
Q01973	Inactive tyrosine-protein kinase transmembrane receptor ROR1 OS=Homo sapiens OX=9606 GN=ROR1 PE=1 SV=2	0,225047873	0,541524

Q14676	Mediator of DNA damage checkpoint protein 1 OS=Homo sapiens OX=9606 GN=MDC1 PE=1 SV=3	-0,131958761	0,541373
Q9UNQ2	Probable dimethyladenosine transferase OS=Homo sapiens OX=9606 GN=DIMT1 PE=1 SV=1	-0,103560593	0,540977
Q14814	Myocyte-specific enhancer factor 2D OS=Homo sapiens OX=9606 GN=MEF2D PE=1 SV=1	-0,224560081	0,540318
P17987	T-complex protein 1 subunit alpha OS=Homo sapiens OX=9606 GN=TCP1 PE=1 SV=1	0,044035431	0,539745
P51531	Probable global transcription activator SNF2L2 OS=Homo sapiens OX=9606 GN=SMARCA2 PE=1 SV=2	0,224287777	0,539645
Q96LB3	Intraflagellar transport protein 74 homolog OS=Homo sapiens OX=9606 GN=IFT74 PE=1 SV=1	-0,258401306	0,539121
Q14103	Heterogeneous nuclear ribonucleoprotein D0 OS=Homo sapiens OX=9606 GN=HNRNPD PE=1 SV=1	-0,065182995	0,538973
Q16851	UTP--glucose-1-phosphate uridylyltransferase OS=Homo sapiens OX=9606 GN=UGP2 PE=1 SV=5	0,054147397	0,538937
P17980	26S proteasome regulatory subunit 6A OS=Homo sapiens OX=9606 GN=PSMC3 PE=1 SV=3	-0,057173764	0,538874
Q96M27	Protein PRRC1 OS=Homo sapiens OX=9606 GN=PRRC1 PE=1 SV=1	-0,087753266	0,537892
O43709	Probable 18S rRNA (guanine-N(7))-methyltransferase OS=Homo sapiens OX=9606 GN=BUD23 PE=1 SV=2	-0,131069043	0,537307
O75030	#N/D	-0,223188946	0,536928
Q03113	Guanine nucleotide-binding protein subunit alpha-12 OS=Homo sapiens OX=9606 GN=GNA12 PE=1 SV=4	-0,223047812	0,536579
Q9P0S3	ORM1-like protein 1 OS=Homo sapiens OX=9606 GN=ORMDL1 PE=1 SV=1	-0,088177164	0,536458
Q07021	Complement component 1 Q subcomponent-binding protein, mitochondrial OS=Homo sapiens OX=9606 GN=C1QBP PE=1	0,082102564	0,536401
Q9Y3R5	Protein dopey-2 OS=Homo sapiens OX=9606 GN=DOP1B PE=1 SV=5	-0,158443929	0,536051
Q6P1N9	Putative deoxyribonuclease TATDN1 OS=Homo sapiens OX=9606 GN=TATDN1 PE=1 SV=2	-0,130614406	0,535231
Q9Y3I1	F-box only protein 7 OS=Homo sapiens OX=9606 GN=FBXO7 PE=1 SV=1	0,31440937	0,534675
P21291	Cysteine and glycine-rich protein 1 OS=Homo sapiens OX=9606 GN=CSRP1 PE=1 SV=3	-0,067285275	0,534575
Q12894	Interferon-related developmental regulator 2 OS=Homo sapiens OX=9606 GN=IFRD2 PE=1 SV=3	-0,222179917	0,534432
Q9H1R3	Myosin light chain kinase 2, skeletal/cardiac muscle OS=Homo sapiens OX=9606 GN=MYLK2 PE=1 SV=3	-0,31279325	0,534241
Q9NRF9	DNA polymerase epsilon subunit 3 OS=Homo sapiens OX=9606 GN=POLE3 PE=1 SV=1	-0,157949845	0,534222
Q13190	Syntaxin-5 OS=Homo sapiens OX=9606 GN=STX5 PE=1 SV=2	-0,13017974	0,533248
P53618	Coatomer subunit beta OS=Homo sapiens OX=9606 GN=COPB1 PE=1 SV=3	-0,038622153	0,533106
Q9NR48	Histone-lysine N-methyltransferase ASH1L OS=Homo sapiens OX=9606 GN=ASH1L PE=1 SV=2	0,157887056	0,532948
Q8WX93	Palladin OS=Homo sapiens OX=9606 GN=PALLD PE=1 SV=3	-0,221410736	0,532529
O15020	Spectrin beta chain, non-erythrocytic 2 OS=Homo sapiens OX=9606 GN=SPTBN2 PE=1 SV=3	-0,157474244	0,532462
O75340	Programmed cell death protein 6 OS=Homo sapiens OX=9606 GN=PDCD6 PE=1 SV=1	-0,081478727	0,531774
Q8TBC3	SH3KBP1-binding protein 1 OS=Homo sapiens OX=9606 GN=SHKBP1 PE=1 SV=2	-0,220992027	0,531493
Q9H444	Charged multivesicular body protein 4b OS=Homo sapiens OX=9606 GN=CHMP4B PE=1 SV=1	-0,06692907	0,531351
Q9BZ29	Dedicator of cytokinesis protein 9 OS=Homo sapiens OX=9606 GN=DOCK9 PE=1 SV=2	-0,157141035	0,531229
Q9H000	Probable E3 ubiquitin-protein ligase makorin-2 OS=Homo sapiens OX=9606 GN=MKRN2 PE=1 SV=2	0,129644387	0,530806
C9JRZ8	Aldo-keto reductase family 1 member B15 OS=Homo sapiens OX=9606 GN=AKR1B15 PE=1 SV=2	0,156101231	0,530782
P55769	NHP2-like protein 1 OS=Homo sapiens OX=9606 GN=SNU13 PE=1 SV=3	-0,101813391	0,530689
Q8N122	Regulatory-associated protein of mTOR OS=Homo sapiens OX=9606 GN=RPTOR PE=1 SV=1	0,12925245	0,52902
O14933	Ubiquitin/ISG15-conjugating enzyme E2 L6 OS=Homo sapiens OX=9606 GN=UBE2L6 PE=1 SV=4	0,081095284	0,528935
P08123	Collagen alpha-2(I) chain OS=Homo sapiens OX=9606 GN=COL1A2 PE=1 SV=7	0,056227333	0,528698
Q9Y217	Myotubularin-related protein 6 OS=Homo sapiens OX=9606 GN=MTMR6 PE=1 SV=3	0,072628706	0,527344
Q03701	CCAAT/enhancer-binding protein zeta OS=Homo sapiens OX=9606 GN=CEBPZ PE=1 SV=3	-0,066482785	0,527318
Q9Y3D0	Cytosolic iron-sulfur assembly component 2B OS=Homo sapiens OX=9606 GN=CIAO2B PE=1 SV=1	0,155974783	0,526917
Q8NCG7	Sn1-specific diacylglycerol lipase beta OS=Homo sapiens OX=9606 GN=DAGLB PE=1 SV=2	0,155798652	0,526266
Q8N5W9	Refilin-B OS=Homo sapiens OX=9606 GN=RFLNB PE=1 SV=1	-0,155689645	0,525863
Q8N9N8	Probable RNA-binding protein EIF1AD OS=Homo sapiens OX=9606 GN=EIF1AD PE=1 SV=1	-0,218701914	0,525825
O95295	SNARE-associated protein Snapin OS=Homo sapiens OX=9606 GN=SNAPIN PE=1 SV=1	-0,128543101	0,52579
O43708	Maleylacetoacetate isomerase OS=Homo sapiens OX=9606 GN=GSTZ1 PE=1 SV=3	0,155576241	0,525444
O75439	Mitochondrial-processing peptidase subunit beta OS=Homo sapiens OX=9606 GN=PMPCB PE=1 SV=2	-0,069102949	0,525181
Q6P3X3	Tetratricopeptide repeat protein 27 OS=Homo sapiens OX=9606 GN=TTC27 PE=1 SV=1	-0,15538583	0,524741
P63241	Eukaryotic translation initiation factor 5A-1 OS=Homo sapiens OX=9606 GN=EIF5A PE=1 SV=2	0,100793928	0,524703
Q9NPF5	DNA methyltransferase 1-associated protein 1 OS=Homo sapiens OX=9606 GN=DMAP1 PE=1 SV=1	0,138694832	0,524383
P25208	Nuclear transcription factor Y subunit beta OS=Homo sapiens OX=9606 GN=NFYB PE=1 SV=2	-0,217940195	0,523939
Q9BPX5	Actin-related protein 2/3 complex subunit 5-like protein OS=Homo sapiens OX=9606 GN=ARPC5L PE=1 SV=1	0,100625147	0,523713
Q86VR2	Reticulophagy regulator 3 OS=Homo sapiens OX=9606 GN=RETREG3 PE=1 SV=1	0,12807883	0,523677
Q9H553	Alpha-1,3/1,6-mannosyltransferase ALG2 OS=Homo sapiens OX=9606 GN=ALG2 PE=1 SV=1	0,155076645	0,523599
O15164	Transcription intermediary factor 1-alpha OS=Homo sapiens OX=9606 GN=TRIM24 PE=1 SV=3	-0,111792144	0,523464
Q9BRL6	Serine/arginine-rich splicing factor 8 OS=Homo sapiens OX=9606 GN=SRSF8 PE=1 SV=1	-0,15503712	0,523453
O75027	ATP-binding cassette sub-family B member 7, mitochondrial OS=Homo sapiens OX=9606 GN=ABCB7 PE=1 SV=2	-0,154865091	0,522818
Q5JPH6	Probable glutamate--tRNA ligase, mitochondrial OS=Homo sapiens OX=9606 GN=EARS2 PE=1 SV=2	0,217472279	0,52278
Q8NFQ8	Torsin-1A-interacting protein 2 OS=Homo sapiens OX=9606 GN=TOR1AIP2 PE=1 SV=1	-0,061179841	0,522466
O14653	Golgi SNAP receptor complex member 2 OS=Homo sapiens OX=9606 GN=GOSR2 PE=1 SV=2	-0,127690358	0,52191
Q13443	Disintegrin and metalloproteinase domain-containing protein 9 OS=Homo sapiens OX=9606 GN=ADAM9 PE=1 SV=1	-0,080139839	0,521872
Q00536	Cyclin-dependent kinase 16 OS=Homo sapiens OX=9606 GN=CDK16 PE=1 SV=1	0,282908424	0,521284
P18621	60S ribosomal protein L17 OS=Homo sapiens OX=9606 GN=RPL17 PE=1 SV=3	-0,091775216	0,520586
O15212	Prefoldin subunit 6 OS=Homo sapiens OX=9606 GN=PFDN6 PE=1 SV=1	-0,100019436	0,520164
P53667	LIM domain kinase 1 OS=Homo sapiens OX=9606 GN=LIMK1 PE=1 SV=3	0,245354	0,519123
O75832	26S proteasome non-ATPase regulatory subunit 10 OS=Homo sapiens OX=9606 GN=PSMD10 PE=1 SV=1	-0,084986528	0,518719
Q9BTL3	RNA guanine-N7 methyltransferase activating subunit OS=Homo sapiens OX=9606 GN=RAMAC PE=1 SV=1	-0,153560232	0,518001
Q9H6T3	RNA polymerase II-associated protein 3 OS=Homo sapiens OX=9606 GN=RPAP3 PE=1 SV=2	0,071478944	0,51787
Q5HYI8	Rab-like protein 3 OS=Homo sapiens OX=9606 GN=RABL3 PE=1 SV=1	-0,215427985	0,517716
O43776	Asparagine--tRNA ligase, cytoplasmic OS=Homo sapiens OX=9606 GN=NARS PE=1 SV=1	0,053652059	0,517426
Q9H6V9	Lipid droplet-associated hydrolase OS=Homo sapiens OX=9606 GN=LDAH PE=1 SV=1	-0,153331625	0,517158
P08237	ATP-dependent 6-phosphofructokinase, muscle type OS=Homo sapiens OX=9606 GN=PFKM PE=1 SV=2	0,055133569	0,516991
Q8N7H5	RNA polymerase II-associated factor 1 homolog OS=Homo sapiens OX=9606 GN=PAF1 PE=1 SV=2	0,074993701	0,516187
Q92567	Protein FAM168A OS=Homo sapiens OX=9606 GN=FAM168A PE=1 SV=2	-0,214798136	0,516156
Q9H5N1	Rab GTPase-binding effector protein 2 OS=Homo sapiens OX=9606 GN=RABEP2 PE=1 SV=2	-0,084533333	0,515592
Q14999	Cullin-7 OS=Homo sapiens OX=9606 GN=CUL7 PE=1 SV=2	0,099208277	0,515418
Q9BTC0	Death-inducer obliterator 1 OS=Homo sapiens OX=9606 GN=DIDO1 PE=1 SV=5	0,079258436	0,515374
P30307	M-phase inducer phosphatase 3 OS=Homo sapiens OX=9606 GN=CDC25C PE=1 SV=2	0,60778286	0,515342
P03891	NADH-ubiquinone oxidoreductase chain 2 OS=Homo sapiens OX=9606 GN=MT-ND2 PE=1 SV=2	-0,214436526	0,51526
Q5T2E6	Armadillo-like helical domain-containing protein 3 OS=Homo sapiens OX=9606 GN=ARMH3 PE=1 SV=1	-0,152735586	0,51496
Q5SW79	Centrosomal protein of 170 kDa OS=Homo sapiens OX=9606 GN=CEP170 PE=1 SV=1	-0,035566242	0,51418
P27449	V-type proton ATPase 16 kDa proteolipid subunit OS=Homo sapiens OX=9606 GN=ATP6V0C PE=1 SV=1	-0,213874015	0,513865
P55795	Heterogeneous nuclear ribonucleoprotein H2 OS=Homo sapiens OX=9606 GN=HNRNPH2 PE=1 SV=1	-0,109959759	0,513858
O95747	Serine/threonine-protein kinase OSR1 OS=Homo sapiens OX=9606 GN=OXSR1 PE=1 SV=1	-0,053270845	0,513242
O75506	Heat shock factor-binding protein 1 OS=Homo sapiens OX=9606 GN=HSBP1 PE=1 SV=1	0,125778552	0,513229
Q96EY5	Multivesicular body subunit 12A OS=Homo sapiens OX=9606 GN=MVB12A PE=1 SV=1	-0,213547756	0,513057
O75153	Clustered mitochondria protein homolog OS=Homo sapiens OX=9606 GN=CLUH PE=1 SV=2	-0,050557871	0,512876
Q9UJ68	Mitochondrial peptide methionine sulfoxide reductase OS=Homo sapiens OX=9606 GN=MSRA PE=1 SV=1	-0,125689291	0,512824
P23610	Factor VIII intron 22 protein OS=Homo sapiens OX=9606 GN=F8A1 PE=1 SV=2	-0,215524406	0,512778
Q15800	Methylsterol monooxygenase 1 OS=Homo sapiens OX=9606 GN=MSMO1 PE=1 SV=1	-0,213208666	0,512216
Q9Y547	Intraflagellar transport protein 25 homolog OS=Homo sapiens OX=9606 GN=HSPB11 PE=1 SV=1	-0,125477618	0,511864
Q9H074	Polyadenylate-binding protein-interacting protein 1 OS=Homo sapiens OX=9606 GN=PAIP1 PE=1 SV=1	-0,078764396	0,511739

P48651	Phosphatidylserine synthase 1 OS=Homo sapiens OX=9606 GN=PTDSS1 PE=1 SV=1	0,212822387	0,511259
Q9P2I0	Cleavage and polyadenylation specificity factor subunit 2 OS=Homo sapiens OX=9606 GN=CPSF2 PE=1 SV=2	0,109325157	0,510538
Q9H999	Pantothenate kinase 3 OS=Homo sapiens OX=9606 GN=PANK3 PE=1 SV=1	0,073746742	0,510487
Q9BUR4	Telomerase Cajal body protein 1 OS=Homo sapiens OX=9606 GN=WRAP53 PE=1 SV=1	-0,151475969	0,510319
Q5TA50	Ceramide-1-phosphate transfer protein OS=Homo sapiens OX=9606 GN=CPTP PE=1 SV=1	-0,212204028	0,509726
P04626	Receptor tyrosine-protein kinase erbB-2 OS=Homo sapiens OX=9606 GN=ERBB2 PE=1 SV=1	-0,212158176	0,509612
Q8IYB5	Stromal membrane-associated protein 1 OS=Homo sapiens OX=9606 GN=SMAP1 PE=1 SV=2	-0,212094002	0,509453
P22033	Methylmalonyl-CoA mutase, mitochondrial OS=Homo sapiens OX=9606 GN=MMUT PE=1 SV=4	0,078405851	0,509105
P04075	Fructose-bisphosphate aldolase A OS=Homo sapiens OX=9606 GN=ALDOA PE=1 SV=2	-0,041817512	0,508803
Q9NZ32	Actin-related protein 10 OS=Homo sapiens OX=9606 GN=ACTR10 PE=1 SV=1	-0,064400397	0,508585
Q12986	Transcriptional repressor NF-X1 OS=Homo sapiens OX=9606 GN=NFX1 PE=1 SV=2	0,059058106	0,508288
Q08170	Serine/arginine-rich splicing factor 4 OS=Homo sapiens OX=9606 GN=SRSF4 PE=1 SV=2	0,127940955	0,508213
Q9H098	Protein FAM107B OS=Homo sapiens OX=9606 GN=FAM107B PE=1 SV=1	0,211490584	0,507957
Q8N5I2	Arrestin domain-containing protein 1 OS=Homo sapiens OX=9606 GN=ARRDC1 PE=1 SV=1	0,10653917	0,507376
O14818	Proteasome subunit alpha type-7 OS=Homo sapiens OX=9606 GN=PSMA7 PE=1 SV=1	0,070158494	0,507035
O94927	HAUS augmin-like complex subunit 5 OS=Homo sapiens OX=9606 GN=HAUS5 PE=1 SV=2	-0,150573204	0,506995
Q9BYK8	Helicase with zinc finger domain 2 OS=Homo sapiens OX=9606 GN=HELZ2 PE=1 SV=6	0,063829753	0,506775
Q9Y2D4	Exocyst complex component 6B OS=Homo sapiens OX=9606 GN=EXOC6B PE=1 SV=3	0,089596745	0,506667
Q14161	ARF GTPase-activating protein GIT2 OS=Homo sapiens OX=9606 GN=GIT2 PE=1 SV=2	0,150356334	0,506197
Q04837	Single-stranded DNA-binding protein, mitochondrial OS=Homo sapiens OX=9606 GN=SSBP1 PE=1 SV=1	-0,097622945	0,506166
P48147	Prolyl endopeptidase OS=Homo sapiens OX=9606 GN=PREP PE=1 SV=2	0,055708355	0,505785
Q9BPX7	UPF0415 protein C7orf25 OS=Homo sapiens OX=9606 GN=C7orf25 PE=1 SV=1	-0,150223063	0,505707
Q86UK7	E3 ubiquitin-protein ligase ZNF598 OS=Homo sapiens OX=9606 GN=ZNF598 PE=1 SV=1	0,077902466	0,50541
P21980	Protein-glutamine gamma-glutamyltransferase 2 OS=Homo sapiens OX=9606 GN=TGM2 PE=1 SV=2	0,033450339	0,504859
Q5TC82	Roquin-1 OS=Homo sapiens OX=9606 GN=RC3H1 PE=1 SV=1	-0,093076854	0,50483
Q9H2C0	Gigaxonin OS=Homo sapiens OX=9606 GN=GAN PE=1 SV=1	-0,210029437	0,504333
Q92896	Golgi apparatus protein 1 OS=Homo sapiens OX=9606 GN=GLG1 PE=1 SV=2	-0,032666334	0,504084
Q9Y5Y5	Peroxisomal membrane protein PEX16 OS=Homo sapiens OX=9606 GN=PEX16 PE=1 SV=2	-0,209916236	0,504053
Q9NYY8	FAST kinase domain-containing protein 2, mitochondrial OS=Homo sapiens OX=9606 GN=FASTKD2 PE=1 SV=1	-0,089087133	0,503422
Q96ER9	Coiled-coil domain-containing protein 51 OS=Homo sapiens OX=9606 GN=CCDC51 PE=1 SV=2	0,123527261	0,503034
O95208	Epsin-2 OS=Homo sapiens OX=9606 GN=EPN2 PE=1 SV=3	0,209500947	0,503023
Q08211	ATP-dependent RNA helicase A OS=Homo sapiens OX=9606 GN=DHX9 PE=1 SV=4	-0,030155932	0,502936
Q6P1J9	Parafibromin OS=Homo sapiens OX=9606 GN=CDC73 PE=1 SV=1	-0,069654535	0,502912
Q71DI3	Histone H3.2 OS=Homo sapiens OX=9606 GN=HIST2H3A PE=1 SV=3	0,209346266	0,502639
Q96D53	Atypical kinase COQ8B, mitochondrial OS=Homo sapiens OX=9606 GN=COQ8B PE=1 SV=2	-0,149326617	0,50241
Q96AG4	Leucine-rich repeat-containing protein 59 OS=Homo sapiens OX=9606 GN=LRRC59 PE=1 SV=1	0,063695115	0,502272
Q6ZUT6	Coiled-coil domain-containing protein 9B OS=Homo sapiens OX=9606 GN=CCDC9B PE=1 SV=1	0,123299907	0,502006
P16871	Interleukin-7 receptor subunit alpha OS=Homo sapiens OX=9606 GN=IL7R PE=1 SV=3	-0,209048991	0,501901
O75915	PRA1 family protein 3 OS=Homo sapiens OX=9606 GN=ARL6IP5 PE=1 SV=1	0,096818635	0,501484
Q9NR50	Translation initiation factor eIF-2B subunit gamma OS=Homo sapiens OX=9606 GN=EIF2B3 PE=1 SV=1	0,066316476	0,501211
Q9UPR3	Protein SMG5 OS=Homo sapiens OX=9606 GN=SMG5 PE=1 SV=3	0,208550334	0,500664
Q9BXS6	Nucleolar and spindle-associated protein 1 OS=Homo sapiens OX=9606 GN=NUSAP1 PE=1 SV=1	0,122827199	0,49987
Q969T4	Ubiquitin-conjugating enzyme E2 E3 OS=Homo sapiens OX=9606 GN=UBE2E3 PE=1 SV=1	0,030549631	0,499088
P53985	Monocarboxylate transporter 1 OS=Homo sapiens OX=9606 GN=SLC16A1 PE=1 SV=3	0,106872635	0,497744
Q8IXQ4	GPALPP motifs-containing protein 1 OS=Homo sapiens OX=9606 GN=GPALPP1 PE=1 SV=1	-0,361594373	0,496886
P30626	Sorcin OS=Homo sapiens OX=9606 GN=SRI PE=1 SV=1	0,081789488	0,496737
P55347	Homeobox protein PKNOX1 OS=Homo sapiens OX=9606 GN=PKNOX1 PE=1 SV=3	0,20627357	0,495015
Q96FJ2	Dynein light chain 2, cytoplasmic OS=Homo sapiens OX=9606 GN=DYNLL2 PE=1 SV=1	-0,106277595	0,494648
O75396	Vesicle-trafficking protein SEC22b OS=Homo sapiens OX=9606 GN=SEC22B PE=1 SV=4	0,081469406	0,494547
Q8IWX8	Calcium homeostasis endoplasmic reticulum protein OS=Homo sapiens OX=9606 GN=CHERP PE=1 SV=3	-0,058267848	0,494314
Q9BRX2	Protein pelota homolog OS=Homo sapiens OX=9606 GN=PELO PE=1 SV=2	0,087620516	0,494103
Q53F19	Nuclear cap-binding protein subunit 3 OS=Homo sapiens OX=9606 GN=NCBP3 PE=1 SV=2	-0,146967984	0,493746
P62256	Ubiquitin-conjugating enzyme E2 H OS=Homo sapiens OX=9606 GN=UBE2H PE=1 SV=1	-0,106056096	0,493496
Q9P2B2	Prostaglandin F2 receptor negative regulator OS=Homo sapiens OX=9606 GN=PTGFRN PE=1 SV=2	-0,205548476	0,493215
P45880	Voltage-dependent anion-selective channel protein 2 OS=Homo sapiens OX=9606 GN=VDAC2 PE=1 SV=2	0,065373863	0,493155
Q14554	Protein disulfide-isomerase A5 OS=Homo sapiens OX=9606 GN=PDIA5 PE=1 SV=1	0,382402583	0,492584
Q04656	Copper-transporting ATPase 1 OS=Homo sapiens OX=9606 GN=ATP7A PE=1 SV=4	1,56724764	0,492544
Q9UHY7	Enolase-phosphatase E1 OS=Homo sapiens OX=9606 GN=ENOPH1 PE=1 SV=1	-0,121128973	0,492208
Q9H0U6	39S ribosomal protein L18, mitochondrial OS=Homo sapiens OX=9606 GN=MRPL18 PE=1 SV=1	-0,146538337	0,49217
Q5VUA4	Zinc finger protein 318 OS=Homo sapiens OX=9606 GN=ZNF318 PE=1 SV=2	-0,205121842	0,492156
Q15388	Mitochondrial import receptor subunit TOM20 homolog OS=Homo sapiens OX=9606 GN=TOMM20 PE=1 SV=1	-0,146512545	0,492075
P43353	Aldehyde dehydrogenase family 3 member B1 OS=Homo sapiens OX=9606 GN=ALDH3B1 PE=1 SV=1	0,138125777	0,491681
Q13325	Interferon-induced protein with tetratricopeptide repeats 5 OS=Homo sapiens OX=9606 GN=IFIT5 PE=1 SV=1	-0,065156219	0,491299
Q5VTE6	Protein angel homolog 2 OS=Homo sapiens OX=9606 GN=ANGEL2 PE=2 SV=1	0,20455195	0,490741
P41091	Eukaryotic translation initiation factor 2 subunit 3 OS=Homo sapiens OX=9606 GN=EIF2S3 PE=1 SV=3	0,045336731	0,48988
Q96S55	ATPase WRNIP1 OS=Homo sapiens OX=9606 GN=WRNIP1 PE=1 SV=2	-0,080716644	0,489403
P09234	U1 small nuclear ribonucleoprotein C OS=Homo sapiens OX=9606 GN=SNRPC PE=1 SV=1	0,145760611	0,489318
P60059	Protein transport protein Sec61 subunit gamma OS=Homo sapiens OX=9606 GN=SEC61G PE=1 SV=1	0,203810338	0,4889
P14550	Aldo-keto reductase family 1 member A1 OS=Homo sapiens OX=9606 GN=AKR1A1 PE=1 SV=3	-0,071468323	0,488825
O75884	Putative hydrolase RBBP9 OS=Homo sapiens OX=9606 GN=RBBP9 PE=1 SV=2	-0,120288462	0,488423
Q15054	DNA polymerase delta subunit 3 OS=Homo sapiens OX=9606 GN=POLD3 PE=1 SV=2	-0,145311743	0,487673
Q5VTR2	E3 ubiquitin-protein ligase BRE1A OS=Homo sapiens OX=9606 GN=RNF20 PE=1 SV=2	0,052369041	0,487655
Q9NUQ2	1-acyl-sn-glycerol-3-phosphate acyltransferase epsilon OS=Homo sapiens OX=9606 GN=AGPAT5 PE=1 SV=3	0,191995897	0,487369
Q96BM9	ADP-ribosylation factor-like protein 8A OS=Homo sapiens OX=9606 GN=ARL8A PE=1 SV=1	0,080390603	0,487178
P46100	Transcriptional regulator ATRX OS=Homo sapiens OX=9606 GN=ATRX PE=1 SV=5	-0,080250734	0,486224
Q9UBN7	Histone deacetylase 6 OS=Homo sapiens OX=9606 GN=HDAC6 PE=1 SV=2	-0,144902784	0,486174
Q5T0N5	Formin-binding protein 1-like OS=Homo sapiens OX=9606 GN=FNBP1L PE=1 SV=3	-0,11971682	0,485851
Q13873	Bone morphogenetic protein receptor type-2 OS=Homo sapiens OX=9606 GN=BMPR2 PE=1 SV=2	-0,405836522	0,485683
Q9Y376	Calcium-binding protein 39 OS=Homo sapiens OX=9606 GN=CAB39 PE=1 SV=1	0,067423967	0,484745
Q9H974	Queuine tRNA-ribosyltransferase accessory subunit 2 OS=Homo sapiens OX=9606 GN=QTRT2 PE=1 SV=1	-0,146326363	0,484295
O95573	Long-chain-fatty-acid--CoA ligase 3 OS=Homo sapiens OX=9606 GN=ACSL3 PE=1 SV=3	-0,044854314	0,483975
Q9Y3C1	Nucleolar protein 16 OS=Homo sapiens OX=9606 GN=NOP16 PE=1 SV=2	-0,093776769	0,483848
Q6SZW1	Sterile alpha and TIR motif-containing protein 1 OS=Homo sapiens OX=9606 GN=SARM1 PE=1 SV=1	-0,064247	0,48356
P24385	G1/S-specific cyclin-D1 OS=Homo sapiens OX=9606 GN=CCND1 PE=1 SV=1	0,15824162	0,483271
P51790	H(+)/Cl(-) exchange transporter 3 OS=Homo sapiens OX=9606 GN=CLCN3 PE=1 SV=2	0,150976133	0,483267
Q9BYJ9	YTH domain-containing family protein 1 OS=Homo sapiens OX=9606 GN=YTHDF1 PE=1 SV=1	0,143879089	0,482426
P27635	60S ribosomal protein L10 OS=Homo sapiens OX=9606 GN=RPL10 PE=1 SV=4	-0,064043435	0,48183
Q9NYL2	Mitogen-activated protein kinase kinase kinase 20 OS=Homo sapiens OX=9606 GN=MAP3K20 PE=1 SV=3	0,074657882	0,481725
Q00839	Heterogeneous nuclear ribonucleoprotein U OS=Homo sapiens OX=9606 GN=HNRNPU PE=1 SV=6	-0,03916106	0,481302
Q93084	Sarcoplasmic/endoplasmic reticulum calcium ATPase 3 OS=Homo sapiens OX=9606 GN=ATP2A3 PE=1 SV=2	0,085574467	0,481159
Q9Y478	5'-AMP-activated protein kinase subunit beta-1 OS=Homo sapiens OX=9606 GN=PRKAB1 PE=1 SV=4	-0,200529627	0,480753

Q96CS3	FAS-associated factor 2 OS=Homo sapiens OX=9606 GN=FAF2 PE=1 SV=2	0,061274545	0,480733
Q8IWA5	Choline transporter-like protein 2 OS=Homo sapiens OX=9606 GN=SLC44A2 PE=1 SV=3	-0,103583612	0,480673
Q8NEB9	Phosphatidylinositol 3-kinase catalytic subunit type 3 OS=Homo sapiens OX=9606 GN=PIK3C3 PE=1 SV=1	-0,070358778	0,480276
P28290	Protein ITPRID2 OS=Homo sapiens OX=9606 GN=ITPRID2 PE=1 SV=3	-0,074423675	0,480024
Q92615	La-related protein 4B OS=Homo sapiens OX=9606 GN=LARP4B PE=1 SV=3	-0,079334881	0,479988
P33240	Cleavage stimulation factor subunit 2 OS=Homo sapiens OX=9606 GN=CSTF2 PE=1 SV=1	-0,074374505	0,479667
P48506	Glutamate--cysteine ligase catalytic subunit OS=Homo sapiens OX=9606 GN=GCLC PE=1 SV=2	0,235822115	0,479321
Q9UPP2	#N/D	0,303492072	0,478846
P62312	U6 snRNA-associated Sm-like protein LSm6 OS=Homo sapiens OX=9606 GN=LSM6 PE=1 SV=1	-0,142784679	0,478422
P10915	Hyaluronan and proteoglycan link protein 1 OS=Homo sapiens OX=9606 GN=HAPLN1 PE=2 SV=2	-0,142628954	0,477853
O43516	WAS/WASL-interacting protein family member 1 OS=Homo sapiens OX=9606 GN=WIPF1 PE=1 SV=3	-0,199263917	0,477609
Q12929	Epidermal growth factor receptor kinase substrate 8 OS=Homo sapiens OX=9606 GN=EPS8 PE=1 SV=1	0,199257799	0,477594
Q15286	Ras-related protein Rab-35 OS=Homo sapiens OX=9606 GN=RAB35 PE=1 SV=1	-0,102844066	0,476849
O43164	E3 ubiquitin-protein ligase Praja-2 OS=Homo sapiens OX=9606 GN=PJA2 PE=1 SV=4	-0,198714326	0,476243
O15327	Inositol polyphosphate 4-phosphatase type II OS=Homo sapiens OX=9606 GN=INPP4B PE=1 SV=4	0,10262415	0,475713
Q96JC1	Vam6/Vps39-like protein OS=Homo sapiens OX=9606 GN=VPS39 PE=1 SV=2	0,141943875	0,475349
Q9BU23	Lipase maturation factor 2 OS=Homo sapiens OX=9606 GN=LMF2 PE=1 SV=2	-0,092257965	0,475086
O00442	RNA 3'-terminal phosphate cyclase OS=Homo sapiens OX=9606 GN=RTCA PE=1 SV=1	0,092141064	0,474413
Q8WUW1	Protein BRICK1 OS=Homo sapiens OX=9606 GN=BRK1 PE=1 SV=1	-0,092086913	0,474101
O14924	Regulator of G-protein signaling 12 OS=Homo sapiens OX=9606 GN=RGS12 PE=1 SV=1	0,302969244	0,473653
P98082	Disabled homolog 2 OS=Homo sapiens OX=9606 GN=DAB2 PE=1 SV=3	-0,066037007	0,473517
Q8NCH0	Carbohydrate sulfotransferase 14 OS=Homo sapiens OX=9606 GN=CHST14 PE=1 SV=2	-0,102188851	0,473465
P04150	Glucocorticoid receptor OS=Homo sapiens OX=9606 GN=NR3C1 PE=1 SV=1	-0,116859553	0,473026
P30049	ATP synthase subunit delta, mitochondrial OS=Homo sapiens OX=9606 GN=ATP5F1D PE=1 SV=2	0,197235064	0,472568
Q99871	HAUS augmin-like complex subunit 7 OS=Homo sapiens OX=9606 GN=HAUS7 PE=1 SV=3	0,141164901	0,472503
Q9NX58	Cell growth-regulating nucleolar protein OS=Homo sapiens OX=9606 GN=LYAR PE=1 SV=2	0,084175591	0,472346
Q9GZZ9	Ubiquitin-like modifier-activating enzyme 5 OS=Homo sapiens OX=9606 GN=UBA5 PE=1 SV=1	-0,078208287	0,472337
Q9BU89	Deoxyhypusine hydroxylase OS=Homo sapiens OX=9606 GN=DOHH PE=1 SV=1	0,073298506	0,471869
P62273	40S ribosomal protein S29 OS=Homo sapiens OX=9606 GN=RPS29 PE=1 SV=2	0,196686963	0,471206
Q6UWP2	Dehydrogenase/reductase SDR family member 11 OS=Homo sapiens OX=9606 GN=DHRS11 PE=1 SV=1	-0,140776028	0,471084
Q8WVB6	Chromosome transmission fidelity protein 18 homolog OS=Homo sapiens OX=9606 GN=CHTF18 PE=1 SV=1	-0,196537202	0,470834
Q9Y2U8	Inner nuclear membrane protein Man1 OS=Homo sapiens OX=9606 GN=LEMD3 PE=1 SV=2	-0,446392928	0,470741
Q13136	Liprin-alpha-1 OS=Homo sapiens OX=9606 GN=PPFIA1 PE=1 SV=1	0,083908329	0,470666
Q9BUH6	Protein PAXX OS=Homo sapiens OX=9606 GN=PAXX PE=1 SV=2	-0,196219413	0,470044
Q9BQ95	Evolutionarily conserved signaling intermediate in Toll pathway, mitochondrial OS=Homo sapiens OX=9606 GN=ECSIT PE=	0,196202248	0,470001
Q13488	V-type proton ATPase 116 kDa subunit a isoform 3 OS=Homo sapiens OX=9606 GN=TCIRG1 PE=1 SV=3	0,139765493	0,467397
Q9H871	E3 ubiquitin-protein transferase RMND5A OS=Homo sapiens OX=9606 GN=RMND5A PE=1 SV=1	-0,195100152	0,467262
P15104	Glutamine synthetase OS=Homo sapiens OX=9606 GN=GLUL PE=1 SV=4	-0,195091172	0,46724
Q9UK45	U6 snRNA-associated Sm-like protein LSm7 OS=Homo sapiens OX=9606 GN=LSM7 PE=1 SV=1	-0,1007267	0,465928
Q6ZRQ5	Protein MMS22-like OS=Homo sapiens OX=9606 GN=MMS22L PE=1 SV=3	0,194384975	0,465485
P30038	Delta-1-pyrroline-5-carboxylate dehydrogenase, mitochondrial OS=Homo sapiens OX=9606 GN=ALDH4A1 PE=1 SV=3	0,208207795	0,465365
P61803	Dolichyl-diphosphooligosaccharide--protein glycosyltransferase subunit DAD1 OS=Homo sapiens OX=9606 GN=DAD1 PE=	0,139086063	0,46492
Q9UMS4	Pre-mRNA-processing factor 19 OS=Homo sapiens OX=9606 GN=PRPF19 PE=1 SV=1	0,059458246	0,464697
Q15459	Splicing factor 3A subunit 1 OS=Homo sapiens OX=9606 GN=SF3A1 PE=1 SV=1	-0,043269629	0,464685
P60174	Triosephosphate isomerase OS=Homo sapiens OX=9606 GN=TPI1 PE=1 SV=3	0,051600734	0,463795
P20810	Calpastatin OS=Homo sapiens OX=9606 GN=CAST PE=1 SV=4	-0,048596871	0,462539
Q8IU81	Interferon regulatory factor 2-binding protein 1 OS=Homo sapiens OX=9606 GN=IRF2BP1 PE=1 SV=1	-0,192822217	0,4616
P08670	Vimentin OS=Homo sapiens OX=9606 GN=VIM PE=1 SV=4	-0,025932176	0,461568
Q9NYP7	Elongation of very long chain fatty acids protein 5 OS=Homo sapiens OX=9606 GN=ELOVL5 PE=1 SV=1	-0,192566456	0,460964
P61956	Small ubiquitin-related modifier 2 OS=Homo sapiens OX=9606 GN=SUMO2 PE=1 SV=3	-0,192018006	0,459601
P14859	POU domain, class 2, transcription factor 1 OS=Homo sapiens OX=9606 GN=POU2F1 PE=1 SV=2	-0,137345229	0,45858
Q8NBQ5	Estradiol 17-beta-dehydrogenase 11 OS=Homo sapiens OX=9606 GN=HSD17B11 PE=1 SV=3	-0,067490426	0,458314
Q6FI81	Anamorsin OS=Homo sapiens OX=9606 GN=CIAPIN1 PE=1 SV=2	0,0586473	0,457573
Q06546	GA-binding protein alpha chain OS=Homo sapiens OX=9606 GN=GABPA PE=1 SV=1	-0,191036556	0,457161
Q9P0L0	Vesicle-associated membrane protein-associated protein A OS=Homo sapiens OX=9606 GN=VAPA PE=1 SV=3	-0,058519721	0,456454
P41252	Isoleucine--tRNA ligase, cytoplasmic OS=Homo sapiens OX=9606 GN=IARS PE=1 SV=2	-0,030461503	0,454461
P07199	Major centromere autoantigen B OS=Homo sapiens OX=9606 GN=CENPB PE=1 SV=2	-0,034244173	0,454306
P62166	Neuronal calcium sensor 1 OS=Homo sapiens OX=9606 GN=NCS1 PE=1 SV=2	-0,189863026	0,454243
Q9NPJ8	NTF2-related export protein 2 OS=Homo sapiens OX=9606 GN=NXT2 PE=1 SV=1	0,136110774	0,45409
Q9NWT6	Hypoxia-inducible factor 1-alpha inhibitor OS=Homo sapiens OX=9606 GN=HIF1AN PE=1 SV=2	0,081175512	0,453547
Q92793	CREB-binding protein OS=Homo sapiens OX=9606 GN=CREBBP PE=1 SV=3	-0,375772728	0,453033
Q8N8N7	Prostaglandin reductase 2 OS=Homo sapiens OX=9606 GN=PTGR2 PE=1 SV=1	0,14623152	0,452453
Q92890	Ubiquitin recognition factor in ER-associated degradation protein 1 OS=Homo sapiens OX=9606 GN=UFD1 PE=1 SV=3	-0,06671543	0,452414
P49848	Transcription initiation factor TFIID subunit 6 OS=Homo sapiens OX=9606 GN=TAF6 PE=1 SV=1	-0,189067834	0,452266
O95067	G2/mitotic-specific cyclin-B2 OS=Homo sapiens OX=9606 GN=CCNB2 PE=1 SV=1	-0,189066576	0,452263
P61421	V-type proton ATPase subunit d 1 OS=Homo sapiens OX=9606 GN=ATP6V0D1 PE=1 SV=1	-0,088232526	0,452001
Q969P0	Immunoglobulin superfamily member 8 OS=Homo sapiens OX=9606 GN=IGSF8 PE=1 SV=1	-0,188748119	0,451471
Q9NWU2	Glucose-induced degradation protein 8 homolog OS=Homo sapiens OX=9606 GN=GID8 PE=1 SV=1	0,11197309	0,451215
Q8NI36	WD repeat-containing protein 36 OS=Homo sapiens OX=9606 GN=WDR36 PE=1 SV=1	0,066535542	0,451047
Q9NQS1	Cell death regulator Aven OS=Homo sapiens OX=9606 GN=AVEN PE=1 SV=1	-0,13505932	0,45027
P42285	Exosome RNA helicase MTR4 OS=Homo sapiens OX=9606 GN=MTREX PE=1 SV=3	0,038818929	0,449407
Q6IN85	Serine/threonine-protein phosphatase 4 regulatory subunit 3A OS=Homo sapiens OX=9606 GN=PPP4R3A PE=1 SV=1	-0,087757029	0,449288
P10515	Dihydrolipoyllysine-residue acetyltransferase component of pyruvate dehydrogenase complex, mitochondrial OS=Homo s	0,070035235	0,448369
P01130	Low-density lipoprotein receptor OS=Homo sapiens OX=9606 GN=LDLR PE=1 SV=1	0,055356469	0,447963
Q9NSK0	Kinesin light chain 4 OS=Homo sapiens OX=9606 GN=KLC4 PE=1 SV=3	-0,069963825	0,447858
Q15032	R3H domain-containing protein 1 OS=Homo sapiens OX=9606 GN=R3HDM1 PE=1 SV=3	0,012318229	0,447577
Q8N5N7	39S ribosomal protein L50, mitochondrial OS=Homo sapiens OX=9606 GN=MRPL50 PE=1 SV=2	0,110718169	0,445638
Q86VH2	Kinesin-like protein KIF27 OS=Homo sapiens OX=9606 GN=KIF27 PE=2 SV=1	0,186391233	0,44561
O14874	[3-methyl-2-oxobutanoate dehydrogenase [lipoamide]] kinase, mitochondrial OS=Homo sapiens OX=9606 GN=BCKDK PE=	-0,249223995	0,445332
P14649	Myosin light chain 6B OS=Homo sapiens OX=9606 GN=MYL6B PE=1 SV=1	0,133531081	0,444724
Q14697	Neutral alpha-glucosidase AB OS=Homo sapiens OX=9606 GN=GANAB PE=1 SV=3	0,029821754	0,443722
P53365	Arfaptin-2 OS=Homo sapiens OX=9606 GN=ARFIP2 PE=1 SV=1	-0,096353939	0,443511
P36578	60S ribosomal protein L4 OS=Homo sapiens OX=9606 GN=RPL4 PE=1 SV=5	-0,038334415	0,443101
Q9BWH2	FUN14 domain-containing protein 2 OS=Homo sapiens OX=9606 GN=FUNDC2 PE=1 SV=2	0,418009294	0,442263
P46736	Lys-63-specific deubiquitinase BRCC36 OS=Homo sapiens OX=9606 GN=BRCC3 PE=1 SV=2	0,109777085	0,441463
P35251	Replication factor C subunit 1 OS=Homo sapiens OX=9606 GN=RFC1 PE=1 SV=4	0,06200566	0,44118
Q8IYS1	Peptidase M20 domain-containing protein 2 OS=Homo sapiens OX=9606 GN=PM20D2 PE=1 SV=2	-0,109597651	0,440668
Q14644	Ras GTPase-activating protein 3 OS=Homo sapiens OX=9606 GN=RASA3 PE=1 SV=3	-0,184203183	0,440168
Q9NQP4	Prefoldin subunit 4 OS=Homo sapiens OX=9606 GN=PFDN4 PE=1 SV=1	0,183758216	0,439061
Q96N67	Dedicator of cytokinesis protein 7 OS=Homo sapiens OX=9606 GN=DOCK7 PE=1 SV=4	0,034963924	0,439003
Q9NRY4	Rho GTPase-activating protein 35 OS=Homo sapiens OX=9606 GN=ARHGAP35 PE=1 SV=3	0,183516544	0,43846

Q93062	RNA-binding protein with multiple splicing OS=Homo sapiens OX=9606 GN=RBPMS PE=1 SV=1	0,140012118	0,437431
Q53H82	Endoribonuclease LACTB2 OS=Homo sapiens OX=9606 GN=LACTB2 PE=1 SV=2	-0,108716398	0,436765
P30825	High affinity cationic amino acid transporter 1 OS=Homo sapiens OX=9606 GN=SLC7A1 PE=1 SV=1	-0,094931158	0,436256
Q8TC44	POC1 centriolar protein homolog B OS=Homo sapiens OX=9606 GN=POC1B PE=1 SV=1	0,250892278	0,435277
Q9UK61	Protein TASOR OS=Homo sapiens OX=9606 GN=TASOR PE=1 SV=3	0,094601619	0,434579
O75382	Tripartite motif-containing protein 3 OS=Homo sapiens OX=9606 GN=TRIM3 PE=1 SV=2	0,094568923	0,434412
Q5ZPR3	CD276 antigen OS=Homo sapiens OX=9606 GN=CD276 PE=1 SV=1	0,072555105	0,4343
Q9BZD4	Kinetochore protein Nuf2 OS=Homo sapiens OX=9606 GN=NUF2 PE=1 SV=2	0,061033572	0,433449
Q9NWS0	PIH1 domain-containing protein 1 OS=Homo sapiens OX=9606 GN=PIH1D1 PE=1 SV=1	0,094343186	0,433264
P45983	Mitogen-activated protein kinase 8 OS=Homo sapiens OX=9606 GN=MAPK8 PE=1 SV=2	-0,181412967	0,433228
P62826	GTP-binding nuclear protein Ran OS=Homo sapiens OX=9606 GN=RAN PE=1 SV=3	-0,072383889	0,433157
P54920	Alpha-soluble NSF attachment protein OS=Homo sapiens OX=9606 GN=NAPA PE=1 SV=3	-0,051807582	0,433035
O60256	Phosphoribosyl pyrophosphate synthase-associated protein 2 OS=Homo sapiens OX=9606 GN=PRPSAP2 PE=1 SV=1	-0,072333914	0,432824
P62241	40S ribosomal protein S8 OS=Homo sapiens OX=9606 GN=RPS8 PE=1 SV=2	-0,05172333	0,432247
Q86UD0	Suppressor APC domain-containing protein 2 OS=Homo sapiens OX=9606 GN=SAPCD2 PE=1 SV=2	0,180978596	0,432148
Q9Y3U8	60S ribosomal protein L36 OS=Homo sapiens OX=9606 GN=RPL36 PE=1 SV=3	-0,094100219	0,432028
Q92791	Endoplasmic reticulum protein SC65 OS=Homo sapiens OX=9606 GN=P3H4 PE=1 SV=1	0,180868449	0,431874
Q13283	Ras GTPase-activating protein-binding protein 1 OS=Homo sapiens OX=9606 GN=G3BP1 PE=1 SV=1	0,041434753	0,43184
P19838	Nuclear factor NF-kappa-B p105 subunit OS=Homo sapiens OX=9606 GN=NFKB1 PE=1 SV=2	-0,077603673	0,431349
O94913	Pre-mRNA cleavage complex 2 protein Pcf11 OS=Homo sapiens OX=9606 GN=PCF11 PE=1 SV=3	-0,180116577	0,430004
Q96SK2	Transmembrane protein 209 OS=Homo sapiens OX=9606 GN=TMEM209 PE=1 SV=2	0,041325915	0,42996
Q9NYU1	UDP-glucose:glycoprotein glucosyltransferase 2 OS=Homo sapiens OX=9606 GN=UGGT2 PE=1 SV=4	-0,093573096	0,42935
Q9P2R3	Rabankyrin-5 OS=Homo sapiens OX=9606 GN=ANKFY1 PE=1 SV=2	0,044288312	0,429294
Q8IUD2	ELKS/Rab6-interacting/CAST family member 1 OS=Homo sapiens OX=9606 GN=ERC1 PE=1 SV=1	-0,084151265	0,428805
P33552	Cyclin-dependent kinases regulatory subunit 2 OS=Homo sapiens OX=9606 GN=CKS2 PE=1 SV=1	0,10681235	0,428349
Q96K37	Solute carrier family 35 member E1 OS=Homo sapiens OX=9606 GN=SLC35E1 PE=1 SV=2	0,12900518	0,428346
P09132	Signal recognition particle 19 kDa protein OS=Homo sapiens OX=9606 GN=SRP19 PE=1 SV=3	0,106800995	0,428299
P15927	Replication protein A 32 kDa subunit OS=Homo sapiens OX=9606 GN=RPA2 PE=1 SV=1	-0,084030077	0,428119
O15116	U6 snRNA-associated Sm-like protein LSm1 OS=Homo sapiens OX=9606 GN=LSM1 PE=1 SV=1	0,093157843	0,427242
Q9NWU5	39S ribosomal protein L22, mitochondrial OS=Homo sapiens OX=9606 GN=MRPL22 PE=1 SV=1	0,106530271	0,427104
Q86UP2	Kinectin OS=Homo sapiens OX=9606 GN=KTN1 PE=1 SV=1	0,04295827	0,426722
Q8WTW3	Conserved oligomeric Golgi complex subunit 1 OS=Homo sapiens OX=9606 GN=COG1 PE=1 SV=1	-0,106423296	0,426632
P25787	Proteasome subunit alpha type-2 OS=Homo sapiens OX=9606 GN=PSMA2 PE=1 SV=2	0,071339356	0,426197
Q9BSK0	MARVEL domain-containing protein 1 OS=Homo sapiens OX=9606 GN=MARVELD1 PE=1 SV=1	-0,17842929	0,425807
Q9BRX5	DNA replication complex GINS protein PSF3 OS=Homo sapiens OX=9606 GN=GINS3 PE=1 SV=1	-0,178411063	0,425762
O14929	Histone acetyltransferase type B catalytic subunit OS=Homo sapiens OX=9606 GN=HAT1 PE=1 SV=1	0,060028467	0,425482
P54277	PMS1 protein homolog 1 OS=Homo sapiens OX=9606 GN=PMS1 PE=1 SV=1	-0,155615713	0,425336
Q9UJ41	Rab5 GDP/GTP exchange factor OS=Homo sapiens OX=9606 GN=RABGEF1 PE=1 SV=3	-0,092762446	0,425236
P61019	Ras-related protein Rab-2A OS=Homo sapiens OX=9606 GN=RAB2A PE=1 SV=1	0,059898681	0,424455
Q96GX2	Ataxin-7-like protein 3B OS=Homo sapiens OX=9606 GN=ATXN7L3B PE=1 SV=2	0,177531596	0,423575
Q92574	Hamartin OS=Homo sapiens OX=9606 GN=TSC1 PE=1 SV=2	-0,127594044	0,423254
Q9BV68	E3 ubiquitin-protein ligase RNF126 OS=Homo sapiens OX=9606 GN=RNF126 PE=1 SV=2	-0,177300915	0,423001
Q9UMR2	#N/D	0,062738155	0,422367
Q9H3K2	Growth hormone-inducible transmembrane protein OS=Homo sapiens OX=9606 GN=GHITM PE=1 SV=2	-0,105294256	0,421656
Q07000	HLA class I histocompatibility antigen, Cw-15 alpha chain OS=Homo sapiens OX=9606 GN=HLA-C PE=1 SV=1	0,127012425	0,421158
P52735	Guanine nucleotide exchange factor VAV2 OS=Homo sapiens OX=9606 GN=VAV2 PE=1 SV=2	0,062486435	0,420478
Q9UBQ5	Eukaryotic translation initiation factor 3 subunit K OS=Homo sapiens OX=9606 GN=EIF3K PE=1 SV=1	-0,070430056	0,420155
O75607	Nucleoplasmin-3 OS=Homo sapiens OX=9606 GN=NPM3 PE=1 SV=3	0,126657433	0,419878
O00217	NADH dehydrogenase [ubiquinone] iron-sulfur protein 8, mitochondrial OS=Homo sapiens OX=9606 GN=NDUFS8 PE=1 SV	0,091643825	0,41957
Q96EI5	Transcription elongation factor A protein-like 4 OS=Homo sapiens OX=9606 GN=TCEAL4 PE=1 SV=2	-0,175656615	0,418912
O15173	Membrane-associated progesterone receptor component 2 OS=Homo sapiens OX=9606 GN=PGRMC2 PE=1 SV=1	0,075565101	0,418768
Q92624	Amyloid protein-binding protein 2 OS=Homo sapiens OX=9606 GN=APPBP2 PE=1 SV=2	-0,175548394	0,418642
P62330	ADP-ribosylation factor 6 OS=Homo sapiens OX=9606 GN=ARF6 PE=1 SV=2	-0,091457796	0,418628
P62195	26S proteasome regulatory subunit 8 OS=Homo sapiens OX=9606 GN=PSMC5 PE=1 SV=1	-0,041219574	0,418496
Q8N5M9	Protein jagunal homolog 1 OS=Homo sapiens OX=9606 GN=JAGN1 PE=1 SV=1	0,17538735	0,418242
O43715	TP53-regulated inhibitor of apoptosis 1 OS=Homo sapiens OX=9606 GN=TRIAP1 PE=1 SV=1	-0,175204122	0,417786
Q9BVG4	Protein PBDC1 OS=Homo sapiens OX=9606 GN=PBDC1 PE=1 SV=1	0,075342477	0,417399
P50991	T-complex protein 1 subunit delta OS=Homo sapiens OX=9606 GN=CCT4 PE=1 SV=4	0,031092302	0,417099
P31751	RAC-beta serine/threonine-protein kinase OS=Homo sapiens OX=9606 GN=AKT2 PE=1 SV=2	-0,091135437	0,416998
Q9Y285	Phenylalanine--tRNA ligase alpha subunit OS=Homo sapiens OX=9606 GN=FARSA PE=1 SV=3	-0,045458821	0,415914
Q9NS86	LanC-like protein 2 OS=Homo sapiens OX=9606 GN=LANCL2 PE=1 SV=1	-0,081836252	0,415741
Q8N1W1	Rho guanine nucleotide exchange factor 28 OS=Homo sapiens OX=9606 GN=ARHGEF28 PE=1 SV=3	0,1743471	0,415655
O95183	Vesicle-associated membrane protein 5 OS=Homo sapiens OX=9606 GN=VAMP5 PE=1 SV=1	-0,103929897	0,415654
P49459	Ubiquitin-conjugating enzyme E2 A OS=Homo sapiens OX=9606 GN=UBE2A PE=1 SV=2	0,174145583	0,415154
Q6ZUJ8	Phosphoinositide 3-kinase adapter protein 1 OS=Homo sapiens OX=9606 GN=PIK3AP1 PE=1 SV=2	0,040802575	0,415027
P31150	Rab GDP dissociation inhibitor alpha OS=Homo sapiens OX=9606 GN=GDI1 PE=1 SV=2	0,034849637	0,414231
Q99594	Transcriptional enhancer factor TEF-5 OS=Homo sapiens OX=9606 GN=TEAD3 PE=1 SV=2	-0,118657051	0,414077
Q9Y3B2	Exosome complex component CSL4 OS=Homo sapiens OX=9606 GN=EXOSC1 PE=1 SV=1	-0,103418391	0,413408
Q9UNK0	Syntaxin-8 OS=Homo sapiens OX=9606 GN=STX8 PE=1 SV=2	-0,173181507	0,412756
Q9NX24	H/ACA ribonucleoprotein complex subunit 2 OS=Homo sapiens OX=9606 GN=NHP2 PE=1 SV=1	-0,103016649	0,411644
Q92609	TBC1 domain family member 5 OS=Homo sapiens OX=9606 GN=TBC1D5 PE=1 SV=1	0,049504619	0,411582
Q9NW68	BSD domain-containing protein 1 OS=Homo sapiens OX=9606 GN=BSDC1 PE=1 SV=1	0,172686215	0,411525
Q96EQ0	Small glutamine-rich tetratricopeptide repeat-containing protein beta OS=Homo sapiens OX=9606 GN=SGTB PE=1 SV=1	0,102972397	0,41145
P50579	Methionine aminopeptidase 2 OS=Homo sapiens OX=9606 GN=METAP2 PE=1 SV=1	0,04631374	0,410856
P21399	Cytoplasmic aconitate hydratase OS=Homo sapiens OX=9606 GN=ACO1 PE=1 SV=3	0,041444221	0,409945
Q92643	GPI-anchor transamidase OS=Homo sapiens OX=9606 GN=PIGK PE=1 SV=2	0,102367507	0,408797
P39687	Acidic leucine-rich nuclear phosphoprotein 32 family member A OS=Homo sapiens OX=9606 GN=ANP32A PE=1 SV=1	0,089463718	0,408562
P08133	Annexin A6 OS=Homo sapiens OX=9606 GN=ANXA6 PE=1 SV=3	-0,027077562	0,407938
P36543	V-type proton ATPase subunit E 1 OS=Homo sapiens OX=9606 GN=ATP6V1E1 PE=1 SV=1	0,064206237	0,406961
Q96DM3	Regulator of MON1-CCZ1 complex OS=Homo sapiens OX=9606 GN=RMC1 PE=1 SV=2	0,170813326	0,406867
Q15904	V-type proton ATPase subunit S1 OS=Homo sapiens OX=9606 GN=ATP6AP1 PE=1 SV=2	0,073575632	0,406554
Q9NXF1	Testis-expressed protein 10 OS=Homo sapiens OX=9606 GN=TEX10 PE=1 SV=2	-0,047316858	0,406287
Q96NT0	Coiled-coil domain-containing protein 115 OS=Homo sapiens OX=9606 GN=CCDC115 PE=1 SV=1	-0,028114535	0,405076
O14965	Aurora kinase A OS=Homo sapiens OX=9606 GN=AURKA PE=1 SV=2	0,170043928	0,404954
Q9H0W9	Ester hydrolase C11orf54 OS=Homo sapiens OX=9606 GN=C11orf54 PE=1 SV=1	0,122426502	0,404669
P49354	Protein farnesyltransferase/geranylgeranyltransferase type-1 subunit alpha OS=Homo sapiens OX=9606 GN=FNTA PE=1 S	0,06803708	0,404327
Q15006	ER membrane protein complex subunit 2 OS=Homo sapiens OX=9606 GN=EMC2 PE=1 SV=1	0,169425002	0,403416
P23458	Tyrosine-protein kinase JAK1 OS=Homo sapiens OX=9606 GN=JAK1 PE=1 SV=2	-0,073030001	0,403215
Q8N3E9	1-phosphatidylinositol 4,5-bisphosphate phosphodiesterase delta-3 OS=Homo sapiens OX=9606 GN=PLCD3 PE=1 SV=3	-0,060134649	0,402907
Q53LP3	Ankyrin repeat domain-containing protein SOWAHC OS=Homo sapiens OX=9606 GN=SOWAHC PE=1 SV=1	0,06546536	0,402707
Q9Y6N5	Sulfide:quinone oxidoreductase, mitochondrial OS=Homo sapiens OX=9606 GN=SQOR PE=1 SV=1	-0,121767483	0,402305

Q9H2G2	STE20-like serine/threonine-protein kinase OS=Homo sapiens OX=9606 GN=SLK PE=1 SV=1	-0,033929017	0,402033
P57721	Poly(rC)-binding protein 3 OS=Homo sapiens OX=9606 GN=PCBP3 PE=2 SV=2	0,121421008	0,401063
P84103	Serine/arginine-rich splicing factor 3 OS=Homo sapiens OX=9606 GN=SRSF3 PE=1 SV=1	0,072444262	0,399636
Q96Q11	CCA tRNA nucleotidyltransferase 1, mitochondrial OS=Homo sapiens OX=9606 GN=TRNT1 PE=1 SV=2	0,078840736	0,398939
Q96JD6	1,5-anhydro-D-fructose reductase OS=Homo sapiens OX=9606 GN=AKR1E2 PE=1 SV=2	0,167521508	0,398684
Q9NRM7	Serine/threonine-protein kinase LATS2 OS=Homo sapiens OX=9606 GN=LATS2 PE=1 SV=2	-0,124222499	0,398394
O94842	TOX high mobility group box family member 4 OS=Homo sapiens OX=9606 GN=TOX4 PE=1 SV=1	0,052542609	0,398186
P51828	Adenylate cyclase type 7 OS=Homo sapiens OX=9606 GN=ADCY7 PE=1 SV=1	0,298962158	0,397866
Q96JG6	Syndetin OS=Homo sapiens OX=9606 GN=VPS50 PE=1 SV=3	-0,07861393	0,397671
O43502	DNA repair protein RAD51 homolog 3 OS=Homo sapiens OX=9606 GN=RAD51C PE=1 SV=1	-0,166933059	0,397221
Q8N6M0	Deubiquitinase OTUD6B OS=Homo sapiens OX=9606 GN=OTUD6B PE=1 SV=1	-0,166697787	0,396636
Q9BPW8	Protein NipSnap homolog 1 OS=Homo sapiens OX=9606 GN=NIPSNAP1 PE=1 SV=1	-0,059285466	0,396596
P12955	Xaa-Pro dipeptidase OS=Homo sapiens OX=9606 GN=PEPD PE=1 SV=3	0,049571302	0,396005
Q92614	Unconventional myosin-XVIIIa OS=Homo sapiens OX=9606 GN=MYO18A PE=1 SV=3	0,035949381	0,395273
Q99714	3-hydroxyacyl-CoA dehydrogenase type-2 OS=Homo sapiens OX=9606 GN=HSD17B10 PE=1 SV=3	-0,049378769	0,394296
O95721	Synaptosomal-associated protein 29 OS=Homo sapiens OX=9606 GN=SNAP29 PE=1 SV=1	-0,077969458	0,394073
Q9NZM3	Intersectin-2 OS=Homo sapiens OX=9606 GN=ITSN2 PE=1 SV=3	-0,098891282	0,3936
P31930	Cytochrome b-c1 complex subunit 1, mitochondrial OS=Homo sapiens OX=9606 GN=UQCRC1 PE=1 SV=3	-0,047551523	0,39355
Q71SY5	Mediator of RNA polymerase II transcription subunit 25 OS=Homo sapiens OX=9606 GN=MED25 PE=1 SV=2	0,119201208	0,393118
Q8TAA9	Vang-like protein 1 OS=Homo sapiens OX=9606 GN=VANGL1 PE=1 SV=1	-0,21366365	0,393038
Q16181	Septin-7 OS=Homo sapiens OX=9606 GN=SEPTIN7 PE=1 SV=2	-0,041992099	0,392767
Q6GMV2	SET and MYND domain-containing protein 5 OS=Homo sapiens OX=9606 GN=SMYD5 PE=1 SV=2	-0,119009667	0,392433
Q5SW96	Low density lipoprotein receptor adapter protein 1 OS=Homo sapiens OX=9606 GN=LDLRAP1 PE=1 SV=3	0,164837369	0,392012
Q6FI13	Histone H2A type 2-A OS=Homo sapiens OX=9606 GN=HIST2H2AA3 PE=1 SV=3	0,118885957	0,391991
O94864	STAGA complex 65 subunit gamma OS=Homo sapiens OX=9606 GN=SUPT7L PE=1 SV=1	-0,221708461	0,391849
P62136	Serine/threonine-protein phosphatase PP1-alpha catalytic subunit OS=Homo sapiens OX=9606 GN=PPP1CA PE=1 SV=1	-0,043031145	0,391249
P21953	2-oxoisovalerate dehydrogenase subunit beta, mitochondrial OS=Homo sapiens OX=9606 GN=BCKDHB PE=1 SV=2	0,164407908	0,390945
Q9Y3F4	Serine-threonine kinase receptor-associated protein OS=Homo sapiens OX=9606 GN=STRAP PE=1 SV=1	-0,047253661	0,390813
Q9P2J3	Kelch-like protein 9 OS=Homo sapiens OX=9606 GN=KLHL9 PE=1 SV=2	-0,16435328	0,390809
Q8WVM8	Sec1 family domain-containing protein 1 OS=Homo sapiens OX=9606 GN=SCFD1 PE=1 SV=4	0,061806466	0,390127
Q9BPU6	Dihydropyrimidinase-related protein 5 OS=Homo sapiens OX=9606 GN=DPYSL5 PE=1 SV=1	-0,058404145	0,390064
Q13310	Polyadenylate-binding protein 4 OS=Homo sapiens OX=9606 GN=PABPC4 PE=1 SV=1	-0,039618664	0,389888
Q9BRJ7	Tudor-interacting repair regulator protein OS=Homo sapiens OX=9606 GN=NUDT16L1 PE=1 SV=1	-0,097674159	0,388298
P45985	Dual specificity mitogen-activated protein kinase kinase 4 OS=Homo sapiens OX=9606 GN=MAP2K4 PE=1 SV=1	-0,076801894	0,387569
P38117	Electron transfer flavoprotein subunit beta OS=Homo sapiens OX=9606 GN=ETFB PE=1 SV=3	0,055166438	0,387333
Q8TE02	Elongator complex protein 5 OS=Homo sapiens OX=9606 GN=ELP5 PE=1 SV=2	-0,162906701	0,387215
Q9UI12	V-type proton ATPase subunit H OS=Homo sapiens OX=9606 GN=ATP6V1H PE=1 SV=1	-0,046861353	0,387213
P29992	Guanine nucleotide-binding protein subunit alpha-11 OS=Homo sapiens OX=9606 GN=GNA11 PE=1 SV=2	-0,055047063	0,386405
Q8IYB8	ATP-dependent RNA helicase SUPV3L1, mitochondrial OS=Homo sapiens OX=9606 GN=SUPV3L1 PE=1 SV=1	-0,162406756	0,385972
O15372	Eukaryotic translation initiation factor 3 subunit H OS=Homo sapiens OX=9606 GN=EIF3H PE=1 SV=1	-0,048365836	0,38533
Q92626	Peroxidasin homolog OS=Homo sapiens OX=9606 GN=PXDN PE=1 SV=2	-0,076369657	0,385165
Q16763	Ubiquitin-conjugating enzyme E2 S OS=Homo sapiens OX=9606 GN=UBE2S PE=1 SV=2	0,076365714	0,385143
O95273	Cyclin-D1-binding protein 1 OS=Homo sapiens OX=9606 GN=CCNDBP1 PE=1 SV=2	0,116927929	0,385
O75436	Vacuolar protein sorting-associated protein 26A OS=Homo sapiens OX=9606 GN=VPS26A PE=1 SV=2	0,057716794	0,384984
Q9BWD1	Acetyl-CoA acetyltransferase, cytosolic OS=Homo sapiens OX=9606 GN=ACAT2 PE=1 SV=2	-0,052355921	0,384638
Q96RQ1	Endoplasmic reticulum-Golgi intermediate compartment protein 2 OS=Homo sapiens OX=9606 GN=ERGIC2 PE=1 SV=2	-0,16173378	0,3843
Q9ULF5	Zinc transporter ZIP10 OS=Homo sapiens OX=9606 GN=SLC39A10 PE=1 SV=2	-0,161560227	0,383869
O14907	Tax1-binding protein 3 OS=Homo sapiens OX=9606 GN=TAX1BP3 PE=1 SV=2	-0,116596026	0,383816
O15061	Synemin OS=Homo sapiens OX=9606 GN=SYNM PE=1 SV=2	-0,161408263	0,383492
Q7Z6B7	SLIT-ROBO Rho GTPase-activating protein 1 OS=Homo sapiens OX=9606 GN=SRGAP1 PE=1 SV=1	0,161398753	0,383468
A6ND36	Protein FAM83G OS=Homo sapiens OX=9606 GN=FAM83G PE=1 SV=2	-0,027842108	0,383096
Q01105	Protein SET OS=Homo sapiens OX=9606 GN=SET PE=1 SV=3	-0,054587225	0,382832
Q9NUJ1	Mycophenolic acid acyl-glucuronide esterase, mitochondrial OS=Homo sapiens OX=9606 GN=ABHD10 PE=1 SV=1	-0,049961779	0,382633
O60664	Perilipin-3 OS=Homo sapiens OX=9606 GN=PLIN3 PE=1 SV=3	-0,036332022	0,382148
P61457	Pterin-4-alpha-carbinolamine dehydratase OS=Homo sapiens OX=9606 GN=PCBD1 PE=1 SV=2	0,084112752	0,381742
Q6UX71	Plexin domain-containing protein 2 OS=Homo sapiens OX=9606 GN=PLXDC2 PE=1 SV=1	0,160699172	0,38173
Q8WVV9	Heterogeneous nuclear ribonucleoprotein L-like OS=Homo sapiens OX=9606 GN=HNRNPLL PE=1 SV=1	0,04784611	0,380744
Q9UQR1	Zinc finger protein 148 OS=Homo sapiens OX=9606 GN=ZNF148 PE=1 SV=2	-0,075485567	0,380257
Q5U5X0	Complex III assembly factor LYRM7 OS=Homo sapiens OX=9606 GN=LYRM7 PE=1 SV=1	-0,159961102	0,379897
Q9UBF2	Coatomer subunit gamma-2 OS=Homo sapiens OX=9606 GN=COPG2 PE=1 SV=1	-0,095687462	0,379667
O75179	Ankyrin repeat domain-containing protein 17 OS=Homo sapiens OX=9606 GN=ANKRD17 PE=1 SV=3	0,095671686	0,379599
Q13242	Serine/arginine-rich splicing factor 9 OS=Homo sapiens OX=9606 GN=SRSF9 PE=1 SV=1	-0,05696613	0,379448
Q53GS9	U4/U6.U5 tri-snRNP-associated protein 2 OS=Homo sapiens OX=9606 GN=USP39 PE=1 SV=2	0,043084148	0,379132
P62072	Mitochondrial import inner membrane translocase subunit Tim10 OS=Homo sapiens OX=9606 GN=TIMM10 PE=1 SV=1	-0,159526073	0,378817
O43318	Mitogen-activated protein kinase kinase kinase 7 OS=Homo sapiens OX=9606 GN=MAP3K7 PE=1 SV=1	-0,159057586	0,377653
O75083	WD repeat-containing protein 1 OS=Homo sapiens OX=9606 GN=WDR1 PE=1 SV=4	0,034502862	0,377644
Q8IWS0	PHD finger protein 6 OS=Homo sapiens OX=9606 GN=PHF6 PE=1 SV=1	-0,158997095	0,377503
Q14653	Interferon regulatory factor 3 OS=Homo sapiens OX=9606 GN=IRF3 PE=1 SV=1	0,158855305	0,377151
Q13535	Serine/threonine-protein kinase ATR OS=Homo sapiens OX=9606 GN=ATR PE=1 SV=3	-0,095047051	0,376891
P60900	Proteasome subunit alpha type-6 OS=Homo sapiens OX=9606 GN=PSMA6 PE=1 SV=1	-0,051373744	0,376659
P33121	Long-chain-fatty-acid--CoA ligase 1 OS=Homo sapiens OX=9606 GN=ACSL1 PE=1 SV=1	0,059862083	0,376578
P16403	Histone H1.2 OS=Homo sapiens OX=9606 GN=HIST1H1C PE=1 SV=2	-0,158595986	0,376507
P49366	Deoxyhypusine synthase OS=Homo sapiens OX=9606 GN=DHPS PE=1 SV=1	-0,05981992	0,376285
Q5R3I4	Tetratricopeptide repeat protein 38 OS=Homo sapiens OX=9606 GN=TTC38 PE=1 SV=1	0,158482881	0,376226
Q4G176	Acyl-CoA synthetase family member 3, mitochondrial OS=Homo sapiens OX=9606 GN=ACSF3 PE=1 SV=3	-0,074637598	0,375558
Q96AB6	Protein N-terminal asparagine amidohydrolase OS=Homo sapiens OX=9606 GN=NTAN1 PE=1 SV=3	-0,158200358	0,375525
Q10567	AP-1 complex subunit beta-1 OS=Homo sapiens OX=9606 GN=AP1B1 PE=1 SV=2	0,0414044	0,374877
P31321	cAMP-dependent protein kinase type I-beta regulatory subunit OS=Homo sapiens OX=9606 GN=PRKAR1B PE=1 SV=4	-0,15793009	0,374853
P08473	Neprilysin OS=Homo sapiens OX=9606 GN=MME PE=1 SV=2	-0,04898751	0,374382
Q0VF96	Cingulin-like protein 1 OS=Homo sapiens OX=9606 GN=CGNL1 PE=1 SV=2	0,055182512	0,374372
Q9Y4D8	Probable E3 ubiquitin-protein ligase HECTD4 OS=Homo sapiens OX=9606 GN=HECTD4 PE=1 SV=5	0,157415844	0,373577
Q13085	Acetyl-CoA carboxylase 1 OS=Homo sapiens OX=9606 GN=ACACA PE=1 SV=2	0,021776506	0,372979
O15160	DNA-directed RNA polymerases I and III subunit RPAC1 OS=Homo sapiens OX=9606 GN=POLR1C PE=1 SV=1	0,063204947	0,372693
O15111	Inhibitor of nuclear factor kappa-B kinase subunit alpha OS=Homo sapiens OX=9606 GN=CHUK PE=1 SV=2	-0,074105588	0,372615
P05198	Eukaryotic translation initiation factor 2 subunit 1 OS=Homo sapiens OX=9606 GN=EIF2S1 PE=1 SV=3	-0,038992061	0,372533
P35579	Myosin-9 OS=Homo sapiens OX=9606 GN=MYH9 PE=1 SV=4	0,015961719	0,371577
Q9C0B1	Alpha-ketoglutarate-dependent dioxygenase FTO OS=Homo sapiens OX=9606 GN=FTO PE=1 SV=3	0,063005693	0,371398
P07919	Cytochrome b-c1 complex subunit 6, mitochondrial OS=Homo sapiens OX=9606 GN=UQCRH PE=1 SV=2	0,156519731	0,371352
P56134	ATP synthase subunit f, mitochondrial OS=Homo sapiens OX=9606 GN=ATP5MF PE=1 SV=3	-0,113089092	0,371336
P60033	CD81 antigen OS=Homo sapiens OX=9606 GN=CD81 PE=1 SV=1	0,113054093	0,371211
Q5XPI4	E3 ubiquitin-protein ligase RNF123 OS=Homo sapiens OX=9606 GN=RNF123 PE=1 SV=1	-0,156456451	0,371195

Q9NVA2	Septin-11 OS=Homo sapiens OX=9606 GN=SEPTIN11 PE=1 SV=3	0,062927922	0,370893
Q8IUR0	Trafficking protein particle complex subunit 5 OS=Homo sapiens OX=9606 GN=TRAPPC5 PE=1 SV=1	-0,081831176	0,370393
Q9UJV9	Probable ATP-dependent RNA helicase DDX41 OS=Homo sapiens OX=9606 GN=DDX41 PE=1 SV=2	-0,112822766	0,37039
A5D8V6	Vacuolar protein sorting-associated protein 37C OS=Homo sapiens OX=9606 GN=VPS37C PE=1 SV=2	0,224942921	0,36978
Q14699	Raftlin OS=Homo sapiens OX=9606 GN=RFTN1 PE=1 SV=4	-0,055613446	0,369508
Q96CP2	FLYWCH family member 2 OS=Homo sapiens OX=9606 GN=FLYWCH2 PE=1 SV=1	0,081642056	0,369455
Q9BR76	Coronin-1B OS=Homo sapiens OX=9606 GN=CORO1B PE=1 SV=1	0,043419102	0,369316
P46940	Ras GTPase-activating-like protein IQGAP1 OS=Homo sapiens OX=9606 GN=IQGAP1 PE=1 SV=1	0,019866471	0,368592
Q9Y3C4	EKC/KEOPS complex subunit TPRKB OS=Homo sapiens OX=9606 GN=TPRKB PE=1 SV=1	0,155331985	0,368404
Q2TAY7	WD40 repeat-containing protein SMU1 OS=Homo sapiens OX=9606 GN=SMU1 PE=1 SV=2	0,046400606	0,368039
Q15796	Mothers against decapentaplegic homolog 2 OS=Homo sapiens OX=9606 GN=SMAD2 PE=1 SV=1	0,081338953	0,367952
P55199	RNA polymerase II elongation factor ELL OS=Homo sapiens OX=9606 GN=ELL PE=1 SV=1	-0,155062327	0,367735
Q15654	Thyroid receptor-interacting protein 6 OS=Homo sapiens OX=9606 GN=TRIP6 PE=1 SV=3	-0,043161637	0,366896
P25098	Beta-adrenergic receptor kinase 1 OS=Homo sapiens OX=9606 GN=GRK2 PE=1 SV=2	0,092722642	0,366837
Q66K74	Microtubule-associated protein 1S OS=Homo sapiens OX=9606 GN=MAP1S PE=1 SV=2	0,037471786	0,366539
O14727	Apoptotic protease-activating factor 1 OS=Homo sapiens OX=9606 GN=APAF1 PE=1 SV=2	0,111726835	0,366501
P13807	Glycogen [starch] synthase, muscle OS=Homo sapiens OX=9606 GN=GYS1 PE=1 SV=2	-0,066967527	0,366433
Q9NVR0	Kelch-like protein 11 OS=Homo sapiens OX=9606 GN=KLHL11 PE=1 SV=1	-0,154473785	0,366274
P54727	UV excision repair protein RAD23 homolog B OS=Homo sapiens OX=9606 GN=RAD23B PE=1 SV=1	-0,052419286	0,366065
Q96DZ1	Endoplasmic reticulum lectin 1 OS=Homo sapiens OX=9606 GN=ERLEC1 PE=1 SV=1	-0,153866643	0,364767
P42765	3-ketoacyl-CoA thiolase, mitochondrial OS=Homo sapiens OX=9606 GN=ACAA2 PE=1 SV=2	-0,044397066	0,364739
P09417	Dihydropteridine reductase OS=Homo sapiens OX=9606 GN=QDPR PE=1 SV=2	-0,080620315	0,364392
P50914	60S ribosomal protein L14 OS=Homo sapiens OX=9606 GN=RPL14 PE=1 SV=4	-0,09210754	0,364184
Q9UK41	Vacuolar protein sorting-associated protein 28 homolog OS=Homo sapiens OX=9606 GN=VPS28 PE=1 SV=1	-0,058067158	0,364144
Q12824	SWI/SNF-related matrix-associated actin-dependent regulator of chromatin subfamily B member 1 OS=Homo sapiens OX=	0,110996246	0,36391
Q15526	Surfeit locus protein 1 OS=Homo sapiens OX=9606 GN=SURF1 PE=1 SV=1	0,153473336	0,363792
Q96RD7	Pannexin-1 OS=Homo sapiens OX=9606 GN=PANX1 PE=1 SV=4	0,153313877	0,363396
P62253	Ubiquitin-conjugating enzyme E2 G1 OS=Homo sapiens OX=9606 GN=UBE2G1 PE=1 SV=3	-0,072389203	0,363145
P35270	Sepiapterin reductase OS=Homo sapiens OX=9606 GN=SPR PE=1 SV=1	0,049678255	0,362952
Q9BXJ4	Complement C1q tumor necrosis factor-related protein 3 OS=Homo sapiens OX=9606 GN=C1QTNF3 PE=1 SV=1	0,378320526	0,362823
Q9C091	GREB1-like protein OS=Homo sapiens OX=9606 GN=GREB1L PE=1 SV=2	0,152873984	0,362305
Q15386	Ubiquitin-protein ligase E3C OS=Homo sapiens OX=9606 GN=UBE3C PE=1 SV=3	0,072058237	0,361323
P49356	Protein farnesyltransferase subunit beta OS=Homo sapiens OX=9606 GN=FNTB PE=1 SV=1	0,152457423	0,361271
P84022	Mothers against decapentaplegic homolog 3 OS=Homo sapiens OX=9606 GN=SMAD3 PE=1 SV=1	-0,111182835	0,359777
P05556	Integrin beta-1 OS=Homo sapiens OX=9606 GN=ITGB1 PE=1 SV=2	-0,030171435	0,359536
P36405	ADP-ribosylation factor-like protein 3 OS=Homo sapiens OX=9606 GN=ARL3 PE=1 SV=2	-0,057280937	0,358719
Q9BXY0	Protein MAK16 homolog OS=Homo sapiens OX=9606 GN=MAK16 PE=1 SV=2	0,109530068	0,358719
O15446	DNA-directed RNA polymerase I subunit RPA34 OS=Homo sapiens OX=9606 GN=CD3EAP PE=1 SV=1	0,151145208	0,358017
Q5TFE4	5'-nucleotidase domain-containing protein 1 OS=Homo sapiens OX=9606 GN=NT5DC1 PE=1 SV=1	0,079180524	0,357275
Q05823	2-5A-dependent ribonuclease OS=Homo sapiens OX=9606 GN=RNASEL PE=1 SV=2	-0,108890924	0,356458
Q9GZS3	WD repeat-containing protein 61 OS=Homo sapiens OX=9606 GN=WDR61 PE=1 SV=1	0,056891876	0,35604
O95168	NADH dehydrogenase [ubiquinone] 1 beta subcomplex subunit 4 OS=Homo sapiens OX=9606 GN=NDUFB4 PE=1 SV=3	-0,108511617	0,355118
P37108	Signal recognition particle 14 kDa protein OS=Homo sapiens OX=9606 GN=SRP14 PE=1 SV=2	-0,064991831	0,354573
Q9Y216	Myotubularin-related protein 7 OS=Homo sapiens OX=9606 GN=MTMR7 PE=1 SV=3	-0,058404198	0,354502
Q9NPJ3	Acyl-coenzyme A thioesterase 13 OS=Homo sapiens OX=9606 GN=ACOT13 PE=1 SV=1	-0,149690342	0,354409
Q6PD62	RNA polymerase-associated protein CTR9 homolog OS=Homo sapiens OX=9606 GN=CTR9 PE=1 SV=1	-0,050895758	0,354359
Q9NWF9	E3 ubiquitin-protein ligase RNF216 OS=Homo sapiens OX=9606 GN=RNF216 PE=1 SV=3	0,084330511	0,353848
P48047	ATP synthase subunit O, mitochondrial OS=Homo sapiens OX=9606 GN=ATP5PO PE=1 SV=1	0,056555925	0,353729
Q9Y6I3	Epsin-1 OS=Homo sapiens OX=9606 GN=EPN1 PE=1 SV=2	-0,064700129	0,352827
Q15276	Rab GTPase-binding effector protein 1 OS=Homo sapiens OX=9606 GN=RABEP1 PE=1 SV=2	-0,060066068	0,352379
O43447	Peptidyl-prolyl cis-trans isomerase H OS=Homo sapiens OX=9606 GN=PPIH PE=1 SV=1	0,059995445	0,351924
O75947	ATP synthase subunit d, mitochondrial OS=Homo sapiens OX=9606 GN=ATP5PD PE=1 SV=3	-0,059983902	0,35185
Q9BQ67	Glutamate-rich WD repeat-containing protein 1 OS=Homo sapiens OX=9606 GN=GRWD1 PE=1 SV=1	0,078061842	0,351759
O43823	A-kinase anchor protein 8 OS=Homo sapiens OX=9606 GN=AKAP8 PE=1 SV=1	0,089124111	0,351349
O75448	Mediator of RNA polymerase II transcription subunit 24 OS=Homo sapiens OX=9606 GN=MED24 PE=1 SV=1	-0,064420173	0,351153
Q07617	Sperm-associated antigen 1 OS=Homo sapiens OX=9606 GN=SPAG1 PE=1 SV=3	0,107271785	0,350739
Q13162	Peroxiredoxin-4 OS=Homo sapiens OX=9606 GN=PRDX4 PE=1 SV=1	-0,048156349	0,350724
P23258	Tubulin gamma-1 chain OS=Homo sapiens OX=9606 GN=TUBG1 PE=1 SV=2	-0,041426642	0,350662
P22830	Ferrochelatase, mitochondrial OS=Homo sapiens OX=9606 GN=FECH PE=1 SV=2	0,147954989	0,350108
P14174	Macrophage migration inhibitory factor OS=Homo sapiens OX=9606 GN=MIF PE=1 SV=4	0,147879471	0,349921
Q6VMQ6	Activating transcription factor 7-interacting protein 1 OS=Homo sapiens OX=9606 GN=ATF7IP PE=1 SV=3	-0,103620094	0,349475
Q9H832	Ubiquitin-conjugating enzyme E2 Z OS=Homo sapiens OX=9606 GN=UBE2Z PE=1 SV=2	-0,050247271	0,349396
P63172	Dynein light chain Tctex-type 1 OS=Homo sapiens OX=9606 GN=DYNLT1 PE=1 SV=1	0,077421495	0,348608
Q9BSQ5	Cerebral cavernous malformations 2 protein OS=Homo sapiens OX=9606 GN=CCM2 PE=1 SV=1	-0,147101573	0,347993
Q9BYD6	39S ribosomal protein L1, mitochondrial OS=Homo sapiens OX=9606 GN=MRPL1 PE=1 SV=2	-0,106229123	0,347062
P57060	RWD domain-containing protein 2B OS=Homo sapiens OX=9606 GN=RWDD2B PE=1 SV=1	-0,146646051	0,346864
P48643	T-complex protein 1 subunit epsilon OS=Homo sapiens OX=9606 GN=CCT5 PE=1 SV=1	0,028696743	0,34632
Q14677	Clathrin interactor 1 OS=Homo sapiens OX=9606 GN=CLINT1 PE=1 SV=1	0,042254552	0,345391
Q9BZL4	Protein phosphatase 1 regulatory subunit 12C OS=Homo sapiens OX=9606 GN=PPP1R12C PE=1 SV=1	0,055241176	0,344708
Q8TDN6	Ribosome biogenesis protein BRX1 homolog OS=Homo sapiens OX=9606 GN=BRIX1 PE=1 SV=2	-0,047367871	0,344416
O60925	Prefoldin subunit 1 OS=Homo sapiens OX=9606 GN=PFDN1 PE=1 SV=2	-0,068946882	0,344268
P41223	Protein BUD31 homolog OS=Homo sapiens OX=9606 GN=BUD31 PE=1 SV=2	-0,076459124	0,34388
P53602	Diphosphomevalonate decarboxylase OS=Homo sapiens OX=9606 GN=MVD PE=1 SV=1	-0,105299119	0,343786
Q9NNW7	Thioredoxin reductase 2, mitochondrial OS=Homo sapiens OX=9606 GN=TXNRD2 PE=1 SV=3	-0,094546434	0,343772
Q99698	Lysosomal-trafficking regulator OS=Homo sapiens OX=9606 GN=LYST PE=1 SV=3	-0,145169644	0,343207
Q02880	DNA topoisomerase 2-beta OS=Homo sapiens OX=9606 GN=TOP2B PE=1 SV=3	-0,035277197	0,342937
Q9H089	Large subunit GTPase 1 homolog OS=Homo sapiens OX=9606 GN=LSG1 PE=1 SV=2	-0,068672159	0,342768
P05023	Sodium/potassium-transporting ATPase subunit alpha-1 OS=Homo sapiens OX=9606 GN=ATP1A1 PE=1 SV=1	0,027910472	0,341728
P84157	Matrix-remodeling-associated protein 7 OS=Homo sapiens OX=9606 GN=MXRA7 PE=1 SV=1	-0,104635962	0,341452
Q7LBR1	Charged multivesicular body protein 1b OS=Homo sapiens OX=9606 GN=CHMP1B PE=1 SV=1	-0,104503758	0,340987
Q9HCN4	GPN-loop GTPase 1 OS=Homo sapiens OX=9606 GN=GPN1 PE=1 SV=1	0,062527173	0,339864
Q9UPU7	TBC1 domain family member 2B OS=Homo sapiens OX=9606 GN=TBC1D2B PE=1 SV=2	0,143604645	0,339332
O95251	Histone acetyltransferase KAT7 OS=Homo sapiens OX=9606 GN=KAT7 PE=1 SV=1	0,14338663	0,338792
Q13885	Tubulin beta-2A chain OS=Homo sapiens OX=9606 GN=TUBB2A PE=1 SV=1	0,026478993	0,338574
O75676	Ribosomal protein S6 kinase alpha-4 OS=Homo sapiens OX=9606 GN=RPS6KA4 PE=1 SV=1	-0,14328146	0,338532
O00459	Phosphatidylinositol 3-kinase regulatory subunit beta OS=Homo sapiens OX=9606 GN=PIK3R2 PE=1 SV=2	0,103779228	0,338439
P49840	Glycogen synthase kinase-3 alpha OS=Homo sapiens OX=9606 GN=GSK3A PE=1 SV=2	-0,057870882	0,338283
Q6QNK2	Adhesion G-protein coupled receptor D1 OS=Homo sapiens OX=9606 GN=ADGRD1 PE=1 SV=1	0,253949795	0,337656
P61009	Signal peptidase complex subunit 3 OS=Homo sapiens OX=9606 GN=SPCS3 PE=1 SV=1	0,103498373	0,337452
Q9NRG0	Chromatin accessibility complex protein 1 OS=Homo sapiens OX=9606 GN=CHRAC1 PE=1 SV=1	0,16205774	0,336856
Q92947	Glutaryl-CoA dehydrogenase, mitochondrial OS=Homo sapiens OX=9606 GN=GCDH PE=1 SV=1	-0,142528531	0,336668

P24539	ATP synthase F(0) complex subunit B1, mitochondrial OS=Homo sapiens OX=9606 GN=ATP5PB PE=1 SV=2	0,051052346	0,336323
Q8N3U4	Cohesin subunit SA-2 OS=Homo sapiens OX=9606 GN=STAG2 PE=1 SV=3	0,142249934	0,335979
Q96JB2	Conserved oligomeric Golgi complex subunit 3 OS=Homo sapiens OX=9606 GN=COG3 PE=1 SV=3	0,102850618	0,335177
Q9GZT4	Serine racemase OS=Homo sapiens OX=9606 GN=SRR PE=1 SV=1	-0,084939688	0,333456
O95684	FGFR1 oncogene partner OS=Homo sapiens OX=9606 GN=FGFR1OP PE=1 SV=1	-0,141000191	0,332887
P20337	Ras-related protein Rab-3B OS=Homo sapiens OX=9606 GN=RAB3B PE=1 SV=2	-0,061334382	0,332781
Q15007	Pre-mRNA-splicing regulator WTAP OS=Homo sapiens OX=9606 GN=WTAP PE=1 SV=2	-0,074098511	0,332322
Q53SF7	Cordon-bleu protein-like 1 OS=Homo sapiens OX=9606 GN=COBLL1 PE=1 SV=2	-0,140566994	0,331815
Q9ULW0	Targeting protein for Xklp2 OS=Homo sapiens OX=9606 GN=TPX2 PE=1 SV=2	-0,043795005	0,330941
Q9ULU4	Protein kinase C-binding protein 1 OS=Homo sapiens OX=9606 GN=ZMYND8 PE=1 SV=2	-0,060883326	0,330108
O60832	H/ACA ribonucleoprotein complex subunit DKC1 OS=Homo sapiens OX=9606 GN=DKC1 PE=1 SV=3	0,047655669	0,329676
O95782	AP-2 complex subunit alpha-1 OS=Homo sapiens OX=9606 GN=AP2A1 PE=1 SV=3	-0,021649297	0,329425
Q86YV9	Hermansky-Pudlak syndrome 6 protein OS=Homo sapiens OX=9606 GN=HPS6 PE=1 SV=1	-0,083945344	0,329223
O94953	Lysine-specific demethylase 4B OS=Homo sapiens OX=9606 GN=KDM4B PE=1 SV=4	0,18246499	0,32916
Q9UKS6	Protein kinase C and casein kinase substrate in neurons protein 3 OS=Homo sapiens OX=9606 GN=PACSIN3 PE=1 SV=2	-0,073352804	0,328682
P41227	N-alpha-acetyltransferase 10 OS=Homo sapiens OX=9606 GN=NAA10 PE=1 SV=1	-0,041792981	0,328044
Q9C005	Protein dpy-30 homolog OS=Homo sapiens OX=9606 GN=DPY30 PE=1 SV=1	0,08354448	0,327518
Q53GS7	Nucleoporin GLE1 OS=Homo sapiens OX=9606 GN=GLE1 PE=1 SV=2	0,065845686	0,327394
P36507	Dual specificity mitogen-activated protein kinase kinase 2 OS=Homo sapiens OX=9606 GN=MAP2K2 PE=1 SV=1	-0,038913925	0,327372
Q8IYT2	Cap-specific mRNA (nucleoside-2'-O-)-methyltransferase 2 OS=Homo sapiens OX=9606 GN=CMTR2 PE=1 SV=2	-0,060168519	0,327355
P14324	Farnesyl pyrophosphate synthase OS=Homo sapiens OX=9606 GN=FDPS PE=1 SV=4	-0,056117269	0,327087
Q96PE2	Rho guanine nucleotide exchange factor 17 OS=Homo sapiens OX=9606 GN=ARHGEF17 PE=1 SV=1	-0,140984481	0,326615
Q9NR56	Muscleblind-like protein 1 OS=Homo sapiens OX=9606 GN=MBNL1 PE=1 SV=2	0,100348865	0,326407
Q8NEY8	Periphilin-1 OS=Homo sapiens OX=9606 GN=PPHLN1 PE=1 SV=2	0,100211777	0,325927
O00244	Copper transport protein ATOX1 OS=Homo sapiens OX=9606 GN=ATOX1 PE=1 SV=1	0,072706634	0,325533
O95674	Phosphatidate cytidylyltransferase 2 OS=Homo sapiens OX=9606 GN=CDS2 PE=1 SV=1	-0,137885588	0,325185
P62837	Ubiquitin-conjugating enzyme E2 D2 OS=Homo sapiens OX=9606 GN=UBE2D2 PE=1 SV=1	-0,099984471	0,325131
Q5SXM2	snRNA-activating protein complex subunit 4 OS=Homo sapiens OX=9606 GN=SNAPC4 PE=1 SV=1	0,137732874	0,324808
Q15208	Serine/threonine-protein kinase 38 OS=Homo sapiens OX=9606 GN=STK38 PE=1 SV=1	0,059946943	0,32457
Q96KP1	Exocyst complex component 2 OS=Homo sapiens OX=9606 GN=EXOC2 PE=1 SV=1	-0,046955694	0,324382
O60504	Vinexin OS=Homo sapiens OX=9606 GN=SORBS3 PE=1 SV=2	-0,044631588	0,322671
O43399	Tumor protein D54 OS=Homo sapiens OX=9606 GN=TPD52L2 PE=1 SV=2	0,039701108	0,322565
Q9Y673	Dolichyl-phosphate beta-glucosyltransferase OS=Homo sapiens OX=9606 GN=ALG5 PE=1 SV=1	0,145746236	0,322389
P62987	Ubiquitin-60S ribosomal protein L40 OS=Homo sapiens OX=9606 GN=UBA52 PE=1 SV=2	-0,071926415	0,321737
Q93063	Exostosin-2 OS=Homo sapiens OX=9606 GN=EXT2 PE=1 SV=1	-0,136101528	0,320777
Q86VV8	Rotatin OS=Homo sapiens OX=9606 GN=RTTN PE=1 SV=3	0,136090806	0,32075
Q16342	Programmed cell death protein 2 OS=Homo sapiens OX=9606 GN=PDCD2 PE=1 SV=2	-0,135977714	0,320471
O15460	Prolyl 4-hydroxylase subunit alpha-2 OS=Homo sapiens OX=9606 GN=P4HA2 PE=1 SV=1	0,033993166	0,320324
O95182	NADH dehydrogenase [ubiquinone] 1 alpha subcomplex subunit 7 OS=Homo sapiens OX=9606 GN=NDUFA7 PE=1 SV=3	0,135870403	0,320206
Q9NVT9	Armadillo repeat-containing protein 1 OS=Homo sapiens OX=9606 GN=ARMC1 PE=1 SV=1	-0,081813233	0,32017
P08134	Rho-related GTP-binding protein RhoC OS=Homo sapiens OX=9606 GN=RHOC PE=1 SV=1	-0,044288256	0,319959
Q15560	Transcription elongation factor A protein 2 OS=Homo sapiens OX=9606 GN=TCEA2 PE=1 SV=1	0,098503589	0,319955
O60573	Eukaryotic translation initiation factor 4E type 2 OS=Homo sapiens OX=9606 GN=EIF4E2 PE=1 SV=1	-0,1357578	0,319928
Q92878	DNA repair protein RAD50 OS=Homo sapiens OX=9606 GN=RAD50 PE=1 SV=1	-0,040775811	0,319318
Q14627	Interleukin-13 receptor subunit alpha-2 OS=Homo sapiens OX=9606 GN=IL13RA2 PE=1 SV=1	-0,071393207	0,319146
O43815	Striatin OS=Homo sapiens OX=9606 GN=STRN PE=1 SV=4	-0,037987194	0,318849
Q99471	Prefoldin subunit 5 OS=Homo sapiens OX=9606 GN=PFDN5 PE=1 SV=2	0,05897397	0,318831
Q9P2K3	#N/D	0,13512646	0,318368
P61960	Ubiquitin-fold modifier 1 OS=Homo sapiens OX=9606 GN=UFM1 PE=1 SV=1	0,097934929	0,317969
Q07157	Tight junction protein ZO-1 OS=Homo sapiens OX=9606 GN=TJP1 PE=1 SV=3	0,032115368	0,317589
P0DMM9	Sulfotransferase 1A3 OS=Homo sapiens OX=9606 GN=SULT1A3 PE=1 SV=1	-0,051249272	0,31755
Q15813	Tubulin-specific chaperone E OS=Homo sapiens OX=9606 GN=TBCE PE=1 SV=1	-0,045851022	0,316053
Q86VW0	SEC14 domain and spectrin repeat-containing protein 1 OS=Homo sapiens OX=9606 GN=SESTD1 PE=1 SV=2	-0,134007154	0,315605
Q3ZCW2	Galectin-related protein OS=Homo sapiens OX=9606 GN=LGALSL PE=1 SV=2	-0,133588643	0,314572
Q99661	Kinesin-like protein KIF2C OS=Homo sapiens OX=9606 GN=KIF2C PE=1 SV=2	-0,05412635	0,314448
Q9UM54	Unconventional myosin-VI OS=Homo sapiens OX=9606 GN=MYO6 PE=1 SV=4	-0,037309942	0,312643
Q96BZ8	Leukocyte receptor cluster member 1 OS=Homo sapiens OX=9606 GN=LENG1 PE=1 SV=1	0,079994734	0,312475
P18583	Protein SON OS=Homo sapiens OX=9606 GN=SON PE=1 SV=4	0,026873395	0,310847
Q14118	Dystroglycan OS=Homo sapiens OX=9606 GN=DAG1 PE=1 SV=2	0,045105119	0,310449
P56962	Syntaxin-17 OS=Homo sapiens OX=9606 GN=STX17 PE=1 SV=2	0,13189476	0,310392
Q9H019	Mitochondrial fission regulator 1-like OS=Homo sapiens OX=9606 GN=MTFR1L PE=1 SV=2	-0,131628273	0,309735
Q8N5M4	Tetratricopeptide repeat protein 9C OS=Homo sapiens OX=9606 GN=TTC9C PE=1 SV=1	0,131580186	0,309616
Q6GMV3	Putative peptidyl-tRNA hydrolase PTRHD1 OS=Homo sapiens OX=9606 GN=PTRHD1 PE=1 SV=1	0,079211207	0,309167
Q99536	Synaptic vesicle membrane protein VAT-1 homolog OS=Homo sapiens OX=9606 GN=VAT1 PE=1 SV=2	0,034714605	0,308869
Q8N2F6	Armadillo repeat-containing protein 10 OS=Homo sapiens OX=9606 GN=ARMC10 PE=1 SV=1	-0,069258997	0,308805
Q9P212	1-phosphatidylinositol 4,5-bisphosphate phosphodiesterase epsilon-1 OS=Homo sapiens OX=9606 GN=PLCE1 PE=1 SV=3	-0,131176264	0,30862
P52758	2-iminobutanoate/2-iminopropanoate deaminase OS=Homo sapiens OX=9606 GN=RIDA PE=1 SV=1	0,095177229	0,30836
P56589	Peroxisomal biogenesis factor 3 OS=Homo sapiens OX=9606 GN=PEX3 PE=1 SV=1	0,13103777	0,308278
P29323	Ephrin type-B receptor 2 OS=Homo sapiens OX=9606 GN=EPHB2 PE=1 SV=5	-0,069061931	0,307853
Q9H788	SH2 domain-containing protein 4A OS=Homo sapiens OX=9606 GN=SH2D4A PE=1 SV=1	0,047021103	0,30742
Q9Y6D9	Mitotic spindle assembly checkpoint protein MAD1 OS=Homo sapiens OX=9606 GN=MAD1L1 PE=1 SV=2	0,062143816	0,307419
O95707	Ribonuclease P protein subunit p29 OS=Homo sapiens OX=9606 GN=POP4 PE=1 SV=2	-0,094715278	0,306754
Q86Y39	NADH dehydrogenase [ubiquinone] 1 alpha subcomplex subunit 11 OS=Homo sapiens OX=9606 GN=NDUFA11 PE=1 SV=3	-0,17056109	0,306732
O00571	ATP-dependent RNA helicase DDX3X OS=Homo sapiens OX=9606 GN=DDX3X PE=1 SV=3	0,025287239	0,306691
Q9BUQ8	Probable ATP-dependent RNA helicase DDX23 OS=Homo sapiens OX=9606 GN=DDX23 PE=1 SV=3	-0,035511529	0,306567
P51149	Ras-related protein Rab-7a OS=Homo sapiens OX=9606 GN=RAB7A PE=1 SV=1	-0,037870827	0,306359
Q9NUQ8	ATP-binding cassette sub-family F member 3 OS=Homo sapiens OX=9606 GN=ABCF3 PE=1 SV=2	-0,035435094	0,305848
P55789	FAD-linked sulfhydryl oxidase ALR OS=Homo sapiens OX=9606 GN=GFER PE=1 SV=2	-0,10710386	0,304395
Q8IY67	Ribonucleoprotein PTB-binding 1 OS=Homo sapiens OX=9606 GN=RAVER1 PE=1 SV=1	-0,040551106	0,304256
Q96KA5	Cleft lip and palate transmembrane protein 1-like protein OS=Homo sapiens OX=9606 GN=CLPTM1L PE=1 SV=1	0,0564561	0,304049
Q7Z7L1	Schlafen family member 11 OS=Homo sapiens OX=9606 GN=SLFN11 PE=1 SV=2	-0,093936718	0,304049
P21127	Cyclin-dependent kinase 11B OS=Homo sapiens OX=9606 GN=CDK11B PE=1 SV=4	-0,049214443	0,303841
P0C0L4	Complement C4-A OS=Homo sapiens OX=9606 GN=C4A PE=1 SV=2	-0,129182251	0,303704
Q8WWI1	LIM domain only protein 7 OS=Homo sapiens OX=9606 GN=LMO7 PE=1 SV=3	-0,018212053	0,303703
Q9Y2H1	Serine/threonine-protein kinase 38-like OS=Homo sapiens OX=9606 GN=STK38L PE=1 SV=3	-0,147820747	0,303452
Q96BP3	Peptidylprolyl isomerase domain and WD repeat-containing protein 1 OS=Homo sapiens OX=9606 GN=PPWD1 PE=1 SV=1	0,129062187	0,303408
Q8TCY9	Up-regulator of cell proliferation OS=Homo sapiens OX=9606 GN=URGCP PE=1 SV=2	-0,077763149	0,303065
Q9NZD8	Maspardin OS=Homo sapiens OX=9606 GN=SPG21 PE=1 SV=1	-0,077406014	0,301563
Q09472	#N/D	0,060998985	0,301278
Q13769	THO complex subunit 5 homolog OS=Homo sapiens OX=9606 GN=THOC5 PE=1 SV=2	-0,127867028	0,300464
Q7L5D6	Golgi to ER traffic protein 4 homolog OS=Homo sapiens OX=9606 GN=GET4 PE=1 SV=1	0,067373467	0,299709

P48730	Casein kinase I isoform delta OS=Homo sapiens OX=9606 GN=CSNK1D PE=1 SV=2	-0,127522113	0,299614
O00584	Ribonuclease T2 OS=Homo sapiens OX=9606 GN=RNASET2 PE=1 SV=2	0,127454019	0,299447
Q9H307	Pinin OS=Homo sapiens OX=9606 GN=PNN PE=1 SV=5	-0,032773305	0,299047
O75165	DnaJ homolog subfamily C member 13 OS=Homo sapiens OX=9606 GN=DNAJC13 PE=1 SV=5	-0,022975192	0,298693
Q6P6C2	RNA demethylase ALKBH5 OS=Homo sapiens OX=9606 GN=ALKBH5 PE=1 SV=2	-0,127133451	0,298657
Q9BW19	Kinesin-like protein KIFC1 OS=Homo sapiens OX=9606 GN=KIFC1 PE=1 SV=2	-0,127099921	0,298575
Q86UE4	Protein LYRIC OS=Homo sapiens OX=9606 GN=MTDH PE=1 SV=2	0,032677605	0,298096
Q658Y4	Protein FAM91A1 OS=Homo sapiens OX=9606 GN=FAM91A1 PE=1 SV=3	-0,076508056	0,297789
P48634	Protein PRRC2A OS=Homo sapiens OX=9606 GN=PRRC2A PE=1 SV=3	-0,048292424	0,297658
Q16643	Drebrin OS=Homo sapiens OX=9606 GN=DBN1 PE=1 SV=4	-0,030194519	0,29692
P57737	Coronin-7 OS=Homo sapiens OX=9606 GN=CORO7 PE=1 SV=2	-0,048056721	0,296081
Q6UN15	Pre-mRNA 3'-end-processing factor FIP1 OS=Homo sapiens OX=9606 GN=FIP1L1 PE=1 SV=1	0,066525212	0,295629
P14868	Aspartate--tRNA ligase, cytoplasmic OS=Homo sapiens OX=9606 GN=DARS PE=1 SV=2	-0,023727245	0,295606
Q10472	Polypeptide N-acetylgalactosaminyltransferase 1 OS=Homo sapiens OX=9606 GN=GALNT1 PE=1 SV=1	-0,05113373	0,29559
Q96I51	RCC1-like G exchanging factor-like protein OS=Homo sapiens OX=9606 GN=RCC1L PE=1 SV=2	0,037521812	0,295502
Q9NSV4	Protein diaphanous homolog 3 OS=Homo sapiens OX=9606 GN=DIAPH3 PE=1 SV=4	0,045320407	0,295347
Q9UJA5	tRNA (adenine(58)-N(1))-methyltransferase non-catalytic subunit TRM6 OS=Homo sapiens OX=9606 GN=TRMT6 PE=1 SV=	0,066436287	0,295201
O95479	GDH/6PGL endoplasmic bifunctional protein OS=Homo sapiens OX=9606 GN=H6PD PE=1 SV=2	0,125604057	0,294892
Q96QU8	Exportin-6 OS=Homo sapiens OX=9606 GN=XPO6 PE=1 SV=1	0,066244634	0,294281
P11182	Lipoamide acyltransferase component of branched-chain alpha-keto acid dehydrogenase complex, mitochondrial OS=Hor	0,090997165	0,293859
P52294	Importin subunit alpha-5 OS=Homo sapiens OX=9606 GN=KPNA1 PE=1 SV=3	-0,0595844	0,293715
P55957	BH3-interacting domain death agonist OS=Homo sapiens OX=9606 GN=BID PE=1 SV=1	-0,050828548	0,293677
Q9Y371	Endophilin-B1 OS=Homo sapiens OX=9606 GN=SH3GLB1 PE=1 SV=1	-0,050798439	0,293488
P21266	Glutathione S-transferase Mu 3 OS=Homo sapiens OX=9606 GN=GSTM3 PE=1 SV=3	-0,039192919	0,293187
Q9UKK3	Protein mono-ADP-ribosyltransferase PARP4 OS=Homo sapiens OX=9606 GN=PARP4 PE=1 SV=3	0,030537158	0,292876
P27105	Erythrocyte band 7 integral membrane protein OS=Homo sapiens OX=9606 GN=STOM PE=1 SV=3	0,04496794	0,292854
Q9HC52	Chromobox protein homolog 8 OS=Homo sapiens OX=9606 GN=CBX8 PE=1 SV=3	0,124548105	0,292294
O95159	Zinc finger protein-like 1 OS=Homo sapiens OX=9606 GN=ZFPL1 PE=1 SV=2	-0,059318036	0,292294
Q9H492	Microtubule-associated proteins 1A/1B light chain 3A OS=Homo sapiens OX=9606 GN=MAP1LC3A PE=1 SV=2	0,090517868	0,292201
Q9P0V9	Septin-10 OS=Homo sapiens OX=9606 GN=SEPTIN10 PE=1 SV=2	-0,032975162	0,292052
P62318	Small nuclear ribonucleoprotein Sm D3 OS=Homo sapiens OX=9606 GN=SNRPD3 PE=1 SV=1	0,075048108	0,291667
O95292	Vesicle-associated membrane protein-associated protein B/C OS=Homo sapiens OX=9606 GN=VAPB PE=1 SV=3	-0,047374433	0,291521
Q96BI1	Solute carrier family 22 member 18 OS=Homo sapiens OX=9606 GN=SLC22A18 PE=1 SV=3	0,124209411	0,291461
Q8NE71	ATP-binding cassette sub-family F member 1 OS=Homo sapiens OX=9606 GN=ABCF1 PE=1 SV=2	0,032906252	0,291389
Q86U42	Polyadenylate-binding protein 2 OS=Homo sapiens OX=9606 GN=PABPN1 PE=1 SV=3	-0,054267893	0,291287
P51003	Poly(A) polymerase alpha OS=Homo sapiens OX=9606 GN=PAPOLA PE=1 SV=4	0,044746098	0,291286
Q96BD5	PHD finger protein 21A OS=Homo sapiens OX=9606 GN=PHF21A PE=1 SV=1	-0,124052698	0,291076
Q8IYB9	Zinc finger protein 595 OS=Homo sapiens OX=9606 GN=ZNF595 PE=2 SV=2	0,038187227	0,290888
Q6UW63	Protein O-glucosyltransferase 2 OS=Homo sapiens OX=9606 GN=POGLUT2 PE=1 SV=1	-0,050350506	0,290683
Q7Z2W4	Zinc finger CCCH-type antiviral protein 1 OS=Homo sapiens OX=9606 GN=ZC3HAV1 PE=1 SV=3	-0,033804506	0,290561
Q8TEQ0	Sorting nexin-29 OS=Homo sapiens OX=9606 GN=SNX29 PE=1 SV=3	0,123773101	0,290388
P29279	CCN family member 2 OS=Homo sapiens OX=9606 GN=CCN2 PE=1 SV=2	0,074700615	0,290212
P50416	Carnitine O-palmitoyltransferase 1, liver isoform OS=Homo sapiens OX=9606 GN=CPT1A PE=1 SV=2	-0,040403854	0,289521
P42025	Beta-centractin OS=Homo sapiens OX=9606 GN=ACTR1B PE=1 SV=1	-0,089591892	0,289001
Q9Y6I9	Testis-expressed protein 264 OS=Homo sapiens OX=9606 GN=TEX264 PE=1 SV=1	0,089531157	0,288791
P67936	Tropomyosin alpha-4 chain OS=Homo sapiens OX=9606 GN=TPM4 PE=1 SV=3	0,027565693	0,288767
O95218	Zinc finger Ran-binding domain-containing protein 2 OS=Homo sapiens OX=9606 GN=ZRANB2 PE=1 SV=2	-0,053605869	0,287441
Q96S19	Methyltransferase-like 26 OS=Homo sapiens OX=9606 GN=METTL26 PE=1 SV=2	-0,089021416	0,287031
P52943	Cysteine-rich protein 2 OS=Homo sapiens OX=9606 GN=CRIP2 PE=1 SV=1	0,049765547	0,287025
O14920	Inhibitor of nuclear factor kappa-B kinase subunit beta OS=Homo sapiens OX=9606 GN=IKBKB PE=1 SV=1	-0,088934537	0,286731
O75427	Leucine-rich repeat and calponin homology domain-containing protein 4 OS=Homo sapiens OX=9606 GN=LRCH4 PE=1 SV=	0,122214921	0,286557
Q9UBI1	COMM domain-containing protein 3 OS=Homo sapiens OX=9606 GN=COMMD3 PE=1 SV=1	-0,064575753	0,28628
Q9BSU1	UPF0183 protein C16orf70 OS=Homo sapiens OX=9606 GN=C16orf70 PE=1 SV=1	0,088802519	0,286276
P62899	60S ribosomal protein L31 OS=Homo sapiens OX=9606 GN=RPL31 PE=1 SV=1	0,05338627	0,286166
Q07955	Serine/arginine-rich splicing factor 1 OS=Homo sapiens OX=9606 GN=SRSF1 PE=1 SV=2	-0,03679938	0,285562
Q99543	DnaJ homolog subfamily C member 2 OS=Homo sapiens OX=9606 GN=DNAJC2 PE=1 SV=4	-0,034300321	0,285295
P53779	#N/D	0,053167241	0,284977
Q8TCD5	5'(3')-deoxyribonucleotidase, cytosolic type OS=Homo sapiens OX=9606 GN=NT5C PE=1 SV=2	0,08814203	0,283998
Q9Y546	Leucine-rich repeat-containing protein 42 OS=Homo sapiens OX=9606 GN=LRRC42 PE=1 SV=1	0,121121701	0,283871
Q9Y5X1	Sorting nexin-9 OS=Homo sapiens OX=9606 GN=SNX9 PE=1 SV=1	0,052836999	0,282983
Q9H7E9	UPF0488 protein C8orf33 OS=Homo sapiens OX=9606 GN=C8orf33 PE=1 SV=1	0,034504832	0,282909
Q9UBD5	Origin recognition complex subunit 3 OS=Homo sapiens OX=9606 GN=ORC3 PE=1 SV=1	-0,049047303	0,282544
O75385	Serine/threonine-protein kinase ULK1 OS=Homo sapiens OX=9606 GN=ULK1 PE=1 SV=2	-0,119941044	0,280972
Q9NRN7	L-aminoadipate-semialdehyde dehydrogenase-phosphopantetheinyl transferase OS=Homo sapiens OX=9606 GN=AASDHI	0,063437245	0,280839
Q8NI22	Multiple coagulation factor deficiency protein 2 OS=Homo sapiens OX=9606 GN=MCFD2 PE=1 SV=1	-0,087183793	0,280697
Q9UK76	Jupiter microtubule associated homolog 1 OS=Homo sapiens OX=9606 GN=JPT1 PE=1 SV=3	-0,045695567	0,280346
O94915	Protein furry homolog-like OS=Homo sapiens OX=9606 GN=FRYL PE=1 SV=2	0,032701443	0,280287
Q9H1K1	Iron-sulfur cluster assembly enzyme ISCU, mitochondrial OS=Homo sapiens OX=9606 GN=ISCU PE=1 SV=2	0,147713524	0,279569
Q9H4L7	SWI/SNF-related matrix-associated actin-dependent regulator of chromatin subfamily A containing DEAD/H box 1 OS=Hor	-0,063080468	0,279136
O75381	Peroxisomal membrane protein PEX14 OS=Homo sapiens OX=9606 GN=PEX14 PE=1 SV=1	0,07708091	0,278883
O75400	Pre-mRNA-processing factor 40 homolog A OS=Homo sapiens OX=9606 GN=PRPF40A PE=1 SV=2	-0,032549387	0,278875
L0R6Q1	SLC35A4 upstream open reading frame protein OS=Homo sapiens OX=9606 GN=SLC35A4 PE=3 SV=1	-0,071831945	0,278237
O75489	NADH dehydrogenase [ubiquinone] iron-sulfur protein 3, mitochondrial OS=Homo sapiens OX=9606 GN=NDUFS3 PE=1 SV	-0,038883556	0,277732
Q8TEW0	Partitioning defective 3 homolog OS=Homo sapiens OX=9606 GN=PARD3 PE=1 SV=2	-0,118415409	0,277228
Q96ES7	SAGA-associated factor 29 OS=Homo sapiens OX=9606 GN=SGF29 PE=1 SV=1	-0,118385769	0,277155
P35244	Replication protein A 14 kDa subunit OS=Homo sapiens OX=9606 GN=RPA3 PE=1 SV=1	-0,056466759	0,27714
Q9H490	Phosphatidylinositol glycan anchor biosynthesis class U protein OS=Homo sapiens OX=9606 GN=PIGU PE=1 SV=3	0,117979855	0,276159
Q9NZI7	Upstream-binding protein 1 OS=Homo sapiens OX=9606 GN=UBP1 PE=1 SV=1	-0,117813509	0,275751
Q14254	Flotillin-2 OS=Homo sapiens OX=9606 GN=FLOT2 PE=1 SV=2	-0,042523317	0,275645
Q16832	Discoidin domain-containing receptor 2 OS=Homo sapiens OX=9606 GN=DDR2 PE=1 SV=2	0,062272238	0,275285
P20618	Proteasome subunit beta type-1 OS=Homo sapiens OX=9606 GN=PSMB1 PE=1 SV=2	-0,038553376	0,275181
Q9NSU2	Three-prime repair exonuclease 1 OS=Homo sapiens OX=9606 GN=TREX1 PE=1 SV=2	-0,117405051	0,27475
O60911	#N/D	-0,064603693	0,274056
A2RUR9	Coiled-coil domain-containing protein 144A OS=Homo sapiens OX=9606 GN=CCDC144A PE=2 SV=1	-0,117118046	0,274046
Q9BVK6	Transmembrane emp24 domain-containing protein 9 OS=Homo sapiens OX=9606 GN=TMED9 PE=1 SV=2	0,116916836	0,273553
Q99623	Prohibitin-2 OS=Homo sapiens OX=9606 GN=PHB2 PE=1 SV=2	-0,032937796	0,273037
P46459	Vesicle-fusing ATPase OS=Homo sapiens OX=9606 GN=NSF PE=1 SV=3	-0,02470583	0,273034
Q9UKZ1	CCR4-NOT transcription complex subunit 11 OS=Homo sapiens OX=9606 GN=CNOT11 PE=1 SV=1	0,116375522	0,272226
O15400	Syntaxin-7 OS=Homo sapiens OX=9606 GN=STX7 PE=1 SV=4	-0,061607867	0,272124
Q92871	Phosphomannomutase 1 OS=Homo sapiens OX=9606 GN=PMM1 PE=1 SV=2	0,115801769	0,271544
P49368	T-complex protein 1 subunit gamma OS=Homo sapiens OX=9606 GN=CCT3 PE=1 SV=4	0,02187358	0,270624

O75116	Rho-associated protein kinase 2 OS=Homo sapiens OX=9606 GN=ROCK2 PE=1 SV=4	-0,022497313	0,270102
P61599	N-alpha-acetyltransferase 20 OS=Homo sapiens OX=9606 GN=NAA20 PE=1 SV=1	0,061080681	0,269619
P62714	Serine/threonine-protein phosphatase 2A catalytic subunit beta isoform OS=Homo sapiens OX=9606 GN=PPP2CB PE=1 SV	-0,03364941	0,269476
Q9Y4P8	WD repeat domain phosphoinositide-interacting protein 2 OS=Homo sapiens OX=9606 GN=WIPI2 PE=1 SV=1	-0,069485024	0,268486
P07099	Epoxide hydrolase 1 OS=Homo sapiens OX=9606 GN=EPHX1 PE=1 SV=1	-0,031386852	0,268114
P16144	Integrin beta-4 OS=Homo sapiens OX=9606 GN=ITGB4 PE=1 SV=5	-0,028848835	0,267975
Q9NW15	Anoctamin-10 OS=Homo sapiens OX=9606 GN=ANO10 PE=1 SV=2	0,054720424	0,267912
Q9Y3E0	Vesicle transport protein GOT1B OS=Homo sapiens OX=9606 GN=GOLT1B PE=1 SV=1	-0,083455161	0,26789
Q8IWW6	Rho GTPase-activating protein 12 OS=Homo sapiens OX=9606 GN=ARHGAP12 PE=1 SV=1	-0,069324062	0,267819
Q6ZRP7	Sulfhydryl oxidase 2 OS=Homo sapiens OX=9606 GN=QSOX2 PE=1 SV=3	0,04666951	0,267777
Q15036	Sorting nexin-17 OS=Homo sapiens OX=9606 GN=SNX17 PE=1 SV=1	0,035879696	0,26644
Q13451	Peptidyl-prolyl cis-trans isomerase FKBP5 OS=Homo sapiens OX=9606 GN=FKBP5 PE=1 SV=2	0,113888603	0,266134
P62701	40S ribosomal protein S4, X isoform OS=Homo sapiens OX=9606 GN=RPS4X PE=1 SV=2	-0,037370077	0,266066
Q8NG11	Tetraspanin-14 OS=Homo sapiens OX=9606 GN=TSPAN14 PE=1 SV=1	0,150801385	0,264758
Q9NP92	39S ribosomal protein S30, mitochondrial OS=Homo sapiens OX=9606 GN=MRPS30 PE=1 SV=2	-0,113135105	0,264289
Q9BQS8	FYVE and coiled-coil domain-containing protein 1 OS=Homo sapiens OX=9606 GN=FYCO1 PE=1 SV=3	0,082377431	0,264201
P32321	Deoxycytidylate deaminase OS=Homo sapiens OX=9606 GN=DCTD PE=1 SV=2	-0,043237871	0,264097
Q9Y262	Eukaryotic translation initiation factor 3 subunit L OS=Homo sapiens OX=9606 GN=EIF3L PE=1 SV=1	-0,02634228	0,262492
P55196	Afadin OS=Homo sapiens OX=9606 GN=AFDN PE=1 SV=3	0,045638374	0,261407
P09382	Galectin-1 OS=Homo sapiens OX=9606 GN=LGALS1 PE=1 SV=2	0,042779694	0,261083
Q9HD42	Charged multivesicular body protein 1a OS=Homo sapiens OX=9606 GN=CHMP1A PE=1 SV=1	0,045564752	0,260952
Q9P2E9	Ribosome-binding protein 1 OS=Homo sapiens OX=9606 GN=RRBP1 PE=1 SV=5	0,01819628	0,260674
Q7Z5G4	Golgin subfamily A member 7 OS=Homo sapiens OX=9606 GN=GOLGA7 PE=1 SV=2	0,111604118	0,260544
Q9UI26	Importin-11 OS=Homo sapiens OX=9606 GN=IPO11 PE=1 SV=1	0,033785681	0,260357
Q96AE7	Tetratricopeptide repeat protein 17 OS=Homo sapiens OX=9606 GN=TTC17 PE=1 SV=1	-0,081110957	0,259872
Q9H6X2	Anthrax toxin receptor 1 OS=Homo sapiens OX=9606 GN=ANTXR1 PE=1 SV=2	0,048710531	0,259222
P47914	60S ribosomal protein L29 OS=Homo sapiens OX=9606 GN=RPL29 PE=1 SV=2	-0,110945746	0,258935
P27448	MAP/microtubule affinity-regulating kinase 3 OS=Homo sapiens OX=9606 GN=MARK3 PE=1 SV=5	0,070325926	0,258643
Q03519	Antigen peptide transporter 2 OS=Homo sapiens OX=9606 GN=TAP2 PE=1 SV=1	0,048571985	0,258429
Q15070	Mitochondrial inner membrane protein OXA1L OS=Homo sapiens OX=9606 GN=OXA1L PE=1 SV=3	-0,110692281	0,258315
Q8IXM2	Chromatin complexes subunit BAP18 OS=Homo sapiens OX=9606 GN=BAP18 PE=1 SV=1	-0,067016923	0,258277
Q9NPQ8	Synembryn-A OS=Homo sapiens OX=9606 GN=RIC8A PE=1 SV=3	-0,026531847	0,258105
P19823	Inter-alpha-trypsin inhibitor heavy chain H2 OS=Homo sapiens OX=9606 GN=ITIH2 PE=1 SV=2	-0,052768841	0,257649
Q06190	Serine/threonine-protein phosphatase 2A regulatory subunit B'' subunit alpha OS=Homo sapiens OX=9606 GN=PPP2R3A I	0,116144443	0,257472
P09493	Tropomyosin alpha-1 chain OS=Homo sapiens OX=9606 GN=TPM1 PE=1 SV=2	0,036239345	0,257396
P07948	Tyrosine-protein kinase Lyn OS=Homo sapiens OX=9606 GN=LYN PE=1 SV=3	0,118853458	0,256902
Q14764	Major vault protein OS=Homo sapiens OX=9606 GN=MVP PE=1 SV=4	-0,018074307	0,255769
P23919	Thymidylate kinase OS=Homo sapiens OX=9606 GN=DTYMK PE=1 SV=4	-0,032049653	0,255679
Q15751	Probable E3 ubiquitin-protein ligase HERC1 OS=Homo sapiens OX=9606 GN=HERC1 PE=1 SV=2	0,109303812	0,254923
Q9HB19	Pleckstrin homology domain-containing family A member 2 OS=Homo sapiens OX=9606 GN=PLEKHA2 PE=1 SV=2	-0,079525604	0,254463
P46782	40S ribosomal protein S5 OS=Homo sapiens OX=9606 GN=RPS5 PE=1 SV=4	-0,031737223	0,252996
Q9H9A5	CCR4-NOT transcription complex subunit 10 OS=Homo sapiens OX=9606 GN=CNOT10 PE=1 SV=1	-0,057539936	0,252872
Q9BSB4	Autophagy-related protein 101 OS=Homo sapiens OX=9606 GN=ATG101 PE=1 SV=1	0,108420348	0,252766
Q96EY1	DnaJ homolog subfamily A member 3, mitochondrial OS=Homo sapiens OX=9606 GN=DNAJA3 PE=1 SV=2	0,065516991	0,252096
Q13888	General transcription factor IIH subunit 2 OS=Homo sapiens OX=9606 GN=GTF2H2 PE=1 SV=1	-0,049653143	0,25204
P35914	Hydroxymethylglutaryl-CoA lyase, mitochondrial OS=Homo sapiens OX=9606 GN=HMGCL PE=1 SV=2	-0,10806943	0,251909
Q765P7	Protein MTSS 2 OS=Homo sapiens OX=9606 GN=MTSS2 PE=1 SV=1	-0,038990243	0,251037
Q9NQC8	Intraflagellar transport protein 46 homolog OS=Homo sapiens OX=9606 GN=IFT46 PE=1 SV=1	-0,107711194	0,251035
Q9UBI6	Guanine nucleotide-binding protein G(I)/G(S)/G(O) subunit gamma-12 OS=Homo sapiens OX=9606 GN=GNG12 PE=1 SV=3	-0,057086093	0,250735
Q8NEU8	DCC-interacting protein 13-beta OS=Homo sapiens OX=9606 GN=APPL2 PE=1 SV=3	-0,033853055	0,25026
P42356	Phosphatidylinositol 4-kinase alpha OS=Homo sapiens OX=9606 GN=PI4KA PE=1 SV=4	-0,047096532	0,250004
Q9HB40	Retinoid-inducible serine carboxypeptidase OS=Homo sapiens OX=9606 GN=SCPEP1 PE=1 SV=1	-0,107002339	0,249305
O00232	26S proteasome non-ATPase regulatory subunit 12 OS=Homo sapiens OX=9606 GN=PSMD12 PE=1 SV=3	0,026307717	0,249262
O43929	Origin recognition complex subunit 4 OS=Homo sapiens OX=9606 GN=ORC4 PE=1 SV=2	0,046937682	0,249099
Q9Y2Z0	Protein SGT1 homolog OS=Homo sapiens OX=9606 GN=SUGT1 PE=1 SV=3	0,030246552	0,249053
Q96DG6	Carboxymethylenebutenolidase homolog OS=Homo sapiens OX=9606 GN=CMBL PE=1 SV=1	0,046929426	0,249052
Q96CN7	Isochorismatase domain-containing protein 1 OS=Homo sapiens OX=9606 GN=ISOC1 PE=1 SV=3	-0,077893632	0,248908
Q9BSY9	Deubiquitinase DESI2 OS=Homo sapiens OX=9606 GN=DESI2 PE=1 SV=1	0,056356979	0,247307
Q9NXR1	Nuclear distribution protein nudE homolog 1 OS=Homo sapiens OX=9606 GN=NDE1 PE=1 SV=3	0,056326813	0,247165
P27824	Calnexin OS=Homo sapiens OX=9606 GN=CANX PE=1 SV=2	-0,020109296	0,247149
Q9H9Q4	Non-homologous end-joining factor 1 OS=Homo sapiens OX=9606 GN=NHEJ1 PE=1 SV=1	0,10604625	0,246974
O00762	Ubiquitin-conjugating enzyme E2 C OS=Homo sapiens OX=9606 GN=UBE2C PE=1 SV=1	-0,043169998	0,24624
Q9Y5Q9	General transcription factor 3C polypeptide 3 OS=Homo sapiens OX=9606 GN=GTF3C3 PE=1 SV=1	-0,050569594	0,246144
Q15057	Arf-GAP with coiled-coil, ANK repeat and PH domain-containing protein 2 OS=Homo sapiens OX=9606 GN=ACAP2 PE=1 SV	0,056085586	0,246032
Q9H013	Disintegrin and metalloproteinase domain-containing protein 19 OS=Homo sapiens OX=9606 GN=ADAM19 PE=1 SV=3	0,091065291	0,245736
Q9Y3M8	StAR-related lipid transfer protein 13 OS=Homo sapiens OX=9606 GN=STARD13 PE=1 SV=2	-0,076957475	0,245727
Q92542	Nicastrin OS=Homo sapiens OX=9606 GN=NCSTN PE=1 SV=2	-0,040415472	0,245601
Q92888	Rho guanine nucleotide exchange factor 1 OS=Homo sapiens OX=9606 GN=ARHGEF1 PE=1 SV=2	0,02237655	0,245369
P61020	Ras-related protein Rab-5B OS=Homo sapiens OX=9606 GN=RAB5B PE=1 SV=1	-0,063830259	0,245166
P24666	Low molecular weight phosphotyrosine protein phosphatase OS=Homo sapiens OX=9606 GN=ACP1 PE=1 SV=3	0,046080924	0,244226
P30533	Alpha-2-macroglobulin receptor-associated protein OS=Homo sapiens OX=9606 GN=LRPAP1 PE=1 SV=1	-0,033073917	0,244075
P50148	Guanine nucleotide-binding protein G(q) subunit alpha OS=Homo sapiens OX=9606 GN=GNAQ PE=1 SV=4	-0,034474052	0,243938
P15531	Nucleoside diphosphate kinase A OS=Homo sapiens OX=9606 GN=NME1 PE=1 SV=1	-0,040156628	0,243913
Q8NBS9	Thioredoxin domain-containing protein 5 OS=Homo sapiens OX=9606 GN=TXNDC5 PE=1 SV=2	-0,02639483	0,243495
P26599	Polypyrimidine tract-binding protein 1 OS=Homo sapiens OX=9606 GN=PTBP1 PE=1 SV=1	-0,028682433	0,243311
Q92508	Piezo-type mechanosensitive ion channel component 1 OS=Homo sapiens OX=9606 GN=PIEZO1 PE=1 SV=4	-0,06329017	0,242951
P14927	Cytochrome b-c1 complex subunit 7 OS=Homo sapiens OX=9606 GN=UQCRB PE=1 SV=2	-0,05539752	0,242804
P12081	Histidine--tRNA ligase, cytoplasmic OS=Homo sapiens OX=9606 GN=HARS PE=1 SV=2	0,024494781	0,242721
A0A0B4J2D5	Glutamine amidotransferase-like class 1 domain-containing protein 3B, mitochondrial OS=Homo sapiens OX=9606 GN=G/	-0,039882603	0,242128
Q9P2D3	HEAT repeat-containing protein 5B OS=Homo sapiens OX=9606 GN=HEATR5B PE=1 SV=2	0,055230531	0,242021
P54105	Methylosome subunit pICln OS=Homo sapiens OX=9606 GN=CLNS1A PE=1 SV=1	0,055202801	0,241891
P62140	Serine/threonine-protein phosphatase PP1-beta catalytic subunit OS=Homo sapiens OX=9606 GN=PPP1CB PE=1 SV=3	0,049590535	0,241044
Q6IBS0	Twinfilin-2 OS=Homo sapiens OX=9606 GN=TWF2 PE=1 SV=2	-0,031261775	0,239499
Q9UJX4	Anaphase-promoting complex subunit 5 OS=Homo sapiens OX=9606 GN=ANAPC5 PE=1 SV=2	-0,102929367	0,239382
Q15059	Bromodomain-containing protein 3 OS=Homo sapiens OX=9606 GN=BRD3 PE=1 SV=1	0,074939983	0,238886
O60313	Dynamin-like 120 kDa protein, mitochondrial OS=Homo sapiens OX=9606 GN=OPA1 PE=1 SV=3	-0,019471786	0,238739
P51553	Isocitrate dehydrogenase [NAD] subunit gamma, mitochondrial OS=Homo sapiens OX=9606 GN=IDH3G PE=1 SV=1	0,04912215	0,238608
O75127	Pentatricopeptide repeat-containing protein 1, mitochondrial OS=Homo sapiens OX=9606 GN=PTCD1 PE=1 SV=2	0,102402034	0,238099
P36873	Serine/threonine-protein phosphatase PP1-gamma catalytic subunit OS=Homo sapiens OX=9606 GN=PPP1CC PE=1 SV=1	-0,054157867	0,237
P60709	Actin, cytoplasmic 1 OS=Homo sapiens OX=9606 GN=ACTB PE=1 SV=1	0,016620172	0,236454
P00492	Hypoxanthine-guanine phosphoribosyltransferase OS=Homo sapiens OX=9606 GN=HPRT1 PE=1 SV=2	0,036839204	0,236206

O60678	Protein arginine N-methyltransferase 3 OS=Homo sapiens OX=9606 GN=PRMT3 PE=1 SV=4	-0,038902883	0,23576
P63272	Transcription elongation factor SPT4 OS=Homo sapiens OX=9606 GN=SUPT4H1 PE=1 SV=1	0,073596395	0,234341
Q9Y4F3	Meiosis regulator and mRNA stability factor 1 OS=Homo sapiens OX=9606 GN=MARF1 PE=1 SV=6	-0,049355046	0,233959
P20073	Annexin A7 OS=Homo sapiens OX=9606 GN=ANXA7 PE=1 SV=3	-0,026027544	0,233161
Q9UPN6	SR-related and CTD-associated factor 8 OS=Homo sapiens OX=9606 GN=SCAF8 PE=1 SV=1	-0,047939008	0,232469
Q04941	Proteolipid protein 2 OS=Homo sapiens OX=9606 GN=PLP2 PE=1 SV=1	0,100040093	0,232356
Q92499	ATP-dependent RNA helicase DDX1 OS=Homo sapiens OX=9606 GN=DDX1 PE=1 SV=2	-0,018449989	0,232248
Q9BWJ5	Splicing factor 3B subunit 5 OS=Homo sapiens OX=9606 GN=SF3B5 PE=1 SV=1	0,060613819	0,232011
P48444	Coatomer subunit delta OS=Homo sapiens OX=9606 GN=ARCN1 PE=1 SV=1	-0,023478362	0,231932
P05388	60S acidic ribosomal protein P0 OS=Homo sapiens OX=9606 GN=RPLP0 PE=1 SV=1	-0,029258347	0,231843
O95197	Reticulon-3 OS=Homo sapiens OX=9606 GN=RTN3 PE=1 SV=2	-0,099650576	0,23141
Q9BQE3	Tubulin alpha-1C chain OS=Homo sapiens OX=9606 GN=TUBA1C PE=1 SV=1	0,032812301	0,231355
P15954	Cytochrome c oxidase subunit 7C, mitochondrial OS=Homo sapiens OX=9606 GN=COX7C PE=1 SV=1	-0,072650845	0,231147
P47813	#N/D	0,052690065	0,23015
P35813	Protein phosphatase 1A OS=Homo sapiens OX=9606 GN=PPM1A PE=1 SV=1	-0,052611872	0,229786
P82673	28S ribosomal protein S35, mitochondrial OS=Homo sapiens OX=9606 GN=MRPS35 PE=1 SV=1	0,052488483	0,229211
Q8WVC0	RNA polymerase-associated protein LEO1 OS=Homo sapiens OX=9606 GN=LEO1 PE=1 SV=1	0,071867086	0,228504
Q96GK7	Fumarylacetoacetate hydrolase domain-containing protein 2A OS=Homo sapiens OX=9606 GN=FAHD2A PE=1 SV=1	-0,047081985	0,228034
Q9UJ83	2-hydroxyacyl-CoA lyase 1 OS=Homo sapiens OX=9606 GN=HACL1 PE=1 SV=2	0,059609985	0,227923
P31937	3-hydroxyisobutyrate dehydrogenase, mitochondrial OS=Homo sapiens OX=9606 GN=HIBADH PE=1 SV=2	0,035587707	0,227629
P47985	Cytochrome b-c1 complex subunit Rieske, mitochondrial OS=Homo sapiens OX=9606 GN=UQCRFS1 PE=1 SV=2	-0,047128344	0,227355
Q6I9Y2	THO complex subunit 7 homolog OS=Homo sapiens OX=9606 GN=THOC7 PE=1 SV=3	0,0715027	0,227275
Q96B54	Zinc finger protein 428 OS=Homo sapiens OX=9606 GN=ZNF428 PE=1 SV=2	-0,071259943	0,226458
Q9UBB4	Ataxin-10 OS=Homo sapiens OX=9606 GN=ATXN10 PE=1 SV=1	-0,033628819	0,226151
Q8IXH7	Negative elongation factor C/D OS=Homo sapiens OX=9606 GN=NELFCD PE=1 SV=2	0,042879373	0,22612
P78356	Phosphatidylinositol 5-phosphate 4-kinase type-2 beta OS=Homo sapiens OX=9606 GN=PIP4K2B PE=1 SV=1	-0,097419361	0,225995
Q32P44	Echinoderm microtubule-associated protein-like 3 OS=Homo sapiens OX=9606 GN=EML3 PE=1 SV=1	-0,097032025	0,225055
Q9UHR5	SAP30-binding protein OS=Homo sapiens OX=9606 GN=SAP30BP PE=1 SV=1	-0,05139278	0,224115
Q96EM0	Trans-3-hydroxy-L-proline dehydratase OS=Homo sapiens OX=9606 GN=L3HYPDH PE=1 SV=2	-0,096553839	0,223896
Q8WU79	Stromal membrane-associated protein 2 OS=Homo sapiens OX=9606 GN=SMAP2 PE=1 SV=1	-0,150237624	0,223803
Q15528	Mediator of RNA polymerase II transcription subunit 22 OS=Homo sapiens OX=9606 GN=MED22 PE=1 SV=2	0,096096389	0,222787
O75781	Paralemmin-1 OS=Homo sapiens OX=9606 GN=PALM PE=1 SV=2	-0,033148811	0,222704
Q5F1R6	DnaJ homolog subfamily C member 21 OS=Homo sapiens OX=9606 GN=DNAJC21 PE=1 SV=2	0,058260896	0,22244
Q8NDX5	Polyhomeotic-like protein 3 OS=Homo sapiens OX=9606 GN=PHC3 PE=1 SV=1	-0,070058828	0,222415
Q9H1I8	Activating signal cointegrator 1 complex subunit 2 OS=Homo sapiens OX=9606 GN=ASCC2 PE=1 SV=3	0,058167949	0,222063
Q9BYM8	RanBP-type and C3HC4-type zinc finger-containing protein 1 OS=Homo sapiens OX=9606 GN=RBCK1 PE=1 SV=2	0,095516473	0,221382
Q8N4C8	Misshapen-like kinase 1 OS=Homo sapiens OX=9606 GN=MINK1 PE=1 SV=2	0,045740391	0,221112
Q96DA6	Mitochondrial import inner membrane translocase subunit TIM14 OS=Homo sapiens OX=9606 GN=DNAJC19 PE=1 SV=3	0,187582937	0,22093
Q9NWV8	BRISC and BRCA1-A complex member 1 OS=Homo sapiens OX=9606 GN=BABAM1 PE=1 SV=1	-0,050702276	0,22091
P23526	Adenosylhomocysteinase OS=Homo sapiens OX=9606 GN=AHCY PE=1 SV=4	-0,024087178	0,220763
Q7Z2W9	39S ribosomal protein L21, mitochondrial OS=Homo sapiens OX=9606 GN=MRPL21 PE=1 SV=2	0,095126527	0,220438
P28072	Proteasome subunit beta type-6 OS=Homo sapiens OX=9606 GN=PSMB6 PE=1 SV=4	-0,038862087	0,220051
Q16795	NADH dehydrogenase [ubiquinone] 1 alpha subcomplex subunit 9, mitochondrial OS=Homo sapiens OX=9606 GN=NDUFA	-0,038796279	0,219653
P61970	Nuclear transport factor 2 OS=Homo sapiens OX=9606 GN=NUTF2 PE=1 SV=1	0,050329488	0,219182
Q9Y2R0	Cytochrome c oxidase assembly factor 3 homolog, mitochondrial OS=Homo sapiens OX=9606 GN=COA3 PE=1 SV=1	0,094444137	0,218785
Q8IWP9	Coiled-coil domain-containing protein 28A OS=Homo sapiens OX=9606 GN=CCDC28A PE=1 SV=1	0,094066052	0,21787
Q9UPT5	Exocyst complex component 7 OS=Homo sapiens OX=9606 GN=EXOC7 PE=1 SV=3	0,028541541	0,217275
O75157	TSC22 domain family protein 2 OS=Homo sapiens OX=9606 GN=TSC22D2 PE=1 SV=3	-0,038440733	0,217035
Q9NZJ4	Sacsin OS=Homo sapiens OX=9606 GN=SACS PE=1 SV=2	-0,024994689	0,216725
Q9BYT8	Neurolysin, mitochondrial OS=Homo sapiens OX=9606 GN=NLN PE=1 SV=1	-0,027349372	0,215715
P03915	NADH-ubiquinone oxidoreductase chain 5 OS=Homo sapiens OX=9606 GN=MT-ND5 PE=1 SV=2	-0,049544695	0,215549
Q02978	Mitochondrial 2-oxoglutarate/malate carrier protein OS=Homo sapiens OX=9606 GN=SLC25A11 PE=1 SV=3	0,032143592	0,215506
Q86U86	Protein polybromo-1 OS=Homo sapiens OX=9606 GN=PBRM1 PE=1 SV=1	-0,076083682	0,215346
O75531	Barrier-to-autointegration factor OS=Homo sapiens OX=9606 GN=BANF1 PE=1 SV=1	0,044583162	0,215161
Q96RL1	BRCA1-A complex subunit RAP80 OS=Homo sapiens OX=9606 GN=UIMC1 PE=1 SV=2	-0,135002968	0,215131
Q9NV31	U3 small nucleolar ribonucleoprotein protein IMP3 OS=Homo sapiens OX=9606 GN=IMP3 PE=1 SV=1	-0,092856231	0,214944
Q9H6S0	3'-5' RNA helicase YTHDC2 OS=Homo sapiens OX=9606 GN=YTHDC2 PE=1 SV=2	0,040839812	0,214674
Q53FT3	Protein Hikeshi OS=Homo sapiens OX=9606 GN=HIKESHI PE=1 SV=2	0,049118422	0,213578
Q8IYI6	Exocyst complex component 8 OS=Homo sapiens OX=9606 GN=EXOC8 PE=1 SV=2	0,049108746	0,213533
O00231	26S proteasome non-ATPase regulatory subunit 11 OS=Homo sapiens OX=9606 GN=PSMD11 PE=1 SV=3	0,019981607	0,213086
P63096	Guanine nucleotide-binding protein G(i) subunit alpha-1 OS=Homo sapiens OX=9606 GN=GNAI1 PE=1 SV=2	0,067263814	0,213037
Q9Y282	Endoplasmic reticulum-Golgi intermediate compartment protein 3 OS=Homo sapiens OX=9606 GN=ERGIC3 PE=1 SV=1	0,055937855	0,213033
O60333	Kinesin-like protein KIF1B OS=Homo sapiens OX=9606 GN=KIF1B PE=1 SV=5	-0,092028591	0,212943
O75438	NADH dehydrogenase [ubiquinone] 1 beta subcomplex subunit 1 OS=Homo sapiens OX=9606 GN=NDUFB1 PE=1 SV=1	0,067208116	0,21285
Q9BTA9	WW domain-containing adapter protein with coiled-coil OS=Homo sapiens OX=9606 GN=WAC PE=1 SV=3	-0,067120483	0,212557
Q8NB16	Mixed lineage kinase domain-like protein OS=Homo sapiens OX=9606 GN=MLKL PE=1 SV=1	0,091808912	0,212412
O00391	Sulfhydryl oxidase 1 OS=Homo sapiens OX=9606 GN=QSOX1 PE=1 SV=3	0,066701535	0,211155
P09497	Clathrin light chain B OS=Homo sapiens OX=9606 GN=CLTB PE=1 SV=1	0,037213568	0,210123
Q9H4M3	F-box only protein 44 OS=Homo sapiens OX=9606 GN=FBXO44 PE=1 SV=3	-0,090620022	0,20954
Q5PRF9	Protein Smaug homolog 2 OS=Homo sapiens OX=9606 GN=SAMD4B PE=1 SV=1	0,04799269	0,208383
O95785	Protein Wiz OS=Homo sapiens OX=9606 GN=WIZ PE=1 SV=2	-0,089927136	0,207867
Q9Y3Q8	TSC22 domain family protein 4 OS=Homo sapiens OX=9606 GN=TSC22D4 PE=1 SV=2	-0,065642516	0,207614
Q9BTU6	Phosphatidylinositol 4-kinase type 2-alpha OS=Homo sapiens OX=9606 GN=PI4K2A PE=1 SV=1	-0,031005764	0,207393
Q9Y5Y2	Cytosolic Fe-S cluster assembly factor NUBP2 OS=Homo sapiens OX=9606 GN=NUBP2 PE=1 SV=1	-0,03670947	0,207098
Q8IZH2	5'-3' exoribonuclease 1 OS=Homo sapiens OX=9606 GN=XRN1 PE=1 SV=1	-0,036699105	0,207036
Q9Y4K4	Mitogen-activated protein kinase kinase kinase kinase 5 OS=Homo sapiens OX=9606 GN=MAP4K5 PE=1 SV=2	-0,089361931	0,206504
O60673	DNA polymerase zeta catalytic subunit OS=Homo sapiens OX=9606 GN=REV3L PE=1 SV=2	0,089087106	0,205841
P40261	Nicotinamide N-methyltransferase OS=Homo sapiens OX=9606 GN=NNMT PE=1 SV=1	0,034250998	0,205812
Q02040	A-kinase anchor protein 17A OS=Homo sapiens OX=9606 GN=AKAP17A PE=1 SV=2	-0,054115524	0,205684
Q7RTV5	Peroxiredoxin-like 2C OS=Homo sapiens OX=9606 GN=PRXL2C PE=2 SV=1	-0,096603549	0,205655
Q96G28	Cilia- and flagella-associated protein 36 OS=Homo sapiens OX=9606 GN=CFAP36 PE=1 SV=2	0,032298645	0,205275
Q9UFW8	CGG triplet repeat-binding protein 1 OS=Homo sapiens OX=9606 GN=CGGBP1 PE=1 SV=2	-0,047258472	0,205003
O43414	ERI1 exoribonuclease 3 OS=Homo sapiens OX=9606 GN=ERI3 PE=1 SV=2	-0,047200656	0,204737
O43684	Mitotic checkpoint protein BUB3 OS=Homo sapiens OX=9606 GN=BUB3 PE=1 SV=1	-0,029133281	0,203796
P56537	Eukaryotic translation initiation factor 6 OS=Homo sapiens OX=9606 GN=EIF6 PE=1 SV=1	0,033671766	0,202116
Q9BZ95	Histone-lysine N-methyltransferase NSD3 OS=Homo sapiens OX=9606 GN=NSD3 PE=1 SV=1	-0,063906291	0,201822
O43598	2'-deoxynucleoside 5'-phosphate N-hydrolase 1 OS=Homo sapiens OX=9606 GN=DNPH1 PE=1 SV=1	-0,063511435	0,200506
Q9UBV7	Beta-1,4-galactosyltransferase 7 OS=Homo sapiens OX=9606 GN=B4GALT7 PE=1 SV=1	-0,086630183	0,19992
Q8NET4	Retrotransposon Gag-like protein 9 OS=Homo sapiens OX=9606 GN=RTL9 PE=1 SV=1	-0,086485942	0,199573
Q96F86	Enhancer of mRNA-decapping protein 3 OS=Homo sapiens OX=9606 GN=EDC3 PE=1 SV=1	0,045774394	0,198187
Q9NVG8	TBC1 domain family member 13 OS=Homo sapiens OX=9606 GN=TBC1D13 PE=1 SV=3	-0,041249107	0,198117

Q9NUL5	Repressor of yield of DENV protein OS=Homo sapiens OX=9606 GN=RYDEN PE=1 SV=2	-0,089670539	0,198028
Q96CN9	GRIP and coiled-coil domain-containing protein 1 OS=Homo sapiens OX=9606 GN=GCC1 PE=1 SV=1	0,085656885	0,197578
P50995	Annexin A11 OS=Homo sapiens OX=9606 GN=ANXA11 PE=1 SV=1	-0,025174729	0,197514
P80303	Nucleobindin-2 OS=Homo sapiens OX=9606 GN=NUCB2 PE=1 SV=3	-0,023547398	0,197105
Q9Y333	U6 snRNA-associated Sm-like protein LSm2 OS=Homo sapiens OX=9606 GN=LSM2 PE=1 SV=1	-0,05191478	0,196844
Q9NQW6	Anillin OS=Homo sapiens OX=9606 GN=ANLN PE=1 SV=2	0,017284089	0,196819
O75909	Cyclin-K OS=Homo sapiens OX=9606 GN=CCNK PE=1 SV=2	0,051706513	0,196009
Q96B97	SH3 domain-containing kinase-binding protein 1 OS=Homo sapiens OX=9606 GN=SH3KBP1 PE=1 SV=2	-0,02933564	0,195548
Q9H5K3	Protein O-mannose kinase OS=Homo sapiens OX=9606 GN=POMK PE=1 SV=1	0,084750313	0,195397
Q5JTJ3	Cytochrome c oxidase assembly factor 6 homolog OS=Homo sapiens OX=9606 GN=COA6 PE=1 SV=1	0,08442723	0,19462
Q9UHY1	Nuclear receptor-binding protein OS=Homo sapiens OX=9606 GN=NRBP1 PE=1 SV=1	-0,034568655	0,194305
P06132	Uroporphyrinogen decarboxylase OS=Homo sapiens OX=9606 GN=UROD PE=1 SV=2	-0,026545494	0,193044
P11310	Medium-chain specific acyl-CoA dehydrogenase, mitochondrial OS=Homo sapiens OX=9606 GN=ACADM PE=1 SV=1	0,04017137	0,19264
Q6PIJ6	F-box only protein 38 OS=Homo sapiens OX=9606 GN=FBXO38 PE=1 SV=3	0,083510542	0,192418
Q9H6Y2	WD repeat-containing protein 55 OS=Homo sapiens OX=9606 GN=WDR55 PE=1 SV=2	-0,044147346	0,190743
P67775	Serine/threonine-protein phosphatase 2A catalytic subunit alpha isoform OS=Homo sapiens OX=9606 GN=PPP2CA PE=1 S	-0,082779701	0,190663
Q8WUY8	N-acetyltransferase 14 OS=Homo sapiens OX=9606 GN=NAT14 PE=1 SV=1	0,082747467	0,190585
Q16698	2,4-dienoyl-CoA reductase, mitochondrial OS=Homo sapiens OX=9606 GN=DECR1 PE=1 SV=1	-0,036478968	0,190429
Q13546	Receptor-interacting serine/threonine-protein kinase 1 OS=Homo sapiens OX=9606 GN=RIPK1 PE=1 SV=3	-0,036442354	0,190227
P07741	Adenine phosphoribosyltransferase OS=Homo sapiens OX=9606 GN=APRT PE=1 SV=2	-0,029992428	0,189761
P55081	Microfibrillar-associated protein 1 OS=Homo sapiens OX=9606 GN=MFAP1 PE=1 SV=2	-0,039497285	0,189222
Q7L2H7	Eukaryotic translation initiation factor 3 subunit M OS=Homo sapiens OX=9606 GN=EIF3M PE=1 SV=1	0,024125167	0,188795
P43487	Ran-specific GTPase-activating protein OS=Homo sapiens OX=9606 GN=RANBP1 PE=1 SV=1	-0,033591093	0,188492
P62333	26S proteasome regulatory subunit 10B OS=Homo sapiens OX=9606 GN=PSMC6 PE=1 SV=1	-0,021298128	0,188319
P04049	RAF proto-oncogene serine/threonine-protein kinase OS=Homo sapiens OX=9606 GN=RAF1 PE=1 SV=1	-0,043527951	0,187916
Q9NQR4	Omega-amidase NIT2 OS=Homo sapiens OX=9606 GN=NIT2 PE=1 SV=1	0,024001025	0,187766
Q15814	Tubulin-specific chaperone C OS=Homo sapiens OX=9606 GN=TBCC PE=1 SV=2	-0,043478145	0,187689
O43310	CBP80/20-dependent translation initiation factor OS=Homo sapiens OX=9606 GN=CTIF PE=1 SV=1	-0,043429842	0,187469
Q8IY21	Probable ATP-dependent RNA helicase DDX60 OS=Homo sapiens OX=9606 GN=DDX60 PE=1 SV=3	-0,219395188	0,187399
Q53H96	Pyrroline-5-carboxylate reductase 3 OS=Homo sapiens OX=9606 GN=PYCR3 PE=1 SV=3	-0,081272267	0,187046
P51946	Cyclin-H OS=Homo sapiens OX=9606 GN=CCNH PE=1 SV=1	-0,059432018	0,186964
P08574	Cytochrome c1, heme protein, mitochondrial OS=Homo sapiens OX=9606 GN=CYC1 PE=1 SV=3	0,029318232	0,185251
Q5JRX3	Presequence protease, mitochondrial OS=Homo sapiens OX=9606 GN=PITRM1 PE=1 SV=3	-0,018206214	0,185139
Q53FA7	Quinone oxidoreductase PIG3 OS=Homo sapiens OX=9606 GN=TP53I3 PE=1 SV=2	0,029241388	0,184737
Q14165	Malectin OS=Homo sapiens OX=9606 GN=MLEC PE=1 SV=1	-0,027787827	0,18464
Q9BW92	Threonine--tRNA ligase, mitochondrial OS=Homo sapiens OX=9606 GN=TARS2 PE=1 SV=1	-0,058681214	0,184481
Q9BUB7	Transmembrane protein 70, mitochondrial OS=Homo sapiens OX=9606 GN=TMEM70 PE=1 SV=2	-0,058656673	0,1844
Q71UM5	40S ribosomal protein S27-like OS=Homo sapiens OX=9606 GN=RPS27L PE=1 SV=3	-0,058648261	0,184372
P51608	Methyl-CpG-binding protein 2 OS=Homo sapiens OX=9606 GN=MECP2 PE=1 SV=1	0,08003758	0,184086
O00139	Kinesin-like protein KIF2A OS=Homo sapiens OX=9606 GN=KIF2A PE=1 SV=3	-0,020816063	0,183811
P60866	40S ribosomal protein S20 OS=Homo sapiens OX=9606 GN=RPS20 PE=1 SV=1	-0,04836545	0,182669
Q5RKV6	Exosome complex component MTR3 OS=Homo sapiens OX=9606 GN=EXOSC6 PE=1 SV=1	0,048359146	0,182643
O75821	Eukaryotic translation initiation factor 3 subunit G OS=Homo sapiens OX=9606 GN=EIF3G PE=1 SV=2	0,022603126	0,182571
Q8WTT2	Nucleolar complex protein 3 homolog OS=Homo sapiens OX=9606 GN=NOC3L PE=1 SV=1	-0,034960755	0,182062
Q86VM9	Zinc finger CCCH domain-containing protein 18 OS=Homo sapiens OX=9606 GN=ZC3H18 PE=1 SV=2	-0,048149375	0,181809
P0DJD0	RANBP2-like and GRIP domain-containing protein 1 OS=Homo sapiens OX=9606 GN=RGPD1 PE=2 SV=1	0,021032194	0,180433
Q9C0E2	Exportin-4 OS=Homo sapiens OX=9606 GN=XPO4 PE=1 SV=2	-0,037735207	0,180318
Q92747	Actin-related protein 2/3 complex subunit 1A OS=Homo sapiens OX=9606 GN=ARPC1A PE=2 SV=2	-0,02593121	0,180143
O94901	SUN domain-containing protein 1 OS=Homo sapiens OX=9606 GN=SUN1 PE=1 SV=3	0,024721033	0,179035
O94826	Mitochondrial import receptor subunit TOM70 OS=Homo sapiens OX=9606 GN=TOMM70 PE=1 SV=1	0,019282163	0,179001
Q96RP9	Elongation factor G, mitochondrial OS=Homo sapiens OX=9606 GN=GFM1 PE=1 SV=2	-0,02146834	0,178727
Q9NRH1	Protein YAE1 homolog OS=Homo sapiens OX=9606 GN=YAE1 PE=1 SV=1	0,056891353	0,178574
Q4VC05	B-cell CLL/lymphoma 7 protein family member A OS=Homo sapiens OX=9606 GN=BCL7A PE=1 SV=1	-0,115460857	0,178484
Q9BSL1	Ubiquitin-associated domain-containing protein 1 OS=Homo sapiens OX=9606 GN=UBAC1 PE=1 SV=1	-0,047285267	0,178375
Q9NRL3	Striatin-4 OS=Homo sapiens OX=9606 GN=STRN4 PE=1 SV=2	-0,077642398	0,178354
Q9P0J7	E3 ubiquitin-protein ligase KCMF1 OS=Homo sapiens OX=9606 GN=KCMF1 PE=1 SV=2	-0,04142057	0,178331
Q92930	Ras-related protein Rab-8B OS=Homo sapiens OX=9606 GN=RAB8B PE=1 SV=2	0,076956412	0,176714
Q92889	DNA repair endonuclease XPF OS=Homo sapiens OX=9606 GN=ERCC4 PE=1 SV=3	0,031160992	0,176435
O14579	Coatomer subunit epsilon OS=Homo sapiens OX=9606 GN=COPE PE=1 SV=3	0,023446195	0,17636
Q8NE86	Calcium uniporter protein, mitochondrial OS=Homo sapiens OX=9606 GN=MCU PE=1 SV=1	-0,056184346	0,176245
Q8NF50	Dedicator of cytokinesis protein 8 OS=Homo sapiens OX=9606 GN=DOCK8 PE=1 SV=3	-0,076652531	0,175988
Q9HD45	Transmembrane 9 superfamily member 3 OS=Homo sapiens OX=9606 GN=TM9SF3 PE=1 SV=2	0,029520735	0,175847
Q8N9T8	Protein KRI1 homolog OS=Homo sapiens OX=9606 GN=KRI1 PE=1 SV=3	-0,046632178	0,175783
P30084	Enoyl-CoA hydratase, mitochondrial OS=Homo sapiens OX=9606 GN=ECHS1 PE=1 SV=4	-0,027835483	0,17537
P04183	Thymidine kinase, cytosolic OS=Homo sapiens OX=9606 GN=TK1 PE=1 SV=2	0,031351913	0,175248
P42336	Phosphatidylinositol 4,5-bisphosphate 3-kinase catalytic subunit alpha isoform OS=Homo sapiens OX=9606 GN=PIK3CA PI	-0,075655636	0,173607
Q9BQA1	Methylosome protein 50 OS=Homo sapiens OX=9606 GN=WDR77 PE=1 SV=1	-0,029143774	0,17348
Q96GM5	SWI/SNF-related matrix-associated actin-dependent regulator of chromatin subfamily D member 1 OS=Homo sapiens OX=	0,040189612	0,172755
Q13952	Nuclear transcription factor Y subunit gamma OS=Homo sapiens OX=9606 GN=NFYC PE=1 SV=3	0,055030002	0,172447
Q96BY6	Dedicator of cytokinesis protein 10 OS=Homo sapiens OX=9606 GN=DOCK10 PE=1 SV=3	-0,045743247	0,172262
Q9P1Z9	Coiled-coil domain-containing protein 180 OS=Homo sapiens OX=9606 GN=CCDC180 PE=2 SV=3	-0,078415552	0,171909
Q9H1B7	Probable E3 ubiquitin-protein ligase IRF2BPL OS=Homo sapiens OX=9606 GN=IRF2BPL PE=1 SV=1	0,074694416	0,171313
Q9BZF1	Oxysterol-binding protein-related protein 8 OS=Homo sapiens OX=9606 GN=OSBPL8 PE=1 SV=3	0,022782243	0,171097
Q969G6	Riboflavin kinase OS=Homo sapiens OX=9606 GN=RFK PE=1 SV=2	0,03977242	0,170869
Q00341	Vigilin OS=Homo sapiens OX=9606 GN=HDLBP PE=1 SV=2	-0,0118198	0,17014
Q99873	Protein arginine N-methyltransferase 1 OS=Homo sapiens OX=9606 GN=PRMT1 PE=1 SV=3	0,018361125	0,169991
Q8IZ73	RNA pseudouridylate synthase domain-containing protein 2 OS=Homo sapiens OX=9606 GN=RPUSD2 PE=1 SV=2	-0,045088567	0,169672
O75146	Huntingtin-interacting protein 1-related protein OS=Homo sapiens OX=9606 GN=HIP1R PE=1 SV=2	0,073897906	0,169414
Q9UHQ9	NADH-cytochrome b5 reductase 1 OS=Homo sapiens OX=9606 GN=CYB5R1 PE=1 SV=1	0,025587862	0,169248
Q8N556	Actin filament-associated protein 1 OS=Homo sapiens OX=9606 GN=AFAP1 PE=1 SV=2	0,039236023	0,168446
Q5JTV8	Torsin-1A-interacting protein 1 OS=Homo sapiens OX=9606 GN=TOR1AIP1 PE=1 SV=2	-0,021646521	0,168372
P09012	U1 small nuclear ribonucleoprotein A OS=Homo sapiens OX=9606 GN=SNRPA PE=1 SV=3	-0,032438928	0,168248
Q03405	Urokinase plasminogen activator surface receptor OS=Homo sapiens OX=9606 GN=PLAUR PE=1 SV=1	-0,030156179	0,168215
Q14689	Disco-interacting protein 2 homolog A OS=Homo sapiens OX=9606 GN=DIP2A PE=1 SV=2	0,117656139	0,168181
O00560	Syntenin-1 OS=Homo sapiens OX=9606 GN=SDCBP PE=1 SV=1	-0,032415002	0,168118
Q9BRQ6	MICOS complex subunit MIC25 OS=Homo sapiens OX=9606 GN=CHCHD6 PE=1 SV=1	-0,073117266	0,167554
Q5VZE5	N-alpha-acetyltransferase 35, NatC auxiliary subunit OS=Homo sapiens OX=9606 GN=NAA35 PE=1 SV=1	-0,053529239	0,167521
O95297	Myelin protein zero-like protein 1 OS=Homo sapiens OX=9606 GN=MPZL1 PE=1 SV=1	-0,044432901	0,167082
Q9UHL4	Dipeptidyl peptidase 2 OS=Homo sapiens OX=9606 GN=DPP7 PE=1 SV=3	-0,025244617	0,166859
P57076	Cilia- and flagella-associated protein 298 OS=Homo sapiens OX=9606 GN=CFAP298 PE=1 SV=1	-0,053123663	0,166192
Q9NUQ3	Gamma-taxilin OS=Homo sapiens OX=9606 GN=TXLNG PE=1 SV=2	0,032020161	0,165965

Q13627	Dual specificity tyrosine-phosphorylation-regulated kinase 1A OS=Homo sapiens OX=9606 GN=DYRK1A PE=1 SV=2	0,053028698	0,165881
P07237	Protein disulfide-isomerase OS=Homo sapiens OX=9606 GN=P4HB PE=1 SV=3	-0,013582759	0,165315
Q9NYP9	Protein Mis18-alpha OS=Homo sapiens OX=9606 GN=MIS18A PE=1 SV=1	-0,071996275	0,164885
P28370	Probable global transcription activator SNF2L1 OS=Homo sapiens OX=9606 GN=SMARCA1 PE=1 SV=2	-0,027728601	0,164623
Q6PJ69	Tripartite motif-containing protein 65 OS=Homo sapiens OX=9606 GN=TRIM65 PE=1 SV=3	-0,058193651	0,163927
O60784	Target of Myb protein 1 OS=Homo sapiens OX=9606 GN=TOM1 PE=1 SV=2	0,043582791	0,163729
Q96JH7	Deubiquitinating protein VCIP135 OS=Homo sapiens OX=9606 GN=VCPIP1 PE=1 SV=2	0,043539504	0,163559
Q9UNN8	Endothelial protein C receptor OS=Homo sapiens OX=9606 GN=PROCR PE=1 SV=1	0,071425778	0,163527
Q96PU5	E3 ubiquitin-protein ligase NEDD4-like OS=Homo sapiens OX=9606 GN=NEDD4L PE=1 SV=2	-0,032025166	0,163436
Q9Y2P8	RNA 3'-terminal phosphate cyclase-like protein OS=Homo sapiens OX=9606 GN=RCL1 PE=1 SV=3	0,043486784	0,163351
O95835	Serine/threonine-protein kinase LATS1 OS=Homo sapiens OX=9606 GN=LATS1 PE=1 SV=1	-0,071346082	0,163338
Q8IZ81	ELMO domain-containing protein 2 OS=Homo sapiens OX=9606 GN=ELMOD2 PE=1 SV=1	-0,052236935	0,163289
O60716	Catenin delta-1 OS=Homo sapiens OX=9606 GN=CTNND1 PE=1 SV=1	-0,012403483	0,163123
P26583	High mobility group protein B2 OS=Homo sapiens OX=9606 GN=HMGB2 PE=1 SV=2	0,027471743	0,163021
Q13137	Calcium-binding and coiled-coil domain-containing protein 2 OS=Homo sapiens OX=9606 GN=CALCOCO2 PE=1 SV=1	-0,034237633	0,162771
Q8NI27	THO complex subunit 2 OS=Homo sapiens OX=9606 GN=THOC2 PE=1 SV=2	0,029112475	0,162099
P38646	Stress-70 protein, mitochondrial OS=Homo sapiens OX=9606 GN=HSPA9 PE=1 SV=2	-0,01277182	0,161708
Q93052	Lipoma-preferred partner OS=Homo sapiens OX=9606 GN=LPP PE=1 SV=1	0,027228903	0,161507
Q15836	Vesicle-associated membrane protein 3 OS=Homo sapiens OX=9606 GN=VAMP3 PE=1 SV=3	-0,037647951	0,161294
Q9UQ80	Proliferation-associated protein 2G4 OS=Homo sapiens OX=9606 GN=PA2G4 PE=1 SV=3	-0,015609614	0,160764
O95777	U6 snRNA-associated Sm-like protein LSm8 OS=Homo sapiens OX=9606 GN=LSM8 PE=1 SV=3	-0,042808687	0,160681
P07108	Acyl-CoA-binding protein OS=Homo sapiens OX=9606 GN=DBI PE=1 SV=2	0,037504352	0,160648
Q8NBI6	Xyloside xylosyltransferase 1 OS=Homo sapiens OX=9606 GN=XXYLT1 PE=1 SV=1	-0,042781626	0,160575
Q15067	Peroxisomal acyl-coenzyme A oxidase 1 OS=Homo sapiens OX=9606 GN=ACOX1 PE=1 SV=3	0,022219892	0,160009
P62328	Thymosin beta-4 OS=Homo sapiens OX=9606 GN=TMSB4X PE=1 SV=2	0,030915678	0,159956
O15144	Actin-related protein 2/3 complex subunit 2 OS=Homo sapiens OX=9606 GN=ARPC2 PE=1 SV=1	0,017724979	0,159535
Q5XKP0	MICOS complex subunit MIC13 OS=Homo sapiens OX=9606 GN=MICOS13 PE=1 SV=1	-0,069714741	0,15946
P57081	tRNA (guanine-N(7)-)-methyltransferase non-catalytic subunit WDR4 OS=Homo sapiens OX=9606 GN=WDR4 PE=1 SV=2	-0,069267797	0,158399
P21359	Neurofibromin OS=Homo sapiens OX=9606 GN=NF1 PE=1 SV=2	-0,022915966	0,158154
Q9Y2D5	A-kinase anchor protein 2 OS=Homo sapiens OX=9606 GN=AKAP2 PE=1 SV=3	0,0185172	0,15778
Q8TE73	Dynein heavy chain 5, axonemal OS=Homo sapiens OX=9606 GN=DNAH5 PE=1 SV=3	0,068950654	0,157646
Q8TC12	Retinol dehydrogenase 11 OS=Homo sapiens OX=9606 GN=RDH11 PE=1 SV=2	0,036698945	0,157033
P36954	DNA-directed RNA polymerase II subunit RPB9 OS=Homo sapiens OX=9606 GN=POLR2I PE=1 SV=1	0,068595363	0,156803
Q9Y2V2	Calcium-regulated heat-stable protein 1 OS=Homo sapiens OX=9606 GN=CARHSP1 PE=1 SV=2	-0,036480523	0,156053
Q14185	Dedicator of cytokinesis protein 1 OS=Homo sapiens OX=9606 GN=DOCK1 PE=1 SV=2	0,036445929	0,155898
O60885	Bromodomain-containing protein 4 OS=Homo sapiens OX=9606 GN=BRD4 PE=1 SV=2	0,068192397	0,155847
Q9Y6J9	TAF6-like RNA polymerase II p300/CBP-associated factor-associated factor 65 kDa subunit 6L OS=Homo sapiens OX=9606	-0,068118854	0,155672
O76071	Probable cytosolic iron-sulfur protein assembly protein CIAO1 OS=Homo sapiens OX=9606 GN=CIAO1 PE=1 SV=1	0,036379772	0,155602
O00566	U3 small nucleolar ribonucleoprotein protein MPP10 OS=Homo sapiens OX=9606 GN=MPHOSPH10 PE=1 SV=2	0,067681264	0,154634
P21912	Succinate dehydrogenase [ubiquinone] iron-sulfur subunit, mitochondrial OS=Homo sapiens OX=9606 GN=SDHB PE=1 SV=	-0,029925394	0,154585
Q8TB03	Uncharacterized protein CXorf38 OS=Homo sapiens OX=9606 GN=CXorf38 PE=1 SV=1	0,053692341	0,154267
Q9Y4P1	Cysteine protease ATG4B OS=Homo sapiens OX=9606 GN=ATG4B PE=1 SV=2	-0,029847944	0,154166
O95376	E3 ubiquitin-protein ligase ARIH2 OS=Homo sapiens OX=9606 GN=ARIH2 PE=1 SV=1	0,025187748	0,154052
O94829	Importin-13 OS=Homo sapiens OX=9606 GN=IPO13 PE=1 SV=3	-0,067396598	0,15396
O95084	Serine protease 23 OS=Homo sapiens OX=9606 GN=PRSS23 PE=1 SV=1	-0,049289901	0,153669
O75356	Ectonucleoside triphosphate diphosphohydrolase 5 OS=Homo sapiens OX=9606 GN=ENTPD5 PE=1 SV=1	-0,220740426	0,153499
Q9BR61	Acyl-CoA-binding domain-containing protein 6 OS=Homo sapiens OX=9606 GN=ACBD6 PE=1 SV=1	0,032356759	0,153404
Q2PZI1	Probable C-mannosyltransferase DPY19L1 OS=Homo sapiens OX=9606 GN=DPY19L1 PE=2 SV=1	-0,040941039	0,153348
Q9H1H9	Kinesin-like protein KIF13A OS=Homo sapiens OX=9606 GN=KIF13A PE=1 SV=2	-0,049177771	0,153304
P61158	Actin-related protein 3 OS=Homo sapiens OX=9606 GN=ACTR3 PE=1 SV=3	0,016612881	0,153017
Q13015	Protein AF1q OS=Homo sapiens OX=9606 GN=MLLT11 PE=1 SV=1	-0,049024193	0,152804
Q9UKK9	ADP-sugar pyrophosphatase OS=Homo sapiens OX=9606 GN=NUDT5 PE=1 SV=1	0,025760651	0,15238
P55084	Trifunctional enzyme subunit beta, mitochondrial OS=Homo sapiens OX=9606 GN=HADHB PE=1 SV=3	-0,016488533	0,151816
Q13838	Spliceosome RNA helicase DDX39B OS=Homo sapiens OX=9606 GN=DDX39B PE=1 SV=1	0,022037568	0,1518
P21796	Voltage-dependent anion-selective channel protein 1 OS=Homo sapiens OX=9606 GN=VDAC1 PE=1 SV=2	0,018372077	0,151711
Q9NXE4	Sphingomyelin phosphodiesterase 4 OS=Homo sapiens OX=9606 GN=SMPD4 PE=1 SV=3	0,029363463	0,151545
Q5T447	E3 ubiquitin-protein ligase HECTD3 OS=Homo sapiens OX=9606 GN=HECTD3 PE=1 SV=1	0,035439281	0,151392
P98160	Basement membrane-specific heparan sulfate proteoglycan core protein OS=Homo sapiens OX=9606 GN=HSPG2 PE=1 SV	0,048442661	0,150913
P84085	ADP-ribosylation factor 5 OS=Homo sapiens OX=9606 GN=ARF5 PE=1 SV=2	0,034979939	0,14934
Q66PJ3	ADP-ribosylation factor-like protein 6-interacting protein 4 OS=Homo sapiens OX=9606 GN=ARL6IP4 PE=1 SV=2	-0,039888533	0,149228
Q49A26	Putative oxidoreductase GLYR1 OS=Homo sapiens OX=9606 GN=GLYR1 PE=1 SV=4	0,031377211	0,148545
Q00765	Receptor expression-enhancing protein 5 OS=Homo sapiens OX=9606 GN=REEP5 PE=1 SV=3	0,031309433	0,14821
Q9H7Z6	Histone acetyltransferase KAT8 OS=Homo sapiens OX=9606 GN=KAT8 PE=1 SV=2	0,064822051	0,147864
Q8WXA9	Splicing regulatory glutamine/lysine-rich protein 1 OS=Homo sapiens OX=9606 GN=SREK1 PE=1 SV=1	-0,034575083	0,147532
Q9HC16	DNA dC->dU-editing enzyme APOBEC-3G OS=Homo sapiens OX=9606 GN=APOBEC3G PE=1 SV=1	-0,015197581	0,14696
Q9UHJ6	Sedoheptulokinase OS=Homo sapiens OX=9606 GN=SHPK PE=1 SV=3	-0,023513372	0,146882
Q9BY77	Polymerase delta-interacting protein 3 OS=Homo sapiens OX=9606 GN=POLDIP3 PE=1 SV=2	0,026435683	0,146509
O60674	Tyrosine-protein kinase JAK2 OS=Homo sapiens OX=9606 GN=JAK2 PE=1 SV=2	0,149225057	0,146473
Q9Y3C6	Peptidyl-prolyl cis-trans isomerase-like 1 OS=Homo sapiens OX=9606 GN=PPIL1 PE=1 SV=1	-0,034232894	0,146006
Q6ZVM7	TOM1-like protein 2 OS=Homo sapiens OX=9606 GN=TOM1L2 PE=1 SV=1	-0,030805603	0,145716
Q6ZMI0	Protein phosphatase 1 regulatory subunit 21 OS=Homo sapiens OX=9606 GN=PPP1R21 PE=1 SV=1	-0,063254979	0,144161
Q12906	Interleukin enhancer-binding factor 3 OS=Homo sapiens OX=9606 GN=ILF3 PE=1 SV=3	-0,01263057	0,143853
Q01968	Inositol polyphosphate 5-phosphatase OCRL-1 OS=Homo sapiens OX=9606 GN=OCRL PE=1 SV=3	-0,03849121	0,143772
P30048	Thioredoxin-dependent peroxide reductase, mitochondrial OS=Homo sapiens OX=9606 GN=PRDX3 PE=1 SV=3	-0,025949964	0,143695
Q4V328	GRIP1-associated protein 1 OS=Homo sapiens OX=9606 GN=GRIPAP1 PE=1 SV=2	0,025912277	0,143476
Q96EY8	Corrinoid adenosyltransferase OS=Homo sapiens OX=9606 GN=MMAB PE=1 SV=1	0,062861509	0,143232
Q9BXW7	Haloacid dehalogenase-like hydrolase domain-containing 5 OS=Homo sapiens OX=9606 GN=HDHD5 PE=1 SV=1	-0,046009865	0,143018
Q07889	Son of sevenless homolog 1 OS=Homo sapiens OX=9606 GN=SOS1 PE=1 SV=1	-0,042343763	0,142732
Q8WYA6	Beta-catenin-like protein 1 OS=Homo sapiens OX=9606 GN=CTNNBL1 PE=1 SV=1	0,025755261	0,142568
P53350	Serine/threonine-protein kinase PLK1 OS=Homo sapiens OX=9606 GN=PLK1 PE=1 SV=1	-0,062548616	0,142494
Q5MNZ6	WD repeat domain phosphoinositide-interacting protein 3 OS=Homo sapiens OX=9606 GN=WDR45B PE=1 SV=2	-0,062453902	0,14227
Q6UX04	Spliceosome-associated protein CWC27 homolog OS=Homo sapiens OX=9606 GN=CWC27 PE=1 SV=1	-0,062371314	0,142076
P20338	Ras-related protein Rab-4A OS=Homo sapiens OX=9606 GN=RAB4A PE=1 SV=3	-0,045634642	0,141803
Q15061	WD repeat-containing protein 43 OS=Homo sapiens OX=9606 GN=WDR43 PE=1 SV=3	0,027535309	0,14169
P14406	Cytochrome c oxidase subunit 7A2, mitochondrial OS=Homo sapiens OX=9606 GN=COX7A2 PE=1 SV=1	-0,061984922	0,141164
Q5BJH7	Protein YIF1B OS=Homo sapiens OX=9606 GN=YIF1B PE=1 SV=1	-0,033087889	0,14091
P62861	40S ribosomal protein S30 OS=Homo sapiens OX=9606 GN=FAU PE=1 SV=1	0,045344992	0,140866
P20936	Ras GTPase-activating protein 1 OS=Homo sapiens OX=9606 GN=RASA1 PE=1 SV=1	-0,032985218	0,140454
Q12955	Ankyrin-3 OS=Homo sapiens OX=9606 GN=ANK3 PE=1 SV=3	0,045085289	0,140026
P23378	Glycine dehydrogenase (decarboxylating), mitochondrial OS=Homo sapiens OX=9606 GN=GLDC PE=1 SV=2	-0,065156734	0,140021
P50895	Basal cell adhesion molecule OS=Homo sapiens OX=9606 GN=BCAM PE=1 SV=2	0,044998181	0,139744

P68402	Platelet-activating factor acetylhydrolase IB subunit beta OS=Homo sapiens OX=9606 GN=PAFAH1B2 PE=1 SV=1	0,027066931	0,139174
P50395	Rab GDP dissociation inhibitor beta OS=Homo sapiens OX=9606 GN=GDI2 PE=1 SV=2	0,014169031	0,139156
Q92828	Coronin-2A OS=Homo sapiens OX=9606 GN=CORO2A PE=1 SV=2	0,061002862	0,138849
P35606	Coatomer subunit beta' OS=Homo sapiens OX=9606 GN=COPB2 PE=1 SV=2	0,011288015	0,138155
Q9NUQ7	Ufm1-specific protease 2 OS=Homo sapiens OX=9606 GN=UFSP2 PE=1 SV=3	-0,023421687	0,13794
O60231	Pre-mRNA-splicing factor ATP-dependent RNA helicase DHX16 OS=Homo sapiens OX=9606 GN=DHX16 PE=1 SV=2	-0,032416305	0,137927
Q9Y487	V-type proton ATPase 116 kDa subunit a isoform 2 OS=Homo sapiens OX=9606 GN=ATP6V0A2 PE=1 SV=2	-0,036834279	0,137324
O14561	Acyl carrier protein, mitochondrial OS=Homo sapiens OX=9606 GN=NDUFAB1 PE=1 SV=3	-0,044086709	0,136799
Q86UY8	5'-nucleotidase domain-containing protein 3 OS=Homo sapiens OX=9606 GN=NT5DC3 PE=1 SV=1	0,035386021	0,136354
Q6P158	Putative ATP-dependent RNA helicase DHX57 OS=Homo sapiens OX=9606 GN=DHX57 PE=1 SV=2	-0,03204322	0,136273
Q86X53	Glutamate-rich protein 1 OS=Homo sapiens OX=9606 GN=ERICH1 PE=1 SV=1	0,031333238	0,136201
O95391	Pre-mRNA-splicing factor SLU7 OS=Homo sapiens OX=9606 GN=SLU7 PE=1 SV=2	-0,059791842	0,135997
Q08379	Golgin subfamily A member 2 OS=Homo sapiens OX=9606 GN=GOLGA2 PE=1 SV=3	0,016370927	0,134474
Q96ER3	Protein SAAL1 OS=Homo sapiens OX=9606 GN=SAAL1 PE=1 SV=2	-0,020405078	0,133516
P61163	Alpha-centractin OS=Homo sapiens OX=9606 GN=ACTR1A PE=1 SV=1	0,014905157	0,133072
O14497	AT-rich interactive domain-containing protein 1A OS=Homo sapiens OX=9606 GN=ARID1A PE=1 SV=3	0,035581059	0,132462
Q8IUR7	Armadillo repeat-containing protein 8 OS=Homo sapiens OX=9606 GN=ARMC8 PE=1 SV=2	0,028018076	0,131985
O60921	Checkpoint protein HUS1 OS=Homo sapiens OX=9606 GN=HUS1 PE=1 SV=1	-0,024766285	0,13196
P58335	Anthrax toxin receptor 2 OS=Homo sapiens OX=9606 GN=ANTXR2 PE=1 SV=5	-0,035396786	0,131748
Q7LGC8	Carbohydrate sulfotransferase 3 OS=Homo sapiens OX=9606 GN=CHST3 PE=1 SV=3	0,042518415	0,131743
P04899	Guanine nucleotide-binding protein G(i) subunit alpha-2 OS=Homo sapiens OX=9606 GN=GNAI2 PE=1 SV=3	-0,014388025	0,131657
Q14651	#N/D	0,035314529	0,13143
Q9H267	Vacuolar protein sorting-associated protein 33B OS=Homo sapiens OX=9606 GN=VPS33B PE=1 SV=2	0,030878212	0,131115
P18074	General transcription and DNA repair factor IIH helicase subunit XPD OS=Homo sapiens OX=9606 GN=ERCC2 PE=1 SV=1	0,03523319	0,131115
Q9Y4Y9	U6 snRNA-associated Sm-like protein LSm5 OS=Homo sapiens OX=9606 GN=LSM5 PE=1 SV=3	0,057701186	0,131082
Q6F5E8	Capping protein, Arp2/3 and myosin-I linker protein 2 OS=Homo sapiens OX=9606 GN=CARMIL2 PE=1 SV=2	0,030852947	0,131004
Q8WUM0	Nuclear pore complex protein Nup133 OS=Homo sapiens OX=9606 GN=NUP133 PE=1 SV=2	0,012339576	0,130773
Q9UNH6	Sorting nexin-7 OS=Homo sapiens OX=9606 GN=SNX7 PE=1 SV=1	-0,057352434	0,130263
P49720	Proteasome subunit beta type-3 OS=Homo sapiens OX=9606 GN=PSMB3 PE=1 SV=2	0,022087475	0,129757
Q9BRJ2	39S ribosomal protein L45, mitochondrial OS=Homo sapiens OX=9606 GN=MRPL45 PE=1 SV=2	-0,02755009	0,12969
Q99961	Endophilin-A2 OS=Homo sapiens OX=9606 GN=SH3GL1 PE=1 SV=1	0,018935786	0,12955
Q5VT52	Regulation of nuclear pre-mRNA domain-containing protein 2 OS=Homo sapiens OX=9606 GN=RPRD2 PE=1 SV=1	-0,022037824	0,129454
O00159	Unconventional myosin-Ic OS=Homo sapiens OX=9606 GN=MYO1C PE=1 SV=4	-0,00883051	0,129275
Q9H269	Vacuolar protein sorting-associated protein 16 homolog OS=Homo sapiens OX=9606 GN=VPS16 PE=1 SV=2	0,023372619	0,128839
Q9H0R6	Glutamyl-tRNA(Gln) amidotransferase subunit A, mitochondrial OS=Homo sapiens OX=9606 GN=QRSL1 PE=1 SV=2	0,056517347	0,128303
P42785	Lysosomal Pro-X carboxypeptidase OS=Homo sapiens OX=9606 GN=PRCP PE=1 SV=1	0,027109277	0,127531
P99999	Cytochrome c OS=Homo sapiens OX=9606 GN=CYCS PE=1 SV=2	-0,023107816	0,12732
Q9Y4W6	AFG3-like protein 2 OS=Homo sapiens OX=9606 GN=AFG3L2 PE=1 SV=2	-0,016017728	0,127224
Q15434	RNA-binding motif, single-stranded-interacting protein 2 OS=Homo sapiens OX=9606 GN=RBMS2 PE=1 SV=1	0,041087773	0,127143
O14613	Cdc42 effector protein 2 OS=Homo sapiens OX=9606 GN=CDC42EP2 PE=1 SV=1	-0,040873533	0,126455
Q9BW60	Elongation of very long chain fatty acids protein 1 OS=Homo sapiens OX=9606 GN=ELOVL1 PE=1 SV=1	-0,055041054	0,124842
P61964	WD repeat-containing protein 5 OS=Homo sapiens OX=9606 GN=WDR5 PE=1 SV=1	-0,026425012	0,124186
Q9UM00	Calcium load-activated calcium channel OS=Homo sapiens OX=9606 GN=TMCO1 PE=1 SV=2	-0,040076466	0,123897
Q5BKZ1	DBIRD complex subunit ZNF326 OS=Homo sapiens OX=9606 GN=ZNF326 PE=1 SV=2	-0,026288951	0,123521
O60271	C-Jun-amino-terminal kinase-interacting protein 4 OS=Homo sapiens OX=9606 GN=SPAG9 PE=1 SV=4	-0,011374361	0,122398
Q9HCE1	Helicase MOV-10 OS=Homo sapiens OX=9606 GN=MOV10 PE=1 SV=2	-0,016521492	0,122243
P30050	60S ribosomal protein L12 OS=Homo sapiens OX=9606 GN=RPL12 PE=1 SV=1	-0,019662651	0,12189
Q9NX14	NADH dehydrogenase [ubiquinone] 1 beta subcomplex subunit 11, mitochondrial OS=Homo sapiens OX=9606 GN=NDUFE	-0,014952009	0,121291
O75674	TOM1-like protein 1 OS=Homo sapiens OX=9606 GN=TOM1L1 PE=1 SV=2	0,032676822	0,121245
Q8IVF7	#N/D	0,053411987	0,121029
P84090	Enhancer of rudimentary homolog OS=Homo sapiens OX=9606 GN=ERH PE=1 SV=1	0,053410329	0,121025
Q6NW29	RWD domain-containing protein 4 OS=Homo sapiens OX=9606 GN=RWDD4 PE=1 SV=3	-0,052997947	0,120061
Q99549	M-phase phosphoprotein 8 OS=Homo sapiens OX=9606 GN=MPHOSPH8 PE=1 SV=2	0,052808602	0,119618
O75477	Erlin-1 OS=Homo sapiens OX=9606 GN=ERLIN1 PE=1 SV=2	-0,019304017	0,119581
O14939	Phospholipase D2 OS=Homo sapiens OX=9606 GN=PLD2 PE=1 SV=2	0,026290818	0,119408
Q9H3P2	Negative elongation factor A OS=Homo sapiens OX=9606 GN=NELFA PE=1 SV=3	0,023314287	0,119148
P25789	Proteasome subunit alpha type-4 OS=Homo sapiens OX=9606 GN=PSMA4 PE=1 SV=1	0,018270919	0,119017
Q7Z4H7	HAUS augmin-like complex subunit 6 OS=Homo sapiens OX=9606 GN=HAUS6 PE=1 SV=2	0,0280709	0,11875
Q8IUW5	RELT-like protein 1 OS=Homo sapiens OX=9606 GN=RELL1 PE=1 SV=1	-0,052205735	0,11821
P06748	Nucleophosmin OS=Homo sapiens OX=9606 GN=NPM1 PE=1 SV=2	0,015939143	0,11777
Q9UPU5	Ubiquitin carboxyl-terminal hydrolase 24 OS=Homo sapiens OX=9606 GN=USP24 PE=1 SV=3	-0,031731086	0,117608
Q6YN16	Hydroxysteroid dehydrogenase-like protein 2 OS=Homo sapiens OX=9606 GN=HSDL2 PE=1 SV=1	-0,031654037	0,117312
Q7L5N7	Lysophosphatidylcholine acyltransferase 2 OS=Homo sapiens OX=9606 GN=LPCAT2 PE=1 SV=1	-0,021239975	0,116612
P49902	Cytosolic purine 5'-nucleotidase OS=Homo sapiens OX=9606 GN=NT5C2 PE=1 SV=1	0,01472359	0,116557
P78332	RNA-binding protein 6 OS=Homo sapiens OX=9606 GN=RBM6 PE=1 SV=5	0,052915531	0,116479
Q8NI38	NF-kappa-B inhibitor delta OS=Homo sapiens OX=9606 GN=NFKBID PE=1 SV=1	0,037656619	0,116192
Q13332	Receptor-type tyrosine-protein phosphatase S OS=Homo sapiens OX=9606 GN=PTPRS PE=1 SV=3	-0,051233914	0,115941
Q9BSV6	tRNA-splicing endonuclease subunit Sen34 OS=Homo sapiens OX=9606 GN=TSEN34 PE=1 SV=1	-0,021057717	0,115606
P23510	Tumor necrosis factor ligand superfamily member 4 OS=Homo sapiens OX=9606 GN=TNFSF4 PE=1 SV=1	0,031166314	0,115439
Q96GQ5	RUS1 family protein C16orf58 OS=Homo sapiens OX=9606 GN=C16orf58 PE=1 SV=2	0,031012985	0,114851
P13995	Bifunctional methylenetetrahydrofolate dehydrogenase/cyclohydrolase, mitochondrial OS=Homo sapiens OX=9606 GN=N	-0,022294212	0,113745
Q96JY6	PDZ and LIM domain protein 2 OS=Homo sapiens OX=9606 GN=PDLIM2 PE=1 SV=1	0,026922564	0,113718
Q12792	Twinfilin-1 OS=Homo sapiens OX=9606 GN=TWF1 PE=1 SV=3	-0,01345747	0,113097
P63173	60S ribosomal protein L38 OS=Homo sapiens OX=9606 GN=RPL38 PE=1 SV=2	0,049992041	0,113045
Q16774	Guanylate kinase OS=Homo sapiens OX=9606 GN=GUK1 PE=1 SV=2	-0,022069453	0,112557
Q9Y4Z0	U6 snRNA-associated Sm-like protein LSm4 OS=Homo sapiens OX=9606 GN=LSM4 PE=1 SV=1	-0,022032122	0,11236
Q6UW68	Transmembrane protein 205 OS=Homo sapiens OX=9606 GN=TMEM205 PE=1 SV=1	-0,049533363	0,111977
Q03111	Protein ENL OS=Homo sapiens OX=9606 GN=MLLT1 PE=1 SV=2	-0,049504785	0,11191
Q92504	Zinc transporter SLC39A7 OS=Homo sapiens OX=9606 GN=SLC39A7 PE=1 SV=2	-0,049478725	0,111849
Q9UQN3	Charged multivesicular body protein 2b OS=Homo sapiens OX=9606 GN=CHMP2B PE=1 SV=1	-0,049265544	0,111353
P51665	26S proteasome non-ATPase regulatory subunit 7 OS=Homo sapiens OX=9606 GN=PSMD7 PE=1 SV=2	0,014541152	0,111134
Q8WXE0	Caskin-2 OS=Homo sapiens OX=9606 GN=CASKIN2 PE=1 SV=2	0,041942082	0,110976
Q9HA65	TBC1 domain family member 17 OS=Homo sapiens OX=9606 GN=TBC1D17 PE=1 SV=2	-0,036031009	0,110972
Q9Y2S6	Translation machinery-associated protein 7 OS=Homo sapiens OX=9606 GN=TMA7 PE=1 SV=1	-0,049023818	0,11079
Q9NUG6	p53 and DNA damage-regulated protein 1 OS=Homo sapiens OX=9606 GN=PDRG1 PE=1 SV=2	0,048904554	0,110513
O14545	TRAF-type zinc finger domain-containing protein 1 OS=Homo sapiens OX=9606 GN=TRAFD1 PE=1 SV=1	0,029877561	0,110501
Q9BVS4	Serine/threonine-protein kinase RIO2 OS=Homo sapiens OX=9606 GN=RIOK2 PE=1 SV=2	-0,020501884	0,109196
P49754	Vacuolar protein sorting-associated protein 41 homolog OS=Homo sapiens OX=9606 GN=VPS41 PE=1 SV=3	0,029525507	0,109154
Q9HB20	Pleckstrin homology domain-containing family A member 3 OS=Homo sapiens OX=9606 GN=PLEKHA3 PE=1 SV=2	-0,048018296	0,108451
P46777	60S ribosomal protein L5 OS=Homo sapiens OX=9606 GN=RPL5 PE=1 SV=3	0,014168915	0,108189
Q9ULV4	Coronin-1C OS=Homo sapiens OX=9606 GN=CORO1C PE=1 SV=1	0,011089039	0,107847

Q9Y608	Leucine-rich repeat flightless-interacting protein 2 OS=Homo sapiens OX=9606 GN=LRRFIP2 PE=1 SV=1	-0,019654469	0,107635
P35613	Basigin OS=Homo sapiens OX=9606 GN=BSG PE=1 SV=2	0,023012316	0,107597
Q9Y6B6	GTP-binding protein SAR1b OS=Homo sapiens OX=9606 GN=SAR1B PE=1 SV=1	0,029036118	0,107284
Q4KMQ2	Anoctamin-6 OS=Homo sapiens OX=9606 GN=ANO6 PE=1 SV=2	-0,022909938	0,107102
Q96P16	Regulation of nuclear pre-mRNA domain-containing protein 1A OS=Homo sapiens OX=9606 GN=RPRD1A PE=1 SV=1	0,028940692	0,106919
Q6NUN0	Acyl-coenzyme A synthetase ACSM5, mitochondrial OS=Homo sapiens OX=9606 GN=ACSM5 PE=1 SV=2	-0,093424326	0,106848
O95563	Mitochondrial pyruvate carrier 2 OS=Homo sapiens OX=9606 GN=MPC2 PE=1 SV=1	0,047237666	0,106636
Q9P2X0	Dolichol-phosphate mannosyltransferase subunit 3 OS=Homo sapiens OX=9606 GN=DPM3 PE=1 SV=2	-0,047116108	0,106354
P51116	Fragile X mental retardation syndrome-related protein 2 OS=Homo sapiens OX=9606 GN=FXR2 PE=1 SV=2	-0,016289689	0,105669
P51151	Ras-related protein Rab-9A OS=Homo sapiens OX=9606 GN=RAB9A PE=1 SV=1	-0,046588902	0,105129
Q5T8D3	Acyl-CoA-binding domain-containing protein 5 OS=Homo sapiens OX=9606 GN=ACBD5 PE=1 SV=1	0,028354805	0,104684
Q14204	Cytoplasmic dynein 1 heavy chain 1 OS=Homo sapiens OX=9606 GN=DYNC1H1 PE=1 SV=5	-0,003536855	0,104582
O94813	Slit homolog 2 protein OS=Homo sapiens OX=9606 GN=SLIT2 PE=1 SV=1	-0,167564819	0,104401
Q9NQ92	Coordinator of PRMT5 and differentiation stimulator OS=Homo sapiens OX=9606 GN=COPRS PE=1 SV=3	0,028259234	0,104319
Q9UBB9	Tuftelin-interacting protein 11 OS=Homo sapiens OX=9606 GN=TFIP11 PE=1 SV=1	-0,018925518	0,103509
P29590	Protein PML OS=Homo sapiens OX=9606 GN=PML PE=1 SV=3	-0,01136954	0,103109
Q04446	1,4-alpha-glucan-branching enzyme OS=Homo sapiens OX=9606 GN=GBE1 PE=1 SV=3	-0,009988635	0,10301
P55209	Nucleosome assembly protein 1-like 1 OS=Homo sapiens OX=9606 GN=NAP1L1 PE=1 SV=1	0,015123665	0,102602
Q9Y3T9	Nucleolar complex protein 2 homolog OS=Homo sapiens OX=9606 GN=NOC2L PE=1 SV=4	0,016624604	0,102433
Q14191	Werner syndrome ATP-dependent helicase OS=Homo sapiens OX=9606 GN=WRN PE=1 SV=2	-0,033270402	0,102204
Q08722	Leukocyte surface antigen CD47 OS=Homo sapiens OX=9606 GN=CD47 PE=1 SV=1	0,045167265	0,101831
P62316	Small nuclear ribonucleoprotein Sm D2 OS=Homo sapiens OX=9606 GN=SNRPD2 PE=1 SV=1	0,014993061	0,101687
O60437	Periplakin OS=Homo sapiens OX=9606 GN=PPL PE=1 SV=4	0,045031194	0,101516
Q7Z7B0	Filamin-A-interacting protein 1 OS=Homo sapiens OX=9606 GN=FILIP1 PE=1 SV=1	-0,045003653	0,101452
Q13188	Serine/threonine-protein kinase 3 OS=Homo sapiens OX=9606 GN=STK3 PE=1 SV=2	0,027256336	0,100499
Q8NBM4	Ubiquitin-associated domain-containing protein 2 OS=Homo sapiens OX=9606 GN=UBAC2 PE=1 SV=1	0,044560075	0,100424
Q9Y696	Chloride intracellular channel protein 4 OS=Homo sapiens OX=9606 GN=CLIC4 PE=1 SV=4	0,011996481	0,100415
O43148	mRNA cap guanine-N7 methyltransferase OS=Homo sapiens OX=9606 GN=RNMT PE=1 SV=1	-0,0196111	0,099616
Q9NZJ6	Ubiquinone biosynthesis O-methyltransferase, mitochondrial OS=Homo sapiens OX=9606 GN=COQ3 PE=1 SV=3	0,014141843	0,09931
P62841	40S ribosomal protein S15 OS=Homo sapiens OX=9606 GN=RPS15 PE=1 SV=2	-0,019547558	0,099283
Q14691	DNA replication complex GINS protein PSF1 OS=Homo sapiens OX=9606 GN=GINS1 PE=1 SV=1	-0,043903723	0,098904
P62854	40S ribosomal protein S26 OS=Homo sapiens OX=9606 GN=RPS26 PE=1 SV=3	-0,02682495	0,098859
P63218	Guanine nucleotide-binding protein G(I)/G(S)/G(O) subunit gamma-5 OS=Homo sapiens OX=9606 GN=GNG5 PE=1 SV=3	0,02662959	0,098117
Q86VP1	Tax1-binding protein 1 OS=Homo sapiens OX=9606 GN=TAX1BP1 PE=1 SV=2	-0,023287313	0,097884
Q96QR8	Transcriptional activator protein Pur-beta OS=Homo sapiens OX=9606 GN=PURB PE=1 SV=3	0,026550321	0,097816
Q9NR28	Diablo homolog, mitochondrial OS=Homo sapiens OX=9606 GN=DIABLO PE=1 SV=1	0,026542343	0,097785
Q93034	Cullin-5 OS=Homo sapiens OX=9606 GN=CUL5 PE=1 SV=4	-0,020954245	0,097672
P26196	Probable ATP-dependent RNA helicase DDX6 OS=Homo sapiens OX=9606 GN=DDX6 PE=1 SV=2	-0,011663104	0,097535
Q9UBQ7	Glyoxylate reductase/hydroxypyruvate reductase OS=Homo sapiens OX=9606 GN=GRHPR PE=1 SV=1	0,015063255	0,09746
Q9Y4C8	Probable RNA-binding protein 19 OS=Homo sapiens OX=9606 GN=RBM19 PE=1 SV=3	-0,043212403	0,097304
O60244	Mediator of RNA polymerase II transcription subunit 14 OS=Homo sapiens OX=9606 GN=MED14 PE=1 SV=2	-0,026394532	0,097224
P23497	Nuclear autoantigen Sp-100 OS=Homo sapiens OX=9606 GN=SP100 PE=1 SV=3	-0,019104076	0,09696
Q68CZ6	HAUS augmin-like complex subunit 3 OS=Homo sapiens OX=9606 GN=HAUS3 PE=1 SV=1	0,031528748	0,096694
Q9Y276	Mitochondrial chaperone BCS1 OS=Homo sapiens OX=9606 GN=BCS1L PE=1 SV=1	0,042859176	0,096487
Q9NVJ2	ADP-ribosylation factor-like protein 8B OS=Homo sapiens OX=9606 GN=ARL8B PE=1 SV=1	-0,031371609	0,096197
P50552	Vasodilator-stimulated phosphoprotein OS=Homo sapiens OX=9606 GN=VASP PE=1 SV=3	-0,011489697	0,096039
Q9Y2L5	Trafficking protein particle complex subunit 8 OS=Homo sapiens OX=9606 GN=TRAPPC8 PE=1 SV=2	-0,031102269	0,095347
Q9UBV8	Peflin OS=Homo sapiens OX=9606 GN=PEF1 PE=1 SV=1	0,030983121	0,094971
Q5VW32	BRO1 domain-containing protein BROX OS=Homo sapiens OX=9606 GN=BROX PE=1 SV=1	-0,020383375	0,09493
Q9NPD3	Exosome complex component RRP41 OS=Homo sapiens OX=9606 GN=EXOSC4 PE=1 SV=3	-0,030854872	0,094567
P40425	Pre-B-cell leukemia transcription factor 2 OS=Homo sapiens OX=9606 GN=PBX2 PE=1 SV=2	-0,041916969	0,09431
O43292	Glycosylphosphatidylinositol anchor attachment 1 protein OS=Homo sapiens OX=9606 GN=GPAA1 PE=1 SV=3	-0,025599974	0,09421
Q8IXI1	Mitochondrial Rho GTPase 2 OS=Homo sapiens OX=9606 GN=RHOT2 PE=1 SV=2	0,030739711	0,094203
P00491	Purine nucleoside phosphorylase OS=Homo sapiens OX=9606 GN=PNP PE=1 SV=2	-0,013348488	0,094185
Q15907	Ras-related protein Rab-11B OS=Homo sapiens OX=9606 GN=RAB11B PE=1 SV=4	-0,016456893	0,094114
P46977	Dolichyl-diphosphooligosaccharide--protein glycosyltransferase subunit STT3A OS=Homo sapiens OX=9606 GN=STT3A PE=	0,013857912	0,093752
Q6RFH5	WD repeat-containing protein 74 OS=Homo sapiens OX=9606 GN=WDR74 PE=1 SV=1	0,022284046	0,09354
O95396	Adenylyltransferase and sulfurtransferase MOCS3 OS=Homo sapiens OX=9606 GN=MOCS3 PE=1 SV=1	0,14521781	0,093447
Q96IK1	#N/D	0,041480027	0,093301
P34059	N-acetylgalactosamine-6-sulfatase OS=Homo sapiens OX=9606 GN=GALNS PE=1 SV=1	0,030186234	0,092458
Q5TA45	Integrator complex subunit 11 OS=Homo sapiens OX=9606 GN=INTS11 PE=1 SV=2	0,021995901	0,092294
Q12800	Alpha-globin transcription factor CP2 OS=Homo sapiens OX=9606 GN=TFCP2 PE=1 SV=2	0,025010557	0,091977
O00214	Galectin-8 OS=Homo sapiens OX=9606 GN=LGALS8 PE=1 SV=4	-0,040899127	0,09196
P42224	Signal transducer and activator of transcription 1-alpha/beta OS=Homo sapiens OX=9606 GN=STAT1 PE=1 SV=2	-0,009447352	0,091401
Q5VZF2	Muscleblind-like protein 2 OS=Homo sapiens OX=9606 GN=MBNL2 PE=1 SV=2	-0,01674136	0,091209
Q9BRP1	Programmed cell death protein 2-like OS=Homo sapiens OX=9606 GN=PDCD2L PE=1 SV=1	0,040567856	0,091196
Q96IJ6	Mannose-1-phosphate guanyltransferase alpha OS=Homo sapiens OX=9606 GN=GMPPA PE=1 SV=1	0,024791381	0,091147
Q9C0D3	Protein zyg-11 homolog B OS=Homo sapiens OX=9606 GN=ZYG11B PE=1 SV=2	-0,0405378	0,091127
Q9H0U4	Ras-related protein Rab-1B OS=Homo sapiens OX=9606 GN=RAB1B PE=1 SV=1	-0,021670482	0,090889
Q9Y3E1	Hepatoma-derived growth factor-related protein 3 OS=Homo sapiens OX=9606 GN=HDGFL3 PE=1 SV=1	-0,024697331	0,090792
Q9H9A6	Leucine-rich repeat-containing protein 40 OS=Homo sapiens OX=9606 GN=LRRC40 PE=1 SV=1	-0,012774727	0,090016
O15121	Sphingolipid delta(4)-desaturase DES1 OS=Homo sapiens OX=9606 GN=DEGS1 PE=1 SV=1	0,024468186	0,089925
P23396	40S ribosomal protein S3 OS=Homo sapiens OX=9606 GN=RPS3 PE=1 SV=2	0,009662489	0,089351
Q8IXT5	RNA-binding protein 12B OS=Homo sapiens OX=9606 GN=RBM12B PE=1 SV=2	0,019210026	0,089307
Q86VP6	Cullin-associated NEDD8-dissociated protein 1 OS=Homo sapiens OX=9606 GN=CAND1 PE=1 SV=2	-0,00627839	0,089002
Q7Z417	Nuclear fragile X mental retardation-interacting protein 2 OS=Homo sapiens OX=9606 GN=NUFIP2 PE=1 SV=1	-0,010626994	0,088618
P25398	40S ribosomal protein S12 OS=Homo sapiens OX=9606 GN=RPS12 PE=1 SV=3	-0,016242795	0,088414
O75592	E3 ubiquitin-protein ligase MYCBP2 OS=Homo sapiens OX=9606 GN=MYCBP2 PE=1 SV=4	-0,029519193	0,087665
Q8WWB7	Glycosylated lysosomal membrane protein OS=Homo sapiens OX=9606 GN=GLMP PE=1 SV=1	0,038950106	0,087469
Q2TAA5	GDP-Man:Man(3)GlcNAc(2)-PP-Dol alpha-1,2-mannosyltransferase OS=Homo sapiens OX=9606 GN=ALG11 PE=1 SV=2	0,038905892	0,087367
Q9BXV9	EKC/KEOPS complex subunit GON7 OS=Homo sapiens OX=9606 GN=GON7 PE=1 SV=2	-0,038717055	0,086932
P21281	V-type proton ATPase subunit B, brain isoform OS=Homo sapiens OX=9606 GN=ATP6V1B2 PE=1 SV=3	0,009604146	0,086642
Q16864	V-type proton ATPase subunit F OS=Homo sapiens OX=9606 GN=ATP6V1F PE=1 SV=2	0,018650141	0,086631
P61769	Beta-2-microglobulin OS=Homo sapiens OX=9606 GN=B2M PE=1 SV=1	0,02819549	0,086197
Q9BY44	Eukaryotic translation initiation factor 2A OS=Homo sapiens OX=9606 GN=EIF2A PE=1 SV=3	0,008917067	0,086125
P0DI81	Trafficking protein particle complex subunit 2 OS=Homo sapiens OX=9606 GN=TRAPPC2 PE=1 SV=1	0,038069876	0,085443
Q9H4I9	Essential MCU regulator, mitochondrial OS=Homo sapiens OX=9606 GN=SMDT1 PE=1 SV=1	-0,037972089	0,085218
Q7Z739	YTH domain-containing family protein 3 OS=Homo sapiens OX=9606 GN=YTHDF3 PE=1 SV=1	-0,01204267	0,084714
Q13523	Serine/threonine-protein kinase PRP4 homolog OS=Homo sapiens OX=9606 GN=PRPF4B PE=1 SV=3	-0,016685687	0,084348
Q15042	Rab3 GTPase-activating protein catalytic subunit OS=Homo sapiens OX=9606 GN=RAB3GAP1 PE=1 SV=3	-0,009842883	0,08428
O43681	ATPase ASNA1 OS=Homo sapiens OX=9606 GN=ASNA1 PE=1 SV=2	0,011435897	0,083586

Q92945	Far upstream element-binding protein 2 OS=Homo sapiens OX=9606 GN=KHSRP PE=1 SV=4	-0,007708956	0,083358
Q9Y2I8	WD repeat-containing protein 37 OS=Homo sapiens OX=9606 GN=WDR37 PE=1 SV=2	0,02721464	0,08312
O95758	Polypyrimidine tract-binding protein 3 OS=Homo sapiens OX=9606 GN=PTBP3 PE=1 SV=2	-0,03703441	0,083064
Q99805	Transmembrane 9 superfamily member 2 OS=Homo sapiens OX=9606 GN=TM9SF2 PE=1 SV=1	-0,013555035	0,083005
P49407	Beta-arrestin-1 OS=Homo sapiens OX=9606 GN=ARRB1 PE=1 SV=2	0,036927548	0,082818
Q9UQ13	Leucine-rich repeat protein SHOC-2 OS=Homo sapiens OX=9606 GN=SHOC2 PE=1 SV=2	0,019688768	0,082356
P49768	Presenilin-1 OS=Homo sapiens OX=9606 GN=PSEN1 PE=1 SV=1	0,037343127	0,08209
Q03169	Tumor necrosis factor alpha-induced protein 2 OS=Homo sapiens OX=9606 GN=TNFAIP2 PE=2 SV=2	0,017692974	0,082065
Q8NCW5	NAD(P)H-hydrate epimerase OS=Homo sapiens OX=9606 GN=NAXE PE=1 SV=2	-0,013340604	0,081657
P30043	Flavin reductase (NADPH) OS=Homo sapiens OX=9606 GN=BLVRB PE=1 SV=3	-0,011567075	0,081279
Q9BUF5	Tubulin beta-6 chain OS=Homo sapiens OX=9606 GN=TUBB6 PE=1 SV=1	-0,012023268	0,081009
Q9BXK1	Krueppel-like factor 16 OS=Homo sapiens OX=9606 GN=KLF16 PE=1 SV=1	-0,019519634	0,080307
P49755	Transmembrane emp24 domain-containing protein 10 OS=Homo sapiens OX=9606 GN=TMED10 PE=1 SV=2	0,019166832	0,080116
O75362	Zinc finger protein 217 OS=Homo sapiens OX=9606 GN=ZNF217 PE=1 SV=1	-0,02186436	0,08011
Q05193	Dynamin-1 OS=Homo sapiens OX=9606 GN=DNM1 PE=1 SV=2	0,006853559	0,080063
Q15853	#N/D	-0,115661803	0,080013
P11387	DNA topoisomerase 1 OS=Homo sapiens OX=9606 GN=TOP1 PE=1 SV=2	-0,008280083	0,07981
Q6UWE0	E3 ubiquitin-protein ligase LRSAM1 OS=Homo sapiens OX=9606 GN=LRSAM1 PE=1 SV=1	-0,013739828	0,079452
O15145	Actin-related protein 2/3 complex subunit 3 OS=Homo sapiens OX=9606 GN=ARPC3 PE=1 SV=3	-0,0156678	0,079069
Q15555	Microtubule-associated protein RP/EB family member 2 OS=Homo sapiens OX=9606 GN=MAPRE2 PE=1 SV=1	0,01867878	0,078024
Q13371	Phosducin-like protein OS=Homo sapiens OX=9606 GN=PDCL PE=1 SV=3	-0,034727754	0,077773
P12931	Proto-oncogene tyrosine-protein kinase Src OS=Homo sapiens OX=9606 GN=SRC PE=1 SV=3	-0,015401442	0,077691
Q05048	Cleavage stimulation factor subunit 1 OS=Homo sapiens OX=9606 GN=CSTF1 PE=1 SV=1	0,012678672	0,077501
P28288	ATP-binding cassette sub-family D member 3 OS=Homo sapiens OX=9606 GN=ABCD3 PE=1 SV=1	-0,011002237	0,077208
Q13613	Myotubularin-related protein 1 OS=Homo sapiens OX=9606 GN=MTMR1 PE=1 SV=4	-0,025257253	0,076996
Q15773	Myeloid leukemia factor 2 OS=Homo sapiens OX=9606 GN=MLF2 PE=1 SV=1	0,034288371	0,076767
O75923	Dysferlin OS=Homo sapiens OX=9606 GN=DYSF PE=1 SV=1	0,014130163	0,076625
P49711	Transcriptional repressor CTCF OS=Homo sapiens OX=9606 GN=CTCF PE=1 SV=1	-0,025067933	0,076405
Q9BRP4	Proteasomal ATPase-associated factor 1 OS=Homo sapiens OX=9606 GN=PAAF1 PE=1 SV=2	0,013968481	0,075726
Q8IXK0	Polyhomeotic-like protein 2 OS=Homo sapiens OX=9606 GN=PHC2 PE=1 SV=1	-0,033828869	0,075715
Q9Y6N1	Cytochrome c oxidase assembly protein COX11, mitochondrial OS=Homo sapiens OX=9606 GN=COX11 PE=1 SV=3	-0,025184455	0,075655
Q9C099	Leucine-rich repeat and coiled-coil domain-containing protein 1 OS=Homo sapiens OX=9606 GN=LRRCC1 PE=1 SV=2	-0,033785023	0,075615
Q16706	Alpha-mannosidase 2 OS=Homo sapiens OX=9606 GN=MAN2A1 PE=1 SV=2	-0,007389337	0,075535
Q96JJ3	Engulfment and cell motility protein 2 OS=Homo sapiens OX=9606 GN=ELMO2 PE=1 SV=2	0,020621264	0,075444
P50402	Emerin OS=Homo sapiens OX=9606 GN=EMD PE=1 SV=1	0,009085535	0,075442
P07195	L-lactate dehydrogenase B chain OS=Homo sapiens OX=9606 GN=LDHB PE=1 SV=2	-0,00932813	0,075199
Q13045	Protein flightless-1 homolog OS=Homo sapiens OX=9606 GN=FLII PE=1 SV=2	-0,005803207	0,075128
P23443	Ribosomal protein S6 kinase beta-1 OS=Homo sapiens OX=9606 GN=RPS6KB1 PE=1 SV=2	-0,02454494	0,074773
P60468	Protein transport protein Sec61 subunit beta OS=Homo sapiens OX=9606 GN=SEC61B PE=1 SV=2	0,024494444	0,074616
Q7Z4W1	L-xylulose reductase OS=Homo sapiens OX=9606 GN=DCXR PE=1 SV=2	-0,012897566	0,074462
Q8NFC6	Biorientation of chromosomes in cell division protein 1-like 1 OS=Homo sapiens OX=9606 GN=BOD1L1 PE=1 SV=2	-0,032608709	0,072925
Q9NY12	H/ACA ribonucleoprotein complex subunit 1 OS=Homo sapiens OX=9606 GN=GAR1 PE=1 SV=1	-0,019891925	0,072713
P62877	E3 ubiquitin-protein ligase RBX1 OS=Homo sapiens OX=9606 GN=RBX1 PE=1 SV=1	-0,023869902	0,072669
Q9P1F3	Costars family protein ABRACL OS=Homo sapiens OX=9606 GN=ABRACL PE=1 SV=1	0,032420462	0,072495
Q06203	Amidophosphoribosyltransferase OS=Homo sapiens OX=9606 GN=PPAT PE=1 SV=1	-0,011239096	0,07213
O14787	Transportin-2 OS=Homo sapiens OX=9606 GN=TNPO2 PE=1 SV=3	-0,010264163	0,071905
Q6NW34	Nucleolus and neural progenitor protein OS=Homo sapiens OX=9606 GN=NEPRO PE=1 SV=3	-0,032003799	0,071544
P20339	Ras-related protein Rab-5A OS=Homo sapiens OX=9606 GN=RAB5A PE=1 SV=2	-0,023274106	0,070815
P13639	Elongation factor 2 OS=Homo sapiens OX=9606 GN=EEF2 PE=1 SV=4	-0,004420896	0,070402
P49590	Probable histidine--tRNA ligase, mitochondrial OS=Homo sapiens OX=9606 GN=HARS2 PE=1 SV=1	0,019227777	0,07023
Q96CB8	Integrator complex subunit 12 OS=Homo sapiens OX=9606 GN=INTS12 PE=1 SV=1	-0,031205985	0,069723
Q8N1F8	Serine/threonine-protein kinase 11-interacting protein OS=Homo sapiens OX=9606 GN=STK11IP PE=1 SV=4	-0,030908813	0,069045
Q9NQ48	Leucine zipper transcription factor-like protein 1 OS=Homo sapiens OX=9606 GN=LZTFL1 PE=1 SV=1	-0,030802537	0,068803
Q2NKX8	DNA excision repair protein ERCC-6-like OS=Homo sapiens OX=9606 GN=ERCC6L PE=1 SV=1	-0,010584963	0,067837
Q13889	General transcription factor IIH subunit 3 OS=Homo sapiens OX=9606 GN=GTF2H3 PE=1 SV=2	0,049464011	0,06774
Q9Y6K0	Choline/ethanolaminephosphotransferase 1 OS=Homo sapiens OX=9606 GN=CEPT1 PE=1 SV=1	0,030214435	0,067463
O00592	Podocalyxin OS=Homo sapiens OX=9606 GN=PODXL PE=1 SV=2	0,009631515	0,067374
Q8WUY3	Protein prune homolog 2 OS=Homo sapiens OX=9606 GN=PRUNE2 PE=1 SV=3	-0,052592791	0,066987
Q15075	Early endosome antigen 1 OS=Homo sapiens OX=9606 GN=EEA1 PE=1 SV=2	-0,005066568	0,066938
P26447	Protein S100-A4 OS=Homo sapiens OX=9606 GN=S100A4 PE=1 SV=1	0,014499627	0,066928
Q9HCK8	#N/D	-0,029922091	0,066797
Q9BZV1	UBX domain-containing protein 6 OS=Homo sapiens OX=9606 GN=UBXN6 PE=1 SV=1	0,010425959	0,066795
Q9UNX4	WD repeat-containing protein 3 OS=Homo sapiens OX=9606 GN=WDR3 PE=1 SV=1	-0,010954657	0,06673
Q8N1G2	Cap-specific mRNA (nucleoside-2'-O-)-methyltransferase 1 OS=Homo sapiens OX=9606 GN=CMTR1 PE=1 SV=1	-0,015954564	0,066397
Q9BRA2	Thioredoxin domain-containing protein 17 OS=Homo sapiens OX=9606 GN=TXNDC17 PE=1 SV=1	-0,018176352	0,066306
Q15427	Splicing factor 3B subunit 4 OS=Homo sapiens OX=9606 GN=SF3B4 PE=1 SV=1	0,015903608	0,06618
Q8N9I9	Probable E3 ubiquitin-protein ligase DTX3 OS=Homo sapiens OX=9606 GN=DTX3 PE=1 SV=2	0,060851119	0,066106
Q96TA2	ATP-dependent zinc metalloprotease YME1L1 OS=Homo sapiens OX=9606 GN=YME1L1 PE=1 SV=2	0,011477045	0,066081
P50336	Protoporphyrinogen oxidase OS=Homo sapiens OX=9606 GN=PPOX PE=1 SV=1	0,012221329	0,066047
O15075	#N/D	0,033671862	0,065816
P54619	5'-AMP-activated protein kinase subunit gamma-1 OS=Homo sapiens OX=9606 GN=PRKAG1 PE=1 SV=1	-0,015763745	0,065586
Q96ST8	Centrosomal protein of 89 kDa OS=Homo sapiens OX=9606 GN=CEP89 PE=1 SV=3	-0,025541409	0,06539
P62979	Ubiquitin-40S ribosomal protein S27a OS=Homo sapiens OX=9606 GN=RPS27A PE=1 SV=2	-0,029196221	0,065145
P61026	Ras-related protein Rab-10 OS=Homo sapiens OX=9606 GN=RAB10 PE=1 SV=1	0,009698816	0,065009
P10606	Cytochrome c oxidase subunit 5B, mitochondrial OS=Homo sapiens OX=9606 GN=COX5B PE=1 SV=2	-0,012828566	0,064436
P22570	NADPH:adrenodoxin oxidoreductase, mitochondrial OS=Homo sapiens OX=9606 GN=FDXR PE=1 SV=3	-0,009205095	0,064327
O43242	26S proteasome non-ATPase regulatory subunit 3 OS=Homo sapiens OX=9606 GN=PSMD3 PE=1 SV=2	-0,00613675	0,063657
O75347	Tubulin-specific chaperone A OS=Homo sapiens OX=9606 GN=TBCA PE=1 SV=3	0,010454219	0,063617
P04179	Superoxide dismutase [Mn], mitochondrial OS=Homo sapiens OX=9606 GN=SOD2 PE=1 SV=3	-0,013780891	0,063541
Q6ZSR9	Uncharacterized protein FLJ45252 OS=Homo sapiens OX=9606 PE=2 SV=2	0,015264	0,063463
Q70EL1	Inactive ubiquitin carboxyl-terminal hydrolase 54 OS=Homo sapiens OX=9606 GN=USP54 PE=1 SV=4	0,028343313	0,063206
Q9BV57	1,2-dihydroxy-3-keto-5-methylthiopentene dioxygenase OS=Homo sapiens OX=9606 GN=ADI1 PE=1 SV=1	-0,010935228	0,062896
Q9BQB6	Vitamin K epoxide reductase complex subunit 1 OS=Homo sapiens OX=9606 GN=VKORC1 PE=1 SV=1	-0,027760536	0,061882
A8MWD9	Putative small nuclear ribonucleoprotein G-like protein 15 OS=Homo sapiens OX=9606 GN=SNRPGP15 PE=5 SV=2	-0,020282412	0,061532
Q969E8	Pre-rRNA-processing protein TSR2 homolog OS=Homo sapiens OX=9606 GN=TSR2 PE=1 SV=1	-0,027181765	0,060568
P05387	60S acidic ribosomal protein P2 OS=Homo sapiens OX=9606 GN=RPLP2 PE=1 SV=1	0,011983572	0,060107
P60953	Cell division control protein 42 homolog OS=Homo sapiens OX=9606 GN=CDC42 PE=1 SV=2	0,009859872	0,059928
O60341	Lysine-specific histone demethylase 1A OS=Homo sapiens OX=9606 GN=KDM1A PE=1 SV=2	-0,008832088	0,059085
P04083	Annexin A1 OS=Homo sapiens OX=9606 GN=ANXA1 PE=1 SV=2	0,00600177	0,058663
O43852	Calumenin OS=Homo sapiens OX=9606 GN=CALU PE=1 SV=2	-0,007547053	0,058645
P61011	Signal recognition particle 54 kDa protein OS=Homo sapiens OX=9606 GN=SRP54 PE=1 SV=1	-0,009151372	0,05847

Q9P0I2	ER membrane protein complex subunit 3 OS=Homo sapiens OX=9606 GN=EMC3 PE=1 SV=3	-0,019140209	0,058002
Q9BW91	ADP-ribose pyrophosphatase, mitochondrial OS=Homo sapiens OX=9606 GN=NUDT9 PE=1 SV=1	-0,01593647	0,05798
P47224	Guanine nucleotide exchange factor MSS4 OS=Homo sapiens OX=9606 GN=RABIF PE=1 SV=2	-0,025978966	0,05784
P55039	Developmentally-regulated GTP-binding protein 2 OS=Homo sapiens OX=9606 GN=DRG2 PE=1 SV=1	-0,012530189	0,057664
Q9GZT9	Egl nine homolog 1 OS=Homo sapiens OX=9606 GN=EGLN1 PE=1 SV=1	0,009493199	0,057656
Q8NEJ9	Neuroguidin OS=Homo sapiens OX=9606 GN=NGDN PE=1 SV=1	-0,025665606	0,05713
Q14155	Rho guanine nucleotide exchange factor 7 OS=Homo sapiens OX=9606 GN=ARHGEF7 PE=1 SV=2	-0,010486033	0,056493
Q9ULC3	Ras-related protein Rab-23 OS=Homo sapiens OX=9606 GN=RAB23 PE=1 SV=1	0,01855325	0,056191
O60551	Glycylpeptide N-tetradecanoyltransferase 2 OS=Homo sapiens OX=9606 GN=NMT2 PE=1 SV=1	-0,012184574	0,056044
O15078	Centrosomal protein of 290 kDa OS=Homo sapiens OX=9606 GN=CEP290 PE=1 SV=2	-0,068982922	0,055723
P07737	Profilin-1 OS=Homo sapiens OX=9606 GN=PFN1 PE=1 SV=2	-0,00833786	0,055717
Q8TCU4	Alstrom syndrome protein 1 OS=Homo sapiens OX=9606 GN=ALMS1 PE=1 SV=4	0,024865746	0,05532
Q7RTV0	PHD finger-like domain-containing protein 5A OS=Homo sapiens OX=9606 GN=PHF5A PE=1 SV=1	-0,013292963	0,055119
Q8N9N7	Leucine-rich repeat-containing protein 57 OS=Homo sapiens OX=9606 GN=LRRC57 PE=1 SV=1	0,024432204	0,054339
P29558	RNA-binding motif, single-stranded-interacting protein 1 OS=Homo sapiens OX=9606 GN=RBMS1 PE=1 SV=3	-0,024244182	0,053914
P15153	Ras-related C3 botulinum toxin substrate 2 OS=Homo sapiens OX=9606 GN=RAC2 PE=1 SV=1	0,011633807	0,053465
Q9UGP4	LIM domain-containing protein 1 OS=Homo sapiens OX=9606 GN=LIMD1 PE=1 SV=1	0,013515331	0,053363
Q14669	E3 ubiquitin-protein ligase TRIP12 OS=Homo sapiens OX=9606 GN=TRIP12 PE=1 SV=1	0,004804402	0,053157
P68366	Tubulin alpha-4A chain OS=Homo sapiens OX=9606 GN=TUBA4A PE=1 SV=1	-0,011538702	0,053021
Q06265	Exosome complex component RRP45 OS=Homo sapiens OX=9606 GN=EXOSC9 PE=1 SV=3	-0,012638789	0,052359
O95149	Snurportin-1 OS=Homo sapiens OX=9606 GN=SNUPN PE=1 SV=1	0,017049548	0,05156
Q8IV08	Phospholipase D3 OS=Homo sapiens OX=9606 GN=PLD3 PE=1 SV=1	-0,007296693	0,050764
Q86VS8	Protein Hook homolog 3 OS=Homo sapiens OX=9606 GN=HOOK3 PE=1 SV=2	-0,006991508	0,050554
P01111	GTPase NRas OS=Homo sapiens OX=9606 GN=NRAS PE=1 SV=1	-0,013830732	0,050192
Q5W0V3	Protein FAM160B1 OS=Homo sapiens OX=9606 GN=FAM160B1 PE=1 SV=1	0,008246093	0,049955
P17655	Calpain-2 catalytic subunit OS=Homo sapiens OX=9606 GN=CAPN2 PE=1 SV=6	0,004377624	0,049133
Q15165	Serum paraoxonase/arylesterase 2 OS=Homo sapiens OX=9606 GN=PON2 PE=1 SV=4	0,00719604	0,047964
Q9Y3B8	Oligoribonuclease, mitochondrial OS=Homo sapiens OX=9606 GN=REXO2 PE=1 SV=3	-0,007517259	0,047862
P51812	Ribosomal protein S6 kinase alpha-3 OS=Homo sapiens OX=9606 GN=RPS6KA3 PE=1 SV=1	0,006373964	0,047727
Q9Y383	Putative RNA-binding protein Luc7-like 2 OS=Homo sapiens OX=9606 GN=LUC7L2 PE=1 SV=2	-0,00885016	0,047539
Q9NPF4	Probable tRNA N6-adenosine threonylcarbamoyltransferase OS=Homo sapiens OX=9606 GN=OSGEP PE=1 SV=1	-0,021312467	0,047298
Q9BVG9	Phosphatidylserine synthase 2 OS=Homo sapiens OX=9606 GN=PTDSS2 PE=1 SV=1	-0,042006442	0,04671
Q14457	Beclin-1 OS=Homo sapiens OX=9606 GN=BECN1 PE=1 SV=2	-0,011216875	0,046377
Q99417	c-Myc-binding protein OS=Homo sapiens OX=9606 GN=MYCBP PE=1 SV=3	-0,012780203	0,046321
Q8WU90	Zinc finger CCCH domain-containing protein 15 OS=Homo sapiens OX=9606 GN=ZC3H15 PE=1 SV=1	0,006394918	0,046173
Q96MW1	Coiled-coil domain-containing protein 43 OS=Homo sapiens OX=9606 GN=CCDC43 PE=1 SV=2	0,015271797	0,046103
O14773	Tripeptidyl-peptidase 1 OS=Homo sapiens OX=9606 GN=TPP1 PE=1 SV=2	0,012503293	0,045302
Q4G148	Glucoside xylosyltransferase 1 OS=Homo sapiens OX=9606 GN=GXYLT1 PE=1 SV=2	-0,014703888	0,044364
P61604	10 kDa heat shock protein, mitochondrial OS=Homo sapiens OX=9606 GN=HSPE1 PE=1 SV=2	-0,006924819	0,044034
O00268	Transcription initiation factor TFIID subunit 4 OS=Homo sapiens OX=9606 GN=TAF4 PE=1 SV=2	0,019804263	0,043905
Q06124	Tyrosine-protein phosphatase non-receptor type 11 OS=Homo sapiens OX=9606 GN=PTPN11 PE=1 SV=2	0,004474523	0,043516
P40818	Ubiquitin carboxyl-terminal hydrolase 8 OS=Homo sapiens OX=9606 GN=USP8 PE=1 SV=1	-0,011962763	0,043316
Q86U70	LIM domain-binding protein 1 OS=Homo sapiens OX=9606 GN=LDB1 PE=1 SV=2	0,020059763	0,043151
P00387	NADH-cytochrome b5 reductase 3 OS=Homo sapiens OX=9606 GN=CYB5R3 PE=1 SV=3	-0,005100313	0,043086
Q15628	Tumor necrosis factor receptor type 1-associated DEATH domain protein OS=Homo sapiens OX=9606 GN=TRADD PE=1 SV	0,011875805	0,042996
P42345	Serine/threonine-protein kinase mTOR OS=Homo sapiens OX=9606 GN=MTOR PE=1 SV=1	0,006301935	0,04192
P29218	Inositol monophosphatase 1 OS=Homo sapiens OX=9606 GN=IMPA1 PE=1 SV=1	0,009056568	0,041457
O95881	Thioredoxin domain-containing protein 12 OS=Homo sapiens OX=9606 GN=TXNDC12 PE=1 SV=1	0,009023135	0,041302
O94992	Protein HEXIM1 OS=Homo sapiens OX=9606 GN=HEXIM1 PE=1 SV=1	-0,013680563	0,041234
P34897	Serine hydroxymethyltransferase, mitochondrial OS=Homo sapiens OX=9606 GN=SHMT2 PE=1 SV=3	0,004062117	0,041054
Q9Y450	HBS1-like protein OS=Homo sapiens OX=9606 GN=HBS1L PE=1 SV=1	0,005492118	0,041032
Q9NXV6	CDKN2A-interacting protein OS=Homo sapiens OX=9606 GN=CDKN2AIP PE=1 SV=3	0,006162452	0,04098
P11586	C-1-tetrahydrofolate synthase, cytoplasmic OS=Homo sapiens OX=9606 GN=MTHFD1 PE=1 SV=3	0,003559236	0,04044
Q13907	Isopentenyl-diphosphate Delta-isomerase 1 OS=Homo sapiens OX=9606 GN=IDI1 PE=1 SV=2	-0,007536334	0,040386
Q659C4	La-related protein 1B OS=Homo sapiens OX=9606 GN=LARP1B PE=1 SV=2	-0,008085273	0,03993
Q96P70	Importin-9 OS=Homo sapiens OX=9606 GN=IPO9 PE=1 SV=3	-0,003621517	0,039893
Q9HAT2	Sialate O-acetylesterase OS=Homo sapiens OX=9606 GN=SIAE PE=1 SV=1	-0,017832576	0,039479
Q01628	Interferon-induced transmembrane protein 3 OS=Homo sapiens OX=9606 GN=IFITM3 PE=1 SV=2	0,012981894	0,039101
P19174	1-phosphatidylinositol 4,5-bisphosphate phosphodiesterase gamma-1 OS=Homo sapiens OX=9606 GN=PLCG1 PE=1 SV=1	0,004173527	0,038834
P53701	Cytochrome c-type heme lyase OS=Homo sapiens OX=9606 GN=HCCS PE=1 SV=1	-0,005263472	0,037899
Q9Y6M9	NADH dehydrogenase [ubiquinone] 1 beta subcomplex subunit 9 OS=Homo sapiens OX=9606 GN=NDUFB9 PE=1 SV=3	-0,010484642	0,037896
Q9H2H8	Peptidyl-prolyl cis-trans isomerase-like 3 OS=Homo sapiens OX=9606 GN=PPIL3 PE=1 SV=1	-0,007283421	0,036245
Q08AM6	Protein VAC14 homolog OS=Homo sapiens OX=9606 GN=VAC14 PE=1 SV=1	-0,007901673	0,036106
Q9NY33	Dipeptidyl peptidase 3 OS=Homo sapiens OX=9606 GN=DPP3 PE=1 SV=2	-0,003867433	0,035951
Q9Y2G8	DnaJ homolog subfamily C member 16 OS=Homo sapiens OX=9606 GN=DNAJC16 PE=2 SV=3	-0,019063581	0,035552
Q96MX6	WD repeat-containing protein 92 OS=Homo sapiens OX=9606 GN=WDR92 PE=1 SV=1	-0,007696819	0,035159
Q9BVM2	Protein DPCD OS=Homo sapiens OX=9606 GN=DPCD PE=1 SV=2	0,015760123	0,03484
Q13561	Dynactin subunit 2 OS=Homo sapiens OX=9606 GN=DCTN2 PE=1 SV=4	0,003940726	0,034077
P52815	39S ribosomal protein L12, mitochondrial OS=Homo sapiens OX=9606 GN=MRPL12 PE=1 SV=2	0,008033086	0,033068
O94907	Dickkopf-related protein 1 OS=Homo sapiens OX=9606 GN=DKK1 PE=1 SV=1	-0,006566662	0,032639
Q9H2P9	Diphthine methyl ester synthase OS=Homo sapiens OX=9606 GN=DPH5 PE=1 SV=2	-0,010640439	0,031974
Q9NXH9	tRNA (guanine(26)-N(2))-dimethyltransferase OS=Homo sapiens OX=9606 GN=TRMT1 PE=1 SV=1	-0,005981533	0,031964
P05386	60S acidic ribosomal protein P1 OS=Homo sapiens OX=9606 GN=RPLP1 PE=1 SV=1	0,010581954	0,031796
P28062	Proteasome subunit beta type-8 OS=Homo sapiens OX=9606 GN=PSMB8 PE=1 SV=3	-0,005922384	0,031645
O15260	Surfeit locus protein 4 OS=Homo sapiens OX=9606 GN=SURF4 PE=1 SV=3	0,006872746	0,031354
Q9Y5N5	Methyltransferase N6AMT1 OS=Homo sapiens OX=9606 GN=N6AMT1 PE=1 SV=4	-0,010154936	0,0305
Q9BZZ5	Apoptosis inhibitor 5 OS=Homo sapiens OX=9606 GN=API5 PE=1 SV=3	0,004086678	0,029341
P62081	40S ribosomal protein S7 OS=Homo sapiens OX=9606 GN=RPS7 PE=1 SV=1	-0,004375514	0,02898
Q9H9H4	Vacuolar protein sorting-associated protein 37B OS=Homo sapiens OX=9606 GN=VPS37B PE=1 SV=1	0,007847705	0,028274
Q96JJ7	Protein disulfide-isomerase TMX3 OS=Homo sapiens OX=9606 GN=TMX3 PE=1 SV=2	-0,00755697	0,027217
Q9NNW5	WD repeat-containing protein 6 OS=Homo sapiens OX=9606 GN=WDR6 PE=1 SV=1	0,007515333	0,027066
O95801	Tetratricopeptide repeat protein 4 OS=Homo sapiens OX=9606 GN=TTC4 PE=1 SV=3	0,005931248	0,02702
Q9P2Q2	FERM domain-containing protein 4A OS=Homo sapiens OX=9606 GN=FRMD4A PE=1 SV=3	-0,007352128	0,026811
Q15155	Nodal modulator 1 OS=Homo sapiens OX=9606 GN=NOMO1 PE=1 SV=5	0,012118953	0,02672
Q8N183	NADH dehydrogenase [ubiquinone] 1 alpha subcomplex assembly factor 2 OS=Homo sapiens OX=9606 GN=NDUFAF2 PE=	0,005303594	0,026305
Q5VV42	Threonylcarbamoyladenosine tRNA methylthiotransferase OS=Homo sapiens OX=9606 GN=CDKAL1 PE=1 SV=1	-0,009055165	0,026248
P56211	cAMP-regulated phosphoprotein 19 OS=Homo sapiens OX=9606 GN=ARPP19 PE=1 SV=2	0,008747212	0,026235
Q96N66	Lysophospholipid acyltransferase 7 OS=Homo sapiens OX=9606 GN=MBOAT7 PE=1 SV=2	0,004363609	0,026226
Q9UKJ3	G patch domain-containing protein 8 OS=Homo sapiens OX=9606 GN=GPATCH8 PE=1 SV=2	0,00871134	0,026126
Q8IZP0	Abl interactor 1 OS=Homo sapiens OX=9606 GN=ABI1 PE=1 SV=4	0,005134889	0,025461
O95714	E3 ubiquitin-protein ligase HERC2 OS=Homo sapiens OX=9606 GN=HERC2 PE=1 SV=2	-0,008488863	0,025453

Q96E29	Transcription termination factor 3, mitochondrial OS=Homo sapiens OX=9606 GN=MTERF3 PE=1 SV=2	0,011284947	0,024867
Q13643	Four and a half LIM domains protein 3 OS=Homo sapiens OX=9606 GN=FHL3 PE=1 SV=4	0,011239897	0,024766
P51571	Translocon-associated protein subunit delta OS=Homo sapiens OX=9606 GN=SSR4 PE=1 SV=1	-0,004943637	0,024505
O15360	Fanconi anemia group A protein OS=Homo sapiens OX=9606 GN=FANCA PE=1 SV=2	0,011036689	0,024315
Q16629	Serine/arginine-rich splicing factor 7 OS=Homo sapiens OX=9606 GN=SRSF7 PE=1 SV=1	-0,004272187	0,024259
Q8WUF5	RelA-associated inhibitor OS=Homo sapiens OX=9606 GN=PPP1R13L PE=1 SV=4	0,003152606	0,024218
Q5VT06	Centrosome-associated protein 350 OS=Homo sapiens OX=9606 GN=CEP350 PE=1 SV=1	-0,010923519	0,024064
P07437	Tubulin beta chain OS=Homo sapiens OX=9606 GN=TUBB PE=1 SV=2	0,005847721	0,023999
Q9NP79	Vacuolar protein sorting-associated protein VTA1 homolog OS=Homo sapiens OX=9606 GN=VTA1 PE=1 SV=1	0,004482671	0,023889
Q9BPZ7	Target of rapamycin complex 2 subunit MAPKAP1 OS=Homo sapiens OX=9606 GN=MAPKAP1 PE=1 SV=2	-0,018043454	0,023483
A5PLL7	Transmembrane protein 189 OS=Homo sapiens OX=9606 GN=TMEM189 PE=1 SV=3	0,010583611	0,023309
Q14156	Protein EFR3 homolog A OS=Homo sapiens OX=9606 GN=EFR3A PE=1 SV=2	-0,007667787	0,022972
O60936	Nucleolar protein 3 OS=Homo sapiens OX=9606 GN=NOL3 PE=1 SV=2	-0,007424372	0,022237
Q16513	Serine/threonine-protein kinase N2 OS=Homo sapiens OX=9606 GN=PKN2 PE=1 SV=1	-0,010092923	0,022221
Q68DQ2	Very large A-kinase anchor protein OS=Homo sapiens OX=9606 GN=CRYBG3 PE=1 SV=3	0,017662096	0,022178
P08754	Guanine nucleotide-binding protein G(i) subunit alpha OS=Homo sapiens OX=9606 GN=GNAI3 PE=1 SV=3	0,004852752	0,02207
O43813	Glutathione S-transferase LANCL1 OS=Homo sapiens OX=9606 GN=LANCL1 PE=1 SV=1	-0,003295297	0,021772
P50747	Biotin--protein ligase OS=Homo sapiens OX=9606 GN=HLCS PE=1 SV=1	-0,009831023	0,02164
O95999	B-cell lymphoma/leukemia 10 OS=Homo sapiens OX=9606 GN=BCL10 PE=1 SV=1	0,009702034	0,021354
P62913	60S ribosomal protein L11 OS=Homo sapiens OX=9606 GN=RPL11 PE=1 SV=2	0,003674492	0,020841
Q13153	Serine/threonine-protein kinase PAK 1 OS=Homo sapiens OX=9606 GN=PAK1 PE=1 SV=2	0,003847526	0,020481
Q9NP97	Dynein light chain roadblock-type 1 OS=Homo sapiens OX=9606 GN=DYNLRB1 PE=1 SV=3	0,004109759	0,020343
P50990	T-complex protein 1 subunit theta OS=Homo sapiens OX=9606 GN=CCT8 PE=1 SV=4	-0,001644295	0,020149
Q9Y224	RNA transcription, translation and transport factor protein OS=Homo sapiens OX=9606 GN=RTRAF PE=1 SV=1	0,003032768	0,020026
P48509	CD151 antigen OS=Homo sapiens OX=9606 GN=CD151 PE=1 SV=3	0,004858523	0,019911
P62306	Small nuclear ribonucleoprotein F OS=Homo sapiens OX=9606 GN=SNRPF PE=1 SV=1	-0,006377213	0,019081
Q13574	Diacylglycerol kinase zeta OS=Homo sapiens OX=9606 GN=DGKZ PE=1 SV=3	0,004360961	0,019026
P08621	U1 small nuclear ribonucleoprotein 70 kDa OS=Homo sapiens OX=9606 GN=SNRNP70 PE=1 SV=2	-0,002387317	0,018302
Q6GQQ9	OTU domain-containing protein 7B OS=Homo sapiens OX=9606 GN=OTUD7B PE=1 SV=1	-0,005044576	0,018113
Q6IQ22	Ras-related protein Rab-12 OS=Homo sapiens OX=9606 GN=RAB12 PE=1 SV=3	0,008145377	0,017907
Q96J42	Thioredoxin domain-containing protein 15 OS=Homo sapiens OX=9606 GN=TXNDC15 PE=1 SV=1	0,005929823	0,017734
Q92734	Protein TFG OS=Homo sapiens OX=9606 GN=TFG PE=1 SV=2	0,002930462	0,017561
P17948	Vascular endothelial growth factor receptor 1 OS=Homo sapiens OX=9606 GN=FLT1 PE=1 SV=2	0,007591315	0,017173
Q06323	Proteasome activator complex subunit 1 OS=Homo sapiens OX=9606 GN=PSME1 PE=1 SV=1	-0,002209035	0,016927
Q9UKY7	Protein CDV3 homolog OS=Homo sapiens OX=9606 GN=CDV3 PE=1 SV=1	0,002773317	0,016614
P62888	60S ribosomal protein L30 OS=Homo sapiens OX=9606 GN=RPL30 PE=1 SV=2	-0,003353487	0,016578
P53801	Pituitary tumor-transforming gene 1 protein-interacting protein OS=Homo sapiens OX=9606 GN=PTTG1IP PE=1 SV=1	0,007479	0,016434
P38435	Vitamin K-dependent gamma-carboxylase OS=Homo sapiens OX=9606 GN=GGCX PE=1 SV=2	0,005451671	0,016296
P06753	Tropomyosin alpha-3 chain OS=Homo sapiens OX=9606 GN=TPM3 PE=1 SV=2	0,004370255	0,015679
P17050	Alpha-N-acetylgalactosaminidase OS=Homo sapiens OX=9606 GN=NAGA PE=1 SV=2	-0,007095374	0,015587
Q969F9	Hermansky-Pudlak syndrome 3 protein OS=Homo sapiens OX=9606 GN=HPS3 PE=1 SV=1	-0,016505024	0,01549
Q9H7Z7	Prostaglandin E synthase 2 OS=Homo sapiens OX=9606 GN=PTGES2 PE=1 SV=1	0,004317305	0,015488
Q9NSP4	Centromere protein M OS=Homo sapiens OX=9606 GN=CENPM PE=1 SV=1	-0,008641093	0,014965
P01116	GTPase KRas OS=Homo sapiens OX=9606 GN=KRAS PE=1 SV=1	0,003637415	0,014882
O14770	Homeobox protein Meis2 OS=Homo sapiens OX=9606 GN=MEIS2 PE=1 SV=2	-0,006564134	0,014414
P06730	Eukaryotic translation initiation factor 4E OS=Homo sapiens OX=9606 GN=EIF4E PE=1 SV=2	0,002778663	0,013723
Q9BRP8	Partner of Y14 and mago OS=Homo sapiens OX=9606 GN=PYM1 PE=1 SV=1	0,003354872	0,01372
Q969Q0	60S ribosomal protein L36a-like OS=Homo sapiens OX=9606 GN=RPL36AL PE=1 SV=3	0,003818568	0,01369
P51636	Caveolin-2 OS=Homo sapiens OX=9606 GN=CAV2 PE=1 SV=2	0,006059414	0,013301
P11171	Protein 4.1 OS=Homo sapiens OX=9606 GN=EPB41 PE=1 SV=4	-0,00438861	0,013104
Q6P4E1	Protein CASC4 OS=Homo sapiens OX=9606 GN=CASC4 PE=1 SV=2	0,005940663	0,013039
P31323	cAMP-dependent protein kinase type II-beta regulatory subunit OS=Homo sapiens OX=9606 GN=PRKAR2B PE=1 SV=3	0,004306413	0,012858
O95429	BAG family molecular chaperone regulator 4 OS=Homo sapiens OX=9606 GN=BAG4 PE=1 SV=1	-0,004279247	0,012776
Q08623	Pseudouridine-5'-phosphatase OS=Homo sapiens OX=9606 GN=PUDP PE=1 SV=3	-0,00347158	0,012441
P43243	Matrin-3 OS=Homo sapiens OX=9606 GN=MATR3 PE=1 SV=2	0,001084454	0,012386
Q92934	Bcl2-associated agonist of cell death OS=Homo sapiens OX=9606 GN=BAD PE=1 SV=3	-0,004057116	0,01211
Q96HW7	Integrator complex subunit 4 OS=Homo sapiens OX=9606 GN=INTS4 PE=1 SV=2	-0,003940668	0,011761
O75170	Serine/threonine-protein phosphatase 6 regulatory subunit 2 OS=Homo sapiens OX=9606 GN=PPP6R2 PE=1 SV=2	0,005317788	0,011667
P55786	Puromycin-sensitive aminopeptidase OS=Homo sapiens OX=9606 GN=NPEPPS PE=1 SV=2	0,000998442	0,0114
P51965	#N/D	0,002584486	0,010762
P49756	RNA-binding protein 25 OS=Homo sapiens OX=9606 GN=RBM25 PE=1 SV=3	-0,001323731	0,010757
P53680	AP-2 complex subunit sigma OS=Homo sapiens OX=9606 GN=AP2S1 PE=1 SV=2	0,00296791	0,010629
Q9H7B4	Histone-lysine N-methyltransferase SMYD3 OS=Homo sapiens OX=9606 GN=SMYD3 PE=1 SV=4	-0,004829765	0,010592
Q15369	Elongin-C OS=Homo sapiens OX=9606 GN=ELOC PE=1 SV=1	-0,002517316	0,010283
Q9Y2L1	Exosome complex exonuclease RRP44 OS=Homo sapiens OX=9606 GN=DIS3 PE=1 SV=2	0,001733553	0,009795
P18085	ADP-ribosylation factor 4 OS=Homo sapiens OX=9606 GN=ARF4 PE=1 SV=3	0,001821278	0,009659
Q7Z4S6	Kinesin-like protein KIF21A OS=Homo sapiens OX=9606 GN=KIF21A PE=1 SV=2	-0,002655095	0,009505
P51668	Ubiquitin-conjugating enzyme E2 D1 OS=Homo sapiens OX=9606 GN=UBE2D1 PE=1 SV=1	-0,004326432	0,009485
P60903	Protein S100-A10 OS=Homo sapiens OX=9606 GN=S100A10 PE=1 SV=2	-0,001738525	0,009219
P54886	Delta-1-pyrroline-5-carboxylate synthase OS=Homo sapiens OX=9606 GN=ALDH18A1 PE=1 SV=2	-0,000920052	0,009199
Q9Y3L5	Ras-related protein Rap-2c OS=Homo sapiens OX=9606 GN=RAP2C PE=1 SV=1	-0,004038612	0,008852
Q9NX20	39S ribosomal protein L16, mitochondrial OS=Homo sapiens OX=9606 GN=MRPL16 PE=1 SV=1	-0,002064463	0,008428
Q96T23	Remodeling and spacing factor 1 OS=Homo sapiens OX=9606 GN=RSF1 PE=1 SV=2	-0,00274795	0,008192
A8MT19	#N/D	-0,00166087	0,008111
P30085	UMP-CMP kinase OS=Homo sapiens OX=9606 GN=CMPK1 PE=1 SV=3	-0,001308879	0,007817
Q9H0C8	Integrin-linked kinase-associated serine/threonine phosphatase 2C OS=Homo sapiens OX=9606 GN=ILKAP PE=1 SV=1	-0,001128307	0,007418
O15357	Phosphatidylinositol 3,4,5-trisphosphate 5-phosphatase 2 OS=Homo sapiens OX=9606 GN=INPPL1 PE=1 SV=2	-0,001042892	0,007149
O95486	Protein transport protein Sec24A OS=Homo sapiens OX=9606 GN=SEC24A PE=1 SV=2	-0,000770216	0,006598
P11166	Solute carrier family 2, facilitated glucose transporter member 1 OS=Homo sapiens OX=9606 GN=SLC2A1 PE=1 SV=2	0,001456993	0,006591
O43169	Cytochrome b5 type B OS=Homo sapiens OX=9606 GN=CYB5B PE=1 SV=3	-0,001419525	0,006421
Q70CQ2	Ubiquitin carboxyl-terminal hydrolase 34 OS=Homo sapiens OX=9606 GN=USP34 PE=1 SV=2	-0,001206586	0,005943
Q9H6F5	Coiled-coil domain-containing protein 86 OS=Homo sapiens OX=9606 GN=CCDC86 PE=1 SV=1	0,002925691	0,005755
Q9H1Y0	Autophagy protein 5 OS=Homo sapiens OX=9606 GN=ATG5 PE=1 SV=2	0,00187432	0,005582
Q9UHB7	AF4/FMR2 family member 4 OS=Homo sapiens OX=9606 GN=AFF4 PE=1 SV=1	-0,001593377	0,004744
Q00059	Transcription factor A, mitochondrial OS=Homo sapiens OX=9606 GN=TFAM PE=1 SV=1	0,000875227	0,004634
Q5VWQ8	Disabled homolog 2-interacting protein OS=Homo sapiens OX=9606 GN=DAB2IP PE=1 SV=2	0,002055357	0,004498
Q9NXV2	BTB/POZ domain-containing protein KCTD5 OS=Homo sapiens OX=9606 GN=KCTD5 PE=1 SV=1	0,001957767	0,004284
O15371	Eukaryotic translation initiation factor 3 subunit D OS=Homo sapiens OX=9606 GN=EIF3D PE=1 SV=1	-0,000445542	0,00391
Q08752	Peptidyl-prolyl cis-trans isomerase D OS=Homo sapiens OX=9606 GN=PPID PE=1 SV=3	-0,000588331	0,003863
Q6NUQ1	RAD50-interacting protein 1 OS=Homo sapiens OX=9606 GN=RINT1 PE=1 SV=1	0,002290852	0,003625
P60228	Eukaryotic translation initiation factor 3 subunit E OS=Homo sapiens OX=9606 GN=EIF3E PE=1 SV=1	-0,00034411	0,003019

P56381	ATP synthase subunit epsilon, mitochondrial OS=Homo sapiens OX=9606 GN=ATP5F1E PE=1 SV=2	-0,001314407	0,002875
Q92841	Probable ATP-dependent RNA helicase DDX17 OS=Homo sapiens OX=9606 GN=DDX17 PE=1 SV=2	-0,000250424	0,00268
Q9NP58	ATP-binding cassette sub-family B member 6, mitochondrial OS=Homo sapiens OX=9606 GN=ABCB6 PE=1 SV=1	-0,000463805	0,002282
P19447	General transcription and DNA repair factor IIH helicase subunit XPB OS=Homo sapiens OX=9606 GN=ERCC3 PE=1 SV=1	-0,000972801	0,002127
P15586	N-acetylglucosamine-6-sulfatase OS=Homo sapiens OX=9606 GN=GNS PE=1 SV=3	0,000335926	0,002106
Q9C098	#N/D	-0,003670086	0,001949
Q16630	Cleavage and polyadenylation specificity factor subunit 6 OS=Homo sapiens OX=9606 GN=CPSF6 PE=1 SV=2	-0,0003236	0,001823
Q9BUN8	Derlin-1 OS=Homo sapiens OX=9606 GN=DERL1 PE=1 SV=1	-0,0005017	0,001791
Q9UG01	Intraflagellar transport protein 172 homolog OS=Homo sapiens OX=9606 GN=IFT172 PE=1 SV=2	-0,000771658	0,001687
Q9HC38	Glyoxalase domain-containing protein 4 OS=Homo sapiens OX=9606 GN=GLOD4 PE=1 SV=1	0,000140034	0,000996
Q9BYV8	Centrosomal protein of 41 kDa OS=Homo sapiens OX=9606 GN=CEP41 PE=1 SV=1	0,000162414	0,000661
Q14149	MORC family CW-type zinc finger protein 3 OS=Homo sapiens OX=9606 GN=MORC3 PE=1 SV=3	-0,000119404	0,000426
Q969S9	Ribosome-releasing factor 2, mitochondrial OS=Homo sapiens OX=9606 GN=GFM2 PE=1 SV=1	4,79751E-05	0,000236
Q15293	Reticulocalbin-1 OS=Homo sapiens OX=9606 GN=RCN1 PE=1 SV=1	-2,77374E-05	0,000197
