# Supplementary material for: Selective cell cycle arrest in glioblastoma cell lines by quantum molecular resonance alone or in combination with temozolomide
Source: Br J Cancer. 2022 Jun 17;127(5):824–35. doi: 10.1038/s41416-022-01865-9 (PMC9427848; doi:10.1038/s41416-022-01865-9)
Supplement: Supplementary file 4 — Supplementary Table 3 [file 41416_2022_1865_MOESM4_ESM.pdf]

Supplementary Table 3: List of proteins identified as differentially expressed between QMR-stimulated BM-MSCs and untreated BM-MSCs.
For each protein, Master Protein Accessions number (Uniprot), Description, Prot. Fold change (QMR-stimulated cells/Untreated cells) and p-value are reported. Prot. Fold change are given as log2-fold changes, while p-value as -log2 (p-value). Statistical confidence was assessed when p-value < 0.05, corresponding to -log2 (p-value) of 4.32.

Master Protein Accessions	Description	Prot. Fold change (QMR/CON)	p-value
Q99715	Collagen alpha-1(XII) chain OS=Homo sapiens OX=9606 GN=COL12A1 PE=1 SV=2	-0,758390162	57,70373
P02452	Collagen alpha-1(I) chain OS=Homo sapiens OX=9606 GN=COL1A1 PE=1 SV=5	-0,757452754	45,72545
P08123	Collagen alpha-2(I) chain OS=Homo sapiens OX=9606 GN=COL1A2 PE=1 SV=7	-0,711646487	41,66983
Q92598	Heat shock protein 105 kDa OS=Homo sapiens OX=9606 GN=HSPH1 PE=1 SV=1	1,126967004	31,2417
P0DMV8	Heat shock 70 kDa protein 1A OS=Homo sapiens OX=9606 GN=HSPA1A PE=1 SV=1	0,896118186	25,49714
P14625	Endoplasmin OS=Homo sapiens OX=9606 GN=HSP90B1 PE=1 SV=1	0,490936114	22,23891
Q02790	Peptidyl-prolyl cis-trans isomerase FKBP4 OS=Homo sapiens OX=9606 GN=FKBP4 PE=1 SV=3	1,121903719	21,48443
P02751	Fibronectin OS=Homo sapiens OX=9606 GN=FN1 PE=1 SV=4	-0,364299163	18,92638
P13667	Protein disulfide-isomerase A4 OS=Homo sapiens OX=9606 GN=PDIA4 PE=1 SV=2	0,510793407	17,79999
P07900	Heat shock protein HSP 90-alpha OS=Homo sapiens OX=9606 GN=HSP90AA1 PE=1 SV=5	0,463011736	17,48043
P05997	Collagen alpha-2(V) chain OS=Homo sapiens OX=9606 GN=COL5A2 PE=1 SV=3	-0,919889616	13,25978
P10809	60 kDa heat shock protein, mitochondrial OS=Homo sapiens OX=9606 GN=HSPD1 PE=1 SV=2	0,410378827	12,47751
P14618	Pyruvate kinase PKM OS=Homo sapiens OX=9606 GN=PKM PE=1 SV=4	0,368823932	12,28614
P08238	Heat shock protein HSP 90-beta OS=Homo sapiens OX=9606 GN=HSP90AB1 PE=1 SV=4	0,423588391	11,32308
Q9BVI4	Nucleolar complex protein 4 homolog OS=Homo sapiens OX=9606 GN=NOC4L PE=1 SV=1	2,560963124	11,05428
P07942	Laminin subunit beta-1 OS=Homo sapiens OX=9606 GN=LAMB1 PE=1 SV=2	0,484130322	10,77715
O94925	Glutaminase kidney isoform, mitochondrial OS=Homo sapiens OX=9606 GN=GLS PE=1 SV=1	0,511246151	10,4096
Q5T4B2	Inactive glycosyltransferase 25 family member 3 OS=Homo sapiens OX=9606 GN=CERCAM PE=1 SV=1	-0,962037358	10,12909
P07237	Protein disulfide-isomerase OS=Homo sapiens OX=9606 GN=P4HB PE=1 SV=3	0,364633371	10,04287
Q9UHI8	A disintegrin and metalloproteinase with thrombospondin motifs 1 OS=Homo sapiens OX=9606 GN=ADAMTS1 PE=1 SV=4	0,544369375	9,990744
Q15113	Procollagen C-endopeptidase enhancer 1 OS=Homo sapiens OX=9606 GN=PCOLCE PE=1 SV=2	-0,786224178	9,7646
P29279	CCN family member 2 OS=Homo sapiens OX=9606 GN=CCN2 PE=1 SV=2	-0,594560748	9,671558
P17813	Endoglin OS=Homo sapiens OX=9606 GN=ENG PE=1 SV=2	0,53898783	9,429922
P21980	Protein-glutamine gamma-glutamyltransferase 2 OS=Homo sapiens OX=9606 GN=TGM2 PE=1 SV=2	0,472303077	9,365748
Q32MZ4	Leucine-rich repeat flightless-interacting protein 1 OS=Homo sapiens OX=9606 GN=LRRFIP1 PE=1 SV=2	0,578456668	9,348843
P16949	Stathmin OS=Homo sapiens OX=9606 GN=STMN1 PE=1 SV=3	1,096310084	9,247624
Q9P2E9	Ribosome-binding protein 1 OS=Homo sapiens OX=9606 GN=RRBP1 PE=1 SV=5	0,271494322	9,109877
Q14240	Eukaryotic initiation factor 4A-II OS=Homo sapiens OX=9606 GN=EIF4A2 PE=1 SV=2	1,048874326	8,892569
P31948	Stress-induced-phosphoprotein 1 OS=Homo sapiens OX=9606 GN=STIP1 PE=1 SV=1	0,350539437	8,835639
P22626	Heterogeneous nuclear ribonucleoproteins A2/B1 OS=Homo sapiens OX=9606 GN=HNRNPA2B1 PE=1 SV=2	0,488253268	8,672425
P16112	Aggrecan core protein OS=Homo sapiens OX=9606 GN=ACAN PE=1 SV=3	-0,499730295	8,552148
P08195	4F2 cell-surface antigen heavy chain OS=Homo sapiens OX=9606 GN=SLC3A2 PE=1 SV=3	-0,433842492	8,347058
Q13217	DnaJ homolog subfamily C member 3 OS=Homo sapiens OX=9606 GN=DNAJC3 PE=1 SV=1	0,564683426	8,130898
P46821	Microtubule-associated protein 1B OS=Homo sapiens OX=9606 GN=MAP1B PE=1 SV=2	0,196642498	8,078958
Q70E73	Ras-associated and pleckstrin homology domains-containing protein 1 OS=Homo sapiens OX=9606 GN=RAPH1 PE=1 SV=3	-0,467818724	7,842566
Q76M96	Coiled-coil domain-containing protein 80 OS=Homo sapiens OX=9606 GN=CCDC80 PE=1 SV=1	-0,498094481	7,736092
P06576	ATP synthase subunit beta, mitochondrial OS=Homo sapiens OX=9606 GN=ATP5F1B PE=1 SV=3	0,346464608	7,625545
P49257	Protein ERGIC-53 OS=Homo sapiens OX=9606 GN=LMAN1 PE=1 SV=2	0,488603022	7,546207
P43243	Matrin-3 OS=Homo sapiens OX=9606 GN=MATR3 PE=1 SV=2	0,349294303	7,49859
Q9UHD1	Cysteine and histidine-rich domain-containing protein 1 OS=Homo sapiens OX=9606 GN=CHORDC1 PE=1 SV=2	0,711697392	7,435989
P08243	Asparagine synthetase [glutamine-hydrolyzing] OS=Homo sapiens OX=9606 GN=ASNS PE=1 SV=4	-0,38616819	7,370111
Q86VP6	Cullin-associated NEDD8-dissociated protein 1 OS=Homo sapiens OX=9606 GN=CAND1 PE=1 SV=2	0,260597439	7,281411
P06454	Prothymosin alpha OS=Homo sapiens OX=9606 GN=PTMA PE=1 SV=2	1,112665098	7,094421
Q6EMK4	Vasorin OS=Homo sapiens OX=9606 GN=VASN PE=1 SV=1	0,681630431	7,091688
P00367	Glutamate dehydrogenase 1, mitochondrial OS=Homo sapiens OX=9606 GN=GLUD1 PE=1 SV=2	0,433367621	7,083755
O00499	Myc box-dependent-interacting protein 1 OS=Homo sapiens OX=9606 GN=BIN1 PE=1 SV=1	0,589831781	7,054452
P07093	Glia-derived nexin OS=Homo sapiens OX=9606 GN=SERPINE2 PE=1 SV=1	0,62075458	6,940288
Q13501	Sequestosome-1 OS=Homo sapiens OX=9606 GN=SQSTM1 PE=1 SV=1	0,804709757	6,91064
Q16270	Insulin-like growth factor-binding protein 7 OS=Homo sapiens OX=9606 GN=IGFBP7 PE=1 SV=1	-0,579213167	6,90364
Q14204	Cytoplasmic dynein 1 heavy chain 1 OS=Homo sapiens OX=9606 GN=DYNC1H1 PE=1 SV=5	0,114461382	6,748619
Q14789	Golgin subfamily B member 1 OS=Homo sapiens OX=9606 GN=GOLGB1 PE=1 SV=2	0,324370163	6,742853
Q9UHB6	LIM domain and actin-binding protein 1 OS=Homo sapiens OX=9606 GN=LIMA1 PE=1 SV=1	0,328350921	6,665372
Q3SY69	Mitochondrial 10-formyltetrahydrofolate dehydrogenase OS=Homo sapiens OX=9606 GN=ALDH1L2 PE=1 SV=2	-0,280136603	6,658198
Q92900	Regulator of nonsense transcripts 1 OS=Homo sapiens OX=9606 GN=UPF1 PE=1 SV=2	0,309374157	6,654579
Q9Y490	Talin-1 OS=Homo sapiens OX=9606 GN=TLN1 PE=1 SV=3	0,148156874	6,593307
Q9UDY4	DnaJ homolog subfamily B member 4 OS=Homo sapiens OX=9606 GN=DNAJB4 PE=1 SV=1	0,63390267	6,537576
O14974	Protein phosphatase 1 regulatory subunit 12A OS=Homo sapiens OX=9606 GN=PPP1R12A PE=1 SV=1	0,270587373	6,465599
P52630	Signal transducer and activator of transcription 2 OS=Homo sapiens OX=9606 GN=STAT2 PE=1 SV=1	-0,583326416	6,454517
P11142	Heat shock cognate 71 kDa protein OS=Homo sapiens OX=9606 GN=HSPA8 PE=1 SV=1	0,291864862	6,436872
P51452	Dual specificity protein phosphatase 3 OS=Homo sapiens OX=9606 GN=DUSP3 PE=1 SV=1	0,846428724	6,423047
Q9Y613	FH1/FH2 domain-containing protein 1 OS=Homo sapiens OX=9606 GN=FHOD1 PE=1 SV=3	0,513242559	6,384057
O15294	UDP-N-acetylglucosamine--peptide N-acetylglucosaminyltransferase 110 kDa subunit OS=Homo sapiens OX=9606 GN=OGT PE=1 SV=3	0,463235166	6,331801
Q8N8S7	Protein enabled homolog OS=Homo sapiens OX=9606 GN=ENAH PE=1 SV=2	0,361982516	6,264167
Q9BXK5	Bcl-2-like protein 13 OS=Homo sapiens OX=9606 GN=BCL2L13 PE=1 SV=1	0,956955429	6,227803
P20908	Collagen alpha-1(V) chain OS=Homo sapiens OX=9606 GN=COL5A1 PE=1 SV=3	-0,294384745	6,192377
P02462	Collagen alpha-1(IV) chain OS=Homo sapiens OX=9606 GN=COL4A1 PE=1 SV=4	-0,601185431	6,153077
Q9BZF9	Uveal autoantigen with coiled-coil domains and ankyrin repeats OS=Homo sapiens OX=9606 GN=UACA PE=1 SV=2	0,403422016	6,149411
Q5T9A4	#N/D	0,80712637	6,131349
Q13620	Cullin-4B OS=Homo sapiens OX=9606 GN=CUL4B PE=1 SV=4	0,32948603	6,100697
Q99497	Protein/nucleic acid deglycase DJ-1 OS=Homo sapiens OX=9606 GN=PARK7 PE=1 SV=2	0,396077386	5,999289
O00410	Importin-5 OS=Homo sapiens OX=9606 GN=IPO5 PE=1 SV=4	0,240400831	5,998405
Q92973	Transportin-1 OS=Homo sapiens OX=9606 GN=TNPO1 PE=1 SV=2	0,349544043	5,967088
O60884	DnaJ homolog subfamily A member 2 OS=Homo sapiens OX=9606 GN=DNAJA2 PE=1 SV=1	0,545380325	5,958594
Q8NI22	Multiple coagulation factor deficiency protein 2 OS=Homo sapiens OX=9606 GN=MCFD2 PE=1 SV=1	0,901242773	5,897994
Q68EM7	Rho GTPase-activating protein 17 OS=Homo sapiens OX=9606 GN=ARHGAP17 PE=1 SV=1	0,687725859	5,869281
P61981	14-3-3 protein gamma OS=Homo sapiens OX=9606 GN=YWHAG PE=1 SV=2	0,479580969	5,864598
Q9Y4L1	Hypoxia up-regulated protein 1 OS=Homo sapiens OX=9606 GN=HYOU1 PE=1 SV=1	0,253146862	5,85958
Q9BUJ2	Heterogeneous nuclear ribonucleoprotein U-like protein 1 OS=Homo sapiens OX=9606 GN=HNRNPUL1 PE=1 SV=2	-0,478530359	5,848413
Q96T23	Remodeling and spacing factor 1 OS=Homo sapiens OX=9606 GN=RSF1 PE=1 SV=2	-1,1859986	5,846452
P13639	Elongation factor 2 OS=Homo sapiens OX=9606 GN=EEF2 PE=1 SV=4	0,182132774	5,839868
Q01433	AMP deaminase 2 OS=Homo sapiens OX=9606 GN=AMPD2 PE=1 SV=2	0,502137758	5,803898
P60709	Actin, cytoplasmic 1 OS=Homo sapiens OX=9606 GN=ACTB PE=1 SV=1	0,275797474	5,780478
Q7Z7H5	Transmembrane emp24 domain-containing protein 4 OS=Homo sapiens OX=9606 GN=TMED4 PE=1 SV=1	0,567551783	5,754638
Q92888	Rho guanine nucleotide exchange factor 1 OS=Homo sapiens OX=9606 GN=ARHGEF1 PE=1 SV=2	-0,383408867	5,741919
P78559	Microtubule-associated protein 1A OS=Homo sapiens OX=9606 GN=MAP1A PE=1 SV=6	0,18838219	5,721904
O43681	ATPase ASNA1 OS=Homo sapiens OX=9606 GN=ASNA1 PE=1 SV=2	0,606062101	5,66486
P17480	Nucleolar transcription factor 1 OS=Homo sapiens OX=9606 GN=UBTF PE=1 SV=1	-1,050014746	5,650917
P22314	Ubiquitin-like modifier-activating enzyme 1 OS=Homo sapiens OX=9606 GN=UBA1 PE=1 SV=3	0,211729124	5,601544
Q08AF3	Schlafen family member 5 OS=Homo sapiens OX=9606 GN=SLFN5 PE=1 SV=1	-0,486071201	5,574479
P43121	Cell surface glycoprotein MUC18 OS=Homo sapiens OX=9606 GN=MCAM PE=1 SV=2	0,374516717	5,562553
P26038	Moesin OS=Homo sapiens OX=9606 GN=MSN PE=1 SV=3	0,246701734	5,521339

O75431	Metaxin-2 OS=Homo sapiens OX=9606 GN=MTX2 PE=1 SV=1	-0,723907363	5,493771
Q8NI36	WD repeat-containing protein 36 OS=Homo sapiens OX=9606 GN=WDR36 PE=1 SV=1	0,715611478	5,476137
P52306	Rap1 GTPase-GDP dissociation stimulator 1 OS=Homo sapiens OX=9606 GN=RAP1GDS1 PE=1 SV=3	0,346516461	5,452996
O60287	Nucleolar pre-ribosomal-associated protein 1 OS=Homo sapiens OX=9606 GN=URB1 PE=1 SV=4	0,167758294	5,41428
Q9UIM3	FK506-binding protein-like OS=Homo sapiens OX=9606 GN=FKBPL PE=1 SV=1	2,674607776	5,389842
P54619	5'-AMP-activated protein kinase subunit gamma-1 OS=Homo sapiens OX=9606 GN=PRKAG1 PE=1 SV=1	-0,579216083	5,379203
Q9BRK3	Matrix remodeling-associated protein 8 OS=Homo sapiens OX=9606 GN=MXRA8 PE=1 SV=1	0,708422948	5,372241
Q86UP2	Kinectin OS=Homo sapiens OX=9606 GN=KTN1 PE=1 SV=1	0,21696171	5,340509
Q96CG8	Collagen triple helix repeat-containing protein 1 OS=Homo sapiens OX=9606 GN=CTHRC1 PE=1 SV=1	-0,629204817	5,328407
Q8TC12	Retinol dehydrogenase 11 OS=Homo sapiens OX=9606 GN=RDH11 PE=1 SV=2	0,793348255	5,228434
P05198	Eukaryotic translation initiation factor 2 subunit 1 OS=Homo sapiens OX=9606 GN=EIF2S1 PE=1 SV=3	0,335988647	5,225976
P78344	Eukaryotic translation initiation factor 4 gamma 2 OS=Homo sapiens OX=9606 GN=EIF4G2 PE=1 SV=1	0,301703563	5,210931
P54802	Alpha-N-acetylglucosaminidase OS=Homo sapiens OX=9606 GN=NAGLU PE=1 SV=2	-0,616415027	5,208668
P14314	Glucosidase 2 subunit beta OS=Homo sapiens OX=9606 GN=PRKCSH PE=1 SV=2	0,345147078	5,201705
Q96GQ7	Probable ATP-dependent RNA helicase DDX27 OS=Homo sapiens OX=9606 GN=DDX27 PE=1 SV=2	0,941566243	5,149508
P46777	60S ribosomal protein L5 OS=Homo sapiens OX=9606 GN=RPL5 PE=1 SV=3	0,340962114	5,115488
O00203	AP-3 complex subunit beta-1 OS=Homo sapiens OX=9606 GN=AP3B1 PE=1 SV=3	-0,267387857	5,106687
P04179	Superoxide dismutase [Mn], mitochondrial OS=Homo sapiens OX=9606 GN=SOD2 PE=1 SV=3	-0,674135989	5,100064
P78371	T-complex protein 1 subunit beta OS=Homo sapiens OX=9606 GN=CCT2 PE=1 SV=4	0,266467529	5,081454
Q92841	Probable ATP-dependent RNA helicase DDX17 OS=Homo sapiens OX=9606 GN=DDX17 PE=1 SV=2	0,276777081	5,06764
P78318	Immunoglobulin-binding protein 1 OS=Homo sapiens OX=9606 GN=IGBP1 PE=1 SV=1	1,218970547	5,059322
Q9BXF6	Rab11 family-interacting protein 5 OS=Homo sapiens OX=9606 GN=RAB11FIP5 PE=1 SV=1	0,509163178	5,057939
P33316	Deoxyuridine 5'-triphosphate nucleotidohydrolase, mitochondrial OS=Homo sapiens OX=9606 GN=DUT PE=1 SV=4	0,667673117	5,0483
O43252	Bifunctional 3'-phosphoadenosine 5'-phosphosulfate synthase 1 OS=Homo sapiens OX=9606 GN=PAPSS1 PE=1 SV=2	0,360515081	5,042258
P38919	Eukaryotic initiation factor 4A-III OS=Homo sapiens OX=9606 GN=EIF4A3 PE=1 SV=4	0,507449419	5,037439
Q96CV9	Optineurin OS=Homo sapiens OX=9606 GN=OPTN PE=1 SV=3	-0,347951681	5,033818
P53801	Pituitary tumor-transforming gene 1 protein-interacting protein OS=Homo sapiens OX=9606 GN=PTTG1IP PE=1 SV=1	-1,206609568	5,021143
Q9UH65	Switch-associated protein 70 OS=Homo sapiens OX=9606 GN=SWAP70 PE=1 SV=1	0,505307297	5,011814
Q9NTJ5	Phosphatidylinositide phosphatase SAC1 OS=Homo sapiens OX=9606 GN=SACM1L PE=1 SV=2	0,358864955	5,011145
Q02818	Nucleobindin-1 OS=Homo sapiens OX=9606 GN=NUCB1 PE=1 SV=4	-0,315217349	4,976281
P48643	T-complex protein 1 subunit epsilon OS=Homo sapiens OX=9606 GN=CCT5 PE=1 SV=1	0,244182887	4,964566
P34932	Heat shock 70 kDa protein 4 OS=Homo sapiens OX=9606 GN=HSPA4 PE=1 SV=4	0,210716714	4,933427
P07384	Calpain-1 catalytic subunit OS=Homo sapiens OX=9606 GN=CAPN1 PE=1 SV=1	0,235350034	4,930358
Q96JY6	PDZ and LIM domain protein 2 OS=Homo sapiens OX=9606 GN=PDLIM2 PE=1 SV=1	-0,381253687	4,907357
Q9NZ08	Endoplasmic reticulum aminopeptidase 1 OS=Homo sapiens OX=9606 GN=ERAP1 PE=1 SV=3	-0,533907557	4,89335
Q92945	Far upstream element-binding protein 2 OS=Homo sapiens OX=9606 GN=KHSRP PE=1 SV=4	0,310197528	4,866517
O43772	Mitochondrial carnitine/acylcarnitine carrier protein OS=Homo sapiens OX=9606 GN=SLC25A20 PE=1 SV=1	0,881532567	4,85731
O14925	Mitochondrial import inner membrane translocase subunit Tim23 OS=Homo sapiens OX=9606 GN=TIMM23 PE=1 SV=1	0,439510472	4,854218
Q86UY8	5'-nucleotidase domain-containing protein 3 OS=Homo sapiens OX=9606 GN=NT5DC3 PE=1 SV=1	-1,624655511	4,85233
Q9Y6I3	Epsin-1 OS=Homo sapiens OX=9606 GN=EPN1 PE=1 SV=2	0,734367348	4,845172
P11021	Endoplasmic reticulum chaperone BiP OS=Homo sapiens OX=9606 GN=HSPA5 PE=1 SV=2	0,218133877	4,806241
Q13813	Spectrin alpha chain, non-erythrocytic 1 OS=Homo sapiens OX=9606 GN=SPTAN1 PE=1 SV=3	-0,113793363	4,805853
Q5T8P6	RNA-binding protein 26 OS=Homo sapiens OX=9606 GN=RBM26 PE=1 SV=3	0,499627566	4,800282
Q12884	Prolyl endopeptidase FAP OS=Homo sapiens OX=9606 GN=FAP PE=1 SV=5	-0,456440144	4,792228
Q92845	Kinesin-associated protein 3 OS=Homo sapiens OX=9606 GN=KIFAP3 PE=1 SV=2	-1,132808485	4,787065
Q6P996	Pyridoxal-dependent decarboxylase domain-containing protein 1 OS=Homo sapiens OX=9606 GN=PDXDC1 PE=1 SV=2	0,346558188	4,780413
P15586	N-acetylglucosamine-6-sulfatase OS=Homo sapiens OX=9606 GN=GNS PE=1 SV=3	-0,485567036	4,77565
Q9UKX5	Integrin alpha-11 OS=Homo sapiens OX=9606 GN=ITGA11 PE=1 SV=2	-0,265356179	4,772034
P31751	RAC-beta serine/threonine-protein kinase OS=Homo sapiens OX=9606 GN=AKT2 PE=1 SV=2	-0,858832315	4,744013
P61086	Ubiquitin-conjugating enzyme E2 K OS=Homo sapiens OX=9606 GN=UBE2K PE=1 SV=3	0,426115568	4,722954
O60518	Ran-binding protein 6 OS=Homo sapiens OX=9606 GN=RANBP6 PE=1 SV=2	0,852463435	4,711945
Q2NL82	Pre-rRNA-processing protein TSR1 homolog OS=Homo sapiens OX=9606 GN=TSR1 PE=1 SV=1	-0,849754475	4,698268
Q8WTS6	Histone-lysine N-methyltransferase SETD7 OS=Homo sapiens OX=9606 GN=SETD7 PE=1 SV=1	-0,624133393	4,695993
Q04206	Transcription factor p65 OS=Homo sapiens OX=9606 GN=RELA PE=1 SV=2	0,402862377	4,693085
Q13247	Serine/arginine-rich splicing factor 6 OS=Homo sapiens OX=9606 GN=SRSF6 PE=1 SV=2	0,512960573	4,667626
Q9UQ35	Serine/arginine repetitive matrix protein 2 OS=Homo sapiens OX=9606 GN=SRRM2 PE=1 SV=2	0,34008229	4,659973
P17936	Insulin-like growth factor-binding protein 3 OS=Homo sapiens OX=9606 GN=IGFBP3 PE=1 SV=2	-0,557732961	4,653413
P49903	Selenide, water dikinase 1 OS=Homo sapiens OX=9606 GN=SEPHS1 PE=1 SV=2	-0,616330205	4,632227
Q8IZ07	Ankyrin repeat domain-containing protein 13A OS=Homo sapiens OX=9606 GN=ANKRD13A PE=1 SV=3	-0,613558264	4,609532
P49915	GMP synthase [glutamine-hydrolyzing] OS=Homo sapiens OX=9606 GN=GMPS PE=1 SV=1	0,336591201	4,595338
P53396	ATP-citrate synthase OS=Homo sapiens OX=9606 GN=ACLY PE=1 SV=3	0,17299529	4,593979
Q00839	Heterogeneous nuclear ribonucleoprotein U OS=Homo sapiens OX=9606 GN=HNRNPU PE=1 SV=6	0,281816344	4,576068
P78527	DNA-dependent protein kinase catalytic subunit OS=Homo sapiens OX=9606 GN=PRKDC PE=1 SV=3	-0,11450803	4,572523
P06396	Gelsolin OS=Homo sapiens OX=9606 GN=GSN PE=1 SV=1	0,257491695	4,571866
P51178	1-phosphatidylinositol 4,5-bisphosphate phosphodiesterase delta-1 OS=Homo sapiens OX=9606 GN=PLCD1 PE=1 SV=2	-0,466695043	4,550024
P62269	40S ribosomal protein S18 OS=Homo sapiens OX=9606 GN=RPS18 PE=1 SV=3	0,437640495	4,546853
Q15424	Scaffold attachment factor B1 OS=Homo sapiens OX=9606 GN=SAFB PE=1 SV=4	0,437013514	4,538686
P35613	Basigin OS=Homo sapiens OX=9606 GN=BSG PE=1 SV=2	-0,604441204	4,534731
P19623	Spermidine synthase OS=Homo sapiens OX=9606 GN=SRM PE=1 SV=1	-0,374491288	4,531655
Q9UFN0	Protein NipSnap homolog 3A OS=Homo sapiens OX=9606 GN=NIPSNAP3A PE=1 SV=2	-0,812652548	4,508698
P59998	Actin-related protein 2/3 complex subunit 4 OS=Homo sapiens OX=9606 GN=ARPC4 PE=1 SV=3	0,542114899	4,50428
Q9H3S7	Tyrosine-protein phosphatase non-receptor type 23 OS=Homo sapiens OX=9606 GN=PTPN23 PE=1 SV=1	-0,496778687	4,492986
Q9Y3B8	Oligoribonuclease, mitochondrial OS=Homo sapiens OX=9606 GN=REXO2 PE=1 SV=3	-0,433467343	4,492519
Q15063	Periostin OS=Homo sapiens OX=9606 GN=POSTN PE=1 SV=2	-0,496146444	4,48616
P13861	cAMP-dependent protein kinase type II-alpha regulatory subunit OS=Homo sapiens OX=9606 GN=PRKAR2A PE=1 SV=2	-0,341911831	4,470916
Q9BWF3	RNA-binding protein 4 OS=Homo sapiens OX=9606 GN=RBM4 PE=1 SV=1	0,536723187	4,452692
Q9NQX3	Gephyrin OS=Homo sapiens OX=9606 GN=GPHN PE=1 SV=1	-1,157357565	4,449531
Q96QD8	Sodium-coupled neutral amino acid transporter 2 OS=Homo sapiens OX=9606 GN=SLC38A2 PE=1 SV=2	1,61230404	4,420164
Q969X5	Endoplasmic reticulum-Golgi intermediate compartment protein 1 OS=Homo sapiens OX=9606 GN=ERGIC1 PE=1 SV=1	0,455145796	4,412129
Q9NQC3	Reticulon-4 OS=Homo sapiens OX=9606 GN=RTN4 PE=1 SV=2	0,403552982	4,405992
Q69YQ0	Cytospin-A OS=Homo sapiens OX=9606 GN=SPECC1L PE=1 SV=2	0,425583135	4,390028
Q92820	Gamma-glutamyl hydrolase OS=Homo sapiens OX=9606 GN=GGH PE=1 SV=2	-0,586319958	4,385372
A1L0T0	Acetolactate synthase-like protein OS=Homo sapiens OX=9606 GN=ILVBL PE=1 SV=2	0,452893223	4,385257
Q92769	Histone deacetylase 2 OS=Homo sapiens OX=9606 GN=HDAC2 PE=1 SV=2	1,01350254	4,384984
Q15084	Protein disulfide-isomerase A6 OS=Homo sapiens OX=9606 GN=PDIA6 PE=1 SV=1	0,31415583	4,379201
P27824	Calnexin OS=Homo sapiens OX=9606 GN=CANX PE=1 SV=2	0,210188445	4,369415
Q92783	Signal transducing adapter molecule 1 OS=Homo sapiens OX=9606 GN=STAM PE=1 SV=3	-0,484686101	4,362411
Q9NRX2	39S ribosomal protein L17, mitochondrial OS=Homo sapiens OX=9606 GN=MRPL17 PE=1 SV=1	-1,294606889	4,356159
O00170	AH receptor-interacting protein OS=Homo sapiens OX=9606 GN=AIP PE=1 SV=2	0,449805298	4,348433
O60493	Sorting nexin-3 OS=Homo sapiens OX=9606 GN=SNX3 PE=1 SV=3	0,525476376	4,34493
Q14165	Malectin OS=Homo sapiens OX=9606 GN=MLEC PE=1 SV=1	0,361584186	4,327127
Q8N183	NADH dehydrogenase [ubiquinone] 1 alpha subcomplex assembly factor 2 OS=Homo sapiens OX=9606 GN=NDUFAF2 PE=1 SV=1	0,656555749	4,321578
Q9NUP9	Protein lin-7 homolog C OS=Homo sapiens OX=9606 GN=LIN7C PE=1 SV=1	-0,775437409	4,314317
Q9UIW2	Plexin-A1 OS=Homo sapiens OX=9606 GN=PLXNA1 PE=1 SV=3	-0,140011856	4,311923
Q9HA77	Probable cysteine--tRNA ligase, mitochondrial OS=Homo sapiens OX=9606 GN=CARS2 PE=1 SV=1	0,774288338	4,308248
Q9H0U4	Ras-related protein Rab-1B OS=Homo sapiens OX=9606 GN=RAB1B PE=1 SV=1	0,654529949	4,307687
O75874	Isocitrate dehydrogenase [NADP] cytoplasmic OS=Homo sapiens OX=9606 GN=IDH1 PE=1 SV=2	0,331138763	4,282559
P15848	Arylsulfatase B OS=Homo sapiens OX=9606 GN=ARSB PE=1 SV=1	-0,476408644	4,273029

Q6P1X6	UPF0598 protein C8orf82 OS=Homo sapiens OX=9606 GN=C8orf82 PE=1 SV=2	0,430889777	4,267842
Q8WUH6	Transmembrane protein 263 OS=Homo sapiens OX=9606 GN=TMEM263 PE=1 SV=1	0,765795526	4,263264
O00116	Alkyldihydroxyacetonephosphate synthase, peroxisomal OS=Homo sapiens OX=9606 GN=AGPS PE=1 SV=1	0,392460783	4,251204
Q06124	Tyrosine-protein phosphatase non-receptor type 11 OS=Homo sapiens OX=9606 GN=PTPN11 PE=1 SV=2	0,307219821	4,247715
P35222	Catenin beta-1 OS=Homo sapiens OX=9606 GN=CTNNB1 PE=1 SV=1	0,226415952	4,238912
O75116	Rho-associated protein kinase 2 OS=Homo sapiens OX=9606 GN=ROCK2 PE=1 SV=4	-0,222457297	4,236234
P08133	Annexin A6 OS=Homo sapiens OX=9606 GN=ANXA6 PE=1 SV=3	0,164914251	4,221733
Q9UHD8	Septin-9 OS=Homo sapiens OX=9606 GN=SEPTIN9 PE=1 SV=2	0,225764191	4,221441
P41252	Isoleucine--tRNA ligase, cytoplasmic OS=Homo sapiens OX=9606 GN=IARS PE=1 SV=2	-0,189153994	4,21098
P30154	Serine/threonine-protein phosphatase 2A 65 kDa regulatory subunit A beta isoform OS=Homo sapiens OX=9606 GN=PPP2R1B PE=1 SV=	-0,370400663	4,207688
O00232	26S proteasome non-ATPase regulatory subunit 12 OS=Homo sapiens OX=9606 GN=PSMD12 PE=1 SV=3	-0,315232586	4,204651
Q9NR50	Translation initiation factor eIF-2B subunit gamma OS=Homo sapiens OX=9606 GN=EIF2B3 PE=1 SV=1	0,411099094	4,202356
Q99700	Ataxin-2 OS=Homo sapiens OX=9606 GN=ATXN2 PE=1 SV=2	0,436978331	4,195675
Q5EBL4	RILP-like protein 1 OS=Homo sapiens OX=9606 GN=RILPL1 PE=1 SV=1	0,563403176	4,195287
Q8WU90	Zinc finger CCCH domain-containing protein 15 OS=Homo sapiens OX=9606 GN=ZC3H15 PE=1 SV=1	0,509491141	4,191466
Q16850	Lanosterol 14-alpha demethylase OS=Homo sapiens OX=9606 GN=CYP51A1 PE=1 SV=3	-0,561126681	4,176337
Q9HDC9	Adipocyte plasma membrane-associated protein OS=Homo sapiens OX=9606 GN=APMAP PE=1 SV=2	0,336948128	4,163987
Q9Y6C2	EMILIN-1 OS=Homo sapiens OX=9606 GN=EMILIN1 PE=1 SV=3	0,263452489	4,163705
Q9HAU4	E3 ubiquitin-protein ligase SMURF2 OS=Homo sapiens OX=9606 GN=SMURF2 PE=1 SV=1	-0,631810846	4,151042
Q8WUJ3	Cell migration-inducing and hyaluronan-binding protein OS=Homo sapiens OX=9606 GN=CEMIP PE=1 SV=2	0,245235914	4,142053
Q8NBJ5	Procollagen galactosyltransferase 1 OS=Homo sapiens OX=9606 GN=COLGALT1 PE=1 SV=1	0,291846288	4,131685
P15170	Eukaryotic peptide chain release factor GTP-binding subunit ERF3A OS=Homo sapiens OX=9606 GN=GSPT1 PE=1 SV=1	0,311173073	4,131375
Q96BY6	Dedicator of cytokinesis protein 10 OS=Homo sapiens OX=9606 GN=DOCK10 PE=1 SV=3	0,431234452	4,127392
O60502	Protein O-GlcNAcase OS=Homo sapiens OX=9606 GN=OGA PE=1 SV=2	-0,430747752	4,12161
P68402	Platelet-activating factor acetylhydrolase IB subunit beta OS=Homo sapiens OX=9606 GN=PAFAH1B2 PE=1 SV=1	0,553898546	4,116092
Q14439	G-protein coupled receptor 176 OS=Homo sapiens OX=9606 GN=GPR176 PE=2 SV=1	0,488026932	4,101284
P62899	60S ribosomal protein L31 OS=Homo sapiens OX=9606 GN=RPL31 PE=1 SV=1	0,619472472	4,065331
Q04446	1,4-alpha-glucan-branching enzyme OS=Homo sapiens OX=9606 GN=GBE1 PE=1 SV=3	0,223809523	4,064681
Q9NX62	Inositol monophosphatase 3 OS=Homo sapiens OX=9606 GN=IMPAD1 PE=1 SV=1	-0,726618333	4,052907
P67809	Nuclease-sensitive element-binding protein 1 OS=Homo sapiens OX=9606 GN=YBX1 PE=1 SV=3	0,398475377	4,039545
Q9NZM1	Myoferlin OS=Homo sapiens OX=9606 GN=MYOF PE=1 SV=1	-0,112907365	4,03368
Q03013	Glutathione S-transferase Mu 4 OS=Homo sapiens OX=9606 GN=GSTM4 PE=1 SV=3	0,491906702	4,022346
P19793	Retinoic acid receptor RXR-alpha OS=Homo sapiens OX=9606 GN=RXRA PE=1 SV=1	0,577757568	4,017954
Q92839	Hyaluronan synthase 1 OS=Homo sapiens OX=9606 GN=HAS1 PE=1 SV=2	0,452608678	4,016208
P55957	BH3-interacting domain death agonist OS=Homo sapiens OX=9606 GN=BID PE=1 SV=1	0,612050158	4,013562
O60256	Phosphoribosyl pyrophosphate synthase-associated protein 2 OS=Homo sapiens OX=9606 GN=PRPSAP2 PE=1 SV=1	-0,717610015	4,00388
P46781	40S ribosomal protein S9 OS=Homo sapiens OX=9606 GN=RPS9 PE=1 SV=3	0,314375737	3,99331
P15104	Glutamine synthetase OS=Homo sapiens OX=9606 GN=GLUL PE=1 SV=4	-0,708666408	3,954964
Q8N6H7	ADP-ribosylation factor GTPase-activating protein 2 OS=Homo sapiens OX=9606 GN=ARFGAP2 PE=1 SV=1	-0,446561052	3,951032
P13804	Electron transfer flavoprotein subunit alpha, mitochondrial OS=Homo sapiens OX=9606 GN=ETFA PE=1 SV=1	0,311775673	3,948889
P05120	Plasminogen activator inhibitor 2 OS=Homo sapiens OX=9606 GN=SERPINB2 PE=1 SV=2	-0,3001931	3,934858
P23246	Splicing factor, proline- and glutamine-rich OS=Homo sapiens OX=9606 GN=SFPQ PE=1 SV=2	0,272900625	3,921141
Q9ULI3	Protein HEG homolog 1 OS=Homo sapiens OX=9606 GN=HEG1 PE=1 SV=3	0,093873958	3,917097
P27797	Calreticulin OS=Homo sapiens OX=9606 GN=CALR PE=1 SV=1	0,235455168	3,910783
Q7Z460	CLIP-associating protein 1 OS=Homo sapiens OX=9606 GN=CLASP1 PE=1 SV=1	0,528517676	3,903714
Q8WZA9	Immunity-related GTPase family Q protein OS=Homo sapiens OX=9606 GN=IRGQ PE=1 SV=1	0,477688257	3,885459
Q9HD45	Transmembrane 9 superfamily member 3 OS=Homo sapiens OX=9606 GN=TM9SF3 PE=1 SV=2	0,439817672	3,878416
P52788	Spermine synthase OS=Homo sapiens OX=9606 GN=SMS PE=1 SV=2	0,365464106	3,877988
Q15393	Splicing factor 3B subunit 3 OS=Homo sapiens OX=9606 GN=SF3B3 PE=1 SV=4	0,270539573	3,874418
Q8WXF1	Paraspeckle component 1 OS=Homo sapiens OX=9606 GN=PSPC1 PE=1 SV=1	-0,524836469	3,872814
Q16881	Thioredoxin reductase 1, cytoplasmic OS=Homo sapiens OX=9606 GN=TXNRD1 PE=1 SV=3	-0,256322148	3,871114
O00299	Chloride intracellular channel protein 1 OS=Homo sapiens OX=9606 GN=CLIC1 PE=1 SV=4	-0,26994152	3,86261
P46778	60S ribosomal protein L21 OS=Homo sapiens OX=9606 GN=RPL21 PE=1 SV=2	-0,5226457	3,854414
O60218	Aldo-keto reductase family 1 member B10 OS=Homo sapiens OX=9606 GN=AKR1B10 PE=1 SV=2	0,766268274	3,849445
O60763	General vesicular transport factor p115 OS=Homo sapiens OX=9606 GN=USO1 PE=1 SV=2	0,21425913	3,820018
Q9BQE3	Tubulin alpha-1C chain OS=Homo sapiens OX=9606 GN=TUBA1C PE=1 SV=1	0,683606778	3,816635
P49770	Translation initiation factor eIF-2B subunit beta OS=Homo sapiens OX=9606 GN=EIF2B2 PE=1 SV=3	0,470473807	3,815981
Q9Y314	Nitric oxide synthase-interacting protein OS=Homo sapiens OX=9606 GN=NOSIP PE=1 SV=1	0,682328428	3,809528
Q9UBP6	tRNA (guanine-N(7)-)-methyltransferase OS=Homo sapiens OX=9606 GN=METTL1 PE=1 SV=1	-0,735546324	3,794525
O43617	Trafficking protein particle complex subunit 3 OS=Homo sapiens OX=9606 GN=TRAPPC3 PE=1 SV=1	0,579711077	3,786261
P08670	Vimentin OS=Homo sapiens OX=9606 GN=VIM PE=1 SV=4	0,140607369	3,786097
O95232	Luc7-like protein 3 OS=Homo sapiens OX=9606 GN=LUC7L3 PE=1 SV=2	0,850903194	3,784829
Q9Y4P1	Cysteine protease ATG4B OS=Homo sapiens OX=9606 GN=ATG4B PE=1 SV=2	0,677523536	3,782776
Q9Y2Y0	ADP-ribosylation factor-like protein 2-binding protein OS=Homo sapiens OX=9606 GN=ARL2BP PE=1 SV=1	-0,850112232	3,781752
P62857	40S ribosomal protein S28 OS=Homo sapiens OX=9606 GN=RPS28 PE=1 SV=1	0,677068873	3,780241
P49411	Elongation factor Tu, mitochondrial OS=Homo sapiens OX=9606 GN=TUFM PE=1 SV=2	0,281830219	3,775786
P04150	Glucocorticoid receptor OS=Homo sapiens OX=9606 GN=NR3C1 PE=1 SV=1	-0,377849549	3,775355
Q6ZMZ3	Nesprin-3 OS=Homo sapiens OX=9606 GN=SYNE3 PE=1 SV=2	-1,26011945	3,774474
Q16740	ATP-dependent Clp protease proteolytic subunit, mitochondrial OS=Homo sapiens OX=9606 GN=CLPP PE=1 SV=1	0,57803748	3,774424
Q9Y6G9	Cytoplasmic dynein 1 light intermediate chain 1 OS=Homo sapiens OX=9606 GN=DYNC1LI1 PE=1 SV=3	0,312843958	3,770956
Q13630	GDP-L-fucose synthase OS=Homo sapiens OX=9606 GN=TSTA3 PE=1 SV=1	-0,511871858	3,763821
P51911	Calponin-1 OS=Homo sapiens OX=9606 GN=CNN1 PE=1 SV=2	0,465015079	3,763412
P32929	Cystathionine gamma-lyase OS=Homo sapiens OX=9606 GN=CTH PE=1 SV=3	-0,844291798	3,75906
O94855	Protein transport protein Sec24D OS=Homo sapiens OX=9606 GN=SEC24D PE=1 SV=2	-0,239161686	3,758484
P35998	26S proteasome regulatory subunit 7 OS=Homo sapiens OX=9606 GN=PSMC2 PE=1 SV=3	0,224206849	3,757465
Q15582	Transforming growth factor-beta-induced protein ig-h3 OS=Homo sapiens OX=9606 GN=TGFBI PE=1 SV=1	-0,219106265	3,740112
P31689	DnaJ homolog subfamily A member 1 OS=Homo sapiens OX=9606 GN=DNAJA1 PE=1 SV=2	0,398477709	3,739927
O00754	Lysosomal alpha-mannosidase OS=Homo sapiens OX=9606 GN=MAN2B1 PE=1 SV=3	-0,397579497	3,729359
P07099	Epoxide hydrolase 1 OS=Homo sapiens OX=9606 GN=EPHX1 PE=1 SV=1	0,288538309	3,729076
Q5VTQ0	Tetratricopeptide repeat protein 39B OS=Homo sapiens OX=9606 GN=TTC39B PE=1 SV=4	-1,234854141	3,723078
P49757	Protein numb homolog OS=Homo sapiens OX=9606 GN=NUMB PE=1 SV=2	-0,835030552	3,722775
Q5SSJ5	Heterochromatin protein 1-binding protein 3 OS=Homo sapiens OX=9606 GN=HP1BP3 PE=1 SV=1	-0,37364503	3,721817
Q6NZY4	Zinc finger CCHC domain-containing protein 8 OS=Homo sapiens OX=9606 GN=ZCCHC8 PE=1 SV=2	-0,507114049	3,720005
P84101	Small EDRK-rich factor 2 OS=Homo sapiens OX=9606 GN=SERF2 PE=1 SV=1	0,663721363	3,705555
Q13838	Spliceosome RNA helicase DDX39B OS=Homo sapiens OX=9606 GN=DDX39B PE=1 SV=1	0,352787239	3,704741
Q9BQ61	Telomerase RNA component interacting RNase OS=Homo sapiens OX=9606 GN=TRIR PE=1 SV=1	0,663065897	3,701875
Q96JB5	CDK5 regulatory subunit-associated protein 3 OS=Homo sapiens OX=9606 GN=CDK5RAP3 PE=1 SV=2	-0,335391865	3,695027
P61224	Ras-related protein Rap-1b OS=Homo sapiens OX=9606 GN=RAP1B PE=1 SV=1	0,422502311	3,69232
Q9P2Q2	FERM domain-containing protein 4A OS=Homo sapiens OX=9606 GN=FRMD4A PE=1 SV=3	0,888418434	3,683772
Q7Z7M9	Polypeptide N-acetylgalactosaminyltransferase 5 OS=Homo sapiens OX=9606 GN=GALNT5 PE=1 SV=1	0,369120009	3,664328
O15357	Phosphatidylinositol 3,4,5-trisphosphate 5-phosphatase 2 OS=Homo sapiens OX=9606 GN=INPPL1 PE=1 SV=2	0,655696021	3,660403
P46087	Probable 28S rRNA (cytosine(4447)-C(5))-methyltransferase OS=Homo sapiens OX=9606 GN=NOP2 PE=1 SV=2	0,419207759	3,656983
P35611	Alpha-adducin OS=Homo sapiens OX=9606 GN=ADD1 PE=1 SV=2	0,229339052	3,656924
P21810	Biglycan OS=Homo sapiens OX=9606 GN=BGN PE=1 SV=2	0,498050972	3,647378
P98179	RNA-binding protein 3 OS=Homo sapiens OX=9606 GN=RBM3 PE=1 SV=1	-0,497120742	3,639533
Q12888	TP53-binding protein 1 OS=Homo sapiens OX=9606 GN=TP53BP1 PE=1 SV=2	0,293227576	3,635729
Q07065	Cytoskeleton-associated protein 4 OS=Homo sapiens OX=9606 GN=CKAP4 PE=1 SV=2	0,187673657	3,634321
Q96HC4	PDZ and LIM domain protein 5 OS=Homo sapiens OX=9606 GN=PDLIM5 PE=1 SV=5	0,206778217	3,632251

Q8TD16	Protein bicaudal D homolog 2 OS=Homo sapiens OX=9606 GN=BICD2 PE=1 SV=1	0,330188785	3,619987
Q9NZW5	MAGUK p55 subfamily member 6 OS=Homo sapiens OX=9606 GN=MPP6 PE=1 SV=2	-0,806262938	3,608673
P04080	Cystatin-B OS=Homo sapiens OX=9606 GN=CSTB PE=1 SV=2	-0,644648863	3,597951
Q15599	Na(+)/H(+) exchange regulatory cofactor NHE-RF2 OS=Homo sapiens OX=9606 GN=SLC9A3R2 PE=1 SV=2	0,413410621	3,59487
Q8WX93	Palladin OS=Homo sapiens OX=9606 GN=PALLD PE=1 SV=3	0,186219486	3,594174
Q9ULX6	A-kinase anchor protein 8-like OS=Homo sapiens OX=9606 GN=AKAP8L PE=1 SV=4	1,123578953	3,588139
P21399	Cytoplasmic aconitate hydratase OS=Homo sapiens OX=9606 GN=ACO1 PE=1 SV=3	0,312933657	3,574126
O43795	Unconventional myosin-Ib OS=Homo sapiens OX=9606 GN=MYO1B PE=1 SV=3	-0,177781469	3,573512
P48163	NADP-dependent malic enzyme OS=Homo sapiens OX=9606 GN=ME1 PE=1 SV=1	-0,326777202	3,570963
P49748	Very long-chain specific acyl-CoA dehydrogenase, mitochondrial OS=Homo sapiens OX=9606 GN=ACADVL PE=1 SV=1	-0,207805755	3,564743
O94979	Protein transport protein Sec31A OS=Homo sapiens OX=9606 GN=SEC31A PE=1 SV=3	0,182094752	3,553939
Q16512	Serine/threonine-protein kinase N1 OS=Homo sapiens OX=9606 GN=PKN1 PE=1 SV=2	-0,724540449	3,539533
Q9BY42	Replication termination factor 2 OS=Homo sapiens OX=9606 GN=RTF2 PE=1 SV=3	1,400908612	3,521601
Q9Y4G6	Talin-2 OS=Homo sapiens OX=9606 GN=TLN2 PE=1 SV=4	-0,252179302	3,516987
Q9BYX2	TBC1 domain family member 2A OS=Homo sapiens OX=9606 GN=TBC1D2 PE=1 SV=3	-0,439032595	3,513345
Q5T5C0	Syntaxin-binding protein 5 OS=Homo sapiens OX=9606 GN=STXBP5 PE=1 SV=1	0,540789521	3,509294
Q01581	Hydroxymethylglutaryl-CoA synthase, cytoplasmic OS=Homo sapiens OX=9606 GN=HMGCS1 PE=1 SV=2	-1,510886329	3,506238
O75844	CAAX prenyl protease 1 homolog OS=Homo sapiens OX=9606 GN=ZMPSTE24 PE=1 SV=2	0,337399803	3,496446
Q10588	ADP-ribosyl cyclase/cyclic ADP-ribose hydrolase 2 OS=Homo sapiens OX=9606 GN=BST1 PE=1 SV=2	-0,626659629	3,495525
Q9NX20	39S ribosomal protein L16, mitochondrial OS=Homo sapiens OX=9606 GN=MRPL16 PE=1 SV=1	1,097726088	3,491797
P10915	Hyaluronan and proteoglycan link protein 1 OS=Homo sapiens OX=9606 GN=HAPLN1 PE=2 SV=2	-0,336934066	3,490178
P35580	Myosin-10 OS=Homo sapiens OX=9606 GN=MYH10 PE=1 SV=3	0,160352523	3,489874
Q86VZ4	Low-density lipoprotein receptor-related protein 11 OS=Homo sapiens OX=9606 GN=LRP11 PE=2 SV=2	-1,012453743	3,489618
P53814	Smoothelin OS=Homo sapiens OX=9606 GN=SMTN PE=1 SV=7	0,307173405	3,48715
P35270	Sepiapterin reductase OS=Homo sapiens OX=9606 GN=SPR PE=1 SV=1	0,537623654	3,486621
P40123	Adenylyl cyclase-associated protein 2 OS=Homo sapiens OX=9606 GN=CAP2 PE=1 SV=1	0,354630779	3,48123
P68104	Elongation factor 1-alpha 1 OS=Homo sapiens OX=9606 GN=EEF1A1 PE=1 SV=1	0,265395926	3,478841
P50454	Serpin H1 OS=Homo sapiens OX=9606 GN=SERPINH1 PE=1 SV=2	0,212116666	3,475635
P55786	Puromycin-sensitive aminopeptidase OS=Homo sapiens OX=9606 GN=NPEPPS PE=1 SV=2	0,200399785	3,474909
P04843	Dolichyl-diphosphooligosaccharide--protein glycosyltransferase subunit 1 OS=Homo sapiens OX=9606 GN=RPN1 PE=1 SV=1	0,196609388	3,467307
P49593	Protein phosphatase 1F OS=Homo sapiens OX=9606 GN=PPM1F PE=1 SV=3	0,534398497	3,463503
P31153	S-adenosylmethionine synthase isoform type-2 OS=Homo sapiens OX=9606 GN=MAT2A PE=1 SV=1	0,318962258	3,459218
Q99426	Tubulin-folding cofactor B OS=Homo sapiens OX=9606 GN=TBCB PE=1 SV=2	0,400447424	3,456316
Q99598	Translin-associated protein X OS=Homo sapiens OX=9606 GN=TSNAX PE=1 SV=1	-0,76616247	3,446064
Q8IX30	Signal peptide, CUB and EGF-like domain-containing protein 3 OS=Homo sapiens OX=9606 GN=SCUBE3 PE=1 SV=1	-0,292142748	3,441126
P30279	G1/S-specific cyclin-D2 OS=Homo sapiens OX=9606 GN=CCND2 PE=1 SV=1	0,736876398	3,434588
O15372	Eukaryotic translation initiation factor 3 subunit H OS=Homo sapiens OX=9606 GN=EIF3H PE=1 SV=1	0,2712093	3,428837
O75369	Filamin-B OS=Homo sapiens OX=9606 GN=FLNB PE=1 SV=2	-0,100192658	3,427645
P39023	60S ribosomal protein L3 OS=Homo sapiens OX=9606 GN=RPL3 PE=1 SV=2	0,262510113	3,42744
Q15057	Arf-GAP with coiled-coil, ANK repeat and PH domain-containing protein 2 OS=Homo sapiens OX=9606 GN=ACAP2 PE=1 SV=3	0,427993606	3,407272
P00338	L-lactate dehydrogenase A chain OS=Homo sapiens OX=9606 GN=LDHA PE=1 SV=2	0,227943986	3,402784
O60762	Dolichol-phosphate mannosyltransferase subunit 1 OS=Homo sapiens OX=9606 GN=DPM1 PE=1 SV=1	0,467818071	3,392023
Q6UB35	Monofunctional C1-tetrahydrofolate synthase, mitochondrial OS=Homo sapiens OX=9606 GN=MTHFD1L PE=1 SV=1	-0,277868906	3,381691
Q13740	CD166 antigen OS=Homo sapiens OX=9606 GN=ALCAM PE=1 SV=2	0,267478548	3,365143
P16615	Sarcoplasmic/endoplasmic reticulum calcium ATPase 2 OS=Homo sapiens OX=9606 GN=ATP2A2 PE=1 SV=1	-0,170293516	3,360616
Q8TEX9	Importin-4 OS=Homo sapiens OX=9606 GN=IPO4 PE=1 SV=2	-0,286818524	3,35756
P50570	Dynamin-2 OS=Homo sapiens OX=9606 GN=DNM2 PE=1 SV=2	0,195231975	3,349324
O00469	Procollagen-lysine,2-oxoglutarate 5-dioxygenase 2 OS=Homo sapiens OX=9606 GN=PLOD2 PE=1 SV=2	0,195086694	3,345818
O14907	Tax1-binding protein 3 OS=Homo sapiens OX=9606 GN=TAX1BP3 PE=1 SV=2	1,060226872	3,3346
Q9UHW9	Solute carrier family 12 member 6 OS=Homo sapiens OX=9606 GN=SLC12A6 PE=1 SV=2	-0,823473375	3,334341
Q8TAQ2	SWI/SNF complex subunit SMARCC2 OS=Homo sapiens OX=9606 GN=SMARCC2 PE=1 SV=1	0,515535016	3,3279
P00558	Phosphoglycerate kinase 1 OS=Homo sapiens OX=9606 GN=PGK1 PE=1 SV=3	0,205559239	3,326076
P29966	Myristoylated alanine-rich C-kinase substrate OS=Homo sapiens OX=9606 GN=MARCKS PE=1 SV=4	0,324596122	3,324973
Q5JTV8	Torsin-1A-interacting protein 1 OS=Homo sapiens OX=9606 GN=TOR1AIP1 PE=1 SV=2	0,309484963	3,324783
P52294	Importin subunit alpha-5 OS=Homo sapiens OX=9606 GN=KPNA1 PE=1 SV=3	0,736674239	3,323822
O95340	Bifunctional 3'-phosphoadenosine 5'-phosphosulfate synthase 2 OS=Homo sapiens OX=9606 GN=PAPSS2 PE=1 SV=2	0,2008739	3,309786
P38606	V-type proton ATPase catalytic subunit A OS=Homo sapiens OX=9606 GN=ATP6V1A PE=1 SV=2	-0,213424964	3,307539
O43847	Nardilysin OS=Homo sapiens OX=9606 GN=NRDC PE=1 SV=3	-0,262970255	3,288639
Q9NRV9	Heme-binding protein 1 OS=Homo sapiens OX=9606 GN=HEBP1 PE=1 SV=1	0,338731322	3,282211
Q2TAA2	Isoamyl acetate-hydrolyzing esterase 1 homolog OS=Homo sapiens OX=9606 GN=IAH1 PE=1 SV=1	-0,587890465	3,271836
Q03518	Antigen peptide transporter 1 OS=Homo sapiens OX=9606 GN=TAP1 PE=1 SV=2	0,724164385	3,271278
P35579	Myosin-9 OS=Homo sapiens OX=9606 GN=MYH9 PE=1 SV=4	0,091383769	3,270503
Q13557	Calcium/calmodulin-dependent protein kinase type II subunit delta OS=Homo sapiens OX=9606 GN=CAMK2D PE=1 SV=3	0,239001117	3,267201
Q08211	ATP-dependent RNA helicase A OS=Homo sapiens OX=9606 GN=DHX9 PE=1 SV=4	0,164557804	3,261916
P10253	Lysosomal alpha-glucosidase OS=Homo sapiens OX=9606 GN=GAA PE=1 SV=4	0,412152768	3,255361
P43490	Nicotinamide phosphoribosyltransferase OS=Homo sapiens OX=9606 GN=NAMPT PE=1 SV=1	0,336412041	3,25336
Q16718	NADH dehydrogenase [ubiquinone] 1 alpha subcomplex subunit 5 OS=Homo sapiens OX=9606 GN=NDUFA5 PE=1 SV=3	0,584530103	3,252266
Q03252	Lamin-B2 OS=Homo sapiens OX=9606 GN=LMNB2 PE=1 SV=4	0,226032185	3,252121
P16070	CD44 antigen OS=Homo sapiens OX=9606 GN=CD44 PE=1 SV=3	0,318709389	3,246744
O00291	Huntingtin-interacting protein 1 OS=Homo sapiens OX=9606 GN=HIP1 PE=1 SV=5	0,279108914	3,237529
Q9ULC4	Malignant T-cell-amplified sequence 1 OS=Homo sapiens OX=9606 GN=MCTS1 PE=1 SV=1	0,449422574	3,236436
Q8TF66	Leucine-rich repeat-containing protein 15 OS=Homo sapiens OX=9606 GN=LRRC15 PE=2 SV=2	0,335038945	3,236303
Q9Y277	Voltage-dependent anion-selective channel protein 3 OS=Homo sapiens OX=9606 GN=VDAC3 PE=1 SV=1	0,35490949	3,231973
O00592	Podocalyxin OS=Homo sapiens OX=9606 GN=PODXL PE=1 SV=2	0,714628381	3,230951
Q96S52	GPI transamidase component PIG-S OS=Homo sapiens OX=9606 GN=PIGS PE=1 SV=3	0,408726386	3,222562
O60814	Histone H2B type 1-K OS=Homo sapiens OX=9606 GN=HIST1H2BK PE=1 SV=3	0,447702013	3,221883
Q6P1J9	Parafibromin OS=Homo sapiens OX=9606 GN=CDC73 PE=1 SV=1	0,578740422	3,218483
O76003	Glutaredoxin-3 OS=Homo sapiens OX=9606 GN=GLRX3 PE=1 SV=2	0,333058929	3,211736
Q9Y4Z0	U6 snRNA-associated Sm-like protein LSm4 OS=Homo sapiens OX=9606 GN=LSM4 PE=1 SV=1	0,709023027	3,207136
Q07666	KH domain-containing, RNA-binding, signal transduction-associated protein 1 OS=Homo sapiens OX=9606 GN=KHDRBS1 PE=1 SV=1	0,445899042	3,206633
P43686	26S proteasome regulatory subunit 6B OS=Homo sapiens OX=9606 GN=PSMC4 PE=1 SV=2	0,21789671	3,196932
Q9Y4D7	Plexin-D1 OS=Homo sapiens OX=9606 GN=PLXND1 PE=1 SV=3	0,24188478	3,194692
Q6NYC1	Bifunctional arginine demethylase and lysyl-hydroxylase JMJD6 OS=Homo sapiens OX=9606 GN=JMJD6 PE=1 SV=1	1,00059173	3,192756
O75935	Dynactin subunit 3 OS=Homo sapiens OX=9606 GN=DCTN3 PE=1 SV=1	0,403711164	3,174598
Q99707	Methionine synthase OS=Homo sapiens OX=9606 GN=MTR PE=1 SV=2	-0,733518279	3,174066
O94874	E3 UFM1-protein ligase 1 OS=Homo sapiens OX=9606 GN=UFL1 PE=1 SV=2	-0,255856984	3,168987
Q9Y394	Dehydrogenase/reductase SDR family member 7 OS=Homo sapiens OX=9606 GN=DHRS7 PE=1 SV=1	0,440268855	3,159016
Q7Z4W1	L-xylulose reductase OS=Homo sapiens OX=9606 GN=DCXR PE=1 SV=2	-0,491764524	3,156195
P30101	Protein disulfide-isomerase A3 OS=Homo sapiens OX=9606 GN=PDIA3 PE=1 SV=4	0,180173716	3,140024
Q9BWD1	Acetyl-CoA acetyltransferase, cytosolic OS=Homo sapiens OX=9606 GN=ACAT2 PE=1 SV=2	0,370250906	3,13586
Q14997	Proteasome activator complex subunit 4 OS=Homo sapiens OX=9606 GN=PSME4 PE=1 SV=2	-0,488143488	3,12997
Q9UH62	Armadillo repeat-containing X-linked protein 3 OS=Homo sapiens OX=9606 GN=ARMCX3 PE=1 SV=1	0,325794347	3,121916
Q8NBJ7	Inactive C-alpha-formylglycine-generating enzyme 2 OS=Homo sapiens OX=9606 GN=SUMF2 PE=1 SV=2	-0,435652464	3,119981
Q9UBI6	Guanine nucleotide-binding protein G(I)/G(S)/G(O) subunit gamma-12 OS=Homo sapiens OX=9606 GN=GNG12 PE=1 SV=3	0,964408413	3,103789
O60610	Protein diaphanous homolog 1 OS=Homo sapiens OX=9606 GN=DIAPH1 PE=1 SV=2	0,218406459	3,102001
Q13310	Polyadenylate-binding protein 4 OS=Homo sapiens OX=9606 GN=PABPC4 PE=1 SV=1	0,270053802	3,098074
Q13546	Receptor-interacting serine/threonine-protein kinase 1 OS=Homo sapiens OX=9606 GN=RIPK1 PE=1 SV=3	0,483596303	3,097015
Q8IY17	Neuropathy target esterase OS=Homo sapiens OX=9606 GN=PNPLA6 PE=1 SV=3	0,366542026	3,096767

Q7KZF4	Staphylococcal nuclease domain-containing protein 1 OS=Homo sapiens OX=9606 GN=SND1 PE=1 SV=1	0,145783639	3,095065
P02511	Alpha-crystallin B chain OS=Homo sapiens OX=9606 GN=CRYAB PE=1 SV=2	0,250835806	3,085325
Q9HD26	Golgi-associated PDZ and coiled-coil motif-containing protein OS=Homo sapiens OX=9606 GN=GOPC PE=1 SV=1	0,341339194	3,076106
Q5GLZ8	Probable E3 ubiquitin-protein ligase HERC4 OS=Homo sapiens OX=9606 GN=HERC4 PE=1 SV=1	-0,194141062	3,071359
P49756	RNA-binding protein 25 OS=Homo sapiens OX=9606 GN=RBM25 PE=1 SV=3	0,305151177	3,068133
Q9NYL4	Peptidyl-prolyl cis-trans isomerase FKBP11 OS=Homo sapiens OX=9606 GN=FKBP11 PE=1 SV=1	-0,478437238	3,059597
Q9NV70	Exocyst complex component 1 OS=Homo sapiens OX=9606 GN=EXOC1 PE=1 SV=4	0,42834883	3,058245
Q8IXM2	Chromatin complexes subunit BAP18 OS=Homo sapiens OX=9606 GN=BAP18 PE=1 SV=1	-0,870102668	3,057001
Q9Y678	Coatomer subunit gamma-1 OS=Homo sapiens OX=9606 GN=COPG1 PE=1 SV=1	0,142879042	3,054945
O60841	Eukaryotic translation initiation factor 5B OS=Homo sapiens OX=9606 GN=EIF5B PE=1 SV=4	0,221102817	3,046648
O15270	Serine palmitoyltransferase 2 OS=Homo sapiens OX=9606 GN=SPTLC2 PE=1 SV=1	-0,426136669	3,039552
P22692	Insulin-like growth factor-binding protein 4 OS=Homo sapiens OX=9606 GN=IGFBP4 PE=1 SV=2	0,475347381	3,037173
Q2TAA5	GDP-Man:Man(3)GlcNAc(2)-PP-Dol alpha-1,2-mannosyltransferase OS=Homo sapiens OX=9606 GN=ALG11 PE=1 SV=2	-0,382588869	3,035292
Q9GZM5	Protein YIPF3 OS=Homo sapiens OX=9606 GN=YIPF3 PE=1 SV=1	0,935900029	3,032083
P17987	T-complex protein 1 subunit alpha OS=Homo sapiens OX=9606 GN=TCP1 PE=1 SV=1	0,175729938	3,032066
Q9P2B4	CTTNBP2 N-terminal-like protein OS=Homo sapiens OX=9606 GN=CTTNBP2NL PE=1 SV=2	0,318311597	3,029935
Q15365	Poly(rC)-binding protein 1 OS=Homo sapiens OX=9606 GN=PCBP1 PE=1 SV=2	0,318102639	3,027375
Q5JPE7	Nodal modulator 2 OS=Homo sapiens OX=9606 GN=NOMO2 PE=1 SV=1	0,188134057	3,02058
P26599	Polypyrimidine tract-binding protein 1 OS=Homo sapiens OX=9606 GN=PTBP1 PE=1 SV=1	0,232030552	3,018825
Q9Y217	Myotubularin-related protein 6 OS=Homo sapiens OX=9606 GN=MTMR6 PE=1 SV=3	-0,541760152	3,000847
Q9UK45	U6 snRNA-associated Sm-like protein LSm7 OS=Homo sapiens OX=9606 GN=LSM7 PE=1 SV=1	0,541733921	3,000691
Q96F86	Enhancer of mRNA-decapping protein 3 OS=Homo sapiens OX=9606 GN=EDC3 PE=1 SV=1	0,660211399	2,996309
Q9GZT8	NIF3-like protein 1 OS=Homo sapiens OX=9606 GN=NIF3L1 PE=1 SV=2	-0,658923442	2,990663
P67812	Signal peptidase complex catalytic subunit SEC11A OS=Homo sapiens OX=9606 GN=SEC11A PE=1 SV=1	0,419838957	2,986359
P07339	Cathepsin D OS=Homo sapiens OX=9606 GN=CTSD PE=1 SV=1	-0,230187026	2,986278
Q8IVF2	Protein AHNAK2 OS=Homo sapiens OX=9606 GN=AHNAK2 PE=1 SV=2	-0,244716635	2,984284
Q13405	39S ribosomal protein L49, mitochondrial OS=Homo sapiens OX=9606 GN=MRPL49 PE=1 SV=1	-0,916959958	2,983637
Q8NHV4	Protein NEDD1 OS=Homo sapiens OX=9606 GN=NEDD1 PE=1 SV=1	0,229149174	2,96663
Q9NQW6	Anillin OS=Homo sapiens OX=9606 GN=ANLN PE=1 SV=2	0,354135476	2,966495
P49753	Acyl-coenzyme A thioesterase 2, mitochondrial OS=Homo sapiens OX=9606 GN=ACOT2 PE=1 SV=6	-1,389756853	2,965842
Q9BYT8	Neurolysin, mitochondrial OS=Homo sapiens OX=9606 GN=NLN PE=1 SV=1	-0,283559948	2,963641
P27694	Replication protein A 70 kDa DNA-binding subunit OS=Homo sapiens OX=9606 GN=RPA1 PE=1 SV=2	-0,297135336	2,963623
P36542	ATP synthase subunit gamma, mitochondrial OS=Homo sapiens OX=9606 GN=ATP5F1C PE=1 SV=1	0,330970398	2,957922
Q6PD74	Alpha- and gamma-adaptin-binding protein p34 OS=Homo sapiens OX=9606 GN=AAGAB PE=1 SV=1	-1,563931893	2,952116
Q16678	Cytochrome P450 1B1 OS=Homo sapiens OX=9606 GN=CYP1B1 PE=1 SV=2	-0,330220928	2,949412
P51809	Vesicle-associated membrane protein 7 OS=Homo sapiens OX=9606 GN=VAMP7 PE=1 SV=3	0,649351733	2,948571
Q9C0C9	(E3-independent) E2 ubiquitin-conjugating enzyme OS=Homo sapiens OX=9606 GN=UBE2O PE=1 SV=3	0,462690553	2,945231
Q14123	Calcium/calmodulin-dependent 3',5'-cyclic nucleotide phosphodiesterase 1C OS=Homo sapiens OX=9606 GN=PDE1C PE=1 SV=1	0,46247107	2,943635
Q9H0W8	Protein SMG9 OS=Homo sapiens OX=9606 GN=SMG9 PE=1 SV=1	-0,900424504	2,940804
O43290	U4/U6.U5 tri-snRNP-associated protein 1 OS=Homo sapiens OX=9606 GN=SART1 PE=1 SV=1	0,378639329	2,935768
O14787	Transportin-2 OS=Homo sapiens OX=9606 GN=TNPO2 PE=1 SV=3	-0,378109973	2,930745
P08240	Signal recognition particle receptor subunit alpha OS=Homo sapiens OX=9606 GN=SRPRA PE=1 SV=2	0,209549904	2,930695
P63261	Actin, cytoplasmic 2 OS=Homo sapiens OX=9606 GN=ACTG1 PE=1 SV=1	0,529099506	2,925649
Q9HC07	Transmembrane protein 165 OS=Homo sapiens OX=9606 GN=TMEM165 PE=1 SV=1	0,529011743	2,925127
Q9UBB6	Neurochondrin OS=Homo sapiens OX=9606 GN=NCDN PE=1 SV=1	-0,309520406	2,922606
Q10713	Mitochondrial-processing peptidase subunit alpha OS=Homo sapiens OX=9606 GN=PMPCA PE=1 SV=2	-0,411918226	2,919511
Q9UHB9	Signal recognition particle subunit SRP68 OS=Homo sapiens OX=9606 GN=SRP68 PE=1 SV=2	0,232982989	2,915164
P26006	Integrin alpha-3 OS=Homo sapiens OX=9606 GN=ITGA3 PE=1 SV=5	-0,293315784	2,914122
O00443	Phosphatidylinositol 4-phosphate 3-kinase C2 domain-containing subunit alpha OS=Homo sapiens OX=9606 GN=PIK3C2A PE=1 SV=2	0,311591543	2,912324
O43765	Small glutamine-rich tetratricopeptide repeat-containing protein alpha OS=Homo sapiens OX=9606 GN=SGTA PE=1 SV=1	0,348567488	2,908299
P17342	Atrial natriuretic peptide receptor 3 OS=Homo sapiens OX=9606 GN=NPR3 PE=1 SV=2	-0,325821265	2,899549
O60888	Protein CutA OS=Homo sapiens OX=9606 GN=CUTA PE=1 SV=2	-0,883762857	2,89713
O00429	Dynamin-1-like protein OS=Homo sapiens OX=9606 GN=DNM1L PE=1 SV=2	0,162238588	2,89677
P51858	Hepatoma-derived growth factor OS=Homo sapiens OX=9606 GN=HDGF PE=1 SV=1	0,256290178	2,889417
Q02543	60S ribosomal protein L18a OS=Homo sapiens OX=9606 GN=RPL18A PE=1 SV=2	0,372661481	2,879103
Q8IWC1	MAP7 domain-containing protein 3 OS=Homo sapiens OX=9606 GN=MAP7D3 PE=1 SV=2	0,111824669	2,877358
Q9H1P3	Oxysterol-binding protein-related protein 2 OS=Homo sapiens OX=9606 GN=OSBPL2 PE=1 SV=1	0,666210861	2,872357
Q9ULZ3	Apoptosis-associated speck-like protein containing a CARD OS=Homo sapiens OX=9606 GN=PYCARD PE=1 SV=2	-0,452307667	2,869719
P55287	Cadherin-11 OS=Homo sapiens OX=9606 GN=CDH11 PE=1 SV=2	0,37165154	2,869541
Q8IYB3	Serine/arginine repetitive matrix protein 1 OS=Homo sapiens OX=9606 GN=SRRM1 PE=1 SV=2	0,630748655	2,866102
Q969P0	Immunoglobulin superfamily member 8 OS=Homo sapiens OX=9606 GN=IGSF8 PE=1 SV=1	0,618167064	2,865338
P25685	DnaJ homolog subfamily B member 1 OS=Homo sapiens OX=9606 GN=DNAJB1 PE=1 SV=4	0,370882256	2,862261
P52758	2-iminobutanoate/2-iminopropanoate deaminase OS=Homo sapiens OX=9606 GN=RIDA PE=1 SV=1	-0,628507401	2,856107
Q12906	Interleukin enhancer-binding factor 3 OS=Homo sapiens OX=9606 GN=ILF3 PE=1 SV=3	0,183980635	2,850953
Q9UKS6	Protein kinase C and casein kinase substrate in neurons protein 3 OS=Homo sapiens OX=9606 GN=PACSIN3 PE=1 SV=2	0,865498618	2,848653
Q5VYK3	Proteasome adapter and scaffold protein ECM29 OS=Homo sapiens OX=9606 GN=ECPAS PE=1 SV=2	-0,179923679	2,839382
P46108	Adapter molecule crk OS=Homo sapiens OX=9606 GN=CRK PE=1 SV=2	0,287478785	2,838862
Q16851	UTP--glucose-1-phosphate uridylyltransferase OS=Homo sapiens OX=9606 GN=UGP2 PE=1 SV=5	0,199813805	2,837735
Q9H173	Nucleotide exchange factor SIL1 OS=Homo sapiens OX=9606 GN=SIL1 PE=1 SV=1	-0,513730413	2,833938
Q9Y6M1	Insulin-like growth factor 2 mRNA-binding protein 2 OS=Homo sapiens OX=9606 GN=IGF2BP2 PE=1 SV=2	-0,287001181	2,832724
Q8TF42	Ubiquitin-associated and SH3 domain-containing protein B OS=Homo sapiens OX=9606 GN=UBASH3B PE=1 SV=2	-0,401434871	2,831151
Q969V3	Nicalin OS=Homo sapiens OX=9606 GN=NCLN PE=1 SV=2	-0,243255801	2,828772
P46934	E3 ubiquitin-protein ligase NEDD4 OS=Homo sapiens OX=9606 GN=NEDD4 PE=1 SV=4	-0,511726609	2,821948
O95202	Mitochondrial proton/calcium exchanger protein OS=Homo sapiens OX=9606 GN=LETM1 PE=1 SV=1	0,285423208	2,812471
O75396	Vesicle-trafficking protein SEC22b OS=Homo sapiens OX=9606 GN=SEC22B PE=1 SV=4	0,30037946	2,811897
O75607	Nucleoplasmin-3 OS=Homo sapiens OX=9606 GN=NPM3 PE=1 SV=3	0,616538019	2,802522
P35606	Coatomer subunit beta' OS=Homo sapiens OX=9606 GN=COPB2 PE=1 SV=2	0,14775235	2,802439
C9JLW8	Mapk-regulated corepressor-interacting protein 1 OS=Homo sapiens OX=9606 GN=MCRIP1 PE=1 SV=1	-0,507748518	2,798123
Q9UK76	Jupiter microtubule associated homolog 1 OS=Homo sapiens OX=9606 GN=JPT1 PE=1 SV=3	0,397246861	2,795896
Q5SRE5	Nucleoporin NUP188 homolog OS=Homo sapiens OX=9606 GN=NUP188 PE=1 SV=1	-0,845229809	2,794104
Q8IWZ3	Ankyrin repeat and KH domain-containing protein 1 OS=Homo sapiens OX=9606 GN=ANKHD1 PE=1 SV=1	0,506461784	2,790411
O60499	Syntaxin-10 OS=Homo sapiens OX=9606 GN=STX10 PE=1 SV=1	0,085287991	2,785756
P07686	Beta-hexosaminidase subunit beta OS=Homo sapiens OX=9606 GN=HEXB PE=1 SV=3	-0,283056903	2,782165
P84157	Matrix-remodeling-associated protein 7 OS=Homo sapiens OX=9606 GN=MXRA7 PE=1 SV=1	0,439624145	2,777403
P52594	Arf-GAP domain and FG repeat-containing protein 1 OS=Homo sapiens OX=9606 GN=AGFG1 PE=1 SV=2	0,50362136	2,773377
Q13509	Tubulin beta-3 chain OS=Homo sapiens OX=9606 GN=TUBB3 PE=1 SV=2	0,231608824	2,771344
Q9H299	SH3 domain-binding glutamic acid-rich-like protein 3 OS=Homo sapiens OX=9606 GN=SH3BGRL3 PE=1 SV=1	0,360892579	2,767918
O75962	Triple functional domain protein OS=Homo sapiens OX=9606 GN=TRIO PE=1 SV=2	-0,239185906	2,765691
Q9NRY4	Rho GTPase-activating protein 35 OS=Homo sapiens OX=9606 GN=ARHGAP35 PE=1 SV=3	-0,239082405	2,764092
P61513	60S ribosomal protein L37a OS=Homo sapiens OX=9606 GN=RPL37A PE=1 SV=2	0,437601723	2,762679
Q8TD43	Transient receptor potential cation channel subfamily M member 4 OS=Homo sapiens OX=9606 GN=TRPM4 PE=1 SV=1	-0,6070486	2,759788
O94921	Cyclin-dependent kinase 14 OS=Homo sapiens OX=9606 GN=CDK14 PE=1 SV=3	0,287296903	2,758018
Q6P1N9	Putative deoxyribonuclease TATDN1 OS=Homo sapiens OX=9606 GN=TATDN1 PE=1 SV=2	-0,831706229	2,757263
P10109	Adrenodoxin, mitochondrial OS=Homo sapiens OX=9606 GN=FDX1 PE=1 SV=1	-0,517545063	2,754487
P20839	Inosine-5'-monophosphate dehydrogenase 1 OS=Homo sapiens OX=9606 GN=IMPDH1 PE=1 SV=2	0,333724095	2,754051
P48960	CD97 antigen OS=Homo sapiens OX=9606 GN=CD97 PE=1 SV=4	0,392156178	2,753082
Q8N0X7	Spartin OS=Homo sapiens OX=9606 GN=SPART PE=1 SV=1	0,435595018	2,748068
P50281	Matrix metalloproteinase-14 OS=Homo sapiens OX=9606 GN=MMP14 PE=1 SV=3	-0,35857385	2,746075

P19174	1-phosphatidylinositol 4,5-bisphosphate phosphodiesterase gamma-1 OS=Homo sapiens OX=9606 GN=PLCG1 PE=1 SV=1	0,603887088	2,745502
P43003	Excitatory amino acid transporter 1 OS=Homo sapiens OX=9606 GN=SLC1A3 PE=1 SV=1	0,237124622	2,744122
Q7Z417	Nuclear fragile X mental retardation-interacting protein 2 OS=Homo sapiens OX=9606 GN=NUFIP2 PE=1 SV=1	0,390878419	2,742343
P13995	Bifunctional methylenetetrahydrofolate dehydrogenase/cyclohydrolase, mitochondrial OS=Homo sapiens OX=9606 GN=MTHFD2 PE=1 S	-0,390723378	2,74104
Q96N66	Lysophospholipid acyltransferase 7 OS=Homo sapiens OX=9606 GN=MBOAT7 PE=1 SV=2	-0,434427078	2,739564
Q9Y680	Peptidyl-prolyl cis-trans isomerase FKBP7 OS=Homo sapiens OX=9606 GN=FKBP7 PE=1 SV=2	-0,331776525	2,733916
O43143	Pre-mRNA-splicing factor ATP-dependent RNA helicase DHX15 OS=Homo sapiens OX=9606 GN=DHX15 PE=1 SV=2	0,204217912	2,733729
O75390	Citrate synthase, mitochondrial OS=Homo sapiens OX=9606 GN=CS PE=1 SV=2	0,22896357	2,728967
Q53EL6	Programmed cell death protein 4 OS=Homo sapiens OX=9606 GN=PDCD4 PE=1 SV=2	-1,02943166	2,726158
Q9UK41	Vacuolar protein sorting-associated protein 28 homolog OS=Homo sapiens OX=9606 GN=VPS28 PE=1 SV=1	-0,432191767	2,723288
P49321	Nuclear autoantigenic sperm protein OS=Homo sapiens OX=9606 GN=NASP PE=1 SV=2	0,278338601	2,72198
Q9BTY2	Plasma alpha-L-fucosidase OS=Homo sapiens OX=9606 GN=FUCA2 PE=1 SV=2	-0,81548608	2,720943
O94829	Importin-13 OS=Homo sapiens OX=9606 GN=IPO13 PE=1 SV=3	-0,845049424	2,717845
Q8IXL7	Methionine-R-sulfoxide reductase B3 OS=Homo sapiens OX=9606 GN=MSRB3 PE=1 SV=2	-0,494040084	2,715817
P09104	Gamma-enolase OS=Homo sapiens OX=9606 GN=ENO2 PE=1 SV=3	0,265249644	2,715011
Q9Y5K6	CD2-associated protein OS=Homo sapiens OX=9606 GN=CD2AP PE=1 SV=1	0,329834353	2,713862
P30566	Adenylosuccinate lyase OS=Homo sapiens OX=9606 GN=ADSL PE=1 SV=2	0,235658517	2,711367
Q15363	Transmembrane emp24 domain-containing protein 2 OS=Homo sapiens OX=9606 GN=TMED2 PE=1 SV=1	0,354358185	2,706421
Q9Y316	Protein MEMO1 OS=Homo sapiens OX=9606 GN=MEMO1 PE=1 SV=1	0,329095601	2,70624
O43237	Cytoplasmic dynein 1 light intermediate chain 2 OS=Homo sapiens OX=9606 GN=DYNC1LI2 PE=1 SV=1	0,276970523	2,70459
Q9UM47	Neurogenic locus notch homolog protein 3 OS=Homo sapiens OX=9606 GN=NOTCH3 PE=1 SV=2	-0,353944824	2,702536
O95757	Heat shock 70 kDa protein 4L OS=Homo sapiens OX=9606 GN=HSPA4L PE=1 SV=3	0,594205927	2,701607
Q9Y296	Trafficking protein particle complex subunit 4 OS=Homo sapiens OX=9606 GN=TRAPPC4 PE=1 SV=1	-0,594035899	2,700834
O43447	Peptidyl-prolyl cis-trans isomerase H OS=Homo sapiens OX=9606 GN=PPIH PE=1 SV=1	-0,810166678	2,697837
Q9NUQ7	Ufm1-specific protease 2 OS=Homo sapiens OX=9606 GN=UFSP2 PE=1 SV=3	0,384226197	2,686485
P13611	Versican core protein OS=Homo sapiens OX=9606 GN=VCAN PE=1 SV=3	0,233920353	2,684719
P08648	Integrin alpha-5 OS=Homo sapiens OX=9606 GN=ITGA5 PE=1 SV=2	0,226050279	2,682532
P07814	Bifunctional glutamate/proline--tRNA ligase OS=Homo sapiens OX=9606 GN=EPRS PE=1 SV=5	-0,108890573	2,681323
O00471	Exocyst complex component 5 OS=Homo sapiens OX=9606 GN=EXOC5 PE=1 SV=1	-0,383337947	2,679033
Q01085	Nucleolysin TIAR OS=Homo sapiens OX=9606 GN=TIAL1 PE=1 SV=1	-0,306119901	2,678317
Q9BRT3	Migration and invasion enhancer 1 OS=Homo sapiens OX=9606 GN=MIEN1 PE=1 SV=1	-0,802617429	2,67679
P04632	Calpain small subunit 1 OS=Homo sapiens OX=9606 GN=CAPNS1 PE=1 SV=1	0,262336263	2,675984
P38936	Cyclin-dependent kinase inhibitor 1 OS=Homo sapiens OX=9606 GN=CDKN1A PE=1 SV=3	0,623088824	2,674201
Q3KQU3	MAP7 domain-containing protein 1 OS=Homo sapiens OX=9606 GN=MAP7D1 PE=1 SV=1	0,288500441	2,669468
Q8WWI1	LIM domain only protein 7 OS=Homo sapiens OX=9606 GN=LMO7 PE=1 SV=3	-0,106890722	2,667356
P08253	72 kDa type IV collagenase OS=Homo sapiens OX=9606 GN=MMP2 PE=1 SV=2	0,381750202	2,665717
Q14669	E3 ubiquitin-protein ligase TRIP12 OS=Homo sapiens OX=9606 GN=TRIP12 PE=1 SV=1	0,349697223	2,662666
P27708	CAD protein OS=Homo sapiens OX=9606 GN=CAD PE=1 SV=3	0,171649479	2,660988
Q9NW15	Anoctamin-10 OS=Homo sapiens OX=9606 GN=ANO10 PE=1 SV=2	0,380927723	2,658821
O75937	DnaJ homolog subfamily C member 8 OS=Homo sapiens OX=9606 GN=DNAJC8 PE=1 SV=2	0,484189589	2,656489
P55809	Succinyl-CoA:3-ketoacid coenzyme A transferase 1, mitochondrial OS=Homo sapiens OX=9606 GN=OXCT1 PE=1 SV=1	-0,304114526	2,655998
Q92688	Acidic leucine-rich nuclear phosphoprotein 32 family member B OS=Homo sapiens OX=9606 GN=ANP32B PE=1 SV=1	-0,583433803	2,652502
Q8NBS9	Thioredoxin domain-containing protein 5 OS=Homo sapiens OX=9606 GN=TXNDC5 PE=1 SV=2	-0,217123172	2,650952
P19823	Inter-alpha-trypsin inhibitor heavy chain H2 OS=Homo sapiens OX=9606 GN=ITIH2 PE=1 SV=2	0,34804334	2,647164
Q14766	Latent-transforming growth factor beta-binding protein 1 OS=Homo sapiens OX=9606 GN=LTBP1 PE=1 SV=4	-0,420600988	2,647073
Q9HBL8	NmrA-like family domain-containing protein 1 OS=Homo sapiens OX=9606 GN=NMRAL1 PE=1 SV=1	-0,631806903	2,646467
Q658P3	Metalloreductase STEAP3 OS=Homo sapiens OX=9606 GN=STEAP3 PE=1 SV=2	-1,725912727	2,646102
P20700	Lamin-B1 OS=Homo sapiens OX=9606 GN=LMNB1 PE=1 SV=2	0,185443421	2,643797
Q9BTW9	Tubulin-specific chaperone D OS=Homo sapiens OX=9606 GN=TBCD PE=1 SV=2	-0,272100283	2,642912
Q13136	Liprin-alpha-1 OS=Homo sapiens OX=9606 GN=PPFIA1 PE=1 SV=1	0,421125351	2,64271
Q8TCT9	Minor histocompatibility antigen H13 OS=Homo sapiens OX=9606 GN=HM13 PE=1 SV=1	0,302662436	2,639861
Q96CT7	Coiled-coil domain-containing protein 124 OS=Homo sapiens OX=9606 GN=CCDC124 PE=1 SV=1	0,346648942	2,634104
Q6UVK1	Chondroitin sulfate proteoglycan 4 OS=Homo sapiens OX=9606 GN=CSPG4 PE=1 SV=2	-0,12454878	2,631712
Q13643	Four and a half LIM domains protein 3 OS=Homo sapiens OX=9606 GN=FHL3 PE=1 SV=4	-0,377601872	2,630952
P62253	Ubiquitin-conjugating enzyme E2 G1 OS=Homo sapiens OX=9606 GN=UBE2G1 PE=1 SV=3	-0,479896103	2,630585
Q9P291	Armadillo repeat-containing X-linked protein 1 OS=Homo sapiens OX=9606 GN=ARMCX1 PE=1 SV=1	-0,345902453	2,627116
Q6ZWT7	Lysophospholipid acyltransferase 2 OS=Homo sapiens OX=9606 GN=MBOAT2 PE=2 SV=2	0,783246623	2,622256
Q9NUJ3	T-complex protein 11-like protein 1 OS=Homo sapiens OX=9606 GN=TCP11L1 PE=1 SV=1	-0,783174177	2,622051
Q9HD42	Charged multivesicular body protein 1a OS=Homo sapiens OX=9606 GN=CHMP1A PE=1 SV=1	-0,47835814	2,621299
Q9Y4X5	E3 ubiquitin-protein ligase ARIH1 OS=Homo sapiens OX=9606 GN=ARIH1 PE=1 SV=2	-0,345161726	2,620184
O00487	26S proteasome non-ATPase regulatory subunit 14 OS=Homo sapiens OX=9606 GN=PSMD14 PE=1 SV=1	-0,344981335	2,618496
P25205	DNA replication licensing factor MCM3 OS=Homo sapiens OX=9606 GN=MCM3 PE=1 SV=3	-0,221904632	2,616888
O96000	NADH dehydrogenase [ubiquinone] 1 beta subcomplex subunit 10 OS=Homo sapiens OX=9606 GN=NDUFB10 PE=1 SV=3	-0,415840109	2,604234
O95292	Vesicle-associated membrane protein-associated protein B/C OS=Homo sapiens OX=9606 GN=VAPB PE=1 SV=3	0,343442232	2,604103
O95980	Reversion-inducing cysteine-rich protein with Kazal motifs OS=Homo sapiens OX=9606 GN=RECK PE=1 SV=1	0,207925013	2,602442
P32455	Guanylate-binding protein 1 OS=Homo sapiens OX=9606 GN=GBP1 PE=1 SV=2	-0,31827244	2,595017
Q12929	Epidermal growth factor receptor kinase substrate 8 OS=Homo sapiens OX=9606 GN=EPS8 PE=1 SV=1	0,256041627	2,592183
P11310	Medium-chain specific acyl-CoA dehydrogenase, mitochondrial OS=Homo sapiens OX=9606 GN=ACADM PE=1 SV=1	-0,473056182	2,589265
Q9Y2H0	Disks large-associated protein 4 OS=Homo sapiens OX=9606 GN=DLGAP4 PE=1 SV=3	0,569323829	2,587771
Q9NRY5	Protein FAM114A2 OS=Homo sapiens OX=9606 GN=FAM114A2 PE=1 SV=4	0,372221656	2,585924
O15131	Importin subunit alpha-6 OS=Homo sapiens OX=9606 GN=KPNA5 PE=1 SV=2	0,28116273	2,582351
Q9NUQ9	Protein FAM49B OS=Homo sapiens OX=9606 GN=FAM49B PE=1 SV=1	-0,317011948	2,582118
P07311	Acylphosphatase-1 OS=Homo sapiens OX=9606 GN=ACYP1 PE=1 SV=2	0,567946128	2,581426
P29317	Ephrin type-A receptor 2 OS=Homo sapiens OX=9606 GN=EPHA2 PE=1 SV=2	0,22644503	2,571035
P04004	Vitronectin OS=Homo sapiens OX=9606 GN=VTN PE=1 SV=1	1,63004117	2,567261
Q15751	Probable E3 ubiquitin-protein ligase HERC1 OS=Homo sapiens OX=9606 GN=HERC1 PE=1 SV=2	-0,325352402	2,564748
P62820	Ras-related protein Rab-1A OS=Homo sapiens OX=9606 GN=RAB1A PE=1 SV=3	0,243099724	2,55877
P62995	Transformer-2 protein homolog beta OS=Homo sapiens OX=9606 GN=TRA2B PE=1 SV=1	-0,467722231	2,557
Q02952	A-kinase anchor protein 12 OS=Homo sapiens OX=9606 GN=AKAP12 PE=1 SV=4	-0,131494634	2,552506
Q9Y2H6	Fibronectin type-III domain-containing protein 3A OS=Homo sapiens OX=9606 GN=FNDC3A PE=1 SV=4	0,337715374	2,550649
Q53EP0	Fibronectin type III domain-containing protein 3B OS=Homo sapiens OX=9606 GN=FNDC3B PE=1 SV=2	-0,278367803	2,549348
O15269	Serine palmitoyltransferase 1 OS=Homo sapiens OX=9606 GN=SPTLC1 PE=1 SV=1	-0,313360135	2,544817
O43175	D-3-phosphoglycerate dehydrogenase OS=Homo sapiens OX=9606 GN=PHGDH PE=1 SV=4	0,224524949	2,54208
O94760	N(G),N(G)-dimethylarginine dimethylaminohydrolase 1 OS=Homo sapiens OX=9606 GN=DDAH1 PE=1 SV=3	0,36675157	2,540218
P18065	Insulin-like growth factor-binding protein 2 OS=Homo sapiens OX=9606 GN=IGFBP2 PE=1 SV=2	-0,754298308	2,539324
P20338	Ras-related protein Rab-4A OS=Homo sapiens OX=9606 GN=RAB4A PE=1 SV=3	-0,751885292	2,532333
Q86V48	Leucine zipper protein 1 OS=Homo sapiens OX=9606 GN=LUZP1 PE=1 SV=2	0,209773893	2,531284
Q99623	Prohibitin-2 OS=Homo sapiens OX=9606 GN=PHB2 PE=1 SV=2	0,231660827	2,526744
Q14517	Protocadherin Fat 1 OS=Homo sapiens OX=9606 GN=FAT1 PE=1 SV=2	0,405134085	2,526328
P25325	3-mercaptopyruvate sulfurtransferase OS=Homo sapiens OX=9606 GN=MPST PE=1 SV=3	0,74870181	2,523091
P02786	Transferrin receptor protein 1 OS=Homo sapiens OX=9606 GN=TFRC PE=1 SV=2	0,183186666	2,520855
Q16537	Serine/threonine-protein phosphatase 2A 56 kDa regulatory subunit epsilon isoform OS=Homo sapiens OX=9606 GN=PPP2R5E PE=1 SV=	0,333925638	2,515367
Q9HCE0	Ectopic P granules protein 5 homolog OS=Homo sapiens OX=9606 GN=EPG5 PE=1 SV=2	0,250159706	2,514537
Q9Y2V2	Calcium-regulated heat-stable protein 1 OS=Homo sapiens OX=9606 GN=CARHSP1 PE=1 SV=2	-0,553330294	2,51385
Q15758	Neutral amino acid transporter B(0) OS=Homo sapiens OX=9606 GN=SLC1A5 PE=1 SV=2	0,27509313	2,510811
Q9UPT5	Exocyst complex component 7 OS=Homo sapiens OX=9606 GN=EXOC7 PE=1 SV=3	-0,309959281	2,51017
Q9UP83	Conserved oligomeric Golgi complex subunit 5 OS=Homo sapiens OX=9606 GN=COG5 PE=1 SV=3	0,459378991	2,506463
Q96RQ3	Methylcrotonoyl-CoA carboxylase subunit alpha, mitochondrial OS=Homo sapiens OX=9606 GN=MCCC1 PE=1 SV=3	-0,740509408	2,499209

P28838	Cytosol aminopeptidase OS=Homo sapiens OX=9606 GN=LAP3 PE=1 SV=3	-0,207664599	2,49727
P30740	Leukocyte elastase inhibitor OS=Homo sapiens OX=9606 GN=SERPINB1 PE=1 SV=1	0,260404243	2,496288
Q9NX08	COMM domain-containing protein 8 OS=Homo sapiens OX=9606 GN=COMMD8 PE=1 SV=1	-0,457161276	2,493017
P62701	40S ribosomal protein S4, X isoform OS=Homo sapiens OX=9606 GN=RPS4X PE=1 SV=2	0,229315477	2,492967
P27695	DNA-(apurinic or apyrimidinic site) lyase OS=Homo sapiens OX=9606 GN=APEX1 PE=1 SV=2	0,26009281	2,492413
P07108	Acyl-CoA-binding protein OS=Homo sapiens OX=9606 GN=DBI PE=1 SV=2	-0,456967891	2,491844
Q9UKV3	Apoptotic chromatin condensation inducer in the nucleus OS=Homo sapiens OX=9606 GN=ACIN1 PE=1 SV=2	0,289230559	2,491597
P61011	Signal recognition particle 54 kDa protein OS=Homo sapiens OX=9606 GN=SRP54 PE=1 SV=1	0,238209866	2,491249
P67936	Tropomyosin alpha-4 chain OS=Homo sapiens OX=9606 GN=TPM4 PE=1 SV=3	-0,18155625	2,490498
Q9BYI3	Hyccin OS=Homo sapiens OX=9606 GN=FAM126A PE=1 SV=2	-0,735872019	2,485629
P52272	Heterogeneous nuclear ribonucleoprotein M OS=Homo sapiens OX=9606 GN=HNRNPM PE=1 SV=3	-0,127492983	2,485242
Q9Y2X3	Nucleolar protein 58 OS=Homo sapiens OX=9606 GN=NOP58 PE=1 SV=1	0,247758742	2,48303
Q03468	DNA excision repair protein ERCC-6 OS=Homo sapiens OX=9606 GN=ERCC6 PE=1 SV=1	-0,734390414	2,48128
Q16630	Cleavage and polyadenylation specificity factor subunit 6 OS=Homo sapiens OX=9606 GN=CPSF6 PE=1 SV=2	0,359112879	2,476529
P61758	Prefoldin subunit 3 OS=Homo sapiens OX=9606 GN=VBP1 PE=1 SV=4	0,287316051	2,470617
Q52LJ0	Protein FAM98B OS=Homo sapiens OX=9606 GN=FAM98B PE=1 SV=2	0,397149982	2,468273
Q86TU7	Actin-histidine N-methyltransferase OS=Homo sapiens OX=9606 GN=SETD3 PE=1 SV=1	-0,396862789	2,466186
Q9ULK4	Mediator of RNA polymerase II transcription subunit 23 OS=Homo sapiens OX=9606 GN=MED23 PE=1 SV=2	-0,366151638	2,461519
Q15417	Calponin-3 OS=Homo sapiens OX=9606 GN=CNN3 PE=1 SV=1	0,193917289	2,45972
Q8N6M3	Fat storage-inducing transmembrane protein 2 OS=Homo sapiens OX=9606 GN=FITM2 PE=1 SV=1	-1,058785647	2,458948
Q14642	Inositol polyphosphate-5-phosphatase A OS=Homo sapiens OX=9606 GN=INPP5A PE=1 SV=1	0,795879349	2,454944
Q8IYI6	Exocyst complex component 8 OS=Homo sapiens OX=9606 GN=EXOC8 PE=1 SV=2	-0,725415537	2,45484
Q9UBR2	Cathepsin Z OS=Homo sapiens OX=9606 GN=CTSZ PE=1 SV=1	-0,327192606	2,452869
P51451	Tyrosine-protein kinase Blk OS=Homo sapiens OX=9606 GN=BLK PE=1 SV=3	2,543201386	2,446316
P26196	Probable ATP-dependent RNA helicase DDX6 OS=Homo sapiens OX=9606 GN=DDX6 PE=1 SV=2	0,210876691	2,444795
Q96GQ5	RUS1 family protein C16orf58 OS=Homo sapiens OX=9606 GN=C16orf58 PE=1 SV=2	-0,538489266	2,444753
P0C0L4	Complement C4-A OS=Homo sapiens OX=9606 GN=C4A PE=1 SV=2	-0,721452364	2,44311
Q9C0B1	Alpha-ketoglutarate-dependent dioxygenase FTO OS=Homo sapiens OX=9606 GN=FTO PE=1 SV=3	0,32590617	2,440956
Q92743	Serine protease HTRA1 OS=Homo sapiens OX=9606 GN=HTRA1 PE=1 SV=1	-0,325889245	2,4408
Q9Y2D4	Exocyst complex component 6B OS=Homo sapiens OX=9606 GN=EXOC6B PE=1 SV=3	0,393250766	2,439939
P46779	60S ribosomal protein L28 OS=Homo sapiens OX=9606 GN=RPL28 PE=1 SV=3	0,325607456	2,438192
Q9BY32	Inosine triphosphate pyrophosphatase OS=Homo sapiens OX=9606 GN=ITPA PE=1 SV=2	0,392680022	2,435793
P51946	Cyclin-H OS=Homo sapiens OX=9606 GN=CCNH PE=1 SV=1	-0,094595739	2,430716
Q9H1R3	Myosin light chain kinase 2, skeletal/cardiac muscle OS=Homo sapiens OX=9606 GN=MYLK2 PE=1 SV=3	1,055126647	2,42016
Q13671	Ras and Rab interactor 1 OS=Homo sapiens OX=9606 GN=RIN1 PE=1 SV=4	-0,533072216	2,419415
Q96QK1	Vacuolar protein sorting-associated protein 35 OS=Homo sapiens OX=9606 GN=VPS35 PE=1 SV=2	-0,160109692	2,4194
Q86TB9	Protein PAT1 homolog 1 OS=Homo sapiens OX=9606 GN=PATL1 PE=1 SV=2	0,897915491	2,41752
Q9P270	SLAIN motif-containing protein 2 OS=Homo sapiens OX=9606 GN=SLAIN2 PE=1 SV=2	0,351537042	2,413537
P35659	Protein DEK OS=Homo sapiens OX=9606 GN=DEK PE=1 SV=1	-0,3228691	2,412869
P30041	Peroxiredoxin-6 OS=Homo sapiens OX=9606 GN=PRDX6 PE=1 SV=3	-0,19090341	2,408519
Q9Y4E8	Ubiquitin carboxyl-terminal hydrolase 15 OS=Homo sapiens OX=9606 GN=USP15 PE=1 SV=3	0,201995997	2,406609
O43708	Maleylacetoacetate isomerase OS=Homo sapiens OX=9606 GN=GSTZ1 PE=1 SV=3	-0,530001581	2,405025
P53779	Mitogen-activated protein kinase 10 OS=Homo sapiens OX=9606 GN=MAPK10 PE=1 SV=2	-0,505583288	2,40309
P30038	Delta-1-pyrroline-5-carboxylate dehydrogenase, mitochondrial OS=Homo sapiens OX=9606 GN=ALDH4A1 PE=1 SV=3	0,349753194	2,398732
Q8NHP8	Putative phospholipase B-like 2 OS=Homo sapiens OX=9606 GN=PLBD2 PE=1 SV=2	-0,44155724	2,398271
Q9BV20	Methylthioribose-1-phosphate isomerase OS=Homo sapiens OX=9606 GN=MRI1 PE=1 SV=1	0,527824314	2,39481
Q16181	Septin-7 OS=Homo sapiens OX=9606 GN=SEPTIN7 PE=1 SV=2	0,200952886	2,390046
Q92520	Protein FAM3C OS=Homo sapiens OX=9606 GN=FAM3C PE=1 SV=1	0,279808807	2,388733
Q8NCA5	Protein FAM98A OS=Homo sapiens OX=9606 GN=FAM98A PE=1 SV=2	0,385718063	2,385244
Q96PD2	Discoidin, CUB and LCCL domain-containing protein 2 OS=Homo sapiens OX=9606 GN=DCBLD2 PE=1 SV=1	0,525019028	2,381634
Q3MHD2	Protein LSM12 homolog OS=Homo sapiens OX=9606 GN=LSM12 PE=1 SV=2	-0,523371925	2,373891
Q9NXG2	THUMP domain-containing protein 1 OS=Homo sapiens OX=9606 GN=THUMPD1 PE=1 SV=2	0,346597983	2,37257
P40222	Alpha-taxilin OS=Homo sapiens OX=9606 GN=TXLNA PE=1 SV=3	-0,220775845	2,371146
Q04323	UBX domain-containing protein 1 OS=Homo sapiens OX=9606 GN=UBXN1 PE=1 SV=2	-0,262958289	2,369265
O60282	Kinesin heavy chain isoform 5C OS=Homo sapiens OX=9606 GN=KIF5C PE=1 SV=1	2,009817435	2,368789
Q6YP21	Kynurenine--oxoglutarate transaminase 3 OS=Homo sapiens OX=9606 GN=KYAT3 PE=1 SV=1	-0,383383333	2,368304
Q8NC51	Plasminogen activator inhibitor 1 RNA-binding protein OS=Homo sapiens OX=9606 GN=SERBP1 PE=1 SV=2	0,220352546	2,365155
P29536	Leiomodin-1 OS=Homo sapiens OX=9606 GN=LMOD1 PE=1 SV=3	0,317474965	2,363115
Q9NRW3	DNA dC->dU-editing enzyme APOBEC-3C OS=Homo sapiens OX=9606 GN=APOBEC3C PE=1 SV=2	-0,520934317	2,362421
P48739	Phosphatidylinositol transfer protein beta isoform OS=Homo sapiens OX=9606 GN=PITPNB PE=1 SV=2	0,238339556	2,3605
P17302	Gap junction alpha-1 protein OS=Homo sapiens OX=9606 GN=GJA1 PE=1 SV=2	-0,344895167	2,358466
O60551	Glycylpeptide N-tetradecanoyltransferase 2 OS=Homo sapiens OX=9606 GN=NMT2 PE=1 SV=1	-0,381981717	2,358137
O94826	Mitochondrial import receptor subunit TOM70 OS=Homo sapiens OX=9606 GN=TOMM70 PE=1 SV=1	0,212122278	2,357518
P21953	2-oxoisovalerate dehydrogenase subunit beta, mitochondrial OS=Homo sapiens OX=9606 GN=BCKDHB PE=1 SV=2	1,450294781	2,35718
Q6PJW8	Consortin OS=Homo sapiens OX=9606 GN=CNST PE=1 SV=3	-0,333191956	2,356899
P61006	Ras-related protein Rab-8A OS=Homo sapiens OX=9606 GN=RAB8A PE=1 SV=1	-0,344258332	2,353193
Q6UUV9	CREB-regulated transcription coactivator 1 OS=Homo sapiens OX=9606 GN=CRTC1 PE=1 SV=2	0,09312567	2,351374
P12268	Inosine-5'-monophosphate dehydrogenase 2 OS=Homo sapiens OX=9606 GN=IMPDH2 PE=1 SV=2	0,173935603	2,350334
P18887	DNA repair protein XRCC1 OS=Homo sapiens OX=9606 GN=XRCC1 PE=1 SV=2	1,387841989	2,347695
O75326	Semaphorin-7A OS=Homo sapiens OX=9606 GN=SEMA7A PE=1 SV=1	0,182351772	2,346019
P62277	40S ribosomal protein S13 OS=Homo sapiens OX=9606 GN=RPS13 PE=1 SV=2	-0,29367628	2,345579
P54105	Methylosome subunit pICln OS=Homo sapiens OX=9606 GN=CLNS1A PE=1 SV=1	-2,130958797	2,345037
Q8NFQ8	Torsin-1A-interacting protein 2 OS=Homo sapiens OX=9606 GN=TOR1AIP2 PE=1 SV=1	0,248028845	2,34356
Q96C23	Aldose 1-epimerase OS=Homo sapiens OX=9606 GN=GALM PE=1 SV=1	-0,97011296	2,340589
Q9UBC2	Epidermal growth factor receptor substrate 15-like 1 OS=Homo sapiens OX=9606 GN=EPS15L1 PE=1 SV=1	0,226813427	2,335943
P11216	Glycogen phosphorylase, brain form OS=Homo sapiens OX=9606 GN=PYGB PE=1 SV=5	-0,131178349	2,334324
Q9H0B6	Kinesin light chain 2 OS=Homo sapiens OX=9606 GN=KLC2 PE=1 SV=1	-0,274750649	2,333917
Q5JWF2	Guanine nucleotide-binding protein G(s) subunit alpha isoforms XLas OS=Homo sapiens OX=9606 GN=GNAS PE=1 SV=2	-0,341888704	2,333588
Q06481	Amyloid-like protein 2 OS=Homo sapiens OX=9606 GN=APLP2 PE=1 SV=2	0,684585922	2,332395
Q15020	Squamous cell carcinoma antigen recognized by T-cells 3 OS=Homo sapiens OX=9606 GN=SART3 PE=1 SV=1	-0,292277977	2,33155
Q8IXB1	DnaJ homolog subfamily C member 10 OS=Homo sapiens OX=9606 GN=DNAJC10 PE=1 SV=2	0,236014042	2,330518
O14972	Vacuolar protein sorting-associated protein 26C OS=Homo sapiens OX=9606 GN=VPS26C PE=1 SV=1	-0,682343262	2,325566
Q6PII5	Hydroxyacylglutathione hydrolase-like protein OS=Homo sapiens OX=9606 GN=HAGHL PE=2 SV=1	1,565704694	2,324421
Q9NWX5	Ankyrin repeat and SOCS box protein 6 OS=Homo sapiens OX=9606 GN=ASB6 PE=1 SV=1	0,680923751	2,321238
O14818	Proteasome subunit alpha type-7 OS=Homo sapiens OX=9606 GN=PSMA7 PE=1 SV=1	-0,273501925	2,32043
Q9UHI6	Probable ATP-dependent RNA helicase DDX20 OS=Homo sapiens OX=9606 GN=DDX20 PE=1 SV=2	0,326795572	2,319059
Q9NZJ7	Mitochondrial carrier homolog 1 OS=Homo sapiens OX=9606 GN=MTCH1 PE=1 SV=1	-0,376265083	2,316699
Q9NVZ3	Adaptin ear-binding coat-associated protein 2 OS=Homo sapiens OX=9606 GN=NECAP2 PE=1 SV=1	0,428133385	2,316605
P42226	Signal transducer and activator of transcription 6 OS=Homo sapiens OX=9606 GN=STAT6 PE=1 SV=1	0,312411431	2,31657
Q9H2W6	39S ribosomal protein L46, mitochondrial OS=Homo sapiens OX=9606 GN=MRPL46 PE=1 SV=1	-1,017688325	2,31514
Q86XP3	ATP-dependent RNA helicase DDX42 OS=Homo sapiens OX=9606 GN=DDX42 PE=1 SV=1	0,290425785	2,312993
Q9Y6E2	Basic leucine zipper and W2 domain-containing protein 2 OS=Homo sapiens OX=9606 GN=BZW2 PE=1 SV=1	0,21607018	2,304815
Q9UBE0	SUMO-activating enzyme subunit 1 OS=Homo sapiens OX=9606 GN=SAE1 PE=1 SV=1	0,257314034	2,30413
Q9NUY8	TBC1 domain family member 23 OS=Homo sapiens OX=9606 GN=TBC1D23 PE=1 SV=3	-0,37398416	2,300178
Q9BTU6	Phosphatidylinositol 4-kinase type 2-alpha OS=Homo sapiens OX=9606 GN=PI4K2A PE=1 SV=1	-0,673992331	2,300043
P11717	Cation-independent mannose-6-phosphate receptor OS=Homo sapiens OX=9606 GN=IGF2R PE=1 SV=3	-0,143703335	2,297333
P49750	YLP motif-containing protein 1 OS=Homo sapiens OX=9606 GN=YLPM1 PE=1 SV=4	0,373463704	2,296409
P06756	Integrin alpha-V OS=Homo sapiens OX=9606 GN=ITGAV PE=1 SV=2	-0,126175451	2,2932

Q6P2Q9	Pre-mRNA-processing-splicing factor 8 OS=Homo sapiens OX=9606 GN=PRPF8 PE=1 SV=2	-0,124548349	2,291552
P61916	NPC intracellular cholesterol transporter 2 OS=Homo sapiens OX=9606 GN=NPC2 PE=1 SV=1	-0,336635009	2,290195
Q99519	Sialidase-1 OS=Homo sapiens OX=9606 GN=NEU1 PE=1 SV=1	-0,555873208	2,290171
P41240	Tyrosine-protein kinase CSK OS=Homo sapiens OX=9606 GN=CSK PE=1 SV=1	0,243503695	2,288363
Q12874	Splicing factor 3A subunit 3 OS=Homo sapiens OX=9606 GN=SF3A3 PE=1 SV=1	0,309281584	2,28788
Q8TCJ2	Dolichyl-diphosphooligosaccharide--protein glycosyltransferase subunit STT3B OS=Homo sapiens OX=9606 GN=STT3B PE=1 SV=1	0,243461716	2,287852
Q14258	E3 ubiquitin/ISG15 ligase TRIM25 OS=Homo sapiens OX=9606 GN=TRIM25 PE=1 SV=2	0,207014644	2,282788
Q9NYZ1	Golgi apparatus membrane protein TVP23 homolog B OS=Homo sapiens OX=9606 GN=TVP23B PE=1 SV=2	1,767864704	2,282104
Q9Y605	MORF4 family-associated protein 1 OS=Homo sapiens OX=9606 GN=MRFAP1 PE=1 SV=1	1,511596145	2,281948
Q9NQA5	Transient receptor potential cation channel subfamily V member 5 OS=Homo sapiens OX=9606 GN=TRPV5 PE=1 SV=2	-1,405623558	2,280499
P35442	Thrombospondin-2 OS=Homo sapiens OX=9606 GN=THBS2 PE=1 SV=2	0,495469306	2,279823
P63267	Actin, gamma-enteric smooth muscle OS=Homo sapiens OX=9606 GN=ACTG2 PE=1 SV=1	0,140616406	2,279737
P19474	E3 ubiquitin-protein ligase TRIM21 OS=Homo sapiens OX=9606 GN=TRIM21 PE=1 SV=1	1,318694133	2,278776
Q6DD88	Atlastin-3 OS=Homo sapiens OX=9606 GN=ATL3 PE=1 SV=1	0,183118467	2,277968
O75347	Tubulin-specific chaperone A OS=Homo sapiens OX=9606 GN=TBCA PE=1 SV=3	-0,254919643	2,276637
Q9NP58	ATP-binding cassette sub-family B member 6, mitochondrial OS=Homo sapiens OX=9606 GN=ABCB6 PE=1 SV=1	-0,960856163	2,275165
P23396	40S ribosomal protein S3 OS=Homo sapiens OX=9606 GN=RPS3 PE=1 SV=2	0,169731648	2,274246
P29692	Elongation factor 1-delta OS=Homo sapiens OX=9606 GN=EEF1D PE=1 SV=5	0,169641673	2,272627
Q07157	Tight junction protein ZO-1 OS=Homo sapiens OX=9606 GN=TJP1 PE=1 SV=3	-0,173678301	2,272151
Q6XQN6	Nicotinate phosphoribosyltransferase OS=Homo sapiens OX=9606 GN=NAPRT PE=1 SV=2	0,702741315	2,270607
Q9Y385	Ubiquitin-conjugating enzyme E2 J1 OS=Homo sapiens OX=9606 GN=UBE2J1 PE=1 SV=2	0,304806587	2,267937
Q8NDV7	Trinucleotide repeat-containing gene 6A protein OS=Homo sapiens OX=9606 GN=TNRC6A PE=1 SV=2	-0,36932903	2,264527
Q7Z3E5	LisH domain-containing protein ARMC9 OS=Homo sapiens OX=9606 GN=ARMC9 PE=1 SV=3	-1,867070813	2,264202
O95433	Activator of 90 kDa heat shock protein ATPase homolog 1 OS=Homo sapiens OX=9606 GN=AHSA1 PE=1 SV=1	0,213092598	2,263142
Q9GZT4	Serine racemase OS=Homo sapiens OX=9606 GN=SRR PE=1 SV=1	-0,419078697	2,26146
O00391	Sulfhydryl oxidase 1 OS=Homo sapiens OX=9606 GN=QSOX1 PE=1 SV=3	-0,661394235	2,261254
Q5VW36	Focadhesin OS=Homo sapiens OX=9606 GN=FOCAD PE=1 SV=1	0,418467966	2,25774
Q6IAN0	Dehydrogenase/reductase SDR family member 7B OS=Homo sapiens OX=9606 GN=DHRS7B PE=1 SV=2	-0,498474426	2,256192
P49591	Serine--tRNA ligase, cytoplasmic OS=Homo sapiens OX=9606 GN=SARS PE=1 SV=3	-0,181701153	2,254468
P04899	Guanine nucleotide-binding protein G(i) subunit alpha-2 OS=Homo sapiens OX=9606 GN=GNAI2 PE=1 SV=3	-0,186738944	2,253723
Q96QR8	Transcriptional activator protein Pur-beta OS=Homo sapiens OX=9606 GN=PURB PE=1 SV=3	0,658936876	2,253648
O95249	Golgi SNAP receptor complex member 1 OS=Homo sapiens OX=9606 GN=GOSR1 PE=1 SV=1	0,658746912	2,25306
P14550	Aldo-keto reductase family 1 member A1 OS=Homo sapiens OX=9606 GN=AKR1A1 PE=1 SV=3	0,229883425	2,252003
Q14195	Dihydropyrimidinase-related protein 3 OS=Homo sapiens OX=9606 GN=DPYSL3 PE=1 SV=1	0,168387299	2,250101
Q9Y4F1	FERM, ARHGEF and pleckstrin domain-containing protein 1 OS=Homo sapiens OX=9606 GN=FARP1 PE=1 SV=1	-0,252594651	2,250023
Q08AM6	Protein VAC14 homolog OS=Homo sapiens OX=9606 GN=VAC14 PE=1 SV=1	-0,366798428	2,248182
Q8IUR0	Trafficking protein particle complex subunit 5 OS=Homo sapiens OX=9606 GN=TRAPPC5 PE=1 SV=1	0,416799791	2,247576
P46379	Large proline-rich protein BAG6 OS=Homo sapiens OX=9606 GN=BAG6 PE=1 SV=2	0,25223811	2,245948
Q9BS26	Endoplasmic reticulum resident protein 44 OS=Homo sapiens OX=9606 GN=ERP44 PE=1 SV=1	0,204304302	2,243441
O95155	Ubiquitin conjugation factor E4 B OS=Homo sapiens OX=9606 GN=UBE4B PE=1 SV=1	0,283356919	2,242456
Q9P121	Neurotrimin OS=Homo sapiens OX=9606 GN=NTM PE=1 SV=1	-0,495395381	2,241555
O75822	Eukaryotic translation initiation factor 3 subunit J OS=Homo sapiens OX=9606 GN=EIF3J PE=1 SV=2	0,283048253	2,239386
P46783	40S ribosomal protein S10 OS=Homo sapiens OX=9606 GN=RPS10 PE=1 SV=1	0,28304683	2,239372
O75947	ATP synthase subunit d, mitochondrial OS=Homo sapiens OX=9606 GN=ATP5PD PE=1 SV=3	-0,283012916	2,239035
P07996	Thrombospondin-1 OS=Homo sapiens OX=9606 GN=THBS1 PE=1 SV=2	0,11294517	2,234671
Q9NR30	Nucleolar RNA helicase 2 OS=Homo sapiens OX=9606 GN=DDX21 PE=1 SV=5	0,210990016	2,233855
Q6P4E1	Protein CASC4 OS=Homo sapiens OX=9606 GN=CASC4 PE=1 SV=2	0,493647652	2,233239
P35080	Profilin-2 OS=Homo sapiens OX=9606 GN=PFN2 PE=1 SV=3	-0,364120811	2,228828
Q9Y617	Phosphoserine aminotransferase OS=Homo sapiens OX=9606 GN=PSAT1 PE=1 SV=2	-0,196669926	2,228695
P62805	Histone H4 OS=Homo sapiens OX=9606 GN=HIST1H4A PE=1 SV=2	0,302691569	2,227678
Q9GZZ9	Ubiquitin-like modifier-activating enzyme 5 OS=Homo sapiens OX=9606 GN=UBA5 PE=1 SV=1	-0,492370477	2,227158
P49815	Tuberin OS=Homo sapiens OX=9606 GN=TSC2 PE=1 SV=2	0,491952088	2,225166
P50238	Cysteine-rich protein 1 OS=Homo sapiens OX=9606 GN=CRIP1 PE=1 SV=3	0,49191766	2,225002
Q9H0H5	Rac GTPase-activating protein 1 OS=Homo sapiens OX=9606 GN=RACGAP1 PE=1 SV=1	-0,491793831	2,224412
P26022	Pentraxin-related protein PTX3 OS=Homo sapiens OX=9606 GN=PTX3 PE=1 SV=3	-0,328286033	2,221458
P49662	Caspase-4 OS=Homo sapiens OX=9606 GN=CASP4 PE=1 SV=1	0,844342247	2,220316
P15289	Arylsulfatase A OS=Homo sapiens OX=9606 GN=ARSA PE=1 SV=3	-0,327876101	2,218091
Q9UEY8	Gamma-adducin OS=Homo sapiens OX=9606 GN=ADD3 PE=1 SV=1	0,195934649	2,217687
P46782	40S ribosomal protein S5 OS=Homo sapiens OX=9606 GN=RPS5 PE=1 SV=4	0,23750802	2,215781
O43865	S-adenosylhomocysteine hydrolase-like protein 1 OS=Homo sapiens OX=9606 GN=AHCYL1 PE=1 SV=2	0,20941226	2,211956
Q9NZU5	LIM and cysteine-rich domains protein 1 OS=Homo sapiens OX=9606 GN=LMCD1 PE=1 SV=1	-0,174370364	2,209648
Q13084	39S ribosomal protein L28, mitochondrial OS=Homo sapiens OX=9606 GN=MRPL28 PE=1 SV=4	1,345289036	2,208454
Q07866	Kinesin light chain 1 OS=Homo sapiens OX=9606 GN=KLC1 PE=1 SV=2	-0,183812972	2,206892
Q96EK5	KIF1-binding protein OS=Homo sapiens OX=9606 GN=KIF1BP PE=1 SV=1	0,300379904	2,206629
Q9BQG0	Myb-binding protein 1A OS=Homo sapiens OX=9606 GN=MYBBP1A PE=1 SV=2	0,201733082	2,206315
P18669	Phosphoglycerate mutase 1 OS=Homo sapiens OX=9606 GN=PGAM1 PE=1 SV=2	0,194932005	2,202705
Q10472	Polypeptide N-acetylgalactosaminyltransferase 1 OS=Homo sapiens OX=9606 GN=GALNT1 PE=1 SV=1	0,194763458	2,20019
Q14739	Delta(14)-sterol reductase OS=Homo sapiens OX=9606 GN=LBR PE=1 SV=2	-0,641181759	2,198306
O60568	Multifunctional procollagen lysine hydroxylase and glycosyltransferase LH3 OS=Homo sapiens OX=9606 GN=PLOD3 PE=1 SV=1	0,154973713	2,195942
Q5T6V5	Queuosine salvage protein OS=Homo sapiens OX=9606 GN=C9orf64 PE=1 SV=1	0,484637703	2,190283
Q14257	Reticulocalbin-2 OS=Homo sapiens OX=9606 GN=RCN2 PE=1 SV=1	-0,235271948	2,188876
P62841	40S ribosomal protein S15 OS=Homo sapiens OX=9606 GN=RPS15 PE=1 SV=2	0,406908641	2,187295
Q8IYB8	ATP-dependent RNA helicase SUPV3L1, mitochondrial OS=Homo sapiens OX=9606 GN=SUPV3L1 PE=1 SV=1	0,520176244	2,185104
Q9Y281	Cofilin-2 OS=Homo sapiens OX=9606 GN=CFL2 PE=1 SV=1	-0,277375629	2,183135
P22392	Nucleoside diphosphate kinase B OS=Homo sapiens OX=9606 GN=NME2 PE=1 SV=1	0,323606756	2,18306
Q96D15	Reticulocalbin-3 OS=Homo sapiens OX=9606 GN=RCN3 PE=1 SV=1	0,224383259	2,182221
Q969Q5	Ras-related protein Rab-24 OS=Homo sapiens OX=9606 GN=RAB24 PE=1 SV=1	-0,489944519	2,181624
P29218	Inositol monophosphatase 1 OS=Homo sapiens OX=9606 GN=IMPA1 PE=1 SV=1	-0,357002664	2,177444
P21291	Cysteine and glycine-rich protein 1 OS=Homo sapiens OX=9606 GN=CSRP1 PE=1 SV=3	-0,214766756	2,175026
A6NHR9	Structural maintenance of chromosomes flexible hinge domain-containing protein 1 OS=Homo sapiens OX=9606 GN=SMCHD1 PE=1 SV=	-0,883648364	2,174607
Q7Z2Z2	Elongation factor-like GTPase 1 OS=Homo sapiens OX=9606 GN=EFL1 PE=1 SV=2	0,259789785	2,173547
P18846	Cyclic AMP-dependent transcription factor ATF-1 OS=Homo sapiens OX=9606 GN=ATF1 PE=1 SV=2	0,254067546	2,173207
O94776	Metastasis-associated protein MTA2 OS=Homo sapiens OX=9606 GN=MTA2 PE=1 SV=1	0,29662644	2,172529
P49368	T-complex protein 1 subunit gamma OS=Homo sapiens OX=9606 GN=CCT3 PE=1 SV=4	0,164032781	2,17252
Q14161	ARF GTPase-activating protein GIT2 OS=Homo sapiens OX=9606 GN=GIT2 PE=1 SV=2	-0,404465832	2,172404
Q9Y4K1	Beta/gamma crystallin domain-containing protein 1 OS=Homo sapiens OX=9606 GN=CRYBG1 PE=1 SV=3	0,348867249	2,170713
P43034	Platelet-activating factor acetylhydrolase IB subunit alpha OS=Homo sapiens OX=9606 GN=PAFAH1B1 PE=1 SV=2	-0,186497913	2,164433
Q7KZI7	Serine/threonine-protein kinase MARK2 OS=Homo sapiens OX=9606 GN=MARK2 PE=1 SV=2	0,585534126	2,163511
P60953	Cell division control protein 42 homolog OS=Homo sapiens OX=9606 GN=CDC42 PE=1 SV=2	-0,258797227	2,163005
Q8WUD4	Coiled-coil domain-containing protein 12 OS=Homo sapiens OX=9606 GN=CCDC12 PE=1 SV=1	0,498938813	2,158206
P24593	Insulin-like growth factor-binding protein 5 OS=Homo sapiens OX=9606 GN=IGFBP5 PE=1 SV=1	-0,991809836	2,156331
P31942	Heterogeneous nuclear ribonucleoprotein H3 OS=Homo sapiens OX=9606 GN=HNRNPH3 PE=1 SV=2	0,353840528	2,154648
P09960	Leukotriene A-4 hydrolase OS=Homo sapiens OX=9606 GN=LTA4H PE=1 SV=2	0,162722016	2,149356
Q8N2K0	Lysophosphatidylserine lipase ABHD12 OS=Homo sapiens OX=9606 GN=ABHD12 PE=1 SV=2	-0,319461547	2,149125
O75608	Acyl-protein thioesterase 1 OS=Homo sapiens OX=9606 GN=LYPLA1 PE=1 SV=1	-0,400522515	2,148366
P61204	ADP-ribosylation factor 3 OS=Homo sapiens OX=9606 GN=ARF3 PE=1 SV=2	0,231403936	2,14255
P12277	Creatine kinase B-type OS=Homo sapiens OX=9606 GN=CKB PE=1 SV=1	-0,203659892	2,132682
Q9H074	Polyadenylate-binding protein-interacting protein 1 OS=Homo sapiens OX=9606 GN=PAIP1 PE=1 SV=1	-0,472302093	2,131247

Q8NBJ9	SID1 transmembrane family member 2 OS=Homo sapiens OX=9606 GN=SIDT2 PE=1 SV=2	-1,180521612	2,131186
Q86Y33	Cell division cycle protein 20 homolog B OS=Homo sapiens OX=9606 GN=CDC20B PE=1 SV=3	1,663321493	2,130427
Q9GZR7	ATP-dependent RNA helicase DDX24 OS=Homo sapiens OX=9606 GN=DDX24 PE=1 SV=1	0,619586208	2,130077
Q96RP9	Elongation factor G, mitochondrial OS=Homo sapiens OX=9606 GN=GFM1 PE=1 SV=2	-0,255537153	2,128465
Q01780	Exosome component 10 OS=Homo sapiens OX=9606 GN=EXOSC10 PE=1 SV=2	-0,618215503	2,125712
O75410	Transforming acidic coiled-coil-containing protein 1 OS=Homo sapiens OX=9606 GN=TACC1 PE=1 SV=2	1,174754344	2,124714
P50479	PDZ and LIM domain protein 4 OS=Homo sapiens OX=9606 GN=PDLIM4 PE=1 SV=2	-0,254960899	2,122373
P62258	14-3-3 protein epsilon OS=Homo sapiens OX=9606 GN=YWHAE PE=1 SV=1	0,178406904	2,121239
Q6PKG0	La-related protein 1 OS=Homo sapiens OX=9606 GN=LARP1 PE=1 SV=2	-0,160957065	2,118303
O95218	Zinc finger Ran-binding domain-containing protein 2 OS=Homo sapiens OX=9606 GN=ZRANB2 PE=1 SV=2	0,348713907	2,117737
Q12959	Disks large homolog 1 OS=Homo sapiens OX=9606 GN=DLG1 PE=1 SV=2	0,469414636	2,117393
O95347	Structural maintenance of chromosomes protein 2 OS=Homo sapiens OX=9606 GN=SMC2 PE=1 SV=2	-0,24074913	2,115693
P12004	Proliferating cell nuclear antigen OS=Homo sapiens OX=9606 GN=PCNA PE=1 SV=1	0,240650287	2,114581
Q8IY81	pre-rRNA 2'-O-ribose RNA methyltransferase FTSJ3 OS=Homo sapiens OX=9606 GN=FTSJ3 PE=1 SV=2	0,614559443	2,114051
Q15043	Zinc transporter ZIP14 OS=Homo sapiens OX=9606 GN=SLC39A14 PE=1 SV=3	0,613558165	2,110853
Q15907	Ras-related protein Rab-11B OS=Homo sapiens OX=9606 GN=RAB11B PE=1 SV=4	-0,170987061	2,110127
Q6ZVM7	TOM1-like protein 2 OS=Homo sapiens OX=9606 GN=TOM1L2 PE=1 SV=1	0,239852559	2,105614
Q9BV57	1,2-dihydroxy-3-keto-5-methylthiopentene dioxygenase OS=Homo sapiens OX=9606 GN=ADI1 PE=1 SV=1	-0,313810191	2,102987
Q8NFA0	Ubiquitin carboxyl-terminal hydrolase 32 OS=Homo sapiens OX=9606 GN=USP32 PE=1 SV=1	-0,1886984	2,102518
Q8TC07	TBC1 domain family member 15 OS=Homo sapiens OX=9606 GN=TBC1D15 PE=1 SV=2	-0,466229963	2,102097
P04792	Heat shock protein beta-1 OS=Homo sapiens OX=9606 GN=HSPB1 PE=1 SV=2	0,172303293	2,100756
P62195	26S proteasome regulatory subunit 8 OS=Homo sapiens OX=9606 GN=PSMC5 PE=1 SV=1	-0,172197269	2,099043
Q9Y5F9	Protocadherin gamma-B6 OS=Homo sapiens OX=9606 GN=PCDHGB6 PE=2 SV=1	1,151537653	2,098387
P09497	Clathrin light chain B OS=Homo sapiens OX=9606 GN=CLTB PE=1 SV=1	-0,345871975	2,0973
Q16543	Hsp90 co-chaperone Cdc37 OS=Homo sapiens OX=9606 GN=CDC37 PE=1 SV=1	0,217381164	2,094308
O95425	Supervillin OS=Homo sapiens OX=9606 GN=SVIL PE=1 SV=2	-0,311216809	2,090754
P09417	Dihydropteridine reductase OS=Homo sapiens OX=9606 GN=QDPR PE=1 SV=2	-0,34447974	2,087296
P99999	Cytochrome c OS=Homo sapiens OX=9606 GN=CYCS PE=1 SV=2	-0,311628478	2,085216
Q9P1W8	#N/D	0,227117118	2,084955
Q13033	Striatin-3 OS=Homo sapiens OX=9606 GN=STRN3 PE=1 SV=3	0,286926412	2,084873
Q9UP52	#N/D	0,257820976	2,084098
Q99704	Docking protein 1 OS=Homo sapiens OX=9606 GN=DOK1 PE=1 SV=1	-0,286693048	2,082773
P53618	Coatomer subunit beta OS=Homo sapiens OX=9606 GN=COPB1 PE=1 SV=3	0,123882451	2,081283
P48634	Protein PRRC2A OS=Homo sapiens OX=9606 GN=PRRC2A PE=1 SV=3	0,60338323	2,078226
Q7Z4V5	Hepatoma-derived growth factor-related protein 2 OS=Homo sapiens OX=9606 GN=HDGFL2 PE=1 SV=1	-1,133296483	2,07739
O60573	Eukaryotic translation initiation factor 4E type 2 OS=Homo sapiens OX=9606 GN=EIF4E2 PE=1 SV=1	-1,130807034	2,074503
Q13191	E3 ubiquitin-protein ligase CBL-B OS=Homo sapiens OX=9606 GN=CBLB PE=1 SV=2	0,34245266	2,072737
Q8N5M9	Protein jagunal homolog 1 OS=Homo sapiens OX=9606 GN=JAGN1 PE=1 SV=1	0,155409967	2,069266
Q63ZY3	KN motif and ankyrin repeat domain-containing protein 2 OS=Homo sapiens OX=9606 GN=KANK2 PE=1 SV=1	0,206634523	2,068361
O14929	Histone acetyltransferase type B catalytic subunit OS=Homo sapiens OX=9606 GN=HAT1 PE=1 SV=1	-0,458522142	2,065014
Q8N5K1	CDGSH iron-sulfur domain-containing protein 2 OS=Homo sapiens OX=9606 GN=CISD2 PE=1 SV=1	1,122057857	2,064315
P98160	Basement membrane-specific heparan sulfate proteoglycan core protein OS=Homo sapiens OX=9606 GN=HSPG2 PE=1 SV=4	0,26509637	2,062452
O94919	Endonuclease domain-containing 1 protein OS=Homo sapiens OX=9606 GN=ENDOD1 PE=1 SV=2	-0,340256993	2,056979
P17931	Galectin-3 OS=Homo sapiens OX=9606 GN=LGALS3 PE=1 SV=5	-0,283770973	2,056509
Q9UM22	Mammalian ependymin-related protein 1 OS=Homo sapiens OX=9606 GN=EPDR1 PE=1 SV=2	-0,59660891	2,05638
P49406	39S ribosomal protein L19, mitochondrial OS=Homo sapiens OX=9606 GN=MRPL19 PE=1 SV=2	-0,456442631	2,054994
Q9Y5X1	Sorting nexin-9 OS=Homo sapiens OX=9606 GN=SNX9 PE=1 SV=1	0,205424625	2,05263
Q15436	Protein transport protein Sec23A OS=Homo sapiens OX=9606 GN=SEC23A PE=1 SV=2	0,134270314	2,049852
O00115	Deoxyribonuclease-2-alpha OS=Homo sapiens OX=9606 GN=DNASE2 PE=1 SV=2	-0,45536953	2,049821
P51970	NADH dehydrogenase [ubiquinone] 1 alpha subcomplex subunit 8 OS=Homo sapiens OX=9606 GN=NDUFA8 PE=1 SV=3	-0,454893227	2,047525
Q9UG63	ATP-binding cassette sub-family F member 2 OS=Homo sapiens OX=9606 GN=ABCF2 PE=1 SV=2	0,190476155	2,046108
P43007	Neutral amino acid transporter A OS=Homo sapiens OX=9606 GN=SLC1A4 PE=1 SV=1	-0,454579476	2,046012
Q6PGP7	Tetratricopeptide repeat protein 37 OS=Homo sapiens OX=9606 GN=TTC37 PE=1 SV=1	-0,197115633	2,043592
Q15388	Mitochondrial import receptor subunit TOM20 homolog OS=Homo sapiens OX=9606 GN=TOMM20 PE=1 SV=1	-0,592403697	2,04277
Q96AE4	Far upstream element-binding protein 1 OS=Homo sapiens OX=9606 GN=FUBP1 PE=1 SV=3	0,168657779	2,042131
Q12974	Protein tyrosine phosphatase type IVA 2 OS=Homo sapiens OX=9606 GN=PTP4A2 PE=1 SV=1	-0,337781618	2,039229
P83111	Serine beta-lactamase-like protein LACTB, mitochondrial OS=Homo sapiens OX=9606 GN=LACTB PE=1 SV=2	-0,337649548	2,038282
O94856	Neurofascin OS=Homo sapiens OX=9606 GN=NFASC PE=1 SV=4	1,099431069	2,037661
P04040	Catalase OS=Homo sapiens OX=9606 GN=CAT PE=1 SV=3	0,168256954	2,03572
P48147	Prolyl endopeptidase OS=Homo sapiens OX=9606 GN=PREP PE=1 SV=2	-0,15982517	2,033799
P62745	Rho-related GTP-binding protein RhoB OS=Homo sapiens OX=9606 GN=RHOB PE=1 SV=1	0,451403139	2,030687
P0CG30	Glutathione S-transferase theta-2B OS=Homo sapiens OX=9606 GN=GSTT2B PE=1 SV=1	0,451304846	2,030212
O43310	CBP80/20-dependent translation initiation factor OS=Homo sapiens OX=9606 GN=CTIF PE=1 SV=1	0,451208804	2,029749
Q9BQA1	Methylosome protein 50 OS=Homo sapiens OX=9606 GN=WDR77 PE=1 SV=1	0,280274082	2,025164
Q7L5N1	COP9 signalosome complex subunit 6 OS=Homo sapiens OX=9606 GN=COPS6 PE=1 SV=1	0,245672936	2,024792
Q99836	Myeloid differentiation primary response protein MyD88 OS=Homo sapiens OX=9606 GN=MYD88 PE=1 SV=1	1,088570594	2,024708
Q53FA7	Quinone oxidoreductase PIG3 OS=Homo sapiens OX=9606 GN=TP53I3 PE=1 SV=2	-0,232488864	2,023319
P61626	Lysozyme C OS=Homo sapiens OX=9606 GN=LYZ PE=1 SV=1	-0,586316049	2,023001
P78417	Glutathione S-transferase omega-1 OS=Homo sapiens OX=9606 GN=GSTO1 PE=1 SV=2	-0,188761259	2,022041
Q9NWT6	Hypoxia-inducible factor 1-alpha inhibitor OS=Homo sapiens OX=9606 GN=HIF1AN PE=1 SV=2	1,085824102	2,021416
Q8TBA6	Golgin subfamily A member 5 OS=Homo sapiens OX=9606 GN=GOLGA5 PE=1 SV=3	-0,303700563	2,020838
P61081	NEDD8-conjugating enzyme Ubc12 OS=Homo sapiens OX=9606 GN=UBE2M PE=1 SV=1	0,379288719	2,018953
Q9HA65	TBC1 domain family member 17 OS=Homo sapiens OX=9606 GN=TBC1D17 PE=1 SV=2	-1,328576694	2,017891
Q7Z3J2	VPS35 endosomal protein sorting factor-like OS=Homo sapiens OX=9606 GN=VPS35L PE=1 SV=2	0,22064255	2,015129
Q9P0J0	NADH dehydrogenase [ubiquinone] 1 alpha subcomplex subunit 13 OS=Homo sapiens OX=9606 GN=NDUFA13 PE=1 SV=3	-0,582792144	2,011521
Q9UP95	Solute carrier family 12 member 4 OS=Homo sapiens OX=9606 GN=SLC12A4 PE=1 SV=2	0,220143125	2,009269
P31146	Coronin-1A OS=Homo sapiens OX=9606 GN=CORO1A PE=1 SV=4	0,497807126	2,005354
Q99613	Eukaryotic translation initiation factor 3 subunit C OS=Homo sapiens OX=9606 GN=EIF3C PE=1 SV=1	0,132059058	2,004791
P49419	Alpha-aminoadipic semialdehyde dehydrogenase OS=Homo sapiens OX=9606 GN=ALDH7A1 PE=1 SV=5	-0,170807289	2,002774
Q9H446	RWD domain-containing protein 1 OS=Homo sapiens OX=9606 GN=RWDD1 PE=1 SV=1	0,308136094	2,002732
P01892	HLA class I histocompatibility antigen, A-2 alpha chain OS=Homo sapiens OX=9606 GN=HLA-A PE=1 SV=1	-0,219415365	2,000738
O15382	Branched-chain-amino-acid aminotransferase, mitochondrial OS=Homo sapiens OX=9606 GN=BCAT2 PE=1 SV=2	-1,068677219	2,000707
P16930	Fumarylacetoacetase OS=Homo sapiens OX=9606 GN=FAH PE=1 SV=2	-0,376238712	2,000376
P46109	Crk-like protein OS=Homo sapiens OX=9606 GN=CRKL PE=1 SV=1	0,332216542	1,999382
O95302	Peptidyl-prolyl cis-trans isomerase FKBP9 OS=Homo sapiens OX=9606 GN=FKBP9 PE=1 SV=2	-0,170473121	1,997617
Q9HC38	Glyoxalase domain-containing protein 4 OS=Homo sapiens OX=9606 GN=GLOD4 PE=1 SV=1	0,300108604	1,991776
P45974	Ubiquitin carboxyl-terminal hydrolase 5 OS=Homo sapiens OX=9606 GN=USP5 PE=1 SV=2	-0,135936771	1,989287
Q9NS69	Mitochondrial import receptor subunit TOM22 homolog OS=Homo sapiens OX=9606 GN=TOMM22 PE=1 SV=3	0,57578214	1,988607
Q643R3	Lysophospholipid acyltransferase LPCAT4 OS=Homo sapiens OX=9606 GN=LPCAT4 PE=1 SV=1	1,056734495	1,986124
P61978	Heterogeneous nuclear ribonucleoprotein K OS=Homo sapiens OX=9606 GN=HNRNPK PE=1 SV=1	0,143753886	1,986061
P35658	Nuclear pore complex protein Nup214 OS=Homo sapiens OX=9606 GN=NUP214 PE=1 SV=2	0,24181207	1,984567
Q6P995	Protein FAM171B OS=Homo sapiens OX=9606 GN=FAM171B PE=2 SV=3	-0,597323957	1,982862
Q9HC35	Echinoderm microtubule-associated protein-like 4 OS=Homo sapiens OX=9606 GN=EML4 PE=1 SV=3	-0,217846527	1,982384
Q9UNM6	26S proteasome non-ATPase regulatory subunit 13 OS=Homo sapiens OX=9606 GN=PSMD13 PE=1 SV=2	-0,17981832	1,980907
O60925	Prefoldin subunit 1 OS=Homo sapiens OX=9606 GN=PFDN1 PE=1 SV=2	0,298595981	1,979559
Q00169	Phosphatidylinositol transfer protein alpha isoform OS=Homo sapiens OX=9606 GN=PITPNA PE=1 SV=2	0,329437392	1,979515
O95071	E3 ubiquitin-protein ligase UBR5 OS=Homo sapiens OX=9606 GN=UBR5 PE=1 SV=2	-0,674742483	1,974869
P45877	Peptidyl-prolyl cis-trans isomerase C OS=Homo sapiens OX=9606 GN=PPIC PE=1 SV=1	-0,502381352	1,973146

Q14746	Conserved oligomeric Golgi complex subunit 2 OS=Homo sapiens OX=9606 GN=COG2 PE=1 SV=1	-1,046189643	1,973138
A6NKB5	Pecanex-like protein 2 OS=Homo sapiens OX=9606 GN=PCNX2 PE=2 SV=3	1,556135836	1,972057
Q96SY0	Integrator complex subunit 14 OS=Homo sapiens OX=9606 GN=INTS14 PE=1 SV=2	-0,699931916	1,969432
Q9GZT3	SRA stem-loop-interacting RNA-binding protein, mitochondrial OS=Homo sapiens OX=9606 GN=SLIRP PE=1 SV=1	-0,370617712	1,966152
Q14344	Guanine nucleotide-binding protein subunit alpha-13 OS=Homo sapiens OX=9606 GN=GNA13 PE=1 SV=2	-0,327324143	1,964423
P61163	Alpha-centractin OS=Homo sapiens OX=9606 GN=ACTR1A PE=1 SV=1	0,168198295	1,962634
Q9UI12	V-type proton ATPase subunit H OS=Homo sapiens OX=9606 GN=ATP6V1H PE=1 SV=1	0,216131788	1,962377
P30046	D-dopachrome decarboxylase OS=Homo sapiens OX=9606 GN=DDT PE=1 SV=3	-0,327026713	1,9623
O95817	BAG family molecular chaperone regulator 3 OS=Homo sapiens OX=9606 GN=BAG3 PE=1 SV=3	0,178520678	1,962223
Q07021	Complement component 1 Q subcomponent-binding protein, mitochondrial OS=Homo sapiens OX=9606 GN=C1QBP PE=1 SV=1	-0,296173813	1,960021
Q9NTJ3	Structural maintenance of chromosomes protein 4 OS=Homo sapiens OX=9606 GN=SMC4 PE=1 SV=2	-0,215848267	1,959074
Q66K74	Microtubule-associated protein 1S OS=Homo sapiens OX=9606 GN=MAP1S PE=1 SV=2	-0,254420938	1,958803
P20339	Ras-related protein Rab-5A OS=Homo sapiens OX=9606 GN=RAB5A PE=1 SV=2	0,326348998	1,957463
O15514	DNA-directed RNA polymerase II subunit RPB4 OS=Homo sapiens OX=9606 GN=POLR2D PE=1 SV=1	-1,033273702	1,957088
Q13042	Cell division cycle protein 16 homolog OS=Homo sapiens OX=9606 GN=CDC16 PE=1 SV=2	0,566163007	1,956995
Q9H1J7	Protein Wnt-5b OS=Homo sapiens OX=9606 GN=WNT5B PE=2 SV=2	-0,325768537	1,953322
P50452	Serpin B8 OS=Homo sapiens OX=9606 GN=SERPINB8 PE=1 SV=2	-0,226147685	1,953159
P84103	Serine/arginine-rich splicing factor 3 OS=Homo sapiens OX=9606 GN=SRSF3 PE=1 SV=1	0,368272531	1,951879
Q9H7F0	Probable cation-transporting ATPase 13A3 OS=Homo sapiens OX=9606 GN=ATP13A3 PE=1 SV=4	-0,459143533	1,949043
P35749	#N/D	0,225743444	1,948709
Q8IUI8	Cytokine receptor-like factor 3 OS=Homo sapiens OX=9606 GN=CRLF3 PE=1 SV=2	0,409366597	1,94495
O43752	Syntaxin-6 OS=Homo sapiens OX=9606 GN=STX6 PE=1 SV=1	1,023282521	1,944563
O43402	ER membrane protein complex subunit 8 OS=Homo sapiens OX=9606 GN=EMC8 PE=1 SV=1	0,433405547	1,943603
P61619	Protein transport protein Sec61 subunit alpha isoform 1 OS=Homo sapiens OX=9606 GN=SEC61A1 PE=1 SV=2	0,205090134	1,942575
Q8WUI4	Histone deacetylase 7 OS=Homo sapiens OX=9606 GN=HDAC7 PE=1 SV=2	1,019699032	1,940047
P19367	Hexokinase-1 OS=Homo sapiens OX=9606 GN=HK1 PE=1 SV=3	0,115867317	1,938063
P51659	Peroxisomal multifunctional enzyme type 2 OS=Homo sapiens OX=9606 GN=HSD17B4 PE=1 SV=3	0,154012324	1,936554
P62263	40S ribosomal protein S14 OS=Homo sapiens OX=9606 GN=RPS14 PE=1 SV=3	0,251953437	1,935021
Q9UBS4	DnaJ homolog subfamily B member 11 OS=Homo sapiens OX=9606 GN=DNAJB11 PE=1 SV=1	0,29169323	1,923966
O15254	Peroxisomal acyl-coenzyme A oxidase 3 OS=Homo sapiens OX=9606 GN=ACOX3 PE=1 SV=2	-0,321551715	1,923267
P13284	Gamma-interferon-inducible lysosomal thiol reductase OS=Homo sapiens OX=9606 GN=IFI30 PE=1 SV=3	-1,005986119	1,922649
O14949	Cytochrome b-c1 complex subunit 8 OS=Homo sapiens OX=9606 GN=UQCRQ PE=1 SV=4	0,555704513	1,922405
Q70UQ0	Inhibitor of nuclear factor kappa-B kinase-interacting protein OS=Homo sapiens OX=9606 GN=IKBIP PE=1 SV=1	0,175724041	1,922151
Q5RI15	Cytochrome c oxidase assembly protein COX20, mitochondrial OS=Homo sapiens OX=9606 GN=COX20 PE=1 SV=2	1,004137253	1,920289
P27348	14-3-3 protein theta OS=Homo sapiens OX=9606 GN=YWHAQ PE=1 SV=1	0,223071434	1,919364
Q8N9N8	Probable RNA-binding protein EIF1AD OS=Homo sapiens OX=9606 GN=EIF1AD PE=1 SV=1	-0,743284861	1,918575
Q9BY43	Charged multivesicular body protein 4a OS=Homo sapiens OX=9606 GN=CHMP4A PE=1 SV=3	-0,320812988	1,918008
O94875	Sorbin and SH3 domain-containing protein 2 OS=Homo sapiens OX=9606 GN=SORBS2 PE=1 SV=3	0,202850313	1,915288
Q8IZ81	ELMO domain-containing protein 2 OS=Homo sapiens OX=9606 GN=ELMOD2 PE=1 SV=1	0,36222296	1,915079
P11169	Solute carrier family 2, facilitated glucose transporter member 3 OS=Homo sapiens OX=9606 GN=SLC2A3 PE=1 SV=1	0,998349577	1,912879
P51991	Heterogeneous nuclear ribonucleoprotein A3 OS=Homo sapiens OX=9606 GN=HNRNPA3 PE=1 SV=2	0,194525472	1,91258
O00303	Eukaryotic translation initiation factor 3 subunit F OS=Homo sapiens OX=9606 GN=EIF3F PE=1 SV=1	-0,222427115	1,912306
Q9Y266	Nuclear migration protein nudC OS=Homo sapiens OX=9606 GN=NUDC PE=1 SV=1	0,175024683	1,912173
Q5TB80	Centrosomal protein of 162 kDa OS=Homo sapiens OX=9606 GN=CEP162 PE=1 SV=2	0,326511857	1,91178
Q9UHN6	Cell surface hyaluronidase OS=Homo sapiens OX=9606 GN=CEMIP2 PE=1 SV=1	-0,552426707	1,911517
P31350	Ribonucleoside-diphosphate reductase subunit M2 OS=Homo sapiens OX=9606 GN=RRM2 PE=1 SV=1	-0,552049028	1,910261
Q99685	Monoglyceride lipase OS=Homo sapiens OX=9606 GN=MGLL PE=1 SV=2	-0,249165111	1,90823
P00390	Glutathione reductase, mitochondrial OS=Homo sapiens OX=9606 GN=GSR PE=1 SV=2	-0,221983895	1,907455
Q9BPX3	Condensin complex subunit 3 OS=Homo sapiens OX=9606 GN=NCAPG PE=1 SV=1	-0,319086761	1,905725
P61221	ATP-binding cassette sub-family E member 1 OS=Homo sapiens OX=9606 GN=ABCE1 PE=1 SV=1	-0,174366493	1,902797
Q5W111	SPRY domain-containing protein 7 OS=Homo sapiens OX=9606 GN=SPRYD7 PE=1 SV=2	-0,549785927	1,902729
Q9BYN0	Sulfiredoxin-1 OS=Homo sapiens OX=9606 GN=SRXN1 PE=1 SV=2	-0,548716247	1,899165
Q16795	NADH dehydrogenase [ubiquinone] 1 alpha subcomplex subunit 9, mitochondrial OS=Homo sapiens OX=9606 GN=NDUFA9 PE=1 SV=2	0,248009477	1,897152
Q6ZYL4	General transcription factor IIH subunit 5 OS=Homo sapiens OX=9606 GN=GTF2H5 PE=1 SV=1	-0,986111606	1,8971
O95864	Acyl-CoA 6-desaturase OS=Homo sapiens OX=9606 GN=FADS2 PE=1 SV=1	-0,382140514	1,896924
Q92538	Golgi-specific brefeldin A-resistance guanine nucleotide exchange factor 1 OS=Homo sapiens OX=9606 GN=GBF1 PE=1 SV=2	0,148090938	1,896791
P30825	High affinity cationic amino acid transporter 1 OS=Homo sapiens OX=9606 GN=SLC7A1 PE=1 SV=1	-0,423638117	1,89618
Q9H4A6	Golgi phosphoprotein 3 OS=Homo sapiens OX=9606 GN=GOLPH3 PE=1 SV=1	0,247889047	1,895998
Q8NBQ5	Estradiol 17-beta-dehydrogenase 11 OS=Homo sapiens OX=9606 GN=HSD17B11 PE=1 SV=3	0,317624283	1,895326
P52298	Nuclear cap-binding protein subunit 2 OS=Homo sapiens OX=9606 GN=NCBP2 PE=1 SV=1	0,984229674	1,89466
Q9BQ67	Glutamate-rich WD repeat-containing protein 1 OS=Homo sapiens OX=9606 GN=GRWD1 PE=1 SV=1	0,422971302	1,892939
P53365	Arfaptin-2 OS=Homo sapiens OX=9606 GN=ARFIP2 PE=1 SV=1	-0,546069595	1,890337
Q16822	Phosphoenolpyruvate carboxykinase [GTP], mitochondrial OS=Homo sapiens OX=9606 GN=PCK2 PE=1 SV=4	-0,147664445	1,889595
Q8WZ82	Esterase OVCA2 OS=Homo sapiens OX=9606 GN=OVCA2 PE=1 SV=1	0,316404375	1,886658
Q99543	DnaJ homolog subfamily C member 2 OS=Homo sapiens OX=9606 GN=DNAJC2 PE=1 SV=4	-0,421000023	1,883354
O60216	Double-strand-break repair protein rad21 homolog OS=Homo sapiens OX=9606 GN=RAD21 PE=1 SV=2	0,420989146	1,883301
Q9BV68	E3 ubiquitin-protein ligase RNF126 OS=Homo sapiens OX=9606 GN=RNF126 PE=1 SV=2	-0,971300752	1,877798
Q9Y6C9	Mitochondrial carrier homolog 2 OS=Homo sapiens OX=9606 GN=MTCH2 PE=1 SV=1	-0,263622384	1,877264
Q9NTI5	Sister chromatid cohesion protein PDS5 homolog B OS=Homo sapiens OX=9606 GN=PDS5B PE=1 SV=1	0,263597504	1,877045
Q96LJ7	Dehydrogenase/reductase SDR family member 1 OS=Homo sapiens OX=9606 GN=DHRS1 PE=1 SV=1	-0,541963381	1,876613
Q05397	Focal adhesion kinase 1 OS=Homo sapiens OX=9606 GN=PTK2 PE=1 SV=2	0,69022394	1,875776
P55209	Nucleosome assembly protein 1-like 1 OS=Homo sapiens OX=9606 GN=NAP1L1 PE=1 SV=1	0,231248556	1,875564
Q9P2R7	Succinate--CoA ligase [ADP-forming] subunit beta, mitochondrial OS=Homo sapiens OX=9606 GN=SUCLA2 PE=1 SV=3	-0,245694872	1,875009
Q9BXB4	Oxysterol-binding protein-related protein 11 OS=Homo sapiens OX=9606 GN=OSBPL11 PE=1 SV=2	-1,08971948	1,873748
Q96QZ7	Membrane-associated guanylate kinase, WW and PDZ domain-containing protein 1 OS=Homo sapiens OX=9606 GN=MAGI1 PE=1 SV=3	-1,708745643	1,873561
Q96S06	Lipase maturation factor 1 OS=Homo sapiens OX=9606 GN=LMF1 PE=1 SV=1	1,069253869	1,872537
Q9Y4C2	TRPM8 channel-associated factor 1 OS=Homo sapiens OX=9606 GN=TCAF1 PE=1 SV=3	-0,418426422	1,870835
Q9P000	COMM domain-containing protein 9 OS=Homo sapiens OX=9606 GN=COMMD9 PE=1 SV=2	-0,284864791	1,86924
Q8WUA7	TBC1 domain family member 22A OS=Homo sapiens OX=9606 GN=TBC1D22A PE=1 SV=2	0,571795843	1,869069
O14983	Sarcoplasmic/endoplasmic reticulum calcium ATPase 1 OS=Homo sapiens OX=9606 GN=ATP2A1 PE=1 SV=1	1,358748948	1,866604
P60520	Gamma-aminobutyric acid receptor-associated protein-like 2 OS=Homo sapiens OX=9606 GN=GABARAPL2 PE=1 SV=1	0,417345254	1,865574
O95070	Protein YIF1A OS=Homo sapiens OX=9606 GN=YIF1A PE=1 SV=2	0,538308232	1,864367
P63279	SUMO-conjugating enzyme UBC9 OS=Homo sapiens OX=9606 GN=UBE2I PE=1 SV=1	-0,262100593	1,863865
O76094	Signal recognition particle subunit SRP72 OS=Homo sapiens OX=9606 GN=SRP72 PE=1 SV=3	0,177248542	1,862849
O60784	Target of Myb protein 1 OS=Homo sapiens OX=9606 GN=TOM1 PE=1 SV=2	-0,177217267	1,862422
P41250	Glycine--tRNA ligase OS=Homo sapiens OX=9606 GN=GARS PE=1 SV=3	-0,120918674	1,861519
Q9Y3B3	Transmembrane emp24 domain-containing protein 7 OS=Homo sapiens OX=9606 GN=TMED7 PE=1 SV=2	0,283789639	1,860649
P62633	Cellular nucleic acid-binding protein OS=Homo sapiens OX=9606 GN=CNBP PE=1 SV=1	-0,31217774	1,856662
Q96CS3	FAS-associated factor 2 OS=Homo sapiens OX=9606 GN=FAF2 PE=1 SV=2	0,229329915	1,855935
Q9H6X2	Anthrax toxin receptor 1 OS=Homo sapiens OX=9606 GN=ANTXR1 PE=1 SV=2	-0,535710137	1,855645
Q9Y2G5	GDP-fucose protein O-fucosyltransferase 2 OS=Homo sapiens OX=9606 GN=POFUT2 PE=1 SV=3	0,206885669	1,855498
Q08945	FACT complex subunit SSRP1 OS=Homo sapiens OX=9606 GN=SSRP1 PE=1 SV=1	-0,260981733	1,854026
Q8TAT6	Nuclear protein localization protein 4 homolog OS=Homo sapiens OX=9606 GN=NPLOC4 PE=1 SV=3	0,206641837	1,852703
Q13733	#N/D	0,351725587	1,851299
Q9Y679	Ancient ubiquitous protein 1 OS=Homo sapiens OX=9606 GN=AUP1 PE=1 SV=2	0,95048319	1,850279
Q96A26	Protein FAM162A OS=Homo sapiens OX=9606 GN=FAM162A PE=1 SV=2	0,950034918	1,849681
Q12905	Interleukin enhancer-binding factor 2 OS=Homo sapiens OX=9606 GN=ILF2 PE=1 SV=2	-0,216398399	1,84661
Q9BWH6	RNA polymerase II-associated protein 1 OS=Homo sapiens OX=9606 GN=RPAP1 PE=1 SV=3	-0,057525467	1,845384

P21266	Glutathione S-transferase Mu 3 OS=Homo sapiens OX=9606 GN=GSTM3 PE=1 SV=3	0,175953439	1,845203
O95571	Persulfide dioxygenase ETHE1, mitochondrial OS=Homo sapiens OX=9606 GN=ETHE1 PE=1 SV=2	-0,310530774	1,844991
Q8N573	Oxidation resistance protein 1 OS=Homo sapiens OX=9606 GN=OXR1 PE=1 SV=2	-0,412975536	1,844299
Q8TDY2	RB1-inducible coiled-coil protein 1 OS=Homo sapiens OX=9606 GN=RB1CC1 PE=1 SV=3	1,329183286	1,844052
Q99460	26S proteasome non-ATPase regulatory subunit 1 OS=Homo sapiens OX=9606 GN=PSMD1 PE=1 SV=2	0,123958096	1,842886
Q9H000	Probable E3 ubiquitin-protein ligase makorin-2 OS=Homo sapiens OX=9606 GN=MKRN2 PE=1 SV=2	-0,531786415	1,842449
Q9Y5L0	Transportin-3 OS=Homo sapiens OX=9606 GN=TNPO3 PE=1 SV=3	0,242193424	1,84163
Q8N556	Actin filament-associated protein 1 OS=Homo sapiens OX=9606 GN=AFAP1 PE=1 SV=2	-0,205581562	1,840561
Q13363	C-terminal-binding protein 1 OS=Homo sapiens OX=9606 GN=CTBP1 PE=1 SV=2	-0,349558152	1,838145
P49768	Presenilin-1 OS=Homo sapiens OX=9606 GN=PSEN1 PE=1 SV=1	-0,431390204	1,838051
P82650	28S ribosomal protein S22, mitochondrial OS=Homo sapiens OX=9606 GN=MRPS22 PE=1 SV=1	-0,349459481	1,837546
Q8WWX9	Selenoprotein M OS=Homo sapiens OX=9606 GN=SELENOM PE=1 SV=3	-0,411371593	1,836486
Q8WXI9	Transcriptional repressor p66-beta OS=Homo sapiens OX=9606 GN=GATAD2B PE=1 SV=1	0,133245139	1,834783
Q9Y2E5	Epididymis-specific alpha-mannosidase OS=Homo sapiens OX=9606 GN=MAN2B2 PE=1 SV=4	-0,938265758	1,833913
Q7Z478	ATP-dependent RNA helicase DHX29 OS=Homo sapiens OX=9606 GN=DHX29 PE=1 SV=2	0,258617342	1,833271
P38571	Lysosomal acid lipase/cholesteryl ester hydrolase OS=Homo sapiens OX=9606 GN=LIPA PE=1 SV=2	-0,936349917	1,831332
O14980	Exportin-1 OS=Homo sapiens OX=9606 GN=XPO1 PE=1 SV=1	0,115837097	1,830923
Q9Y4W6	AFG3-like protein 2 OS=Homo sapiens OX=9606 GN=AFG3L2 PE=1 SV=2	-0,258122091	1,82893
P36915	Guanine nucleotide-binding protein-like 1 OS=Homo sapiens OX=9606 GN=GNL1 PE=1 SV=2	-0,347887315	1,828008
P07951	Tropomyosin beta chain OS=Homo sapiens OX=9606 GN=TPM2 PE=1 SV=1	0,279586371	1,827129
Q9UPN7	Serine/threonine-protein phosphatase 6 regulatory subunit 1 OS=Homo sapiens OX=9606 GN=PPP6R1 PE=1 SV=5	-0,347699045	1,826866
Q8WUH1	Protein Churchill OS=Homo sapiens OX=9606 GN=CHURC1 PE=1 SV=2	-1,05305369	1,823469
Q13123	Protein Red OS=Homo sapiens OX=9606 GN=IK PE=1 SV=3	-0,408300044	1,821516
O43169	Cytochrome b5 type B OS=Homo sapiens OX=9606 GN=CYB5B PE=1 SV=3	-0,346483317	1,819493
P11498	Pyruvate carboxylase, mitochondrial OS=Homo sapiens OX=9606 GN=PC PE=1 SV=2	-0,187104467	1,818966
Q6ZSR9	Uncharacterized protein FLJ45252 OS=Homo sapiens OX=9606 PE=2 SV=2	0,407721871	1,818698
Q4V9L6	Transmembrane protein 119 OS=Homo sapiens OX=9606 GN=TMEM119 PE=1 SV=1	0,346338369	1,818614
Q8NF37	Lysophosphatidylcholine acyltransferase 1 OS=Homo sapiens OX=9606 GN=LPCAT1 PE=1 SV=2	0,524014348	1,816217
P51531	Probable global transcription activator SNF2L2 OS=Homo sapiens OX=9606 GN=SMARCA2 PE=1 SV=2	0,333591044	1,813858
Q14934	Nuclear factor of activated T-cells, cytoplasmic 4 OS=Homo sapiens OX=9606 GN=NFATC4 PE=1 SV=2	-0,305289625	1,807914
P25705	ATP synthase subunit alpha, mitochondrial OS=Homo sapiens OX=9606 GN=ATP5F1A PE=1 SV=1	0,126434445	1,80703
Q16706	Alpha-mannosidase 2 OS=Homo sapiens OX=9606 GN=MAN2A1 PE=1 SV=2	-0,521234776	1,806806
P12110	Collagen alpha-2(VI) chain OS=Homo sapiens OX=9606 GN=COL6A2 PE=1 SV=4	-0,142711199	1,806733
O15321	Transmembrane 9 superfamily member 1 OS=Homo sapiens OX=9606 GN=TM9SF1 PE=2 SV=2	0,917680478	1,805968
P20674	Cytochrome c oxidase subunit 5A, mitochondrial OS=Homo sapiens OX=9606 GN=COX5A PE=1 SV=2	-0,276687295	1,804074
Q68CQ7	Glycosyltransferase 8 domain-containing protein 1 OS=Homo sapiens OX=9606 GN=GLT8D1 PE=1 SV=2	-0,915456771	1,802921
Q15717	ELAV-like protein 1 OS=Homo sapiens OX=9606 GN=ELAVL1 PE=1 SV=2	0,223779984	1,799459
P09651	Heterogeneous nuclear ribonucleoprotein A1 OS=Homo sapiens OX=9606 GN=HNRNPA1 PE=1 SV=5	-0,185444294	1,798221
P07947	Tyrosine-protein kinase Yes OS=Homo sapiens OX=9606 GN=YES1 PE=1 SV=3	-0,342810844	1,79723
Q96JB1	Dynein heavy chain 8, axonemal OS=Homo sapiens OX=9606 GN=DNAH8 PE=1 SV=2	0,247607185	1,796023
Q8TD55	Pleckstrin homology domain-containing family O member 2 OS=Homo sapiens OX=9606 GN=PLEKHO2 PE=1 SV=1	0,303539965	1,795559
P00374	Dihydrofolate reductase OS=Homo sapiens OX=9606 GN=DHFR PE=1 SV=2	-0,908548509	1,793419
Q9Y3C6	Peptidyl-prolyl cis-trans isomerase-like 1 OS=Homo sapiens OX=9606 GN=PPIL1 PE=1 SV=1	0,402533983	1,793395
Q14534	Squalene monooxygenase OS=Homo sapiens OX=9606 GN=SQLE PE=1 SV=3	0,453567032	1,788058
Q96P47	Arf-GAP with GTPase, ANK repeat and PH domain-containing protein 3 OS=Homo sapiens OX=9606 GN=AGAP3 PE=1 SV=2	0,459321375	1,786624
Q6UWP7	Lysocardiolipin acyltransferase 1 OS=Homo sapiens OX=9606 GN=LCLAT1 PE=1 SV=1	0,515158883	1,786182
P80723	Brain acid soluble protein 1 OS=Homo sapiens OX=9606 GN=BASP1 PE=1 SV=2	0,177676101	1,786076
Q8TEA8	D-aminoacyl-tRNA deacylase 1 OS=Homo sapiens OX=9606 GN=DTD1 PE=1 SV=2	-0,515119718	1,786048
Q9UKV8	Protein argonaute-2 OS=Homo sapiens OX=9606 GN=AGO2 PE=1 SV=3	-0,340964964	1,786048
P00568	Adenylate kinase isoenzyme 1 OS=Homo sapiens OX=9606 GN=AK1 PE=1 SV=3	0,184265789	1,783539
Q9UJM3	ERBB receptor feedback inhibitor 1 OS=Homo sapiens OX=9606 GN=ERRFI1 PE=1 SV=1	-0,293502598	1,782225
P78357	Contactin-associated protein 1 OS=Homo sapiens OX=9606 GN=CNTNAP1 PE=1 SV=1	-0,438115759	1,781453
P51571	Translocon-associated protein subunit delta OS=Homo sapiens OX=9606 GN=SSR4 PE=1 SV=1	0,301434463	1,780707
Q9C0E2	Exportin-4 OS=Homo sapiens OX=9606 GN=XPO4 PE=1 SV=2	-0,3997117	1,779622
O60673	DNA polymerase zeta catalytic subunit OS=Homo sapiens OX=9606 GN=REV3L PE=1 SV=2	-0,536276514	1,777509
Q8IU81	Interferon regulatory factor 2-binding protein 1 OS=Homo sapiens OX=9606 GN=IRF2BP1 PE=1 SV=1	1,352225192	1,776172
Q04837	Single-stranded DNA-binding protein, mitochondrial OS=Homo sapiens OX=9606 GN=SSBP1 PE=1 SV=1	-0,398984124	1,77607
Q16643	Drebrin OS=Homo sapiens OX=9606 GN=DBN1 PE=1 SV=4	0,159774843	1,767331
O60701	UDP-glucose 6-dehydrogenase OS=Homo sapiens OX=9606 GN=UGDH PE=1 SV=1	0,114444441	1,766541
Q15276	Rab GTPase-binding effector protein 1 OS=Homo sapiens OX=9606 GN=RABEP1 PE=1 SV=2	0,337368366	1,764272
O43491	Band 4.1-like protein 2 OS=Homo sapiens OX=9606 GN=EPB41L2 PE=1 SV=1	-0,131413369	1,764079
O43707	Alpha-actinin-4 OS=Homo sapiens OX=9606 GN=ACTN4 PE=1 SV=2	-0,095717267	1,763119
Q9UEU0	Vesicle transport through interaction with t-SNAREs homolog 1B OS=Homo sapiens OX=9606 GN=VTI1B PE=1 SV=3	-0,336593499	1,759583
P35813	Protein phosphatase 1A OS=Homo sapiens OX=9606 GN=PPM1A PE=1 SV=1	0,298127946	1,757418
O95445	Apolipoprotein M OS=Homo sapiens OX=9606 GN=APOM PE=1 SV=2	0,882420695	1,75699
P41743	Protein kinase C iota type OS=Homo sapiens OX=9606 GN=PRKCI PE=1 SV=2	0,394190837	1,752662
Q5TAQ9	DDB1- and CUL4-associated factor 8 OS=Homo sapiens OX=9606 GN=DCAF8 PE=1 SV=1	-0,505093851	1,751856
O60684	Importin subunit alpha-7 OS=Homo sapiens OX=9606 GN=KPNA6 PE=1 SV=1	0,335228397	1,751325
P62495	Eukaryotic peptide chain release factor subunit 1 OS=Homo sapiens OX=9606 GN=ETF1 PE=1 SV=3	0,163543009	1,750791
P61604	10 kDa heat shock protein, mitochondrial OS=Homo sapiens OX=9606 GN=HSPE1 PE=1 SV=2	0,249151431	1,750689
Q9Y312	Protein AAR2 homolog OS=Homo sapiens OX=9606 GN=AAR2 PE=1 SV=2	0,87786593	1,750557
Q8WUM4	Programmed cell death 6-interacting protein OS=Homo sapiens OX=9606 GN=PDCD6IP PE=1 SV=1	0,100706295	1,750255
P19338	Nucleolin OS=Homo sapiens OX=9606 GN=NCL PE=1 SV=3	0,136197253	1,749985
Q6EEV4	DNA-directed RNA polymerase II subunit GRINL1A, isoforms 4/5 OS=Homo sapiens OX=9606 GN=POLR2M PE=1 SV=1	0,877333304	1,749804
P40855	Peroxisomal biogenesis factor 19 OS=Homo sapiens OX=9606 GN=PEX19 PE=1 SV=1	0,297038464	1,749754
Q9NZN4	EH domain-containing protein 2 OS=Homo sapiens OX=9606 GN=EHD2 PE=1 SV=2	0,123147146	1,745463
Q8IWB9	Testis-expressed protein 2 OS=Homo sapiens OX=9606 GN=TEX2 PE=1 SV=2	0,149018425	1,743939
Q99653	Calcineurin B homologous protein 1 OS=Homo sapiens OX=9606 GN=CHP1 PE=1 SV=3	-0,793137248	1,742497
O75746	Calcium-binding mitochondrial carrier protein Aralar1 OS=Homo sapiens OX=9606 GN=SLC25A12 PE=1 SV=2	-0,295975914	1,742284
P07910	Heterogeneous nuclear ribonucleoproteins C1/C2 OS=Homo sapiens OX=9606 GN=HNRNPC PE=1 SV=4	0,168243999	1,741281
Q8WVX9	Fatty acyl-CoA reductase 1 OS=Homo sapiens OX=9606 GN=FAR1 PE=1 SV=1	-0,870987346	1,740797
Q9BSJ2	Gamma-tubulin complex component 2 OS=Homo sapiens OX=9606 GN=TUBGCP2 PE=1 SV=2	-0,268657305	1,740501
Q13158	FAS-associated death domain protein OS=Homo sapiens OX=9606 GN=FADD PE=1 SV=1	-0,39152306	1,739628
Q15006	ER membrane protein complex subunit 2 OS=Homo sapiens OX=9606 GN=EMC2 PE=1 SV=1	0,501173247	1,738433
P35637	RNA-binding protein FUS OS=Homo sapiens OX=9606 GN=FUS PE=1 SV=1	-0,332597231	1,735415
Q9Y6B6	GTP-binding protein SAR1b OS=Homo sapiens OX=9606 GN=SAR1B PE=1 SV=1	0,294620327	1,73276
P63208	S-phase kinase-associated protein 1 OS=Homo sapiens OX=9606 GN=SKP1 PE=1 SV=2	0,196070812	1,732687
Q9P289	Serine/threonine-protein kinase 26 OS=Homo sapiens OX=9606 GN=STK26 PE=1 SV=2	-1,669572998	1,732455
Q6UN15	Pre-mRNA 3'-end-processing factor FIP1 OS=Homo sapiens OX=9606 GN=FIP1L1 PE=1 SV=1	0,389875598	1,731577
P19883	Follistatin OS=Homo sapiens OX=9606 GN=FST PE=1 SV=2	0,643250619	1,731523
Q96RT1	Erbin OS=Homo sapiens OX=9606 GN=ERBIN PE=1 SV=2	-0,162108724	1,730958
Q14839	Chromodomain-helicase-DNA-binding protein 4 OS=Homo sapiens OX=9606 GN=CHD4 PE=1 SV=2	-0,216898519	1,730069
Q12769	Nuclear pore complex protein Nup160 OS=Homo sapiens OX=9606 GN=NUP160 PE=1 SV=3	-0,246671499	1,729192
P26583	High mobility group protein B2 OS=Homo sapiens OX=9606 GN=HMGB2 PE=1 SV=2	-0,230223817	1,728627
Q9Y3D9	28S ribosomal protein S23, mitochondrial OS=Homo sapiens OX=9606 GN=MRPS23 PE=1 SV=2	-0,862299723	1,728389
P52735	Guanine nucleotide exchange factor VAV2 OS=Homo sapiens OX=9606 GN=VAV2 PE=1 SV=2	-0,33129318	1,727534
P54819	Adenylate kinase 2, mitochondrial OS=Homo sapiens OX=9606 GN=AK2 PE=1 SV=2	-0,205318382	1,727532
P11532	Dystrophin OS=Homo sapiens OX=9606 GN=DMD PE=1 SV=3	0,185882139	1,727162

P16435	NADPH--cytochrome P450 reductase OS=Homo sapiens OX=9606 GN=POR PE=1 SV=2	0,187084983	1,72651
Q9UPY8	Microtubule-associated protein RP/EB family member 3 OS=Homo sapiens OX=9606 GN=MAPRE3 PE=1 SV=1	0,266469032	1,72325
Q9NP84	Tumor necrosis factor receptor superfamily member 12A OS=Homo sapiens OX=9606 GN=TNFRSF12A PE=1 SV=1	-0,248999712	1,723104
P42126	Enoyl-CoA delta isomerase 1, mitochondrial OS=Homo sapiens OX=9606 GN=ECI1 PE=1 SV=1	-0,245965675	1,723084
Q9NP81	Serine--tRNA ligase, mitochondrial OS=Homo sapiens OX=9606 GN=SARS2 PE=1 SV=1	0,471863823	1,719858
Q14914	Prostaglandin reductase 1 OS=Homo sapiens OX=9606 GN=PTGR1 PE=1 SV=2	0,204540859	1,719258
Q14011	Cold-inducible RNA-binding protein OS=Homo sapiens OX=9606 GN=CIRBP PE=1 SV=1	-0,387242648	1,718707
Q09666	Neuroblast differentiation-associated protein AHNAK OS=Homo sapiens OX=9606 GN=AHNAK PE=1 SV=2	0,037957508	1,717422
P28072	Proteasome subunit beta type-6 OS=Homo sapiens OX=9606 GN=PSMB6 PE=1 SV=4	-0,292171199	1,715574
Q93096	Protein tyrosine phosphatase type IVA 1 OS=Homo sapiens OX=9606 GN=PTP4A1 PE=1 SV=2	0,227102417	1,71545
Q9UQ13	Leucine-rich repeat protein SHOC-2 OS=Homo sapiens OX=9606 GN=SHOC2 PE=1 SV=2	-0,265433216	1,715097
Q93083	Metallothionein-1L OS=Homo sapiens OX=9606 GN=MT1L PE=2 SV=1	-0,7167102	1,712544
P13473	Lysosome-associated membrane glycoprotein 2 OS=Homo sapiens OX=9606 GN=LAMP2 PE=1 SV=2	0,328300477	1,709459
Q6NUQ4	Transmembrane protein 214 OS=Homo sapiens OX=9606 GN=TMEM214 PE=1 SV=2	0,165842109	1,709299
O75323	Protein NipSnap homolog 2 OS=Homo sapiens OX=9606 GN=NIPSNAP2 PE=1 SV=1	0,847315602	1,706774
Q9BRK5	45 kDa calcium-binding protein OS=Homo sapiens OX=9606 GN=SDF4 PE=1 SV=1	0,214518629	1,706239
Q12907	Vesicular integral-membrane protein VIP36 OS=Homo sapiens OX=9606 GN=LMAN2 PE=1 SV=1	0,203229385	1,705328
Q03154	Aminoacylase-1 OS=Homo sapiens OX=9606 GN=ACY1 PE=1 SV=1	-0,845913279	1,704737
Q96AB3	Isochorismatase domain-containing protein 2 OS=Homo sapiens OX=9606 GN=ISOC2 PE=1 SV=1	0,29030446	1,702491
Q86XA9	HEAT repeat-containing protein 5A OS=Homo sapiens OX=9606 GN=HEATR5A PE=1 SV=2	0,263673346	1,70126
Q8N5C1	Calcium homeostasis modulator protein 5 OS=Homo sapiens OX=9606 GN=CALHM5 PE=2 SV=1	0,383595834	1,700875
O14498	Immunoglobulin superfamily containing leucine-rich repeat protein OS=Homo sapiens OX=9606 GN=ISLR PE=2 SV=1	0,840270372	1,696515
P62081	40S ribosomal protein S7 OS=Homo sapiens OX=9606 GN=RPS7 PE=1 SV=1	0,226782976	1,696468
Q969X1	Protein lifeguard 3 OS=Homo sapiens OX=9606 GN=TMBIM1 PE=1 SV=2	0,839172052	1,694911
Q9UIA9	Exportin-7 OS=Homo sapiens OX=9606 GN=XPO7 PE=1 SV=3	-0,226542266	1,694224
P09496	Clathrin light chain A OS=Homo sapiens OX=9606 GN=CLTA PE=1 SV=1	-0,2890903	1,693989
Q9UMX5	Neudesin OS=Homo sapiens OX=9606 GN=NENF PE=1 SV=1	-0,382078164	1,693453
Q9UHQ9	NADH-cytochrome b5 reductase 1 OS=Homo sapiens OX=9606 GN=CYB5R1 PE=1 SV=1	0,213113511	1,69221
Q8TDX7	Serine/threonine-protein kinase Nek7 OS=Homo sapiens OX=9606 GN=NEK7 PE=1 SV=1	0,154490618	1,692003
Q00325	Phosphate carrier protein, mitochondrial OS=Homo sapiens OX=9606 GN=SLC25A3 PE=1 SV=2	0,176842384	1,691909
Q9NR31	GTP-binding protein SAR1a OS=Homo sapiens OX=9606 GN=SAR1A PE=1 SV=1	-0,201957476	1,691848
O75531	Barrier-to-autointegration factor OS=Homo sapiens OX=9606 GN=BANF1 PE=1 SV=1	-0,487328175	1,690794
O43598	2'-deoxynucleoside 5'-phosphate N-hydrolase 1 OS=Homo sapiens OX=9606 GN=DNPH1 PE=1 SV=1	-0,325117718	1,690252
Q8N5G2	Macoilin OS=Homo sapiens OX=9606 GN=MACO1 PE=1 SV=1	-0,835248637	1,689166
Q96I24	Far upstream element-binding protein 3 OS=Homo sapiens OX=9606 GN=FUBP3 PE=1 SV=2	-0,192065089	1,687819
Q92499	ATP-dependent RNA helicase DDX1 OS=Homo sapiens OX=9606 GN=DDX1 PE=1 SV=2	-0,105815431	1,687473
Q9NZV1	Cysteine-rich motor neuron 1 protein OS=Homo sapiens OX=9606 GN=CRIM1 PE=1 SV=1	0,261384394	1,683296
P50990	T-complex protein 1 subunit theta OS=Homo sapiens OX=9606 GN=CCT8 PE=1 SV=4	0,100152096	1,682484
P37235	Hippocalcin-like protein 1 OS=Homo sapiens OX=9606 GN=HPCAL1 PE=1 SV=3	-0,261156828	1,681512
Q68DQ2	Very large A-kinase anchor protein OS=Homo sapiens OX=9606 GN=CRYBG3 PE=1 SV=3	0,095765361	1,681502
P30048	Thioredoxin-dependent peroxide reductase, mitochondrial OS=Homo sapiens OX=9606 GN=PRDX3 PE=1 SV=3	0,241029824	1,680508
Q9UNE7	E3 ubiquitin-protein ligase CHIP OS=Homo sapiens OX=9606 GN=STUB1 PE=1 SV=2	0,200684802	1,67839
O15144	Actin-related protein 2/3 complex subunit 2 OS=Homo sapiens OX=9606 GN=ARPC2 PE=1 SV=1	0,129051453	1,677225
Q8NBF2	NHL repeat-containing protein 2 OS=Homo sapiens OX=9606 GN=NHLRC2 PE=1 SV=1	0,260558486	1,676823
P31327	Carbamoyl-phosphate synthase [ammonia], mitochondrial OS=Homo sapiens OX=9606 GN=CPS1 PE=1 SV=2	0,825986698	1,675527
Q9BUL8	Programmed cell death protein 10 OS=Homo sapiens OX=9606 GN=PDCD10 PE=1 SV=1	-0,224476309	1,674993
P25445	Tumor necrosis factor receptor superfamily member 6 OS=Homo sapiens OX=9606 GN=FAS PE=1 SV=1	0,286191688	1,67372
O60220	Mitochondrial import inner membrane translocase subunit Tim8 A OS=Homo sapiens OX=9606 GN=TIMM8A PE=1 SV=1	0,824646849	1,673545
P18085	ADP-ribosylation factor 4 OS=Homo sapiens OX=9606 GN=ARF4 PE=1 SV=3	0,224247765	1,672868
P78346	Ribonuclease P protein subunit p30 OS=Homo sapiens OX=9606 GN=RPP30 PE=1 SV=1	-0,481964669	1,672243
P53582	Methionine aminopeptidase 1 OS=Homo sapiens OX=9606 GN=METAP1 PE=1 SV=2	0,224179622	1,672235
Q9H857	5'-nucleotidase domain-containing protein 2 OS=Homo sapiens OX=9606 GN=NT5DC2 PE=1 SV=1	-0,481528344	1,670731
Q9Y3C4	EKC/KEOPS complex subunit TPRKB OS=Homo sapiens OX=9606 GN=TPRKB PE=1 SV=1	-0,481505848	1,670653
Q71U36	Tubulin alpha-1A chain OS=Homo sapiens OX=9606 GN=TUBA1A PE=1 SV=1	0,105004707	1,670524
P52789	Hexokinase-2 OS=Homo sapiens OX=9606 GN=HK2 PE=1 SV=2	0,157676519	1,670136
P00492	Hypoxanthine-guanine phosphoribosyltransferase OS=Homo sapiens OX=9606 GN=HPRT1 PE=1 SV=2	-0,223826542	1,668955
Q02880	DNA topoisomerase 2-beta OS=Homo sapiens OX=9606 GN=TOP2B PE=1 SV=3	0,376968295	1,668454
P22830	Ferrochelatase, mitochondrial OS=Homo sapiens OX=9606 GN=FECH PE=1 SV=2	-0,48083907	1,668343
Q8IWA4	#N/D	-0,166552329	1,668224
Q9NQR4	Omega-amidase NIT2 OS=Homo sapiens OX=9606 GN=NIT2 PE=1 SV=1	-0,199606259	1,667008
P31025	Lipocalin-1 OS=Homo sapiens OX=9606 GN=LCN1 PE=1 SV=1	-0,819641544	1,666121
O95166	Gamma-aminobutyric acid receptor-associated protein OS=Homo sapiens OX=9606 GN=GABARAP PE=1 SV=1	1,773200424	1,665006
Q8WUX9	Charged multivesicular body protein 7 OS=Homo sapiens OX=9606 GN=CHMP7 PE=1 SV=1	0,375930663	1,663377
O15460	Prolyl 4-hydroxylase subunit alpha-2 OS=Homo sapiens OX=9606 GN=P4HA2 PE=1 SV=1	-0,125566973	1,662204
P62304	Small nuclear ribonucleoprotein E OS=Homo sapiens OX=9606 GN=SNRPE PE=1 SV=1	-0,478557871	1,660431
Q8TCT8	Signal peptide peptidase-like 2A OS=Homo sapiens OX=9606 GN=SPPL2A PE=1 SV=2	-0,815419941	1,659835
Q05655	Protein kinase C delta type OS=Homo sapiens OX=9606 GN=PRKCD PE=1 SV=2	-0,930149636	1,659049
O43324	Eukaryotic translation elongation factor 1 epsilon-1 OS=Homo sapiens OX=9606 GN=EEF1E1 PE=1 SV=1	-0,319869741	1,658623
P35754	Glutaredoxin-1 OS=Homo sapiens OX=9606 GN=GLRX PE=1 SV=2	-0,283740972	1,656611
A6NDU8	UPF0600 protein C5orf51 OS=Homo sapiens OX=9606 GN=C5orf51 PE=1 SV=1	0,534230627	1,655638
P61018	Ras-related protein Rab-4B OS=Homo sapiens OX=9606 GN=RAB4B PE=1 SV=1	0,575164933	1,653881
P46976	Glycogenin-1 OS=Homo sapiens OX=9606 GN=GYG1 PE=1 SV=4	0,373710407	1,652511
Q14315	Filamin-C OS=Homo sapiens OX=9606 GN=FLNC PE=1 SV=3	0,06733266	1,651524
P68400	Casein kinase II subunit alpha OS=Homo sapiens OX=9606 GN=CSNK2A1 PE=1 SV=1	-0,237612596	1,651173
Q14554	Protein disulfide-isomerase A5 OS=Homo sapiens OX=9606 GN=PDIA5 PE=1 SV=1	-0,167069303	1,650225
O14735	CDP-diacylglycerol--inositol 3-phosphatidyltransferase OS=Homo sapiens OX=9606 GN=CDIPT PE=1 SV=1	0,808353257	1,64926
Q96A35	39S ribosomal protein L24, mitochondrial OS=Homo sapiens OX=9606 GN=MRPL24 PE=1 SV=1	-0,475244376	1,648924
O60271	C-Jun-amino-terminal kinase-interacting protein 4 OS=Homo sapiens OX=9606 GN=SPAG9 PE=1 SV=4	0,108583832	1,648693
Q96T76	MMS19 nucleotide excision repair protein homolog OS=Homo sapiens OX=9606 GN=MMS19 PE=1 SV=2	0,1884976	1,648148
Q92879	CUGBP Elav-like family member 1 OS=Homo sapiens OX=9606 GN=CELF1 PE=1 SV=2	0,256880227	1,648056
Q92544	Transmembrane 9 superfamily member 4 OS=Homo sapiens OX=9606 GN=TM9SF4 PE=1 SV=2	0,237226289	1,647864
Q9H2P9	Diphthine methyl ester synthase OS=Homo sapiens OX=9606 GN=DPH5 PE=1 SV=2	-0,663675541	1,646154
O75521	Enoyl-CoA delta isomerase 2, mitochondrial OS=Homo sapiens OX=9606 GN=ECI2 PE=1 SV=4	0,282190449	1,645801
P13693	Translationally-controlled tumor protein OS=Homo sapiens OX=9606 GN=TPT1 PE=1 SV=1	0,180034555	1,643998
O75027	ATP-binding cassette sub-family B member 7, mitochondrial OS=Homo sapiens OX=9606 GN=ABCB7 PE=1 SV=2	0,684147788	1,642737
O43493	Trans-Golgi network integral membrane protein 2 OS=Homo sapiens OX=9606 GN=TGOLN2 PE=1 SV=3	0,236519395	1,641813
Q9BT78	COP9 signalosome complex subunit 4 OS=Homo sapiens OX=9606 GN=COPS4 PE=1 SV=1	0,14664182	1,6416
Q9UDY8	Mucosa-associated lymphoid tissue lymphoma translocation protein 1 OS=Homo sapiens OX=9606 GN=MALT1 PE=1 SV=1	0,316654113	1,63927
Q63HK5	Teashirt homolog 3 OS=Homo sapiens OX=9606 GN=TSHZ3 PE=1 SV=2	1,452053278	1,63829
P07355	Annexin A2 OS=Homo sapiens OX=9606 GN=ANXA2 PE=1 SV=2	-0,115254141	1,637802
O94832	Unconventional myosin-Id OS=Homo sapiens OX=9606 GN=MYO1D PE=1 SV=2	-0,608727483	1,636428
Q9H7C9	Mth938 domain-containing protein OS=Homo sapiens OX=9606 GN=AAMDC PE=1 SV=1	-0,370419292	1,636402
P63096	Guanine nucleotide-binding protein G(i) subunit alpha-1 OS=Homo sapiens OX=9606 GN=GNAI1 PE=1 SV=2	0,798997917	1,635161
Q9UQ80	Proliferation-associated protein 2G4 OS=Homo sapiens OX=9606 GN=PA2G4 PE=1 SV=3	0,135303655	1,634962
P35249	Replication factor C subunit 4 OS=Homo sapiens OX=9606 GN=RFC4 PE=1 SV=2	0,771607431	1,634752
Q9NRR5	Ubiquilin-4 OS=Homo sapiens OX=9606 GN=UBQLN4 PE=1 SV=2	0,79868481	1,634687
P04075	Fructose-bisphosphate aldolase A OS=Homo sapiens OX=9606 GN=ALDOA PE=1 SV=2	0,115000055	1,633097
Q15796	Mothers against decapentaplegic homolog 2 OS=Homo sapiens OX=9606 GN=SMAD2 PE=1 SV=1	0,797528263	1,632936

Q93008	Probable ubiquitin carboxyl-terminal hydrolase FAF-X OS=Homo sapiens OX=9606 GN=USP9X PE=1 SV=3	-0,099103176	1,632047
Q07955	Serine/arginine-rich splicing factor 1 OS=Homo sapiens OX=9606 GN=SRSF1 PE=1 SV=2	0,196274441	1,631985
P20962	Parathymosin OS=Homo sapiens OX=9606 GN=PTMS PE=1 SV=2	-0,470358546	1,631919
P17655	Calpain-2 catalytic subunit OS=Homo sapiens OX=9606 GN=CAPN2 PE=1 SV=6	0,116958481	1,631642
P14209	CD99 antigen OS=Homo sapiens OX=9606 GN=CD99 PE=1 SV=1	0,36937758	1,631303
O14684	Prostaglandin E synthase OS=Homo sapiens OX=9606 GN=PTGES PE=1 SV=2	0,280061599	1,630977
O14979	Heterogeneous nuclear ribonucleoprotein D-like OS=Homo sapiens OX=9606 GN=HNRNPDL PE=1 SV=3	-0,21964564	1,630239
O43264	Centromere/kinetochore protein zw10 homolog OS=Homo sapiens OX=9606 GN=ZW10 PE=1 SV=3	0,219595482	1,629776
P10619	Lysosomal protective protein OS=Homo sapiens OX=9606 GN=CTSA PE=1 SV=2	-0,219341505	1,627431
P61160	Actin-related protein 2 OS=Homo sapiens OX=9606 GN=ACTR2 PE=1 SV=1	0,154281288	1,624022
P04183	Thymidine kinase, cytosolic OS=Homo sapiens OX=9606 GN=TK1 PE=1 SV=2	0,467919539	1,623414
P19022	Cadherin-2 OS=Homo sapiens OX=9606 GN=CDH2 PE=1 SV=4	-0,195283017	1,621603
Q9BQE4	Selenoprotein S OS=Homo sapiens OX=9606 GN=SELENOS PE=1 SV=3	-0,467393539	1,621579
P30504	HLA class I histocompatibility antigen, Cw-4 alpha chain OS=Homo sapiens OX=9606 GN=HLA-C PE=1 SV=1	0,789333	1,620475
P24928	DNA-directed RNA polymerase II subunit RPB1 OS=Homo sapiens OX=9606 GN=POLR2A PE=1 SV=2	-0,467004779	1,620222
Q92896	Golgi apparatus protein 1 OS=Homo sapiens OX=9606 GN=GLG1 PE=1 SV=2	0,105539346	1,619872
P30405	Peptidyl-prolyl cis-trans isomerase F, mitochondrial OS=Homo sapiens OX=9606 GN=PPIF PE=1 SV=1	-0,277941599	1,616235
Q9H2U1	ATP-dependent DNA/RNA helicase DHX36 OS=Homo sapiens OX=9606 GN=DHX36 PE=1 SV=2	0,31264149	1,615149
Q96EE3	Nucleoporin SEH1 OS=Homo sapiens OX=9606 GN=SEH1L PE=1 SV=3	-0,277725884	1,614736
P19838	Nuclear factor NF-kappa-B p105 subunit OS=Homo sapiens OX=9606 GN=NFKB1 PE=1 SV=2	0,252524672	1,614119
P00533	Epidermal growth factor receptor OS=Homo sapiens OX=9606 GN=EGFR PE=1 SV=2	0,204974132	1,611553
P0DP25	Calmodulin-3 OS=Homo sapiens OX=9606 GN=CALM3 PE=1 SV=1	-0,177230903	1,611524
O75306	NADH dehydrogenase [ubiquinone] iron-sulfur protein 2, mitochondrial OS=Homo sapiens OX=9606 GN=NDUFS2 PE=1 SV=2	-0,311626551	1,609053
Q9BW72	HIG1 domain family member 2A, mitochondrial OS=Homo sapiens OX=9606 GN=HIGD2A PE=1 SV=1	-0,463623902	1,60841
Q13547	Histone deacetylase 1 OS=Homo sapiens OX=9606 GN=HDAC1 PE=1 SV=1	0,276712103	1,607696
P26641	Elongation factor 1-gamma OS=Homo sapiens OX=9606 GN=EEF1G PE=1 SV=3	0,122345018	1,606973
Q8N1F8	Serine/threonine-protein kinase 11-interacting protein OS=Homo sapiens OX=9606 GN=STK11IP PE=1 SV=4	0,136279173	1,602801
Q8IV38	Ankyrin repeat and MYND domain-containing protein 2 OS=Homo sapiens OX=9606 GN=ANKMY2 PE=1 SV=1	-0,530145529	1,602487
Q15813	Tubulin-specific chaperone E OS=Homo sapiens OX=9606 GN=TBCE PE=1 SV=1	-0,250963426	1,601989
Q9H9J2	39S ribosomal protein L44, mitochondrial OS=Homo sapiens OX=9606 GN=MRPL44 PE=1 SV=1	-0,36335829	1,601834
P02795	Metallothionein-2 OS=Homo sapiens OX=9606 GN=MT2A PE=1 SV=1	0,461595419	1,601314
Q15738	Sterol-4-alpha-carboxylate 3-dehydrogenase, decarboxylating OS=Homo sapiens OX=9606 GN=NSDHL PE=1 SV=2	0,193212358	1,59998
P14174	Macrophage migration inhibitory factor OS=Homo sapiens OX=9606 GN=MIF PE=1 SV=4	-0,775805091	1,59971
Q5QJ74	Tubulin-specific chaperone cofactor E-like protein OS=Homo sapiens OX=9606 GN=TBCEL PE=1 SV=2	1,151095546	1,599404
P50502	Hsc70-interacting protein OS=Homo sapiens OX=9606 GN=ST13 PE=1 SV=2	0,193023602	1,598013
Q8IVD9	NudC domain-containing protein 3 OS=Homo sapiens OX=9606 GN=NUDCD3 PE=1 SV=3	0,250438879	1,597917
Q14767	Latent-transforming growth factor beta-binding protein 2 OS=Homo sapiens OX=9606 GN=LTBP2 PE=1 SV=3	0,124102851	1,594165
Q9BXJ9	N-alpha-acetyltransferase 15, NatA auxiliary subunit OS=Homo sapiens OX=9606 GN=NAA15 PE=1 SV=1	0,147502238	1,594049
Q6VEQ5	WAS protein family homolog 2 OS=Homo sapiens OX=9606 GN=WASH2P PE=2 SV=2	0,361669912	1,593567
P42704	Leucine-rich PPR motif-containing protein, mitochondrial OS=Homo sapiens OX=9606 GN=LRPPRC PE=1 SV=3	-0,094890414	1,593204
Q96A65	Exocyst complex component 4 OS=Homo sapiens OX=9606 GN=EXOC4 PE=1 SV=1	-0,20308065	1,59294
Q6IBS0	Twinfilin-2 OS=Homo sapiens OX=9606 GN=TWF2 PE=1 SV=2	-0,20305289	1,592668
Q15648	Mediator of RNA polymerase II transcription subunit 1 OS=Homo sapiens OX=9606 GN=MED1 PE=1 SV=4	-0,771193239	1,592575
Q9Y3D6	Mitochondrial fission 1 protein OS=Homo sapiens OX=9606 GN=FIS1 PE=1 SV=2	0,249603482	1,591438
Q16774	Guanylate kinase OS=Homo sapiens OX=9606 GN=GUK1 PE=1 SV=2	-0,274111203	1,589655
Q9Y5H0	Protocadherin gamma-A3 OS=Homo sapiens OX=9606 GN=PCDHGA3 PE=2 SV=2	1,124404459	1,58901
P04424	Argininosuccinate lyase OS=Homo sapiens OX=9606 GN=ASL PE=1 SV=4	0,215142334	1,588791
O95394	Phosphoacetylglucosamine mutase OS=Homo sapiens OX=9606 GN=PGM3 PE=1 SV=1	0,135582739	1,58833
P01130	Low-density lipoprotein receptor OS=Homo sapiens OX=9606 GN=LDLR PE=1 SV=1	-0,457841458	1,588162
Q13425	Beta-2-syntrophin OS=Homo sapiens OX=9606 GN=SNTB2 PE=1 SV=1	0,214712818	1,584852
Q9H270	Vacuolar protein sorting-associated protein 11 homolog OS=Homo sapiens OX=9606 GN=VPS11 PE=1 SV=1	0,307482906	1,584191
Q9Y5V3	Melanoma-associated antigen D1 OS=Homo sapiens OX=9606 GN=MAGED1 PE=1 SV=3	0,273227305	1,583531
Q96AG4	Leucine-rich repeat-containing protein 59 OS=Homo sapiens OX=9606 GN=LRRC59 PE=1 SV=1	0,167857993	1,583012
Q9BU61	NADH dehydrogenase [ubiquinone] 1 alpha subcomplex assembly factor 3 OS=Homo sapiens OX=9606 GN=NDUFAF3 PE=1 SV=1	0,818407316	1,582004
P24666	Low molecular weight phosphotyrosine protein phosphatase OS=Homo sapiens OX=9606 GN=ACP1 PE=1 SV=3	-0,272898859	1,581256
Q13488	V-type proton ATPase 116 kDa subunit a isoform 3 OS=Homo sapiens OX=9606 GN=TCIRG1 PE=1 SV=3	0,24786659	1,577982
P43235	Cathepsin K OS=Homo sapiens OX=9606 GN=CTSK PE=1 SV=1	-0,737743783	1,577517
P15559	NAD(P)H dehydrogenase [quinone] 1 OS=Homo sapiens OX=9606 GN=NQO1 PE=1 SV=1	-0,16730685	1,576405
Q13438	Protein OS-9 OS=Homo sapiens OX=9606 GN=OS9 PE=1 SV=1	0,454065679	1,57491
P21333	Filamin-A OS=Homo sapiens OX=9606 GN=FLNA PE=1 SV=4	-0,051450481	1,574567
O00571	ATP-dependent RNA helicase DDX3X OS=Homo sapiens OX=9606 GN=DDX3X PE=1 SV=3	0,106391769	1,573533
Q92797	Symplekin OS=Homo sapiens OX=9606 GN=SYMPK PE=1 SV=2	0,758853552	1,573341
Q14498	RNA-binding protein 39 OS=Homo sapiens OX=9606 GN=RBM39 PE=1 SV=2	0,16694802	1,572107
O60879	Protein diaphanous homolog 2 OS=Homo sapiens OX=9606 GN=DIAPH2 PE=1 SV=1	-0,757970878	1,571957
Q96TA2	ATP-dependent zinc metalloprotease YME1L1 OS=Homo sapiens OX=9606 GN=YME1L1 PE=1 SV=2	-0,453029467	1,571269
P83731	60S ribosomal protein L24 OS=Homo sapiens OX=9606 GN=RPL24 PE=1 SV=1	0,304971938	1,569144
Q15018	BRISC complex subunit Abraxas 2 OS=Homo sapiens OX=9606 GN=ABRAXAS2 PE=1 SV=2	-0,756177316	1,569141
P01040	Cystatin-A OS=Homo sapiens OX=9606 GN=CSTA PE=1 SV=1	-0,452044784	1,567807
Q96P70	Importin-9 OS=Homo sapiens OX=9606 GN=IPO9 PE=1 SV=3	-0,18117268	1,567556
P49790	Nuclear pore complex protein Nup153 OS=Homo sapiens OX=9606 GN=NUP153 PE=1 SV=2	0,356217646	1,566871
Q92522	Histone H1x OS=Homo sapiens OX=9606 GN=H1FX PE=1 SV=1	-0,451600933	1,566246
O60936	Nucleolar protein 3 OS=Homo sapiens OX=9606 GN=NOL3 PE=1 SV=2	-0,356058781	1,566093
Q9BYX7	Putative beta-actin-like protein 3 OS=Homo sapiens OX=9606 GN=POTEKP PE=5 SV=1	-0,355825632	1,564951
Q13098	COP9 signalosome complex subunit 1 OS=Homo sapiens OX=9606 GN=GPS1 PE=1 SV=4	0,180930406	1,56491
Q13573	SNW domain-containing protein 1 OS=Homo sapiens OX=9606 GN=SNW1 PE=1 SV=1	0,355744024	1,564552
Q9BTM9	Ubiquitin-related modifier 1 OS=Homo sapiens OX=9606 GN=URM1 PE=1 SV=1	-0,752421715	1,563232
O60832	H/ACA ribonucleoprotein complex subunit DKC1 OS=Homo sapiens OX=9606 GN=DKC1 PE=1 SV=3	0,212177224	1,561648
Q96B36	Proline-rich AKT1 substrate 1 OS=Homo sapiens OX=9606 GN=AKT1S1 PE=1 SV=1	-0,450221549	1,561393
P47712	Cytosolic phospholipase A2 OS=Homo sapiens OX=9606 GN=PLA2G4A PE=1 SV=2	0,245700567	1,561235
P51532	Transcription activator BRG1 OS=Homo sapiens OX=9606 GN=SMARCA4 PE=1 SV=2	0,450143579	1,561118
P30050	60S ribosomal protein L12 OS=Homo sapiens OX=9606 GN=RPL12 PE=1 SV=1	0,245674615	1,561035
Q9H6U6	Breast carcinoma-amplified sequence 3 OS=Homo sapiens OX=9606 GN=BCAS3 PE=1 SV=3	0,606951515	1,560747
O00233	26S proteasome non-ATPase regulatory subunit 9 OS=Homo sapiens OX=9606 GN=PSMD9 PE=1 SV=3	-0,269908174	1,560571
Q9H425	Uncharacterized protein C1orf198 OS=Homo sapiens OX=9606 GN=C1orf198 PE=1 SV=1	-0,18052176	1,560451
P07195	L-lactate dehydrogenase B chain OS=Homo sapiens OX=9606 GN=LDHB PE=1 SV=2	0,137049976	1,5581
O75335	Liprin-alpha-4 OS=Homo sapiens OX=9606 GN=PPFIA4 PE=2 SV=3	-0,130384942	1,557072
Q14938	Nuclear factor 1 X-type OS=Homo sapiens OX=9606 GN=NFIX PE=1 SV=2	0,825382531	1,556641
Q8IWE2	Protein NOXP20 OS=Homo sapiens OX=9606 GN=FAM114A1 PE=1 SV=2	0,144791635	1,556572
E9PAV3	Nascent polypeptide-associated complex subunit alpha, muscle-specific form OS=Homo sapiens OX=9606 GN=NACA PE=1 SV=1	-0,268693892	1,552185
P55263	Adenosine kinase OS=Homo sapiens OX=9606 GN=ADK PE=1 SV=2	-0,172054224	1,552074
P82979	SAP domain-containing ribonucleoprotein OS=Homo sapiens OX=9606 GN=SARNP PE=1 SV=3	-0,302062054	1,551725
Q9UBQ5	Eukaryotic translation initiation factor 3 subunit K OS=Homo sapiens OX=9606 GN=EIF3K PE=1 SV=1	0,225910185	1,551615
P10644	cAMP-dependent protein kinase type I-alpha regulatory subunit OS=Homo sapiens OX=9606 GN=PRKAR1A PE=1 SV=1	0,153774574	1,551507
Q8N684	Cleavage and polyadenylation specificity factor subunit 7 OS=Homo sapiens OX=9606 GN=CPSF7 PE=1 SV=1	-0,352990543	1,55107
Q9UL46	Proteasome activator complex subunit 2 OS=Homo sapiens OX=9606 GN=PSME2 PE=1 SV=4	0,153643615	1,549822
Q6P1N0	Coiled-coil and C2 domain-containing protein 1A OS=Homo sapiens OX=9606 GN=CC2D1A PE=1 SV=1	-0,30141562	1,547858
P60510	Serine/threonine-protein phosphatase 4 catalytic subunit OS=Homo sapiens OX=9606 GN=PPP4C PE=1 SV=1	0,301233729	1,54677
Q16629	Serine/arginine-rich splicing factor 7 OS=Homo sapiens OX=9606 GN=SRSF7 PE=1 SV=1	0,225268981	1,546201

P07203	Glutathione peroxidase 1 OS=Homo sapiens OX=9606 GN=GPX1 PE=1 SV=4	-0,210291069	1,544443
O75828	Carbonyl reductase [NADPH] 3 OS=Homo sapiens OX=9606 GN=CBR3 PE=1 SV=3	0,179039808	1,54431
O15484	Calpain-5 OS=Homo sapiens OX=9606 GN=CAPN5 PE=1 SV=2	-0,445088631	1,543306
Q9Y5B9	FACT complex subunit SPT16 OS=Homo sapiens OX=9606 GN=SUPT16H PE=1 SV=1	0,171284268	1,543289
Q13185	Chromobox protein homolog 3 OS=Homo sapiens OX=9606 GN=CBX3 PE=1 SV=4	-0,243327215	1,542927
Q9UBT2	SUMO-activating enzyme subunit 2 OS=Homo sapiens OX=9606 GN=UBA2 PE=1 SV=2	0,187646509	1,542262
Q4ZHG4	Fibronectin type III domain-containing protein 1 OS=Homo sapiens OX=9606 GN=FNDC1 PE=2 SV=4	-0,351176303	1,542187
P17096	High mobility group protein HMG-I/HMG-Y OS=Homo sapiens OX=9606 GN=HMGA1 PE=1 SV=3	0,300412594	1,54186
O15305	Phosphomannomutase 2 OS=Homo sapiens OX=9606 GN=PMM2 PE=1 SV=1	-0,18749636	1,540714
P52435	DNA-directed RNA polymerase II subunit RPB11-a OS=Homo sapiens OX=9606 GN=POLR2J PE=1 SV=1	-0,737817247	1,540065
P04066	Tissue alpha-L-fucosidase OS=Homo sapiens OX=9606 GN=FUCA1 PE=1 SV=4	-0,670978328	1,539372
Q8TAE8	Growth arrest and DNA damage-inducible proteins-interacting protein 1 OS=Homo sapiens OX=9606 GN=GADD45GIP1 PE=1 SV=1	-0,737098952	1,538917
P15880	40S ribosomal protein S2 OS=Homo sapiens OX=9606 GN=RPS2 PE=1 SV=2	-0,158072333	1,53766
Q00526	Cyclin-dependent kinase 3 OS=Homo sapiens OX=9606 GN=CDK3 PE=1 SV=1	-0,823010919	1,537208
O76071	Probable cytosolic iron-sulfur protein assembly protein CIAO1 OS=Homo sapiens OX=9606 GN=CIAO1 PE=1 SV=1	0,442780002	1,535156
Q9NX63	MICOS complex subunit MIC19 OS=Homo sapiens OX=9606 GN=CHCHD3 PE=1 SV=1	0,266160672	1,534714
P21589	5'-nucleotidase OS=Homo sapiens OX=9606 GN=NT5E PE=1 SV=1	-0,128804086	1,534329
Q9Y4B6	DDB1- and CUL4-associated factor 1 OS=Homo sapiens OX=9606 GN=DCAF1 PE=1 SV=3	-0,217675085	1,532667
Q7KYR7	Butyrophilin subfamily 2 member A1 OS=Homo sapiens OX=9606 GN=BTN2A1 PE=1 SV=3	0,733140456	1,532582
Q9UJ68	Mitochondrial peptide methionine sulfoxide reductase OS=Homo sapiens OX=9606 GN=MSRA PE=1 SV=1	-0,441143662	1,529375
P12956	X-ray repair cross-complementing protein 6 OS=Homo sapiens OX=9606 GN=XRCC6 PE=1 SV=2	0,115402775	1,528294
P50148	Guanine nucleotide-binding protein G(q) subunit alpha OS=Homo sapiens OX=9606 GN=GNAQ PE=1 SV=4	0,177463802	1,527199
O95163	Elongator complex protein 1 OS=Homo sapiens OX=9606 GN=ELP1 PE=1 SV=3	0,163162907	1,526994
Q06830	Peroxiredoxin-1 OS=Homo sapiens OX=9606 GN=PRDX1 PE=1 SV=1	0,157061184	1,525165
Q86UU0	B-cell CLL/lymphoma 9-like protein OS=Homo sapiens OX=9606 GN=BCL9L PE=1 SV=1	0,698109617	1,523882
Q8IY31	Intraflagellar transport protein 20 homolog OS=Homo sapiens OX=9606 GN=IFT20 PE=1 SV=1	-0,439229372	1,522606
Q00577	Transcriptional activator protein Pur-alpha OS=Homo sapiens OX=9606 GN=PURA PE=1 SV=2	-0,264361598	1,522327
P51610	Host cell factor 1 OS=Homo sapiens OX=9606 GN=HCFC1 PE=1 SV=2	-0,207809013	1,521876
P37837	Transaldolase OS=Homo sapiens OX=9606 GN=TALDO1 PE=1 SV=2	-0,138250016	1,521864
P53992	Protein transport protein Sec24C OS=Homo sapiens OX=9606 GN=SEC24C PE=1 SV=3	0,127868957	1,520028
Q92979	Ribosomal RNA small subunit methyltransferase NEP1 OS=Homo sapiens OX=9606 GN=EMG1 PE=1 SV=4	-0,346379786	1,518704
Q9BSJ8	Extended synaptotagmin-1 OS=Homo sapiens OX=9606 GN=ESYT1 PE=1 SV=1	0,10191159	1,518127
Q99848	Probable rRNA-processing protein EBP2 OS=Homo sapiens OX=9606 GN=EBNA1BP2 PE=1 SV=2	0,72399639	1,517862
Q6PCE3	Glucose 1,6-bisphosphate synthase OS=Homo sapiens OX=9606 GN=PGM2L1 PE=1 SV=3	0,296189922	1,516638
Q04760	Lactoylglutathione lyase OS=Homo sapiens OX=9606 GN=GLO1 PE=1 SV=4	-0,1851548	1,516617
Q9Y570	Protein phosphatase methylesterase 1 OS=Homo sapiens OX=9606 GN=PPME1 PE=1 SV=3	0,16879003	1,514933
P42677	40S ribosomal protein S27 OS=Homo sapiens OX=9606 GN=RPS27 PE=1 SV=3	0,292625823	1,513646
P31749	RAC-alpha serine/threonine-protein kinase OS=Homo sapiens OX=9606 GN=AKT1 PE=1 SV=2	-0,345049262	1,512191
P50991	T-complex protein 1 subunit delta OS=Homo sapiens OX=9606 GN=CCT4 PE=1 SV=4	0,101540395	1,510976
P42224	Signal transducer and activator of transcription 1-alpha/beta OS=Homo sapiens OX=9606 GN=STAT1 PE=1 SV=2	-0,104653014	1,508935
Q9HAN9	Nicotinamide/nicotinic acid mononucleotide adenylyltransferase 1 OS=Homo sapiens OX=9606 GN=NMNAT1 PE=1 SV=1	0,718131812	1,508358
Q15691	Microtubule-associated protein RP/EB family member 1 OS=Homo sapiens OX=9606 GN=MAPRE1 PE=1 SV=3	0,155693463	1,508314
Q96RS6	NudC domain-containing protein 1 OS=Homo sapiens OX=9606 GN=NUDCD1 PE=1 SV=2	0,238665694	1,507098
P54652	Heat shock-related 70 kDa protein 2 OS=Homo sapiens OX=9606 GN=HSPA2 PE=1 SV=1	0,150205891	1,505781
Q13151	Heterogeneous nuclear ribonucleoprotein A0 OS=Homo sapiens OX=9606 GN=HNRNPA0 PE=1 SV=1	-0,261729019	1,504231
Q9GZL7	Ribosome biogenesis protein WDR12 OS=Homo sapiens OX=9606 GN=WDR12 PE=1 SV=2	0,293798863	1,502377
Q5CZC0	Fibrous sheath-interacting protein 2 OS=Homo sapiens OX=9606 GN=FSIP2 PE=2 SV=4	-0,714382865	1,502256
Q9H0R4	Haloacid dehalogenase-like hydrolase domain-containing protein 2 OS=Homo sapiens OX=9606 GN=HDHD2 PE=1 SV=1	-0,433125002	1,500983
O95396	Adenylyltransferase and sulfurtransferase MOCS3 OS=Homo sapiens OX=9606 GN=MOCS3 PE=1 SV=1	0,532507168	1,500924
P51798	H(+)/Cl(-) exchange transporter 7 OS=Homo sapiens OX=9606 GN=CLCN7 PE=1 SV=2	0,712709909	1,499527
Q9UGI8	Testin OS=Homo sapiens OX=9606 GN=TES PE=1 SV=1	0,154951016	1,499191
Q6NXS1	#N/D	0,34238606	1,499156
Q7L523	Ras-related GTP-binding protein A OS=Homo sapiens OX=9606 GN=RRAGA PE=1 SV=1	-0,711409994	1,497403
P23634	Plasma membrane calcium-transporting ATPase 4 OS=Homo sapiens OX=9606 GN=ATP2B4 PE=1 SV=2	0,123459191	1,497101
Q13642	Four and a half LIM domains protein 1 OS=Homo sapiens OX=9606 GN=FHL1 PE=1 SV=4	-0,192832466	1,493216
Q8IWB7	WD repeat and FYVE domain-containing protein 1 OS=Homo sapiens OX=9606 GN=WDFY1 PE=1 SV=1	0,259966348	1,492136
Q32P28	Prolyl 3-hydroxylase 1 OS=Homo sapiens OX=9606 GN=P3H1 PE=1 SV=2	0,144392846	1,492113
Q6PIU2	Neutral cholesterol ester hydrolase 1 OS=Homo sapiens OX=9606 GN=NCEH1 PE=1 SV=3	0,174159055	1,491498
P33993	DNA replication licensing factor MCM7 OS=Homo sapiens OX=9606 GN=MCM7 PE=1 SV=4	0,236434257	1,490009
P40763	Signal transducer and activator of transcription 3 OS=Homo sapiens OX=9606 GN=STAT3 PE=1 SV=2	-0,13593914	1,489418
Q9Y508	E3 ubiquitin-protein ligase RNF114 OS=Homo sapiens OX=9606 GN=RNF114 PE=1 SV=1	0,34033573	1,489122
Q08379	Golgin subfamily A member 2 OS=Homo sapiens OX=9606 GN=GOLGA2 PE=1 SV=3	0,159897545	1,488399
Q9Y5M8	Signal recognition particle receptor subunit beta OS=Homo sapiens OX=9606 GN=SRPRB PE=1 SV=3	0,166251923	1,486239
P49427	Ubiquitin-conjugating enzyme E2 R1 OS=Homo sapiens OX=9606 GN=CDC34 PE=1 SV=2	-0,428422138	1,484285
P16403	Histone H1.2 OS=Homo sapiens OX=9606 GN=HIST1H1C PE=1 SV=2	0,290389934	1,482072
Q14558	Phosphoribosyl pyrophosphate synthase-associated protein 1 OS=Homo sapiens OX=9606 GN=PRPSAP1 PE=1 SV=2	0,235231406	1,480814
Q8WZA0	Protein LZIC OS=Homo sapiens OX=9606 GN=LZIC PE=1 SV=1	-0,29010932	1,480402
P51570	Galactokinase OS=Homo sapiens OX=9606 GN=GALK1 PE=1 SV=1	0,181456918	1,47878
Q9C0E8	Endoplasmic reticulum junction formation protein lunapark OS=Homo sapiens OX=9606 GN=LNPK PE=1 SV=2	0,289768298	1,478373
P82930	28S ribosomal protein S34, mitochondrial OS=Homo sapiens OX=9606 GN=MRPS34 PE=1 SV=2	0,569592367	1,478372
P0DI81	Trafficking protein particle complex subunit 2 OS=Homo sapiens OX=9606 GN=TRAPPC2 PE=1 SV=1	-0,698782383	1,476645
Q8WWY3	U4/U6 small nuclear ribonucleoprotein Prp31 OS=Homo sapiens OX=9606 GN=PRPF31 PE=1 SV=2	0,426205864	1,476404
P08572	Collagen alpha-2(IV) chain OS=Homo sapiens OX=9606 GN=COL4A2 PE=1 SV=4	0,426010918	1,47571
P0CAP2	DNA-directed RNA polymerase II subunit GRINL1A OS=Homo sapiens OX=9606 GN=POLR2M PE=1 SV=1	0,441477835	1,475194
P11413	Glucose-6-phosphate 1-dehydrogenase OS=Homo sapiens OX=9606 GN=G6PD PE=1 SV=4	-0,106299772	1,474875
P36021	Monocarboxylate transporter 8 OS=Homo sapiens OX=9606 GN=SLC16A2 PE=1 SV=2	0,946326473	1,474496
Q969U7	Proteasome assembly chaperone 2 OS=Homo sapiens OX=9606 GN=PSMG2 PE=1 SV=1	-0,425589013	1,474209
P28300	Protein-lysine 6-oxidase OS=Homo sapiens OX=9606 GN=LOX PE=1 SV=2	0,165137517	1,473692
Q4KMQ2	Anoctamin-6 OS=Homo sapiens OX=9606 GN=ANO6 PE=1 SV=2	0,696719559	1,473232
Q9NPA0	ER membrane protein complex subunit 7 OS=Homo sapiens OX=9606 GN=EMC7 PE=1 SV=1	0,336800582	1,471824
Q07617	Sperm-associated antigen 1 OS=Homo sapiens OX=9606 GN=SPAG1 PE=1 SV=3	3,290563932	1,471546
P25686	DnaJ homolog subfamily B member 2 OS=Homo sapiens OX=9606 GN=DNAJB2 PE=1 SV=3	0,424617668	1,470752
P26447	Protein S100-A4 OS=Homo sapiens OX=9606 GN=S100A4 PE=1 SV=1	0,424432174	1,470091
P06702	Protein S100-A9 OS=Homo sapiens OX=9606 GN=S100A9 PE=1 SV=1	-0,69415532	1,46898
Q01518	Adenylyl cyclase-associated protein 1 OS=Homo sapiens OX=9606 GN=CAP1 PE=1 SV=5	-0,10418917	1,468955
P54709	Sodium/potassium-transporting ATPase subunit beta-3 OS=Homo sapiens OX=9606 GN=ATP1B3 PE=1 SV=1	0,215945268	1,467986
P31939	Bifunctional purine biosynthesis protein PURH OS=Homo sapiens OX=9606 GN=ATIC PE=1 SV=3	-0,096313085	1,465878
Q96SW2	Protein cereblon OS=Homo sapiens OX=9606 GN=CRBN PE=1 SV=1	0,053560056	1,465524
Q9NPJ3	Acyl-coenzyme A thioesterase 13 OS=Homo sapiens OX=9606 GN=ACOT13 PE=1 SV=1	-0,282047314	1,464958
P49069	Calcium signal-modulating cyclophilin ligand OS=Homo sapiens OX=9606 GN=CAMLG PE=1 SV=1	0,325512403	1,463986
Q8NFP7	#N/D	-0,690957446	1,463663
Q06323	Proteasome activator complex subunit 1 OS=Homo sapiens OX=9606 GN=PSME1 PE=1 SV=1	0,157777229	1,463499
P30040	Endoplasmic reticulum resident protein 29 OS=Homo sapiens OX=9606 GN=ERP29 PE=1 SV=4	-0,189719614	1,46327
O95147	Dual specificity protein phosphatase 14 OS=Homo sapiens OX=9606 GN=DUSP14 PE=1 SV=1	0,690392815	1,462723
P31483	Nucleolysin TIA-1 isoform p40 OS=Homo sapiens OX=9606 GN=TIA1 PE=1 SV=3	-0,308604557	1,462345
P10515	Dihydrolipoyllysine-residue acetyltransferase component of pyruvate dehydrogenase complex, mitochondrial OS=Homo sapiens OX=96(	0,189604809	1,462169
P63010	AP-2 complex subunit beta OS=Homo sapiens OX=9606 GN=AP2B1 PE=1 SV=1	0,092235153	1,461728
Q12979	Active breakpoint cluster region-related protein OS=Homo sapiens OX=9606 GN=ABR PE=1 SV=2	-0,215100465	1,460947

Q01130	Serine/arginine-rich splicing factor 2 OS=Homo sapiens OX=9606 GN=SRSF2 PE=1 SV=4	0,23249949	1,459975
Q9H9G7	Protein argonaute-3 OS=Homo sapiens OX=9606 GN=AGO3 PE=1 SV=2	-1,169947267	1,45888
Q13586	Stromal interaction molecule 1 OS=Homo sapiens OX=9606 GN=STIM1 PE=1 SV=3	-0,189079127	1,457129
Q9BUH6	Protein PAXX OS=Homo sapiens OX=9606 GN=PAXX PE=1 SV=2	0,685826308	1,455101
Q02224	Centromere-associated protein E OS=Homo sapiens OX=9606 GN=CENPE PE=1 SV=2	1,883837453	1,454233
O95834	Echinoderm microtubule-associated protein-like 2 OS=Homo sapiens OX=9606 GN=EML2 PE=1 SV=1	-0,419896447	1,453928
O43739	Cytohesin-3 OS=Homo sapiens OX=9606 GN=CYTH3 PE=1 SV=2	-0,649950991	1,453743
Q9Y2T2	AP-3 complex subunit mu-1 OS=Homo sapiens OX=9606 GN=AP3M1 PE=1 SV=1	-0,200224641	1,453445
Q14571	Inositol 1,4,5-trisphosphate receptor type 2 OS=Homo sapiens OX=9606 GN=ITPR2 PE=1 SV=2	0,382926969	1,452575
Q9NQP4	Prefoldin subunit 4 OS=Homo sapiens OX=9606 GN=PFDN4 PE=1 SV=1	-0,683896614	1,45187
Q99442	Translocation protein SEC62 OS=Homo sapiens OX=9606 GN=SEC62 PE=1 SV=1	0,419163109	1,451311
Q09028	Histone-binding protein RBBP4 OS=Homo sapiens OX=9606 GN=RBBP4 PE=1 SV=3	0,231275528	1,450659
Q9H7Z7	Prostaglandin E synthase 2 OS=Homo sapiens OX=9606 GN=PTGES2 PE=1 SV=1	-0,285047762	1,450318
Q15257	Serine/threonine-protein phosphatase 2A activator OS=Homo sapiens OX=9606 GN=PTPA PE=1 SV=3	-0,231190133	1,45001
Q6IAA8	Ragulator complex protein LAMTOR1 OS=Homo sapiens OX=9606 GN=LAMTOR1 PE=1 SV=2	0,284871129	1,449269
Q9NYU1	UDP-glucose:glycoprotein glucosyltransferase 2 OS=Homo sapiens OX=9606 GN=UGGT2 PE=1 SV=4	-0,253546394	1,448227
O95470	Sphingosine-1-phosphate lyase 1 OS=Homo sapiens OX=9606 GN=SGPL1 PE=1 SV=3	0,199637602	1,448182
P12694	2-oxoisovalerate dehydrogenase subunit alpha, mitochondrial OS=Homo sapiens OX=9606 GN=BCKDHA PE=1 SV=2	0,680823162	1,446714
O15067	Phosphoribosylformylglycinamidine synthase OS=Homo sapiens OX=9606 GN=PFAS PE=1 SV=4	0,156040511	1,4432
P32856	Syntaxin-2 OS=Homo sapiens OX=9606 GN=STX2 PE=1 SV=3	0,518119002	1,44037
O75170	Serine/threonine-protein phosphatase 6 regulatory subunit 2 OS=Homo sapiens OX=9606 GN=PPP6R2 PE=1 SV=2	-0,676938905	1,440176
P01137	Transforming growth factor beta-1 proprotein OS=Homo sapiens OX=9606 GN=TGFB1 PE=1 SV=2	0,675927436	1,43847
O95816	BAG family molecular chaperone regulator 2 OS=Homo sapiens OX=9606 GN=BAG2 PE=1 SV=1	0,187093527	1,438134
Q9Y376	Calcium-binding protein 39 OS=Homo sapiens OX=9606 GN=CAB39 PE=1 SV=1	-0,229464467	1,436898
P49959	Double-strand break repair protein MRE11 OS=Homo sapiens OX=9606 GN=MRE11 PE=1 SV=3	-0,41451618	1,434716
Q9H4A3	Serine/threonine-protein kinase WNK1 OS=Homo sapiens OX=9606 GN=WNK1 PE=1 SV=2	-0,198096027	1,434384
P41221	Protein Wnt-5a OS=Homo sapiens OX=9606 GN=WNT5A PE=1 SV=2	-0,414286032	1,433893
P09132	Signal recognition particle 19 kDa protein OS=Homo sapiens OX=9606 GN=SRP19 PE=1 SV=3	-0,328923317	1,433299
O95861	3'(2'),5'-bisphosphate nucleotidase 1 OS=Homo sapiens OX=9606 GN=BPNT1 PE=1 SV=1	0,197958706	1,433157
O75477	Erlin-1 OS=Homo sapiens OX=9606 GN=ERLIN1 PE=1 SV=2	0,176910698	1,432633
Q01469	Fatty acid-binding protein 5 OS=Homo sapiens OX=9606 GN=FABP5 PE=1 SV=3	-0,211630784	1,432119
Q15024	Exosome complex component RRP42 OS=Homo sapiens OX=9606 GN=EXOSC7 PE=1 SV=3	0,41367271	1,4317
O95427	GPI ethanolamine phosphate transferase 1 OS=Homo sapiens OX=9606 GN=PIGN PE=1 SV=1	1,056381598	1,430825
Q14999	Cullin-7 OS=Homo sapiens OX=9606 GN=CUL7 PE=1 SV=2	0,619366819	1,429906
P11182	Lipoamide acyltransferase component of branched-chain alpha-keto acid dehydrogenase complex, mitochondrial OS=Homo sapiens OX	0,327425831	1,425979
P34931	Heat shock 70 kDa protein 1-like OS=Homo sapiens OX=9606 GN=HSPA1L PE=1 SV=2	-0,609231614	1,424797
Q5JSH3	WD repeat-containing protein 44 OS=Homo sapiens OX=9606 GN=WDR44 PE=1 SV=1	0,167787233	1,42336
Q9Y6A5	Transforming acidic coiled-coil-containing protein 3 OS=Homo sapiens OX=9606 GN=TACC3 PE=1 SV=1	0,411186484	1,422805
Q9H098	Protein FAM107B OS=Homo sapiens OX=9606 GN=FAM107B PE=1 SV=1	-0,664807891	1,419611
Q06136	3-ketodihydrosphingosine reductase OS=Homo sapiens OX=9606 GN=KDSR PE=1 SV=1	-0,325990609	1,418965
Q9UL25	Ras-related protein Rab-21 OS=Homo sapiens OX=9606 GN=RAB21 PE=1 SV=3	0,19634461	1,418749
P21127	Cyclin-dependent kinase 11B OS=Homo sapiens OX=9606 GN=CDK11B PE=1 SV=4	0,664226661	1,41862
Q8IV08	Phospholipase D3 OS=Homo sapiens OX=9606 GN=PLD3 PE=1 SV=1	-0,324994851	1,414099
P62826	GTP-binding nuclear protein Ran OS=Homo sapiens OX=9606 GN=RAN PE=1 SV=3	0,184520413	1,41362
Q9Y2H1	Serine/threonine-protein kinase 38-like OS=Homo sapiens OX=9606 GN=STK38L PE=1 SV=3	-0,324697055	1,412644
P54578	Ubiquitin carboxyl-terminal hydrolase 14 OS=Homo sapiens OX=9606 GN=USP14 PE=1 SV=3	0,117877617	1,411085
Q9UMS6	Synaptopodin-2 OS=Homo sapiens OX=9606 GN=SYNPO2 PE=1 SV=2	-0,184148181	1,410083
Q8NEU8	DCC-interacting protein 13-beta OS=Homo sapiens OX=9606 GN=APPL2 PE=1 SV=3	-0,16638577	1,408497
Q9UHG3	Prenylcysteine oxidase 1 OS=Homo sapiens OX=9606 GN=PCYOX1 PE=1 SV=3	0,153041319	1,408348
Q04721	Neurogenic locus notch homolog protein 2 OS=Homo sapiens OX=9606 GN=NOTCH2 PE=1 SV=3	-0,123426979	1,408305
Q969S3	Zinc finger protein 622 OS=Homo sapiens OX=9606 GN=ZNF622 PE=1 SV=1	0,657423627	1,406982
O75691	Small subunit processome component 20 homolog OS=Homo sapiens OX=9606 GN=UTP20 PE=1 SV=3	-0,58111637	1,405857
O15231	Zinc finger protein 185 OS=Homo sapiens OX=9606 GN=ZNF185 PE=1 SV=3	-0,317792157	1,404448
Q9NZ01	Very-long-chain enoyl-CoA reductase OS=Homo sapiens OX=9606 GN=TECR PE=1 SV=1	-0,194602076	1,403237
P24821	Tenascin OS=Homo sapiens OX=9606 GN=TNC PE=1 SV=3	-0,069358655	1,401472
O00186	Syntaxin-binding protein 3 OS=Homo sapiens OX=9606 GN=STXBP3 PE=1 SV=2	0,183230058	1,40137
P84243	Histone H3.3 OS=Homo sapiens OX=9606 GN=H3F3A PE=1 SV=2	0,404728335	1,399661
Q9UNF0	Protein kinase C and casein kinase substrate in neurons protein 2 OS=Homo sapiens OX=9606 GN=PACSIN2 PE=1 SV=2	0,141736733	1,399006
Q7Z6B7	SLIT-ROBO Rho GTPase-activating protein 1 OS=Homo sapiens OX=9606 GN=SRGAP1 PE=1 SV=1	0,403677167	1,395888
Q12981	Vesicle transport protein SEC20 OS=Homo sapiens OX=9606 GN=BNIP1 PE=1 SV=3	-0,650264833	1,394658
Q14061	Cytochrome c oxidase copper chaperone OS=Homo sapiens OX=9606 GN=COX17 PE=1 SV=2	0,402752938	1,39257
Q9H6S3	Epidermal growth factor receptor kinase substrate 8-like protein 2 OS=Homo sapiens OX=9606 GN=EPS8L2 PE=1 SV=2	-0,223514505	1,391886
Q9BQS8	FYVE and coiled-coil domain-containing protein 1 OS=Homo sapiens OX=9606 GN=FYCO1 PE=1 SV=3	0,320320353	1,391266
Q9H4A4	Aminopeptidase B OS=Homo sapiens OX=9606 GN=RNPEP PE=1 SV=2	-0,125405433	1,391084
Q9Y450	HBS1-like protein OS=Homo sapiens OX=9606 GN=HBS1L PE=1 SV=1	0,164624484	1,389883
Q9BVG9	Phosphatidylserine synthase 2 OS=Homo sapiens OX=9606 GN=PTDSS2 PE=1 SV=1	0,682072499	1,38873
Q9NZL9	Methionine adenosyltransferase 2 subunit beta OS=Homo sapiens OX=9606 GN=MAT2B PE=1 SV=1	0,192948581	1,388558
Q02750	Dual specificity mitogen-activated protein kinase kinase 1 OS=Homo sapiens OX=9606 GN=MAP2K1 PE=1 SV=2	0,206341305	1,388436
Q14667	Protein KIAA0100 OS=Homo sapiens OX=9606 GN=KIAA0100 PE=1 SV=3	0,646469653	1,388092
Q9H488	GDP-fucose protein O-fucosyltransferase 1 OS=Homo sapiens OX=9606 GN=POFUT1 PE=1 SV=1	0,222892612	1,387199
Q13287	N-myc-interactor OS=Homo sapiens OX=9606 GN=NMI PE=1 SV=2	-0,770143438	1,386617
Q9Y282	Endoplasmic reticulum-Golgi intermediate compartment protein 3 OS=Homo sapiens OX=9606 GN=ERGIC3 PE=1 SV=1	0,244460162	1,386485
Q5VT25	Serine/threonine-protein kinase MRCK alpha OS=Homo sapiens OX=9606 GN=CDC42BPA PE=1 SV=1	-0,244405522	1,386116
Q15075	Early endosome antigen 1 OS=Homo sapiens OX=9606 GN=EEA1 PE=1 SV=2	0,085177154	1,38542
Q9Y4P3	Transducin beta-like protein 2 OS=Homo sapiens OX=9606 GN=TBL2 PE=1 SV=1	0,150856716	1,383124
O00139	Kinesin-like protein KIF2A OS=Homo sapiens OX=9606 GN=KIF2A PE=1 SV=3	-0,205622213	1,382523
P50995	Annexin A11 OS=Homo sapiens OX=9606 GN=ANXA11 PE=1 SV=1	0,131909061	1,382458
Q9BSL1	Ubiquitin-associated domain-containing protein 1 OS=Homo sapiens OX=9606 GN=UBAC1 PE=1 SV=1	0,563340964	1,381018
P48723	Heat shock 70 kDa protein 13 OS=Homo sapiens OX=9606 GN=HSPA13 PE=1 SV=1	-0,318181758	1,380825
Q13542	Eukaryotic translation initiation factor 4E-binding protein 2 OS=Homo sapiens OX=9606 GN=EIF4EBP2 PE=1 SV=1	0,875281094	1,379526
P31151	Protein S100-A7 OS=Homo sapiens OX=9606 GN=S100A7 PE=1 SV=4	-0,640470273	1,377666
Q13564	NEDD8-activating enzyme E1 regulatory subunit OS=Homo sapiens OX=9606 GN=NAE1 PE=1 SV=1	0,163384619	1,376823
P11234	Ras-related protein Ral-B OS=Homo sapiens OX=9606 GN=RALB PE=1 SV=1	0,639571575	1,376099
Q9H944	Mediator of RNA polymerase II transcription subunit 20 OS=Homo sapiens OX=9606 GN=MED20 PE=1 SV=1	0,638576294	1,374363
Q96ET8	Golgi apparatus membrane protein TVP23 homolog C OS=Homo sapiens OX=9606 GN=TVP23C PE=1 SV=3	0,638359967	1,373985
Q96K76	Ubiquitin carboxyl-terminal hydrolase 47 OS=Homo sapiens OX=9606 GN=USP47 PE=1 SV=3	-0,221044591	1,373291
Q13232	Nucleoside diphosphate kinase 3 OS=Homo sapiens OX=9606 GN=NME3 PE=1 SV=2	0,36276817	1,370997
O14920	Inhibitor of nuclear factor kappa-B kinase subunit beta OS=Homo sapiens OX=9606 GN=IKBKB PE=1 SV=1	-0,636642873	1,370985
Q16527	Cysteine and glycine-rich protein 2 OS=Homo sapiens OX=9606 GN=CSRP2 PE=1 SV=3	-0,20419271	1,370785
Q15836	Vesicle-associated membrane protein 3 OS=Homo sapiens OX=9606 GN=VAMP3 PE=1 SV=3	-0,220698713	1,370692
Q01970	1-phosphatidylinositol 4,5-bisphosphate phosphodiesterase beta-3 OS=Homo sapiens OX=9606 GN=PLCB3 PE=1 SV=2	0,162768523	1,370346
P29590	Protein PML OS=Homo sapiens OX=9606 GN=PML PE=1 SV=3	0,144291336	1,370117
Q8IZ83	Aldehyde dehydrogenase family 16 member A1 OS=Homo sapiens OX=9606 GN=ALDH16A1 PE=1 SV=2	-0,24193185	1,369392
Q8IYB5	Stromal membrane-associated protein 1 OS=Homo sapiens OX=9606 GN=SMAP1 PE=1 SV=2	-0,635714834	1,369361
Q9H3H3	UPF0696 protein C11orf68 OS=Homo sapiens OX=9606 GN=C11orf68 PE=1 SV=3	-0,220399109	1,368441
O14578	Citron Rho-interacting kinase OS=Homo sapiens OX=9606 GN=CIT PE=1 SV=2	-0,393484171	1,368351
P57678	Gem-associated protein 4 OS=Homo sapiens OX=9606 GN=GEMIN4 PE=1 SV=2	0,168152972	1,36767
O00764	Pyridoxal kinase OS=Homo sapiens OX=9606 GN=PDXK PE=1 SV=1	-0,190502088	1,366913

Q9UM54	Unconventional myosin-VI OS=Homo sapiens OX=9606 GN=MYO6 PE=1 SV=4	-0,149395135	1,366326
Q16666	Gamma-interferon-inducible protein 16 OS=Homo sapiens OX=9606 GN=IFI16 PE=1 SV=3	-0,19028547	1,365
Q14515	SPARC-like protein 1 OS=Homo sapiens OX=9606 GN=SPARCL1 PE=1 SV=2	1,005870469	1,364086
P61026	Ras-related protein Rab-10 OS=Homo sapiens OX=9606 GN=RAB10 PE=1 SV=1	-0,179077019	1,362138
Q9UGV2	Protein NDRG3 OS=Homo sapiens OX=9606 GN=NDRG3 PE=1 SV=2	0,313595705	1,358447
Q8IUD2	ELKS/Rab6-interacting/CAST family member 1 OS=Homo sapiens OX=9606 GN=ERC1 PE=1 SV=1	0,178553612	1,357215
O14786	Neuropilin-1 OS=Homo sapiens OX=9606 GN=NRP1 PE=1 SV=3	0,148594675	1,357152
P45985	Dual specificity mitogen-activated protein kinase kinase 4 OS=Homo sapiens OX=9606 GN=MAP2K4 PE=1 SV=1	-0,628489429	1,356675
P13010	X-ray repair cross-complementing protein 5 OS=Homo sapiens OX=9606 GN=XRCC5 PE=1 SV=3	0,099552184	1,356069
Q9Y3Q3	Transmembrane emp24 domain-containing protein 3 OS=Homo sapiens OX=9606 GN=TMED3 PE=1 SV=1	-0,627687404	1,355262
O60613	Selenoprotein F OS=Homo sapiens OX=9606 GN=SELENOF PE=1 SV=4	-0,312862891	1,354872
P49207	60S ribosomal protein L34 OS=Homo sapiens OX=9606 GN=RPL34 PE=1 SV=3	0,218500668	1,354197
O15027	Protein transport protein Sec16A OS=Homo sapiens OX=9606 GN=SEC16A PE=1 SV=4	-0,111459428	1,35167
Q8TDZ2	[F-actin]-monooxygenase MICAL1 OS=Homo sapiens OX=9606 GN=MICAL1 PE=1 SV=2	-0,142707241	1,351244
Q16762	Thiosulfate sulfurtransferase OS=Homo sapiens OX=9606 GN=TST PE=1 SV=4	-0,312089416	1,351101
P0DPB6	DNA-directed RNA polymerases I and III subunit RPAC2 OS=Homo sapiens OX=9606 GN=POLR1D PE=1 SV=1	0,625315781	1,351076
Q99570	Phosphoinositide 3-kinase regulatory subunit 4 OS=Homo sapiens OX=9606 GN=PIK3R4 PE=1 SV=3	-0,268034141	1,349777
O00217	NADH dehydrogenase [ubiquinone] iron-sulfur protein 8, mitochondrial OS=Homo sapiens OX=9606 GN=NDUFS8 PE=1 SV=1	0,26793661	1,349203
Q53S08	Ras-related protein Rab-6D OS=Homo sapiens OX=9606 GN=RAB6D PE=2 SV=1	0,390695991	1,349184
O75694	Nuclear pore complex protein Nup155 OS=Homo sapiens OX=9606 GN=NUP155 PE=1 SV=1	-0,147825187	1,348351
P15144	Aminopeptidase N OS=Homo sapiens OX=9606 GN=ANPEP PE=1 SV=4	-0,088897868	1,346777
Q8TDH9	Biogenesis of lysosome-related organelles complex 1 subunit 5 OS=Homo sapiens OX=9606 GN=BLOC1S5 PE=1 SV=1	0,782822746	1,346364
P43897	Elongation factor Ts, mitochondrial OS=Homo sapiens OX=9606 GN=TSFM PE=1 SV=2	-0,267425112	1,346196
Q96DZ1	Endoplasmic reticulum lectin 1 OS=Homo sapiens OX=9606 GN=ERLEC1 PE=1 SV=1	-0,201167013	1,34602
O14964	Hepatocyte growth factor-regulated tyrosine kinase substrate OS=Homo sapiens OX=9606 GN=HGS PE=1 SV=1	0,129063388	1,344899
Q9BSD7	Cancer-related nucleoside-triphosphatase OS=Homo sapiens OX=9606 GN=NTPCR PE=1 SV=1	0,621497699	1,34432
P32969	60S ribosomal protein L9 OS=Homo sapiens OX=9606 GN=RPL9 PE=1 SV=1	0,187798535	1,343094
P51398	28S ribosomal protein S29, mitochondrial OS=Homo sapiens OX=9606 GN=DAP3 PE=1 SV=1	-0,38868136	1,341917
Q9Y3E1	Hepatoma-derived growth factor-related protein 3 OS=Homo sapiens OX=9606 GN=HDGFL3 PE=1 SV=1	-0,388615311	1,341678
P05783	#N/D	0,176855118	1,341272
Q8IVL5	Prolyl 3-hydroxylase 2 OS=Homo sapiens OX=9606 GN=P3H2 PE=1 SV=1	-0,176636015	1,339219
Q9Y3B7	39S ribosomal protein L11, mitochondrial OS=Homo sapiens OX=9606 GN=MRPL11 PE=1 SV=1	0,519817227	1,337626
Q9BXI6	TBC1 domain family member 10A OS=Homo sapiens OX=9606 GN=TBC1D10A PE=1 SV=1	-0,52029273	1,337437
Q96E11	Ribosome-recycling factor, mitochondrial OS=Homo sapiens OX=9606 GN=MRRF PE=1 SV=1	2,0435438	1,33632
P48307	Tissue factor pathway inhibitor 2 OS=Homo sapiens OX=9606 GN=TFPI2 PE=1 SV=1	0,308959982	1,335845
P63151	Serine/threonine-protein phosphatase 2A 55 kDa regulatory subunit B alpha isoform OS=Homo sapiens OX=9606 GN=PPP2R2A PE=1 SV	0,159169851	1,332692
Q86W92	Liprin-beta-1 OS=Homo sapiens OX=9606 GN=PPFIBP1 PE=1 SV=2	-0,124494187	1,332116
Q14653	Interferon regulatory factor 3 OS=Homo sapiens OX=9606 GN=IRF3 PE=1 SV=1	-0,614562526	1,331986
Q9HB71	Calcyclin-binding protein OS=Homo sapiens OX=9606 GN=CACYBP PE=1 SV=2	0,17586317	1,331985
Q13330	Metastasis-associated protein MTA1 OS=Homo sapiens OX=9606 GN=MTA1 PE=1 SV=2	0,614153879	1,331257
Q96T51	RUN and FYVE domain-containing protein 1 OS=Homo sapiens OX=9606 GN=RUFY1 PE=1 SV=2	0,14631469	1,331125
O14763	Tumor necrosis factor receptor superfamily member 10B OS=Homo sapiens OX=9606 GN=TNFRSF10B PE=1 SV=2	0,613517897	1,330122
P01033	Metalloproteinase inhibitor 1 OS=Homo sapiens OX=9606 GN=TIMP1 PE=1 SV=1	-0,264475561	1,328871
P40939	Trifunctional enzyme subunit alpha, mitochondrial OS=Homo sapiens OX=9606 GN=HADHA PE=1 SV=2	0,091826306	1,328086
Q969H8	Myeloid-derived growth factor OS=Homo sapiens OX=9606 GN=MYDGF PE=1 SV=1	-0,235707908	1,327483
P24534	Elongation factor 1-beta OS=Homo sapiens OX=9606 GN=EEF1B2 PE=1 SV=3	0,175226992	1,326038
O95429	BAG family molecular chaperone regulator 4 OS=Homo sapiens OX=9606 GN=BAG4 PE=1 SV=1	0,384195051	1,325717
O95833	Chloride intracellular channel protein 3 OS=Homo sapiens OX=9606 GN=CLIC3 PE=1 SV=2	0,26390812	1,325541
P50402	Emerin OS=Homo sapiens OX=9606 GN=EMD PE=1 SV=1	0,166195748	1,325515
Q9BSQ5	Cerebral cavernous malformations 2 protein OS=Homo sapiens OX=9606 GN=CCM2 PE=1 SV=1	-0,870350492	1,325288
P23588	Eukaryotic translation initiation factor 4B OS=Homo sapiens OX=9606 GN=EIF4B PE=1 SV=2	0,114746227	1,324121
Q8WVV9	Heterogeneous nuclear ribonucleoprotein L-like OS=Homo sapiens OX=9606 GN=HNRNPLL PE=1 SV=1	0,234995263	1,3227
O60232	Protein ZNRD2 OS=Homo sapiens OX=9606 GN=ZNRD2 PE=1 SV=1	-0,38310644	1,321782
Q9BTZ2	Dehydrogenase/reductase SDR family member 4 OS=Homo sapiens OX=9606 GN=DHRS4 PE=1 SV=3	0,681136221	1,321275
Q9Y696	Chloride intracellular channel protein 4 OS=Homo sapiens OX=9606 GN=CLIC4 PE=1 SV=4	0,109346579	1,319281
O14558	Heat shock protein beta-6 OS=Homo sapiens OX=9606 GN=HSPB6 PE=1 SV=2	-0,19777916	1,318421
Q13595	Transformer-2 protein homolog alpha OS=Homo sapiens OX=9606 GN=TRA2A PE=1 SV=1	-0,606953241	1,318365
O95352	Ubiquitin-like modifier-activating enzyme ATG7 OS=Homo sapiens OX=9606 GN=ATG7 PE=1 SV=1	-0,197704161	1,317811
P35221	Catenin alpha-1 OS=Homo sapiens OX=9606 GN=CTNNA1 PE=1 SV=1	0,08291898	1,317746
Q14562	ATP-dependent RNA helicase DHX8 OS=Homo sapiens OX=9606 GN=DHX8 PE=1 SV=1	0,606408721	1,317387
Q9H4L5	Oxysterol-binding protein-related protein 3 OS=Homo sapiens OX=9606 GN=OSBPL3 PE=1 SV=1	0,262458414	1,31704
Q96EM0	Trans-3-hydroxy-L-proline dehydratase OS=Homo sapiens OX=9606 GN=L3HYPDH PE=1 SV=2	0,304904691	1,316091
Q5HYK7	SH3 domain-containing protein 19 OS=Homo sapiens OX=9606 GN=SH3D19 PE=1 SV=2	0,592480412	1,315331
Q99714	3-hydroxyacyl-CoA dehydrogenase type-2 OS=Homo sapiens OX=9606 GN=HSD17B10 PE=1 SV=3	0,144855716	1,31455
P11362	Fibroblast growth factor receptor 1 OS=Homo sapiens OX=9606 GN=FGFR1 PE=1 SV=3	0,326599052	1,314084
P22413	Ectonucleotide pyrophosphatase/phosphodiesterase family member 1 OS=Homo sapiens OX=9606 GN=ENPP1 PE=1 SV=2	-0,100621626	1,313788
Q9Y6K0	Choline/ethanolaminephosphotransferase 1 OS=Homo sapiens OX=9606 GN=CEPT1 PE=1 SV=1	0,603687774	1,31249
Q9NY12	H/ACA ribonucleoprotein complex subunit 1 OS=Homo sapiens OX=9606 GN=GAR1 PE=1 SV=1	0,380265439	1,311508
Q86TI2	Dipeptidyl peptidase 9 OS=Homo sapiens OX=9606 GN=DPP9 PE=1 SV=3	-0,261121228	1,309206
P17844	Probable ATP-dependent RNA helicase DDX5 OS=Homo sapiens OX=9606 GN=DDX5 PE=1 SV=1	0,134074928	1,304549
P20020	Plasma membrane calcium-transporting ATPase 1 OS=Homo sapiens OX=9606 GN=ATP2B1 PE=1 SV=4	0,122411275	1,304099
Q9BTE6	Alanyl-tRNA editing protein Aarsd1 OS=Homo sapiens OX=9606 GN=AARSD1 PE=1 SV=2	-0,302350097	1,303656
Q92734	Protein TFG OS=Homo sapiens OX=9606 GN=TFG PE=1 SV=2	-0,183224213	1,303041
P30044	Peroxiredoxin-5, mitochondrial OS=Homo sapiens OX=9606 GN=PRDX5 PE=1 SV=4	-0,163915106	1,303018
Q13905	Rap guanine nucleotide exchange factor 1 OS=Homo sapiens OX=9606 GN=RAPGEF1 PE=1 SV=3	0,447257039	1,302411
Q9NWB1	RNA binding protein fox-1 homolog 1 OS=Homo sapiens OX=9606 GN=RBFOX1 PE=1 SV=2	-1,194661628	1,300618
P61970	Nuclear transport factor 2 OS=Homo sapiens OX=9606 GN=NUTF2 PE=1 SV=1	-0,231579439	1,29982
P33121	Long-chain-fatty-acid--CoA ligase 1 OS=Homo sapiens OX=9606 GN=ACSL1 PE=1 SV=1	0,182850556	1,299784
Q9UKM7	Endoplasmic reticulum mannosyl-oligosaccharide 1,2-alpha-mannosidase OS=Homo sapiens OX=9606 GN=MAN1B1 PE=1 SV=2	-0,231494541	1,299252
Q8TCG1	Protein CIP2A OS=Homo sapiens OX=9606 GN=CIP2A PE=1 SV=2	-0,376839317	1,299106
Q14738	Serine/threonine-protein phosphatase 2A 56 kDa regulatory subunit delta isoform OS=Homo sapiens OX=9606 GN=PPP2R5D PE=1 SV=1	0,149323105	1,2991
Q9NUU7	ATP-dependent RNA helicase DDX19A OS=Homo sapiens OX=9606 GN=DDX19A PE=1 SV=1	0,301410655	1,299085
Q6NZ67	Mitotic-spindle organizing protein 2B OS=Homo sapiens OX=9606 GN=MZT2B PE=1 SV=1	-0,59574535	1,29813
O95456	Proteasome assembly chaperone 1 OS=Homo sapiens OX=9606 GN=PSMG1 PE=1 SV=1	-0,301148682	1,29781
O75368	SH3 domain-binding glutamic acid-rich-like protein OS=Homo sapiens OX=9606 GN=SH3BGRL PE=1 SV=1	0,231274634	1,297782
Q9NYB0	Telomeric repeat-binding factor 2-interacting protein 1 OS=Homo sapiens OX=9606 GN=TERF2IP PE=1 SV=1	0,364606311	1,294417
Q02218	2-oxoglutarate dehydrogenase, mitochondrial OS=Homo sapiens OX=9606 GN=OGDH PE=1 SV=3	0,097564987	1,292762
P27816	Microtubule-associated protein 4 OS=Homo sapiens OX=9606 GN=MAP4 PE=1 SV=3	0,071593319	1,291106
Q6NXE6	Armadillo repeat-containing protein 6 OS=Homo sapiens OX=9606 GN=ARMC6 PE=1 SV=2	-0,299707769	1,290801
Q9Y3A6	Transmembrane emp24 domain-containing protein 5 OS=Homo sapiens OX=9606 GN=TMED5 PE=1 SV=1	0,591491643	1,290396
Q9P0K7	Ankycorbin OS=Homo sapiens OX=9606 GN=RAI14 PE=1 SV=2	0,101070987	1,290153
P54098	DNA polymerase subunit gamma-1 OS=Homo sapiens OX=9606 GN=POLG PE=1 SV=1	-0,644072707	1,289889
Q8NE71	ATP-binding cassette sub-family F member 1 OS=Homo sapiens OX=9606 GN=ABCF1 PE=1 SV=2	0,137438172	1,289044
O94763	Unconventional prefoldin RPB5 interactor 1 OS=Homo sapiens OX=9606 GN=URI1 PE=1 SV=3	-0,590147267	1,287945
Q13263	Transcription intermediary factor 1-beta OS=Homo sapiens OX=9606 GN=TRIM28 PE=1 SV=5	0,09888135	1,285086
P34897	Serine hydroxymethyltransferase, mitochondrial OS=Homo sapiens OX=9606 GN=SHMT2 PE=1 SV=3	0,120813869	1,282742
Q07960	Rho GTPase-activating protein 1 OS=Homo sapiens OX=9606 GN=ARHGAP1 PE=1 SV=1	-0,124156937	1,280915
O14683	Tumor protein p53-inducible protein 11 OS=Homo sapiens OX=9606 GN=TP53I11 PE=1 SV=2	0,585862469	1,280115

Q93062	RNA-binding protein with multiple splicing OS=Homo sapiens OX=9606 GN=RBPMS PE=1 SV=1	0,371497923	1,279746
Q9UBQ0	Vacuolar protein sorting-associated protein 29 OS=Homo sapiens OX=9606 GN=VPS29 PE=1 SV=1	-0,180484086	1,279199
O15143	Actin-related protein 2/3 complex subunit 1B OS=Homo sapiens OX=9606 GN=ARPC1B PE=1 SV=3	0,127806407	1,279019
Q14008	Cytoskeleton-associated protein 5 OS=Homo sapiens OX=9606 GN=CKAP5 PE=1 SV=3	0,07996227	1,278143
P19387	DNA-directed RNA polymerase II subunit RPB3 OS=Homo sapiens OX=9606 GN=POLR2C PE=1 SV=2	-0,150757391	1,27801
Q9H2G2	STE20-like serine/threonine-protein kinase OS=Homo sapiens OX=9606 GN=SLK PE=1 SV=1	0,127692987	1,277598
Q8TAA9	Vang-like protein 1 OS=Homo sapiens OX=9606 GN=VANGL1 PE=1 SV=1	-0,363775259	1,277191
Q9C0D5	Protein TANC1 OS=Homo sapiens OX=9606 GN=TANC1 PE=1 SV=3	1,166621841	1,276334
Q9ULA0	Aspartyl aminopeptidase OS=Homo sapiens OX=9606 GN=DNPEP PE=1 SV=1	-0,29671165	1,276236
P11166	Solute carrier family 2, facilitated glucose transporter member 1 OS=Homo sapiens OX=9606 GN=SLC2A1 PE=1 SV=2	0,254712232	1,271752
O95782	AP-2 complex subunit alpha-1 OS=Homo sapiens OX=9606 GN=AP2A1 PE=1 SV=3	-0,078497151	1,267676
P15531	Nucleoside diphosphate kinase A OS=Homo sapiens OX=9606 GN=NME1 PE=1 SV=1	0,168892174	1,267214
Q01081	Splicing factor U2AF 35 kDa subunit OS=Homo sapiens OX=9606 GN=U2AF1 PE=1 SV=3	-0,226636607	1,266846
Q9NTZ6	RNA-binding protein 12 OS=Homo sapiens OX=9606 GN=RBM12 PE=1 SV=1	-0,294280719	1,264426
P26440	Isovaleryl-CoA dehydrogenase, mitochondrial OS=Homo sapiens OX=9606 GN=IVD PE=1 SV=2	0,190823426	1,262192
Q13011	Delta(3,5)-Delta(2,4)-dienoyl-CoA isomerase, mitochondrial OS=Homo sapiens OX=9606 GN=ECH1 PE=1 SV=2	0,190400254	1,25879
P20936	Ras GTPase-activating protein 1 OS=Homo sapiens OX=9606 GN=RASA1 PE=1 SV=1	-0,139791203	1,257497
Q13243	Serine/arginine-rich splicing factor 5 OS=Homo sapiens OX=9606 GN=SRSF5 PE=1 SV=1	-0,365205063	1,2569
P20290	Transcription factor BTF3 OS=Homo sapiens OX=9606 GN=BTF3 PE=1 SV=1	0,177679921	1,254918
Q9HAU5	Regulator of nonsense transcripts 2 OS=Homo sapiens OX=9606 GN=UPF2 PE=1 SV=1	-0,571592223	1,253818
Q13636	Ras-related protein Rab-31 OS=Homo sapiens OX=9606 GN=RAB31 PE=1 SV=1	0,523201733	1,252386
O43660	Pleiotropic regulator 1 OS=Homo sapiens OX=9606 GN=PLRG1 PE=1 SV=1	-0,570562752	1,251907
Q96SB4	SRSF protein kinase 1 OS=Homo sapiens OX=9606 GN=SRPK1 PE=1 SV=2	0,570472314	1,25174
P49754	Vacuolar protein sorting-associated protein 41 homolog OS=Homo sapiens OX=9606 GN=VPS41 PE=1 SV=3	0,291624583	1,251531
Q96KR1	Zinc finger RNA-binding protein OS=Homo sapiens OX=9606 GN=ZFR PE=1 SV=2	0,167179333	1,251433
O75436	Vacuolar protein sorting-associated protein 26A OS=Homo sapiens OX=9606 GN=VPS26A PE=1 SV=2	-0,167132256	1,251
P63000	Ras-related C3 botulinum toxin substrate 1 OS=Homo sapiens OX=9606 GN=RAC1 PE=1 SV=1	0,291232099	1,249627
P61088	Ubiquitin-conjugating enzyme E2 N OS=Homo sapiens OX=9606 GN=UBE2N PE=1 SV=1	0,166721566	1,247225
Q13492	Phosphatidylinositol-binding clathrin assembly protein OS=Homo sapiens OX=9606 GN=PICALM PE=1 SV=2	0,11496744	1,246699
Q99615	DnaJ homolog subfamily C member 7 OS=Homo sapiens OX=9606 GN=DNAJC7 PE=1 SV=2	-0,158149277	1,246626
Q15785	Mitochondrial import receptor subunit TOM34 OS=Homo sapiens OX=9606 GN=TOMM34 PE=1 SV=2	0,204009705	1,246564
O15523	ATP-dependent RNA helicase DDX3Y OS=Homo sapiens OX=9606 GN=DDX3Y PE=1 SV=2	0,290107128	1,244169
Q9ULS6	Potassium voltage-gated channel subfamily S member 2 OS=Homo sapiens OX=9606 GN=KCNS2 PE=1 SV=2	0,565664345	1,242794
Q7Z7A1	Centriolin OS=Homo sapiens OX=9606 GN=CNTRL PE=1 SV=2	-0,919992036	1,242517
O15127	Secretory carrier-associated membrane protein 2 OS=Homo sapiens OX=9606 GN=SCAMP2 PE=1 SV=2	0,361160703	1,242198
Q14112	Nidogen-2 OS=Homo sapiens OX=9606 GN=NID2 PE=1 SV=3	0,508441507	1,242186
P62306	Small nuclear ribonucleoprotein F OS=Homo sapiens OX=9606 GN=SNRPF PE=1 SV=1	0,361128366	1,24208
P62328	Thymosin beta-4 OS=Homo sapiens OX=9606 GN=TMSB4X PE=1 SV=2	0,361083586	1,241918
P40227	T-complex protein 1 subunit zeta OS=Homo sapiens OX=9606 GN=CCT6A PE=1 SV=3	0,102029776	1,241657
Q9NUJ1	Mycophenolic acid acyl-glucuronide esterase, mitochondrial OS=Homo sapiens OX=9606 GN=ABHD10 PE=1 SV=1	-0,222270891	1,237859
Q8N6T3	ADP-ribosylation factor GTPase-activating protein 1 OS=Homo sapiens OX=9606 GN=ARFGAP1 PE=1 SV=2	-0,187650953	1,236745
O15091	Mitochondrial ribonuclease P catalytic subunit OS=Homo sapiens OX=9606 GN=KIAA0391 PE=1 SV=2	0,47031154	1,23573
Q14624	Inter-alpha-trypsin inhibitor heavy chain H4 OS=Homo sapiens OX=9606 GN=ITIH4 PE=1 SV=4	-0,358651984	1,233071
P18124	60S ribosomal protein L7 OS=Homo sapiens OX=9606 GN=RPL7 PE=1 SV=1	0,137593748	1,232979
Q9NQ88	Fructose-2,6-bisphosphatase TIGAR OS=Homo sapiens OX=9606 GN=TIGAR PE=1 SV=1	0,221255811	1,231138
Q15293	Reticulocalbin-1 OS=Homo sapiens OX=9606 GN=RCN1 PE=1 SV=1	-0,116907203	1,230982
Q15437	Protein transport protein Sec23B OS=Homo sapiens OX=9606 GN=SEC23B PE=1 SV=2	-0,174861003	1,23063
O00161	Synaptosomal-associated protein 23 OS=Homo sapiens OX=9606 GN=SNAP23 PE=1 SV=1	0,186883449	1,230608
Q86WB0	Nuclear-interacting partner of ALK OS=Homo sapiens OX=9606 GN=ZC3HC1 PE=1 SV=1	0,357766364	1,229847
Q01813	ATP-dependent 6-phosphofructokinase, platelet type OS=Homo sapiens OX=9606 GN=PFKP PE=1 SV=2	0,097233466	1,22871
O94903	Pyridoxal phosphate homeostasis protein OS=Homo sapiens OX=9606 GN=PLPBP PE=1 SV=1	0,247119323	1,227588
P28799	Progranulin OS=Homo sapiens OX=9606 GN=GRN PE=1 SV=2	-0,186472991	1,227329
P14866	Heterogeneous nuclear ribonucleoprotein L OS=Homo sapiens OX=9606 GN=HNRNPL PE=1 SV=2	0,119942814	1,226746
P09234	U1 small nuclear ribonucleoprotein C OS=Homo sapiens OX=9606 GN=SNRPC PE=1 SV=1	-0,356911135	1,226734
Q9H330	Transmembrane protein 245 OS=Homo sapiens OX=9606 GN=TMEM245 PE=1 SV=3	-1,079749141	1,226153
P55212	Caspase-6 OS=Homo sapiens OX=9606 GN=CASP6 PE=1 SV=2	-0,286368158	1,226042
P35237	Serpin B6 OS=Homo sapiens OX=9606 GN=SERPINB6 PE=1 SV=3	0,127552894	1,225766
Q16555	Dihydropyrimidinase-related protein 2 OS=Homo sapiens OX=9606 GN=DPYSL2 PE=1 SV=1	0,098930932	1,22532
Q9Y2Z0	Protein SGT1 homolog OS=Homo sapiens OX=9606 GN=SUGT1 PE=1 SV=3	0,123473762	1,225072
P31946	14-3-3 protein beta/alpha OS=Homo sapiens OX=9606 GN=YWHAB PE=1 SV=3	0,186052912	1,223975
O00193	Small acidic protein OS=Homo sapiens OX=9606 GN=SMAP PE=1 SV=1	0,285839242	1,223479
Q9UDY2	Tight junction protein ZO-2 OS=Homo sapiens OX=9606 GN=TJP2 PE=1 SV=2	0,142217798	1,222802
O95831	Apoptosis-inducing factor 1, mitochondrial OS=Homo sapiens OX=9606 GN=AIFM1 PE=1 SV=1	0,185888513	1,222663
Q7L311	Armadillo repeat-containing X-linked protein 2 OS=Homo sapiens OX=9606 GN=ARMCX2 PE=2 SV=1	-0,219972228	1,222649
Q9BXP5	Serrate RNA effector molecule homolog OS=Homo sapiens OX=9606 GN=SRRT PE=1 SV=1	0,163887649	1,221256
Q8ND56	Protein LSM14 homolog A OS=Homo sapiens OX=9606 GN=LSM14A PE=1 SV=3	-0,355399577	1,221229
P25398	40S ribosomal protein S12 OS=Homo sapiens OX=9606 GN=RPS12 PE=1 SV=3	0,185303756	1,217999
Q969S9	Ribosome-releasing factor 2, mitochondrial OS=Homo sapiens OX=9606 GN=GFM2 PE=1 SV=1	-0,220988403	1,217101
O75146	Huntingtin-interacting protein 1-related protein OS=Homo sapiens OX=9606 GN=HIP1R PE=1 SV=2	1,755168282	1,217012
O95644	Nuclear factor of activated T-cells, cytoplasmic 1 OS=Homo sapiens OX=9606 GN=NFATC1 PE=1 SV=3	-0,218730575	1,215693
O75880	Protein SCO1 homolog, mitochondrial OS=Homo sapiens OX=9606 GN=SCO1 PE=1 SV=1	-0,551101917	1,215461
Q9BRX2	Protein pelota homolog OS=Homo sapiens OX=9606 GN=PELO PE=1 SV=2	-0,284059453	1,214859
Q7LBC6	Lysine-specific demethylase 3B OS=Homo sapiens OX=9606 GN=KDM3B PE=1 SV=2	-0,549752015	1,212909
Q9NR12	PDZ and LIM domain protein 7 OS=Homo sapiens OX=9606 GN=PDLIM7 PE=1 SV=1	-0,112385074	1,211764
P62877	E3 ubiquitin-protein ligase RBX1 OS=Homo sapiens OX=9606 GN=RBX1 PE=1 SV=1	-0,548990297	1,211468
Q6UXH1	Cysteine-rich with EGF-like domain protein 2 OS=Homo sapiens OX=9606 GN=CRELD2 PE=1 SV=1	0,199157987	1,210975
Q9Y243	RAC-gamma serine/threonine-protein kinase OS=Homo sapiens OX=9606 GN=AKT3 PE=1 SV=1	0,126650257	1,210532
Q6P3X3	Tetratricopeptide repeat protein 27 OS=Homo sapiens OX=9606 GN=TTC27 PE=1 SV=1	-0,282930314	1,209393
O43741	5'-AMP-activated protein kinase subunit beta-2 OS=Homo sapiens OX=9606 GN=PRKAB2 PE=1 SV=1	0,351897807	1,20847
P62906	60S ribosomal protein L10a OS=Homo sapiens OX=9606 GN=RPL10A PE=1 SV=2	0,140754808	1,20726
Q9UNW1	Multiple inositol polyphosphate phosphatase 1 OS=Homo sapiens OX=9606 GN=MINPP1 PE=1 SV=1	0,183845697	1,20639
Q8N5N7	39S ribosomal protein L50, mitochondrial OS=Homo sapiens OX=9606 GN=MRPL50 PE=1 SV=2	-0,546153898	1,206092
Q6L8Q7	2',5'-phosphodiesterase 12 OS=Homo sapiens OX=9606 GN=PDE12 PE=1 SV=2	-0,545801912	1,205424
Q9NVE7	Pantothenate kinase 4 OS=Homo sapiens OX=9606 GN=PANK4 PE=1 SV=1	-0,282104004	1,205394
Q9NPA8	Transcription and mRNA export factor ENY2 OS=Homo sapiens OX=9606 GN=ENY2 PE=1 SV=1	-0,545228643	1,204336
Q6UWF9	Protein FAM180A OS=Homo sapiens OX=9606 GN=FAM180A PE=2 SV=1	0,133117507	1,203273
P48651	Phosphatidylserine synthase 1 OS=Homo sapiens OX=9606 GN=PTDSS1 PE=1 SV=1	0,543410225	1,200879
Q96BW5	Phosphotriesterase-related protein OS=Homo sapiens OX=9606 GN=PTER PE=1 SV=1	-0,543251975	1,200578
P14927	Cytochrome b-c1 complex subunit 7 OS=Homo sapiens OX=9606 GN=UQCRB PE=1 SV=2	-0,349502748	1,199738
P17612	cAMP-dependent protein kinase catalytic subunit alpha OS=Homo sapiens OX=9606 GN=PRKACA PE=1 SV=2	-0,153167794	1,19847
Q8IWB1	Inositol 1,4,5-trisphosphate receptor-interacting protein OS=Homo sapiens OX=9606 GN=ITPRIP PE=1 SV=1	-0,242040402	1,19818
P48449	Lanosterol synthase OS=Homo sapiens OX=9606 GN=LSS PE=1 SV=1	0,161167432	1,196469
P09669	Cytochrome c oxidase subunit 6C OS=Homo sapiens OX=9606 GN=COX6C PE=1 SV=2	-0,348007374	1,194284
Q86UT6	NLR family member X1 OS=Homo sapiens OX=9606 GN=NLRX1 PE=1 SV=1	-0,251991366	1,191883
Q15532	Protein SSXT OS=Homo sapiens OX=9606 GN=SS18 PE=1 SV=3	-0,537690139	1,18997
Q4J6C6	Prolyl endopeptidase-like OS=Homo sapiens OX=9606 GN=PREPL PE=1 SV=1	0,240437896	1,188925
P51636	Caveolin-2 OS=Homo sapiens OX=9606 GN=CAV2 PE=1 SV=2	-0,536875591	1,188412
Q15181	Inorganic pyrophosphatase OS=Homo sapiens OX=9606 GN=PPA1 PE=1 SV=2	-0,133395723	1,186538

Q01484	Ankyrin-2 OS=Homo sapiens OX=9606 GN=ANK2 PE=1 SV=4	0,094421433	1,186288
P09543	2',3'-cyclic-nucleotide 3'-phosphodiesterase OS=Homo sapiens OX=9606 GN=CNP PE=1 SV=2	0,128523631	1,18586
P36507	Dual specificity mitogen-activated protein kinase kinase 2 OS=Homo sapiens OX=9606 GN=MAP2K2 PE=1 SV=1	0,15175047	1,184865
Q5VSL9	Striatin-interacting protein 1 OS=Homo sapiens OX=9606 GN=STRIP1 PE=1 SV=1	-0,214085397	1,183867
O60826	Coiled-coil domain-containing protein 22 OS=Homo sapiens OX=9606 GN=CCDC22 PE=1 SV=1	0,239380876	1,182826
P11586	C-1-tetrahydrofolate synthase, cytoplasmic OS=Homo sapiens OX=9606 GN=MTHFD1 PE=1 SV=3	0,085017025	1,181836
P00387	NADH-cytochrome b5 reductase 3 OS=Homo sapiens OX=9606 GN=CYB5R3 PE=1 SV=3	0,123756721	1,180605
Q9C005	Protein dpy-30 homolog OS=Homo sapiens OX=9606 GN=DPY30 PE=1 SV=1	-0,344136022	1,180156
Q96JJ3	Engulfment and cell motility protein 2 OS=Homo sapiens OX=9606 GN=ELMO2 PE=1 SV=2	-0,127977731	1,179625
Q96KG9	N-terminal kinase-like protein OS=Homo sapiens OX=9606 GN=SCYL1 PE=1 SV=1	0,102126932	1,177601
O95825	Quinone oxidoreductase-like protein 1 OS=Homo sapiens OX=9606 GN=CRYZL1 PE=1 SV=2	-0,343377903	1,177388
Q96KP1	Exocyst complex component 2 OS=Homo sapiens OX=9606 GN=EXOC2 PE=1 SV=1	-0,194373541	1,176107
Q9UET6	Putative tRNA (cytidine(32)/guanosine(34)-2'-O)-methyltransferase OS=Homo sapiens OX=9606 GN=FTSJ1 PE=1 SV=2	0,342725694	1,175007
O75223	Gamma-glutamylcyclotransferase OS=Homo sapiens OX=9606 GN=GGCT PE=1 SV=1	0,342522115	1,174263
O15160	DNA-directed RNA polymerases I and III subunit RPAC1 OS=Homo sapiens OX=9606 GN=POLR1C PE=1 SV=1	0,342181668	1,17302
O94967	WD repeat-containing protein 47 OS=Homo sapiens OX=9606 GN=WDR47 PE=1 SV=1	-0,528756563	1,17282
Q05086	Ubiquitin-protein ligase E3A OS=Homo sapiens OX=9606 GN=UBE3A PE=1 SV=4	-0,212334515	1,17238
O75955	Flotillin-1 OS=Homo sapiens OX=9606 GN=FLOT1 PE=1 SV=3	0,212307677	1,172204
Q96DB5	Regulator of microtubule dynamics protein 1 OS=Homo sapiens OX=9606 GN=RMDN1 PE=1 SV=1	0,158430502	1,171667
Q9Y547	Intraflagellar transport protein 25 homolog OS=Homo sapiens OX=9606 GN=HSPB11 PE=1 SV=1	0,52812315	1,171599
P30530	Tyrosine-protein kinase receptor UFO OS=Homo sapiens OX=9606 GN=AXL PE=1 SV=4	0,341349453	1,16998
O75179	Ankyrin repeat domain-containing protein 17 OS=Homo sapiens OX=9606 GN=ANKRD17 PE=1 SV=3	-0,722182584	1,169799
Q92819	Hyaluronan synthase 2 OS=Homo sapiens OX=9606 GN=HAS2 PE=1 SV=1	-0,723868462	1,169672
Q96FV9	THO complex subunit 1 OS=Homo sapiens OX=9606 GN=THOC1 PE=1 SV=1	-0,365232699	1,168963
Q9NX55	Huntingtin-interacting protein K OS=Homo sapiens OX=9606 GN=HYPK PE=1 SV=2	-0,526199646	1,167887
P35269	General transcription factor IIF subunit 1 OS=Homo sapiens OX=9606 GN=GTF2F1 PE=1 SV=2	-0,193135976	1,167125
Q13427	Peptidyl-prolyl cis-trans isomerase G OS=Homo sapiens OX=9606 GN=PPIG PE=1 SV=2	-0,525361681	1,166267
Q9P253	Vacuolar protein sorting-associated protein 18 homolog OS=Homo sapiens OX=9606 GN=VPS18 PE=1 SV=2	0,192919281	1,165554
Q8IWR0	Zinc finger CCCH domain-containing protein 7A OS=Homo sapiens OX=9606 GN=ZC3H7A PE=1 SV=1	-0,524866055	1,165309
Q6IA86	Elongator complex protein 2 OS=Homo sapiens OX=9606 GN=ELP2 PE=1 SV=2	0,273360169	1,163149
Q6GMV2	SET and MYND domain-containing protein 5 OS=Homo sapiens OX=9606 GN=SMYD5 PE=1 SV=2	-0,27317937	1,162277
Q9NUI1	Peroxisomal 2,4-dienoyl-CoA reductase OS=Homo sapiens OX=9606 GN=DECR2 PE=1 SV=1	-0,523285107	1,162249
Q13188	Serine/threonine-protein kinase 3 OS=Homo sapiens OX=9606 GN=STK3 PE=1 SV=2	-0,195873262	1,161086
Q6Q759	Sperm-associated antigen 17 OS=Homo sapiens OX=9606 GN=SPAG17 PE=2 SV=1	0,522005982	1,15977
Q9UHD2	Serine/threonine-protein kinase TBK1 OS=Homo sapiens OX=9606 GN=TBK1 PE=1 SV=1	0,521873774	1,159514
O94905	Erlin-2 OS=Homo sapiens OX=9606 GN=ERLIN2 PE=1 SV=1	0,157045954	1,159174
Q9P258	Protein RCC2 OS=Homo sapiens OX=9606 GN=RCC2 PE=1 SV=2	-0,177862094	1,159032
Q15555	Microtubule-associated protein RP/EB family member 2 OS=Homo sapiens OX=9606 GN=MAPRE2 PE=1 SV=1	0,351959603	1,158915
P09661	U2 small nuclear ribonucleoprotein A' OS=Homo sapiens OX=9606 GN=SNRPA1 PE=1 SV=2	0,210219286	1,158533
Q96HH9	GRAM domain-containing protein 2B OS=Homo sapiens OX=9606 GN=GRAMD2B PE=1 SV=1	-0,33813428	1,158233
Q9UKI2	Cdc42 effector protein 3 OS=Homo sapiens OX=9606 GN=CDC42EP3 PE=1 SV=1	0,210137149	1,157995
Q02539	Histone H1.1 OS=Homo sapiens OX=9606 GN=HIST1H1A PE=1 SV=3	-0,181653122	1,156802
P05114	Non-histone chromosomal protein HMG-14 OS=Homo sapiens OX=9606 GN=HMGN1 PE=1 SV=3	0,271837461	1,155806
Q14573	Inositol 1,4,5-trisphosphate receptor type 3 OS=Homo sapiens OX=9606 GN=ITPR3 PE=1 SV=2	-0,141756153	1,154832
P17252	Protein kinase C alpha type OS=Homo sapiens OX=9606 GN=PRKCA PE=1 SV=4	0,12561247	1,152727
Q63HN8	E3 ubiquitin-protein ligase RNF213 OS=Homo sapiens OX=9606 GN=RNF213 PE=1 SV=3	0,191139483	1,152668
P24386	Rab proteins geranylgeranyltransferase component A 1 OS=Homo sapiens OX=9606 GN=CHM PE=1 SV=3	0,559849905	1,1516
P34741	Syndecan-2 OS=Homo sapiens OX=9606 GN=SDC2 PE=1 SV=2	0,336216392	1,151223
P25788	Proteasome subunit alpha type-3 OS=Homo sapiens OX=9606 GN=PSMA3 PE=1 SV=2	-0,148220171	1,151166
P36551	Oxygen-dependent coproporphyrinogen-III oxidase, mitochondrial OS=Homo sapiens OX=9606 GN=CPOX PE=1 SV=3	0,517376218	1,150775
Q9BPZ7	Target of rapamycin complex 2 subunit MAPKAP1 OS=Homo sapiens OX=9606 GN=MAPKAP1 PE=1 SV=2	-0,468117155	1,150155
P30501	HLA class I histocompatibility antigen, Cw-2 alpha chain OS=Homo sapiens OX=9606 GN=HLA-C PE=1 SV=1	-0,335385412	1,148185
O75787	Renin receptor OS=Homo sapiens OX=9606 GN=ATP6AP2 PE=1 SV=2	0,395530226	1,147518
P15927	Replication protein A 32 kDa subunit OS=Homo sapiens OX=9606 GN=RPA2 PE=1 SV=1	0,334965617	1,14665
Q3ZCQ8	Mitochondrial import inner membrane translocase subunit TIM50 OS=Homo sapiens OX=9606 GN=TIMM50 PE=1 SV=2	-0,23283928	1,145192
P51808	Dynein light chain Tctex-type 3 OS=Homo sapiens OX=9606 GN=DYNLT3 PE=1 SV=1	-0,26952454	1,14466
Q9Y3X0	Coiled-coil domain-containing protein 9 OS=Homo sapiens OX=9606 GN=CCDC9 PE=1 SV=1	-0,513762686	1,143729
Q9UH99	SUN domain-containing protein 2 OS=Homo sapiens OX=9606 GN=SUN2 PE=1 SV=3	-0,155293445	1,143412
P57088	Transmembrane protein 33 OS=Homo sapiens OX=9606 GN=TMEM33 PE=1 SV=2	-0,269169674	1,14295
Q86Y56	Dynein assembly factor 5, axonemal OS=Homo sapiens OX=9606 GN=DNAAF5 PE=1 SV=4	-0,333571143	1,14155
Q12792	Twinfilin-1 OS=Homo sapiens OX=9606 GN=TWF1 PE=1 SV=3	0,140367138	1,14096
P82673	28S ribosomal protein S35, mitochondrial OS=Homo sapiens OX=9606 GN=MRPS35 PE=1 SV=1	0,33272148	1,138442
Q96C90	Protein phosphatase 1 regulatory subunit 14B OS=Homo sapiens OX=9606 GN=PPP1R14B PE=1 SV=3	-0,268116904	1,137881
P25789	Proteasome subunit alpha type-4 OS=Homo sapiens OX=9606 GN=PSMA4 PE=1 SV=1	-0,14679489	1,137636
P28290	Protein ITPRID2 OS=Homo sapiens OX=9606 GN=ITPRID2 PE=1 SV=3	0,510630016	1,137602
Q9Y295	Developmentally-regulated GTP-binding protein 1 OS=Homo sapiens OX=9606 GN=DRG1 PE=1 SV=1	0,154600464	1,137195
Q5JTB6	Placenta-specific protein 9 OS=Homo sapiens OX=9606 GN=PLAC9 PE=1 SV=1	0,510026565	1,13642
Q9H019	Mitochondrial fission regulator 1-like OS=Homo sapiens OX=9606 GN=MTFR1L PE=1 SV=2	0,509808541	1,135993
P26640	Valine--tRNA ligase OS=Homo sapiens OX=9606 GN=VARS PE=1 SV=4	0,078664796	1,133922
P54821	Paired mesoderm homeobox protein 1 OS=Homo sapiens OX=9606 GN=PRRX1 PE=1 SV=2	0,508605491	1,133634
P32321	Deoxycytidylate deaminase OS=Homo sapiens OX=9606 GN=DCTD PE=1 SV=2	-0,230592428	1,132311
Q9P265	Disco-interacting protein 2 homolog B OS=Homo sapiens OX=9606 GN=DIP2B PE=1 SV=3	-0,330780845	1,131343
Q9P016	Thymocyte nuclear protein 1 OS=Homo sapiens OX=9606 GN=THYN1 PE=1 SV=1	-0,507366224	1,131201
O43505	Beta-1,4-glucuronyltransferase 1 OS=Homo sapiens OX=9606 GN=B4GAT1 PE=1 SV=1	0,507087491	1,130653
Q96AY3	Peptidyl-prolyl cis-trans isomerase FKBP10 OS=Homo sapiens OX=9606 GN=FKBP10 PE=1 SV=1	-0,087663045	1,130235
Q92600	CCR4-NOT transcription complex subunit 9 OS=Homo sapiens OX=9606 GN=CNOT9 PE=1 SV=1	0,646286052	1,129145
P34059	N-acetylgalactosamine-6-sulfatase OS=Homo sapiens OX=9606 GN=GALNS PE=1 SV=1	-0,329782831	1,127691
P62273	40S ribosomal protein S29 OS=Homo sapiens OX=9606 GN=RPS29 PE=1 SV=2	-0,265907845	1,127252
Q9Y6M5	Zinc transporter 1 OS=Homo sapiens OX=9606 GN=SLC30A1 PE=1 SV=3	-0,205366549	1,126889
Q8NBN3	Transmembrane protein 87A OS=Homo sapiens OX=9606 GN=TMEM87A PE=1 SV=3	0,329535165	1,126784
Q9Y2I1	Nischarin OS=Homo sapiens OX=9606 GN=NISCH PE=1 SV=3	-0,194151139	1,125889
O60443	Gasdermin-E OS=Homo sapiens OX=9606 GN=GSDME PE=1 SV=2	-0,229445576	1,125746
P48061	Stromal cell-derived factor 1 OS=Homo sapiens OX=9606 GN=CXCL12 PE=1 SV=1	0,504451945	1,12547
Q96J02	E3 ubiquitin-protein ligase Itchy homolog OS=Homo sapiens OX=9606 GN=ITCH PE=1 SV=2	-0,187315475	1,125093
Q9Y399	28S ribosomal protein S2, mitochondrial OS=Homo sapiens OX=9606 GN=MRPS2 PE=1 SV=1	-0,328521272	1,123074
Q9UHQ4	B-cell receptor-associated protein 29 OS=Homo sapiens OX=9606 GN=BCAP29 PE=1 SV=2	0,228593918	1,120874
Q8WXF7	Atlastin-1 OS=Homo sapiens OX=9606 GN=ATL1 PE=1 SV=1	0,327885539	1,120747
Q9NYJ8	TGF-beta-activated kinase 1 and MAP3K7-binding protein 2 OS=Homo sapiens OX=9606 GN=TAB2 PE=1 SV=1	0,918845737	1,120345
P62879	Guanine nucleotide-binding protein G(I)/G(S)/G(T) subunit beta-2 OS=Homo sapiens OX=9606 GN=GNB2 PE=1 SV=3	0,327481512	1,119268
Q9Y2K2	Serine/threonine-protein kinase SIK3 OS=Homo sapiens OX=9606 GN=SIK3 PE=1 SV=4	0,505750983	1,119121
P27658	Collagen alpha-1(VIII) chain OS=Homo sapiens OX=9606 GN=COL8A1 PE=1 SV=2	0,3272974	1,118594
Q13541	Eukaryotic translation initiation factor 4E-binding protein 1 OS=Homo sapiens OX=9606 GN=EIF4EBP1 PE=1 SV=3	-0,327185056	1,118183
P40261	Nicotinamide N-methyltransferase OS=Homo sapiens OX=9606 GN=NNMT PE=1 SV=1	0,138059171	1,118016
Q9BTE3	Mini-chromosome maintenance complex-binding protein OS=Homo sapiens OX=9606 GN=MCMBP PE=1 SV=2	0,500116788	1,116917
Q9H2D6	TRIO and F-actin-binding protein OS=Homo sapiens OX=9606 GN=TRIOBP PE=1 SV=3	0,122425647	1,116777
Q15843	NEDD8 OS=Homo sapiens OX=9606 GN=NEDD8 PE=1 SV=1	-0,263722918	1,116747
P48506	Glutamate--cysteine ligase catalytic subunit OS=Homo sapiens OX=9606 GN=GCLC PE=1 SV=2	0,499838877	1,116367
Q9H553	Alpha-1,3/1,6-mannosyltransferase ALG2 OS=Homo sapiens OX=9606 GN=ALG2 PE=1 SV=1	-0,227762804	1,116124

Q9Y697	Cysteine desulfurase, mitochondrial OS=Homo sapiens OX=9606 GN=NFS1 PE=1 SV=3	0,263591911	1,116118
Q13951	Core-binding factor subunit beta OS=Homo sapiens OX=9606 GN=CBFB PE=1 SV=2	0,326554875	1,115876
Q9UL45	Biogenesis of lysosome-related organelles complex 1 subunit 6 OS=Homo sapiens OX=9606 GN=BLOC1S6 PE=1 SV=1	0,499192927	1,11509
P17405	Sphingomyelin phosphodiesterase OS=Homo sapiens OX=9606 GN=SMPD1 PE=1 SV=5	0,227477064	1,114491
Q99733	Nucleosome assembly protein 1-like 4 OS=Homo sapiens OX=9606 GN=NAP1L4 PE=1 SV=1	-0,161134209	1,114137
Q9H9A5	CCR4-NOT transcription complex subunit 10 OS=Homo sapiens OX=9606 GN=CNOT10 PE=1 SV=1	-0,49773599	1,112206
O15446	DNA-directed RNA polymerase I subunit RPA34 OS=Homo sapiens OX=9606 GN=CD3EAP PE=1 SV=1	0,175506626	1,112202
P14854	Cytochrome c oxidase subunit 6B1 OS=Homo sapiens OX=9606 GN=COX6B1 PE=1 SV=2	-0,227004901	1,111794
Q9NVT9	Armadillo repeat-containing protein 1 OS=Homo sapiens OX=9606 GN=ARMC1 PE=1 SV=1	0,49673442	1,110221
Q08170	Serine/arginine-rich splicing factor 4 OS=Homo sapiens OX=9606 GN=SRSF4 PE=1 SV=2	0,092897413	1,109892
P49674	Casein kinase I isoform epsilon OS=Homo sapiens OX=9606 GN=CSNK1E PE=1 SV=1	-0,365411738	1,109057
Q9H7D0	Dedicator of cytokinesis protein 5 OS=Homo sapiens OX=9606 GN=DOCK5 PE=1 SV=3	-0,107471746	1,108749
Q96CP2	FLYWCH family member 2 OS=Homo sapiens OX=9606 GN=FLYWCH2 PE=1 SV=1	0,495969923	1,108705
Q16531	DNA damage-binding protein 1 OS=Homo sapiens OX=9606 GN=DDB1 PE=1 SV=1	-0,082051483	1,10853
Q3LXA3	Triokinase/FMN cyclase OS=Homo sapiens OX=9606 GN=TKFC PE=1 SV=2	0,202502366	1,108296
Q9Y512	Sorting and assembly machinery component 50 homolog OS=Homo sapiens OX=9606 GN=SAMM50 PE=1 SV=3	-0,160331431	1,107417
P18847	Cyclic AMP-dependent transcription factor ATF-3 OS=Homo sapiens OX=9606 GN=ATF3 PE=1 SV=2	0,302394081	1,106477
Q9H9T3	Elongator complex protein 3 OS=Homo sapiens OX=9606 GN=ELP3 PE=1 SV=2	-0,323752109	1,105613
Q9H0V9	VIP36-like protein OS=Homo sapiens OX=9606 GN=LMAN2L PE=1 SV=1	0,225857516	1,105246
P61599	N-alpha-acetyltransferase 20 OS=Homo sapiens OX=9606 GN=NAA20 PE=1 SV=1	-0,49394568	1,104686
P10599	Thioredoxin OS=Homo sapiens OX=9606 GN=TXN PE=1 SV=3	-0,184429547	1,104382
P02458	Collagen alpha-1(II) chain OS=Homo sapiens OX=9606 GN=COL2A1 PE=1 SV=3	-0,201491023	1,101745
P24752	Acetyl-CoA acetyltransferase, mitochondrial OS=Homo sapiens OX=9606 GN=ACAT1 PE=1 SV=1	-0,121061669	1,101494
Q53TN4	Cytochrome b reductase 1 OS=Homo sapiens OX=9606 GN=CYBRD1 PE=1 SV=1	-0,492150559	1,101116
Q13428	Treacle protein OS=Homo sapiens OX=9606 GN=TCOF1 PE=1 SV=3	0,260241862	1,100032
P49747	Cartilage oligomeric matrix protein OS=Homo sapiens OX=9606 GN=COMP PE=1 SV=2	-0,224921574	1,099908
Q9BY89	Uncharacterized protein KIAA1671 OS=Homo sapiens OX=9606 GN=KIAA1671 PE=1 SV=2	-0,072690013	1,09841
Q7Z6Z7	E3 ubiquitin-protein ligase HUWE1 OS=Homo sapiens OX=9606 GN=HUWE1 PE=1 SV=3	-0,065502615	1,09782
Q16659	Mitogen-activated protein kinase 6 OS=Homo sapiens OX=9606 GN=MAPK6 PE=1 SV=1	0,490119657	1,09707
Q86VX2	COMM domain-containing protein 7 OS=Homo sapiens OX=9606 GN=COMMD7 PE=1 SV=2	-0,253501443	1,096868
Q9UII2	ATPase inhibitor, mitochondrial OS=Homo sapiens OX=9606 GN=ATP5IF1 PE=1 SV=1	-0,489936193	1,096704
P52179	Myomesin-1 OS=Homo sapiens OX=9606 GN=MYOM1 PE=1 SV=2	0,489777068	1,096387
P18754	Regulator of chromosome condensation OS=Homo sapiens OX=9606 GN=RCC1 PE=1 SV=1	0,321226391	1,096362
Q86Y82	Syntaxin-12 OS=Homo sapiens OX=9606 GN=STX12 PE=1 SV=1	0,149819571	1,094553
P33176	Kinesin-1 heavy chain OS=Homo sapiens OX=9606 GN=KIF5B PE=1 SV=1	-0,063394	1,094354
Q9NP80	Calcium-independent phospholipase A2-gamma OS=Homo sapiens OX=9606 GN=PNPLA8 PE=1 SV=1	0,352555308	1,093591
Q96DI7	U5 small nuclear ribonucleoprotein 40 kDa protein OS=Homo sapiens OX=9606 GN=SNRNP40 PE=1 SV=1	-0,223670209	1,092779
P10398	Serine/threonine-protein kinase A-Raf OS=Homo sapiens OX=9606 GN=ARAF PE=1 SV=2	-0,592878867	1,092284
Q04917	14-3-3 protein eta OS=Homo sapiens OX=9606 GN=YWHAH PE=1 SV=4	0,12462128	1,091183
Q9BQA9	Cytochrome b-245 chaperone 1 OS=Homo sapiens OX=9606 GN=CYBC1 PE=1 SV=1	0,31956802	1,090286
P86791	Vacuolar fusion protein CCZ1 homolog OS=Homo sapiens OX=9606 GN=CCZ1 PE=1 SV=1	0,617681101	1,089333
Q92620	Pre-mRNA-splicing factor ATP-dependent RNA helicase PRP16 OS=Homo sapiens OX=9606 GN=DHX38 PE=1 SV=2	0,199485686	1,088781
Q9C0I1	Myotubularin-related protein 12 OS=Homo sapiens OX=9606 GN=MTMR12 PE=1 SV=2	0,146225951	1,088698
P48637	Glutathione synthetase OS=Homo sapiens OX=9606 GN=GSS PE=1 SV=1	-0,135037057	1,088171
Q6IEE8	Schlafen family member 12-like OS=Homo sapiens OX=9606 GN=SLFN12L PE=2 SV=4	-0,657033534	1,088143
Q7Z2T5	TRMT1-like protein OS=Homo sapiens OX=9606 GN=TRMT1L PE=1 SV=2	0,967187513	1,087321
Q14847	LIM and SH3 domain protein 1 OS=Homo sapiens OX=9606 GN=LASP1 PE=1 SV=2	0,100309957	1,086031
Q8NCH0	Carbohydrate sulfotransferase 14 OS=Homo sapiens OX=9606 GN=CHST14 PE=1 SV=2	0,484309747	1,085456
Q15050	Ribosome biogenesis regulatory protein homolog OS=Homo sapiens OX=9606 GN=RRS1 PE=1 SV=2	0,222360954	1,085328
Q969J3	BLOC-1-related complex subunit 5 OS=Homo sapiens OX=9606 GN=BORCS5 PE=1 SV=1	-0,367442895	1,083503
Q9HD15	Steroid receptor RNA activator 1 OS=Homo sapiens OX=9606 GN=SRA1 PE=1 SV=1	0,221856795	1,082462
Q9NUP1	Biogenesis of lysosome-related organelles complex 1 subunit 4 OS=Homo sapiens OX=9606 GN=BLOC1S4 PE=1 SV=1	0,055967348	1,082359
Q13162	Peroxiredoxin-4 OS=Homo sapiens OX=9606 GN=PRDX4 PE=1 SV=1	0,140835911	1,081551
P60891	Ribose-phosphate pyrophosphokinase 1 OS=Homo sapiens OX=9606 GN=PRPS1 PE=1 SV=2	0,198328768	1,081315
Q9UNF1	Melanoma-associated antigen D2 OS=Homo sapiens OX=9606 GN=MAGED2 PE=1 SV=2	0,108308689	1,081013
P51648	Aldehyde dehydrogenase family 3 member A2 OS=Homo sapiens OX=9606 GN=ALDH3A2 PE=1 SV=1	0,140747613	1,080726
Q13523	Serine/threonine-protein kinase PRP4 homolog OS=Homo sapiens OX=9606 GN=PRPF4B PE=1 SV=3	-0,481719457	1,08026
Q8WTW3	Conserved oligomeric Golgi complex subunit 1 OS=Homo sapiens OX=9606 GN=COG1 PE=1 SV=1	0,316205084	1,077963
P11802	Cyclin-dependent kinase 4 OS=Homo sapiens OX=9606 GN=CDK4 PE=1 SV=2	-0,376791228	1,077017
P10606	Cytochrome c oxidase subunit 5B, mitochondrial OS=Homo sapiens OX=9606 GN=COX5B PE=1 SV=2	-0,255407123	1,076861
Q13045	Protein flightless-1 homolog OS=Homo sapiens OX=9606 GN=FLII PE=1 SV=2	0,089148618	1,076238
Q96HD1	Cysteine-rich with EGF-like domain protein 1 OS=Homo sapiens OX=9606 GN=CRELD1 PE=1 SV=3	0,255036599	1,075087
P11940	Polyadenylate-binding protein 1 OS=Homo sapiens OX=9606 GN=PABPC1 PE=1 SV=2	0,114621975	1,074049
Q13443	Disintegrin and metalloproteinase domain-containing protein 9 OS=Homo sapiens OX=9606 GN=ADAM9 PE=1 SV=1	0,220283747	1,073525
Q13555	Calcium/calmodulin-dependent protein kinase type II subunit gamma OS=Homo sapiens OX=9606 GN=CAMK2G PE=1 SV=4	-0,314700207	1,072447
Q9P225	Dynein heavy chain 2, axonemal OS=Homo sapiens OX=9606 GN=DNAH2 PE=2 SV=3	0,934752291	1,071827
P23743	Diacylglycerol kinase alpha OS=Homo sapiens OX=9606 GN=DGKA PE=1 SV=3	-0,166636811	1,071455
Q92542	Nicastrin OS=Homo sapiens OX=9606 GN=NCSTN PE=1 SV=2	-0,314159155	1,070464
Q7Z7K0	COX assembly mitochondrial protein homolog OS=Homo sapiens OX=9606 GN=CMC1 PE=1 SV=1	0,476481772	1,069717
O94992	Protein HEXIM1 OS=Homo sapiens OX=9606 GN=HEXIM1 PE=1 SV=1	-0,476234297	1,069218
O14617	AP-3 complex subunit delta-1 OS=Homo sapiens OX=9606 GN=AP3D1 PE=1 SV=1	-0,104329687	1,068923
P51580	Thiopurine S-methyltransferase OS=Homo sapiens OX=9606 GN=TPMT PE=1 SV=1	0,166182657	1,067948
Q8N2F6	Armadillo repeat-containing protein 10 OS=Homo sapiens OX=9606 GN=ARMC10 PE=1 SV=1	0,475119487	1,066967
Q9NNW5	WD repeat-containing protein 6 OS=Homo sapiens OX=9606 GN=WDR6 PE=1 SV=1	0,219038953	1,066462
O75381	Peroxisomal membrane protein PEX14 OS=Homo sapiens OX=9606 GN=PEX14 PE=1 SV=1	0,179088131	1,066283
P24043	Laminin subunit alpha-2 OS=Homo sapiens OX=9606 GN=LAMA2 PE=1 SV=4	0,474530179	1,065776
Q641Q2	WASH complex subunit 2A OS=Homo sapiens OX=9606 GN=WASHC2A PE=1 SV=3	0,357744744	1,065159
Q8N7H5	RNA polymerase II-associated factor 1 homolog OS=Homo sapiens OX=9606 GN=PAF1 PE=1 SV=2	0,312298967	1,063644
Q6P3W7	SCY1-like protein 2 OS=Homo sapiens OX=9606 GN=SCYL2 PE=1 SV=1	0,178710838	1,063603
O14662	Syntaxin-16 OS=Homo sapiens OX=9606 GN=STX16 PE=1 SV=3	-0,473150139	1,062986
Q9Y5S2	Serine/threonine-protein kinase MRCK beta OS=Homo sapiens OX=9606 GN=CDC42BPB PE=1 SV=2	0,098476509	1,061613
O75475	PC4 and SFRS1-interacting protein OS=Homo sapiens OX=9606 GN=PSIP1 PE=1 SV=1	-0,252212999	1,061581
P34949	Mannose-6-phosphate isomerase OS=Homo sapiens OX=9606 GN=MPI PE=1 SV=2	0,195257526	1,061548
Q13616	Cullin-1 OS=Homo sapiens OX=9606 GN=CUL1 PE=1 SV=2	0,138675911	1,061414
O43181	NADH dehydrogenase [ubiquinone] iron-sulfur protein 4, mitochondrial OS=Homo sapiens OX=9606 GN=NDUFS4 PE=1 SV=1	-0,311688817	1,061407
Q86VR2	Reticulophagy regulator 3 OS=Homo sapiens OX=9606 GN=RETREG3 PE=1 SV=1	0,218025455	1,060717
O96005	Cleft lip and palate transmembrane protein 1 OS=Homo sapiens OX=9606 GN=CLPTM1 PE=1 SV=1	0,165244535	1,060711
Q8WUM0	Nuclear pore complex protein Nup133 OS=Homo sapiens OX=9606 GN=NUP133 PE=1 SV=2	-0,145980979	1,060632
O43583	Density-regulated protein OS=Homo sapiens OX=9606 GN=DENR PE=1 SV=2	-0,194936207	1,059484
P55884	Eukaryotic translation initiation factor 3 subunit B OS=Homo sapiens OX=9606 GN=EIF3B PE=1 SV=3	-0,080395152	1,059252
O75792	Ribonuclease H2 subunit A OS=Homo sapiens OX=9606 GN=RNASEH2A PE=1 SV=2	-0,471117194	1,058869
Q96SU4	Oxysterol-binding protein-related protein 9 OS=Homo sapiens OX=9606 GN=OSBPL9 PE=1 SV=2	-0,310304056	1,05633
Q96P50	Arf-GAP with coiled-coil, ANK repeat and PH domain-containing protein 3 OS=Homo sapiens OX=9606 GN=ACAP3 PE=1 SV=2	0,29207527	1,056239
Q9Y478	5'-AMP-activated protein kinase subunit beta-1 OS=Homo sapiens OX=9606 GN=PRKAB1 PE=1 SV=4	0,108962926	1,056117
O95967	EGF-containing fibulin-like extracellular matrix protein 2 OS=Homo sapiens OX=9606 GN=EFEMP2 PE=1 SV=3	0,217197657	1,056029
Q9UHV9	Prefoldin subunit 2 OS=Homo sapiens OX=9606 GN=PFDN2 PE=1 SV=1	0,194322461	1,055544
P62424	60S ribosomal protein L7a OS=Homo sapiens OX=9606 GN=RPL7A PE=1 SV=2	-0,112966618	1,055061
Q9HAV0	Guanine nucleotide-binding protein subunit beta-4 OS=Homo sapiens OX=9606 GN=GNB4 PE=1 SV=3	0,137890674	1,054119

Q9UKF6	Cleavage and polyadenylation specificity factor subunit 3 OS=Homo sapiens OX=9606 GN=CPSF3 PE=1 SV=1	0,309515879	1,05344
Q6AI08	HEAT repeat-containing protein 6 OS=Homo sapiens OX=9606 GN=HEATR6 PE=1 SV=1	0,042628893	1,052237
P17900	Ganglioside GM2 activator OS=Homo sapiens OX=9606 GN=GM2A PE=1 SV=4	0,467385854	1,051295
P32456	Guanylate-binding protein 2 OS=Homo sapiens OX=9606 GN=GBP2 PE=1 SV=3	-0,216360083	1,051289
Q9GZN8	UPF0687 protein C20orf27 OS=Homo sapiens OX=9606 GN=C20orf27 PE=1 SV=3	-0,109079258	1,050741
Q8IVF7	Formin-like protein 3 OS=Homo sapiens OX=9606 GN=FMNL3 PE=1 SV=3	-0,607750363	1,050722
Q15819	Ubiquitin-conjugating enzyme E2 variant 2 OS=Homo sapiens OX=9606 GN=UBE2V2 PE=1 SV=4	-0,249854384	1,050313
Q96ER9	Coiled-coil domain-containing protein 51 OS=Homo sapiens OX=9606 GN=CCDC51 PE=1 SV=2	-0,182369678	1,049439
Q13228	Methanethiol oxidase OS=Homo sapiens OX=9606 GN=SELENBP1 PE=1 SV=2	-0,11633014	1,048956
A0A0U1RRL7	Protein MMP24OS OS=Homo sapiens OX=9606 GN=MMP24OS PE=1 SV=1	-0,466154387	1,04879
P50552	Vasodilator-stimulated phosphoprotein OS=Homo sapiens OX=9606 GN=VASP PE=1 SV=3	0,108829096	1,048082
Q9Y6Y8	SEC23-interacting protein OS=Homo sapiens OX=9606 GN=SEC23IP PE=1 SV=1	0,11621539	1,047691
Q9UL15	BAG family molecular chaperone regulator 5 OS=Homo sapiens OX=9606 GN=BAG5 PE=1 SV=1	0,215644104	1,04724
Q8WXA9	Splicing regulatory glutamine/lysine-rich protein 1 OS=Homo sapiens OX=9606 GN=SREK1 PE=1 SV=1	0,307689789	1,046744
Q15477	Helicase SKI2W OS=Homo sapiens OX=9606 GN=SKIV2L PE=1 SV=3	-0,130628818	1,045044
P55084	Trifunctional enzyme subunit beta, mitochondrial OS=Homo sapiens OX=9606 GN=HADHB PE=1 SV=3	-0,102409816	1,044805
Q9NXU5	ADP-ribosylation factor-like protein 15 OS=Homo sapiens OX=9606 GN=ARL15 PE=1 SV=1	-0,462590524	1,044161
O75131	Copine-3 OS=Homo sapiens OX=9606 GN=CPNE3 PE=1 SV=1	0,111983623	1,043833
Q5MNZ9	WD repeat domain phosphoinositide-interacting protein 1 OS=Homo sapiens OX=9606 GN=WIPI1 PE=1 SV=3	0,463540101	1,043463
Q8N128	Protein FAM177A1 OS=Homo sapiens OX=9606 GN=FAM177A1 PE=1 SV=1	-0,463526579	1,043436
Q8N392	Rho GTPase-activating protein 18 OS=Homo sapiens OX=9606 GN=ARHGAP18 PE=1 SV=3	0,143988183	1,043133
P62714	Serine/threonine-protein phosphatase 2A catalytic subunit beta isoform OS=Homo sapiens OX=9606 GN=PPP2CB PE=1 SV=1	-0,115796566	1,043078
P12814	Alpha-actinin-1 OS=Homo sapiens OX=9606 GN=ACTN1 PE=1 SV=2	-0,053625426	1,042676
Q08623	Pseudouridine-5'-phosphatase OS=Homo sapiens OX=9606 GN=PUDP PE=1 SV=3	0,120863291	1,04177
Q9Y6B7	AP-4 complex subunit beta-1 OS=Homo sapiens OX=9606 GN=AP4B1 PE=1 SV=2	0,18330568	1,041047
P60981	Destrin OS=Homo sapiens OX=9606 GN=DSTN PE=1 SV=3	-0,119914988	1,041002
P53990	IST1 homolog OS=Homo sapiens OX=9606 GN=IST1 PE=1 SV=1	0,175350823	1,039807
Q9ULP9	TBC1 domain family member 24 OS=Homo sapiens OX=9606 GN=TBC1D24 PE=1 SV=2	-0,305116695	1,037307
Q86X83	COMM domain-containing protein 2 OS=Homo sapiens OX=9606 GN=COMMD2 PE=1 SV=2	-0,247023959	1,036809
Q96JH7	Deubiquitinating protein VCIP135 OS=Homo sapiens OX=9606 GN=VCPIP1 PE=1 SV=2	-0,246995772	1,036674
Q9NX46	ADP-ribose glycohydrolase ARH3 OS=Homo sapiens OX=9606 GN=ADPRHL2 PE=1 SV=1	0,246765388	1,035576
P12107	Collagen alpha-1(XI) chain OS=Homo sapiens OX=9606 GN=COL11A1 PE=1 SV=4	1,28994476	1,034026
Q15629	Translocating chain-associated membrane protein 1 OS=Homo sapiens OX=9606 GN=TRAM1 PE=1 SV=3	0,213299751	1,034001
Q14232	Translation initiation factor eIF-2B subunit alpha OS=Homo sapiens OX=9606 GN=EIF2B1 PE=1 SV=1	-0,151464738	1,033896
Q06587	E3 ubiquitin-protein ligase RING1 OS=Homo sapiens OX=9606 GN=RING1 PE=1 SV=2	-0,458421988	1,033001
Q12955	Ankyrin-3 OS=Homo sapiens OX=9606 GN=ANK3 PE=1 SV=3	0,213045237	1,032565
O00505	Importin subunit alpha-4 OS=Homo sapiens OX=9606 GN=KPNA3 PE=1 SV=2	-0,151104789	1,030939
P0CW20	LIM and senescent cell antigen-like-containing domain protein 4 OS=Homo sapiens OX=9606 GN=LIMS4 PE=1 SV=1	-0,302950317	1,029361
Q15139	Serine/threonine-protein kinase D1 OS=Homo sapiens OX=9606 GN=PRKD1 PE=1 SV=2	0,302516281	1,027769
Q15149	Plectin OS=Homo sapiens OX=9606 GN=PLEC PE=1 SV=3	-0,025862171	1,027669
Q9H269	Vacuolar protein sorting-associated protein 16 homolog OS=Homo sapiens OX=9606 GN=VPS16 PE=1 SV=2	-0,302162626	1,026471
P36776	Lon protease homolog, mitochondrial OS=Homo sapiens OX=9606 GN=LONP1 PE=1 SV=2	0,09576197	1,025772
P12955	Xaa-Pro dipeptidase OS=Homo sapiens OX=9606 GN=PEPD PE=1 SV=3	-0,118452386	1,025545
P08134	Rho-related GTP-binding protein RhoC OS=Homo sapiens OX=9606 GN=RHOC PE=1 SV=1	-0,15037162	1,024922
Q8N4V1	Membrane magnesium transporter 1 OS=Homo sapiens OX=9606 GN=MMGT1 PE=1 SV=1	0,454359985	1,024666
P41567	Eukaryotic translation initiation factor 1 OS=Homo sapiens OX=9606 GN=EIF1 PE=1 SV=1	-0,172958412	1,022939
Q9NWH9	SAFB-like transcription modulator OS=Homo sapiens OX=9606 GN=SLTM PE=1 SV=2	0,453198726	1,022277
P36269	Glutathione hydrolase 5 proenzyme OS=Homo sapiens OX=9606 GN=GGT5 PE=1 SV=2	0,28025001	1,021467
Q7L273	BTB/POZ domain-containing protein KCTD9 OS=Homo sapiens OX=9606 GN=KCTD9 PE=1 SV=1	0,363825549	1,020638
P51828	Adenylate cyclase type 7 OS=Homo sapiens OX=9606 GN=ADCY7 PE=1 SV=1	-0,4518233	1,019446
Q9BZE9	Tether containing UBX domain for GLUT4 OS=Homo sapiens OX=9606 GN=ASPSCR1 PE=1 SV=1	-0,210678177	1,01923
O76054	SEC14-like protein 2 OS=Homo sapiens OX=9606 GN=SEC14L2 PE=1 SV=1	0,451309216	1,018387
P18206	Vinculin OS=Homo sapiens OX=9606 GN=VCL PE=1 SV=4	0,049670094	1,017669
P01891	HLA class I histocompatibility antigen, A-68 alpha chain OS=Homo sapiens OX=9606 GN=HLA-A PE=1 SV=4	-0,450894409	1,017532
P15311	Ezrin OS=Homo sapiens OX=9606 GN=EZR PE=1 SV=4	0,085093845	1,016248
Q00341	Vigilin OS=Homo sapiens OX=9606 GN=HDLBP PE=1 SV=2	0,056496817	1,016129
Q9BPX5	Actin-related protein 2/3 complex subunit 5-like protein OS=Homo sapiens OX=9606 GN=ARPC5L PE=1 SV=1	0,171955407	1,015886
Q15434	RNA-binding motif, single-stranded-interacting protein 2 OS=Homo sapiens OX=9606 GN=RBMS2 PE=1 SV=1	-0,187882329	1,014387
Q9Y2V7	Conserved oligomeric Golgi complex subunit 6 OS=Homo sapiens OX=9606 GN=COG6 PE=1 SV=2	0,20975144	1,014018
Q9BTV4	Transmembrane protein 43 OS=Homo sapiens OX=9606 GN=TMEM43 PE=1 SV=1	-0,113093422	1,013447
O95758	Polypyrimidine tract-binding protein 3 OS=Homo sapiens OX=9606 GN=PTBP3 PE=1 SV=2	-0,476490225	1,012631
Q92508	Piezo-type mechanosensitive ion channel component 1 OS=Homo sapiens OX=9606 GN=PIEZO1 PE=1 SV=4	-0,187496963	1,011935
P53602	Diphosphomevalonate decarboxylase OS=Homo sapiens OX=9606 GN=MVD PE=1 SV=1	-0,297991661	1,011171
P43307	Translocon-associated protein subunit alpha OS=Homo sapiens OX=9606 GN=SSR1 PE=1 SV=3	-0,20906924	1,010184
Q16637	Survival motor neuron protein OS=Homo sapiens OX=9606 GN=SMN1 PE=1 SV=1	0,064929158	1,009707
P46939	Utrophin OS=Homo sapiens OX=9606 GN=UTRN PE=1 SV=2	0,053462044	1,009407
P38435	Vitamin K-dependent gamma-carboxylase OS=Homo sapiens OX=9606 GN=GGCX PE=1 SV=2	-0,44674661	1,008966
O94973	AP-2 complex subunit alpha-2 OS=Homo sapiens OX=9606 GN=AP2A2 PE=1 SV=2	0,099428902	1,007685
O75380	NADH dehydrogenase [ubiquinone] iron-sulfur protein 6, mitochondrial OS=Homo sapiens OX=9606 GN=NDUFS6 PE=1 SV=1	-0,240797029	1,00717
P26639	Threonine--tRNA ligase, cytoplasmic OS=Homo sapiens OX=9606 GN=TARS PE=1 SV=3	-0,075921507	1,007085
Q8N668	COMM domain-containing protein 1 OS=Homo sapiens OX=9606 GN=COMMD1 PE=1 SV=1	-0,296587678	1,006021
Q13404	Ubiquitin-conjugating enzyme E2 variant 1 OS=Homo sapiens OX=9606 GN=UBE2V1 PE=1 SV=2	-0,186559026	1,005972
Q8N3V7	Synaptopodin OS=Homo sapiens OX=9606 GN=SYNPO PE=1 SV=2	0,296454321	1,005531
P08397	Porphobilinogen deaminase OS=Homo sapiens OX=9606 GN=HMBS PE=1 SV=2	0,186440468	1,005219
Q1KMD3	Heterogeneous nuclear ribonucleoprotein U-like protein 2 OS=Homo sapiens OX=9606 GN=HNRNPUL2 PE=1 SV=1	0,096394844	1,002451
Q9H4G4	Golgi-associated plant pathogenesis-related protein 1 OS=Homo sapiens OX=9606 GN=GLIPR2 PE=1 SV=3	0,442866882	1,000928
P50897	Palmitoyl-protein thioesterase 1 OS=Homo sapiens OX=9606 GN=PPT1 PE=1 SV=1	-0,239431254	1,000683
P37802	Transgelin-2 OS=Homo sapiens OX=9606 GN=TAGLN2 PE=1 SV=3	-0,089410199	0,999157
P57721	Poly(rC)-binding protein 3 OS=Homo sapiens OX=9606 GN=PCBP3 PE=2 SV=2	0,185443082	0,998887
P28161	Glutathione S-transferase Mu 2 OS=Homo sapiens OX=9606 GN=GSTM2 PE=1 SV=2	-0,147043904	0,997726
Q9NV96	Cell cycle control protein 50A OS=Homo sapiens OX=9606 GN=TMEM30A PE=1 SV=1	-0,238782102	0,997601
Q13724	Mannosyl-oligosaccharide glucosidase OS=Homo sapiens OX=9606 GN=MOGS PE=1 SV=5	0,101411724	0,997287
Q9Y6Y0	Influenza virus NS1A-binding protein OS=Homo sapiens OX=9606 GN=IVNS1ABP PE=1 SV=3	-0,440694986	0,996417
P56134	ATP synthase subunit f, mitochondrial OS=Homo sapiens OX=9606 GN=ATP5MF PE=1 SV=3	-0,238393227	0,995756
Q9BW60	Elongation of very long chain fatty acids protein 1 OS=Homo sapiens OX=9606 GN=ELOVL1 PE=1 SV=1	-0,293775961	0,995706
P62854	40S ribosomal protein S26 OS=Homo sapiens OX=9606 GN=RPS26 PE=1 SV=3	0,2383771	0,995679
O95684	FGFR1 oncogene partner OS=Homo sapiens OX=9606 GN=FGFR1OP PE=1 SV=1	0,515691404	0,99437
Q8NB16	Mixed lineage kinase domain-like protein OS=Homo sapiens OX=9606 GN=MLKL PE=1 SV=1	-0,439378281	0,993678
Q9UKI8	Serine/threonine-protein kinase tousled-like 1 OS=Homo sapiens OX=9606 GN=TLK1 PE=1 SV=2	-0,43859391	0,992045
Q8N0U8	Vitamin K epoxide reductase complex subunit 1-like protein 1 OS=Homo sapiens OX=9606 GN=VKORC1L1 PE=1 SV=2	-0,292231216	0,990039
Q9UJX2	Cell division cycle protein 23 homolog OS=Homo sapiens OX=9606 GN=CDC23 PE=1 SV=3	0,437223868	0,98919
Q12846	Syntaxin-4 OS=Homo sapiens OX=9606 GN=STX4 PE=1 SV=2	-0,183851374	0,988799
Q9NSD9	Phenylalanine--tRNA ligase beta subunit OS=Homo sapiens OX=9606 GN=FARSB PE=1 SV=3	-0,103617589	0,987114
Q9HD67	Unconventional myosin-X OS=Homo sapiens OX=9606 GN=MYO10 PE=1 SV=3	-0,436200919	0,987057
P30533	Alpha-2-macroglobulin receptor-associated protein OS=Homo sapiens OX=9606 GN=LRPAP1 PE=1 SV=1	0,103608941	0,987014
P36578	60S ribosomal protein L4 OS=Homo sapiens OX=9606 GN=RPL4 PE=1 SV=5	-0,084719937	0,986037
Q92604	Acyl-CoA:lysophosphatidylglycerol acyltransferase 1 OS=Homo sapiens OX=9606 GN=LPGAT1 PE=1 SV=1	-0,435598328	0,985799
Q92859	Neogenin OS=Homo sapiens OX=9606 GN=NEO1 PE=1 SV=2	0,43476498	0,984059

P42167	Lamina-associated polypeptide 2, isoforms beta/gamma OS=Homo sapiens OX=9606 GN=TMPO PE=1 SV=2	0,124207423	0,983093
Q8N5B7	Ceramide synthase 5 OS=Homo sapiens OX=9606 GN=CERS5 PE=2 SV=1	0,447592699	0,982964
P63244	Receptor of activated protein C kinase 1 OS=Homo sapiens OX=9606 GN=RACK1 PE=1 SV=3	0,097403384	0,98269
P23258	Tubulin gamma-1 chain OS=Homo sapiens OX=9606 GN=TUBG1 PE=1 SV=2	0,137021797	0,982571
O94842	TOX high mobility group box family member 4 OS=Homo sapiens OX=9606 GN=TOX4 PE=1 SV=1	0,433871382	0,982192
P68366	Tubulin alpha-4A chain OS=Homo sapiens OX=9606 GN=TUBA4A PE=1 SV=1	-0,203973012	0,981622
P11388	DNA topoisomerase 2-alpha OS=Homo sapiens OX=9606 GN=TOP2A PE=1 SV=3	0,094608999	0,979828
Q9BUN8	Derlin-1 OS=Homo sapiens OX=9606 GN=DERL1 PE=1 SV=1	0,432383342	0,979079
P55145	Mesencephalic astrocyte-derived neurotrophic factor OS=Homo sapiens OX=9606 GN=MANF PE=1 SV=3	-0,203466514	0,978791
Q9NVA2	Septin-11 OS=Homo sapiens OX=9606 GN=SEPTIN11 PE=1 SV=3	-0,123757081	0,978788
Q13315	Serine-protein kinase ATM OS=Homo sapiens OX=9606 GN=ATM PE=1 SV=4	-0,468792277	0,978644
P52943	Cysteine-rich protein 2 OS=Homo sapiens OX=9606 GN=CRIP2 PE=1 SV=1	-0,182152682	0,978057
Q9BQ70	Transcription factor 25 OS=Homo sapiens OX=9606 GN=TCF25 PE=1 SV=1	-0,136437004	0,97753
Q9BXT8	RING finger protein 17 OS=Homo sapiens OX=9606 GN=RNF17 PE=1 SV=3	0,144653448	0,977439
P55060	Exportin-2 OS=Homo sapiens OX=9606 GN=CSE1L PE=1 SV=3	0,075243985	0,976401
Q9UBM7	7-dehydrocholesterol reductase OS=Homo sapiens OX=9606 GN=DHCR7 PE=1 SV=1	0,202849988	0,975348
O95139	NADH dehydrogenase [ubiquinone] 1 beta subcomplex subunit 6 OS=Homo sapiens OX=9606 GN=NDUFB6 PE=1 SV=3	0,43028654	0,974687
Q9NWU5	39S ribosomal protein L22, mitochondrial OS=Homo sapiens OX=9606 GN=MRPL22 PE=1 SV=1	0,811180682	0,974554
Q5VZM2	#N/D	0,287989058	0,974477
Q9NY27	Serine/threonine-protein phosphatase 4 regulatory subunit 2 OS=Homo sapiens OX=9606 GN=PPP4R2 PE=1 SV=3	0,287667206	0,973297
Q9UGR2	Zinc finger CCCH domain-containing protein 7B OS=Homo sapiens OX=9606 GN=ZC3H7B PE=1 SV=2	0,202480456	0,973284
Q5JVS0	Intracellular hyaluronan-binding protein 4 OS=Homo sapiens OX=9606 GN=HABP4 PE=1 SV=1	-0,287600989	0,973054
P10746	Uroporphyrinogen-III synthase OS=Homo sapiens OX=9606 GN=UROS PE=1 SV=1	0,429271599	0,972558
P57105	Synaptojanin-2-binding protein OS=Homo sapiens OX=9606 GN=SYNJ2BP PE=1 SV=2	0,509264725	0,97163
Q96FQ6	Protein S100-A16 OS=Homo sapiens OX=9606 GN=S100A16 PE=1 SV=1	0,165511406	0,970838
P15153	Ras-related C3 botulinum toxin substrate 2 OS=Homo sapiens OX=9606 GN=RAC2 PE=1 SV=1	-0,165469403	0,970546
P26572	Alpha-1,3-mannosyl-glycoprotein 2-beta-N-acetylglucosaminyltransferase OS=Homo sapiens OX=9606 GN=MGAT1 PE=1 SV=2	0,42822628	0,970364
O43776	Asparagine--tRNA ligase, cytoplasmic OS=Homo sapiens OX=9606 GN=NARS PE=1 SV=1	-0,087283381	0,970197
Q9P0J7	E3 ubiquitin-protein ligase KCMF1 OS=Homo sapiens OX=9606 GN=KCMF1 PE=1 SV=2	0,428111058	0,970122
Q9NP61	ADP-ribosylation factor GTPase-activating protein 3 OS=Homo sapiens OX=9606 GN=ARFGAP3 PE=1 SV=1	0,143502772	0,968989
Q8WZ42	Titin OS=Homo sapiens OX=9606 GN=TTN PE=1 SV=4	-0,201463271	0,967609
P62937	Peptidyl-prolyl cis-trans isomerase A OS=Homo sapiens OX=9606 GN=PPIA PE=1 SV=2	-0,096172585	0,967592
P30084	Enoyl-CoA hydratase, mitochondrial OS=Homo sapiens OX=9606 GN=ECHS1 PE=1 SV=4	0,135161801	0,966562
Q96H20	Vacuolar-sorting protein SNF8 OS=Homo sapiens OX=9606 GN=SNF8 PE=1 SV=1	-0,426348845	0,966418
Q12931	Heat shock protein 75 kDa, mitochondrial OS=Homo sapiens OX=9606 GN=TRAP1 PE=1 SV=3	-0,12242515	0,966084
Q8WU76	Sec1 family domain-containing protein 2 OS=Homo sapiens OX=9606 GN=SCFD2 PE=1 SV=2	-0,426048797	0,965787
P42858	Huntingtin OS=Homo sapiens OX=9606 GN=HTT PE=1 SV=2	0,285599156	0,965711
Q9NW64	Pre-mRNA-splicing factor RBM22 OS=Homo sapiens OX=9606 GN=RBM22 PE=1 SV=1	0,424718265	0,962986
P84077	ADP-ribosylation factor 1 OS=Homo sapiens OX=9606 GN=ARF1 PE=1 SV=2	0,548650148	0,960948
Q96PK6	RNA-binding protein 14 OS=Homo sapiens OX=9606 GN=RBM14 PE=1 SV=2	0,15194495	0,959426
P30876	DNA-directed RNA polymerase II subunit RPB2 OS=Homo sapiens OX=9606 GN=POLR2B PE=1 SV=1	-0,19998917	0,959396
O95989	Diphosphoinositol polyphosphate phosphohydrolase 1 OS=Homo sapiens OX=9606 GN=NUDT3 PE=1 SV=1	-0,42296165	0,959284
O95373	Importin-7 OS=Homo sapiens OX=9606 GN=IPO7 PE=1 SV=1	0,076742264	0,959262
Q9HB40	Retinoid-inducible serine carboxypeptidase OS=Homo sapiens OX=9606 GN=SCPEP1 PE=1 SV=1	-0,422811893	0,958968
Q13432	Protein unc-119 homolog A OS=Homo sapiens OX=9606 GN=UNC119 PE=1 SV=1	-0,422753835	0,958846
P40429	60S ribosomal protein L13a OS=Homo sapiens OX=9606 GN=RPL13A PE=1 SV=2	0,142152882	0,958091
Q9NQ92	Coordinator of PRMT5 and differentiation stimulator OS=Homo sapiens OX=9606 GN=COPRS PE=1 SV=3	0,282891006	0,955779
Q7L576	Cytoplasmic FMR1-interacting protein 1 OS=Homo sapiens OX=9606 GN=CYFIP1 PE=1 SV=1	0,07384258	0,954221
Q8WVJ2	NudC domain-containing protein 2 OS=Homo sapiens OX=9606 GN=NUDCD2 PE=1 SV=1	-0,282442279	0,954133
Q5T8D3	Acyl-CoA-binding domain-containing protein 5 OS=Homo sapiens OX=9606 GN=ACBD5 PE=1 SV=1	0,282309106	0,953645
P68431	Histone H3.1 OS=Homo sapiens OX=9606 GN=HIST1H3A PE=1 SV=2	0,928579006	0,953126
O43678	NADH dehydrogenase [ubiquinone] 1 alpha subcomplex subunit 2 OS=Homo sapiens OX=9606 GN=NDUFA2 PE=1 SV=3	0,419996074	0,953023
Q92890	Ubiquitin recognition factor in ER-associated degradation protein 1 OS=Homo sapiens OX=9606 GN=UFD1 PE=1 SV=3	-0,133567704	0,952896
O00264	Membrane-associated progesterone receptor component 1 OS=Homo sapiens OX=9606 GN=PGRMC1 PE=1 SV=3	0,178116396	0,95263
Q86WR0	Coiled-coil domain-containing protein 25 OS=Homo sapiens OX=9606 GN=CCDC25 PE=1 SV=2	-0,419103418	0,951135
Q9Y3C0	WASH complex subunit 3 OS=Homo sapiens OX=9606 GN=WASHC3 PE=1 SV=1	0,281328067	0,950047
Q12972	Nuclear inhibitor of protein phosphatase 1 OS=Homo sapiens OX=9606 GN=PPP1R8 PE=1 SV=2	0,417724185	0,948216
Q13596	Sorting nexin-1 OS=Homo sapiens OX=9606 GN=SNX1 PE=1 SV=3	0,126049567	0,945783
Q13131	5'-AMP-activated protein kinase catalytic subunit alpha-1 OS=Homo sapiens OX=9606 GN=PRKAA1 PE=1 SV=4	-0,150082477	0,945441
Q9UL63	Muskelin OS=Homo sapiens OX=9606 GN=MKLN1 PE=1 SV=2	-1,735909042	0,944808
O94804	Serine/threonine-protein kinase 10 OS=Homo sapiens OX=9606 GN=STK10 PE=1 SV=1	-0,140341227	0,943513
Q8IYD1	Eukaryotic peptide chain release factor GTP-binding subunit ERF3B OS=Homo sapiens OX=9606 GN=GSPT2 PE=1 SV=2	-0,279442529	0,943133
Q9H0D6	5'-3' exoribonuclease 2 OS=Homo sapiens OX=9606 GN=XRN2 PE=1 SV=1	-0,132412603	0,943025
P51553	Isocitrate dehydrogenase [NAD] subunit gamma, mitochondrial OS=Homo sapiens OX=9606 GN=IDH3G PE=1 SV=1	0,279369884	0,942867
Q6UW63	Protein O-glucosyltransferase 2 OS=Homo sapiens OX=9606 GN=POGLUT2 PE=1 SV=1	-0,414742452	0,941894
Q6P9B6	MTOR-associated protein MEAK7 OS=Homo sapiens OX=9606 GN=MEAK7 PE=1 SV=2	-0,149500181	0,941078
O00231	26S proteasome non-ATPase regulatory subunit 11 OS=Homo sapiens OX=9606 GN=PSMD11 PE=1 SV=3	-0,085101642	0,940753
Q9BT22	Chitobiosyldiphosphodolichol beta-mannosyltransferase OS=Homo sapiens OX=9606 GN=ALG1 PE=1 SV=2	0,278757245	0,94062
Q15147	1-phosphatidylinositol 4,5-bisphosphate phosphodiesterase beta-4 OS=Homo sapiens OX=9606 GN=PLCB4 PE=1 SV=3	-0,278421729	0,93939
O95630	STAM-binding protein OS=Homo sapiens OX=9606 GN=STAMBP PE=1 SV=1	-0,175738197	0,937714
O43399	Tumor protein D54 OS=Homo sapiens OX=9606 GN=TPD52L2 PE=1 SV=2	0,109965303	0,937156
P47897	Glutamine--tRNA ligase OS=Homo sapiens OX=9606 GN=QARS PE=1 SV=1	-0,069356513	0,936459
Q9UN86	Ras GTPase-activating protein-binding protein 2 OS=Homo sapiens OX=9606 GN=G3BP2 PE=1 SV=2	-0,139455467	0,936406
P61956	Small ubiquitin-related modifier 2 OS=Homo sapiens OX=9606 GN=SUMO2 PE=1 SV=3	-0,388742327	0,93631
P42766	60S ribosomal protein L35 OS=Homo sapiens OX=9606 GN=RPL35 PE=1 SV=2	-0,277472976	0,935912
P60604	Ubiquitin-conjugating enzyme E2 G2 OS=Homo sapiens OX=9606 GN=UBE2G2 PE=1 SV=1	0,41192167	0,9359
Q9BV38	WD repeat-containing protein 18 OS=Homo sapiens OX=9606 GN=WDR18 PE=1 SV=2	0,225698734	0,935743
Q9UHR5	SAP30-binding protein OS=Homo sapiens OX=9606 GN=SAP30BP PE=1 SV=1	-0,411779589	0,935598
Q9NUQ6	SPATS2-like protein OS=Homo sapiens OX=9606 GN=SPATS2L PE=1 SV=2	0,124901763	0,93545
Q5T160	Probable arginine--tRNA ligase, mitochondrial OS=Homo sapiens OX=9606 GN=RARS2 PE=1 SV=1	-0,411262477	0,934497
Q9GZM8	Nuclear distribution protein nudE-like 1 OS=Homo sapiens OX=9606 GN=NDEL1 PE=1 SV=1	-0,242592219	0,934023
Q9Y3A5	Ribosome maturation protein SBDS OS=Homo sapiens OX=9606 GN=SBDS PE=1 SV=4	0,119022233	0,933831
Q9UNZ2	NSFL1 cofactor p47 OS=Homo sapiens OX=9606 GN=NSFL1C PE=1 SV=2	-0,124721118	0,933826
Q9H2J4	Phosducin-like protein 3 OS=Homo sapiens OX=9606 GN=PDCL3 PE=1 SV=1	0,148338882	0,932393
P33947	ER lumen protein-retaining receptor 2 OS=Homo sapiens OX=9606 GN=KDELR2 PE=1 SV=1	0,195105912	0,932276
Q9P015	39S ribosomal protein L15, mitochondrial OS=Homo sapiens OX=9606 GN=MRPL15 PE=1 SV=1	-0,40975238	0,931281
Q03001	Dystonin OS=Homo sapiens OX=9606 GN=DST PE=1 SV=4	0,067991365	0,930693
Q99747	Gamma-soluble NSF attachment protein OS=Homo sapiens OX=9606 GN=NAPG PE=1 SV=1	-0,174501619	0,929978
P81605	Dermcidin OS=Homo sapiens OX=9606 GN=DCD PE=1 SV=2	-0,408659927	0,928952
P35241	Radixin OS=Homo sapiens OX=9606 GN=RDX PE=1 SV=1	-0,072207013	0,928531
P61201	COP9 signalosome complex subunit 2 OS=Homo sapiens OX=9606 GN=COPS2 PE=1 SV=1	-0,138389407	0,927871
Q15005	Signal peptidase complex subunit 2 OS=Homo sapiens OX=9606 GN=SPCS2 PE=1 SV=3	0,159248735	0,927508
Q9H910	Jupiter microtubule associated homolog 2 OS=Homo sapiens OX=9606 GN=JPT2 PE=1 SV=1	0,194227485	0,927412
P54136	Arginine--tRNA ligase, cytoplasmic OS=Homo sapiens OX=9606 GN=RARS PE=1 SV=2	-0,069839347	0,927015
A0FGR8	Extended synaptotagmin-2 OS=Homo sapiens OX=9606 GN=ESYT2 PE=1 SV=1	0,098353464	0,926615
Q00796	Sorbitol dehydrogenase OS=Homo sapiens OX=9606 GN=SORD PE=1 SV=4	-0,13807134	0,925328
P39687	Acidic leucine-rich nuclear phosphoprotein 32 family member A OS=Homo sapiens OX=9606 GN=ANP32A PE=1 SV=1	0,147336734	0,924913
P0CG39	POTE ankyrin domain family member J OS=Homo sapiens OX=9606 GN=POTEJ PE=3 SV=1	0,406264772	0,923839

Q14108	Lysosome membrane protein 2 OS=Homo sapiens OX=9606 GN=SCARB2 PE=1 SV=2	-0,117874438	0,923019
Q92947	Glutaryl-CoA dehydrogenase, mitochondrial OS=Homo sapiens OX=9606 GN=GCDH PE=1 SV=1	-0,273627401	0,921816
Q15070	Mitochondrial inner membrane protein OXA1L OS=Homo sapiens OX=9606 GN=OXA1L PE=1 SV=3	-0,404023457	0,919046
Q96F85	CB1 cannabinoid receptor-interacting protein 1 OS=Homo sapiens OX=9606 GN=CNRIP1 PE=1 SV=1	-0,192697449	0,918951
P30519	Heme oxygenase 2 OS=Homo sapiens OX=9606 GN=HMOX2 PE=1 SV=2	0,192556762	0,918174
Q14137	Ribosome biogenesis protein BOP1 OS=Homo sapiens OX=9606 GN=BOP1 PE=1 SV=2	0,221880164	0,917783
O43395	U4/U6 small nuclear ribonucleoprotein Prp3 OS=Homo sapiens OX=9606 GN=PRPF3 PE=1 SV=2	-0,272373287	0,91722
Q02252	Methylmalonate-semialdehyde dehydrogenase [acylating], mitochondrial OS=Homo sapiens OX=9606 GN=ALDH6A1 PE=1 SV=2	0,272367162	0,917198
P26368	Splicing factor U2AF 65 kDa subunit OS=Homo sapiens OX=9606 GN=U2AF2 PE=1 SV=4	-0,117187044	0,91656
P55010	Eukaryotic translation initiation factor 5 OS=Homo sapiens OX=9606 GN=EIF5 PE=1 SV=2	-0,117141206	0,91613
Q9UQE7	Structural maintenance of chromosomes protein 3 OS=Homo sapiens OX=9606 GN=SMC3 PE=1 SV=2	-0,172159566	0,915362
Q8IWF9	Coiled-coil domain-containing protein 83 OS=Homo sapiens OX=9606 GN=CCDC83 PE=2 SV=2	-0,6885822	0,914422
P28066	Proteasome subunit alpha type-5 OS=Homo sapiens OX=9606 GN=PSMA5 PE=1 SV=3	-0,122537421	0,914259
O43633	Charged multivesicular body protein 2a OS=Homo sapiens OX=9606 GN=CHMP2A PE=1 SV=1	-0,17191711	0,913851
P42345	Serine/threonine-protein kinase mTOR OS=Homo sapiens OX=9606 GN=MTOR PE=1 SV=1	0,191691911	0,913398
Q96NE9	FERM domain-containing protein 6 OS=Homo sapiens OX=9606 GN=FRMD6 PE=1 SV=1	-0,191628653	0,913049
P56377	AP-1 complex subunit sigma-2 OS=Homo sapiens OX=9606 GN=AP1S2 PE=1 SV=1	-0,366150084	0,912864
P04156	Major prion protein OS=Homo sapiens OX=9606 GN=PRNP PE=1 SV=1	0,271019794	0,912261
P05026	Sodium/potassium-transporting ATPase subunit beta-1 OS=Homo sapiens OX=9606 GN=ATP1B1 PE=1 SV=1	0,400111404	0,91066
P04844	Dolichyl-diphosphooligosaccharide--protein glycosyltransferase subunit 2 OS=Homo sapiens OX=9606 GN=RPN2 PE=1 SV=3	0,082845824	0,910591
O15355	Protein phosphatase 1G OS=Homo sapiens OX=9606 GN=PPM1G PE=1 SV=1	0,111655712	0,910373
Q8WVQ1	Soluble calcium-activated nucleotidase 1 OS=Homo sapiens OX=9606 GN=CANT1 PE=1 SV=1	-0,282709646	0,910366
Q08378	Golgin subfamily A member 3 OS=Homo sapiens OX=9606 GN=GOLGA3 PE=1 SV=2	-0,077737951	0,910126
Q8TB45	DEP domain-containing mTOR-interacting protein OS=Homo sapiens OX=9606 GN=DEPTOR PE=1 SV=2	-0,399169637	0,908638
P07602	Prosaposin OS=Homo sapiens OX=9606 GN=PSAP PE=1 SV=2	-0,107072551	0,907541
Q9BRP4	Proteasomal ATPase-associated factor 1 OS=Homo sapiens OX=9606 GN=PAAF1 PE=1 SV=2	-0,398278809	0,906723
P27144	Adenylate kinase 4, mitochondrial OS=Homo sapiens OX=9606 GN=AK4 PE=1 SV=1	0,156103829	0,90592
Q9NY65	#N/D	-0,134001944	0,905692
Q5K651	Sterile alpha motif domain-containing protein 9 OS=Homo sapiens OX=9606 GN=SAMD9 PE=1 SV=1	0,397711583	0,905504
P27707	Deoxycytidine kinase OS=Homo sapiens OX=9606 GN=DCK PE=1 SV=1	-0,268846754	0,9043
P60174	Triosephosphate isomerase OS=Homo sapiens OX=9606 GN=TPI1 PE=1 SV=3	-0,096279874	0,903084
Q16401	26S proteasome non-ATPase regulatory subunit 5 OS=Homo sapiens OX=9606 GN=PSMD5 PE=1 SV=3	0,096265885	0,902926
Q05048	Cleavage stimulation factor subunit 1 OS=Homo sapiens OX=9606 GN=CSTF1 PE=1 SV=1	0,39634508	0,902563
Q8NI27	THO complex subunit 2 OS=Homo sapiens OX=9606 GN=THOC2 PE=1 SV=2	0,218625237	0,902511
P53355	Death-associated protein kinase 1 OS=Homo sapiens OX=9606 GN=DAPK1 PE=1 SV=6	0,297154735	0,90227
O14976	Cyclin-G-associated kinase OS=Homo sapiens OX=9606 GN=GAK PE=1 SV=2	-0,21856994	0,902252
P42566	Epidermal growth factor receptor substrate 15 OS=Homo sapiens OX=9606 GN=EPS15 PE=1 SV=2	0,110797826	0,901981
Q9Y4E6	WD repeat-containing protein 7 OS=Homo sapiens OX=9606 GN=WDR7 PE=1 SV=2	-0,133573051	0,901393
Q15036	Sorting nexin-17 OS=Homo sapiens OX=9606 GN=SNX17 PE=1 SV=1	-0,155428752	0,901301
Q9H8M7	Ubiquitin carboxyl-terminal hydrolase MINDY-3 OS=Homo sapiens OX=9606 GN=MINDY3 PE=1 SV=1	0,395715471	0,901207
Q14151	Scaffold attachment factor B2 OS=Homo sapiens OX=9606 GN=SAFB2 PE=1 SV=1	0,39561871	0,900999
Q92615	La-related protein 4B OS=Homo sapiens OX=9606 GN=LARP4B PE=1 SV=3	0,189370372	0,900602
Q92793	CREB-binding protein OS=Homo sapiens OX=9606 GN=CREBBP PE=1 SV=3	-0,043253449	0,900286
Q08431	Lactadherin OS=Homo sapiens OX=9606 GN=MFGE8 PE=1 SV=3	-0,120853332	0,899243
O75569	Interferon-inducible double-stranded RNA-dependent protein kinase activator A OS=Homo sapiens OX=9606 GN=PRKRA PE=1 SV=1	-0,189055568	0,898869
Q13510	Acid ceramidase OS=Homo sapiens OX=9606 GN=ASAH1 PE=1 SV=5	-0,169467512	0,898621
P49137	MAP kinase-activated protein kinase 2 OS=Homo sapiens OX=9606 GN=MAPKAPK2 PE=1 SV=1	-0,169440321	0,898452
O14613	Cdc42 effector protein 2 OS=Homo sapiens OX=9606 GN=CDC42EP2 PE=1 SV=1	-0,394117686	0,897764
Q14103	Heterogeneous nuclear ribonucleoprotein D0 OS=Homo sapiens OX=9606 GN=HNRNPD PE=1 SV=1	-0,114890345	0,895067
P20645	Cation-dependent mannose-6-phosphate receptor OS=Homo sapiens OX=9606 GN=M6PR PE=1 SV=1	-0,217011295	0,894951
Q7Z5L9	Interferon regulatory factor 2-binding protein 2 OS=Homo sapiens OX=9606 GN=IRF2BP2 PE=1 SV=2	0,216981572	0,894812
Q9NYF8	Bcl-2-associated transcription factor 1 OS=Homo sapiens OX=9606 GN=BCLAF1 PE=1 SV=2	0,11482127	0,894423
P49189	4-trimethylaminobutyraldehyde dehydrogenase OS=Homo sapiens OX=9606 GN=ALDH9A1 PE=1 SV=3	-0,098581434	0,894264
Q9BZE4	Nucleolar GTP-binding protein 1 OS=Homo sapiens OX=9606 GN=GTPBP4 PE=1 SV=3	0,216841204	0,894155
Q5JRX3	Presequence protease, mitochondrial OS=Homo sapiens OX=9606 GN=PITRM1 PE=1 SV=3	-0,087719474	0,893982
Q15366	Poly(rC)-binding protein 2 OS=Homo sapiens OX=9606 GN=PCBP2 PE=1 SV=1	-0,114702899	0,893319
Q9NWZ3	Interleukin-1 receptor-associated kinase 4 OS=Homo sapiens OX=9606 GN=IRAK4 PE=1 SV=1	-0,391934115	0,893051
O95965	Integrin beta-like protein 1 OS=Homo sapiens OX=9606 GN=ITGBL1 PE=2 SV=1	0,142728528	0,890706
Q9P260	RAB11-binding protein RELCH OS=Homo sapiens OX=9606 GN=RELCH PE=1 SV=2	-0,153814187	0,890276
Q13464	Rho-associated protein kinase 1 OS=Homo sapiens OX=9606 GN=ROCK1 PE=1 SV=1	0,119838233	0,890223
P00403	Cytochrome c oxidase subunit 2 OS=Homo sapiens OX=9606 GN=MT-CO2 PE=1 SV=1	-0,153640579	0,889092
O43414	ERI1 exoribonuclease 3 OS=Homo sapiens OX=9606 GN=ERI3 PE=1 SV=2	-0,264592082	0,88872
O15040	Tectonin beta-propeller repeat-containing protein 2 OS=Homo sapiens OX=9606 GN=TECPR2 PE=1 SV=4	-0,389669886	0,888156
P42765	3-ketoacyl-CoA thiolase, mitochondrial OS=Homo sapiens OX=9606 GN=ACAA2 PE=1 SV=2	0,101399348	0,888104
Q86TM6	E3 ubiquitin-protein ligase synoviolin OS=Homo sapiens OX=9606 GN=SYVN1 PE=1 SV=2	-0,63888173	0,888009
P29728	2'-5'-oligoadenylate synthase 2 OS=Homo sapiens OX=9606 GN=OAS2 PE=1 SV=3	-0,187865526	0,887524
Q9NWV4	CXXC motif containing zinc binding protein OS=Homo sapiens OX=9606 GN=CZIB PE=1 SV=1	-0,186991158	0,887521
P28065	Proteasome subunit beta type-9 OS=Homo sapiens OX=9606 GN=PSMB9 PE=1 SV=2	-0,389094182	0,88691
Q99943	1-acyl-sn-glycerol-3-phosphate acyltransferase alpha OS=Homo sapiens OX=9606 GN=AGPAT1 PE=1 SV=2	-0,388954242	0,886607
Q13049	E3 ubiquitin-protein ligase TRIM32 OS=Homo sapiens OX=9606 GN=TRIM32 PE=1 SV=2	-0,186617188	0,885468
O43184	Disintegrin and metalloproteinase domain-containing protein 12 OS=Homo sapiens OX=9606 GN=ADAM12 PE=1 SV=3	0,388237112	0,885054
Q9BSK0	MARVEL domain-containing protein 1 OS=Homo sapiens OX=9606 GN=MARVELD1 PE=1 SV=1	-0,388185361	0,884942
Q6IA69	Glutamine-dependent NAD(+) synthetase OS=Homo sapiens OX=9606 GN=NADSYN1 PE=1 SV=3	1,178726365	0,884787
Q9UJX3	Anaphase-promoting complex subunit 7 OS=Homo sapiens OX=9606 GN=ANAPC7 PE=1 SV=4	-0,214822474	0,884712
A6NDG6	Glycerol-3-phosphate phosphatase OS=Homo sapiens OX=9606 GN=PGP PE=1 SV=1	0,167078647	0,88382
Q9BR76	Coronin-1B OS=Homo sapiens OX=9606 GN=CORO1B PE=1 SV=1	0,100982548	0,883722
Q567U6	Coiled-coil domain-containing protein 93 OS=Homo sapiens OX=9606 GN=CCDC93 PE=1 SV=2	0,214599799	0,883671
Q14692	Ribosome biogenesis protein BMS1 homolog OS=Homo sapiens OX=9606 GN=BMS1 PE=1 SV=1	0,252649784	0,882714
O75165	DnaJ homolog subfamily C member 13 OS=Homo sapiens OX=9606 GN=DNAJC13 PE=1 SV=5	-0,084562874	0,881842
P17050	Alpha-N-acetylgalactosaminidase OS=Homo sapiens OX=9606 GN=NAGA PE=1 SV=2	-0,386578264	0,881459
P30536	Translocator protein OS=Homo sapiens OX=9606 GN=TSPO PE=1 SV=3	-0,214031399	0,881015
P04818	Thymidylate synthase OS=Homo sapiens OX=9606 GN=TYMS PE=1 SV=3	0,214017511	0,88095
P04083	Annexin A1 OS=Homo sapiens OX=9606 GN=ANXA1 PE=1 SV=2	0,077202015	0,88077
P52888	Thimet oligopeptidase OS=Homo sapiens OX=9606 GN=THOP1 PE=1 SV=2	-0,10442214	0,880636
Q16836	Hydroxyacyl-coenzyme A dehydrogenase, mitochondrial OS=Homo sapiens OX=9606 GN=HADH PE=1 SV=3	0,152337924	0,880222
Q8NDT2	Putative RNA-binding protein 15B OS=Homo sapiens OX=9606 GN=RBM15B PE=1 SV=3	0,228718038	0,878353
Q04637	Eukaryotic translation initiation factor 4 gamma 1 OS=Homo sapiens OX=9606 GN=EIF4G1 PE=1 SV=4	0,057686231	0,878216
Q96BJ3	Axin interactor, dorsalization-associated protein OS=Homo sapiens OX=9606 GN=AIDA PE=1 SV=1	-0,185290714	0,878193
O43809	Cleavage and polyadenylation specificity factor subunit 5 OS=Homo sapiens OX=9606 GN=NUDT21 PE=1 SV=1	-0,152024133	0,878088
Q10570	Cleavage and polyadenylation specificity factor subunit 1 OS=Homo sapiens OX=9606 GN=CPSF1 PE=1 SV=2	-0,384750253	0,877492
Q16539	Mitogen-activated protein kinase 14 OS=Homo sapiens OX=9606 GN=MAPK14 PE=1 SV=3	-0,140852217	0,876866
Q9BY77	Polymerase delta-interacting protein 3 OS=Homo sapiens OX=9606 GN=POLDIP3 PE=1 SV=2	0,212819454	0,875355
O14807	Ras-related protein M-Ras OS=Homo sapiens OX=9606 GN=MRAS PE=1 SV=2	0,511375836	0,874638
P16083	Ribosyldihydronicotinamide dehydrogenase [quinone] OS=Homo sapiens OX=9606 GN=NQO2 PE=1 SV=5	-0,165548074	0,874364
Q712K3	Ubiquitin-conjugating enzyme E2 R2 OS=Homo sapiens OX=9606 GN=UBE2R2 PE=1 SV=1	-0,281860926	0,873994
P63313	Thymosin beta-10 OS=Homo sapiens OX=9606 GN=TMSB10 PE=1 SV=2	-0,38295099	0,873582
Q8N3R9	MAGUK p55 subfamily member 5 OS=Homo sapiens OX=9606 GN=MPP5 PE=1 SV=3	-0,382807584	0,87327
Q08209	Serine/threonine-protein phosphatase 2B catalytic subunit alpha isoform OS=Homo sapiens OX=9606 GN=PPP3CA PE=1 SV=1	0,184314509	0,872846

Q99459	Cell division cycle 5-like protein OS=Homo sapiens OX=9606 GN=CDC5L PE=1 SV=2	0,151068252	0,871596
Q0VDF9	Heat shock 70 kDa protein 14 OS=Homo sapiens OX=9606 GN=HSPA14 PE=1 SV=1	-0,259695185	0,870799
Q8NB49	Phospholipid-transporting ATPase IG OS=Homo sapiens OX=9606 GN=ATP11C PE=1 SV=3	0,211835171	0,870763
Q9Y646	Carboxypeptidase Q OS=Homo sapiens OX=9606 GN=CPQ PE=1 SV=1	-0,140002671	0,870617
P15374	Ubiquitin carboxyl-terminal hydrolase isozyme L3 OS=Homo sapiens OX=9606 GN=UCHL3 PE=1 SV=1	0,164825124	0,869905
O15111	Inhibitor of nuclear factor kappa-B kinase subunit alpha OS=Homo sapiens OX=9606 GN=CHUK PE=1 SV=2	-0,211105561	0,867361
Q8N3C0	Activating signal cointegrator 1 complex subunit 3 OS=Homo sapiens OX=9606 GN=ASCC3 PE=1 SV=3	-0,183298699	0,867289
Q14160	Protein scribble homolog OS=Homo sapiens OX=9606 GN=SCRIB PE=1 SV=4	-0,210918773	0,86649
P24539	ATP synthase F(0) complex subunit B1, mitochondrial OS=Homo sapiens OX=9606 GN=ATP5PB PE=1 SV=2	0,11164091	0,864891
Q9Y320	Thioredoxin-related transmembrane protein 2 OS=Homo sapiens OX=9606 GN=TMX2 PE=1 SV=1	0,149955898	0,864054
Q13769	THO complex subunit 5 homolog OS=Homo sapiens OX=9606 GN=THOC5 PE=1 SV=2	0,535941474	0,86289
O15260	Surfeit locus protein 4 OS=Homo sapiens OX=9606 GN=SURF4 PE=1 SV=3	0,182476711	0,862797
O75832	26S proteasome non-ATPase regulatory subunit 10 OS=Homo sapiens OX=9606 GN=PSMD10 PE=1 SV=1	0,130124432	0,862362
Q03135	Caveolin-1 OS=Homo sapiens OX=9606 GN=CAV1 PE=1 SV=4	0,13867806	0,860895
Q05682	Caldesmon OS=Homo sapiens OX=9606 GN=CALD1 PE=1 SV=3	-0,060969785	0,858037
Q9UNK0	Syntaxin-8 OS=Homo sapiens OX=9606 GN=STX8 PE=1 SV=2	-0,640788332	0,857089
P50542	Peroxisomal targeting signal 1 receptor OS=Homo sapiens OX=9606 GN=PEX5 PE=1 SV=3	-0,255697051	0,856178
P11441	Ubiquitin-like protein 4A OS=Homo sapiens OX=9606 GN=UBL4A PE=1 SV=1	-0,286059175	0,855723
Q99757	Thioredoxin, mitochondrial OS=Homo sapiens OX=9606 GN=TXN2 PE=1 SV=2	-0,208590879	0,855648
P11177	Pyruvate dehydrogenase E1 component subunit beta, mitochondrial OS=Homo sapiens OX=9606 GN=PDHB PE=1 SV=3	0,110589755	0,855188
P43246	DNA mismatch repair protein Msh2 OS=Homo sapiens OX=9606 GN=MSH2 PE=1 SV=1	-0,162391626	0,85493
Q10567	AP-1 complex subunit beta-1 OS=Homo sapiens OX=9606 GN=AP1B1 PE=1 SV=2	0,086744796	0,854207
P08651	Nuclear factor 1 C-type OS=Homo sapiens OX=9606 GN=NFIC PE=1 SV=2	0,373514596	0,852993
Q96HE7	ERO1-like protein alpha OS=Homo sapiens OX=9606 GN=ERO1A PE=1 SV=2	-0,105724212	0,852777
O75503	Ceroid-lipofuscinosis neuronal protein 5 OS=Homo sapiens OX=9606 GN=CLN5 PE=1 SV=2	-0,373385979	0,852712
Q9Y3P9	Rab GTPase-activating protein 1 OS=Homo sapiens OX=9606 GN=RABGAP1 PE=1 SV=3	0,110288321	0,852411
P42785	Lysosomal Pro-X carboxypeptidase OS=Homo sapiens OX=9606 GN=PRCP PE=1 SV=1	-0,180540184	0,85223
P42679	Megakaryocyte-associated tyrosine-protein kinase OS=Homo sapiens OX=9606 GN=MATK PE=1 SV=1	-0,372928334	0,851709
Q13409	Cytoplasmic dynein 1 intermediate chain 2 OS=Homo sapiens OX=9606 GN=DYNC1I2 PE=1 SV=3	0,105582209	0,85141
Q96B54	Zinc finger protein 428 OS=Homo sapiens OX=9606 GN=ZNF428 PE=1 SV=2	0,372772818	0,851369
P09525	Annexin A4 OS=Homo sapiens OX=9606 GN=ANXA4 PE=1 SV=4	0,080111738	0,850588
Q04726	Transducin-like enhancer protein 3 OS=Homo sapiens OX=9606 GN=TLE3 PE=1 SV=2	0,338957937	0,850547
Q7Z3B4	Nucleoporin p54 OS=Homo sapiens OX=9606 GN=NUP54 PE=1 SV=2	0,161677129	0,850544
P30039	Phenazine biosynthesis-like domain-containing protein OS=Homo sapiens OX=9606 GN=PBLD PE=1 SV=2	0,377251993	0,850429
Q9P0I2	ER membrane protein complex subunit 3 OS=Homo sapiens OX=9606 GN=EMC3 PE=1 SV=3	0,161520865	0,849585
P38570	Integrin alpha-E OS=Homo sapiens OX=9606 GN=ITGAE PE=1 SV=3	0,371793087	0,849222
Q9H6R4	Nucleolar protein 6 OS=Homo sapiens OX=9606 GN=NOL6 PE=1 SV=2	-0,371269281	0,848074
O95295	SNARE-associated protein Snapin OS=Homo sapiens OX=9606 GN=SNAPIN PE=1 SV=1	-0,20691426	0,847852
P21281	V-type proton ATPase subunit B, brain isoform OS=Homo sapiens OX=9606 GN=ATP6V1B2 PE=1 SV=3	0,094192482	0,846718
O43516	WAS/WASL-interacting protein family member 1 OS=Homo sapiens OX=9606 GN=WIPF1 PE=1 SV=3	0,370640987	0,846696
Q9Y5Q8	General transcription factor 3C polypeptide 5 OS=Homo sapiens OX=9606 GN=GTF3C5 PE=1 SV=2	-0,370542567	0,84648
Q8NBX0	Saccharopine dehydrogenase-like oxidoreductase OS=Homo sapiens OX=9606 GN=SCCPDH PE=1 SV=1	-0,128056964	0,846162
Q9NS00	Glycoprotein-N-acetylgalactosamine 3-beta-galactosyltransferase 1 OS=Homo sapiens OX=9606 GN=C1GALT1 PE=1 SV=1	0,206326184	0,845119
P62888	60S ribosomal protein L30 OS=Homo sapiens OX=9606 GN=RPL30 PE=1 SV=2	-0,160743181	0,844817
Q8TEQ6	Gem-associated protein 5 OS=Homo sapiens OX=9606 GN=GEMIN5 PE=1 SV=3	-0,206241662	0,844727
Q99973	Telomerase protein component 1 OS=Homo sapiens OX=9606 GN=TEP1 PE=1 SV=2	0,356765969	0,842952
Q9UKZ1	CCR4-NOT transcription complex subunit 11 OS=Homo sapiens OX=9606 GN=CNOT11 PE=1 SV=1	-0,152108909	0,842606
O43897	Tolloid-like protein 1 OS=Homo sapiens OX=9606 GN=TLL1 PE=1 SV=1	0,368661578	0,84235
Q01105	Protein SET OS=Homo sapiens OX=9606 GN=SET PE=1 SV=3	0,136120432	0,842195
O60343	TBC1 domain family member 4 OS=Homo sapiens OX=9606 GN=TBC1D4 PE=1 SV=2	-0,193572396	0,841638
P63241	Eukaryotic translation initiation factor 5A-1 OS=Homo sapiens OX=9606 GN=EIF5A PE=1 SV=2	-0,104535853	0,841359
Q14651	Plastin-1 OS=Homo sapiens OX=9606 GN=PLS1 PE=1 SV=2	0,178495286	0,841098
P42892	Endothelin-converting enzyme 1 OS=Homo sapiens OX=9606 GN=ECE1 PE=1 SV=2	-0,160120768	0,841005
Q9H1K6	Talin rod domain-containing protein 1 OS=Homo sapiens OX=9606 GN=TLNRD1 PE=1 SV=1	0,367350974	0,83947
Q6ZRR7	Leucine-rich repeat-containing protein 9 OS=Homo sapiens OX=9606 GN=LRRC9 PE=2 SV=2	0,367262758	0,839276
Q93063	Exostosin-2 OS=Homo sapiens OX=9606 GN=EXT2 PE=1 SV=1	0,367203291	0,839145
Q9NRZ7	1-acyl-sn-glycerol-3-phosphate acyltransferase gamma OS=Homo sapiens OX=9606 GN=AGPAT3 PE=1 SV=1	-0,367163298	0,839057
P46977	Dolichyl-diphosphooligosaccharide--protein glycosyltransferase subunit STT3A OS=Homo sapiens OX=9606 GN=STT3A PE=1 SV=2	0,093465586	0,838913
P16219	Short-chain specific acyl-CoA dehydrogenase, mitochondrial OS=Homo sapiens OX=9606 GN=ACADS PE=1 SV=1	-0,422391151	0,838715
P16278	Beta-galactosidase OS=Homo sapiens OX=9606 GN=GLB1 PE=1 SV=2	-0,159604168	0,837844
O15264	#N/D	-0,078083333	0,837786
P78356	Phosphatidylinositol 5-phosphate 4-kinase type-2 beta OS=Homo sapiens OX=9606 GN=PIP4K2B PE=1 SV=1	-0,110528819	0,837544
O14745	Na(+)/H(+) exchange regulatory cofactor NHE-RF1 OS=Homo sapiens OX=9606 GN=SLC9A3R1 PE=1 SV=4	0,159545345	0,837484
Q14320	Protein FAM50A OS=Homo sapiens OX=9606 GN=FAM50A PE=1 SV=2	0,250442634	0,836979
P46926	Glucosamine-6-phosphate isomerase 1 OS=Homo sapiens OX=9606 GN=GNPDA1 PE=1 SV=1	-0,135351613	0,836593
Q9Y2Z4	Tyrosine--tRNA ligase, mitochondrial OS=Homo sapiens OX=9606 GN=YARS2 PE=1 SV=2	-0,326037155	0,83405
Q709C8	Vacuolar protein sorting-associated protein 13C OS=Homo sapiens OX=9606 GN=VPS13C PE=1 SV=1	-0,203523546	0,832114
Q96GZ6	Solute carrier family 41 member 3 OS=Homo sapiens OX=9606 GN=SLC41A3 PE=1 SV=2	-0,258827319	0,831129
O14950	Myosin regulatory light chain 12B OS=Homo sapiens OX=9606 GN=MYL12B PE=1 SV=2	-0,248746098	0,830785
Q9BW83	Intraflagellar transport protein 27 homolog OS=Homo sapiens OX=9606 GN=IFT27 PE=1 SV=1	0,363398956	0,830768
P48357	Leptin receptor OS=Homo sapiens OX=9606 GN=LEPR PE=1 SV=2	0,373675187	0,830514
O95881	Thioredoxin domain-containing protein 12 OS=Homo sapiens OX=9606 GN=TXNDC12 PE=1 SV=1	-0,158385397	0,830396
Q9Y2W2	WW domain-binding protein 11 OS=Homo sapiens OX=9606 GN=WBP11 PE=1 SV=1	0,203073575	0,830029
Q9UNH6	Sorting nexin-7 OS=Homo sapiens OX=9606 GN=SNX7 PE=1 SV=1	0,212306107	0,82981
Q8TCZ2	CD99 antigen-like protein 2 OS=Homo sapiens OX=9606 GN=CD99L2 PE=1 SV=1	0,202955905	0,829484
P16220	Cyclic AMP-responsive element-binding protein 1 OS=Homo sapiens OX=9606 GN=CREB1 PE=1 SV=2	0,362394361	0,828553
Q8NEB9	Phosphatidylinositol 3-kinase catalytic subunit type 3 OS=Homo sapiens OX=9606 GN=PIK3C3 PE=1 SV=1	-0,157736353	0,826435
P31431	Syndecan-4 OS=Homo sapiens OX=9606 GN=SDC4 PE=1 SV=2	0,361193157	0,825901
Q8NHP6	Motile sperm domain-containing protein 2 OS=Homo sapiens OX=9606 GN=MOSPD2 PE=1 SV=1	0,201934279	0,824753
Q7Z2W4	Zinc finger CCCH-type antiviral protein 1 OS=Homo sapiens OX=9606 GN=ZC3HAV1 PE=1 SV=3	0,08919181	0,823939
Q9NPF4	Probable tRNA N6-adenosine threonylcarbamoyltransferase OS=Homo sapiens OX=9606 GN=OSGEP PE=1 SV=1	0,360122132	0,823535
Q01968	Inositol polyphosphate 5-phosphatase OCRL-1 OS=Homo sapiens OX=9606 GN=OCRL PE=1 SV=3	-0,175079683	0,822565
Q8IXJ6	NAD-dependent protein deacetylase sirtuin-2 OS=Homo sapiens OX=9606 GN=SIRT2 PE=1 SV=2	-0,359005348	0,821067
Q9Y3I1	F-box only protein 7 OS=Homo sapiens OX=9606 GN=FBXO7 PE=1 SV=1	0,35888491	0,8208
O43396	Thioredoxin-like protein 1 OS=Homo sapiens OX=9606 GN=TXNL1 PE=1 SV=3	0,094931575	0,820779
Q6IN85	Serine/threonine-protein phosphatase 4 regulatory subunit 3A OS=Homo sapiens OX=9606 GN=PPP4R3A PE=1 SV=1	0,358754765	0,820512
O14657	Torsin-1B OS=Homo sapiens OX=9606 GN=TOR1B PE=1 SV=2	-0,200943012	0,820165
P61764	Syntaxin-binding protein 1 OS=Homo sapiens OX=9606 GN=STXBP1 PE=1 SV=1	0,124652609	0,81965
Q6NUK1	Calcium-binding mitochondrial carrier protein SCaMC-1 OS=Homo sapiens OX=9606 GN=SLC25A24 PE=1 SV=2	-0,094801722	0,819442
Q08257	Quinone oxidoreductase OS=Homo sapiens OX=9606 GN=CRYZ PE=1 SV=1	-0,143316646	0,819348
P49589	Cysteine--tRNA ligase, cytoplasmic OS=Homo sapiens OX=9606 GN=CARS PE=1 SV=3	-0,064021205	0,818865
Q9P0R6	GSK3B-interacting protein OS=Homo sapiens OX=9606 GN=GSKIP PE=1 SV=2	-0,357958359	0,81875
Q92917	G-patch domain and KOW motifs-containing protein OS=Homo sapiens OX=9606 GN=GPKOW PE=1 SV=2	-0,245024853	0,817206
Q13637	Ras-related protein Rab-32 OS=Homo sapiens OX=9606 GN=RAB32 PE=1 SV=3	0,111428946	0,816418
Q9BPW8	Protein NipSnap homolog 1 OS=Homo sapiens OX=9606 GN=NIPSNAP1 PE=1 SV=1	0,244699074	0,816017
Q03393	6-pyruvoyl tetrahydrobiopterin synthase OS=Homo sapiens OX=9606 GN=PTS PE=1 SV=1	-0,356518528	0,815562
Q01995	Transgelin OS=Homo sapiens OX=9606 GN=TAGLN PE=1 SV=4	0,064823547	0,815165
Q92643	GPI-anchor transamidase OS=Homo sapiens OX=9606 GN=PIGK PE=1 SV=2	0,244278595	0,814484

Q96KP4	Cytosolic non-specific dipeptidase OS=Homo sapiens OX=9606 GN=CNDP2 PE=1 SV=2	0,094314476	0,81443
O75157	TSC22 domain family protein 2 OS=Homo sapiens OX=9606 GN=TSC22D2 PE=1 SV=3	0,355477614	0,813256
Q9NVI7	ATPase family AAA domain-containing protein 3A OS=Homo sapiens OX=9606 GN=ATAD3A PE=1 SV=2	-0,123754623	0,812692
Q9Y2L1	Exosome complex exonuclease RRP44 OS=Homo sapiens OX=9606 GN=DIS3 PE=1 SV=2	-0,17311223	0,811924
Q9H307	Pinin OS=Homo sapiens OX=9606 GN=PNN PE=1 SV=5	0,199147471	0,811866
Q6P1M0	Long-chain fatty acid transport protein 4 OS=Homo sapiens OX=9606 GN=SLC27A4 PE=1 SV=1	0,350191592	0,811675
O00567	Nucleolar protein 56 OS=Homo sapiens OX=9606 GN=NOP56 PE=1 SV=4	0,110859031	0,811475
O43854	EGF-like repeat and discoidin I-like domain-containing protein 3 OS=Homo sapiens OX=9606 GN=EDIL3 PE=1 SV=1	0,123358053	0,809623
Q9UBT7	Alpha-catulin OS=Homo sapiens OX=9606 GN=CTNNAL1 PE=1 SV=2	0,353801594	0,809538
Q9P212	1-phosphatidylinositol 4,5-bisphosphate phosphodiesterase epsilon-1 OS=Homo sapiens OX=9606 GN=PLCE1 PE=1 SV=3	-0,353760316	0,809447
P42330	Aldo-keto reductase family 1 member C3 OS=Homo sapiens OX=9606 GN=AKR1C3 PE=1 SV=4	0,154911513	0,809243
Q96B97	SH3 domain-containing kinase-binding protein 1 OS=Homo sapiens OX=9606 GN=SH3KBP1 PE=1 SV=2	0,101134841	0,808906
P33992	DNA replication licensing factor MCM5 OS=Homo sapiens OX=9606 GN=MCM5 PE=1 SV=5	0,172390946	0,808029
Q8TBX8	Phosphatidylinositol 5-phosphate 4-kinase type-2 gamma OS=Homo sapiens OX=9606 GN=PIP4K2C PE=1 SV=3	0,352419792	0,80647
Q9UBW8	COP9 signalosome complex subunit 7a OS=Homo sapiens OX=9606 GN=COPS7A PE=1 SV=1	-0,131195202	0,80646
O75976	Carboxypeptidase D OS=Homo sapiens OX=9606 GN=CPD PE=1 SV=2	0,352254978	0,806104
O43301	Heat shock 70 kDa protein 12A OS=Homo sapiens OX=9606 GN=HSPA12A PE=1 SV=2	-0,209971076	0,805662
O43815	Striatin OS=Homo sapiens OX=9606 GN=STRN PE=1 SV=4	0,197664631	0,805021
Q12765	Secernin-1 OS=Homo sapiens OX=9606 GN=SCRN1 PE=1 SV=2	-0,100672413	0,804519
Q96QG7	Myotubularin-related protein 9 OS=Homo sapiens OX=9606 GN=MTMR9 PE=1 SV=1	-0,35139946	0,804203
P27635	60S ribosomal protein L10 OS=Homo sapiens OX=9606 GN=RPL10 PE=1 SV=4	0,100618939	0,804012
Q99829	Copine-1 OS=Homo sapiens OX=9606 GN=CPNE1 PE=1 SV=1	0,104901958	0,803182
O94813	Slit homolog 2 protein OS=Homo sapiens OX=9606 GN=SLIT2 PE=1 SV=1	-0,350158208	0,801443
P48454	Serine/threonine-protein phosphatase 2B catalytic subunit gamma isoform OS=Homo sapiens OX=9606 GN=PPP3CC PE=1 SV=3	-0,325831044	0,800793
Q02878	60S ribosomal protein L6 OS=Homo sapiens OX=9606 GN=RPL6 PE=1 SV=3	0,089853101	0,800411
Q9BYD3	39S ribosomal protein L4, mitochondrial OS=Homo sapiens OX=9606 GN=MRPL4 PE=1 SV=1	-0,349603897	0,80021
Q8NEY1	Neuron navigator 1 OS=Homo sapiens OX=9606 GN=NAV1 PE=1 SV=2	0,122134441	0,800173
Q8N8Z6	Discoidin, CUB and LCCL domain-containing protein 1 OS=Homo sapiens OX=9606 GN=DCBLD1 PE=1 SV=2	0,223301601	0,799385
Q99798	Aconitate hydratase, mitochondrial OS=Homo sapiens OX=9606 GN=ACO2 PE=1 SV=2	0,069754875	0,798909
Q8TDQ7	Glucosamine-6-phosphate isomerase 2 OS=Homo sapiens OX=9606 GN=GNPDA2 PE=1 SV=1	0,239997554	0,79888
Q16563	Synaptophysin-like protein 1 OS=Homo sapiens OX=9606 GN=SYPL1 PE=1 SV=1	0,196306216	0,798757
P15121	Aldo-keto reductase family 1 member B1 OS=Homo sapiens OX=9606 GN=AKR1B1 PE=1 SV=3	0,09621025	0,798679
Q96SB3	Neurabin-2 OS=Homo sapiens OX=9606 GN=PPP1R9B PE=1 SV=3	-0,153038473	0,797885
Q562R1	Beta-actin-like protein 2 OS=Homo sapiens OX=9606 GN=ACTBL2 PE=1 SV=2	-0,196066661	0,797654
P35232	Prohibitin OS=Homo sapiens OX=9606 GN=PHB PE=1 SV=1	-0,092618029	0,797044
Q13610	Periodic tryptophan protein 1 homolog OS=Homo sapiens OX=9606 GN=PWP1 PE=1 SV=1	-0,152853291	0,796764
P52434	DNA-directed RNA polymerases I, II, and III subunit RPABC3 OS=Homo sapiens OX=9606 GN=POLR2H PE=1 SV=4	-0,348044684	0,796738
Q0VGL1	Ragulator complex protein LAMTOR4 OS=Homo sapiens OX=9606 GN=LAMTOR4 PE=1 SV=1	0,347808458	0,796212
O75886	Signal transducing adapter molecule 2 OS=Homo sapiens OX=9606 GN=STAM2 PE=1 SV=1	-0,12160399	0,796084
Q7KZ85	Transcription elongation factor SPT6 OS=Homo sapiens OX=9606 GN=SUPT6H PE=1 SV=2	0,239053272	0,79544
Q8TCD5	5'(3')-deoxyribonucleotidase, cytosolic type OS=Homo sapiens OX=9606 GN=NT5C PE=1 SV=2	-0,239048176	0,795422
O15013	Rho guanine nucleotide exchange factor 10 OS=Homo sapiens OX=9606 GN=ARHGEF10 PE=1 SV=4	-0,169992296	0,795102
P61769	Beta-2-microglobulin OS=Homo sapiens OX=9606 GN=B2M PE=1 SV=1	-0,238831548	0,794633
Q5T6F2	Ubiquitin-associated protein 2 OS=Homo sapiens OX=9606 GN=UBAP2 PE=1 SV=1	0,238627472	0,79389
Q8NC96	Adaptin ear-binding coat-associated protein 1 OS=Homo sapiens OX=9606 GN=NECAP1 PE=1 SV=2	0,188334174	0,793577
P62979	Ubiquitin-40S ribosomal protein S27a OS=Homo sapiens OX=9606 GN=RPS27A PE=1 SV=2	-0,139452337	0,793575
P11387	DNA topoisomerase 1 OS=Homo sapiens OX=9606 GN=TOP1 PE=1 SV=2	0,099483958	0,793273
P04049	RAF proto-oncogene serine/threonine-protein kinase OS=Homo sapiens OX=9606 GN=RAF1 PE=1 SV=1	0,169566638	0,792811
Q14156	Protein EFR3 homolog A OS=Homo sapiens OX=9606 GN=EFR3A PE=1 SV=2	0,346052411	0,792297
Q9UMZ2	Synergin gamma OS=Homo sapiens OX=9606 GN=SYNRG PE=1 SV=2	0,442273113	0,790966
Q15459	Splicing factor 3A subunit 1 OS=Homo sapiens OX=9606 GN=SF3A1 PE=1 SV=1	-0,088887303	0,790199
Q01082	Spectrin beta chain, non-erythrocytic 1 OS=Homo sapiens OX=9606 GN=SPTBN1 PE=1 SV=2	-0,031564081	0,789838
Q9HA64	Ketosamine-3-kinase OS=Homo sapiens OX=9606 GN=FN3KRP PE=1 SV=2	-0,34462051	0,789102
A6NCE7	Microtubule-associated proteins 1A/1B light chain 3 beta 2 OS=Homo sapiens OX=9606 GN=MAP1LC3B2 PE=2 SV=1	0,181023572	0,789092
P40616	ADP-ribosylation factor-like protein 1 OS=Homo sapiens OX=9606 GN=ARL1 PE=1 SV=1	-0,138744167	0,788871
Q8NB90	ATPase family protein 2 homolog OS=Homo sapiens OX=9606 GN=SPATA5 PE=1 SV=3	-0,237097173	0,788318
Q9Y608	Leucine-rich repeat flightless-interacting protein 2 OS=Homo sapiens OX=9606 GN=LRRFIP2 PE=1 SV=1	0,1384936	0,787209
P49585	Choline-phosphate cytidylyltransferase A OS=Homo sapiens OX=9606 GN=PCYT1A PE=1 SV=2	-0,102918852	0,785248
P62241	40S ribosomal protein S8 OS=Homo sapiens OX=9606 GN=RPS8 PE=1 SV=2	-0,113438615	0,78474
Q86VS8	Protein Hook homolog 3 OS=Homo sapiens OX=9606 GN=HOOK3 PE=1 SV=2	0,091346716	0,784081
P41208	Centrin-2 OS=Homo sapiens OX=9606 GN=CETN2 PE=1 SV=1	0,235803371	0,78361
Q9H3Z4	DnaJ homolog subfamily C member 5 OS=Homo sapiens OX=9606 GN=DNAJC5 PE=1 SV=1	0,447273908	0,782965
P48058	Glutamate receptor 4 OS=Homo sapiens OX=9606 GN=GRIA4 PE=1 SV=2	0,234956387	0,780528
Q96J01	THO complex subunit 3 OS=Homo sapiens OX=9606 GN=THOC3 PE=1 SV=1	0,234949749	0,780504
P61803	Dolichyl-diphosphooligosaccharide--protein glycosyltransferase subunit DAD1 OS=Homo sapiens OX=9606 GN=DAD1 PE=1 SV=3	-0,192022746	0,779054
O15371	Eukaryotic translation initiation factor 3 subunit D OS=Homo sapiens OX=9606 GN=EIF3D PE=1 SV=1	-0,090794429	0,778466
Q53FV1	ORM1-like protein 2 OS=Homo sapiens OX=9606 GN=ORMDL2 PE=1 SV=2	0,124216942	0,778234
Q99417	c-Myc-binding protein OS=Homo sapiens OX=9606 GN=MYCBP PE=1 SV=3	0,339606784	0,77789
Q10469	Alpha-1,6-mannosyl-glycoprotein 2-beta-N-acetylglucosaminyltransferase OS=Homo sapiens OX=9606 GN=MGAT2 PE=1 SV=1	0,339037103	0,776614
Q9BZH6	WD repeat-containing protein 11 OS=Homo sapiens OX=9606 GN=WDR11 PE=1 SV=1	-0,14937248	0,775751
Q96C36	Pyrroline-5-carboxylate reductase 2 OS=Homo sapiens OX=9606 GN=PYCR2 PE=1 SV=1	-0,136741928	0,775607
Q495W5	Alpha-(1,3)-fucosyltransferase 11 OS=Homo sapiens OX=9606 GN=FUT11 PE=1 SV=1	0,338518952	0,775453
Q9NWM8	Peptidyl-prolyl cis-trans isomerase FKBP14 OS=Homo sapiens OX=9606 GN=FKBP14 PE=1 SV=1	0,149283011	0,775213
Q9HB90	Ras-related GTP-binding protein C OS=Homo sapiens OX=9606 GN=RRAGC PE=1 SV=1	-0,16625295	0,775026
Q96HY6	DDRGK domain-containing protein 1 OS=Homo sapiens OX=9606 GN=DDRGK1 PE=1 SV=2	0,166105414	0,774236
Q9Y2B0	Protein canopy homolog 2 OS=Homo sapiens OX=9606 GN=CNPY2 PE=1 SV=1	0,126684082	0,77405
Q9NVP1	ATP-dependent RNA helicase DDX18 OS=Homo sapiens OX=9606 GN=DDX18 PE=1 SV=2	-0,190910486	0,773949
P55039	Developmentally-regulated GTP-binding protein 2 OS=Homo sapiens OX=9606 GN=DRG2 PE=1 SV=1	0,14871046	0,771768
Q9GZU8	PSME3-interacting protein OS=Homo sapiens OX=9606 GN=FAM192A PE=1 SV=1	-0,336769552	0,77153
O00423	Echinoderm microtubule-associated protein-like 1 OS=Homo sapiens OX=9606 GN=EML1 PE=1 SV=3	0,232420186	0,771306
O95183	Vesicle-associated membrane protein 5 OS=Homo sapiens OX=9606 GN=VAMP5 PE=1 SV=1	0,336656399	0,771276
P10244	Myb-related protein B OS=Homo sapiens OX=9606 GN=MYBL2 PE=1 SV=1	0,612896447	0,771173
Q71RC2	La-related protein 4 OS=Homo sapiens OX=9606 GN=LARP4 PE=1 SV=3	-0,232069505	0,770031
Q2NKX8	DNA excision repair protein ERCC-6-like OS=Homo sapiens OX=9606 GN=ERCC6L PE=1 SV=1	0,336067457	0,769954
O94906	Pre-mRNA-processing factor 6 OS=Homo sapiens OX=9606 GN=PRPF6 PE=1 SV=1	-0,148406607	0,769941
Q5TFE4	5'-nucleotidase domain-containing protein 1 OS=Homo sapiens OX=9606 GN=NT5DC1 PE=1 SV=1	0,232038362	0,769918
Q86VY4	Testis-specific Y-encoded-like protein 5 OS=Homo sapiens OX=9606 GN=TSPYL5 PE=1 SV=2	0,124846706	0,768136
Q9NWY4	Histone PARylation factor 1 OS=Homo sapiens OX=9606 GN=HPF1 PE=1 SV=2	0,278214505	0,767983
Q15287	RNA-binding protein with serine-rich domain 1 OS=Homo sapiens OX=9606 GN=RNPS1 PE=1 SV=1	-0,231334889	0,767361
Q9H3M7	Thioredoxin-interacting protein OS=Homo sapiens OX=9606 GN=TXNIP PE=1 SV=1	0,334583287	0,766621
Q9NZN5	Rho guanine nucleotide exchange factor 12 OS=Homo sapiens OX=9606 GN=ARHGEF12 PE=1 SV=1	0,334551437	0,766549
O75382	Tripartite motif-containing protein 3 OS=Homo sapiens OX=9606 GN=TRIM3 PE=1 SV=2	0,189276256	0,766458
Q9H832	Ubiquitin-conjugating enzyme E2 Z OS=Homo sapiens OX=9606 GN=UBE2Z PE=1 SV=2	0,125405461	0,76492
Q5VIR6	Vacuolar protein sorting-associated protein 53 homolog OS=Homo sapiens OX=9606 GN=VPS53 PE=1 SV=1	0,164312531	0,764646
Q53T59	HCLS1-binding protein 3 OS=Homo sapiens OX=9606 GN=HS1BP3 PE=1 SV=1	-0,876415499	0,764492
Q9H4M3	F-box only protein 44 OS=Homo sapiens OX=9606 GN=FBXO44 PE=1 SV=3	0,182275311	0,764416
Q9Y287	Integral membrane protein 2B OS=Homo sapiens OX=9606 GN=ITM2B PE=1 SV=1	-0,333002262	0,763067
Q9P0L0	Vesicle-associated membrane protein-associated protein A OS=Homo sapiens OX=9606 GN=VAPA PE=1 SV=3	0,10038269	0,762463

Q9C037	E3 ubiquitin-protein ligase TRIM4 OS=Homo sapiens OX=9606 GN=TRIM4 PE=1 SV=2	0,229962461	0,762375
Q9H2K8	Serine/threonine-protein kinase TAO3 OS=Homo sapiens OX=9606 GN=TAOK3 PE=1 SV=2	0,332379783	0,761667
Q9UBY9	Heat shock protein beta-7 OS=Homo sapiens OX=9606 GN=HSPB7 PE=1 SV=1	-0,105062247	0,761635
Q9HB07	UPF0160 protein MYG1, mitochondrial OS=Homo sapiens OX=9606 GN=C12orf10 PE=1 SV=2	-0,163497357	0,760293
Q13242	Serine/arginine-rich splicing factor 9 OS=Homo sapiens OX=9606 GN=SRSF9 PE=1 SV=1	0,124720324	0,760038
P48059	LIM and senescent cell antigen-like-containing domain protein 1 OS=Homo sapiens OX=9606 GN=LIMS1 PE=1 SV=4	-0,095885507	0,759472
Q14684	Ribosomal RNA processing protein 1 homolog B OS=Homo sapiens OX=9606 GN=RRP1B PE=1 SV=3	-0,064104566	0,759044
P68371	Tubulin beta-4B chain OS=Homo sapiens OX=9606 GN=TUBB4B PE=1 SV=1	-0,053081144	0,758989
P53999	Activated RNA polymerase II transcriptional coactivator p15 OS=Homo sapiens OX=9606 GN=SUB1 PE=1 SV=3	-0,163060562	0,757962
O75439	Mitochondrial-processing peptidase subunit beta OS=Homo sapiens OX=9606 GN=PMPCB PE=1 SV=2	-0,095711056	0,757843
P12270	Nucleoprotein TPR OS=Homo sapiens OX=9606 GN=TPR PE=1 SV=3	0,068023239	0,756983
Q9UHL4	Dipeptidyl peptidase 2 OS=Homo sapiens OX=9606 GN=DPP7 PE=1 SV=3	-0,228461662	0,756925
P04062	Lysosomal acid glucosylceramidase OS=Homo sapiens OX=9606 GN=GBA PE=1 SV=3	-0,116461954	0,756712
O14737	Programmed cell death protein 5 OS=Homo sapiens OX=9606 GN=PDCD5 PE=1 SV=3	0,14602286	0,755641
P07741	Adenine phosphoribosyltransferase OS=Homo sapiens OX=9606 GN=APRT PE=1 SV=2	-0,104299482	0,755136
Q9H2H8	Peptidyl-prolyl cis-trans isomerase-like 3 OS=Homo sapiens OX=9606 GN=PPIL3 PE=1 SV=1	-0,22794328	0,755044
Q16891	MICOS complex subunit MIC60 OS=Homo sapiens OX=9606 GN=IMMT PE=1 SV=1	-0,069224561	0,753855
Q5R3I4	Tetratricopeptide repeat protein 38 OS=Homo sapiens OX=9606 GN=TTC38 PE=1 SV=1	-0,184219479	0,753236
Q9H5N1	Rab GTPase-binding effector protein 2 OS=Homo sapiens OX=9606 GN=RABEP2 PE=1 SV=2	-0,18630711	0,752875
Q8TBC4	NEDD8-activating enzyme E1 catalytic subunit OS=Homo sapiens OX=9606 GN=UBA3 PE=1 SV=2	-0,123581097	0,751937
Q8WYL5	Protein phosphatase Slingshot homolog 1 OS=Homo sapiens OX=9606 GN=SSH1 PE=1 SV=2	0,456398402	0,750424
Q9Y371	Endophilin-B1 OS=Homo sapiens OX=9606 GN=SH3GLB1 PE=1 SV=1	-0,123311439	0,750022
Q8WXA3	RUN and FYVE domain-containing protein 2 OS=Homo sapiens OX=9606 GN=RUFY2 PE=1 SV=3	-0,327188383	0,749968
P61254	60S ribosomal protein L26 OS=Homo sapiens OX=9606 GN=RPL26 PE=1 SV=1	-0,123302268	0,749957
Q86V21	Acetoacetyl-CoA synthetase OS=Homo sapiens OX=9606 GN=AACS PE=1 SV=1	-0,092557287	0,749427
Q7LBR1	Charged multivesicular body protein 1b OS=Homo sapiens OX=9606 GN=CHMP1B PE=1 SV=1	0,185525335	0,749304
P12111	Collagen alpha-3(VI) chain OS=Homo sapiens OX=9606 GN=COL6A3 PE=1 SV=5	-0,030972645	0,749156
A6NHL2	Tubulin alpha chain-like 3 OS=Homo sapiens OX=9606 GN=TUBAL3 PE=1 SV=2	-0,123179427	0,749085
Q6ZT12	E3 ubiquitin-protein ligase UBR3 OS=Homo sapiens OX=9606 GN=UBR3 PE=2 SV=2	0,342327216	0,748976
O95573	Long-chain-fatty-acid--CoA ligase 3 OS=Homo sapiens OX=9606 GN=ACSL3 PE=1 SV=3	0,094744076	0,748828
Q4G0F5	Vacuolar protein sorting-associated protein 26B OS=Homo sapiens OX=9606 GN=VPS26B PE=1 SV=2	-0,13242879	0,747204
Q9HAT2	Sialate O-acetylesterase OS=Homo sapiens OX=9606 GN=SIAE PE=1 SV=1	-0,225778364	0,747188
P07711	Cathepsin L1 OS=Homo sapiens OX=9606 GN=CTSL PE=1 SV=2	-0,184769787	0,745856
Q9Y496	Kinesin-like protein KIF3A OS=Homo sapiens OX=9606 GN=KIF3A PE=1 SV=4	0,325088373	0,745226
O43318	Mitogen-activated protein kinase kinase kinase 7 OS=Homo sapiens OX=9606 GN=MAP3K7 PE=1 SV=1	0,419394391	0,744228
Q9NW13	RNA-binding protein 28 OS=Homo sapiens OX=9606 GN=RBM28 PE=1 SV=3	0,248282198	0,743995
P60983	Glia maturation factor beta OS=Homo sapiens OX=9606 GN=GMFB PE=1 SV=2	0,160320281	0,743371
Q8N8A2	Serine/threonine-protein phosphatase 6 regulatory ankyrin repeat subunit B OS=Homo sapiens OX=9606 GN=ANKRD44 PE=1 SV=3	-0,117267218	0,742716
Q9UKY7	Protein CDV3 homolog OS=Homo sapiens OX=9606 GN=CDV3 PE=1 SV=1	0,102541244	0,740206
Q86Y07	Serine/threonine-protein kinase VRK2 OS=Homo sapiens OX=9606 GN=VRK2 PE=1 SV=3	-0,173243148	0,738876
Q9UJS0	Calcium-binding mitochondrial carrier protein Aralar2 OS=Homo sapiens OX=9606 GN=SLC25A13 PE=1 SV=2	0,183195031	0,738677
P80217	Interferon-induced 35 kDa protein OS=Homo sapiens OX=9606 GN=IFI35 PE=1 SV=5	-0,808435368	0,738428
Q5T0F9	Coiled-coil and C2 domain-containing protein 1B OS=Homo sapiens OX=9606 GN=CC2D1B PE=1 SV=1	-0,223096457	0,737465
O43823	A-kinase anchor protein 8 OS=Homo sapiens OX=9606 GN=AKAP8 PE=1 SV=1	-0,222812463	0,736436
Q9H2V7	Protein spinster homolog 1 OS=Homo sapiens OX=9606 GN=SPNS1 PE=1 SV=1	0,603898133	0,735446
O00422	Histone deacetylase complex subunit SAP18 OS=Homo sapiens OX=9606 GN=SAP18 PE=1 SV=1	0,222431794	0,735057
Q13907	Isopentenyl-diphosphate Delta-isomerase 1 OS=Homo sapiens OX=9606 GN=IDI1 PE=1 SV=2	-0,18238137	0,734971
P29373	Cellular retinoic acid-binding protein 2 OS=Homo sapiens OX=9606 GN=CRABP2 PE=1 SV=2	-0,142276408	0,733278
Q9P2X3	Protein IMPACT OS=Homo sapiens OX=9606 GN=IMPACT PE=1 SV=2	-0,319793207	0,733242
Q9NX24	H/ACA ribonucleoprotein complex subunit 2 OS=Homo sapiens OX=9606 GN=NHP2 PE=1 SV=1	0,221668586	0,732292
P45880	Voltage-dependent anion-selective channel protein 2 OS=Homo sapiens OX=9606 GN=VDAC2 PE=1 SV=2	-0,101567213	0,731967
O60313	Dynamin-like 120 kDa protein, mitochondrial OS=Homo sapiens OX=9606 GN=OPA1 PE=1 SV=3	0,113170166	0,731758
P20929	Nebulin OS=Homo sapiens OX=9606 GN=NEB PE=1 SV=5	-0,226874976	0,731749
Q9Y305	Acyl-coenzyme A thioesterase 9, mitochondrial OS=Homo sapiens OX=9606 GN=ACOT9 PE=1 SV=2	0,101514448	0,731522
Q8NC56	LEM domain-containing protein 2 OS=Homo sapiens OX=9606 GN=LEMD2 PE=1 SV=1	-0,157712119	0,729532
Q9H1A4	Anaphase-promoting complex subunit 1 OS=Homo sapiens OX=9606 GN=ANAPC1 PE=1 SV=1	-0,181083892	0,729068
P58215	Lysyl oxidase homolog 3 OS=Homo sapiens OX=9606 GN=LOXL3 PE=1 SV=1	0,220720499	0,728859
O60565	Gremlin-1 OS=Homo sapiens OX=9606 GN=GREM1 PE=1 SV=1	-0,352298205	0,728696
P26885	Peptidyl-prolyl cis-trans isomerase FKBP2 OS=Homo sapiens OX=9606 GN=FKBP2 PE=1 SV=2	0,157361989	0,727678
Q9UIC8	Leucine carboxyl methyltransferase 1 OS=Homo sapiens OX=9606 GN=LCMT1 PE=1 SV=2	-0,317333283	0,727662
P62917	60S ribosomal protein L8 OS=Homo sapiens OX=9606 GN=RPL8 PE=1 SV=2	-0,088872559	0,727252
Q96T37	RNA-binding protein 15 OS=Homo sapiens OX=9606 GN=RBM15 PE=1 SV=2	0,316825574	0,726509
Q92626	Peroxidasin homolog OS=Homo sapiens OX=9606 GN=PXDN PE=1 SV=2	-0,11237683	0,725774
O00273	DNA fragmentation factor subunit alpha OS=Homo sapiens OX=9606 GN=DFFA PE=1 SV=1	0,21962793	0,724904
O15037	Protein KHNYN OS=Homo sapiens OX=9606 GN=KHNYN PE=1 SV=3	-0,384773596	0,724476
O15085	Rho guanine nucleotide exchange factor 11 OS=Homo sapiens OX=9606 GN=ARHGEF11 PE=1 SV=1	-0,156510654	0,724313
Q9UKM9	RNA-binding protein Raly OS=Homo sapiens OX=9606 GN=RALY PE=1 SV=1	0,128910863	0,72421
P48735	Isocitrate dehydrogenase [NADP], mitochondrial OS=Homo sapiens OX=9606 GN=IDH2 PE=1 SV=2	-0,092031852	0,72369
Q5T7N3	KN motif and ankyrin repeat domain-containing protein 4 OS=Homo sapiens OX=9606 GN=KANK4 PE=1 SV=1	0,315281584	0,723002
Q86SR1	Polypeptide N-acetylgalactosaminyltransferase 10 OS=Homo sapiens OX=9606 GN=GALNT10 PE=1 SV=2	0,128679457	0,722703
P43304	Glycerol-3-phosphate dehydrogenase, mitochondrial OS=Homo sapiens OX=9606 GN=GPD2 PE=1 SV=3	-0,068260742	0,722489
Q13867	Bleomycin hydrolase OS=Homo sapiens OX=9606 GN=BLMH PE=1 SV=1	-0,140375046	0,721981
P80404	4-aminobutyrate aminotransferase, mitochondrial OS=Homo sapiens OX=9606 GN=ABAT PE=1 SV=3	0,218787094	0,721861
Q9H0U3	Magnesium transporter protein 1 OS=Homo sapiens OX=9606 GN=MAGT1 PE=1 SV=1	0,218660699	0,721404
Q13641	Trophoblast glycoprotein OS=Homo sapiens OX=9606 GN=TPBG PE=1 SV=1	-0,140231769	0,721132
Q9NWV8	BRISC and BRCA1-A complex member 1 OS=Homo sapiens OX=9606 GN=BABAM1 PE=1 SV=1	-0,218529704	0,72093
Q14203	Dynactin subunit 1 OS=Homo sapiens OX=9606 GN=DCTN1 PE=1 SV=3	-0,047797455	0,720446
Q9BZQ8	Protein Niban OS=Homo sapiens OX=9606 GN=FAM129A PE=1 SV=1	-0,13998301	0,719657
P28074	Proteasome subunit beta type-5 OS=Homo sapiens OX=9606 GN=PSMB5 PE=1 SV=3	-0,095514522	0,719201
Q14690	Protein RRP5 homolog OS=Homo sapiens OX=9606 GN=PDCD11 PE=1 SV=3	-0,161695056	0,719113
Q96EK6	Glucosamine 6-phosphate N-acetyltransferase OS=Homo sapiens OX=9606 GN=GNPNAT1 PE=1 SV=1	-0,118839079	0,718428
P78316	Nucleolar protein 14 OS=Homo sapiens OX=9606 GN=NOP14 PE=1 SV=3	0,28006929	0,715123
Q9H936	Mitochondrial glutamate carrier 1 OS=Homo sapiens OX=9606 GN=SLC25A22 PE=1 SV=1	-0,177961646	0,714893
Q9HD20	Manganese-transporting ATPase 13A1 OS=Homo sapiens OX=9606 GN=ATP13A1 PE=1 SV=2	-0,081641776	0,7147
P49184	Deoxyribonuclease-1-like 1 OS=Homo sapiens OX=9606 GN=DNASE1L1 PE=1 SV=1	-0,311369634	0,714102
Q9UID3	Vacuolar protein sorting-associated protein 51 homolog OS=Homo sapiens OX=9606 GN=VPS51 PE=1 SV=2	-0,177757867	0,713969
P04745	Alpha-amylase 1 OS=Homo sapiens OX=9606 GN=AMY1A PE=1 SV=2	-0,348047865	0,713619
Q9UIG0	Tyrosine-protein kinase BAZ1B OS=Homo sapiens OX=9606 GN=BAZ1B PE=1 SV=2	-0,310551997	0,71224
P06899	Histone H2B type 1-J OS=Homo sapiens OX=9606 GN=HIST1H2BJ PE=1 SV=3	1,77219416	0,711946
O00165	HCLS1-associated protein X-1 OS=Homo sapiens OX=9606 GN=HAX1 PE=1 SV=2	-0,164960276	0,710919
Q9BZQ6	ER degradation-enhancing alpha-mannosidase-like protein 3 OS=Homo sapiens OX=9606 GN=EDEM3 PE=1 SV=2	-0,176932997	0,710232
P05161	Ubiquitin-like protein ISG15 OS=Homo sapiens OX=9606 GN=ISG15 PE=1 SV=5	0,176683521	0,709102
Q93009	Ubiquitin carboxyl-terminal hydrolase 7 OS=Homo sapiens OX=9606 GN=USP7 PE=1 SV=2	0,078549236	0,708865
Q9Y5R8	Trafficking protein particle complex subunit 1 OS=Homo sapiens OX=9606 GN=TRAPPC1 PE=1 SV=1	-0,308568518	0,707718
O15258	Protein RER1 OS=Homo sapiens OX=9606 GN=RER1 PE=1 SV=1	0,126342943	0,707525
Q9UBS8	E3 ubiquitin-protein ligase RNF14 OS=Homo sapiens OX=9606 GN=RNF14 PE=1 SV=1	-0,153520838	0,707395
Q9UBV8	Peflin OS=Homo sapiens OX=9606 GN=PEF1 PE=1 SV=1	-0,214762994	0,707314
Q96EL3	39S ribosomal protein L53, mitochondrial OS=Homo sapiens OX=9606 GN=MRPL53 PE=1 SV=1	0,569947465	0,705804

Q9Y6W3	Calpain-7 OS=Homo sapiens OX=9606 GN=CAPN7 PE=1 SV=1	-0,21843731	0,705075
O95219	Sorting nexin-4 OS=Homo sapiens OX=9606 GN=SNX4 PE=1 SV=1	0,175759073	0,704918
Q96FJ2	Dynein light chain 2, cytoplasmic OS=Homo sapiens OX=9606 GN=DYNLL2 PE=1 SV=1	-0,137478199	0,70484
O75251	NADH dehydrogenase [ubiquinone] iron-sulfur protein 7, mitochondrial OS=Homo sapiens OX=9606 GN=NDUFS7 PE=1 SV=3	0,307255734	0,704723
Q8NHU6	Tudor domain-containing protein 7 OS=Homo sapiens OX=9606 GN=TDRD7 PE=1 SV=2	-1,152514642	0,70371
Q96CW5	Gamma-tubulin complex component 3 OS=Homo sapiens OX=9606 GN=TUBGCP3 PE=1 SV=2	0,2723009	0,703628
Q8N1G4	Leucine-rich repeat-containing protein 47 OS=Homo sapiens OX=9606 GN=LRRC47 PE=1 SV=1	-0,086257125	0,702207
Q13868	Exosome complex component RRP4 OS=Homo sapiens OX=9606 GN=EXOSC2 PE=1 SV=2	-0,245345229	0,700966
O43390	Heterogeneous nuclear ribonucleoprotein R OS=Homo sapiens OX=9606 GN=HNRNPR PE=1 SV=1	0,073313066	0,699623
Q9NR28	Diablo homolog, mitochondrial OS=Homo sapiens OX=9606 GN=DIABLO PE=1 SV=1	0,212361923	0,698645
O14908	PDZ domain-containing protein GIPC1 OS=Homo sapiens OX=9606 GN=GIPC1 PE=1 SV=2	0,09312859	0,698227
P62072	Mitochondrial import inner membrane translocase subunit Tim10 OS=Homo sapiens OX=9606 GN=TIMM10 PE=1 SV=1	-0,212199291	0,698058
Q9Y4K0	Lysyl oxidase homolog 2 OS=Homo sapiens OX=9606 GN=LOXL2 PE=1 SV=1	0,136208003	0,69735
Q96G03	Phosphoglucomutase-2 OS=Homo sapiens OX=9606 GN=PGM2 PE=1 SV=4	0,073009046	0,696219
Q9NTX5	Ethylmalonyl-CoA decarboxylase OS=Homo sapiens OX=9606 GN=ECHDC1 PE=1 SV=2	0,173663976	0,69545
Q9BRF8	Serine/threonine-protein phosphatase CPPED1 OS=Homo sapiens OX=9606 GN=CPPED1 PE=1 SV=3	-0,115526626	0,695228
Q6ZS17	Rho family-interacting cell polarization regulator 1 OS=Homo sapiens OX=9606 GN=RIPOR1 PE=1 SV=1	-0,124177541	0,693521
Q9GZP4	PITH domain-containing protein 1 OS=Homo sapiens OX=9606 GN=PITHD1 PE=1 SV=1	-0,30228656	0,693365
P28070	Proteasome subunit beta type-4 OS=Homo sapiens OX=9606 GN=PSMB4 PE=1 SV=4	-0,115218863	0,693081
Q9Y5X3	Sorting nexin-5 OS=Homo sapiens OX=9606 GN=SNX5 PE=1 SV=1	-0,124034573	0,692599
Q53HC9	EARP and GARP complex-interacting protein 1 OS=Homo sapiens OX=9606 GN=EIPR1 PE=1 SV=2	0,301864393	0,692399
P45954	Short/branched chain specific acyl-CoA dehydrogenase, mitochondrial OS=Homo sapiens OX=9606 GN=ACADSB PE=1 SV=1	-0,301404979	0,691347
Q96M27	Protein PRRC1 OS=Homo sapiens OX=9606 GN=PRRC1 PE=1 SV=1	0,101658538	0,690201
O60828	Polyglutamine-binding protein 1 OS=Homo sapiens OX=9606 GN=PQBP1 PE=1 SV=1	0,209919427	0,689835
Q9BZL6	Serine/threonine-protein kinase D2 OS=Homo sapiens OX=9606 GN=PRKD2 PE=1 SV=3	0,343361075	0,689571
Q8N2G8	GH3 domain-containing protein OS=Homo sapiens OX=9606 GN=GHDC PE=1 SV=2	-0,278298879	0,688026
Q5VU43	Myomegalin OS=Homo sapiens OX=9606 GN=PDE4DIP PE=1 SV=3	0,149381307	0,685656
P36404	ADP-ribosylation factor-like protein 2 OS=Homo sapiens OX=9606 GN=ARL2 PE=1 SV=4	-0,208604728	0,685097
Q92599	Septin-8 OS=Homo sapiens OX=9606 GN=SEPTIN8 PE=1 SV=4	-0,122858016	0,685017
Q9UKA4	A-kinase anchor protein 11 OS=Homo sapiens OX=9606 GN=AKAP11 PE=1 SV=1	0,298309514	0,684254
Q5T1J5	Putative coiled-coil-helix-coiled-coil-helix domain-containing protein CHCHD2P9, mitochondrial OS=Homo sapiens OX=9606 GN=CHCHD	0,298256968	0,684134
Q9NPL8	Complex I assembly factor TIMMDC1, mitochondrial OS=Homo sapiens OX=9606 GN=TIMMDC1 PE=1 SV=2	0,298199414	0,684002
Q969T9	WW domain-binding protein 2 OS=Homo sapiens OX=9606 GN=WBP2 PE=1 SV=1	-0,208119629	0,683349
P19971	Thymidine phosphorylase OS=Homo sapiens OX=9606 GN=TYMP PE=1 SV=2	0,208063784	0,683148
Q6P2E9	Enhancer of mRNA-decapping protein 4 OS=Homo sapiens OX=9606 GN=EDC4 PE=1 SV=1	0,081228032	0,68289
Q96D46	60S ribosomal export protein NMD3 OS=Homo sapiens OX=9606 GN=NMD3 PE=1 SV=1	-0,170821293	0,682634
Q6NUQ1	RAD50-interacting protein 1 OS=Homo sapiens OX=9606 GN=RINT1 PE=1 SV=1	0,297344857	0,682041
Q13057	Bifunctional coenzyme A synthase OS=Homo sapiens OX=9606 GN=COASY PE=1 SV=4	0,207551877	0,681305
Q96DG6	Carboxymethylenebutenolidase homolog OS=Homo sapiens OX=9606 GN=CMBL PE=1 SV=1	-0,148544451	0,681276
Q96PY5	Formin-like protein 2 OS=Homo sapiens OX=9606 GN=FMNL2 PE=1 SV=3	0,964896958	0,680624
A1A4S6	Rho GTPase-activating protein 10 OS=Homo sapiens OX=9606 GN=ARHGAP10 PE=1 SV=1	-0,101092416	0,680123
P17858	ATP-dependent 6-phosphofructokinase, liver type OS=Homo sapiens OX=9606 GN=PFKL PE=1 SV=6	-0,073514532	0,679171
P35869	Aryl hydrocarbon receptor OS=Homo sapiens OX=9606 GN=AHR PE=1 SV=2	-0,147972269	0,678285
O43294	Transforming growth factor beta-1-induced transcript 1 protein OS=Homo sapiens OX=9606 GN=TGFB1I1 PE=1 SV=2	0,083660707	0,677561
P23458	Tyrosine-protein kinase JAK1 OS=Homo sapiens OX=9606 GN=JAK1 PE=1 SV=2	0,105775276	0,676421
P01023	Alpha-2-macroglobulin OS=Homo sapiens OX=9606 GN=A2M PE=1 SV=3	0,121458309	0,676021
P35354	Prostaglandin G/H synthase 2 OS=Homo sapiens OX=9606 GN=PTGS2 PE=1 SV=2	-0,324325661	0,675476
P33897	ATP-binding cassette sub-family D member 1 OS=Homo sapiens OX=9606 GN=ABCD1 PE=1 SV=2	-0,294391629	0,675261
P53701	Cytochrome c-type heme lyase OS=Homo sapiens OX=9606 GN=HCCS PE=1 SV=1	0,147340465	0,674984
Q92804	TATA-binding protein-associated factor 2N OS=Homo sapiens OX=9606 GN=TAF15 PE=1 SV=1	0,294115084	0,674625
Q00653	Nuclear factor NF-kappa-B p100 subunit OS=Homo sapiens OX=9606 GN=NFKB2 PE=1 SV=4	0,205663946	0,674509
O60934	Nibrin OS=Homo sapiens OX=9606 GN=NBN PE=1 SV=1	-0,293946294	0,674237
Q12800	Alpha-globin transcription factor CP2 OS=Homo sapiens OX=9606 GN=TFCP2 PE=1 SV=2	0,205567672	0,674162
Q4G176	Acyl-CoA synthetase family member 3, mitochondrial OS=Homo sapiens OX=9606 GN=ACSF3 PE=1 SV=3	0,293451145	0,673099
Q9H0V1	Transmembrane protein 168 OS=Homo sapiens OX=9606 GN=TMEM168 PE=2 SV=2	0,334430978	0,672191
Q92574	Hamartin OS=Homo sapiens OX=9606 GN=TSC1 PE=1 SV=2	0,292845267	0,671706
P42771	Cyclin-dependent kinase inhibitor 2A OS=Homo sapiens OX=9606 GN=CDKN2A PE=1 SV=2	-0,204875112	0,671671
P23193	Transcription elongation factor A protein 1 OS=Homo sapiens OX=9606 GN=TCEA1 PE=1 SV=2	0,099224729	0,671032
P54687	Branched-chain-amino-acid aminotransferase, cytosolic OS=Homo sapiens OX=9606 GN=BCAT1 PE=1 SV=3	-0,111929023	0,670225
P35052	Glypican-1 OS=Homo sapiens OX=9606 GN=GPC1 PE=1 SV=2	0,08983384	0,669513
P62070	Ras-related protein R-Ras2 OS=Homo sapiens OX=9606 GN=RRAS2 PE=1 SV=1	0,120318048	0,668712
Q92616	eIF-2-alpha kinase activator GCN1 OS=Homo sapiens OX=9606 GN=GCN1 PE=1 SV=6	-0,031788074	0,668647
Q8IZH2	5'-3' exoribonuclease 1 OS=Homo sapiens OX=9606 GN=XRN1 PE=1 SV=1	-0,167687021	0,668546
P41226	Ubiquitin-like modifier-activating enzyme 7 OS=Homo sapiens OX=9606 GN=UBA7 PE=1 SV=2	-0,291237918	0,668009
Q92636	Protein FAN OS=Homo sapiens OX=9606 GN=NSMAF PE=1 SV=2	0,29049652	0,666302
P52907	F-actin-capping protein subunit alpha-1 OS=Homo sapiens OX=9606 GN=CAPZA1 PE=1 SV=3	-0,093641889	0,665767
P06744	Glucose-6-phosphate isomerase OS=Homo sapiens OX=9606 GN=GPI PE=1 SV=4	0,07026803	0,665719
P08581	Hepatocyte growth factor receptor OS=Homo sapiens OX=9606 GN=MET PE=1 SV=4	0,208342875	0,664893
Q9NXV6	CDKN2A-interacting protein OS=Homo sapiens OX=9606 GN=CDKN2AIP PE=1 SV=3	0,145322436	0,664462
Q13308	Inactive tyrosine-protein kinase 7 OS=Homo sapiens OX=9606 GN=PTK7 PE=1 SV=2	-0,093321244	0,663121
Q8WWB7	Glycosylated lysosomal membrane protein OS=Homo sapiens OX=9606 GN=GLMP PE=1 SV=1	-0,201799287	0,660616
Q9Y6K8	Adenylate kinase isoenzyme 5 OS=Homo sapiens OX=9606 GN=AK5 PE=1 SV=2	-0,20167765	0,660179
P48509	CD151 antigen OS=Homo sapiens OX=9606 GN=CD151 PE=1 SV=3	0,118872432	0,659469
Q99816	Tumor susceptibility gene 101 protein OS=Homo sapiens OX=9606 GN=TSG101 PE=1 SV=2	0,103385178	0,658751
Q92575	UBX domain-containing protein 4 OS=Homo sapiens OX=9606 GN=UBXN4 PE=1 SV=2	-0,165438525	0,658467
O94822	E3 ubiquitin-protein ligase listerin OS=Homo sapiens OX=9606 GN=LTN1 PE=1 SV=6	-0,286473691	0,657033
Q0IIM8	TBC1 domain family member 8B OS=Homo sapiens OX=9606 GN=TBC1D8B PE=1 SV=2	-0,200798765	0,657024
Q99758	ATP-binding cassette sub-family A member 3 OS=Homo sapiens OX=9606 GN=ABCA3 PE=1 SV=2	-0,265133283	0,656932
Q9Y5J9	Mitochondrial import inner membrane translocase subunit Tim8 B OS=Homo sapiens OX=9606 GN=TIMM8B PE=1 SV=1	-0,072532633	0,656358
A6NM62	Leucine-rich repeat-containing protein 53 OS=Homo sapiens OX=9606 GN=LRRC53 PE=4 SV=2	0,421759764	0,655742
Q7L014	Probable ATP-dependent RNA helicase DDX46 OS=Homo sapiens OX=9606 GN=DDX46 PE=1 SV=2	0,097265623	0,655694
P27105	Erythrocyte band 7 integral membrane protein OS=Homo sapiens OX=9606 GN=STOM PE=1 SV=3	0,109753936	0,655208
P51116	Fragile X mental retardation syndrome-related protein 2 OS=Homo sapiens OX=9606 GN=FXR2 PE=1 SV=2	0,109708004	0,654891
P25787	Proteasome subunit alpha type-2 OS=Homo sapiens OX=9606 GN=PSMA2 PE=1 SV=2	0,118057371	0,654269
Q27J81	Inverted formin-2 OS=Homo sapiens OX=9606 GN=INF2 PE=1 SV=2	0,054649776	0,653023
Q8WW12	PEST proteolytic signal-containing nuclear protein OS=Homo sapiens OX=9606 GN=PCNP PE=1 SV=2	0,143050509	0,652653
Q96QD9	UAP56-interacting factor OS=Homo sapiens OX=9606 GN=FYTTD1 PE=1 SV=3	-0,199567415	0,652605
P01111	GTPase NRas OS=Homo sapiens OX=9606 GN=NRAS PE=1 SV=1	-0,128413831	0,651751
Q16576	Histone-binding protein RBBP7 OS=Homo sapiens OX=9606 GN=RBBP7 PE=1 SV=1	-0,080897139	0,651565
Q9BZJ0	Crooked neck-like protein 1 OS=Homo sapiens OX=9606 GN=CRNKL1 PE=1 SV=4	0,283787064	0,650832
O15078	Centrosomal protein of 290 kDa OS=Homo sapiens OX=9606 GN=CEP290 PE=1 SV=2	0,149774384	0,650318
Q96K37	Solute carrier family 35 member E1 OS=Homo sapiens OX=9606 GN=SLC35E1 PE=1 SV=2	-0,142561603	0,650117
Q9HD33	39S ribosomal protein L47, mitochondrial OS=Homo sapiens OX=9606 GN=MRPL47 PE=1 SV=2	-0,198638565	0,649273
Q9H9A6	Leucine-rich repeat-containing protein 40 OS=Homo sapiens OX=9606 GN=LRRC40 PE=1 SV=1	-0,117177548	0,648667
P47895	Aldehyde dehydrogenase family 1 member A3 OS=Homo sapiens OX=9606 GN=ALDH1A3 PE=1 SV=2	-0,074971495	0,646928
Q9H694	Protein bicaudal C homolog 1 OS=Homo sapiens OX=9606 GN=BICC1 PE=1 SV=2	0,282052021	0,646824
P09110	3-ketoacyl-CoA thiolase, peroxisomal OS=Homo sapiens OX=9606 GN=ACAA1 PE=1 SV=2	-0,101739371	0,646645
Q4KMP7	TBC1 domain family member 10B OS=Homo sapiens OX=9606 GN=TBC1D10B PE=1 SV=3	-0,197515055	0,645245

A0A0B4J2D5	Glutamine amidotransferase-like class 1 domain-containing protein 3B, mitochondrial OS=Homo sapiens OX=9606 GN=GATD3B PE=1 SV	0,108212226	0,644608
P07437	Tubulin beta chain OS=Homo sapiens OX=9606 GN=TUBB PE=1 SV=2	0,116456886	0,644085
Q15165	Serum paraoxonase/arylesterase 2 OS=Homo sapiens OX=9606 GN=PON2 PE=1 SV=4	0,101186039	0,642586
Q92609	TBC1 domain family member 5 OS=Homo sapiens OX=9606 GN=TBC1D5 PE=1 SV=1	-0,097967913	0,64258
Q9UN37	Vacuolar protein sorting-associated protein 4A OS=Homo sapiens OX=9606 GN=VPS4A PE=1 SV=1	0,161417689	0,640503
Q9BQE5	Apolipoprotein L2 OS=Homo sapiens OX=9606 GN=APOL2 PE=1 SV=1	-0,126459141	0,640413
P49590	Probable histidine--tRNA ligase, mitochondrial OS=Homo sapiens OX=9606 GN=HARS2 PE=1 SV=1	0,279247133	0,640338
Q9Y5K8	V-type proton ATPase subunit D OS=Homo sapiens OX=9606 GN=ATP6V1D PE=1 SV=1	-0,196062285	0,640041
Q8NFY4	Semaphorin-6D OS=Homo sapiens OX=9606 GN=SEMA6D PE=1 SV=1	0,29487823	0,63969
P21796	Voltage-dependent anion-selective channel protein 1 OS=Homo sapiens OX=9606 GN=VDAC1 PE=1 SV=2	-0,074242535	0,639623
Q15003	Condensin complex subunit 2 OS=Homo sapiens OX=9606 GN=NCAPH PE=1 SV=3	0,140451791	0,639193
Q9BVM2	Protein DPCD OS=Homo sapiens OX=9606 GN=DPCD PE=1 SV=2	0,278563315	0,638755
O75122	CLIP-associating protein 2 OS=Homo sapiens OX=9606 GN=CLASP2 PE=1 SV=3	-0,27837568	0,638321
Q6FI81	Anamorsin OS=Homo sapiens OX=9606 GN=CIAPIN1 PE=1 SV=2	-0,115524118	0,638165
P61158	Actin-related protein 3 OS=Homo sapiens OX=9606 GN=ACTR3 PE=1 SV=3	0,064345	0,638133
Q9H4M9	EH domain-containing protein 1 OS=Homo sapiens OX=9606 GN=EHD1 PE=1 SV=2	0,061379364	0,637903
Q14192	Four and a half LIM domains protein 2 OS=Homo sapiens OX=9606 GN=FHL2 PE=1 SV=3	0,067734895	0,637836
Q13325	Interferon-induced protein with tetratricopeptide repeats 5 OS=Homo sapiens OX=9606 GN=IFIT5 PE=1 SV=1	-0,160671537	0,637178
P63167	Dynein light chain 1, cytoplasmic OS=Homo sapiens OX=9606 GN=DYNLL1 PE=1 SV=1	0,277772632	0,636925
Q96P48	Arf-GAP with Rho-GAP domain, ANK repeat and PH domain-containing protein 1 OS=Homo sapiens OX=9606 GN=ARAP1 PE=1 SV=3	-0,16059644	0,636843
Q68CZ2	Tensin-3 OS=Homo sapiens OX=9606 GN=TNS3 PE=1 SV=2	-0,195094561	0,636576
P13489	Ribonuclease inhibitor OS=Homo sapiens OX=9606 GN=RNH1 PE=1 SV=2	0,057481214	0,635939
O14828	Secretory carrier-associated membrane protein 3 OS=Homo sapiens OX=9606 GN=SCAMP3 PE=1 SV=3	0,125655385	0,635762
Q9Y224	RNA transcription, translation and transport factor protein OS=Homo sapiens OX=9606 GN=RTRAF PE=1 SV=1	0,085879594	0,635433
P04181	Ornithine aminotransferase, mitochondrial OS=Homo sapiens OX=9606 GN=OAT PE=1 SV=1	-0,085815947	0,634887
Q5T5P2	Sickle tail protein homolog OS=Homo sapiens OX=9606 GN=KIAA1217 PE=1 SV=2	-0,194564669	0,63468
Q9BZG1	Ras-related protein Rab-34 OS=Homo sapiens OX=9606 GN=RAB34 PE=1 SV=1	0,114939697	0,634462
Q8N4A0	Polypeptide N-acetylgalactosaminyltransferase 4 OS=Homo sapiens OX=9606 GN=GALNT4 PE=1 SV=2	-0,276669823	0,634371
Q7Z739	YTH domain-containing family protein 3 OS=Homo sapiens OX=9606 GN=YTHDF3 PE=1 SV=1	0,106692416	0,634196
Q86V87	#N/D	0,276337261	0,6336
P68036	Ubiquitin-conjugating enzyme E2 L3 OS=Homo sapiens OX=9606 GN=UBE2L3 PE=1 SV=1	-0,106582175	0,633443
P47914	60S ribosomal protein L29 OS=Homo sapiens OX=9606 GN=RPL29 PE=1 SV=2	0,276207322	0,633299
O14579	Coatomer subunit epsilon OS=Homo sapiens OX=9606 GN=COPE PE=1 SV=3	-0,085622221	0,633229
Q9NPQ8	Synembryn-A OS=Homo sapiens OX=9606 GN=RIC8A PE=1 SV=3	0,078927018	0,633182
O94854	Uncharacterized protein KIAA0754 OS=Homo sapiens OX=9606 GN=KIAA0754 PE=2 SV=4	-0,275352142	0,631317
Q99832	T-complex protein 1 subunit eta OS=Homo sapiens OX=9606 GN=CCT7 PE=1 SV=2	0,048349772	0,630919
P30086	Phosphatidylethanolamine-binding protein 1 OS=Homo sapiens OX=9606 GN=PEBP1 PE=1 SV=3	-0,085350929	0,630908
P09486	SPARC OS=Homo sapiens OX=9606 GN=SPARC PE=1 SV=1	-0,085334437	0,630767
P49354	Protein farnesyltransferase/geranylgeranyltransferase type-1 subunit alpha OS=Homo sapiens OX=9606 GN=FNTA PE=1 SV=1	-0,09400962	0,630382
Q7Z4H8	Protein O-glucosyltransferase 3 OS=Homo sapiens OX=9606 GN=POGLUT3 PE=1 SV=2	-0,085255768	0,630094
Q96RU3	Formin-binding protein 1 OS=Homo sapiens OX=9606 GN=FNBP1 PE=1 SV=2	0,274801551	0,630041
Q15650	Activating signal cointegrator 1 OS=Homo sapiens OX=9606 GN=TRIP4 PE=1 SV=4	0,266506586	0,629819
Q9H3F6	BTB/POZ domain-containing adapter for CUL3-mediated RhoA degradation protein 3 OS=Homo sapiens OX=9606 GN=KCTD10 PE=1 SV=	-0,292863947	0,629754
O00461	Golgi integral membrane protein 4 OS=Homo sapiens OX=9606 GN=GOLIM4 PE=1 SV=1	0,073240287	0,629611
Q7L1Q6	Basic leucine zipper and W2 domain-containing protein 1 OS=Homo sapiens OX=9606 GN=BZW1 PE=1 SV=1	0,066857711	0,628249
P06753	Tropomyosin alpha-3 chain OS=Homo sapiens OX=9606 GN=TPM3 PE=1 SV=2	-0,192686783	0,627963
P28331	NADH-ubiquinone oxidoreductase 75 kDa subunit, mitochondrial OS=Homo sapiens OX=9606 GN=NDUFS1 PE=1 SV=3	-0,078297454	0,627334
Q9NQT5	Exosome complex component RRP40 OS=Homo sapiens OX=9606 GN=EXOSC3 PE=1 SV=3	-0,273152318	0,626216
O60264	SWI/SNF-related matrix-associated actin-dependent regulator of chromatin subfamily A member 5 OS=Homo sapiens OX=9606 GN=SM/	-0,137888627	0,625967
Q8TDB6	E3 ubiquitin-protein ligase DTX3L OS=Homo sapiens OX=9606 GN=DTX3L PE=1 SV=1	-0,272957285	0,625764
P47756	F-actin-capping protein subunit beta OS=Homo sapiens OX=9606 GN=CAPZB PE=1 SV=4	0,070479553	0,624636
P23229	Integrin alpha-6 OS=Homo sapiens OX=9606 GN=ITGA6 PE=1 SV=5	0,191687723	0,624393
Q8WVM8	Sec1 family domain-containing protein 1 OS=Homo sapiens OX=9606 GN=SCFD1 PE=1 SV=4	0,064702911	0,623727
Q2M389	WASH complex subunit 4 OS=Homo sapiens OX=9606 GN=WASHC4 PE=1 SV=2	-0,084447703	0,623195
Q9HAB8	Phosphopantothenate--cysteine ligase OS=Homo sapiens OX=9606 GN=PPCS PE=1 SV=2	0,137214482	0,622497
Q8IWA5	Choline transporter-like protein 2 OS=Homo sapiens OX=9606 GN=SLC44A2 PE=1 SV=3	-0,157272885	0,622066
P19404	NADH dehydrogenase [ubiquinone] flavoprotein 2, mitochondrial OS=Homo sapiens OX=9606 GN=NDUFV2 PE=1 SV=2	-0,271043533	0,621322
P13807	Glycogen [starch] synthase, muscle OS=Homo sapiens OX=9606 GN=GYS1 PE=1 SV=2	0,12300637	0,620483
P55769	NHP2-like protein 1 OS=Homo sapiens OX=9606 GN=SNU13 PE=1 SV=3	-0,190532888	0,620268
P06703	Protein S100-A6 OS=Homo sapiens OX=9606 GN=S100A6 PE=1 SV=1	0,088064305	0,620079
O75533	Splicing factor 3B subunit 1 OS=Homo sapiens OX=9606 GN=SF3B1 PE=1 SV=3	-0,049766543	0,619688
P06737	Glycogen phosphorylase, liver form OS=Homo sapiens OX=9606 GN=PYGL PE=1 SV=4	-0,072229522	0,619553
O75718	Cartilage-associated protein OS=Homo sapiens OX=9606 GN=CRTAP PE=1 SV=1	-0,067917676	0,619351
Q9UBV2	Protein sel-1 homolog 1 OS=Homo sapiens OX=9606 GN=SEL1L PE=1 SV=3	-0,136516235	0,618906
P62834	Ras-related protein Rap-1A OS=Homo sapiens OX=9606 GN=RAP1A PE=1 SV=1	0,036206854	0,618898
Q9UI10	Translation initiation factor eIF-2B subunit delta OS=Homo sapiens OX=9606 GN=EIF2B4 PE=1 SV=2	0,190079421	0,618649
Q9Y6A4	Cilia- and flagella-associated protein 20 OS=Homo sapiens OX=9606 GN=CFAP20 PE=1 SV=1	-0,150654915	0,618266
Q9NRG7	Epimerase family protein SDR39U1 OS=Homo sapiens OX=9606 GN=SDR39U1 PE=1 SV=3	-0,269703045	0,618209
O75494	Serine/arginine-rich splicing factor 10 OS=Homo sapiens OX=9606 GN=SRSF10 PE=1 SV=1	0,156274574	0,617638
P56545	C-terminal-binding protein 2 OS=Homo sapiens OX=9606 GN=CTBP2 PE=1 SV=1	-0,1224993	0,617566
O60664	Perilipin-3 OS=Homo sapiens OX=9606 GN=PLIN3 PE=1 SV=3	-0,056013614	0,617102
Q9P2T1	GMP reductase 2 OS=Homo sapiens OX=9606 GN=GMPR2 PE=1 SV=1	-0,112158012	0,616897
Q9NQS3	Nectin-3 OS=Homo sapiens OX=9606 GN=NECTIN3 PE=1 SV=1	-0,269117332	0,616848
Q14019	Coactosin-like protein OS=Homo sapiens OX=9606 GN=COTL1 PE=1 SV=3	-0,097514402	0,615798
Q92990	Glomulin OS=Homo sapiens OX=9606 GN=GLMN PE=1 SV=2	-0,189260317	0,615725
P45984	Mitogen-activated protein kinase 9 OS=Homo sapiens OX=9606 GN=MAPK9 PE=1 SV=2	-0,189198281	0,615504
P20073	Annexin A7 OS=Homo sapiens OX=9606 GN=ANXA7 PE=1 SV=3	0,065625071	0,614836
Q9BUF5	Tubulin beta-6 chain OS=Homo sapiens OX=9606 GN=TUBB6 PE=1 SV=1	-0,087353355	0,614309
P49458	Signal recognition particle 9 kDa protein OS=Homo sapiens OX=9606 GN=SRP9 PE=1 SV=2	0,135507089	0,613724
P58546	Myotrophin OS=Homo sapiens OX=9606 GN=MTPN PE=1 SV=2	0,111547737	0,613057
Q6ZS30	Neurobeachin-like protein 1 OS=Homo sapiens OX=9606 GN=NBEAL1 PE=2 SV=3	-0,118049741	0,612761
P22694	cAMP-dependent protein kinase catalytic subunit beta OS=Homo sapiens OX=9606 GN=PRKACB PE=1 SV=2	0,188256538	0,612145
Q13496	Myotubularin OS=Homo sapiens OX=9606 GN=MTM1 PE=1 SV=2	-0,170991729	0,611504
Q9Y5S9	RNA-binding protein 8A OS=Homo sapiens OX=9606 GN=RBM8A PE=1 SV=1	0,135034275	0,611298
Q9UHY1	Nuclear receptor-binding protein OS=Homo sapiens OX=9606 GN=NRBP1 PE=1 SV=1	-0,103253621	0,610774
Q9BUP0	EF-hand domain-containing protein D1 OS=Homo sapiens OX=9606 GN=EFHD1 PE=1 SV=1	0,134928457	0,610756
Q99961	Endophilin-A2 OS=Homo sapiens OX=9606 GN=SH3GL1 PE=1 SV=1	-0,079513806	0,610459
Q9Y3I0	tRNA-splicing ligase RtcB homolog OS=Homo sapiens OX=9606 GN=RTCB PE=1 SV=1	0,065195603	0,610179
O00300	Tumor necrosis factor receptor superfamily member 11B OS=Homo sapiens OX=9606 GN=TNFRSF11B PE=1 SV=3	-0,264825292	0,606866
Q15386	Ubiquitin-protein ligase E3C OS=Homo sapiens OX=9606 GN=UBE3C PE=1 SV=3	0,110416233	0,605951
P82675	28S ribosomal protein S5, mitochondrial OS=Homo sapiens OX=9606 GN=MRPS5 PE=1 SV=2	0,264423916	0,605931
Q9BRJ7	Tudor-interacting repair regulator protein OS=Homo sapiens OX=9606 GN=NUDT16L1 PE=1 SV=1	-0,263885175	0,604677
P41236	Protein phosphatase inhibitor 2 OS=Homo sapiens OX=9606 GN=PPP1R2 PE=1 SV=2	-0,325866541	0,604227
P53597	Succinate--CoA ligase [ADP/GDP-forming] subunit alpha, mitochondrial OS=Homo sapiens OX=9606 GN=SUCLG1 PE=1 SV=4	-0,110053684	0,603678
Q9Y625	Glypican-6 OS=Homo sapiens OX=9606 GN=GPC6 PE=1 SV=1	0,26334344	0,603416
O43148	mRNA cap guanine-N7 methyltransferase OS=Homo sapiens OX=9606 GN=RNMT PE=1 SV=1	-0,263042199	0,602714
Q9Y5P4	Collagen type IV alpha-3-binding protein OS=Homo sapiens OX=9606 GN=COL4A3BP PE=1 SV=1	-0,109867086	0,602509
Q96GC9	Vacuole membrane protein 1 OS=Homo sapiens OX=9606 GN=VMP1 PE=1 SV=1	-0,262678885	0,601868

Q9BT23	LIM domain-containing protein 2 OS=Homo sapiens OX=9606 GN=LIMD2 PE=1 SV=1	-0,132904406	0,600967
Q13459	Unconventional myosin-IXb OS=Homo sapiens OX=9606 GN=MYO9B PE=1 SV=3	-0,070341425	0,600866
Q9BW27	Nuclear pore complex protein Nup85 OS=Homo sapiens OX=9606 GN=NUP85 PE=1 SV=1	-0,109560007	0,600585
Q8NEZ4	Histone-lysine N-methyltransferase 2C OS=Homo sapiens OX=9606 GN=KMT2C PE=1 SV=3	0,262101884	0,600524
Q7Z3C6	Autophagy-related protein 9A OS=Homo sapiens OX=9606 GN=ATG9A PE=1 SV=3	-0,262075569	0,600462
Q9Y2Q9	28S ribosomal protein S28, mitochondrial OS=Homo sapiens OX=9606 GN=MRPS28 PE=1 SV=1	0,26192553	0,600113
Q5QJE6	Deoxynucleotidyltransferase terminal-interacting protein 2 OS=Homo sapiens OX=9606 GN=DNTTIP2 PE=1 SV=2	0,313871865	0,60007
P62316	Small nuclear ribonucleoprotein Sm D2 OS=Homo sapiens OX=9606 GN=SNRPD2 PE=1 SV=1	-0,09531076	0,599841
Q9UJU6	Drebrin-like protein OS=Homo sapiens OX=9606 GN=DBNL PE=1 SV=1	-0,072637212	0,599768
Q9NQZ2	Something about silencing protein 10 OS=Homo sapiens OX=9606 GN=UTP3 PE=1 SV=1	0,261736868	0,599673
P55036	26S proteasome non-ATPase regulatory subunit 4 OS=Homo sapiens OX=9606 GN=PSMD4 PE=1 SV=1	0,095263648	0,599501
Q5T9L3	Protein wntless homolog OS=Homo sapiens OX=9606 GN=WLS PE=1 SV=2	0,261647272	0,599464
Q96QU8	Exportin-6 OS=Homo sapiens OX=9606 GN=XPO6 PE=1 SV=1	-0,320141129	0,599164
Q9BXS5	AP-1 complex subunit mu-1 OS=Homo sapiens OX=9606 GN=AP1M1 PE=1 SV=3	0,078186432	0,598722
O14618	Copper chaperone for superoxide dismutase OS=Homo sapiens OX=9606 GN=CCS PE=1 SV=1	-0,26114587	0,598296
Q02241	Kinesin-like protein KIF23 OS=Homo sapiens OX=9606 GN=KIF23 PE=1 SV=3	0,261035072	0,598037
P07858	Cathepsin B OS=Homo sapiens OX=9606 GN=CTSB PE=1 SV=3	0,094866587	0,596636
Q9H2M9	Rab3 GTPase-activating protein non-catalytic subunit OS=Homo sapiens OX=9606 GN=RAB3GAP2 PE=1 SV=1	0,074927173	0,596245
Q9H792	Inactive tyrosine-protein kinase PEAK1 OS=Homo sapiens OX=9606 GN=PEAK1 PE=1 SV=4	0,118633492	0,595421
P55081	Microfibrillar-associated protein 1 OS=Homo sapiens OX=9606 GN=MFAP1 PE=1 SV=2	0,183533764	0,595326
Q96KA5	Cleft lip and palate transmembrane protein 1-like protein OS=Homo sapiens OX=9606 GN=CLPTM1L PE=1 SV=1	-0,151070353	0,594633
Q9BXW7	Haloacid dehalogenase-like hydrolase domain-containing 5 OS=Homo sapiens OX=9606 GN=HDHD5 PE=1 SV=1	0,171959739	0,593448
Q9ULR0	Pre-mRNA-splicing factor ISY1 homolog OS=Homo sapiens OX=9606 GN=ISY1 PE=1 SV=3	0,258920263	0,593106
Q9UMY4	Sorting nexin-12 OS=Homo sapiens OX=9606 GN=SNX12 PE=1 SV=3	-0,118226381	0,593099
Q8N3F8	MICAL-like protein 1 OS=Homo sapiens OX=9606 GN=MICALL1 PE=1 SV=2	-0,258892593	0,593042
P15291	Beta-1,4-galactosyltransferase 1 OS=Homo sapiens OX=9606 GN=B4GALT1 PE=1 SV=5	-0,108324362	0,592859
Q8N9T8	Protein KRI1 homolog OS=Homo sapiens OX=9606 GN=KRI1 PE=1 SV=3	0,258647734	0,59247
O43149	Zinc finger ZZ-type and EF-hand domain-containing protein 1 OS=Homo sapiens OX=9606 GN=ZZEF1 PE=1 SV=6	-0,258430819	0,591964
Q99575	Ribonucleases P/MRP protein subunit POP1 OS=Homo sapiens OX=9606 GN=POP1 PE=1 SV=2	-0,258378635	0,591843
Q9NZI7	Upstream-binding protein 1 OS=Homo sapiens OX=9606 GN=UBP1 PE=1 SV=1	-0,257457334	0,589693
P06733	Alpha-enolase OS=Homo sapiens OX=9606 GN=ENO1 PE=1 SV=2	-0,057349789	0,589609
Q6WCQ1	Myosin phosphatase Rho-interacting protein OS=Homo sapiens OX=9606 GN=MPRIP PE=1 SV=3	0,05000905	0,589037
Q13418	Integrin-linked protein kinase OS=Homo sapiens OX=9606 GN=ILK PE=1 SV=2	0,057270998	0,588673
O95486	Protein transport protein Sec24A OS=Homo sapiens OX=9606 GN=SEC24A PE=1 SV=2	-0,071251944	0,586605
P41223	Protein BUD31 homolog OS=Homo sapiens OX=9606 GN=BUD31 PE=1 SV=2	0,181074341	0,586586
O95747	Serine/threonine-protein kinase OSR1 OS=Homo sapiens OX=9606 GN=OXSR1 PE=1 SV=1	-0,068787002	0,585583
Q13485	Mothers against decapentaplegic homolog 4 OS=Homo sapiens OX=9606 GN=SMAD4 PE=1 SV=1	-0,1299755	0,585457
Q00610	Clathrin heavy chain 1 OS=Homo sapiens OX=9606 GN=CLTC PE=1 SV=5	-0,027905317	0,585452
O95628	CCR4-NOT transcription complex subunit 4 OS=Homo sapiens OX=9606 GN=CNOT4 PE=1 SV=3	0,21554237	0,584858
Q8IX01	SURP and G-patch domain-containing protein 2 OS=Homo sapiens OX=9606 GN=SUGP2 PE=1 SV=2	0,255091038	0,584168
Q96J84	Kin of IRRE-like protein 1 OS=Homo sapiens OX=9606 GN=KIRREL1 PE=1 SV=2	0,129523967	0,58316
Q63HR2	Tensin-2 OS=Homo sapiens OX=9606 GN=TNS2 PE=1 SV=2	-0,254640648	0,583116
Q15427	Splicing factor 3B subunit 4 OS=Homo sapiens OX=9606 GN=SF3B4 PE=1 SV=1	0,129483585	0,582955
O14817	Tetraspanin-4 OS=Homo sapiens OX=9606 GN=TSPAN4 PE=1 SV=1	0,254417827	0,582595
Q96A72	Protein mago nashi homolog 2 OS=Homo sapiens OX=9606 GN=MAGOHB PE=1 SV=1	-0,083978832	0,581628
P62993	Growth factor receptor-bound protein 2 OS=Homo sapiens OX=9606 GN=GRB2 PE=1 SV=1	0,087631472	0,581457
Q86TB3	Alpha-protein kinase 2 OS=Homo sapiens OX=9606 GN=ALPK2 PE=2 SV=3	-0,179305676	0,580309
O15042	U2 snRNP-associated SURP motif-containing protein OS=Homo sapiens OX=9606 GN=U2SURP PE=1 SV=2	0,147777511	0,580147
Q8WWM9	Cytoglobin OS=Homo sapiens OX=9606 GN=CYGB PE=1 SV=1	-0,147435428	0,578646
O43242	26S proteasome non-ATPase regulatory subunit 3 OS=Homo sapiens OX=9606 GN=PSMD3 PE=1 SV=2	-0,057725896	0,578604
P47224	Guanine nucleotide exchange factor MSS4 OS=Homo sapiens OX=9606 GN=RABIF PE=1 SV=2	-0,252166604	0,577334
P78330	Phosphoserine phosphatase OS=Homo sapiens OX=9606 GN=PSPH PE=1 SV=2	-0,105786363	0,577053
Q12899	Tripartite motif-containing protein 26 OS=Homo sapiens OX=9606 GN=TRIM26 PE=1 SV=1	-0,251846866	0,576586
Q14699	Raftlin OS=Homo sapiens OX=9606 GN=RFTN1 PE=1 SV=4	0,067778764	0,575718
Q13561	Dynactin subunit 2 OS=Homo sapiens OX=9606 GN=DCTN2 PE=1 SV=4	0,06028794	0,574807
Q659C4	La-related protein 1B OS=Homo sapiens OX=9606 GN=LARP1B PE=1 SV=2	-0,031401553	0,572421
Q96AQ6	Pre-B-cell leukemia transcription factor-interacting protein 1 OS=Homo sapiens OX=9606 GN=PBXIP1 PE=1 SV=1	0,176682813	0,571013
Q08380	Galectin-3-binding protein OS=Homo sapiens OX=9606 GN=LGALS3BP PE=1 SV=1	-0,145640221	0,570775
Q8IZL8	Proline-, glutamic acid- and leucine-rich protein 1 OS=Homo sapiens OX=9606 GN=PELP1 PE=1 SV=2	0,145436036	0,569881
Q8IZP0	Abl interactor 1 OS=Homo sapiens OX=9606 GN=ABI1 PE=1 SV=4	-0,104591093	0,569639
P16989	Y-box-binding protein 3 OS=Homo sapiens OX=9606 GN=YBX3 PE=1 SV=4	0,091078583	0,569452
P38117	Electron transfer flavoprotein subunit beta OS=Homo sapiens OX=9606 GN=ETFB PE=1 SV=3	0,081772381	0,569439
Q8WWH5	Probable tRNA pseudouridine synthase 1 OS=Homo sapiens OX=9606 GN=TRUB1 PE=1 SV=1	-0,248455092	0,568651
Q9UI26	Importin-11 OS=Homo sapiens OX=9606 GN=IPO11 PE=1 SV=1	0,175891557	0,568211
Q9BUK6	Protein misato homolog 1 OS=Homo sapiens OX=9606 GN=MSTO1 PE=1 SV=1	-0,175875767	0,568155
O76031	ATP-dependent Clp protease ATP-binding subunit clpX-like, mitochondrial OS=Homo sapiens OX=9606 GN=CLPX PE=1 SV=2	0,14500203	0,567981
Q5W0V3	Protein FAM160B1 OS=Homo sapiens OX=9606 GN=FAM160B1 PE=1 SV=1	0,069256454	0,567762
Q9Y3Z3	Deoxynucleoside triphosphate triphosphohydrolase SAMHD1 OS=Homo sapiens OX=9606 GN=SAMHD1 PE=1 SV=2	-0,175722414	0,567613
Q02978	Mitochondrial 2-oxoglutarate/malate carrier protein OS=Homo sapiens OX=9606 GN=SLC25A11 PE=1 SV=3	0,081518271	0,567414
P49327	Fatty acid synthase OS=Homo sapiens OX=9606 GN=FASN PE=1 SV=3	-0,027753602	0,567244
O14545	TRAF-type zinc finger domain-containing protein 1 OS=Homo sapiens OX=9606 GN=TRAFD1 PE=1 SV=1	-0,144763235	0,566936
P09429	High mobility group protein B1 OS=Homo sapiens OX=9606 GN=HMGB1 PE=1 SV=3	0,090522183	0,565482
Q86VN1	Vacuolar protein-sorting-associated protein 36 OS=Homo sapiens OX=9606 GN=VPS36 PE=1 SV=1	0,458442197	0,565324
Q9UN70	Protocadherin gamma-C3 OS=Homo sapiens OX=9606 GN=PCDHGC3 PE=1 SV=1	-0,144083896	0,565282
Q96TC7	Regulator of microtubule dynamics protein 3 OS=Homo sapiens OX=9606 GN=RMDN3 PE=1 SV=2	-0,125765485	0,564106
Q7L5D6	Golgi to ER traffic protein 4 homolog OS=Homo sapiens OX=9606 GN=GET4 PE=1 SV=1	0,246457439	0,563974
P06493	Cyclin-dependent kinase 1 OS=Homo sapiens OX=9606 GN=CDK1 PE=1 SV=3	0,143984521	0,563531
Q15021	Condensin complex subunit 1 OS=Homo sapiens OX=9606 GN=NCAPD2 PE=1 SV=3	0,096190993	0,563265
P20042	Eukaryotic translation initiation factor 2 subunit 2 OS=Homo sapiens OX=9606 GN=EIF2S2 PE=1 SV=2	0,056326463	0,562493
P52948	Nuclear pore complex protein Nup98-Nup96 OS=Homo sapiens OX=9606 GN=NUP98 PE=1 SV=4	-0,112788636	0,562242
Q96PU8	Protein quaking OS=Homo sapiens OX=9606 GN=QKI PE=1 SV=1	-0,143644052	0,562043
O75340	Programmed cell death protein 6 OS=Homo sapiens OX=9606 GN=PDCD6 PE=1 SV=1	0,103294775	0,56162
Q14671	Pumilio homolog 1 OS=Homo sapiens OX=9606 GN=PUM1 PE=1 SV=3	-0,143527624	0,561534
O14561	Acyl carrier protein, mitochondrial OS=Homo sapiens OX=9606 GN=NDUFAB1 PE=1 SV=3	0,173961111	0,561383
Q9Y6W5	Wiskott-Aldrich syndrome protein family member 2 OS=Homo sapiens OX=9606 GN=WASF2 PE=1 SV=3	-0,125210086	0,5613
Q13563	Polycystin-2 OS=Homo sapiens OX=9606 GN=PKD2 PE=1 SV=3	-0,245257316	0,561163
Q9ULF5	Zinc transporter ZIP10 OS=Homo sapiens OX=9606 GN=SLC39A10 PE=1 SV=2	-0,17373922	0,560598
Q96AC1	Fermitin family homolog 2 OS=Homo sapiens OX=9606 GN=FERMT2 PE=1 SV=1	-0,044932141	0,560393
P01344	Insulin-like growth factor II OS=Homo sapiens OX=9606 GN=IGF2 PE=1 SV=1	-0,244860603	0,560233
O75044	SLIT-ROBO Rho GTPase-activating protein 2 OS=Homo sapiens OX=9606 GN=SRGAP2 PE=1 SV=3	-0,244639383	0,559715
Q9UMS4	Pre-mRNA-processing factor 19 OS=Homo sapiens OX=9606 GN=PRPF19 PE=1 SV=1	-0,076866804	0,55932
P49588	Alanine--tRNA ligase, cytoplasmic OS=Homo sapiens OX=9606 GN=AARS PE=1 SV=2	-0,044850542	0,559223
Q99590	Protein SCAF11 OS=Homo sapiens OX=9606 GN=SCAF11 PE=1 SV=2	0,243789793	0,557724
P21964	Catechol O-methyltransferase OS=Homo sapiens OX=9606 GN=COMT PE=1 SV=2	-0,068178443	0,557641
Q9H6B4	CXADR-like membrane protein OS=Homo sapiens OX=9606 GN=CLMP PE=1 SV=1	0,124068623	0,555541
Q99470	Stromal cell-derived factor 2 OS=Homo sapiens OX=9606 GN=SDF2 PE=1 SV=2	0,24265832	0,555071
Q9BTT0	Acidic leucine-rich nuclear phosphoprotein 32 family member E OS=Homo sapiens OX=9606 GN=ANP32E PE=1 SV=1	-0,242457132	0,554599
Q96MW5	Conserved oligomeric Golgi complex subunit 8 OS=Homo sapiens OX=9606 GN=COG8 PE=1 SV=2	0,171976988	0,554373

P11117	Lysosomal acid phosphatase OS=Homo sapiens OX=9606 GN=ACP2 PE=1 SV=3	-0,10207602	0,554102
O43592	Exportin-T OS=Homo sapiens OX=9606 GN=XPOT PE=1 SV=2	0,055586962	0,554022
Q99755	Phosphatidylinositol 4-phosphate 5-kinase type-1 alpha OS=Homo sapiens OX=9606 GN=PIP5K1A PE=1 SV=1	-0,171854896	0,553942
P49356	Protein farnesyltransferase subunit beta OS=Homo sapiens OX=9606 GN=FNTB PE=1 SV=1	0,242101137	0,553764
Q9Y3Y2	Chromatin target of PRMT1 protein OS=Homo sapiens OX=9606 GN=CHTOP PE=1 SV=2	0,241895548	0,553282
P46063	ATP-dependent DNA helicase Q1 OS=Homo sapiens OX=9606 GN=RECQL PE=1 SV=3	-0,054267344	0,553282
Q9NYU2	UDP-glucose:glycoprotein glucosyltransferase 1 OS=Homo sapiens OX=9606 GN=UGGT1 PE=1 SV=3	0,0414186	0,552935
O95379	Tumor necrosis factor alpha-induced protein 8 OS=Homo sapiens OX=9606 GN=TNFAIP8 PE=1 SV=1	-0,141507007	0,552718
Q96G28	Cilia- and flagella-associated protein 36 OS=Homo sapiens OX=9606 GN=CFAP36 PE=1 SV=2	0,241601494	0,552593
Q8IXI2	Mitochondrial Rho GTPase 1 OS=Homo sapiens OX=9606 GN=RHOT1 PE=1 SV=2	-0,241503696	0,552363
Q14353	Guanidinoacetate N-methyltransferase OS=Homo sapiens OX=9606 GN=GAMT PE=1 SV=1	-0,240250157	0,549423
Q96IJ6	Mannose-1-phosphate guanyltransferase alpha OS=Homo sapiens OX=9606 GN=GMPPA PE=1 SV=1	0,079236236	0,549299
Q96GG9	DCN1-like protein 1 OS=Homo sapiens OX=9606 GN=DCUN1D1 PE=1 SV=1	-0,110172313	0,54751
P09211	Glutathione S-transferase P OS=Homo sapiens OX=9606 GN=GSTP1 PE=1 SV=2	0,067010572	0,546721
P48444	Coatomer subunit delta OS=Homo sapiens OX=9606 GN=ARCN1 PE=1 SV=1	0,050391164	0,546216
P60900	Proteasome subunit alpha type-6 OS=Homo sapiens OX=9606 GN=PSMA6 PE=1 SV=1	-0,075217337	0,545627
P30711	Glutathione S-transferase theta-1 OS=Homo sapiens OX=9606 GN=GSTT1 PE=1 SV=4	-0,238575614	0,545493
Q9NUL3	Double-stranded RNA-binding protein Staufen homolog 2 OS=Homo sapiens OX=9606 GN=STAU2 PE=1 SV=2	-0,238545173	0,545421
O00743	Serine/threonine-protein phosphatase 6 catalytic subunit OS=Homo sapiens OX=9606 GN=PPP6C PE=1 SV=1	-0,078625383	0,544471
Q9NRX4	14 kDa phosphohistidine phosphatase OS=Homo sapiens OX=9606 GN=PHPT1 PE=1 SV=1	-0,121846189	0,544358
O00154	Cytosolic acyl coenzyme A thioester hydrolase OS=Homo sapiens OX=9606 GN=ACOT7 PE=1 SV=3	-0,082643511	0,543806
Q9HBI1	Beta-parvin OS=Homo sapiens OX=9606 GN=PARVB PE=1 SV=1	-0,168927735	0,54362
Q13107	Ubiquitin carboxyl-terminal hydrolase 4 OS=Homo sapiens OX=9606 GN=USP4 PE=1 SV=3	-0,121592077	0,543082
P42285	Exosome RNA helicase MTR4 OS=Homo sapiens OX=9606 GN=MTREX PE=1 SV=3	0,092937714	0,541652
Q8IWJ2	GRIP and coiled-coil domain-containing protein 2 OS=Homo sapiens OX=9606 GN=GCC2 PE=1 SV=4	0,138812571	0,540995
O75084	Frizzled-7 OS=Homo sapiens OX=9606 GN=FZD7 PE=1 SV=2	0,236634607	0,540935
P01112	GTPase HRas OS=Homo sapiens OX=9606 GN=HRAS PE=1 SV=1	-0,236315051	0,540185
Q9UJ41	Rab5 GDP/GTP exchange factor OS=Homo sapiens OX=9606 GN=RABGEF1 PE=1 SV=3	-0,108851431	0,540101
Q13200	26S proteasome non-ATPase regulatory subunit 2 OS=Homo sapiens OX=9606 GN=PSMD2 PE=1 SV=3	-0,039522075	0,539468
Q14677	Clathrin interactor 1 OS=Homo sapiens OX=9606 GN=CLINT1 PE=1 SV=1	0,071379803	0,539358
Q8IX05	CD302 antigen OS=Homo sapiens OX=9606 GN=CD302 PE=1 SV=1	0,141113684	0,538667
P05387	60S acidic ribosomal protein P2 OS=Homo sapiens OX=9606 GN=RPLP2 PE=1 SV=1	-0,108555689	0,538444
Q00535	Cyclin-dependent-like kinase 5 OS=Homo sapiens OX=9606 GN=CDK5 PE=1 SV=3	0,120562692	0,537918
O94888	UBX domain-containing protein 7 OS=Homo sapiens OX=9606 GN=UBXN7 PE=1 SV=2	0,235008743	0,537116
Q15678	Tyrosine-protein phosphatase non-receptor type 14 OS=Homo sapiens OX=9606 GN=PTPN14 PE=1 SV=2	-0,166778797	0,536055
P18615	Negative elongation factor E OS=Homo sapiens OX=9606 GN=NELFE PE=1 SV=3	-0,166750639	0,535955
Q9NZD8	Maspardin OS=Homo sapiens OX=9606 GN=SPG21 PE=1 SV=1	0,293037668	0,53534
Q15397	Pumilio homolog 3 OS=Homo sapiens OX=9606 GN=PUM3 PE=1 SV=3	0,233786492	0,534244
Q9Y3F4	Serine-threonine kinase receptor-associated protein OS=Homo sapiens OX=9606 GN=STRAP PE=1 SV=1	0,061479569	0,533786
P54725	UV excision repair protein RAD23 homolog A OS=Homo sapiens OX=9606 GN=RAD23A PE=1 SV=1	-0,098718135	0,533492
Q9NS86	LanC-like protein 2 OS=Homo sapiens OX=9606 GN=LANCL2 PE=1 SV=1	-0,107658084	0,533423
Q9H6T3	RNA polymerase II-associated protein 3 OS=Homo sapiens OX=9606 GN=RPAP3 PE=1 SV=2	0,233157069	0,532765
Q7L775	EPM2A-interacting protein 1 OS=Homo sapiens OX=9606 GN=EPM2AIP1 PE=1 SV=1	0,211249033	0,53154
P67870	Casein kinase II subunit beta OS=Homo sapiens OX=9606 GN=CSNK2B PE=1 SV=1	-0,107052141	0,530038
Q9P266	Junctional protein associated with coronary artery disease OS=Homo sapiens OX=9606 GN=JCAD PE=1 SV=3	0,231962273	0,529956
Q9UG01	Intraflagellar transport protein 172 homolog OS=Homo sapiens OX=9606 GN=IFT172 PE=1 SV=2	0,231655094	0,529234
Q96II8	Leucine-rich repeat and calponin homology domain-containing protein 3 OS=Homo sapiens OX=9606 GN=LRCH3 PE=1 SV=2	-0,231166202	0,528084
O96008	Mitochondrial import receptor subunit TOM40 homolog OS=Homo sapiens OX=9606 GN=TOMM40 PE=1 SV=1	-0,106631652	0,527692
Q5EB52	Mesoderm-specific transcript homolog protein OS=Homo sapiens OX=9606 GN=MEST PE=2 SV=2	0,118501682	0,527604
O00178	GTP-binding protein 1 OS=Homo sapiens OX=9606 GN=GTPBP1 PE=1 SV=3	-0,164229179	0,527094
P60468	Protein transport protein Sec61 subunit beta OS=Homo sapiens OX=9606 GN=SEC61B PE=1 SV=2	0,135485238	0,526572
Q9Y230	RuvB-like 2 OS=Homo sapiens OX=9606 GN=RUVBL2 PE=1 SV=3	0,058957893	0,526386
Q9H492	Microtubule-associated proteins 1A/1B light chain 3A OS=Homo sapiens OX=9606 GN=MAP1LC3A PE=1 SV=2	-0,163961088	0,526152
Q9NRN7	L-aminoadipate-semialdehyde dehydrogenase-phosphopantetheinyl transferase OS=Homo sapiens OX=9606 GN=AASDHPPT PE=1 SV=2	-0,230248243	0,525925
P0DN79	Cystathionine beta-synthase-like protein OS=Homo sapiens OX=9606 GN=CBSL PE=1 SV=1	0,090405828	0,524952
P56385	ATP synthase subunit e, mitochondrial OS=Homo sapiens OX=9606 GN=ATP5ME PE=1 SV=2	-0,134934859	0,524192
P09012	U1 small nuclear ribonucleoprotein A OS=Homo sapiens OX=9606 GN=SNRPA PE=1 SV=3	0,105973107	0,524021
P37198	Nuclear pore glycoprotein p62 OS=Homo sapiens OX=9606 GN=NUP62 PE=1 SV=3	-0,117712032	0,523662
Q15654	Thyroid receptor-interacting protein 6 OS=Homo sapiens OX=9606 GN=TRIP6 PE=1 SV=3	-0,069533615	0,523494
Q8WXX5	DnaJ homolog subfamily C member 9 OS=Homo sapiens OX=9606 GN=DNAJC9 PE=1 SV=1	-0,1176523	0,523364
P10301	Ras-related protein R-Ras OS=Homo sapiens OX=9606 GN=RRAS PE=1 SV=1	0,084524207	0,523058
P61289	Proteasome activator complex subunit 3 OS=Homo sapiens OX=9606 GN=PSME3 PE=1 SV=1	-0,0758627	0,522751
Q9Y5Z4	Heme-binding protein 2 OS=Homo sapiens OX=9606 GN=HEBP2 PE=1 SV=1	-0,117508609	0,522648
Q9BTC0	Death-inducer obliterator 1 OS=Homo sapiens OX=9606 GN=DIDO1 PE=1 SV=5	0,162849886	0,522253
Q9Y639	Neuroplastin OS=Homo sapiens OX=9606 GN=NPTN PE=1 SV=2	0,117429361	0,522253
Q8TC20	Cancer-associated gene 1 protein OS=Homo sapiens OX=9606 GN=CAGE1 PE=1 SV=2	-0,419397832	0,522249
P57740	Nuclear pore complex protein Nup107 OS=Homo sapiens OX=9606 GN=NUP107 PE=1 SV=1	0,10563351	0,522129
Q969G5	Caveolae-associated protein 3 OS=Homo sapiens OX=9606 GN=CAVIN3 PE=1 SV=3	-0,084224894	0,520959
Q9Y520	Protein PRRC2C OS=Homo sapiens OX=9606 GN=PRRC2C PE=1 SV=4	-0,062101143	0,520881
Q7LG56	Ribonucleoside-diphosphate reductase subunit M2 B OS=Homo sapiens OX=9606 GN=RRM2B PE=1 SV=1	0,134080961	0,520503
P46776	60S ribosomal protein L27a OS=Homo sapiens OX=9606 GN=RPL27A PE=1 SV=2	-0,133906744	0,519751
Q99436	Proteasome subunit beta type-7 OS=Homo sapiens OX=9606 GN=PSMB7 PE=1 SV=1	-0,071971054	0,518893
Q9NR09	Baculoviral IAP repeat-containing protein 6 OS=Homo sapiens OX=9606 GN=BIRC6 PE=1 SV=2	0,161732625	0,518335
P35625	Metalloproteinase inhibitor 3 OS=Homo sapiens OX=9606 GN=TIMP3 PE=1 SV=2	0,08378524	0,517879
P12821	Angiotensin-converting enzyme OS=Homo sapiens OX=9606 GN=ACE PE=1 SV=1	-0,226027901	0,515995
P62753	40S ribosomal protein S6 OS=Homo sapiens OX=9606 GN=RPS6 PE=1 SV=1	0,083401414	0,515193
Q53FT3	Protein Hikeshi OS=Homo sapiens OX=9606 GN=HIKESHI PE=1 SV=2	-0,225604769	0,514998
Q5BJH7	Protein YIF1B OS=Homo sapiens OX=9606 GN=YIF1B PE=1 SV=1	-0,160569536	0,514261
Q9C0G0	Zinc finger protein 407 OS=Homo sapiens OX=9606 GN=ZNF407 PE=1 SV=2	0,215534473	0,513921
Q96DV4	39S ribosomal protein L38, mitochondrial OS=Homo sapiens OX=9606 GN=MRPL38 PE=1 SV=2	-0,160361025	0,51353
Q9P035	Very-long-chain (3R)-3-hydroxyacyl-CoA dehydratase 3 OS=Homo sapiens OX=9606 GN=HACD3 PE=1 SV=2	0,095369751	0,513094
P25786	Proteasome subunit alpha type-1 OS=Homo sapiens OX=9606 GN=PSMA1 PE=1 SV=1	0,061270759	0,512963
Q8N122	Regulatory-associated protein of mTOR OS=Homo sapiens OX=9606 GN=RPTOR PE=1 SV=1	0,224507454	0,512415
P08579	U2 small nuclear ribonucleoprotein B'' OS=Homo sapiens OX=9606 GN=SNRPB2 PE=1 SV=1	-0,132127128	0,512077
P00167	Cytochrome b5 OS=Homo sapiens OX=9606 GN=CYB5A PE=1 SV=2	-0,103596092	0,51081
O15511	Actin-related protein 2/3 complex subunit 5 OS=Homo sapiens OX=9606 GN=ARPC5 PE=1 SV=3	0,088216574	0,510596
O43665	Regulator of G-protein signaling 10 OS=Homo sapiens OX=9606 GN=RGS10 PE=1 SV=3	0,223264724	0,509488
P09493	Tropomyosin alpha-1 chain OS=Homo sapiens OX=9606 GN=TPM1 PE=1 SV=2	-0,055711358	0,509479
O75643	U5 small nuclear ribonucleoprotein 200 kDa helicase OS=Homo sapiens OX=9606 GN=SNRNP200 PE=1 SV=2	-0,044812541	0,509154
Q9NXC5	GATOR complex protein MIOS OS=Homo sapiens OX=9606 GN=MIOS PE=1 SV=2	0,222941759	0,508727
Q13144	Translation initiation factor eIF-2B subunit epsilon OS=Homo sapiens OX=9606 GN=EIF2B5 PE=1 SV=3	0,114483657	0,5076
P51812	Ribosomal protein S6 kinase alpha-3 OS=Homo sapiens OX=9606 GN=RPS6KA3 PE=1 SV=1	0,11445309	0,507448
P54727	UV excision repair protein RAD23 homolog B OS=Homo sapiens OX=9606 GN=RAD23B PE=1 SV=1	-0,077753413	0,507417
A5D8V6	Vacuolar protein sorting-associated protein 37C OS=Homo sapiens OX=9606 GN=VPS37C PE=1 SV=2	-0,222203976	0,506989
Q6DKJ4	Nucleoredoxin OS=Homo sapiens OX=9606 GN=NXN PE=1 SV=2	-0,073753808	0,506296
Q9Y6R1	Electrogenic sodium bicarbonate cotransporter 1 OS=Homo sapiens OX=9606 GN=SLC4A4 PE=1 SV=1	0,158214833	0,506022
Q8TD19	Serine/threonine-protein kinase Nek9 OS=Homo sapiens OX=9606 GN=NEK9 PE=1 SV=2	-0,070277864	0,505062
O43615	Mitochondrial import inner membrane translocase subunit TIM44 OS=Homo sapiens OX=9606 GN=TIMM44 PE=1 SV=2	0,102432024	0,504363

Q96D71	RalBP1-associated Eps domain-containing protein 1 OS=Homo sapiens OX=9606 GN=REPS1 PE=1 SV=3	-0,220676551	0,50339
Q9BU89	Deoxyhypusine hydroxylase OS=Homo sapiens OX=9606 GN=DOHH PE=1 SV=1	-0,113500584	0,502726
P55735	Protein SEC13 homolog OS=Homo sapiens OX=9606 GN=SEC13 PE=1 SV=3	-0,0581663	0,501162
P07197	Neurofilament medium polypeptide OS=Homo sapiens OX=9606 GN=NEFM PE=1 SV=3	0,219230988	0,499983
P61923	Coatomer subunit zeta-1 OS=Homo sapiens OX=9606 GN=COPZ1 PE=1 SV=1	0,129287154	0,499868
P08237	ATP-dependent 6-phosphofructokinase, muscle type OS=Homo sapiens OX=9606 GN=PFKM PE=1 SV=2	-0,086537631	0,499641
Q9BRS8	La-related protein 6 OS=Homo sapiens OX=9606 GN=LARP6 PE=1 SV=1	-0,21902133	0,499489
P10620	Microsomal glutathione S-transferase 1 OS=Homo sapiens OX=9606 GN=MGST1 PE=1 SV=1	0,15632022	0,499404
P62341	Thioredoxin reductase-like selenoprotein T OS=Homo sapiens OX=9606 GN=SELENOT PE=1 SV=2	0,218837332	0,499056
Q13439	Golgin subfamily A member 4 OS=Homo sapiens OX=9606 GN=GOLGA4 PE=1 SV=1	0,061829766	0,498857
Q15046	Lysine--tRNA ligase OS=Homo sapiens OX=9606 GN=KARS PE=1 SV=3	-0,046535328	0,498774
O14562	Ubiquitin domain-containing protein UBFD1 OS=Homo sapiens OX=9606 GN=UBFD1 PE=1 SV=2	0,128989156	0,498589
P50225	Sulfotransferase 1A1 OS=Homo sapiens OX=9606 GN=SULT1A1 PE=1 SV=3	-0,386696116	0,49844
Q8IXS6	Paralemmin-2 OS=Homo sapiens OX=9606 GN=PALM2 PE=1 SV=3	0,218517508	0,498302
Q9H267	Vacuolar protein sorting-associated protein 33B OS=Homo sapiens OX=9606 GN=VPS33B PE=1 SV=2	-0,112604809	0,498291
P30043	Flavin reductase (NADPH) OS=Homo sapiens OX=9606 GN=BLVRB PE=1 SV=3	-0,092837759	0,497772
Q9UIJ7	GTP:AMP phosphotransferase AK3, mitochondrial OS=Homo sapiens OX=9606 GN=AK3 PE=1 SV=4	0,12878137	0,497698
P40121	Macrophage-capping protein OS=Homo sapiens OX=9606 GN=CAPG PE=1 SV=2	0,072576021	0,497154
O14744	Protein arginine N-methyltransferase 5 OS=Homo sapiens OX=9606 GN=PRMT5 PE=1 SV=4	0,059545108	0,49659
P07954	Fumarate hydratase, mitochondrial OS=Homo sapiens OX=9606 GN=FH PE=1 SV=3	0,056012602	0,496579
P16234	Platelet-derived growth factor receptor alpha OS=Homo sapiens OX=9606 GN=PDGFRA PE=1 SV=1	0,128493483	0,496463
P63098	Calcineurin subunit B type 1 OS=Homo sapiens OX=9606 GN=PPP3R1 PE=1 SV=2	0,217533976	0,495983
Q16864	V-type proton ATPase subunit F OS=Homo sapiens OX=9606 GN=ATP6V1F PE=1 SV=2	-0,155298346	0,495838
Q9UPQ0	LIM and calponin homology domains-containing protein 1 OS=Homo sapiens OX=9606 GN=LIMCH1 PE=1 SV=4	0,048214857	0,495633
P31937	3-hydroxyisobutyrate dehydrogenase, mitochondrial OS=Homo sapiens OX=9606 GN=HIBADH PE=1 SV=2	0,076143944	0,495555
P28370	Probable global transcription activator SNF2L1 OS=Homo sapiens OX=9606 GN=SMARCA1 PE=1 SV=2	-0,217344769	0,495537
P35250	Replication factor C subunit 2 OS=Homo sapiens OX=9606 GN=RFC2 PE=1 SV=3	-0,217027807	0,49479
O75367	Core histone macro-H2A.1 OS=Homo sapiens OX=9606 GN=H2AFY PE=1 SV=4	0,076038707	0,494781
Q9BX68	Histidine triad nucleotide-binding protein 2, mitochondrial OS=Homo sapiens OX=9606 GN=HINT2 PE=1 SV=1	-0,111893973	0,494778
Q15746	Myosin light chain kinase, smooth muscle OS=Homo sapiens OX=9606 GN=MYLK PE=1 SV=4	0,068940854	0,494196
Q9NVS9	Pyridoxine-5'-phosphate oxidase OS=Homo sapiens OX=9606 GN=PNPO PE=1 SV=1	-0,127739008	0,493231
Q53QV2	Protein LBH OS=Homo sapiens OX=9606 GN=LBH PE=1 SV=1	0,216316641	0,493113
Q9BWJ5	Splicing factor 3B subunit 5 OS=Homo sapiens OX=9606 GN=SF3B5 PE=1 SV=1	-0,154503419	0,493067
P05121	Plasminogen activator inhibitor 1 OS=Homo sapiens OX=9606 GN=SERPINE1 PE=1 SV=1	0,054077432	0,492545
P18621	60S ribosomal protein L17 OS=Homo sapiens OX=9606 GN=RPL17 PE=1 SV=3	0,085375827	0,492087
A5YKK6	CCR4-NOT transcription complex subunit 1 OS=Homo sapiens OX=9606 GN=CNOT1 PE=1 SV=2	-0,065790663	0,491641
P09874	Poly [ADP-ribose] polymerase 1 OS=Homo sapiens OX=9606 GN=PARP1 PE=1 SV=4	-0,060995424	0,491237
P41227	N-alpha-acetyltransferase 10 OS=Homo sapiens OX=9606 GN=NAA10 PE=1 SV=1	-0,091604483	0,490342
Q9NWU2	Glucose-induced degradation protein 8 homolog OS=Homo sapiens OX=9606 GN=GID8 PE=1 SV=1	-0,110930412	0,490021
P62249	40S ribosomal protein S16 OS=Homo sapiens OX=9606 GN=RPS16 PE=1 SV=2	0,091540753	0,489958
P61960	Ubiquitin-fold modifier 1 OS=Homo sapiens OX=9606 GN=UFM1 PE=1 SV=1	-0,1268469	0,489413
O60231	Pre-mRNA-splicing factor ATP-dependent RNA helicase DHX16 OS=Homo sapiens OX=9606 GN=DHX16 PE=1 SV=2	-0,214710031	0,489324
Q3V6T2	Girdin OS=Homo sapiens OX=9606 GN=CCDC88A PE=1 SV=2	-0,21459506	0,489052
P29558	RNA-binding motif, single-stranded-interacting protein 1 OS=Homo sapiens OX=9606 GN=RBMS1 PE=1 SV=3	-0,214561271	0,488973
Q8NHH9	Atlastin-2 OS=Homo sapiens OX=9606 GN=ATL2 PE=1 SV=2	-0,21437843	0,488541
Q9HBH5	Retinol dehydrogenase 14 OS=Homo sapiens OX=9606 GN=RDH14 PE=1 SV=1	0,214339151	0,488449
P28288	ATP-binding cassette sub-family D member 3 OS=Homo sapiens OX=9606 GN=ABCD3 PE=1 SV=1	0,079374328	0,487181
Q9H993	Damage-control phosphatase ARMT1 OS=Homo sapiens OX=9606 GN=ARMT1 PE=1 SV=1	-0,110248273	0,486659
P62244	40S ribosomal protein S15a OS=Homo sapiens OX=9606 GN=RPS15A PE=1 SV=2	-0,090969994	0,486527
Q9BX40	Protein LSM14 homolog B OS=Homo sapiens OX=9606 GN=LSM14B PE=1 SV=1	0,152368074	0,48563
Q96JB2	Conserved oligomeric Golgi complex subunit 3 OS=Homo sapiens OX=9606 GN=COG3 PE=1 SV=3	-0,152341833	0,485538
Q9NXF1	Testis-expressed protein 10 OS=Homo sapiens OX=9606 GN=TEX10 PE=1 SV=2	-0,152279637	0,485322
Q96B26	Exosome complex component RRP43 OS=Homo sapiens OX=9606 GN=EXOSC8 PE=1 SV=1	0,152250385	0,48522
Q99805	Transmembrane 9 superfamily member 2 OS=Homo sapiens OX=9606 GN=TM9SF2 PE=1 SV=1	-0,078839192	0,483482
O14880	Microsomal glutathione S-transferase 3 OS=Homo sapiens OX=9606 GN=MGST3 PE=1 SV=1	0,098604239	0,483271
P08574	Cytochrome c1, heme protein, mitochondrial OS=Homo sapiens OX=9606 GN=CYC1 PE=1 SV=3	-0,098449088	0,48242
P52597	Heterogeneous nuclear ribonucleoprotein F OS=Homo sapiens OX=9606 GN=HNRNPF PE=1 SV=3	0,0743458	0,482369
Q96CM8	Acyl-CoA synthetase family member 2, mitochondrial OS=Homo sapiens OX=9606 GN=ACSF2 PE=1 SV=2	0,150825261	0,480265
Q8IWI9	MAX gene-associated protein OS=Homo sapiens OX=9606 GN=MGA PE=1 SV=4	0,150557631	0,479335
Q9NQW7	Xaa-Pro aminopeptidase 1 OS=Homo sapiens OX=9606 GN=XPNPEP1 PE=1 SV=3	0,054280987	0,479228
P55072	Transitional endoplasmic reticulum ATPase OS=Homo sapiens OX=9606 GN=VCP PE=1 SV=4	0,028430029	0,47907
P49459	Ubiquitin-conjugating enzyme E2 A OS=Homo sapiens OX=9606 GN=UBE2A PE=1 SV=2	-0,17365719	0,478743
Q7Z7G0	Target of Nesh-SH3 OS=Homo sapiens OX=9606 GN=ABI3BP PE=1 SV=1	0,124347938	0,478741
Q8N1B4	Vacuolar protein sorting-associated protein 52 homolog OS=Homo sapiens OX=9606 GN=VPS52 PE=1 SV=1	0,23884056	0,4787
Q6NVY1	3-hydroxyisobutyryl-CoA hydrolase, mitochondrial OS=Homo sapiens OX=9606 GN=HIBCH PE=1 SV=2	-0,097516316	0,477307
Q9NVJ2	ADP-ribosylation factor-like protein 8B OS=Homo sapiens OX=9606 GN=ARL8B PE=1 SV=1	-0,209446463	0,476904
P20618	Proteasome subunit beta type-1 OS=Homo sapiens OX=9606 GN=PSMB1 PE=1 SV=2	0,063867185	0,475434
Q9ULH0	Kinase D-interacting substrate of 220 kDa OS=Homo sapiens OX=9606 GN=KIDINS220 PE=1 SV=3	-0,208339292	0,474291
Q9NZZ3	Charged multivesicular body protein 5 OS=Homo sapiens OX=9606 GN=CHMP5 PE=1 SV=1	-0,123124305	0,473529
Q92747	Actin-related protein 2/3 complex subunit 1A OS=Homo sapiens OX=9606 GN=ARPC1A PE=2 SV=2	0,057088573	0,473476
P42338	Phosphatidylinositol 4,5-bisphosphate 3-kinase catalytic subunit beta isoform OS=Homo sapiens OX=9606 GN=PIK3CB PE=1 SV=1	0,650768388	0,472489
Q14157	Ubiquitin-associated protein 2-like OS=Homo sapiens OX=9606 GN=UBAP2L PE=1 SV=2	-0,049477945	0,4722
P56192	Methionine--tRNA ligase, cytoplasmic OS=Homo sapiens OX=9606 GN=MARS PE=1 SV=2	0,045238192	0,472173
Q08AD1	Calmodulin-regulated spectrin-associated protein 2 OS=Homo sapiens OX=9606 GN=CAMSAP2 PE=1 SV=3	-0,148416925	0,471903
O95168	NADH dehydrogenase [ubiquinone] 1 beta subcomplex subunit 4 OS=Homo sapiens OX=9606 GN=NDUFB4 PE=1 SV=3	-0,207178321	0,47155
Q8NFH5	Nucleoporin NUP35 OS=Homo sapiens OX=9606 GN=NUP35 PE=1 SV=1	-0,25726871	0,470555
P69905	Hemoglobin subunit alpha OS=Homo sapiens OX=9606 GN=HBA1 PE=1 SV=2	0,287608576	0,470517
Q9UMS0	NFU1 iron-sulfur cluster scaffold homolog, mitochondrial OS=Homo sapiens OX=9606 GN=NFU1 PE=1 SV=2	-0,206670339	0,470351
P23141	Liver carboxylesterase 1 OS=Homo sapiens OX=9606 GN=CES1 PE=1 SV=2	-0,072698077	0,470348
Q15262	Receptor-type tyrosine-protein phosphatase kappa OS=Homo sapiens OX=9606 GN=PTPRK PE=1 SV=2	-0,206565316	0,470103
P78560	Death domain-containing protein CRADD OS=Homo sapiens OX=9606 GN=CRADD PE=1 SV=1	-0,20654801	0,470062
Q05209	Tyrosine-protein phosphatase non-receptor type 12 OS=Homo sapiens OX=9606 GN=PTPN12 PE=1 SV=3	-0,088148758	0,469632
P32322	Pyrroline-5-carboxylate reductase 1, mitochondrial OS=Homo sapiens OX=9606 GN=PYCR1 PE=1 SV=2	0,068955306	0,469261
P19525	Interferon-induced, double-stranded RNA-activated protein kinase OS=Homo sapiens OX=9606 GN=EIF2AK2 PE=1 SV=2	-0,063059618	0,468662
Q8NE86	Calcium uniporter protein, mitochondrial OS=Homo sapiens OX=9606 GN=MCU PE=1 SV=1	-0,106461013	0,468062
Q8WUP2	Filamin-binding LIM protein 1 OS=Homo sapiens OX=9606 GN=FBLIM1 PE=1 SV=2	0,065693111	0,468002
Q15631	Translin OS=Homo sapiens OX=9606 GN=TSN PE=1 SV=1	0,087820784	0,467676
Q15369	Elongin-C OS=Homo sapiens OX=9606 GN=ELOC PE=1 SV=1	0,095705439	0,467408
P11172	Uridine 5'-monophosphate synthase OS=Homo sapiens OX=9606 GN=UMPS PE=1 SV=1	-0,072239137	0,46701
P30049	ATP synthase subunit delta, mitochondrial OS=Homo sapiens OX=9606 GN=ATP5F1D PE=1 SV=2	-0,204506225	0,465241
A1X283	SH3 and PX domain-containing protein 2B OS=Homo sapiens OX=9606 GN=SH3PXD2B PE=1 SV=3	-0,071959369	0,464978
Q96FV2	Secernin-2 OS=Homo sapiens OX=9606 GN=SCRN2 PE=1 SV=3	-0,121064266	0,464773
O43292	Glycosylphosphatidylinositol anchor attachment 1 protein OS=Homo sapiens OX=9606 GN=GPAA1 PE=1 SV=3	-0,204131921	0,464357
P20337	Ras-related protein Rab-3B OS=Homo sapiens OX=9606 GN=RAB3B PE=1 SV=2	0,068311008	0,464332
P61009	Signal peptidase complex subunit 3 OS=Homo sapiens OX=9606 GN=SPCS3 PE=1 SV=1	-0,146214067	0,464269
O15397	Importin-8 OS=Homo sapiens OX=9606 GN=IPO8 PE=1 SV=2	0,146160563	0,464084
Q9GZY8	Mitochondrial fission factor OS=Homo sapiens OX=9606 GN=MFF PE=1 SV=1	0,120697341	0,463216
O75970	Multiple PDZ domain protein OS=Homo sapiens OX=9606 GN=MPDZ PE=1 SV=2	0,203614777	0,463136

P05386	60S acidic ribosomal protein P1 OS=Homo sapiens OX=9606 GN=RPLP1 PE=1 SV=1	-0,145222659	0,460839
Q9BY44	Eukaryotic translation initiation factor 2A OS=Homo sapiens OX=9606 GN=EIF2A PE=1 SV=3	-0,05750127	0,459588
Q15056	Eukaryotic translation initiation factor 4H OS=Homo sapiens OX=9606 GN=EIF4H PE=1 SV=5	-0,071157257	0,45916
P07737	Profilin-1 OS=Homo sapiens OX=9606 GN=PFN1 PE=1 SV=2	0,05953916	0,458959
Q14249	Endonuclease G, mitochondrial OS=Homo sapiens OX=9606 GN=ENDOG PE=1 SV=4	-0,201566692	0,458299
Q14444	Caprin-1 OS=Homo sapiens OX=9606 GN=CAPRIN1 PE=1 SV=2	0,059450417	0,45819
P02461	Collagen alpha-1(III) chain OS=Homo sapiens OX=9606 GN=COL3A1 PE=1 SV=4	0,049399231	0,458172
P49441	Inositol polyphosphate 1-phosphatase OS=Homo sapiens OX=9606 GN=INPP1 PE=1 SV=1	-0,144293993	0,457627
Q8IWS0	PHD finger protein 6 OS=Homo sapiens OX=9606 GN=PHF6 PE=1 SV=1	0,201197257	0,457426
Q9NVV4	Poly(A) RNA polymerase, mitochondrial OS=Homo sapiens OX=9606 GN=MTPAP PE=1 SV=1	-0,20111282	0,457227
Q12805	EGF-containing fibulin-like extracellular matrix protein 1 OS=Homo sapiens OX=9606 GN=EFEMP1 PE=1 SV=2	0,119076797	0,456349
Q15643	Thyroid receptor-interacting protein 11 OS=Homo sapiens OX=9606 GN=TRIP11 PE=1 SV=3	-0,059168099	0,455743
Q9UBU9	Nuclear RNA export factor 1 OS=Homo sapiens OX=9606 GN=NXF1 PE=1 SV=1	0,143127936	0,453599
Q9NRG9	Aladin OS=Homo sapiens OX=9606 GN=AAAS PE=1 SV=1	0,142565209	0,451656
P19320	Vascular cell adhesion protein 1 OS=Homo sapiens OX=9606 GN=VCAM1 PE=1 SV=1	-0,066645064	0,451632
P22102	Trifunctional purine biosynthetic protein adenosine-3 OS=Homo sapiens OX=9606 GN=GART PE=1 SV=1	-0,041771729	0,451164
Q6Y7W6	GRB10-interacting GYF protein 2 OS=Homo sapiens OX=9606 GN=GIGYF2 PE=1 SV=1	0,06085612	0,450285
P51805	Plexin-A3 OS=Homo sapiens OX=9606 GN=PLXNA3 PE=1 SV=2	-0,117466762	0,449542
Q8N6R0	eEF1A lysine and N-terminal methyltransferase OS=Homo sapiens OX=9606 GN=EEF1AKNMT PE=1 SV=1	-0,19753699	0,44878
Q9HCU5	Prolactin regulatory element-binding protein OS=Homo sapiens OX=9606 GN=PREB PE=1 SV=2	0,092180157	0,448243
O14530	Thioredoxin domain-containing protein 9 OS=Homo sapiens OX=9606 GN=TXNDC9 PE=1 SV=2	-0,102388733	0,448204
Q9UIQ6	Leucyl-cystinyl aminopeptidase OS=Homo sapiens OX=9606 GN=LNPEP PE=1 SV=3	0,066171857	0,448038
Q9H8S9	MOB kinase activator 1A OS=Homo sapiens OX=9606 GN=MOB1A PE=1 SV=4	0,084511384	0,448015
O76024	Wolframin OS=Homo sapiens OX=9606 GN=WFS1 PE=1 SV=2	-0,102301641	0,44778
Q9HCC0	Methylcrotonoyl-CoA carboxylase beta chain, mitochondrial OS=Homo sapiens OX=9606 GN=MCCC2 PE=1 SV=1	-0,102200487	0,447289
Q13685	Angio-associated migratory cell protein OS=Homo sapiens OX=9606 GN=AAMP PE=1 SV=2	0,09197184	0,447115
P51665	26S proteasome non-ATPase regulatory subunit 7 OS=Homo sapiens OX=9606 GN=PSMD7 PE=1 SV=2	0,066030699	0,446966
P43378	Tyrosine-protein phosphatase non-receptor type 9 OS=Homo sapiens OX=9606 GN=PTPN9 PE=1 SV=1	0,141146157	0,446762
O95248	Myotubularin-related protein 5 OS=Homo sapiens OX=9606 GN=SBF1 PE=1 SV=4	-0,116719897	0,44639
Q3YEC7	Rab-like protein 6 OS=Homo sapiens OX=9606 GN=RABL6 PE=1 SV=2	0,116719032	0,446386
Q07954	Prolow-density lipoprotein receptor-related protein 1 OS=Homo sapiens OX=9606 GN=LRP1 PE=1 SV=2	-0,020124946	0,445763
O00560	Syntenin-1 OS=Homo sapiens OX=9606 GN=SDCBP PE=1 SV=1	0,116506066	0,445488
O14672	Disintegrin and metalloproteinase domain-containing protein 10 OS=Homo sapiens OX=9606 GN=ADAM10 PE=1 SV=1	-0,140664662	0,445103
Q15750	TGF-beta-activated kinase 1 and MAP3K7-binding protein 1 OS=Homo sapiens OX=9606 GN=TAB1 PE=1 SV=1	-0,195735503	0,444523
O43166	Signal-induced proliferation-associated 1-like protein 1 OS=Homo sapiens OX=9606 GN=SIPA1L1 PE=1 SV=4	0,06559557	0,443667
O00629	Importin subunit alpha-3 OS=Homo sapiens OX=9606 GN=KPNA4 PE=1 SV=1	0,065524303	0,443127
Q86V85	Integral membrane protein GPR180 OS=Homo sapiens OX=9606 GN=GPR180 PE=2 SV=1	0,173098242	0,442592
Q13885	Tubulin beta-2A chain OS=Homo sapiens OX=9606 GN=TUBB2A PE=1 SV=1	0,035975842	0,442572
Q9GZT6	Coiled-coil domain-containing protein 90B, mitochondrial OS=Homo sapiens OX=9606 GN=CCDC90B PE=1 SV=2	0,164530753	0,442315
Q06265	Exosome complex component RRP45 OS=Homo sapiens OX=9606 GN=EXOSC9 PE=1 SV=3	-0,194275738	0,441074
Q969E4	Transcription elongation factor A protein-like 3 OS=Homo sapiens OX=9606 GN=TCEAL3 PE=1 SV=1	0,194208422	0,440915
Q01432	AMP deaminase 3 OS=Homo sapiens OX=9606 GN=AMPD3 PE=1 SV=1	0,194008742	0,440444
Q15029	116 kDa U5 small nuclear ribonucleoprotein component OS=Homo sapiens OX=9606 GN=EFTUD2 PE=1 SV=1	-0,046446583	0,439745
Q13614	Myotubularin-related protein 2 OS=Homo sapiens OX=9606 GN=MTMR2 PE=1 SV=4	-0,30246454	0,439454
Q9H2U2	Inorganic pyrophosphatase 2, mitochondrial OS=Homo sapiens OX=9606 GN=PPA2 PE=1 SV=2	0,06835022	0,438913
P54289	Voltage-dependent calcium channel subunit alpha-2/delta-1 OS=Homo sapiens OX=9606 GN=CACNA2D1 PE=1 SV=3	-0,062029963	0,438803
Q92552	28S ribosomal protein S27, mitochondrial OS=Homo sapiens OX=9606 GN=MRPS27 PE=1 SV=3	0,193207356	0,43855
P28482	Mitogen-activated protein kinase 1 OS=Homo sapiens OX=9606 GN=MAPK1 PE=1 SV=3	0,055146895	0,438504
Q9HCJ1	Progressive ankylosis protein homolog OS=Homo sapiens OX=9606 GN=ANKH PE=1 SV=2	-0,193158709	0,438435
P62913	60S ribosomal protein L11 OS=Homo sapiens OX=9606 GN=RPL11 PE=1 SV=2	0,114818843	0,43838
Q15286	Ras-related protein Rab-35 OS=Homo sapiens OX=9606 GN=RAB35 PE=1 SV=1	0,090341106	0,438302
Q86XZ4	Spermatogenesis-associated serine-rich protein 2 OS=Homo sapiens OX=9606 GN=SPATS2 PE=1 SV=1	-0,106818572	0,437855
O15498	Synaptobrevin homolog YKT6 OS=Homo sapiens OX=9606 GN=YKT6 PE=1 SV=1	0,076907429	0,437702
Q14139	Ubiquitin conjugation factor E4 A OS=Homo sapiens OX=9606 GN=UBE4A PE=1 SV=2	0,138458251	0,437508
O43768	Alpha-endosulfine OS=Homo sapiens OX=9606 GN=ENSA PE=1 SV=1	-0,11456741	0,437323
O76041	Nebulette OS=Homo sapiens OX=9606 GN=NEBL PE=1 SV=1	0,353895635	0,4367
P09619	Platelet-derived growth factor receptor beta OS=Homo sapiens OX=9606 GN=PDGFRB PE=1 SV=1	0,047277191	0,436106
Q15067	Peroxisomal acyl-coenzyme A oxidase 1 OS=Homo sapiens OX=9606 GN=ACOX1 PE=1 SV=3	0,064488612	0,435295
P62256	Ubiquitin-conjugating enzyme E2 H OS=Homo sapiens OX=9606 GN=UBE2H PE=1 SV=1	-0,112576241	0,435003
P38646	Stress-70 protein, mitochondrial OS=Homo sapiens OX=9606 GN=HSPA9 PE=1 SV=2	0,037630147	0,434289
Q96I25	Splicing factor 45 OS=Homo sapiens OX=9606 GN=RBM17 PE=1 SV=1	0,191229097	0,433876
A8MWD9	Putative small nuclear ribonucleoprotein G-like protein 15 OS=Homo sapiens OX=9606 GN=SNRPGP15 PE=5 SV=2	-0,136971063	0,432397
Q14644	Ras GTPase-activating protein 3 OS=Homo sapiens OX=9606 GN=RASA3 PE=1 SV=3	-0,136942649	0,4323
P54577	Tyrosine--tRNA ligase, cytoplasmic OS=Homo sapiens OX=9606 GN=YARS PE=1 SV=4	-0,038760312	0,432142
Q9NSK0	Kinesin light chain 4 OS=Homo sapiens OX=9606 GN=KLC4 PE=1 SV=3	0,089179851	0,432044
Q9UPU5	Ubiquitin carboxyl-terminal hydrolase 24 OS=Homo sapiens OX=9606 GN=USP24 PE=1 SV=3	0,113185587	0,431516
Q7Z3B1	Neuronal growth regulator 1 OS=Homo sapiens OX=9606 GN=NEGR1 PE=1 SV=3	-0,098913246	0,431369
P09601	Heme oxygenase 1 OS=Homo sapiens OX=9606 GN=HMOX1 PE=1 SV=1	-0,058557031	0,431264
P23921	Ribonucleoside-diphosphate reductase large subunit OS=Homo sapiens OX=9606 GN=RRM1 PE=1 SV=1	0,058536601	0,431096
P16298	#N/D	0,09870433	0,43036
Q96BP3	Peptidylprolyl isomerase domain and WD repeat-containing protein 1 OS=Homo sapiens OX=9606 GN=PPWD1 PE=1 SV=1	-0,305105155	0,430212
Q9NWZ5	Uridine-cytidine kinase-like 1 OS=Homo sapiens OX=9606 GN=UCKL1 PE=1 SV=2	0,043636204	0,429627
Q8IY67	Ribonucleoprotein PTB-binding 1 OS=Homo sapiens OX=9606 GN=RAVER1 PE=1 SV=1	0,215674866	0,429339
Q9NYP7	Elongation of very long chain fatty acids protein 5 OS=Homo sapiens OX=9606 GN=ELOVL5 PE=1 SV=1	-0,082370411	0,428705
P04114	Apolipoprotein B-100 OS=Homo sapiens OX=9606 GN=APOB PE=1 SV=2	-0,088536683	0,428585
Q9H9H4	Vacuolar protein sorting-associated protein 37B OS=Homo sapiens OX=9606 GN=VPS37B PE=1 SV=1	0,13475131	0,424782
P18084	Integrin beta-5 OS=Homo sapiens OX=9606 GN=ITGB5 PE=1 SV=1	0,042897672	0,4239
O95260	Arginyl-tRNA--protein transferase 1 OS=Homo sapiens OX=9606 GN=ATE1 PE=1 SV=2	-0,186975783	0,423826
Q96T88	E3 ubiquitin-protein ligase UHRF1 OS=Homo sapiens OX=9606 GN=UHRF1 PE=1 SV=1	0,134436678	0,423704
Q12849	G-rich sequence factor 1 OS=Homo sapiens OX=9606 GN=GRSF1 PE=1 SV=3	-0,069929658	0,422709
Q13442	28 kDa heat- and acid-stable phosphoprotein OS=Homo sapiens OX=9606 GN=PDAP1 PE=1 SV=1	-0,096955636	0,421933
Q8N7R7	Cyclin-Y-like protein 1 OS=Homo sapiens OX=9606 GN=CCNYL1 PE=1 SV=2	0,18611169	0,421784
P51159	Ras-related protein Rab-27A OS=Homo sapiens OX=9606 GN=RAB27A PE=1 SV=3	0,074560284	0,421543
P78406	mRNA export factor OS=Homo sapiens OX=9606 GN=RAE1 PE=1 SV=1	-0,096853624	0,421442
Q8WX92	Negative elongation factor B OS=Homo sapiens OX=9606 GN=NELFB PE=1 SV=1	0,185540226	0,420434
P22570	NADPH:adrenodoxin oxidoreductase, mitochondrial OS=Homo sapiens OX=9606 GN=FDXR PE=1 SV=3	-0,185464869	0,420256
Q96I99	Succinate--CoA ligase [GDP-forming] subunit beta, mitochondrial OS=Homo sapiens OX=9606 GN=SUCLG2 PE=1 SV=2	-0,065728366	0,420159
P05023	Sodium/potassium-transporting ATPase subunit alpha-1 OS=Homo sapiens OX=9606 GN=ATP1A1 PE=1 SV=1	-0,038445807	0,419706
P28062	Proteasome subunit beta type-8 OS=Homo sapiens OX=9606 GN=PSMB8 PE=1 SV=3	0,185035229	0,419241
O75462	Cytokine receptor-like factor 1 OS=Homo sapiens OX=9606 GN=CRLF1 PE=1 SV=1	0,185025331	0,419217
O43172	U4/U6 small nuclear ribonucleoprotein Prp4 OS=Homo sapiens OX=9606 GN=PRPF4 PE=1 SV=2	-0,086781112	0,419167
P21283	V-type proton ATPase subunit C 1 OS=Homo sapiens OX=9606 GN=ATP6V1C1 PE=1 SV=4	0,069311629	0,418551
Q9P2R3	Rabankyrin-5 OS=Homo sapiens OX=9606 GN=ANKFY1 PE=1 SV=2	0,065466638	0,418295
Q15847	Adipogenesis regulatory factor OS=Homo sapiens OX=9606 GN=ADIRF PE=1 SV=1	-0,109999603	0,418173
Q9Y5S1	Transient receptor potential cation channel subfamily V member 2 OS=Homo sapiens OX=9606 GN=TRPV2 PE=1 SV=1	-0,065313442	0,417205
Q9BS40	Latexin OS=Homo sapiens OX=9606 GN=LXN PE=1 SV=2	-0,184096493	0,417023
P57764	Gasdermin-D OS=Homo sapiens OX=9606 GN=GSDMD PE=1 SV=1	0,0959071	0,416893
Q96N67	Dedicator of cytokinesis protein 7 OS=Homo sapiens OX=9606 GN=DOCK7 PE=1 SV=4	-0,049120754	0,414346

P39748	Flap endonuclease 1 OS=Homo sapiens OX=9606 GN=FEN1 PE=1 SV=1	0,131671916	0,414244
Q9H6Z4	Ran-binding protein 3 OS=Homo sapiens OX=9606 GN=RANBP3 PE=1 SV=1	0,085831663	0,414089
Q9NP79	Vacuolar protein sorting-associated protein VTA1 homolog OS=Homo sapiens OX=9606 GN=VTA1 PE=1 SV=1	0,068601856	0,413784
O95674	Phosphatidate cytidylyltransferase 2 OS=Homo sapiens OX=9606 GN=CDS2 PE=1 SV=1	-0,43385045	0,412805
Q96JG6	Syndetin OS=Homo sapiens OX=9606 GN=VPS50 PE=1 SV=3	0,13098609	0,411901
Q8NEW0	Zinc transporter 7 OS=Homo sapiens OX=9606 GN=SLC30A7 PE=2 SV=1	-0,181896167	0,411824
P83916	Chromobox protein homolog 1 OS=Homo sapiens OX=9606 GN=CBX1 PE=1 SV=1	0,18178946	0,411572
Q99523	Sortilin OS=Homo sapiens OX=9606 GN=SORT1 PE=1 SV=3	-0,108293745	0,411054
Q53HC0	Coiled-coil domain-containing protein 92 OS=Homo sapiens OX=9606 GN=CCDC92 PE=1 SV=2	-0,250778142	0,410954
O95447	Lebercilin-like protein OS=Homo sapiens OX=9606 GN=LCA5L PE=1 SV=1	0,692618124	0,410722
Q9GZZ1	N-alpha-acetyltransferase 50 OS=Homo sapiens OX=9606 GN=NAA50 PE=1 SV=1	-0,130570949	0,410484
P12081	Histidine--tRNA ligase, cytoplasmic OS=Homo sapiens OX=9606 GN=HARS PE=1 SV=2	-0,042634034	0,410384
O14495	Phospholipid phosphatase 3 OS=Homo sapiens OX=9606 GN=PLPP3 PE=1 SV=1	-0,072516437	0,409975
P53367	Arfaptin-1 OS=Homo sapiens OX=9606 GN=ARFIP1 PE=1 SV=2	-0,07245011	0,409558
P45973	Chromobox protein homolog 5 OS=Homo sapiens OX=9606 GN=CBX5 PE=1 SV=1	0,130019504	0,408602
P15502	Elastin OS=Homo sapiens OX=9606 GN=ELN PE=1 SV=4	-0,067788781	0,408336
P18077	60S ribosomal protein L35a OS=Homo sapiens OX=9606 GN=RPL35A PE=1 SV=2	0,084721693	0,408165
O75150	E3 ubiquitin-protein ligase BRE1B OS=Homo sapiens OX=9606 GN=RNF40 PE=1 SV=5	-0,180244056	0,407921
O95400	CD2 antigen cytoplasmic tail-binding protein 2 OS=Homo sapiens OX=9606 GN=CD2BP2 PE=1 SV=1	-0,129817843	0,407914
Q96IX5	ATP synthase membrane subunit DAPIT, mitochondrial OS=Homo sapiens OX=9606 GN=ATP5MD PE=1 SV=1	-0,071075341	0,407735
P52701	DNA mismatch repair protein Msh6 OS=Homo sapiens OX=9606 GN=MSH6 PE=1 SV=2	0,107327012	0,407028
P16152	Carbonyl reductase [NADPH] 1 OS=Homo sapiens OX=9606 GN=CBR1 PE=1 SV=3	-0,053277204	0,405289
Q8TCE6	Protein FAM45A OS=Homo sapiens OX=9606 GN=FAM45A PE=2 SV=1	-0,178929793	0,404817
Q6P158	Putative ATP-dependent RNA helicase DHX57 OS=Homo sapiens OX=9606 GN=DHX57 PE=1 SV=2	-0,178854414	0,404639
Q86X55	Histone-arginine methyltransferase CARM1 OS=Homo sapiens OX=9606 GN=CARM1 PE=1 SV=3	-0,067175092	0,404232
Q9Y315	Deoxyribose-phosphate aldolase OS=Homo sapiens OX=9606 GN=DERA PE=1 SV=2	-0,10626737	0,402621
Q92633	Lysophosphatidic acid receptor 1 OS=Homo sapiens OX=9606 GN=LPAR1 PE=1 SV=3	0,177839379	0,402241
P56182	Ribosomal RNA processing protein 1 homolog A OS=Homo sapiens OX=9606 GN=RRP1 PE=1 SV=1	-0,177792651	0,402131
Q96EK7	Constitutive coactivator of peroxisome proliferator-activated receptor gamma OS=Homo sapiens OX=9606 GN=FAM120B PE=1 SV=1	-0,177345236	0,401074
P29320	Ephrin type-A receptor 3 OS=Homo sapiens OX=9606 GN=EPHA3 PE=1 SV=2	-0,377302765	0,400509
Q8TB52	F-box only protein 30 OS=Homo sapiens OX=9606 GN=FBXO30 PE=1 SV=3	-0,127402236	0,399683
Q8NCW5	NAD(P)H-hydrate epimerase OS=Homo sapiens OX=9606 GN=NAXE PE=1 SV=2	0,092300977	0,399633
Q96NY7	Chloride intracellular channel protein 6 OS=Homo sapiens OX=9606 GN=CLIC6 PE=2 SV=3	0,24327122	0,399598
O75884	Putative hydrolase RBBP9 OS=Homo sapiens OX=9606 GN=RBBP9 PE=1 SV=2	0,176061822	0,398043
P07305	Histone H1.0 OS=Homo sapiens OX=9606 GN=H1F0 PE=1 SV=3	-0,126733862	0,39741
Q8TDY4	Arf-GAP with SH3 domain, ANK repeat and PH domain-containing protein 3 OS=Homo sapiens OX=9606 GN=ASAP3 PE=1 SV=1	0,175532324	0,396793
Q16890	Tumor protein D53 OS=Homo sapiens OX=9606 GN=TPD52L1 PE=1 SV=1	-0,12650347	0,396626
Q99643	Succinate dehydrogenase cytochrome b560 subunit, mitochondrial OS=Homo sapiens OX=9606 GN=SDHC PE=1 SV=1	0,175448535	0,396595
Q92597	Protein NDRG1 OS=Homo sapiens OX=9606 GN=NDRG1 PE=1 SV=1	0,062372881	0,396378
P61586	Transforming protein RhoA OS=Homo sapiens OX=9606 GN=RHOA PE=1 SV=1	-0,104732659	0,396251
P62318	Small nuclear ribonucleoprotein Sm D3 OS=Homo sapiens OX=9606 GN=SNRPD3 PE=1 SV=1	-0,104687775	0,396065
Q8IXI1	Mitochondrial Rho GTPase 2 OS=Homo sapiens OX=9606 GN=RHOT2 PE=1 SV=2	0,175023998	0,395592
Q9BV44	THUMP domain-containing protein 3 OS=Homo sapiens OX=9606 GN=THUMPD3 PE=1 SV=1	0,174876423	0,395244
Q13938	Calcyphosin OS=Homo sapiens OX=9606 GN=CAPS PE=1 SV=2	-0,174623307	0,394646
P12931	Proto-oncogene tyrosine-protein kinase Src OS=Homo sapiens OX=9606 GN=SRC PE=1 SV=3	-0,174590186	0,394568
Q7Z6J0	E3 ubiquitin-protein ligase SH3RF1 OS=Homo sapiens OX=9606 GN=SH3RF1 PE=1 SV=2	0,174436494	0,394205
P62314	Small nuclear ribonucleoprotein Sm D1 OS=Homo sapiens OX=9606 GN=SNRPD1 PE=1 SV=1	0,125699904	0,393895
Q7L3B6	Hsp90 co-chaperone Cdc37-like 1 OS=Homo sapiens OX=9606 GN=CDC37L1 PE=1 SV=1	-0,174238561	0,393738
Q9HCB6	Spondin-1 OS=Homo sapiens OX=9606 GN=SPON1 PE=1 SV=2	-0,174193803	0,393632
O15056	Synaptojanin-2 OS=Homo sapiens OX=9606 GN=SYNJ2 PE=1 SV=3	-0,081965662	0,393518
P78324	Tyrosine-protein phosphatase non-receptor type substrate 1 OS=Homo sapiens OX=9606 GN=SIRPA PE=1 SV=2	0,104060756	0,393467
O95208	Epsin-2 OS=Homo sapiens OX=9606 GN=EPN2 PE=1 SV=3	0,125305128	0,392554
Q9BZL4	Protein phosphatase 1 regulatory subunit 12C OS=Homo sapiens OX=9606 GN=PPP1R12C PE=1 SV=1	-0,125191738	0,392169
Q9UK55	Protein Z-dependent protease inhibitor OS=Homo sapiens OX=9606 GN=SERPINA10 PE=1 SV=1	-0,213660251	0,391984
O94901	SUN domain-containing protein 1 OS=Homo sapiens OX=9606 GN=SUN1 PE=1 SV=3	-0,125100778	0,39186
Q8NBL1	Protein O-glucosyltransferase 1 OS=Homo sapiens OX=9606 GN=POGLUT1 PE=1 SV=1	-0,173334473	0,391603
Q15404	Ras suppressor protein 1 OS=Homo sapiens OX=9606 GN=RSU1 PE=1 SV=3	-0,05585556	0,390413
Q14247	Src substrate cortactin OS=Homo sapiens OX=9606 GN=CTTN PE=1 SV=2	0,03669987	0,390331
P46734	Dual specificity mitogen-activated protein kinase kinase 3 OS=Homo sapiens OX=9606 GN=MAP2K3 PE=1 SV=2	-0,058471702	0,390318
Q16134	Electron transfer flavoprotein-ubiquinone oxidoreductase, mitochondrial OS=Homo sapiens OX=9606 GN=ETFDH PE=1 SV=2	0,172334403	0,389242
O43432	Eukaryotic translation initiation factor 4 gamma 3 OS=Homo sapiens OX=9606 GN=EIF4G3 PE=1 SV=2	0,043824549	0,389053
Q9BZF1	Oxysterol-binding protein-related protein 8 OS=Homo sapiens OX=9606 GN=OSBPL8 PE=1 SV=3	-0,068877945	0,387245
Q9H3Q1	Cdc42 effector protein 4 OS=Homo sapiens OX=9606 GN=CDC42EP4 PE=1 SV=1	-0,214900971	0,386806
Q9Y3T9	Nucleolar complex protein 2 homolog OS=Homo sapiens OX=9606 GN=NOC2L PE=1 SV=4	-0,12344483	0,386242
Q9H4G0	Band 4.1-like protein 1 OS=Homo sapiens OX=9606 GN=EPB41L1 PE=1 SV=2	-0,123093109	0,38505
Q9H223	EH domain-containing protein 4 OS=Homo sapiens OX=9606 GN=EHD4 PE=1 SV=1	0,042240276	0,384597
Q765P7	Protein MTSS 2 OS=Homo sapiens OX=9606 GN=MTSS2 PE=1 SV=1	-0,170359709	0,384581
Q9NVG8	TBC1 domain family member 13 OS=Homo sapiens OX=9606 GN=TBC1D13 PE=1 SV=3	-0,080214143	0,384255
O00160	Unconventional myosin-If OS=Homo sapiens OX=9606 GN=MYO1F PE=1 SV=3	-0,623378301	0,384129
P30153	Serine/threonine-protein phosphatase 2A 65 kDa regulatory subunit A alpha isoform OS=Homo sapiens OX=9606 GN=PPP2R1A PE=1 SV	-0,040173257	0,384106
Q9NQ29	Putative RNA-binding protein Luc7-like 1 OS=Homo sapiens OX=9606 GN=LUC7L PE=1 SV=1	-0,10175462	0,383932
P35244	Replication protein A 14 kDa subunit OS=Homo sapiens OX=9606 GN=RPA3 PE=1 SV=1	-0,088978558	0,383834
Q99538	Legumain OS=Homo sapiens OX=9606 GN=LGMN PE=1 SV=1	0,122659744	0,383582
Q8N766	ER membrane protein complex subunit 1 OS=Homo sapiens OX=9606 GN=EMC1 PE=1 SV=1	0,04208729	0,383052
Q66PJ3	ADP-ribosylation factor-like protein 6-interacting protein 4 OS=Homo sapiens OX=9606 GN=ARL6IP4 PE=1 SV=2	0,169709516	0,383047
P42336	Phosphatidylinositol 4,5-bisphosphate 3-kinase catalytic subunit alpha isoform OS=Homo sapiens OX=9606 GN=PIK3CA PE=1 SV=2	-0,169616322	0,382827
Q9BV86	N-terminal Xaa-Pro-Lys N-methyltransferase 1 OS=Homo sapiens OX=9606 GN=NTMT1 PE=1 SV=3	0,088702254	0,382525
Q14694	Ubiquitin carboxyl-terminal hydrolase 10 OS=Homo sapiens OX=9606 GN=USP10 PE=1 SV=2	0,057405741	0,382443
P08754	Guanine nucleotide-binding protein G(i) subunit alpha OS=Homo sapiens OX=9606 GN=GNAI3 PE=1 SV=3	0,088677575	0,382408
Q9NQS1	Cell death regulator Aven OS=Homo sapiens OX=9606 GN=AVEN PE=1 SV=1	-0,169416124	0,382354
P20340	Ras-related protein Rab-6A OS=Homo sapiens OX=9606 GN=RAB6A PE=1 SV=3	0,122182641	0,381966
Q92974	Rho guanine nucleotide exchange factor 2 OS=Homo sapiens OX=9606 GN=ARHGEF2 PE=1 SV=4	0,048722746	0,381964
Q86YS6	Ras-related protein Rab-43 OS=Homo sapiens OX=9606 GN=RAB43 PE=1 SV=1	-0,117616627	0,381712
Q9Y241	HIG1 domain family member 1A, mitochondrial OS=Homo sapiens OX=9606 GN=HIGD1A PE=1 SV=1	-0,168967054	0,381294
Q9UI32	Glutaminase liver isoform, mitochondrial OS=Homo sapiens OX=9606 GN=GLS2 PE=1 SV=2	0,168904553	0,381147
Q8N6G6	ADAMTS-like protein 1 OS=Homo sapiens OX=9606 GN=ADAMTSL1 PE=1 SV=4	0,186163542	0,379883
O60645	Exocyst complex component 3 OS=Homo sapiens OX=9606 GN=EXOC3 PE=1 SV=3	0,079377556	0,379843
P09622	Dihydrolipoyl dehydrogenase, mitochondrial OS=Homo sapiens OX=9606 GN=DLD PE=1 SV=2	-0,056919551	0,378861
Q07092	Collagen alpha-1(XVI) chain OS=Homo sapiens OX=9606 GN=COL16A1 PE=1 SV=2	-0,167917246	0,378817
P23284	Peptidyl-prolyl cis-trans isomerase B OS=Homo sapiens OX=9606 GN=PPIB PE=1 SV=2	-0,043977881	0,378649
O43920	NADH dehydrogenase [ubiquinone] iron-sulfur protein 5 OS=Homo sapiens OX=9606 GN=NDUFS5 PE=1 SV=3	0,100176332	0,377425
O00622	CCN family member 1 OS=Homo sapiens OX=9606 GN=CCN1 PE=1 SV=1	-0,045123663	0,377033
O00400	Acetyl-coenzyme A transporter 1 OS=Homo sapiens OX=9606 GN=SLC33A1 PE=1 SV=1	0,120647066	0,376772
Q9NZN3	#N/D	0,06718904	0,376771
O00151	PDZ and LIM domain protein 1 OS=Homo sapiens OX=9606 GN=PDLIM1 PE=1 SV=4	-0,04981976	0,376201
Q8NFF5	FAD synthase OS=Homo sapiens OX=9606 GN=FLAD1 PE=1 SV=1	0,292466663	0,375567
P14324	Farnesyl pyrophosphate synthase OS=Homo sapiens OX=9606 GN=FDPS PE=1 SV=4	-0,099664872	0,37532
Q9HCE1	Helicase MOV-10 OS=Homo sapiens OX=9606 GN=MOV10 PE=1 SV=2	0,066884402	0,374886

O95865	N(G),N(G)-dimethylarginine dimethylaminohydrolase 2 OS=Homo sapiens OX=9606 GN=DDAH2 PE=1 SV=1	-0,05637782	0,374876
Q6P587	Acylpyruvase FAHD1, mitochondrial OS=Homo sapiens OX=9606 GN=FAHD1 PE=1 SV=2	0,120070532	0,374824
O75821	Eukaryotic translation initiation factor 3 subunit G OS=Homo sapiens OX=9606 GN=EIF3G PE=1 SV=2	0,051512477	0,373967
Q96KB5	Lymphokine-activated killer T-cell-originated protein kinase OS=Homo sapiens OX=9606 GN=PBK PE=1 SV=3	0,086845626	0,373744
Q9Y4K4	Mitogen-activated protein kinase kinase kinase kinase 5 OS=Homo sapiens OX=9606 GN=MAP4K5 PE=1 SV=2	0,062450091	0,372881
Q15637	Splicing factor 1 OS=Homo sapiens OX=9606 GN=SF1 PE=1 SV=4	0,056058805	0,372533
P09972	Fructose-bisphosphate aldolase C OS=Homo sapiens OX=9606 GN=ALDOC PE=1 SV=2	-0,165236152	0,372493
Q9H583	HEAT repeat-containing protein 1 OS=Homo sapiens OX=9606 GN=HEATR1 PE=1 SV=3	0,119316367	0,372277
Q5VTL8	Pre-mRNA-splicing factor 38B OS=Homo sapiens OX=9606 GN=PRPF38B PE=1 SV=1	-0,134082668	0,37191
Q6IQ22	Ras-related protein Rab-12 OS=Homo sapiens OX=9606 GN=RAB12 PE=1 SV=3	-0,164939744	0,371794
O15400	Syntaxin-7 OS=Homo sapiens OX=9606 GN=STX7 PE=1 SV=4	-0,066201561	0,370669
Q9NRG4	N-lysine methyltransferase SMYD2 OS=Homo sapiens OX=9606 GN=SMYD2 PE=1 SV=2	-0,164389426	0,370496
Q16775	Hydroxyacylglutathione hydrolase, mitochondrial OS=Homo sapiens OX=9606 GN=HAGH PE=1 SV=2	0,077475248	0,369842
Q9UNS2	COP9 signalosome complex subunit 3 OS=Homo sapiens OX=9606 GN=COPS3 PE=1 SV=3	0,055657712	0,36959
Q8NDA8	Maestro heat-like repeat-containing protein family member 1 OS=Homo sapiens OX=9606 GN=MROH1 PE=2 SV=3	-0,163823213	0,36916
P09001	39S ribosomal protein L3, mitochondrial OS=Homo sapiens OX=9606 GN=MRPL3 PE=1 SV=1	0,118295976	0,368834
Q9UBF2	Coatomer subunit gamma-2 OS=Homo sapiens OX=9606 GN=COPG2 PE=1 SV=1	-0,085737688	0,368519
Q9NZJ4	Sacsin OS=Homo sapiens OX=9606 GN=SACS PE=1 SV=2	-0,16342318	0,368217
Q8WVY7	Ubiquitin-like domain-containing CTD phosphatase 1 OS=Homo sapiens OX=9606 GN=UBLCP1 PE=1 SV=2	-0,085655256	0,368131
Q9UHX1	Poly(U)-binding-splicing factor PUF60 OS=Homo sapiens OX=9606 GN=PUF60 PE=1 SV=1	0,058289874	0,367776
P22059	Oxysterol-binding protein 1 OS=Homo sapiens OX=9606 GN=OSBP PE=1 SV=1	-0,061655278	0,36765
O00267	Transcription elongation factor SPT5 OS=Homo sapiens OX=9606 GN=SUPT5H PE=1 SV=1	0,070665794	0,367439
Q9Y2Q3	Glutathione S-transferase kappa 1 OS=Homo sapiens OX=9606 GN=GSTK1 PE=1 SV=3	0,061543862	0,366918
P61225	Ras-related protein Rap-2b OS=Homo sapiens OX=9606 GN=RAP2B PE=1 SV=1	0,16237933	0,365756
Q9C0C2	182 kDa tankyrase-1-binding protein OS=Homo sapiens OX=9606 GN=TNKS1BP1 PE=1 SV=4	0,030283151	0,365214
P39656	Dolichyl-diphosphooligosaccharide--protein glycosyltransferase 48 kDa subunit OS=Homo sapiens OX=9606 GN=DDOST PE=1 SV=4	0,048459383	0,364862
P22234	Multifunctional protein ADE2 OS=Homo sapiens OX=9606 GN=PAICS PE=1 SV=3	-0,045197078	0,364802
Q8TCS8	Polyribonucleotide nucleotidyltransferase 1, mitochondrial OS=Homo sapiens OX=9606 GN=PNPT1 PE=1 SV=2	-0,117042546	0,364611
Q9BRR6	ADP-dependent glucokinase OS=Homo sapiens OX=9606 GN=ADPGK PE=1 SV=1	-0,084884416	0,364502
O95793	Double-stranded RNA-binding protein Staufen homolog 1 OS=Homo sapiens OX=9606 GN=STAU1 PE=1 SV=2	0,057713091	0,363765
A0AVT1	Ubiquitin-like modifier-activating enzyme 6 OS=Homo sapiens OX=9606 GN=UBA6 PE=1 SV=1	0,032626319	0,363635
P51149	Ras-related protein Rab-7a OS=Homo sapiens OX=9606 GN=RAB7A PE=1 SV=1	-0,052316742	0,363147
Q9NXR1	#N/D	0,081179962	0,361846
Q9H1E5	Thioredoxin-related transmembrane protein 4 OS=Homo sapiens OX=9606 GN=TMX4 PE=1 SV=1	-0,160538456	0,361417
P51114	Fragile X mental retardation syndrome-related protein 1 OS=Homo sapiens OX=9606 GN=FXR1 PE=1 SV=3	0,040984503	0,361231
Q9BW91	ADP-ribose pyrophosphatase, mitochondrial OS=Homo sapiens OX=9606 GN=NUDT9 PE=1 SV=1	0,115847502	0,360589
Q15125	3-beta-hydroxysteroid-Delta(8),Delta(7)-isomerase OS=Homo sapiens OX=9606 GN=EBP PE=1 SV=3	-0,115839714	0,360563
O15126	Secretory carrier-associated membrane protein 1 OS=Homo sapiens OX=9606 GN=SCAMP1 PE=1 SV=2	-0,095884254	0,359811
P62136	Serine/threonine-protein phosphatase PP1-alpha catalytic subunit OS=Homo sapiens OX=9606 GN=PPP1CA PE=1 SV=1	0,041968901	0,359567
Q5ZPR3	CD276 antigen OS=Homo sapiens OX=9606 GN=CD276 PE=1 SV=1	-0,075353831	0,358739
P05186	Alkaline phosphatase, tissue-nonspecific isozyme OS=Homo sapiens OX=9606 GN=ALPL PE=1 SV=4	-0,095533344	0,358377
P07585	#N/D	0,189157006	0,357873
P14868	Aspartate--tRNA ligase, cytoplasmic OS=Homo sapiens OX=9606 GN=DARS PE=1 SV=2	0,032156677	0,357855
Q9Y6M7	Sodium bicarbonate cotransporter 3 OS=Homo sapiens OX=9606 GN=SLC4A7 PE=1 SV=2	-0,083452333	0,357776
Q9NRY6	Phospholipid scramblase 3 OS=Homo sapiens OX=9606 GN=PLSCR3 PE=1 SV=2	0,114941848	0,357545
P33240	Cleavage stimulation factor subunit 2 OS=Homo sapiens OX=9606 GN=CSTF2 PE=1 SV=1	0,068882819	0,357262
Q86X76	Deaminated glutathione amidase OS=Homo sapiens OX=9606 GN=NIT1 PE=1 SV=2	-0,08325447	0,356848
Q6NUM9	All-trans-retinol 13,14-reductase OS=Homo sapiens OX=9606 GN=RETSAT PE=1 SV=2	0,068764684	0,356589
Q9UNH7	Sorting nexin-6 OS=Homo sapiens OX=9606 GN=SNX6 PE=1 SV=1	-0,04280992	0,355728
Q71UM5	40S ribosomal protein S27-like OS=Homo sapiens OX=9606 GN=RPS27L PE=1 SV=3	0,114301309	0,355393
Q5VYS4	Mesenteric estrogen-dependent adipogenesis protein OS=Homo sapiens OX=9606 GN=MEDAG PE=2 SV=1	-0,09437517	0,353646
P40925	Malate dehydrogenase, cytoplasmic OS=Homo sapiens OX=9606 GN=MDH1 PE=1 SV=4	-0,047007209	0,352825
P62910	60S ribosomal protein L32 OS=Homo sapiens OX=9606 GN=RPL32 PE=1 SV=2	0,067993347	0,352202
Q96GX9	Methylthioribulose-1-phosphate dehydratase OS=Homo sapiens OX=9606 GN=APIP PE=1 SV=1	0,158218609	0,351098
P61077	Ubiquitin-conjugating enzyme E2 D3 OS=Homo sapiens OX=9606 GN=UBE2D3 PE=1 SV=1	0,112995095	0,351011
O00541	Pescadillo homolog OS=Homo sapiens OX=9606 GN=PES1 PE=1 SV=1	0,112822399	0,350432
P0CG08	Golgi pH regulator B OS=Homo sapiens OX=9606 GN=GPR89B PE=1 SV=1	-0,112800403	0,350358
Q9P2W9	Syntaxin-18 OS=Homo sapiens OX=9606 GN=STX18 PE=1 SV=1	-0,155635765	0,349869
Q08752	Peptidyl-prolyl cis-trans isomerase D OS=Homo sapiens OX=9606 GN=PPID PE=1 SV=3	0,093444599	0,349852
Q9NRN5	Olfactomedin-like protein 3 OS=Homo sapiens OX=9606 GN=OLFML3 PE=2 SV=1	0,185337247	0,349807
Q08J23	tRNA (cytosine(34)-C(5))-methyltransferase OS=Homo sapiens OX=9606 GN=NSUN2 PE=1 SV=2	0,058920447	0,349746
Q9Y6D6	Brefeldin A-inhibited guanine nucleotide-exchange protein 1 OS=Homo sapiens OX=9606 GN=ARFGEF1 PE=1 SV=2	-0,093361833	0,349515
P22307	Non-specific lipid-transfer protein OS=Homo sapiens OX=9606 GN=SCP2 PE=1 SV=2	0,048388565	0,349033
P0C0S5	Histone H2A.Z OS=Homo sapiens OX=9606 GN=H2AFZ PE=1 SV=2	0,081509291	0,34868
Q13085	Acetyl-CoA carboxylase 1 OS=Homo sapiens OX=9606 GN=ACACA PE=1 SV=2	0,081496274	0,348619
O60486	Plexin-C1 OS=Homo sapiens OX=9606 GN=PLXNC1 PE=1 SV=1	0,154272043	0,346659
Q53H96	Pyrroline-5-carboxylate reductase 3 OS=Homo sapiens OX=9606 GN=PYCR3 PE=1 SV=3	-0,154240676	0,346585
P36639	7,8-dihydro-8-oxoguanine triphosphatase OS=Homo sapiens OX=9606 GN=NUDT1 PE=1 SV=3	0,111609575	0,346369
Q16513	Serine/threonine-protein kinase N2 OS=Homo sapiens OX=9606 GN=PKN2 PE=1 SV=1	0,06696273	0,346353
P05141	ADP/ATP translocase 2 OS=Homo sapiens OX=9606 GN=SLC25A5 PE=1 SV=7	0,06693992	0,346224
Q9UMR2	ATP-dependent RNA helicase DDX19B OS=Homo sapiens OX=9606 GN=DDX19B PE=1 SV=1	0,044536515	0,345898
Q9P283	Semaphorin-5B OS=Homo sapiens OX=9606 GN=SEMA5B PE=2 SV=4	0,153867502	0,345706
Q658Y4	Protein FAM91A1 OS=Homo sapiens OX=9606 GN=FAM91A1 PE=1 SV=3	-0,092362291	0,345446
Q5JRA6	Transport and Golgi organization protein 1 homolog OS=Homo sapiens OX=9606 GN=MIA3 PE=1 SV=1	0,05498876	0,344922
O76021	Ribosomal L1 domain-containing protein 1 OS=Homo sapiens OX=9606 GN=RSL1D1 PE=1 SV=3	0,047837466	0,344665
Q15019	Septin-2 OS=Homo sapiens OX=9606 GN=SEPTIN2 PE=1 SV=1	-0,040317094	0,344007
P11233	Ras-related protein Ral-A OS=Homo sapiens OX=9606 GN=RALA PE=1 SV=1	0,080470436	0,343831
Q96C19	EF-hand domain-containing protein D2 OS=Homo sapiens OX=9606 GN=EFHD2 PE=1 SV=1	0,057947316	0,343411
P60866	40S ribosomal protein S20 OS=Homo sapiens OX=9606 GN=RPS20 PE=1 SV=1	0,091818025	0,343233
P53041	Serine/threonine-protein phosphatase 5 OS=Homo sapiens OX=9606 GN=PPP5C PE=1 SV=1	-0,049693874	0,34316
Q9BXJ4	Complement C1q tumor necrosis factor-related protein 3 OS=Homo sapiens OX=9606 GN=C1QTNF3 PE=1 SV=1	0,152559781	0,342629
Q9H1B7	#N/D	-0,35987303	0,342427
Q6ZMT4	Lysine-specific demethylase 7A OS=Homo sapiens OX=9606 GN=KDM7A PE=1 SV=2	0,35202097	0,342337
Q8TAE6	Protein phosphatase 1 regulatory subunit 14C OS=Homo sapiens OX=9606 GN=PPP1R14C PE=1 SV=3	-0,15238777	0,342225
Q13201	Multimerin-1 OS=Homo sapiens OX=9606 GN=MMRN1 PE=1 SV=3	0,180374942	0,342059
Q9NXR7	BRISC and BRCA1-A complex member 2 OS=Homo sapiens OX=9606 GN=BABAM2 PE=1 SV=2	0,110309427	0,342019
Q99614	Tetratricopeptide repeat protein 1 OS=Homo sapiens OX=9606 GN=TTC1 PE=1 SV=1	0,066126486	0,341619
Q9BVP2	Guanine nucleotide-binding protein-like 3 OS=Homo sapiens OX=9606 GN=GNL3 PE=1 SV=2	0,079858292	0,340979
Q9BRG1	Vacuolar protein-sorting-associated protein 25 OS=Homo sapiens OX=9606 GN=VPS25 PE=1 SV=1	-0,0798084	0,340746
Q5BKZ1	DBIRD complex subunit ZNF326 OS=Homo sapiens OX=9606 GN=ZNF326 PE=1 SV=2	0,091148753	0,340515
P82909	28S ribosomal protein S36, mitochondrial OS=Homo sapiens OX=9606 GN=MRPS36 PE=1 SV=2	-0,151503549	0,340145
Q13948	Protein CASP OS=Homo sapiens OX=9606 GN=CUX1 PE=1 SV=2	0,109729081	0,340079
P56945	Breast cancer anti-estrogen resistance protein 1 OS=Homo sapiens OX=9606 GN=BCAR1 PE=1 SV=2	-0,051604092	0,340076
P17174	Aspartate aminotransferase, cytoplasmic OS=Homo sapiens OX=9606 GN=GOT1 PE=1 SV=3	0,041047995	0,339648
P18859	ATP synthase-coupling factor 6, mitochondrial OS=Homo sapiens OX=9606 GN=ATP5PF PE=1 SV=1	-0,071652831	0,339496
Q9P0J1	[Pyruvate dehydrogenase [acetyl-transferring]]-phosphatase 1, mitochondrial OS=Homo sapiens OX=9606 GN=PDP1 PE=1 SV=3	-0,151136479	0,339281
Q96EY1	DnaJ homolog subfamily A member 3, mitochondrial OS=Homo sapiens OX=9606 GN=DNAJA3 PE=1 SV=2	-0,109474215	0,339228
Q9UNX3	60S ribosomal protein L26-like 1 OS=Homo sapiens OX=9606 GN=RPL26L1 PE=1 SV=1	-0,066765743	0,338823

Q9UIF9	Bromodomain adjacent to zinc finger domain protein 2A OS=Homo sapiens OX=9606 GN=BAZ2A PE=1 SV=4	-0,34707914	0,338352
Q8WYA6	Beta-catenin-like protein 1 OS=Homo sapiens OX=9606 GN=CTNNBL1 PE=1 SV=1	0,150690874	0,338233
P62266	40S ribosomal protein S23 OS=Homo sapiens OX=9606 GN=RPS23 PE=1 SV=3	0,078992843	0,336951
P54886	Delta-1-pyrroline-5-carboxylate synthase OS=Homo sapiens OX=9606 GN=ALDH18A1 PE=1 SV=2	-0,032693916	0,336276
Q8NF91	Nesprin-1 OS=Homo sapiens OX=9606 GN=SYNE1 PE=1 SV=4	0,026936191	0,336091
P53384	Cytosolic Fe-S cluster assembly factor NUBP1 OS=Homo sapiens OX=9606 GN=NUBP1 PE=1 SV=2	-0,07097708	0,336
Q5T447	E3 ubiquitin-protein ligase HECTD3 OS=Homo sapiens OX=9606 GN=HECTD3 PE=1 SV=1	0,149427123	0,335262
A2RRP1	Neuroblastoma-amplified sequence OS=Homo sapiens OX=9606 GN=NBAS PE=1 SV=2	0,044835593	0,334952
Q13126	S-methyl-5'-thioadenosine phosphorylase OS=Homo sapiens OX=9606 GN=MTAP PE=1 SV=2	-0,050813119	0,334364
Q96IZ0	PRKC apoptosis WT1 regulator protein OS=Homo sapiens OX=9606 GN=PAWR PE=1 SV=1	0,053374455	0,333834
Q9BRZ2	E3 ubiquitin-protein ligase TRIM56 OS=Homo sapiens OX=9606 GN=TRIM56 PE=1 SV=3	-0,025779433	0,333399
Q12768	WASH complex subunit 5 OS=Homo sapiens OX=9606 GN=WASHC5 PE=1 SV=1	0,050676157	0,333376
P05156	Complement factor I OS=Homo sapiens OX=9606 GN=CFI PE=1 SV=2	0,107505444	0,33266
O75352	Mannose-P-dolichol utilization defect 1 protein OS=Homo sapiens OX=9606 GN=MPDU1 PE=1 SV=2	-0,089194944	0,332596
O75348	V-type proton ATPase subunit G 1 OS=Homo sapiens OX=9606 GN=ATP6V1G1 PE=1 SV=3	-0,089104163	0,332229
Q9NZI8	#N/D	-0,056415429	0,332095
Q00059	Transcription factor A, mitochondrial OS=Homo sapiens OX=9606 GN=TFAM PE=1 SV=1	0,147964768	0,331825
Q9Y5L4	Mitochondrial import inner membrane translocase subunit Tim13 OS=Homo sapiens OX=9606 GN=TIMM13 PE=1 SV=1	-0,147839546	0,33153
Q9UI09	NADH dehydrogenase [ubiquinone] 1 alpha subcomplex subunit 12 OS=Homo sapiens OX=9606 GN=NDUFA12 PE=1 SV=1	-0,147741637	0,3313
Q9H089	Large subunit GTPase 1 homolog OS=Homo sapiens OX=9606 GN=LSG1 PE=1 SV=2	0,147532854	0,33081
P11766	Alcohol dehydrogenase class-3 OS=Homo sapiens OX=9606 GN=ADH5 PE=1 SV=4	0,044274201	0,330357
O75688	Protein phosphatase 1B OS=Homo sapiens OX=9606 GN=PPM1B PE=1 SV=1	-0,137041244	0,330157
Q15121	Astrocytic phosphoprotein PEA-15 OS=Homo sapiens OX=9606 GN=PEA15 PE=1 SV=2	-0,064079023	0,33007
Q8WUA2	Peptidyl-prolyl cis-trans isomerase-like 4 OS=Homo sapiens OX=9606 GN=PPIL4 PE=1 SV=1	0,147185034	0,329993
P27361	Mitogen-activated protein kinase 3 OS=Homo sapiens OX=9606 GN=MAPK3 PE=1 SV=4	-0,047933678	0,329852
Q9P2D3	HEAT repeat-containing protein 5B OS=Homo sapiens OX=9606 GN=HEATR5B PE=1 SV=2	-0,106489375	0,329276
Q86XL3	Ankyrin repeat and LEM domain-containing protein 2 OS=Homo sapiens OX=9606 GN=ANKLE2 PE=1 SV=4	-0,063818428	0,328605
P37108	Signal recognition particle 14 kDa protein OS=Homo sapiens OX=9606 GN=SRP14 PE=1 SV=2	0,077157488	0,328434
Q9P0V9	Septin-10 OS=Homo sapiens OX=9606 GN=SEPTIN10 PE=1 SV=2	-0,087859854	0,327199
Q9UJY1	Heat shock protein beta-8 OS=Homo sapiens OX=9606 GN=HSPB8 PE=1 SV=1	0,087748799	0,326751
Q7Z4F1	Low-density lipoprotein receptor-related protein 10 OS=Homo sapiens OX=9606 GN=LRP10 PE=1 SV=2	-0,148960874	0,326537
Q9H7Z6	Histone acetyltransferase KAT8 OS=Homo sapiens OX=9606 GN=KAT8 PE=1 SV=2	0,145696178	0,326495
O60488	Long-chain-fatty-acid--CoA ligase 4 OS=Homo sapiens OX=9606 GN=ACSL4 PE=1 SV=2	0,055284018	0,326167
Q14155	Rho guanine nucleotide exchange factor 7 OS=Homo sapiens OX=9606 GN=ARHGEF7 PE=1 SV=2	0,076355005	0,32472
Q9UBB4	Ataxin-10 OS=Homo sapiens OX=9606 GN=ATXN10 PE=1 SV=1	-0,047139565	0,323876
Q8WUD1	Ras-related protein Rab-2B OS=Homo sapiens OX=9606 GN=RAB2B PE=1 SV=1	-0,062883828	0,323357
Q12797	Aspartyl/asparaginyl beta-hydroxylase OS=Homo sapiens OX=9606 GN=ASPH PE=1 SV=3	0,038012729	0,322494
Q6GMV3	Putative peptidyl-tRNA hydrolase PTRHD1 OS=Homo sapiens OX=9606 GN=PTRHD1 PE=1 SV=1	-0,14389282	0,322261
Q9BQB6	Vitamin K epoxide reductase complex subunit 1 OS=Homo sapiens OX=9606 GN=VKORC1 PE=1 SV=1	0,104153934	0,321512
P62847	40S ribosomal protein S24 OS=Homo sapiens OX=9606 GN=RPS24 PE=1 SV=1	-0,075614014	0,321295
O75506	Heat shock factor-binding protein 1 OS=Homo sapiens OX=9606 GN=HSBP1 PE=1 SV=1	-0,086363057	0,321162
Q9NYL9	Tropomodulin-3 OS=Homo sapiens OX=9606 GN=TMOD3 PE=1 SV=1	0,041596997	0,320941
Q13445	Transmembrane emp24 domain-containing protein 1 OS=Homo sapiens OX=9606 GN=TMED1 PE=1 SV=1	-0,103919368	0,320733
Q99729	Heterogeneous nuclear ribonucleoprotein A/B OS=Homo sapiens OX=9606 GN=HNRNPAB PE=1 SV=2	0,067938378	0,320347
Q8TDJ6	DmX-like protein 2 OS=Homo sapiens OX=9606 GN=DMXL2 PE=1 SV=2	-0,228716288	0,320313
Q9Y3Q8	TSC22 domain family protein 4 OS=Homo sapiens OX=9606 GN=TSC22D4 PE=1 SV=2	0,143039834	0,320259
P51784	Ubiquitin carboxyl-terminal hydrolase 11 OS=Homo sapiens OX=9606 GN=USP11 PE=1 SV=3	0,103744272	0,320152
Q9BTY7	Protein HGH1 homolog OS=Homo sapiens OX=9606 GN=HGH1 PE=1 SV=1	0,140198914	0,319143
Q9NSY0	Nuclear receptor-binding protein 2 OS=Homo sapiens OX=9606 GN=NRBP2 PE=1 SV=2	0,089950115	0,31883
Q96ST2	Protein IWS1 homolog OS=Homo sapiens OX=9606 GN=IWS1 PE=1 SV=2	-0,103287776	0,318638
Q7Z4G1	COMM domain-containing protein 6 OS=Homo sapiens OX=9606 GN=COMMD6 PE=1 SV=1	0,142292658	0,318506
Q96RT7	Gamma-tubulin complex component 6 OS=Homo sapiens OX=9606 GN=TUBGCP6 PE=1 SV=3	0,318291295	0,3185
P63220	40S ribosomal protein S21 OS=Homo sapiens OX=9606 GN=RPS21 PE=1 SV=1	0,057341766	0,316664
Q2TAL8	Glutamine-rich protein 1 OS=Homo sapiens OX=9606 GN=QRICH1 PE=1 SV=1	-0,141505133	0,316659
P04406	Glyceraldehyde-3-phosphate dehydrogenase OS=Homo sapiens OX=9606 GN=GAPDH PE=1 SV=3	0,030247318	0,315389
O75400	Pre-mRNA-processing factor 40 homolog A OS=Homo sapiens OX=9606 GN=PRPF40A PE=1 SV=2	-0,074329312	0,31537
P62736	Actin, aortic smooth muscle OS=Homo sapiens OX=9606 GN=ACTA2 PE=1 SV=1	0,140942228	0,315338
P24844	Myosin regulatory light polypeptide 9 OS=Homo sapiens OX=9606 GN=MYL9 PE=1 SV=4	0,039528353	0,314869
P36873	Serine/threonine-protein phosphatase PP1-gamma catalytic subunit OS=Homo sapiens OX=9606 GN=PPP1CC PE=1 SV=1	-0,102141709	0,314839
Q7L7X3	Serine/threonine-protein kinase TAO1 OS=Homo sapiens OX=9606 GN=TAOK1 PE=1 SV=1	0,084537076	0,313818
Q9NYY8	FAST kinase domain-containing protein 2, mitochondrial OS=Homo sapiens OX=9606 GN=FASTKD2 PE=1 SV=1	-0,206387133	0,313445
Q6XZF7	Dynamin-binding protein OS=Homo sapiens OX=9606 GN=DNMBP PE=1 SV=1	-0,140108079	0,313383
P49840	Glycogen synthase kinase-3 alpha OS=Homo sapiens OX=9606 GN=GSK3A PE=1 SV=2	-0,084377823	0,313179
P82094	TATA element modulatory factor OS=Homo sapiens OX=9606 GN=TMF1 PE=1 SV=2	0,066355686	0,312237
Q9UPN3	Microtubule-actin cross-linking factor 1, isoforms 1/2/3/5 OS=Homo sapiens OX=9606 GN=MACF1 PE=1 SV=4	0,021474963	0,312024
Q13336	Urea transporter 1 OS=Homo sapiens OX=9606 GN=SLC14A1 PE=1 SV=2	0,139225043	0,311313
Q6YHU6	Thyroid adenoma-associated protein OS=Homo sapiens OX=9606 GN=THADA PE=1 SV=1	-0,0734417	0,311286
Q12904	Aminoacyl tRNA synthase complex-interacting multifunctional protein 1 OS=Homo sapiens OX=9606 GN=AIMP1 PE=1 SV=2	0,052829671	0,3104
Q9Y263	Phospholipase A-2-activating protein OS=Homo sapiens OX=9606 GN=PLAA PE=1 SV=2	0,036683449	0,310187
Q9Y6R0	Numb-like protein OS=Homo sapiens OX=9606 GN=NUMBL PE=1 SV=1	-0,22933951	0,309923
P53680	AP-2 complex subunit sigma OS=Homo sapiens OX=9606 GN=AP2S1 PE=1 SV=2	-0,083500615	0,30966
Q86YR5	G-protein-signaling modulator 1 OS=Homo sapiens OX=9606 GN=GPSM1 PE=1 SV=2	-0,13831544	0,309181
P13798	Acylamino-acid-releasing enzyme OS=Homo sapiens OX=9606 GN=APEH PE=1 SV=4	-0,038841845	0,308904
P00966	Argininosuccinate synthase OS=Homo sapiens OX=9606 GN=ASS1 PE=1 SV=2	-0,038803389	0,30857
P54920	Alpha-soluble NSF attachment protein OS=Homo sapiens OX=9606 GN=NAPA PE=1 SV=3	0,043234347	0,308533
Q12772	Sterol regulatory element-binding protein 2 OS=Homo sapiens OX=9606 GN=SREBF2 PE=1 SV=2	0,473875629	0,308176
O43639	Cytoplasmic protein NCK2 OS=Homo sapiens OX=9606 GN=NCK2 PE=1 SV=2	-0,23889105	0,307768
Q7RTV0	PHD finger-like domain-containing protein 5A OS=Homo sapiens OX=9606 GN=PHF5A PE=1 SV=1	-0,072604278	0,307439
P49366	Deoxyhypusine synthase OS=Homo sapiens OX=9606 GN=DHPS PE=1 SV=1	-0,137495993	0,307261
P36954	DNA-directed RNA polymerase II subunit RPB9 OS=Homo sapiens OX=9606 GN=POLR2I PE=1 SV=1	0,137460892	0,307179
Q00688	Peptidyl-prolyl cis-trans isomerase FKBP3 OS=Homo sapiens OX=9606 GN=FKBP3 PE=1 SV=1	0,055757706	0,30715
P30626	Sorcin OS=Homo sapiens OX=9606 GN=SRI PE=1 SV=1	0,052264657	0,306787
P83436	Conserved oligomeric Golgi complex subunit 7 OS=Homo sapiens OX=9606 GN=COG7 PE=1 SV=1	-0,065118807	0,305921
Q9H3P7	Golgi resident protein GCP60 OS=Homo sapiens OX=9606 GN=ACBD3 PE=1 SV=4	-0,041261554	0,30587
Q14683	Structural maintenance of chromosomes protein 1A OS=Homo sapiens OX=9606 GN=SMC1A PE=1 SV=2	0,072161526	0,305407
P20810	Calpastatin OS=Homo sapiens OX=9606 GN=CAST PE=1 SV=4	0,036136304	0,305143
P52565	Rho GDP-dissociation inhibitor 1 OS=Homo sapiens OX=9606 GN=ARHGDIA PE=1 SV=3	-0,041151941	0,304985
P07738	Bisphosphoglycerate mutase OS=Homo sapiens OX=9606 GN=BPGM PE=1 SV=2	-0,05537508	0,304858
O15164	Transcription intermediary factor 1-alpha OS=Homo sapiens OX=9606 GN=TRIM24 PE=1 SV=3	-0,173070245	0,303893
Q16082	Heat shock protein beta-2 OS=Homo sapiens OX=9606 GN=HSPB2 PE=1 SV=2	-0,13571382	0,303088
Q8N3D4	EH domain-binding protein 1-like protein 1 OS=Homo sapiens OX=9606 GN=EHBP1L1 PE=1 SV=2	0,044244917	0,302238
Q16832	Discoidin domain-containing receptor 2 OS=Homo sapiens OX=9606 GN=DDR2 PE=1 SV=2	0,081585222	0,301993
P12236	ADP/ATP translocase 3 OS=Homo sapiens OX=9606 GN=SLC25A6 PE=1 SV=4	0,03387848	0,30177
Q9BVA1	Tubulin beta-2B chain OS=Homo sapiens OX=9606 GN=TUBB2B PE=1 SV=1	-0,088201731	0,301556
P06132	Uroporphyrinogen decarboxylase OS=Homo sapiens OX=9606 GN=UROD PE=1 SV=2	-0,048584726	0,301276
P30419	Glycylpeptide N-tetradecanoyltransferase 1 OS=Homo sapiens OX=9606 GN=NMT1 PE=1 SV=2	-0,051396181	0,301246
Q9UI15	Transgelin-3 OS=Homo sapiens OX=9606 GN=TAGLN3 PE=1 SV=2	0,081174855	0,300354
O15344	E3 ubiquitin-protein ligase Midline-1 OS=Homo sapiens OX=9606 GN=MID1 PE=1 SV=1	0,058746996	0,300281

Q9NSC5	Homer protein homolog 3 OS=Homo sapiens OX=9606 GN=HOMER3 PE=1 SV=2	0,070883298	0,299554
P50914	60S ribosomal protein L14 OS=Homo sapiens OX=9606 GN=RPL14 PE=1 SV=4	-0,097457342	0,299366
Q14980	Nuclear mitotic apparatus protein 1 OS=Homo sapiens OX=9606 GN=NUMA1 PE=1 SV=2	0,031241078	0,298739
Q13435	Splicing factor 3B subunit 2 OS=Homo sapiens OX=9606 GN=SF3B2 PE=1 SV=2	-0,037545298	0,297686
Q9BXR0	Queuine tRNA-ribosyltransferase catalytic subunit 1 OS=Homo sapiens OX=9606 GN=QTRT1 PE=1 SV=3	-0,133310117	0,297463
P55290	Cadherin-13 OS=Homo sapiens OX=9606 GN=CDH13 PE=1 SV=1	0,070419362	0,297433
Q03701	CCAAT/enhancer-binding protein zeta OS=Homo sapiens OX=9606 GN=CEBPZ PE=1 SV=3	-0,133185682	0,297172
Q96GA7	Serine dehydratase-like OS=Homo sapiens OX=9606 GN=SDSL PE=1 SV=1	-0,080321061	0,296947
O94966	Ubiquitin carboxyl-terminal hydrolase 19 OS=Homo sapiens OX=9606 GN=USP19 PE=1 SV=2	-0,046391645	0,296741
Q7Z5R6	Amyloid beta A4 precursor protein-binding family B member 1-interacting protein OS=Homo sapiens OX=9606 GN=APBB1IP PE=1 SV=1	0,318485376	0,296178
Q9H008	Phospholysine phosphohistidine inorganic pyrophosphate phosphatase OS=Homo sapiens OX=9606 GN=LHPP PE=1 SV=2	-0,132718957	0,296081
P78504	Protein jagged-1 OS=Homo sapiens OX=9606 GN=JAG1 PE=1 SV=3	-0,132435582	0,295418
Q02809	Procollagen-lysine,2-oxoglutarate 5-dioxygenase 1 OS=Homo sapiens OX=9606 GN=PLOD1 PE=1 SV=2	-0,026548554	0,295404
Q96EY8	Corrinoid adenosyltransferase OS=Homo sapiens OX=9606 GN=MMAB PE=1 SV=1	0,108874695	0,293285
O60869	Endothelial differentiation-related factor 1 OS=Homo sapiens OX=9606 GN=EDF1 PE=1 SV=1	0,062596033	0,293094
Q12965	Unconventional myosin-Ie OS=Homo sapiens OX=9606 GN=MYO1E PE=1 SV=2	-0,032151748	0,292959
O43719	HIV Tat-specific factor 1 OS=Homo sapiens OX=9606 GN=HTATSF1 PE=1 SV=1	-0,095385902	0,292552
P36969	Phospholipid hydroperoxide glutathione peroxidase OS=Homo sapiens OX=9606 GN=GPX4 PE=1 SV=3	-0,130836015	0,291679
Q15714	TSC22 domain family protein 1 OS=Homo sapiens OX=9606 GN=TSC22D1 PE=1 SV=3	0,037461812	0,290592
P56211	cAMP-regulated phosphoprotein 19 OS=Homo sapiens OX=9606 GN=ARPP19 PE=1 SV=2	0,094750234	0,290464
P22061	Protein-L-isoaspartate(D-aspartate) O-methyltransferase OS=Homo sapiens OX=9606 GN=PCMT1 PE=1 SV=4	0,044610115	0,29011
O43852	Calumenin OS=Homo sapiens OX=9606 GN=CALU PE=1 SV=2	-0,031826421	0,289738
P33991	DNA replication licensing factor MCM4 OS=Homo sapiens OX=9606 GN=MCM4 PE=1 SV=5	-0,046859998	0,289678
Q9H777	Zinc phosphodiesterase ELAC protein 1 OS=Homo sapiens OX=9606 GN=ELAC1 PE=1 SV=2	0,173144974	0,288838
O43488	Aflatoxin B1 aldehyde reductase member 2 OS=Homo sapiens OX=9606 GN=AKR7A2 PE=1 SV=3	-0,049392816	0,288521
Q5BJH2	Transmembrane protein 128 OS=Homo sapiens OX=9606 GN=TMEM128 PE=1 SV=2	-0,128798141	0,286919
P47755	F-actin-capping protein subunit alpha-2 OS=Homo sapiens OX=9606 GN=CAPZA2 PE=1 SV=3	0,042158936	0,286788
Q92621	Nuclear pore complex protein Nup205 OS=Homo sapiens OX=9606 GN=NUP205 PE=1 SV=3	-0,04636344	0,286351
P30622	CAP-Gly domain-containing linker protein 1 OS=Homo sapiens OX=9606 GN=CLIP1 PE=1 SV=2	-0,029981027	0,28566
P36871	Phosphoglucomutase-1 OS=Homo sapiens OX=9606 GN=PGM1 PE=1 SV=3	-0,028549141	0,283729
Q96AN5	Transmembrane protein 143 OS=Homo sapiens OX=9606 GN=TMEM143 PE=2 SV=1	0,12738231	0,283614
Q03405	Urokinase plasminogen activator surface receptor OS=Homo sapiens OX=9606 GN=PLAUR PE=1 SV=1	-0,067124573	0,282429
Q13444	Disintegrin and metalloproteinase domain-containing protein 15 OS=Homo sapiens OX=9606 GN=ADAM15 PE=1 SV=4	0,126691176	0,282002
O95479	GDH/6PGL endoplasmic bifunctional protein OS=Homo sapiens OX=9606 GN=H6PD PE=1 SV=2	0,036899903	0,281689
Q16763	Ubiquitin-conjugating enzyme E2 S OS=Homo sapiens OX=9606 GN=UBE2S PE=1 SV=2	0,119056431	0,281251
P49821	NADH dehydrogenase [ubiquinone] flavoprotein 1, mitochondrial OS=Homo sapiens OX=9606 GN=NDUFV1 PE=1 SV=4	0,066811912	0,281011
Q9H814	Phosphorylated adapter RNA export protein OS=Homo sapiens OX=9606 GN=PHAX PE=1 SV=1	0,086675133	0,280849
Q99622	Protein C10 OS=Homo sapiens OX=9606 GN=C12orf57 PE=1 SV=1	-0,060106441	0,280511
P08631	Tyrosine-protein kinase HCK OS=Homo sapiens OX=9606 GN=HCK PE=1 SV=5	-0,017404726	0,280201
P40818	Ubiquitin carboxyl-terminal hydrolase 8 OS=Homo sapiens OX=9606 GN=USP8 PE=1 SV=1	0,05997716	0,27986
Q15126	Phosphomevalonate kinase OS=Homo sapiens OX=9606 GN=PMVK PE=1 SV=3	-0,055030142	0,279761
Q8NCM8	Cytoplasmic dynein 2 heavy chain 1 OS=Homo sapiens OX=9606 GN=DYNC2H1 PE=1 SV=4	0,125724514	0,279747
Q96Q11	CCA tRNA nucleotidyltransferase 1, mitochondrial OS=Homo sapiens OX=9606 GN=TRNT1 PE=1 SV=2	-0,091429733	0,279585
Q9H773	dCTP pyrophosphatase 1 OS=Homo sapiens OX=9606 GN=DCTPP1 PE=1 SV=1	0,091360698	0,279359
Q8IWE4	DCN1-like protein 3 OS=Homo sapiens OX=9606 GN=DCUN1D3 PE=1 SV=1	-0,075819125	0,279063
Q9H1H9	Kinesin-like protein KIF13A OS=Homo sapiens OX=9606 GN=KIF13A PE=1 SV=2	-0,125146236	0,278399
Q9NZQ7	Programmed cell death 1 ligand 1 OS=Homo sapiens OX=9606 GN=CD274 PE=1 SV=1	0,499618671	0,278289
P17812	CTP synthase 1 OS=Homo sapiens OX=9606 GN=CTPS1 PE=1 SV=2	-0,030630582	0,277944
Q8WU79	Stromal membrane-associated protein 2 OS=Homo sapiens OX=9606 GN=SMAP2 PE=1 SV=1	-0,090841914	0,277664
P50750	Cyclin-dependent kinase 9 OS=Homo sapiens OX=9606 GN=CDK9 PE=1 SV=3	0,124619983	0,277172
P03891	NADH-ubiquinone oxidoreductase chain 2 OS=Homo sapiens OX=9606 GN=MT-ND2 PE=1 SV=2	0,124153627	0,276085
Q6YN16	Hydroxysteroid dehydrogenase-like protein 2 OS=Homo sapiens OX=9606 GN=HSDL2 PE=1 SV=1	0,042605447	0,276013
Q9Y5A7	NEDD8 ultimate buster 1 OS=Homo sapiens OX=9606 GN=NUB1 PE=1 SV=2	-0,090304953	0,27591
Q6NZI2	Caveolae-associated protein 1 OS=Homo sapiens OX=9606 GN=CAVIN1 PE=1 SV=1	0,033924777	0,275909
Q9BYV8	Centrosomal protein of 41 kDa OS=Homo sapiens OX=9606 GN=CEP41 PE=1 SV=1	-0,124068813	0,275887
O96019	Actin-like protein 6A OS=Homo sapiens OX=9606 GN=ACTL6A PE=1 SV=1	0,050492427	0,275835
Q5MIZ7	Serine/threonine-protein phosphatase 4 regulatory subunit 3B OS=Homo sapiens OX=9606 GN=PPP4R3B PE=1 SV=2	-0,252234243	0,275243
O14653	Golgi SNAP receptor complex member 2 OS=Homo sapiens OX=9606 GN=GOSR2 PE=1 SV=2	-0,074520569	0,273931
Q9NY33	Dipeptidyl peptidase 3 OS=Homo sapiens OX=9606 GN=DPP3 PE=1 SV=2	-0,030971446	0,273757
Q9NRX1	RNA-binding protein PNO1 OS=Homo sapiens OX=9606 GN=PNO1 PE=1 SV=1	0,08960933	0,27364
Q92805	Golgin subfamily A member 1 OS=Homo sapiens OX=9606 GN=GOLGA1 PE=1 SV=3	0,089494638	0,273266
Q9NR46	Endophilin-B2 OS=Homo sapiens OX=9606 GN=SH3GLB2 PE=1 SV=1	0,089439439	0,273086
P08865	40S ribosomal protein SA OS=Homo sapiens OX=9606 GN=RPSA PE=1 SV=4	0,03355294	0,272637
Q96EK9	Protein KTI12 homolog OS=Homo sapiens OX=9606 GN=KTI12 PE=1 SV=1	-0,124248627	0,271542
Q16647	Prostacyclin synthase OS=Homo sapiens OX=9606 GN=PTGIS PE=1 SV=1	-0,064642307	0,271193
P48556	26S proteasome non-ATPase regulatory subunit 8 OS=Homo sapiens OX=9606 GN=PSMD8 PE=1 SV=2	-0,046638599	0,271156
P17301	Integrin alpha-2 OS=Homo sapiens OX=9606 GN=ITGA2 PE=1 SV=1	0,122017316	0,271109
Q9NPI6	mRNA-decapping enzyme 1A OS=Homo sapiens OX=9606 GN=DCP1A PE=1 SV=3	0,121845573	0,270709
Q5T2E6	Armadillo-like helical domain-containing protein 3 OS=Homo sapiens OX=9606 GN=ARMH3 PE=1 SV=1	0,073639317	0,270454
Q05519	Serine/arginine-rich splicing factor 11 OS=Homo sapiens OX=9606 GN=SRSF11 PE=1 SV=1	-0,064448014	0,270316
Q0VG06	Fanconi anemia core complex-associated protein 100 OS=Homo sapiens OX=9606 GN=FAAP100 PE=1 SV=3	0,12117268	0,269143
P50416	Carnitine O-palmitoyltransferase 1, liver isoform OS=Homo sapiens OX=9606 GN=CPT1A PE=1 SV=2	0,04370821	0,268654
P05556	Integrin beta-1 OS=Homo sapiens OX=9606 GN=ITGB1 PE=1 SV=2	0,024733262	0,268642
Q9H788	SH2 domain-containing protein 4A OS=Homo sapiens OX=9606 GN=SH2D4A PE=1 SV=1	0,064031472	0,268437
Q8IU85	Calcium/calmodulin-dependent protein kinase type 1D OS=Homo sapiens OX=9606 GN=CAMK1D PE=1 SV=1	-0,087755596	0,2676
P31930	Cytochrome b-c1 complex subunit 1, mitochondrial OS=Homo sapiens OX=9606 GN=UQCRC1 PE=1 SV=3	-0,034984831	0,265906
O75915	PRA1 family protein 3 OS=Homo sapiens OX=9606 GN=ARL6IP5 PE=1 SV=1	0,048782339	0,265768
O00762	Ubiquitin-conjugating enzyme E2 C OS=Homo sapiens OX=9606 GN=UBE2C PE=1 SV=1	-0,087010101	0,265175
Q9UMX0	Ubiquilin-1 OS=Homo sapiens OX=9606 GN=UBQLN1 PE=1 SV=2	0,041052475	0,26516
Q9Y6A9	Signal peptidase complex subunit 1 OS=Homo sapiens OX=9606 GN=SPCS1 PE=1 SV=4	0,119426133	0,26508
P28340	DNA polymerase delta catalytic subunit OS=Homo sapiens OX=9606 GN=POLD1 PE=1 SV=2	-0,086586264	0,264928
Q14254	Flotillin-2 OS=Homo sapiens OX=9606 GN=FLOT2 PE=1 SV=2	0,045595089	0,264616
P49755	Transmembrane emp24 domain-containing protein 10 OS=Homo sapiens OX=9606 GN=TMED10 PE=1 SV=2	0,052246096	0,264523
O75489	NADH dehydrogenase [ubiquinone] iron-sulfur protein 3, mitochondrial OS=Homo sapiens OX=9606 GN=NDUFS3 PE=1 SV=1	-0,063116955	0,264318
P84098	60S ribosomal protein L19 OS=Homo sapiens OX=9606 GN=RPL19 PE=1 SV=1	0,056764429	0,263738
P42166	Lamina-associated polypeptide 2, isoform alpha OS=Homo sapiens OX=9606 GN=TMPO PE=1 SV=2	0,045448528	0,263699
O43731	ER lumen protein-retaining receptor 3 OS=Homo sapiens OX=9606 GN=KDELR3 PE=2 SV=1	0,118446219	0,262802
O75438	NADH dehydrogenase [ubiquinone] 1 beta subcomplex subunit 1 OS=Homo sapiens OX=9606 GN=NDUFB1 PE=1 SV=1	-0,118388327	0,262668
Q9NVD7	Alpha-parvin OS=Homo sapiens OX=9606 GN=PARVA PE=1 SV=1	0,045231397	0,262342
Q9Y2D5	A-kinase anchor protein 2 OS=Homo sapiens OX=9606 GN=AKAP2 PE=1 SV=3	-0,028351123	0,262333
P40938	Replication factor C subunit 3 OS=Homo sapiens OX=9606 GN=RFC3 PE=1 SV=2	-0,118198262	0,262226
Q9UBL3	Set1/Ash2 histone methyltransferase complex subunit ASH2 OS=Homo sapiens OX=9606 GN=ASH2L PE=1 SV=1	0,062606043	0,26202
O60506	Heterogeneous nuclear ribonucleoprotein Q OS=Homo sapiens OX=9606 GN=SYNCRIP PE=1 SV=2	-0,032329047	0,261908
Q13617	Cullin-2 OS=Homo sapiens OX=9606 GN=CUL2 PE=1 SV=2	0,040530536	0,261526
Q5JTJ3	Cytochrome c oxidase assembly factor 6 homolog OS=Homo sapiens OX=9606 GN=COA6 PE=1 SV=1	0,117765231	0,26122
P60228	Eukaryotic translation initiation factor 3 subunit E OS=Homo sapiens OX=9606 GN=EIF3E PE=1 SV=1	-0,030437968	0,261104
Q9H7C4	Syncoilin OS=Homo sapiens OX=9606 GN=SYNC PE=1 SV=3	0,04038111	0,260487
P11279	Lysosome-associated membrane glycoprotein 1 OS=Homo sapiens OX=9606 GN=LAMP1 PE=1 SV=3	-0,056065407	0,260247

Q6ZRP7	Sulfhydryl oxidase 2 OS=Homo sapiens OX=9606 GN=QSOX2 PE=1 SV=3	-0,11727846	0,260089
P19634	Sodium/hydrogen exchanger 1 OS=Homo sapiens OX=9606 GN=SLC9A1 PE=1 SV=2	-0,116293399	0,257802
P48060	Glioma pathogenesis-related protein 1 OS=Homo sapiens OX=9606 GN=GLIPR1 PE=1 SV=3	-0,084722634	0,257748
Q8IUR7	Armadillo repeat-containing protein 8 OS=Homo sapiens OX=9606 GN=ARMC8 PE=1 SV=2	-0,116046855	0,257229
P43487	Ran-specific GTPase-activating protein OS=Homo sapiens OX=9606 GN=RANBP1 PE=1 SV=1	0,050900289	0,257198
P82932	28S ribosomal protein S6, mitochondrial OS=Homo sapiens OX=9606 GN=MRPS6 PE=1 SV=3	-0,084414995	0,256751
P55011	Solute carrier family 12 member 2 OS=Homo sapiens OX=9606 GN=SLC12A2 PE=1 SV=1	0,084395685	0,256689
A8K2U0	Alpha-2-macroglobulin-like protein 1 OS=Homo sapiens OX=9606 GN=A2ML1 PE=1 SV=3	-0,115808754	0,256676
Q15642	Cdc42-interacting protein 4 OS=Homo sapiens OX=9606 GN=TRIP10 PE=1 SV=3	0,070114123	0,256601
P62280	40S ribosomal protein S11 OS=Homo sapiens OX=9606 GN=RPS11 PE=1 SV=3	-0,037994358	0,256298
O75083	WD repeat-containing protein 1 OS=Homo sapiens OX=9606 GN=WDR1 PE=1 SV=4	-0,024492418	0,255915
P53004	Biliverdin reductase A OS=Homo sapiens OX=9606 GN=BLVRA PE=1 SV=2	0,041767577	0,25582
Q9NP72	Ras-related protein Rab-18 OS=Homo sapiens OX=9606 GN=RAB18 PE=1 SV=1	0,047078714	0,255788
Q9NRF9	DNA polymerase epsilon subunit 3 OS=Homo sapiens OX=9606 GN=POLE3 PE=1 SV=1	-0,115421994	0,255779
Q9NTK5	Obg-like ATPase 1 OS=Homo sapiens OX=9606 GN=OLA1 PE=1 SV=2	0,033686812	0,255282
Q8IWX8	Calcium homeostasis endoplasmic reticulum protein OS=Homo sapiens OX=9606 GN=CHERP PE=1 SV=3	-0,115125349	0,25509
P60903	Protein S100-A10 OS=Homo sapiens OX=9606 GN=S100A10 PE=1 SV=2	0,050501588	0,255033
P23381	Tryptophan--tRNA ligase, cytoplasmic OS=Homo sapiens OX=9606 GN=WARS PE=1 SV=2	-0,02969635	0,254252
Q9NQT8	Kinesin-like protein KIF13B OS=Homo sapiens OX=9606 GN=KIF13B PE=1 SV=2	0,015978856	0,253849
Q96TA1	Niban-like protein 1 OS=Homo sapiens OX=9606 GN=FAM129B PE=1 SV=3	0,02270591	0,253792
P06730	Eukaryotic translation initiation factor 4E OS=Homo sapiens OX=9606 GN=EIF4E PE=1 SV=2	0,050157621	0,253167
Q0JRZ9	F-BAR domain only protein 2 OS=Homo sapiens OX=9606 GN=FCHO2 PE=1 SV=2	0,083067988	0,25239
O60503	Adenylate cyclase type 9 OS=Homo sapiens OX=9606 GN=ADCY9 PE=1 SV=4	-0,113915281	0,252283
Q16222	UDP-N-acetylhexosamine pyrophosphorylase OS=Homo sapiens OX=9606 GN=UAP1 PE=1 SV=3	-0,026707933	0,252097
Q15042	Rab3 GTPase-activating protein catalytic subunit OS=Homo sapiens OX=9606 GN=RAB3GAP1 PE=1 SV=3	0,030211442	0,251343
Q99627	COP9 signalosome complex subunit 8 OS=Homo sapiens OX=9606 GN=COPS8 PE=1 SV=1	0,068629298	0,250791
Q12788	Transducin beta-like protein 3 OS=Homo sapiens OX=9606 GN=TBL3 PE=1 SV=2	-0,082289663	0,249874
Q6PD62	RNA polymerase-associated protein CTR9 homolog OS=Homo sapiens OX=9606 GN=CTR9 PE=1 SV=1	0,096844075	0,249
Q7RTP6	[F-actin]-monooxygenase MICAL3 OS=Homo sapiens OX=9606 GN=MICAL3 PE=1 SV=2	-0,06811417	0,248779
Q6ZSZ5	#N/D	0,027999307	0,248041
O76074	cGMP-specific 3',5'-cyclic phosphodiesterase OS=Homo sapiens OX=9606 GN=PDE5A PE=1 SV=2	-0,111872983	0,24755
Q12996	Cleavage stimulation factor subunit 3 OS=Homo sapiens OX=9606 GN=CSTF3 PE=1 SV=1	0,059151998	0,246551
P30520	Adenylosuccinate synthetase isozyme 2 OS=Homo sapiens OX=9606 GN=ADSS PE=1 SV=3	0,036550204	0,245836
O94851	[F-actin]-monooxygenase MICAL2 OS=Homo sapiens OX=9606 GN=MICAL2 PE=1 SV=1	0,032468819	0,245367
Q9Y6E0	Serine/threonine-protein kinase 24 OS=Homo sapiens OX=9606 GN=STK24 PE=1 SV=1	0,053047456	0,245242
P46940	Ras GTPase-activating-like protein IQGAP1 OS=Homo sapiens OX=9606 GN=IQGAP1 PE=1 SV=1	0,013918065	0,245059
Q9UI14	Prenylated Rab acceptor protein 1 OS=Homo sapiens OX=9606 GN=RABAC1 PE=1 SV=1	-0,110748638	0,244946
Q9UKG1	DCC-interacting protein 13-alpha OS=Homo sapiens OX=9606 GN=APPL1 PE=1 SV=1	0,06708908	0,244781
Q8N129	Protein canopy homolog 4 OS=Homo sapiens OX=9606 GN=CNPY4 PE=2 SV=1	0,110423155	0,244193
Q8TED1	Probable glutathione peroxidase 8 OS=Homo sapiens OX=9606 GN=GPX8 PE=1 SV=2	0,048429689	0,243821
Q9NRL3	Striatin-4 OS=Homo sapiens OX=9606 GN=STRN4 PE=1 SV=2	-0,080390976	0,243746
P02794	Ferritin heavy chain OS=Homo sapiens OX=9606 GN=FTH1 PE=1 SV=2	0,066800679	0,243658
Q9P287	BRCA2 and CDKN1A-interacting protein OS=Homo sapiens OX=9606 GN=BCCIP PE=1 SV=1	-0,110041811	0,24331
P23142	Fibulin-1 OS=Homo sapiens OX=9606 GN=FBLN1 PE=1 SV=4	0,033377376	0,243182
P78362	#N/D	-0,114720685	0,242644
Q8WUW1	Protein BRICK1 OS=Homo sapiens OX=9606 GN=BRK1 PE=1 SV=1	-0,052444431	0,242257
Q16658	Fascin OS=Homo sapiens OX=9606 GN=FSCN1 PE=1 SV=3	-0,023258521	0,242067
P41091	Eukaryotic translation initiation factor 2 subunit 3 OS=Homo sapiens OX=9606 GN=EIF2S3 PE=1 SV=3	-0,026942697	0,242024
P49721	Proteasome subunit beta type-2 OS=Homo sapiens OX=9606 GN=PSMB2 PE=1 SV=1	-0,037644086	0,241552
Q9Y3B2	Exosome complex component CSL4 OS=Homo sapiens OX=9606 GN=EXOSC1 PE=1 SV=1	-0,079593466	0,241176
Q92541	RNA polymerase-associated protein RTF1 homolog OS=Homo sapiens OX=9606 GN=RTF1 PE=1 SV=4	-0,051388695	0,240774
Q92882	Osteoclast-stimulating factor 1 OS=Homo sapiens OX=9606 GN=OSTF1 PE=1 SV=2	0,057759158	0,240346
Q16610	Extracellular matrix protein 1 OS=Homo sapiens OX=9606 GN=ECM1 PE=1 SV=2	-0,108665784	0,240127
Q9P2K8	eIF-2-alpha kinase GCN2 OS=Homo sapiens OX=9606 GN=EIF2AK4 PE=1 SV=3	0,108662812	0,24012
Q13257	Mitotic spindle assembly checkpoint protein MAD2A OS=Homo sapiens OX=9606 GN=MAD2L1 PE=1 SV=1	0,108436624	0,239597
P29401	Transketolase OS=Homo sapiens OX=9606 GN=TKT PE=1 SV=3	-0,020853995	0,239404
Q96EI5	Transcription elongation factor A protein-like 4 OS=Homo sapiens OX=9606 GN=TCEAL4 PE=1 SV=2	-0,108327092	0,239344
Q7Z422	SUZ domain-containing protein 1 OS=Homo sapiens OX=9606 GN=SZRD1 PE=1 SV=1	-0,108193721	0,239036
Q969Z0	FAST kinase domain-containing protein 4 OS=Homo sapiens OX=9606 GN=TBRG4 PE=1 SV=1	-0,078903664	0,238956
Q13449	Limbic system-associated membrane protein OS=Homo sapiens OX=9606 GN=LSAMP PE=1 SV=2	0,078577891	0,237909
Q8IWV8	E3 ubiquitin-protein ligase UBR2 OS=Homo sapiens OX=9606 GN=UBR2 PE=1 SV=1	-0,095872214	0,236392
Q13526	Peptidyl-prolyl cis-trans isomerase NIMA-interacting 1 OS=Homo sapiens OX=9606 GN=PIN1 PE=1 SV=1	0,064891255	0,236235
Q9UQN3	Charged multivesicular body protein 2b OS=Homo sapiens OX=9606 GN=CHMP2B PE=1 SV=1	-0,106823454	0,235869
Q14318	Peptidyl-prolyl cis-trans isomerase FKBP8 OS=Homo sapiens OX=9606 GN=FKBP8 PE=1 SV=2	0,038689766	0,235637
P04732	Metallothionein-1E OS=Homo sapiens OX=9606 GN=MT1E PE=1 SV=1	0,106717012	0,235623
P56199	Integrin alpha-1 OS=Homo sapiens OX=9606 GN=ITGA1 PE=1 SV=2	-0,077864945	0,235617
P62330	ADP-ribosylation factor 6 OS=Homo sapiens OX=9606 GN=ARF6 PE=1 SV=2	0,051031222	0,23528
P09936	Ubiquitin carboxyl-terminal hydrolase isozyme L1 OS=Homo sapiens OX=9606 GN=UCHL1 PE=1 SV=2	-0,038592356	0,235002
P60484	Phosphatidylinositol 3,4,5-trisphosphate 3-phosphatase and dual-specificity protein phosphatase PTEN OS=Homo sapiens OX=9606 GN=	0,106427939	0,234956
Q8N3P4	Vacuolar protein sorting-associated protein 8 homolog OS=Homo sapiens OX=9606 GN=VPS8 PE=1 SV=3	0,106329533	0,234728
P61966	AP-1 complex subunit sigma-1A OS=Homo sapiens OX=9606 GN=AP1S1 PE=1 SV=1	-0,05638696	0,23425
Q9UJW0	Dynactin subunit 4 OS=Homo sapiens OX=9606 GN=DCTN4 PE=1 SV=1	-0,036541822	0,233979
P15924	Desmoplakin OS=Homo sapiens OX=9606 GN=DSP PE=1 SV=3	0,011433706	0,233734
Q16644	MAP kinase-activated protein kinase 3 OS=Homo sapiens OX=9606 GN=MAPKAPK3 PE=1 SV=1	0,102814635	0,233713
Q13895	Bystin OS=Homo sapiens OX=9606 GN=BYSL PE=1 SV=3	0,077184104	0,233431
Q9ULT8	E3 ubiquitin-protein ligase HECTD1 OS=Homo sapiens OX=9606 GN=HECTD1 PE=1 SV=3	0,03212626	0,23342
Q9P0S9	Transmembrane protein 14C OS=Homo sapiens OX=9606 GN=TMEM14C PE=1 SV=1	0,105228019	0,232185
Q17RY0	#N/D	-0,108667734	0,231903
Q53GS9	U4/U6.U5 tri-snRNP-associated protein 2 OS=Homo sapiens OX=9606 GN=USP39 PE=1 SV=2	-0,05581908	0,231733
O00401	Neural Wiskott-Aldrich syndrome protein OS=Homo sapiens OX=9606 GN=WASL PE=1 SV=2	0,076559961	0,231429
Q9UM00	Calcium load-activated calcium channel OS=Homo sapiens OX=9606 GN=TMCO1 PE=1 SV=2	0,063562731	0,231085
Q9NVH1	DnaJ homolog subfamily C member 11 OS=Homo sapiens OX=9606 GN=DNAJC11 PE=1 SV=2	-0,055631997	0,230905
P26358	DNA (cytosine-5)-methyltransferase 1 OS=Homo sapiens OX=9606 GN=DNMT1 PE=1 SV=2	-0,045866653	0,230038
Q9BZZ5	Apoptosis inhibitor 5 OS=Homo sapiens OX=9606 GN=API5 PE=1 SV=3	-0,042634394	0,22999
P84095	Rho-related GTP-binding protein RhoG OS=Homo sapiens OX=9606 GN=RHOG PE=1 SV=1	-0,049953974	0,229978
Q9NR56	Muscleblind-like protein 1 OS=Homo sapiens OX=9606 GN=MBNL1 PE=1 SV=2	0,045795334	0,229656
P21912	Succinate dehydrogenase [ubiquinone] iron-sulfur subunit, mitochondrial OS=Homo sapiens OX=9606 GN=SDHB PE=1 SV=3	-0,039852145	0,229009
Q9HBL0	Tensin-1 OS=Homo sapiens OX=9606 GN=TNS1 PE=1 SV=2	-0,018656736	0,228933
Q9NQG5	Regulation of nuclear pre-mRNA domain-containing protein 1B OS=Homo sapiens OX=9606 GN=RPRD1B PE=1 SV=1	-0,075736347	0,228789
P62851	40S ribosomal protein S25 OS=Homo sapiens OX=9606 GN=RPS25 PE=1 SV=1	0,075723435	0,228748
Q5H9R7	Serine/threonine-protein phosphatase 6 regulatory subunit 3 OS=Homo sapiens OX=9606 GN=PPP6R3 PE=1 SV=2	-0,045595711	0,228587
Q9H7B2	Ribosome production factor 2 homolog OS=Homo sapiens OX=9606 GN=RPF2 PE=1 SV=2	-0,103590308	0,228408
Q9Y2U8	Inner nuclear membrane protein Man1 OS=Homo sapiens OX=9606 GN=LEMD3 PE=1 SV=2	-0,130823539	0,228155
Q8TB61	Adenosine 3'-phospho 5'-phosphosulfate transporter 1 OS=Homo sapiens OX=9606 GN=SLC35B2 PE=1 SV=1	-0,054961008	0,227936
A0A1B0GUX0	Protein ATP6V1FNB OS=Homo sapiens OX=9606 GN=ATP6V1FNB PE=4 SV=1	0,329929651	0,227856
Q14790	Caspase-8 OS=Homo sapiens OX=9606 GN=CASP8 PE=1 SV=1	-0,103313128	0,227768
O15173	Membrane-associated progesterone receptor component 2 OS=Homo sapiens OX=9606 GN=PGRMC2 PE=1 SV=1	0,045419656	0,227644
Q9H6K4	Optic atrophy 3 protein OS=Homo sapiens OX=9606 GN=OPA3 PE=1 SV=1	-0,102973571	0,226986

Q9H490	Phosphatidylinositol glycan anchor biosynthesis class U protein OS=Homo sapiens OX=9606 GN=PIGU PE=1 SV=3	0,102967598	0,226972
O15228	Dihydroxyacetone phosphate acyltransferase OS=Homo sapiens OX=9606 GN=GNPAT PE=1 SV=1	0,075133983	0,22686
P35573	Glycogen debranching enzyme OS=Homo sapiens OX=9606 GN=AGL PE=1 SV=3	0,102803538	0,226594
Q01628	Interferon-induced transmembrane protein 3 OS=Homo sapiens OX=9606 GN=IFITM3 PE=1 SV=2	-0,102640024	0,226217
Q14764	Major vault protein OS=Homo sapiens OX=9606 GN=MVP PE=1 SV=4	0,017759369	0,226074
P13987	CD59 glycoprotein OS=Homo sapiens OX=9606 GN=CD59 PE=1 SV=1	0,062220781	0,225895
Q07812	Apoptosis regulator BAX OS=Homo sapiens OX=9606 GN=BAX PE=1 SV=1	-0,045087981	0,22587
Q96PC5	Melanoma inhibitory activity protein 2 OS=Homo sapiens OX=9606 GN=MIA2 PE=1 SV=4	-0,102340831	0,225528
Q6Y288	Beta-1,3-glucosyltransferase OS=Homo sapiens OX=9606 GN=B3GLCT PE=1 SV=2	-0,102303913	0,225443
P23528	Cofilin-1 OS=Homo sapiens OX=9606 GN=CFL1 PE=1 SV=3	0,028999785	0,225328
Q9P1T7	MyoD family inhibitor domain-containing protein OS=Homo sapiens OX=9606 GN=MDFIC PE=1 SV=2	0,102254085	0,225328
P52815	39S ribosomal protein L12, mitochondrial OS=Homo sapiens OX=9606 GN=MRPL12 PE=1 SV=2	-0,101956556	0,224642
P61247	40S ribosomal protein S3a OS=Homo sapiens OX=9606 GN=RPS3A PE=1 SV=2	0,025130467	0,224622
P00846	ATP synthase subunit a OS=Homo sapiens OX=9606 GN=MT-ATP6 PE=1 SV=1	-0,101891657	0,224493
Q13017	Rho GTPase-activating protein 5 OS=Homo sapiens OX=9606 GN=ARHGAP5 PE=1 SV=2	-0,061724017	0,223978
Q14974	Importin subunit beta-1 OS=Homo sapiens OX=9606 GN=KPNB1 PE=1 SV=2	0,019009192	0,223633
Q96PE2	Rho guanine nucleotide exchange factor 17 OS=Homo sapiens OX=9606 GN=ARHGEF17 PE=1 SV=1	-0,033418483	0,223344
P49736	DNA replication licensing factor MCM2 OS=Homo sapiens OX=9606 GN=MCM2 PE=1 SV=4	0,036726449	0,222873
Q9UKK3	Protein mono-ADP-ribosyltransferase PARP4 OS=Homo sapiens OX=9606 GN=PARP4 PE=1 SV=3	0,033311078	0,222578
Q99650	Oncostatin-M-specific receptor subunit beta OS=Homo sapiens OX=9606 GN=OSMR PE=1 SV=1	0,101015577	0,222476
Q9BXV9	EKC/KEOPS complex subunit GON7 OS=Homo sapiens OX=9606 GN=GON7 PE=1 SV=2	-0,100916791	0,222248
P62191	26S proteasome regulatory subunit 4 OS=Homo sapiens OX=9606 GN=PSMC1 PE=1 SV=1	0,027708375	0,221942
P18440	Arylamine N-acetyltransferase 1 OS=Homo sapiens OX=9606 GN=NAT1 PE=1 SV=2	0,100664188	0,221667
P52732	Kinesin-like protein KIF11 OS=Homo sapiens OX=9606 GN=KIF11 PE=1 SV=2	-0,100429379	0,221126
P50213	Isocitrate dehydrogenase [NAD] subunit alpha, mitochondrial OS=Homo sapiens OX=9606 GN=IDH3A PE=1 SV=1	0,041081535	0,221057
Q9BWU0	Kanadaptin OS=Homo sapiens OX=9606 GN=SLC4A1AP PE=1 SV=1	-0,100334598	0,220908
Q14118	Dystroglycan OS=Homo sapiens OX=9606 GN=DAG1 PE=1 SV=2	0,044088114	0,220531
Q9BX67	Junctional adhesion molecule C OS=Homo sapiens OX=9606 GN=JAM3 PE=1 SV=1	0,077029458	0,219686
Q9NXW2	DnaJ homolog subfamily B member 12 OS=Homo sapiens OX=9606 GN=DNAJB12 PE=1 SV=5	-0,072743292	0,219221
Q9Y265	RuvB-like 1 OS=Homo sapiens OX=9606 GN=RUVBL1 PE=1 SV=1	0,03280734	0,218986
P49773	Histidine triad nucleotide-binding protein 1 OS=Homo sapiens OX=9606 GN=HINT1 PE=1 SV=2	0,04767781	0,218821
Q13177	Serine/threonine-protein kinase PAK 2 OS=Homo sapiens OX=9606 GN=PAK2 PE=1 SV=3	0,025133169	0,218709
P20742	Pregnancy zone protein OS=Homo sapiens OX=9606 GN=PZP PE=1 SV=4	0,099358976	0,218664
Q15428	Splicing factor 3A subunit 2 OS=Homo sapiens OX=9606 GN=SF3A2 PE=1 SV=2	0,052610752	0,217571
Q09161	Nuclear cap-binding protein subunit 1 OS=Homo sapiens OX=9606 GN=NCBP1 PE=1 SV=1	0,035855015	0,217236
Q9NZ56	Formin-2 OS=Homo sapiens OX=9606 GN=FMN2 PE=1 SV=4	-0,098683156	0,21711
Q8TDN6	Ribosome biogenesis protein BRX1 homolog OS=Homo sapiens OX=9606 GN=BRIX1 PE=1 SV=2	-0,072005373	0,216869
Q9Y5N5	Methyltransferase N6AMT1 OS=Homo sapiens OX=9606 GN=N6AMT1 PE=1 SV=4	0,068071862	0,21637
Q86SF2	N-acetylgalactosaminyltransferase 7 OS=Homo sapiens OX=9606 GN=GALNT7 PE=1 SV=1	0,040242362	0,216247
P13716	Delta-aminolevulinic acid dehydratase OS=Homo sapiens OX=9606 GN=ALAD PE=1 SV=1	-0,047053927	0,215774
Q9NX40	OCIA domain-containing protein 1 OS=Homo sapiens OX=9606 GN=OCIAD1 PE=1 SV=1	-0,039934374	0,214484
Q9NSE4	Isoleucine--tRNA ligase, mitochondrial OS=Homo sapiens OX=9606 GN=IARS2 PE=1 SV=2	-0,027633559	0,214013
P32119	Peroxiredoxin-2 OS=Homo sapiens OX=9606 GN=PRDX2 PE=1 SV=5	-0,033609146	0,213978
O60725	Protein-S-isoprenylcysteine O-methyltransferase OS=Homo sapiens OX=9606 GN=ICMT PE=1 SV=1	0,096999487	0,213242
O95084	Serine protease 23 OS=Homo sapiens OX=9606 GN=PRSS23 PE=1 SV=1	-0,096995341	0,213232
Q13505	Metaxin-1 OS=Homo sapiens OX=9606 GN=MTX1 PE=1 SV=3	-0,070760267	0,212904
Q13148	TAR DNA-binding protein 43 OS=Homo sapiens OX=9606 GN=TARDBP PE=1 SV=1	-0,037097977	0,212166
Q9Y5A9	YTH domain-containing family protein 2 OS=Homo sapiens OX=9606 GN=YTHDF2 PE=1 SV=2	0,046267074	0,211938
O95299	NADH dehydrogenase [ubiquinone] 1 alpha subcomplex subunit 10, mitochondrial OS=Homo sapiens OX=9606 GN=NDUFA10 PE=1 SV=:	-0,058508227	0,211606
O75351	Vacuolar protein sorting-associated protein 4B OS=Homo sapiens OX=9606 GN=VPS4B PE=1 SV=2	0,051227076	0,211494
Q6ZMI0	Protein phosphatase 1 regulatory subunit 21 OS=Homo sapiens OX=9606 GN=PPP1R21 PE=1 SV=1	0,127507582	0,211078
P08621	U1 small nuclear ribonucleoprotein 70 kDa OS=Homo sapiens OX=9606 GN=SNRNP70 PE=1 SV=2	-0,030365951	0,210886
Q5VYS8	Terminal uridylyltransferase 7 OS=Homo sapiens OX=9606 GN=TUT7 PE=1 SV=1	0,105662524	0,210267
Q96Q42	Alsin OS=Homo sapiens OX=9606 GN=ALS2 PE=1 SV=2	-0,106259531	0,210109
Q14141	Septin-6 OS=Homo sapiens OX=9606 GN=SEPTIN6 PE=1 SV=4	0,050747989	0,209394
Q15435	Protein phosphatase 1 regulatory subunit 7 OS=Homo sapiens OX=9606 GN=PPP1R7 PE=1 SV=1	-0,026977714	0,208606
Q8IXT5	RNA-binding protein 12B OS=Homo sapiens OX=9606 GN=RBM12B PE=1 SV=2	-0,098675777	0,208299
O95999	B-cell lymphoma/leukemia 10 OS=Homo sapiens OX=9606 GN=BCL10 PE=1 SV=1	0,050475601	0,208202
Q9H0A0	RNA cytidine acetyltransferase OS=Homo sapiens OX=9606 GN=NAT10 PE=1 SV=2	0,034450523	0,208185
Q92922	SWI/SNF complex subunit SMARCC1 OS=Homo sapiens OX=9606 GN=SMARCC1 PE=1 SV=3	-0,058960923	0,208167
Q92503	SEC14-like protein 1 OS=Homo sapiens OX=9606 GN=SEC14L1 PE=1 SV=2	0,091781518	0,207365
Q8NE35	Cytoplasmic polyadenylation element-binding protein 3 OS=Homo sapiens OX=9606 GN=CPEB3 PE=1 SV=2	0,067923235	0,207107
Q96F24	Nuclear receptor-binding factor 2 OS=Homo sapiens OX=9606 GN=NRBF2 PE=1 SV=1	-0,094238056	0,206907
P48426	Phosphatidylinositol 5-phosphate 4-kinase type-2 alpha OS=Homo sapiens OX=9606 GN=PIP4K2A PE=1 SV=2	-0,041473089	0,206638
Q6UW02	Cytochrome P450 20A1 OS=Homo sapiens OX=9606 GN=CYP20A1 PE=1 SV=1	-0,050079505	0,206468
Q9NX14	NADH dehydrogenase [ubiquinone] 1 beta subcomplex subunit 11, mitochondrial OS=Homo sapiens OX=9606 GN=NDUFB11 PE=1 SV=1	0,094040857	0,206455
Q14152	Eukaryotic translation initiation factor 3 subunit A OS=Homo sapiens OX=9606 GN=EIF3A PE=1 SV=1	0,014295869	0,205399
P35240	Merlin OS=Homo sapiens OX=9606 GN=NF2 PE=1 SV=1	0,09357245	0,205381
Q9UPT8	Zinc finger CCCH domain-containing protein 4 OS=Homo sapiens OX=9606 GN=ZC3H4 PE=1 SV=3	-0,093324904	0,204814
Q9H0C8	Integrin-linked kinase-associated serine/threonine phosphatase 2C OS=Homo sapiens OX=9606 GN=ILKAP PE=1 SV=1	-0,068080955	0,204397
Q15904	V-type proton ATPase subunit S1 OS=Homo sapiens OX=9606 GN=ATP6AP1 PE=1 SV=2	-0,068054972	0,204315
P18583	Protein SON OS=Homo sapiens OX=9606 GN=SON PE=1 SV=4	-0,049570367	0,204243
P13984	General transcription factor IIF subunit 2 OS=Homo sapiens OX=9606 GN=GTF2F2 PE=1 SV=2	0,092662247	0,203297
P62333	26S proteasome regulatory subunit 10B OS=Homo sapiens OX=9606 GN=PSMC6 PE=1 SV=1	-0,024758308	0,203143
P55268	Laminin subunit beta-2 OS=Homo sapiens OX=9606 GN=LAMB2 PE=1 SV=2	0,024722823	0,202833
Q96BN8	Ubiquitin thioesterase otulin OS=Homo sapiens OX=9606 GN=OTULIN PE=1 SV=3	-0,067225754	0,201689
Q12802	A-kinase anchor protein 13 OS=Homo sapiens OX=9606 GN=AKAP13 PE=1 SV=2	-0,091835038	0,201403
Q92692	Nectin-2 OS=Homo sapiens OX=9606 GN=NECTIN2 PE=1 SV=1	0,055791629	0,201213
O43314	Inositol hexakisphosphate and diphosphoinositol-pentakisphosphate kinase 2 OS=Homo sapiens OX=9606 GN=PPIP5K2 PE=1 SV=3	-0,048837702	0,201045
P35268	60S ribosomal protein L22 OS=Homo sapiens OX=9606 GN=RPL22 PE=1 SV=2	0,04400977	0,200976
O75964	ATP synthase subunit g, mitochondrial OS=Homo sapiens OX=9606 GN=ATP5MG PE=1 SV=3	0,066913433	0,2007
Q9BTE1	Dynactin subunit 5 OS=Homo sapiens OX=9606 GN=DCTN5 PE=1 SV=1	0,055607081	0,200509
P52895	Aldo-keto reductase family 1 member C2 OS=Homo sapiens OX=9606 GN=AKR1C2 PE=1 SV=3	-0,035130984	0,200228
Q9Y285	Phenylalanine--tRNA ligase alpha subunit OS=Homo sapiens OX=9606 GN=FARSA PE=1 SV=3	-0,025952143	0,200183
Q969G3	SWI/SNF-related matrix-associated actin-dependent regulator of chromatin subfamily E member 1 OS=Homo sapiens OX=9606 GN=SMA	0,048633096	0,200152
Q4VC31	Coiled-coil domain-containing protein 58 OS=Homo sapiens OX=9606 GN=CCDC58 PE=1 SV=1	-0,091169203	0,19988
Q8ND24	RING finger protein 214 OS=Homo sapiens OX=9606 GN=RNF214 PE=1 SV=2	0,055139877	0,198727
Q5JTH9	RRP12-like protein OS=Homo sapiens OX=9606 GN=RRP12 PE=1 SV=2	0,034820208	0,198349
O96007	Molybdopterin synthase catalytic subunit OS=Homo sapiens OX=9606 GN=MOCS2 PE=1 SV=1	0,090445157	0,198224
P13674	Prolyl 4-hydroxylase subunit alpha-1 OS=Homo sapiens OX=9606 GN=P4HA1 PE=1 SV=2	-0,022336421	0,198112
P51153	Ras-related protein Rab-13 OS=Homo sapiens OX=9606 GN=RAB13 PE=1 SV=1	-0,09009239	0,197418
Q9Y572	Receptor-interacting serine/threonine-protein kinase 3 OS=Homo sapiens OX=9606 GN=RIPK3 PE=1 SV=2	-0,090032776	0,197281
Q9H8Y8	Golgi reassembly-stacking protein 2 OS=Homo sapiens OX=9606 GN=GORASP2 PE=1 SV=3	0,032733545	0,197181
Q9BVK6	Transmembrane emp24 domain-containing protein 9 OS=Homo sapiens OX=9606 GN=TMED9 PE=1 SV=2	-0,047872177	0,196838
P51148	Ras-related protein Rab-5C OS=Homo sapiens OX=9606 GN=RAB5C PE=1 SV=2	0,032585101	0,196233
P60660	Myosin light polypeptide 6 OS=Homo sapiens OX=9606 GN=MYL6 PE=1 SV=2	-0,027129186	0,194933
O43924	Retinal rod rhodopsin-sensitive cGMP 3',5'-cyclic phosphodiesterase subunit delta OS=Homo sapiens OX=9606 GN=PDE6D PE=1 SV=1	-0,088772368	0,194402
Q15007	Pre-mRNA-splicing regulator WTAP OS=Homo sapiens OX=9606 GN=WTAP PE=1 SV=2	-0,0647943	0,194006

Q6UWE0	E3 ubiquitin-protein ligase LRSAM1 OS=Homo sapiens OX=9606 GN=LRSAM1 PE=1 SV=1	0,088552439	0,1939
Q9BTE7	DCN1-like protein 5 OS=Homo sapiens OX=9606 GN=DCUN1D5 PE=1 SV=1	-0,09324418	0,193508
Q13362	Serine/threonine-protein phosphatase 2A 56 kDa regulatory subunit gamma isoform OS=Homo sapiens OX=9606 GN=PPP2R5C PE=1 SV=	0,053711664	0,193291
Q86SX6	Glutaredoxin-related protein 5, mitochondrial OS=Homo sapiens OX=9606 GN=GLRX5 PE=1 SV=2	-0,088205408	0,193108
Q99567	Nuclear pore complex protein Nup88 OS=Homo sapiens OX=9606 GN=NUP88 PE=1 SV=2	0,053652194	0,193065
Q8NFH4	Nucleoporin Nup37 OS=Homo sapiens OX=9606 GN=NUP37 PE=1 SV=1	-0,064471754	0,192989
Q9HAV7	GrpE protein homolog 1, mitochondrial OS=Homo sapiens OX=9606 GN=GRPEL1 PE=1 SV=2	-0,038884395	0,192985
Q92504	Zinc transporter SLC39A7 OS=Homo sapiens OX=9606 GN=SLC39A7 PE=1 SV=2	0,064205524	0,19215
P53985	Monocarboxylate transporter 1 OS=Homo sapiens OX=9606 GN=SLC16A1 PE=1 SV=3	-0,053274825	0,191632
Q9NWB6	Arginine and glutamate-rich protein 1 OS=Homo sapiens OX=9606 GN=ARGLU1 PE=1 SV=1	0,030536003	0,191519
Q01844	RNA-binding protein EWS OS=Homo sapiens OX=9606 GN=EWSR1 PE=1 SV=1	0,063836056	0,190986
P62829	60S ribosomal protein L23 OS=Homo sapiens OX=9606 GN=RPL23 PE=1 SV=1	0,038422554	0,190559
P42025	Beta-centractin OS=Homo sapiens OX=9606 GN=ACTR1B PE=1 SV=1	-0,063133394	0,188774
P68871	Hemoglobin subunit beta OS=Homo sapiens OX=9606 GN=HBB PE=1 SV=2	-0,086027723	0,188141
Q96RE7	Nucleus accumbens-associated protein 1 OS=Homo sapiens OX=9606 GN=NACC1 PE=1 SV=1	0,06291031	0,188072
P14635	G2/mitotic-specific cyclin-B1 OS=Homo sapiens OX=9606 GN=CCNB1 PE=1 SV=1	0,085986399	0,188047
P21359	Neurofibromin OS=Homo sapiens OX=9606 GN=NF1 PE=1 SV=2	-0,085940905	0,187943
Q9Y3E5	Peptidyl-tRNA hydrolase 2, mitochondrial OS=Homo sapiens OX=9606 GN=PTRH2 PE=1 SV=1	-0,052290619	0,187897
Q2M2I8	AP2-associated protein kinase 1 OS=Homo sapiens OX=9606 GN=AAK1 PE=1 SV=3	0,033062547	0,187758
Q03113	#N/D	-0,031353606	0,187744
Q6P179	Endoplasmic reticulum aminopeptidase 2 OS=Homo sapiens OX=9606 GN=ERAP2 PE=1 SV=2	-0,035149411	0,187316
P31949	Protein S100-A11 OS=Homo sapiens OX=9606 GN=S100A11 PE=1 SV=2	-0,035149013	0,187314
P51688	N-sulphoglucosamine sulphohydrolase OS=Homo sapiens OX=9606 GN=SGSH PE=1 SV=1	0,16402561	0,187199
Q9H3N1	Thioredoxin-related transmembrane protein 1 OS=Homo sapiens OX=9606 GN=TMX1 PE=1 SV=1	0,041094098	0,18691
P54852	Epithelial membrane protein 3 OS=Homo sapiens OX=9606 GN=EMP3 PE=1 SV=1	0,062386688	0,186426
P22033	Methylmalonyl-CoA mutase, mitochondrial OS=Homo sapiens OX=9606 GN=MMUT PE=1 SV=4	-0,04526109	0,185507
Q13795	ADP-ribosylation factor-related protein 1 OS=Homo sapiens OX=9606 GN=ARFRP1 PE=1 SV=1	-0,061851705	0,184745
P08559	Pyruvate dehydrogenase E1 component subunit alpha, somatic form, mitochondrial OS=Homo sapiens OX=9606 GN=PDHA1 PE=1 SV=3	-0,025702667	0,184094
Q9Y276	Mitochondrial chaperone BCS1 OS=Homo sapiens OX=9606 GN=BCS1L PE=1 SV=1	0,084188258	0,183952
Q9Y237	Peptidyl-prolyl cis-trans isomerase NIMA-interacting 4 OS=Homo sapiens OX=9606 GN=PIN4 PE=1 SV=1	0,083896866	0,183288
P61326	Protein mago nashi homolog OS=Homo sapiens OX=9606 GN=MAGOH PE=1 SV=1	-0,040184144	0,182542
O95159	Zinc finger protein-like 1 OS=Homo sapiens OX=9606 GN=ZFPL1 PE=1 SV=2	0,044489242	0,182171
Q96CX2	BTB/POZ domain-containing protein KCTD12 OS=Homo sapiens OX=9606 GN=KCTD12 PE=1 SV=1	0,021701425	0,181952
Q2TBA0	Kelch-like protein 40 OS=Homo sapiens OX=9606 GN=KLHL40 PE=1 SV=2	0,074583352	0,181861
P98175	RNA-binding protein 10 OS=Homo sapiens OX=9606 GN=RBM10 PE=1 SV=3	-0,050203107	0,18
Q9Y5J7	Mitochondrial import inner membrane translocase subunit Tim9 OS=Homo sapiens OX=9606 GN=TIMM9 PE=1 SV=1	0,0603349	0,179987
Q9UJ14	Glutathione hydrolase 7 OS=Homo sapiens OX=9606 GN=GGT7 PE=1 SV=2	-0,08223502	0,179509
Q9NZD2	Glycolipid transfer protein OS=Homo sapiens OX=9606 GN=GLTP PE=1 SV=3	-0,043602932	0,178347
Q8WYP5	Protein ELYS OS=Homo sapiens OX=9606 GN=AHCTF1 PE=1 SV=3	0,02540819	0,17804
P51668	Ubiquitin-conjugating enzyme E2 D1 OS=Homo sapiens OX=9606 GN=UBE2D1 PE=1 SV=1	0,059650604	0,177844
Q7Z4Q2	HEAT repeat-containing protein 3 OS=Homo sapiens OX=9606 GN=HEATR3 PE=1 SV=2	-0,059613819	0,177728
Q96AX1	Vacuolar protein sorting-associated protein 33A OS=Homo sapiens OX=9606 GN=VPS33A PE=1 SV=1	0,043410897	0,177519
Q9NT62	Ubiquitin-like-conjugating enzyme ATG3 OS=Homo sapiens OX=9606 GN=ATG3 PE=1 SV=1	-0,038962616	0,176694
Q05707	Collagen alpha-1(XIV) chain OS=Homo sapiens OX=9606 GN=COL14A1 PE=1 SV=3	0,035743904	0,176556
Q9HCD5	Nuclear receptor coactivator 5 OS=Homo sapiens OX=9606 GN=NCOA5 PE=1 SV=2	0,080830445	0,176319
Q9NZL4	Hsp70-binding protein 1 OS=Homo sapiens OX=9606 GN=HSPBP1 PE=1 SV=2	0,038789234	0,175865
Q9UNL2	Translocon-associated protein subunit gamma OS=Homo sapiens OX=9606 GN=SSR3 PE=1 SV=1	0,080599264	0,175795
P21926	CD9 antigen OS=Homo sapiens OX=9606 GN=CD9 PE=1 SV=4	0,048763065	0,174571
Q04941	Proteolipid protein 2 OS=Homo sapiens OX=9606 GN=PLP2 PE=1 SV=1	-0,080022157	0,174485
P08758	Annexin A5 OS=Homo sapiens OX=9606 GN=ANXA5 PE=1 SV=2	0,017326407	0,173492
Q9NR19	Acetyl-coenzyme A synthetase, cytoplasmic OS=Homo sapiens OX=9606 GN=ACSS2 PE=1 SV=1	-0,079446108	0,173178
Q8TD26	Chromodomain-helicase-DNA-binding protein 6 OS=Homo sapiens OX=9606 GN=CHD6 PE=1 SV=4	0,079255113	0,172745
Q9H061	Transmembrane protein 126A OS=Homo sapiens OX=9606 GN=TMEM126A PE=1 SV=1	-0,079032147	0,17224
Q9NP77	RNA polymerase II subunit A C-terminal domain phosphatase SSU72 OS=Homo sapiens OX=9606 GN=SSU72 PE=1 SV=1	0,057788189	0,172022
P61353	60S ribosomal protein L27 OS=Homo sapiens OX=9606 GN=RPL27 PE=1 SV=2	0,034775285	0,171518
Q06210	Glutamine--fructose-6-phosphate aminotransferase [isomerizing] 1 OS=Homo sapiens OX=9606 GN=GFPT1 PE=1 SV=3	0,014021274	0,171334
Q96AM1	Mas-related G-protein coupled receptor member F OS=Homo sapiens OX=9606 GN=MRGPRF PE=2 SV=1	-0,078630866	0,17133
Q5VZE5	N-alpha-acetyltransferase 35, NatC auxiliary subunit OS=Homo sapiens OX=9606 GN=NAA35 PE=1 SV=1	-0,078611217	0,171286
P31040	Succinate dehydrogenase [ubiquinone] flavoprotein subunit, mitochondrial OS=Homo sapiens OX=9606 GN=SDHA PE=1 SV=2	-0,023145088	0,171176
P63165	Small ubiquitin-related modifier 1 OS=Homo sapiens OX=9606 GN=SUMO1 PE=1 SV=1	0,047493028	0,169795
O75794	Cell division cycle protein 123 homolog OS=Homo sapiens OX=9606 GN=CDC123 PE=1 SV=1	0,13873199	0,169012
Q96EU7	C1GALT1-specific chaperone 1 OS=Homo sapiens OX=9606 GN=C1GALT1C1 PE=1 SV=1	0,077239079	0,168178
Q9ULC3	Ras-related protein Rab-23 OS=Homo sapiens OX=9606 GN=RAB23 PE=1 SV=1	-0,037164158	0,168118
O75312	Zinc finger protein ZPR1 OS=Homo sapiens OX=9606 GN=ZPR1 PE=1 SV=1	-0,031632319	0,167601
Q9UJF2	Ras GTPase-activating protein nGAP OS=Homo sapiens OX=9606 GN=RASAL2 PE=1 SV=2	0,04677926	0,167117
P39019	40S ribosomal protein S19 OS=Homo sapiens OX=9606 GN=RPS19 PE=1 SV=2	-0,029526097	0,166635
O00148	ATP-dependent RNA helicase DDX39A OS=Homo sapiens OX=9606 GN=DDX39A PE=1 SV=2	-0,021693318	0,165625
P30085	UMP-CMP kinase OS=Homo sapiens OX=9606 GN=CMPK1 PE=1 SV=3	-0,025165624	0,165359
Q9Y3D8	Adenylate kinase isoenzyme 6 OS=Homo sapiens OX=9606 GN=AK6 PE=1 SV=1	-0,075797011	0,164916
O14656	Torsin-1A OS=Homo sapiens OX=9606 GN=TOR1A PE=1 SV=1	-0,03348336	0,164821
Q9NX58	Cell growth-regulating nucleolar protein OS=Homo sapiens OX=9606 GN=LYAR PE=1 SV=2	0,055239707	0,164082
Q9H3K6	BolA-like protein 2 OS=Homo sapiens OX=9606 GN=BOLA2 PE=1 SV=1	0,040215705	0,163804
P48681	Nestin OS=Homo sapiens OX=9606 GN=NES PE=1 SV=2	0,03325034	0,163615
Q9Y5P6	Mannose-1-phosphate guanyltransferase beta OS=Homo sapiens OX=9606 GN=GMPPB PE=1 SV=2	0,033246146	0,163594
Q9UHD9	Ubiquilin-2 OS=Homo sapiens OX=9606 GN=UBQLN2 PE=1 SV=2	0,045737248	0,163213
Q9BSH4	Translational activator of cytochrome c oxidase 1 OS=Homo sapiens OX=9606 GN=TACO1 PE=1 SV=1	-0,045641216	0,162853
Q96C86	m7GpppX diphosphatase OS=Homo sapiens OX=9606 GN=DCPS PE=1 SV=2	0,045605119	0,162718
P56537	Eukaryotic translation initiation factor 6 OS=Homo sapiens OX=9606 GN=EIF6 PE=1 SV=1	-0,027252408	0,162494
Q96G23	Ceramide synthase 2 OS=Homo sapiens OX=9606 GN=CERS2 PE=1 SV=1	0,054708267	0,16243
Q5T1M5	FK506-binding protein 15 OS=Homo sapiens OX=9606 GN=FKBP15 PE=1 SV=2	-0,024678553	0,161995
P31321	cAMP-dependent protein kinase type I-beta regulatory subunit OS=Homo sapiens OX=9606 GN=PRKAR1B PE=1 SV=4	-0,263128397	0,161769
Q969T4	Ubiquitin-conjugating enzyme E2 E3 OS=Homo sapiens OX=9606 GN=UBE2E3 PE=1 SV=1	-0,074386561	0,161729
Q9GZS3	WD repeat-containing protein 61 OS=Homo sapiens OX=9606 GN=WDR61 PE=1 SV=1	0,030486709	0,161226
P12235	ADP/ATP translocase 1 OS=Homo sapiens OX=9606 GN=SLC25A4 PE=1 SV=4	0,044809188	0,159743
Q07020	60S ribosomal protein L18 OS=Homo sapiens OX=9606 GN=RPL18 PE=1 SV=2	0,035310495	0,159321
Q9ULG6	Cell cycle progression protein 1 OS=Homo sapiens OX=9606 GN=CCPG1 PE=1 SV=3	0,044657476	0,159176
Q99447	Ethanolamine-phosphate cytidylyltransferase OS=Homo sapiens OX=9606 GN=PCYT2 PE=1 SV=1	-0,030093826	0,159045
Q9P1F3	Costars family protein ABRACL OS=Homo sapiens OX=9606 GN=ABRACL PE=1 SV=1	0,073094799	0,158813
Q6ZXV5	Protein O-mannosyl-transferase TMTC3 OS=Homo sapiens OX=9606 GN=TMTC3 PE=1 SV=2	-0,030049332	0,158798
O60341	Lysine-specific histone demethylase 1A OS=Homo sapiens OX=9606 GN=KDM1A PE=1 SV=2	0,053077168	0,157369
P29353	SHC-transforming protein 1 OS=Homo sapiens OX=9606 GN=SHC1 PE=1 SV=4	0,044172402	0,157365
Q9H0W9	Ester hydrolase C11orf54 OS=Homo sapiens OX=9606 GN=C11orf54 PE=1 SV=1	-0,072122237	0,15662
P55210	Caspase-7 OS=Homo sapiens OX=9606 GN=CASP7 PE=1 SV=1	-0,038416094	0,156125
Q9BQK8	Phosphatidate phosphatase LPIN3 OS=Homo sapiens OX=9606 GN=LPIN3 PE=1 SV=3	0,071859027	0,156026
O60547	GDP-mannose 4,6 dehydratase OS=Homo sapiens OX=9606 GN=GMDS PE=1 SV=1	0,034604326	0,155981
Q6NYC8	Phostensin OS=Homo sapiens OX=9606 GN=PPP1R18 PE=1 SV=1	0,018257513	0,155946
Q15773	Myeloid leukemia factor 2 OS=Homo sapiens OX=9606 GN=MLF2 PE=1 SV=1	-0,07749807	0,155724
P62750	60S ribosomal protein L23a OS=Homo sapiens OX=9606 GN=RPL23A PE=1 SV=1	0,02948077	0,155647

O43813	Glutathione S-transferase LANCL1 OS=Homo sapiens OX=9606 GN=LANCL1 PE=1 SV=1	0,031702485	0,15563
Q9Y3E0	Vesicle transport protein GOT1B OS=Homo sapiens OX=9606 GN=GOLT1B PE=1 SV=1	0,052391915	0,155246
O00625	Pirin OS=Homo sapiens OX=9606 GN=PIR PE=1 SV=1	0,071193859	0,154528
Q10471	Polypeptide N-acetylgalactosaminyltransferase 2 OS=Homo sapiens OX=9606 GN=GALNT2 PE=1 SV=1	0,017628152	0,154316
Q92572	AP-3 complex subunit sigma-1 OS=Homo sapiens OX=9606 GN=AP3S1 PE=1 SV=1	-0,037956598	0,154169
Q9H9Q2	COP9 signalosome complex subunit 7b OS=Homo sapiens OX=9606 GN=COPS7B PE=1 SV=1	-0,052030986	0,154129
Q9NZ32	Actin-related protein 10 OS=Homo sapiens OX=9606 GN=ACTR10 PE=1 SV=1	0,024528796	0,153405
P63172	Dynein light chain Tctex-type 1 OS=Homo sapiens OX=9606 GN=DYNLT1 PE=1 SV=1	0,037733154	0,153219
Q00587	Cdc42 effector protein 1 OS=Homo sapiens OX=9606 GN=CDC42EP1 PE=1 SV=1	0,037656631	0,152893
Q8NDI1	EH domain-binding protein 1 OS=Homo sapiens OX=9606 GN=EHBP1 PE=1 SV=3	0,02570095	0,152797
Q9NW68	BSD domain-containing protein 1 OS=Homo sapiens OX=9606 GN=BSDC1 PE=1 SV=1	0,070377925	0,152691
P84090	Enhancer of rudimentary homolog OS=Homo sapiens OX=9606 GN=ERH PE=1 SV=1	-0,051564788	0,152687
O95169	NADH dehydrogenase [ubiquinone] 1 beta subcomplex subunit 8, mitochondrial OS=Homo sapiens OX=9606 GN=NDUFB8 PE=1 SV=1	-0,020539573	0,15236
Q92925	SWI/SNF-related matrix-associated actin-dependent regulator of chromatin subfamily D member 2 OS=Homo sapiens OX=9606 GN=SMA	-0,051377132	0,152107
Q9UFG5	UPF0449 protein C19orf25 OS=Homo sapiens OX=9606 GN=C19orf25 PE=1 SV=2	-0,094898309	0,152098
P55795	Heterogeneous nuclear ribonucleoprotein H2 OS=Homo sapiens OX=9606 GN=HNRNPH2 PE=1 SV=1	0,033730967	0,151858
Q9P2X0	Dolichol-phosphate mannosyltransferase subunit 3 OS=Homo sapiens OX=9606 GN=DPM3 PE=1 SV=2	-0,074109131	0,15179
Q86U42	Polyadenylate-binding protein 2 OS=Homo sapiens OX=9606 GN=PABPN1 PE=1 SV=3	0,033645401	0,151455
Q2M1P5	Kinesin-like protein KIF7 OS=Homo sapiens OX=9606 GN=KIF7 PE=1 SV=2	-0,069728978	0,15123
Q96L92	Sorting nexin-27 OS=Homo sapiens OX=9606 GN=SNX27 PE=1 SV=2	-0,069556757	0,150843
Q96JN8	Neuralized-like protein 4 OS=Homo sapiens OX=9606 GN=NEURL4 PE=1 SV=2	0,069548158	0,150824
P55196	Afadin OS=Homo sapiens OX=9606 GN=AFDN PE=1 SV=3	0,050908795	0,15066
P09038	Fibroblast growth factor 2 OS=Homo sapiens OX=9606 GN=FGF2 PE=1 SV=3	-0,042342415	0,15055
P22090	40S ribosomal protein S4, Y isoform 1 OS=Homo sapiens OX=9606 GN=RPS4Y1 PE=1 SV=2	-0,022931878	0,149985
Q16363	Laminin subunit alpha-4 OS=Homo sapiens OX=9606 GN=LAMA4 PE=1 SV=4	0,069154385	0,149938
Q15382	GTP-binding protein Rheb OS=Homo sapiens OX=9606 GN=RHEB PE=1 SV=1	-0,030501871	0,14946
P00491	Purine nucleoside phosphorylase OS=Homo sapiens OX=9606 GN=PNP PE=1 SV=2	-0,017998444	0,149433
Q14696	LRP chaperone MESD OS=Homo sapiens OX=9606 GN=MESD PE=1 SV=2	-0,028094573	0,147988
P02545	Prelamin-A/C OS=Homo sapiens OX=9606 GN=LMNA PE=1 SV=1	0,010087689	0,147671
Q9Y5Q0	Fatty acid desaturase 3 OS=Homo sapiens OX=9606 GN=FADS3 PE=1 SV=1	0,036243472	0,146896
P10155	60 kDa SS-A/Ro ribonucleoprotein OS=Homo sapiens OX=9606 GN=RO60 PE=1 SV=2	0,021517361	0,14661
Q7L2E3	ATP-dependent RNA helicase DHX30 OS=Homo sapiens OX=9606 GN=DHX30 PE=1 SV=1	0,029926877	0,146513
Q969N2	GPI transamidase component PIG-T OS=Homo sapiens OX=9606 GN=PIGT PE=1 SV=1	0,032457608	0,145865
O60504	Vinexin OS=Homo sapiens OX=9606 GN=SORBS3 PE=1 SV=2	-0,032456648	0,145861
Q9HAV4	Exportin-5 OS=Homo sapiens OX=9606 GN=XPO5 PE=1 SV=1	0,03236768	0,145443
Q9UBI1	COMM domain-containing protein 3 OS=Homo sapiens OX=9606 GN=COMMD3 PE=1 SV=1	-0,040836017	0,144959
Q6UXN9	WD repeat-containing protein 82 OS=Homo sapiens OX=9606 GN=WDR82 PE=1 SV=1	0,048759896	0,144033
P04350	#N/D	-0,031966291	0,143558
Q9Y3L5	Ras-related protein Rap-2c OS=Homo sapiens OX=9606 GN=RAP2C PE=1 SV=1	-0,111573028	0,143163
Q8N138	ORM1-like protein 3 OS=Homo sapiens OX=9606 GN=ORMDL3 PE=1 SV=1	-0,066070658	0,143014
O14602	Eukaryotic translation initiation factor 1A, Y-chromosomal OS=Homo sapiens OX=9606 GN=EIF1AY PE=1 SV=4	-0,061760401	0,142652
Q9BVL2	Nucleoporin p58/p45 OS=Homo sapiens OX=9606 GN=NUP58 PE=1 SV=1	-0,065768393	0,142336
Q5PRF9	Protein Smaug homolog 2 OS=Homo sapiens OX=9606 GN=SAMD4B PE=1 SV=1	-0,088131874	0,142186
Q99471	Prefoldin subunit 5 OS=Homo sapiens OX=9606 GN=PFDN5 PE=1 SV=2	0,025299035	0,141714
O60333	Kinesin-like protein KIF1B OS=Homo sapiens OX=9606 GN=KIF1B PE=1 SV=5	0,047521692	0,140225
Q9H7D7	WD repeat-containing protein 26 OS=Homo sapiens OX=9606 GN=WDR26 PE=1 SV=3	0,047402793	0,13986
Q9Y2A7	Nck-associated protein 1 OS=Homo sapiens OX=9606 GN=NCKAP1 PE=1 SV=1	-0,015662813	0,1398
Q86UE4	Protein LYRIC OS=Homo sapiens OX=9606 GN=MTDH PE=1 SV=2	0,018443955	0,139712
Q9BZE1	39S ribosomal protein L37, mitochondrial OS=Homo sapiens OX=9606 GN=MRPL37 PE=1 SV=2	-0,031121976	0,139601
Q6KCM7	Calcium-binding mitochondrial carrier protein SCaMC-2 OS=Homo sapiens OX=9606 GN=SLC25A25 PE=1 SV=1	-0,203943299	0,139572
Q86U86	Protein polybromo-1 OS=Homo sapiens OX=9606 GN=PBRM1 PE=1 SV=1	0,075501812	0,139418
Q9UDT6	CAP-Gly domain-containing linker protein 2 OS=Homo sapiens OX=9606 GN=CLIP2 PE=1 SV=1	0,028439896	0,138915
Q9BQ39	ATP-dependent RNA helicase DDX50 OS=Homo sapiens OX=9606 GN=DDX50 PE=1 SV=1	0,034318408	0,138758
P52292	Importin subunit alpha-1 OS=Homo sapiens OX=9606 GN=KPNA2 PE=1 SV=1	0,019624222	0,138646
P23497	Nuclear autoantigen Sp-100 OS=Homo sapiens OX=9606 GN=SP100 PE=1 SV=3	-0,023404756	0,138546
Q8N6M0	Deubiquitinase OTUD6B OS=Homo sapiens OX=9606 GN=OTUD6B PE=1 SV=1	-0,119703355	0,138523
P24390	ER lumen protein-retaining receptor 1 OS=Homo sapiens OX=9606 GN=KDELR1 PE=1 SV=1	-0,046758671	0,137883
Q676U5	Autophagy-related protein 16-1 OS=Homo sapiens OX=9606 GN=ATG16L1 PE=1 SV=2	0,063740399	0,137794
P28039	Acyloxyacyl hydrolase OS=Homo sapiens OX=9606 GN=AOAH PE=1 SV=1	-0,063728424	0,137768
P82933	28S ribosomal protein S9, mitochondrial OS=Homo sapiens OX=9606 GN=MRPS9 PE=1 SV=2	0,03877683	0,137343
O75934	Pre-mRNA-splicing factor SPF27 OS=Homo sapiens OX=9606 GN=BCAS2 PE=1 SV=1	-0,046570532	0,137305
Q8IWT6	Volume-regulated anion channel subunit LRRC8A OS=Homo sapiens OX=9606 GN=LRRC8A PE=1 SV=1	-0,05619892	0,137111
Q9UI30	Multifunctional methyltransferase subunit TRM112-like protein OS=Homo sapiens OX=9606 GN=TRMT112 PE=1 SV=1	-0,046487368	0,13705
P17661	Desmin OS=Homo sapiens OX=9606 GN=DES PE=1 SV=3	0,03868884	0,137018
Q9Y3C8	Ubiquitin-fold modifier-conjugating enzyme 1 OS=Homo sapiens OX=9606 GN=UFC1 PE=1 SV=3	-0,046379243	0,136719
Q14CX7	N-alpha-acetyltransferase 25, NatB auxiliary subunit OS=Homo sapiens OX=9606 GN=NAA25 PE=1 SV=1	-0,04635215	0,136635
P62942	Peptidyl-prolyl cis-trans isomerase FKBP1A OS=Homo sapiens OX=9606 GN=FKBP1A PE=1 SV=2	0,030393317	0,136192
Q93052	Lipoma-preferred partner OS=Homo sapiens OX=9606 GN=LPP PE=1 SV=1	0,01857747	0,135933
Q8IYM9	E3 ubiquitin-protein ligase TRIM22 OS=Homo sapiens OX=9606 GN=TRIM22 PE=1 SV=1	0,062859566	0,135824
P61106	Ras-related protein Rab-14 OS=Homo sapiens OX=9606 GN=RAB14 PE=1 SV=4	-0,019232463	0,135757
P0C7V7	#N/D	-0,101598438	0,134863
Q8TE73	Dynein heavy chain 5, axonemal OS=Homo sapiens OX=9606 GN=DNAH5 PE=1 SV=3	0,062372454	0,134736
Q8NFW8	N-acylneuraminate cytidylyltransferase OS=Homo sapiens OX=9606 GN=CMAS PE=1 SV=2	0,062227327	0,134411
Q66K14	TBC1 domain family member 9B OS=Homo sapiens OX=9606 GN=TBC1D9B PE=1 SV=3	-0,062133488	0,134202
P16104	Histone H2AX OS=Homo sapiens OX=9606 GN=H2AFX PE=1 SV=2	-0,062059055	0,134035
Q13423	NAD(P) transhydrogenase, mitochondrial OS=Homo sapiens OX=9606 GN=NNT PE=1 SV=3	-0,015016244	0,13378
Q86UU1	Pleckstrin homology-like domain family B member 1 OS=Homo sapiens OX=9606 GN=PHLDB1 PE=1 SV=1	-0,022478593	0,132831
O43663	Protein regulator of cytokinesis 1 OS=Homo sapiens OX=9606 GN=PRC1 PE=1 SV=2	-0,044911379	0,132222
Q9NR45	Sialic acid synthase OS=Homo sapiens OX=9606 GN=NANS PE=1 SV=2	-0,021260514	0,132105
P16401	Histone H1.5 OS=Homo sapiens OX=9606 GN=HIST1H1B PE=1 SV=3	0,022314564	0,13182
Q7Z3U7	Protein MON2 homolog OS=Homo sapiens OX=9606 GN=MON2 PE=1 SV=3	0,025086278	0,131482
P01210	Proenkephalin-A OS=Homo sapiens OX=9606 GN=PENK PE=1 SV=1	-0,080218447	0,131276
O00442	RNA 3'-terminal phosphate cyclase OS=Homo sapiens OX=9606 GN=RTCA PE=1 SV=1	0,044600758	0,131272
Q93050	V-type proton ATPase 116 kDa subunit a isoform 1 OS=Homo sapiens OX=9606 GN=ATP6V0A1 PE=1 SV=3	-0,032476747	0,131008
Q5T4S7	E3 ubiquitin-protein ligase UBR4 OS=Homo sapiens OX=9606 GN=UBR4 PE=1 SV=1	-0,008280719	0,13065
P36405	ADP-ribosylation factor-like protein 3 OS=Homo sapiens OX=9606 GN=ARL3 PE=1 SV=2	-0,023402488	0,130648
Q9UJ70	N-acetyl-D-glucosamine kinase OS=Homo sapiens OX=9606 GN=NAGK PE=1 SV=4	-0,017870002	0,130538
O95372	Acyl-protein thioesterase 2 OS=Homo sapiens OX=9606 GN=LYPLA2 PE=1 SV=1	0,029167267	0,130473
Q14C86	GTPase-activating protein and VPS9 domain-containing protein 1 OS=Homo sapiens OX=9606 GN=GAPVD1 PE=1 SV=2	-0,026680067	0,129966
O15530	3-phosphoinositide-dependent protein kinase 1 OS=Homo sapiens OX=9606 GN=PDPK1 PE=1 SV=1	-0,060154117	0,129783
Q8IX12	Cell division cycle and apoptosis regulator protein 1 OS=Homo sapiens OX=9606 GN=CCAR1 PE=1 SV=2	-0,024704698	0,129399
O76061	Stanniocalcin-2 OS=Homo sapiens OX=9606 GN=STC2 PE=1 SV=1	-0,03657267	0,129226
Q92905	COP9 signalosome complex subunit 5 OS=Homo sapiens OX=9606 GN=COPS5 PE=1 SV=4	-0,019871416	0,129141
Q15392	Delta(24)-sterol reductase OS=Homo sapiens OX=9606 GN=DHCR24 PE=1 SV=2	-0,043524383	0,127983
P50395	Rab GDP dissociation inhibitor beta OS=Homo sapiens OX=9606 GN=GDI2 PE=1 SV=2	0,013423799	0,127426
Q86V81	THO complex subunit 4 OS=Homo sapiens OX=9606 GN=ALYREF PE=1 SV=3	0,024264612	0,127001
Q9BUT1	3-hydroxybutyrate dehydrogenase type 2 OS=Homo sapiens OX=9606 GN=BDH2 PE=1 SV=2	-0,031453686	0,126717
Q96S59	Ran-binding protein 9 OS=Homo sapiens OX=9606 GN=RANBP9 PE=1 SV=1	0,031436891	0,126647

Q9BVJ6	U3 small nucleolar RNA-associated protein 14 homolog A OS=Homo sapiens OX=9606 GN=UTP14A PE=1 SV=1	0,031367443	0,126356
Q9Y2S7	Polymerase delta-interacting protein 2 OS=Homo sapiens OX=9606 GN=POLDIP2 PE=1 SV=1	-0,058410896	0,125899
Q9UJZ1	Stomatin-like protein 2, mitochondrial OS=Homo sapiens OX=9606 GN=STOML2 PE=1 SV=1	0,021312519	0,125662
P11047	Laminin subunit gamma-1 OS=Homo sapiens OX=9606 GN=LAMC1 PE=1 SV=3	-0,010839309	0,125541
P98194	Calcium-transporting ATPase type 2C member 1 OS=Homo sapiens OX=9606 GN=ATP2C1 PE=1 SV=3	0,031116668	0,125306
Q12841	Follistatin-related protein 1 OS=Homo sapiens OX=9606 GN=FSTL1 PE=1 SV=1	-0,017809173	0,125305
Q9BRJ6	Uncharacterized protein C7orf50 OS=Homo sapiens OX=9606 GN=C7orf50 PE=1 SV=1	-0,035386053	0,124872
Q6NUP7	Serine/threonine-protein phosphatase 4 regulatory subunit 4 OS=Homo sapiens OX=9606 GN=PPP4R4 PE=1 SV=1	0,281385438	0,124772
Q9UKD2	mRNA turnover protein 4 homolog OS=Homo sapiens OX=9606 GN=MRTO4 PE=1 SV=2	-0,057892843	0,124746
Q9UBN7	Histone deacetylase 6 OS=Homo sapiens OX=9606 GN=HDAC6 PE=1 SV=2	0,057827079	0,124599
P24385	G1/S-specific cyclin-D1 OS=Homo sapiens OX=9606 GN=CCND1 PE=1 SV=1	-0,057673986	0,124259
Q9BZV1	UBX domain-containing protein 6 OS=Homo sapiens OX=9606 GN=UBXN6 PE=1 SV=1	0,027787939	0,124061
Q5SWX8	Protein odr-4 homolog OS=Homo sapiens OX=9606 GN=ODR4 PE=1 SV=1	-0,033644304	0,124054
Q53GQ0	Very-long-chain 3-oxoacyl-CoA reductase OS=Homo sapiens OX=9606 GN=HSD17B12 PE=1 SV=2	0,019971221	0,123775
Q13283	Ras GTPase-activating protein-binding protein 1 OS=Homo sapiens OX=9606 GN=G3BP1 PE=1 SV=1	-0,017597873	0,123759
Q05639	#N/D	0,019047419	0,123573
Q9UBG0	C-type mannose receptor 2 OS=Homo sapiens OX=9606 GN=MRC2 PE=1 SV=2	0,011833894	0,123114
Q9UNP9	Peptidyl-prolyl cis-trans isomerase E OS=Homo sapiens OX=9606 GN=PPIE PE=1 SV=1	0,101467535	0,123052
Q9Y6D5	Brefeldin A-inhibited guanine nucleotide-exchange protein 2 OS=Homo sapiens OX=9606 GN=ARFGEF2 PE=1 SV=3	-0,019857489	0,123042
P08962	CD63 antigen OS=Homo sapiens OX=9606 GN=CD63 PE=1 SV=2	-0,056940444	0,122627
O75663	TIP41-like protein OS=Homo sapiens OX=9606 GN=TIPRL PE=1 SV=2	0,021859615	0,121699
Q9BTD8	RNA-binding protein 42 OS=Homo sapiens OX=9606 GN=RBM42 PE=1 SV=1	-0,056394495	0,121414
Q96A33	Coiled-coil domain-containing protein 47 OS=Homo sapiens OX=9606 GN=CCDC47 PE=1 SV=1	-0,015605605	0,121284
Q9Y3U8	60S ribosomal protein L36 OS=Homo sapiens OX=9606 GN=RPL36 PE=1 SV=3	-0,02711952	0,120962
Q13884	Beta-1-syntrophin OS=Homo sapiens OX=9606 GN=SNTB1 PE=1 SV=3	-0,029985063	0,120576
Q9NZU1	Leucine-rich repeat transmembrane protein FLRT1 OS=Homo sapiens OX=9606 GN=FLRT1 PE=1 SV=3	-0,11877811	0,120449
Q9UGP8	Translocation protein SEC63 homolog OS=Homo sapiens OX=9606 GN=SEC63 PE=1 SV=2	0,015967834	0,120228
O15439	Multidrug resistance-associated protein 4 OS=Homo sapiens OX=9606 GN=ABCC4 PE=1 SV=3	0,040836085	0,119795
Q9BQ52	Zinc phosphodiesterase ELAC protein 2 OS=Homo sapiens OX=9606 GN=ELAC2 PE=1 SV=2	0,115671786	0,119617
P36543	V-type proton ATPase subunit E 1 OS=Homo sapiens OX=9606 GN=ATP6V1E1 PE=1 SV=1	-0,021468019	0,119435
P05091	Aldehyde dehydrogenase, mitochondrial OS=Homo sapiens OX=9606 GN=ALDH2 PE=1 SV=2	0,020293304	0,119422
Q01650	Large neutral amino acids transporter small subunit 1 OS=Homo sapiens OX=9606 GN=SLC7A5 PE=1 SV=2	0,055016483	0,118354
Q8IWV7	E3 ubiquitin-protein ligase UBR1 OS=Homo sapiens OX=9606 GN=UBR1 PE=1 SV=1	-0,040317557	0,118219
Q03169	Tumor necrosis factor alpha-induced protein 2 OS=Homo sapiens OX=9606 GN=TNFAIP2 PE=2 SV=2	0,054929817	0,118161
P47813	#N/D	0,040275109	0,118091
Q9Y2W1	Thyroid hormone receptor-associated protein 3 OS=Homo sapiens OX=9606 GN=THRAP3 PE=1 SV=2	-0,016813322	0,118031
Q9UDR5	Alpha-aminoadipic semialdehyde synthase, mitochondrial OS=Homo sapiens OX=9606 GN=AASS PE=1 SV=1	-0,054822004	0,117922
Q6DN90	IQ motif and SEC7 domain-containing protein 1 OS=Homo sapiens OX=9606 GN=IQSEC1 PE=1 SV=1	-0,033354687	0,117442
P48047	ATP synthase subunit O, mitochondrial OS=Homo sapiens OX=9606 GN=ATP5PO PE=1 SV=1	0,018910314	0,116951
Q9HCU0	Endosialin OS=Homo sapiens OX=9606 GN=CD248 PE=1 SV=1	-0,016060556	0,116821
O00506	Serine/threonine-protein kinase 25 OS=Homo sapiens OX=9606 GN=STK25 PE=1 SV=1	0,024040756	0,116631
Q9NZT2	Opioid growth factor receptor OS=Homo sapiens OX=9606 GN=OGFR PE=1 SV=3	-0,032987749	0,116103
P55285	Cadherin-6 OS=Homo sapiens OX=9606 GN=CDH6 PE=1 SV=1	-0,028769178	0,115508
Q6EMB2	Tubulin polyglutamylase TTLL5 OS=Homo sapiens OX=9606 GN=TTLL5 PE=1 SV=3	0,068688844	0,115318
Q9Y3B4	Splicing factor 3B subunit 6 OS=Homo sapiens OX=9606 GN=SF3B6 PE=1 SV=1	-0,039330367	0,115224
Q9Y6N5	Sulfide:quinone oxidoreductase, mitochondrial OS=Homo sapiens OX=9606 GN=SQOR PE=1 SV=1	-0,015315001	0,115129
P31150	Rab GDP dissociation inhibitor alpha OS=Homo sapiens OX=9606 GN=GDI1 PE=1 SV=2	0,011681499	0,114879
O60524	Nuclear export mediator factor NEMF OS=Homo sapiens OX=9606 GN=NEMF PE=1 SV=4	0,3155544	0,114819
Q9BYG3	MKI67 FHA domain-interacting nucleolar phosphoprotein OS=Homo sapiens OX=9606 GN=NIFK PE=1 SV=1	0,028562516	0,114649
P19784	Casein kinase II subunit alpha' OS=Homo sapiens OX=9606 GN=CSNK2A2 PE=1 SV=1	-0,025581968	0,113855
Q00537	Cyclin-dependent kinase 17 OS=Homo sapiens OX=9606 GN=CDK17 PE=1 SV=2	-0,052855687	0,113849
Q15008	26S proteasome non-ATPase regulatory subunit 6 OS=Homo sapiens OX=9606 GN=PSMD6 PE=1 SV=1	0,014239371	0,11371
Q8NFP9	Neurobeachin OS=Homo sapiens OX=9606 GN=NBEA PE=1 SV=3	-0,05257555	0,112943
P53007	Tricarboxylate transport protein, mitochondrial OS=Homo sapiens OX=9606 GN=SLC25A1 PE=1 SV=2	0,032035278	0,112633
P67775	Serine/threonine-protein phosphatase 2A catalytic subunit alpha isoform OS=Homo sapiens OX=9606 GN=PPP2CA PE=1 SV=1	0,052269519	0,112266
Q9H845	Acyl-CoA dehydrogenase family member 9, mitochondrial OS=Homo sapiens OX=9606 GN=ACAD9 PE=1 SV=1	-0,022915704	0,110978
P22695	Cytochrome b-c1 complex subunit 2, mitochondrial OS=Homo sapiens OX=9606 GN=UQCRC2 PE=1 SV=3	-0,014769314	0,110878
P30260	Cell division cycle protein 27 homolog OS=Homo sapiens OX=9606 GN=CDC27 PE=1 SV=2	-0,037816676	0,11064
P14923	#N/D	-0,03144881	0,1105
Q9H6Y2	WD repeat-containing protein 55 OS=Homo sapiens OX=9606 GN=WDR55 PE=1 SV=2	-0,03163283	0,109599
Q9H254	Spectrin beta chain, non-erythrocytic 4 OS=Homo sapiens OX=9606 GN=SPTBN4 PE=1 SV=2	-0,037405135	0,109396
Q2TAY7	WD40 repeat-containing protein SMU1 OS=Homo sapiens OX=9606 GN=SMU1 PE=1 SV=2	0,022408362	0,108435
Q6PCB7	Long-chain fatty acid transport protein 1 OS=Homo sapiens OX=9606 GN=SLC27A1 PE=1 SV=1	0,027023513	0,108259
Q15345	Leucine-rich repeat-containing protein 41 OS=Homo sapiens OX=9606 GN=LRRC41 PE=1 SV=3	-0,050311882	0,107938
Q9NVH0	Exonuclease 3'-5' domain-containing protein 2 OS=Homo sapiens OX=9606 GN=EXD2 PE=1 SV=2	0,05012643	0,107528
Q9Y3E7	Charged multivesicular body protein 3 OS=Homo sapiens OX=9606 GN=CHMP3 PE=1 SV=3	0,024200926	0,107497
Q969E8	Pre-rRNA-processing protein TSR2 homolog OS=Homo sapiens OX=9606 GN=TSR2 PE=1 SV=1	0,055803604	0,107401
Q9P299	Coatomer subunit zeta-2 OS=Homo sapiens OX=9606 GN=COPZ2 PE=2 SV=1	-0,030457063	0,106898
Q86WV6	Stimulator of interferon genes protein OS=Homo sapiens OX=9606 GN=TMEM173 PE=1 SV=1	0,030339011	0,10647
Q86TX2	Acyl-coenzyme A thioesterase 1 OS=Homo sapiens OX=9606 GN=ACOT1 PE=1 SV=1	-0,018162869	0,106453
Q9BT09	Protein canopy homolog 3 OS=Homo sapiens OX=9606 GN=CNPY3 PE=1 SV=1	-0,023785743	0,105591
Q9BVC6	Transmembrane protein 109 OS=Homo sapiens OX=9606 GN=TMEM109 PE=1 SV=1	0,036143753	0,105588
P62861	40S ribosomal protein S30 OS=Homo sapiens OX=9606 GN=FAU PE=1 SV=1	-0,049110755	0,105287
P14678	Small nuclear ribonucleoprotein-associated proteins B and B' OS=Homo sapiens OX=9606 GN=SNRPB PE=1 SV=2	-0,021741606	0,1051
P98082	Disabled homolog 2 OS=Homo sapiens OX=9606 GN=DAB2 PE=1 SV=3	0,014903491	0,104169
P80303	Nucleobindin-2 OS=Homo sapiens OX=9606 GN=NUCB2 PE=1 SV=3	0,01347076	0,104129
O60749	Sorting nexin-2 OS=Homo sapiens OX=9606 GN=SNX2 PE=1 SV=2	-0,015439402	0,10381
Q92930	Ras-related protein Rab-8B OS=Homo sapiens OX=9606 GN=RAB8B PE=1 SV=2	-0,04838909	0,103695
Q9BX95	Sphingosine-1-phosphate phosphatase 1 OS=Homo sapiens OX=9606 GN=SGPP1 PE=1 SV=2	-0,048111818	0,103084
O75695	Protein XRP2 OS=Homo sapiens OX=9606 GN=RP2 PE=1 SV=4	-0,025750168	0,10299
Q9Y5Y2	Cytosolic Fe-S cluster assembly factor NUBP2 OS=Homo sapiens OX=9606 GN=NUBP2 PE=1 SV=1	0,021136268	0,102077
P63173	60S ribosomal protein L38 OS=Homo sapiens OX=9606 GN=RPL38 PE=1 SV=2	0,047456396	0,10164
O95336	6-phosphogluconolactonase OS=Homo sapiens OX=9606 GN=PGLS PE=1 SV=2	-0,014545193	0,101582
Q9Y3C1	Nucleolar protein 16 OS=Homo sapiens OX=9606 GN=NOP16 PE=1 SV=2	-0,020939976	0,101537
P53621	Coatomer subunit alpha OS=Homo sapiens OX=9606 GN=COPA PE=1 SV=2	0,00669049	0,101445
Q07889	Son of sevenless homolog 1 OS=Homo sapiens OX=9606 GN=SOS1 PE=1 SV=1	0,095908821	0,100817
Q53H12	Acylglycerol kinase, mitochondrial OS=Homo sapiens OX=9606 GN=AGK PE=1 SV=2	0,025220848	0,100805
Q16186	Proteasomal ubiquitin receptor ADRM1 OS=Homo sapiens OX=9606 GN=ADRM1 PE=1 SV=2	-0,020799239	0,100396
P07919	Cytochrome b-c1 complex subunit 6, mitochondrial OS=Homo sapiens OX=9606 GN=UQCRH PE=1 SV=2	0,046511582	0,099561
O75487	Glypican-4 OS=Homo sapiens OX=9606 GN=GPC4 PE=1 SV=4	0,019168858	0,099475
Q9BRA2	Thioredoxin domain-containing protein 17 OS=Homo sapiens OX=9606 GN=TXNDC17 PE=1 SV=1	0,024843035	0,099247
Q15942	Zyxin OS=Homo sapiens OX=9606 GN=ZYX PE=1 SV=1	0,01247363	0,099152
Q96C01	Protein FAM136A OS=Homo sapiens OX=9606 GN=FAM136A PE=1 SV=1	-0,046294421	0,099083
Q16204	Coiled-coil domain-containing protein 6 OS=Homo sapiens OX=9606 GN=CCDC6 PE=1 SV=2	-0,017840939	0,098612
P41214	Eukaryotic translation initiation factor 2D OS=Homo sapiens OX=9606 GN=EIF2D PE=1 SV=3	-0,024342978	0,097187
Q96AT9	Ribulose-phosphate 3-epimerase OS=Homo sapiens OX=9606 GN=RPE PE=1 SV=1	0,04536622	0,097042
P30837	Aldehyde dehydrogenase X, mitochondrial OS=Homo sapiens OX=9606 GN=ALDH1B1 PE=1 SV=3	-0,016590828	0,096948
P08708	40S ribosomal protein S17 OS=Homo sapiens OX=9606 GN=RPS17 PE=1 SV=2	-0,018693993	0,096933

Q9Y2Q5	Ragulator complex protein LAMTOR2 OS=Homo sapiens OX=9606 GN=LAMTOR2 PE=1 SV=1	-0,033259231	0,09691
Q9H1Y0	Autophagy protein 5 OS=Homo sapiens OX=9606 GN=ATG5 PE=1 SV=2	0,033252827	0,096891
P23786	Carnitine O-palmitoyltransferase 2, mitochondrial OS=Homo sapiens OX=9606 GN=CPT2 PE=1 SV=2	0,045224979	0,096732
O43684	Mitotic checkpoint protein BUB3 OS=Homo sapiens OX=9606 GN=BUB3 PE=1 SV=1	0,017490479	0,096614
Q8NB37	Glutamine amidotransferase-like class 1 domain-containing protein 1 OS=Homo sapiens OX=9606 GN=GATD1 PE=1 SV=1	0,045120094	0,096502
O95361	Tripartite motif-containing protein 16 OS=Homo sapiens OX=9606 GN=TRIM16 PE=1 SV=3	0,044665288	0,095503
P57772	Selenocysteine-specific elongation factor OS=Homo sapiens OX=9606 GN=EEFSEC PE=1 SV=4	0,065911895	0,094932
P51572	B-cell receptor-associated protein 31 OS=Homo sapiens OX=9606 GN=BCAP31 PE=1 SV=3	-0,017083962	0,094299
P40926	Malate dehydrogenase, mitochondrial OS=Homo sapiens OX=9606 GN=MDH2 PE=1 SV=3	0,012612505	0,094187
Q9BRT2	Ubiquinol-cytochrome-c reductase complex assembly factor 2 OS=Homo sapiens OX=9606 GN=UQCC2 PE=1 SV=1	0,01392173	0,093859
Q92629	Delta-sarcoglycan OS=Homo sapiens OX=9606 GN=SGCD PE=1 SV=2	0,032200669	0,093736
Q969Q0	60S ribosomal protein L36a-like OS=Homo sapiens OX=9606 GN=RPL36AL PE=1 SV=3	-0,03218051	0,093676
P16333	Cytoplasmic protein NCK1 OS=Homo sapiens OX=9606 GN=NCK1 PE=1 SV=1	-0,021179281	0,09367
P11908	Ribose-phosphate pyrophosphokinase 2 OS=Homo sapiens OX=9606 GN=PRPS2 PE=1 SV=2	-0,014537474	0,093425
Q9H0E2	Toll-interacting protein OS=Homo sapiens OX=9606 GN=TOLLIP PE=1 SV=1	-0,02665893	0,093174
P52209	6-phosphogluconate dehydrogenase, decarboxylating OS=Homo sapiens OX=9606 GN=PGD PE=1 SV=3	-0,010107503	0,092947
P84085	ADP-ribosylation factor 5 OS=Homo sapiens OX=9606 GN=ARF5 PE=1 SV=2	0,020836591	0,092109
O96033	Molybdopterin synthase sulfur carrier subunit OS=Homo sapiens OX=9606 GN=MOCS2 PE=1 SV=1	0,031652758	0,092095
Q15102	Platelet-activating factor acetylhydrolase IB subunit gamma OS=Homo sapiens OX=9606 GN=PAFAH1B3 PE=1 SV=1	-0,031546197	0,091777
Q9UNN8	Endothelial protein C receptor OS=Homo sapiens OX=9606 GN=PROCR PE=1 SV=1	0,031234015	0,090843
Q9H9B4	Sideroflexin-1 OS=Homo sapiens OX=9606 GN=SFXN1 PE=1 SV=4	-0,017529269	0,090715
Q29RF7	Sister chromatid cohesion protein PDS5 homolog A OS=Homo sapiens OX=9606 GN=PDS5A PE=1 SV=1	0,02591602	0,090503
P09488	Glutathione S-transferase Mu 1 OS=Homo sapiens OX=9606 GN=GSTM1 PE=1 SV=3	0,022496389	0,089602
Q92696	Geranylgeranyl transferase type-2 subunit alpha OS=Homo sapiens OX=9606 GN=RABGGTA PE=1 SV=2	0,016231188	0,089454
P78347	General transcription factor II-I OS=Homo sapiens OX=9606 GN=GTF2I PE=1 SV=2	0,011993056	0,089425
Q9H1I8	Activating signal cointegrator 1 complex subunit 2 OS=Homo sapiens OX=9606 GN=ASCC2 PE=1 SV=3	0,025553513	0,089201
Q99720	Sigma non-opioid intracellular receptor 1 OS=Homo sapiens OX=9606 GN=SIGMAR1 PE=1 SV=1	-0,086063112	0,08917
O95819	Mitogen-activated protein kinase kinase kinase kinase 4 OS=Homo sapiens OX=9606 GN=MAP4K4 PE=1 SV=2	-0,011233728	0,089007
P12109	Collagen alpha-1(VI) chain OS=Homo sapiens OX=9606 GN=COL6A1 PE=1 SV=3	-0,007468745	0,088952
Q6PJT7	Zinc finger CCCH domain-containing protein 14 OS=Homo sapiens OX=9606 GN=ZC3H14 PE=1 SV=1	-0,030558419	0,088823
O95487	Protein transport protein Sec24B OS=Homo sapiens OX=9606 GN=SEC24B PE=1 SV=2	-0,015976119	0,088008
P48729	Casein kinase I isoform alpha OS=Homo sapiens OX=9606 GN=CSNK1A1 PE=1 SV=2	0,02199888	0,087564
L0R6Q1	SLC35A4 upstream open reading frame protein OS=Homo sapiens OX=9606 GN=SLC35A4 PE=3 SV=1	-0,041024934	0,087526
Q96S66	Chloride channel CLIC-like protein 1 OS=Homo sapiens OX=9606 GN=CLCC1 PE=1 SV=1	0,018138579	0,087189
Q7Z4I7	LIM and senescent cell antigen-like-containing domain protein 2 OS=Homo sapiens OX=9606 GN=LIMS2 PE=1 SV=1	0,021764927	0,086607
P05455	Lupus La protein OS=Homo sapiens OX=9606 GN=SSB PE=1 SV=2	0,010347187	0,086482
Q8NBJ4	Golgi membrane protein 1 OS=Homo sapiens OX=9606 GN=GOLM1 PE=1 SV=1	-0,02474068	0,086285
Q96FW1	Ubiquitin thioesterase OTUB1 OS=Homo sapiens OX=9606 GN=OTUB1 PE=1 SV=2	-0,012807988	0,08562
Q9Y223	Bifunctional UDP-N-acetylglucosamine 2-epimerase/N-acetylmannosamine kinase OS=Homo sapiens OX=9606 GN=GNE PE=1 SV=1	-0,016459878	0,085027
Q9Y6M9	NADH dehydrogenase [ubiquinone] 1 beta subcomplex subunit 9 OS=Homo sapiens OX=9606 GN=NDUFB9 PE=1 SV=3	0,074352355	0,084342
Q9HA72	Calcium homeostasis modulator protein 2 OS=Homo sapiens OX=9606 GN=CALHM2 PE=2 SV=1	-0,039449549	0,084083
P23526	Adenosylhomocysteinase OS=Homo sapiens OX=9606 GN=AHCY PE=1 SV=4	-0,012054166	0,083706
O15121	Sphingolipid delta(4)-desaturase DES1 OS=Homo sapiens OX=9606 GN=DEGS1 PE=1 SV=1	0,039209349	0,083559
Q96QC0	Serine/threonine-protein phosphatase 1 regulatory subunit 10 OS=Homo sapiens OX=9606 GN=PPP1R10 PE=1 SV=1	0,039119738	0,083363
O75592	E3 ubiquitin-protein ligase MYCBP2 OS=Homo sapiens OX=9606 GN=MYCBP2 PE=1 SV=4	-0,038609935	0,082251
P30613	Pyruvate kinase PKLR OS=Homo sapiens OX=9606 GN=PKLR PE=1 SV=2	0,025660145	0,082228
Q9ULH1	Arf-GAP with SH3 domain, ANK repeat and PH domain-containing protein 1 OS=Homo sapiens OX=9606 GN=ASAP1 PE=1 SV=4	-0,023592927	0,082177
Q15370	Elongin-B OS=Homo sapiens OX=9606 GN=ELOB PE=1 SV=1	-0,017108548	0,082105
Q00765	Receptor expression-enhancing protein 5 OS=Homo sapiens OX=9606 GN=REEP5 PE=1 SV=3	-0,017057365	0,081853
Q3KQV9	UDP-N-acetylhexosamine pyrophosphorylase-like protein 1 OS=Homo sapiens OX=9606 GN=UAP1L1 PE=1 SV=2	-0,020548749	0,081639
P48507	Glutamate--cysteine ligase regulatory subunit OS=Homo sapiens OX=9606 GN=GCLM PE=1 SV=1	0,018386966	0,080995
P29992	Guanine nucleotide-binding protein subunit alpha-11 OS=Homo sapiens OX=9606 GN=GNA11 PE=1 SV=2	0,013900703	0,080811
Q9NZB2	Constitutive coactivator of PPAR-gamma-like protein 1 OS=Homo sapiens OX=9606 GN=FAM120A PE=1 SV=2	-0,00811365	0,080395
Q8N4B5	Proline-rich protein 18 OS=Homo sapiens OX=9606 GN=PRR18 PE=2 SV=2	-0,050336529	0,079948
Q99541	Perilipin-2 OS=Homo sapiens OX=9606 GN=PLIN2 PE=1 SV=2	0,037482883	0,079794
O43747	AP-1 complex subunit gamma-1 OS=Homo sapiens OX=9606 GN=AP1G1 PE=1 SV=5	-0,009566402	0,079784
P49841	Glycogen synthase kinase-3 beta OS=Homo sapiens OX=9606 GN=GSK3B PE=1 SV=2	-0,027509467	0,079739
P47985	Cytochrome b-c1 complex subunit Rieske, mitochondrial OS=Homo sapiens OX=9606 GN=UQCRFS1 PE=1 SV=2	-0,013035405	0,079672
P00441	Superoxide dismutase [Cu-Zn] OS=Homo sapiens OX=9606 GN=SOD1 PE=1 SV=2	-0,020054281	0,079624
P62873	Guanine nucleotide-binding protein G(I)/G(S)/G(T) subunit beta-1 OS=Homo sapiens OX=9606 GN=GNB1 PE=1 SV=3	-0,014481175	0,079557
P10768	S-formylglutathione hydrolase OS=Homo sapiens OX=9606 GN=ESD PE=1 SV=2	0,011468387	0,079531
Q14166	Tubulin--tyrosine ligase-like protein 12 OS=Homo sapiens OX=9606 GN=TTLL12 PE=1 SV=2	0,011019053	0,079197
Q96MW1	Coiled-coil domain-containing protein 43 OS=Homo sapiens OX=9606 GN=CCDC43 PE=1 SV=2	0,027324483	0,079189
Q96JJ7	Protein disulfide-isomerase TMX3 OS=Homo sapiens OX=9606 GN=TMX3 PE=1 SV=2	0,011386591	0,078949
O75940	Survival of motor neuron-related-splicing factor 30 OS=Homo sapiens OX=9606 GN=SMNDC1 PE=1 SV=1	-0,027194828	0,078804
Q7Z7A4	PX domain-containing protein kinase-like protein OS=Homo sapiens OX=9606 GN=PXK PE=1 SV=1	-0,027172081	0,078737
Q96EP0	E3 ubiquitin-protein ligase RNF31 OS=Homo sapiens OX=9606 GN=RNF31 PE=1 SV=1	-0,028274568	0,078209
Q9Y262	Eukaryotic translation initiation factor 3 subunit L OS=Homo sapiens OX=9606 GN=EIF3L PE=1 SV=1	-0,007891955	0,078142
Q99996	A-kinase anchor protein 9 OS=Homo sapiens OX=9606 GN=AKAP9 PE=1 SV=4	-0,036300401	0,07722
Q9P2I0	Cleavage and polyadenylation specificity factor subunit 2 OS=Homo sapiens OX=9606 GN=CPSF2 PE=1 SV=2	-0,036251621	0,077114
Q96AY4	Tetratricopeptide repeat protein 28 OS=Homo sapiens OX=9606 GN=TTC28 PE=1 SV=4	0,15335642	0,075826
Q5VW32	BRO1 domain-containing protein BROX OS=Homo sapiens OX=9606 GN=BROX PE=1 SV=1	-0,021733735	0,075543
Q9P0V3	SH3 domain-binding protein 4 OS=Homo sapiens OX=9606 GN=SH3BP4 PE=1 SV=1	-0,009696067	0,074236
P55265	Double-stranded RNA-specific adenosine deaminase OS=Homo sapiens OX=9606 GN=ADAR PE=1 SV=4	0,01071327	0,074166
P61964	WD repeat-containing protein 5 OS=Homo sapiens OX=9606 GN=WDR5 PE=1 SV=1	-0,055989667	0,074065
Q9NYL2	Mitogen-activated protein kinase kinase kinase 20 OS=Homo sapiens OX=9606 GN=MAP3K20 PE=1 SV=3	-0,034745034	0,07384
Q8NEZ2	Vacuolar protein sorting-associated protein 37A OS=Homo sapiens OX=9606 GN=VPS37A PE=1 SV=1	0,01659031	0,072892
Q96PZ0	Pseudouridylate synthase 7 homolog OS=Homo sapiens OX=9606 GN=PUS7 PE=1 SV=2	0,031613458	0,072741
Q8WV99	AN1-type zinc finger protein 2B OS=Homo sapiens OX=9606 GN=ZFAND2B PE=1 SV=1	-0,033112202	0,070297
P29144	Tripeptidyl-peptidase 2 OS=Homo sapiens OX=9606 GN=TPP2 PE=1 SV=4	0,008024447	0,070057
Q9UKK9	ADP-sugar pyrophosphatase OS=Homo sapiens OX=9606 GN=NUDT5 PE=1 SV=1	0,015950424	0,070015
Q9UHY7	Enolase-phosphatase E1 OS=Homo sapiens OX=9606 GN=ENOPH1 PE=1 SV=1	-0,024175726	0,069861
Q14697	Neutral alpha-glucosidase AB OS=Homo sapiens OX=9606 GN=GANAB PE=1 SV=3	-0,005421195	0,069618
Q08722	Leukocyte surface antigen CD47 OS=Homo sapiens OX=9606 GN=CD47 PE=1 SV=1	0,024081273	0,069582
Q14978	Nucleolar and coiled-body phosphoprotein 1 OS=Homo sapiens OX=9606 GN=NOLC1 PE=1 SV=2	-0,032722938	0,069454
Q96BM9	#N/D	-0,017461761	0,069098
Q5SW79	Centrosomal protein of 170 kDa OS=Homo sapiens OX=9606 GN=CEP170 PE=1 SV=1	0,009631865	0,068999
P05230	Fibroblast growth factor 1 OS=Homo sapiens OX=9606 GN=FGF1 PE=1 SV=1	0,019864349	0,0689
Q92614	Unconventional myosin-XVIIIa OS=Homo sapiens OX=9606 GN=MYO18A PE=1 SV=3	0,023722901	0,068524
Q13347	Eukaryotic translation initiation factor 3 subunit I OS=Homo sapiens OX=9606 GN=EIF3I PE=1 SV=1	0,009243854	0,06846
Q9NRX5	Serine incorporator 1 OS=Homo sapiens OX=9606 GN=SERINC1 PE=1 SV=1	0,032240666	0,068409
Q93034	Cullin-5 OS=Homo sapiens OX=9606 GN=CUL5 PE=1 SV=4	-0,011055687	0,067302
Q14566	DNA replication licensing factor MCM6 OS=Homo sapiens OX=9606 GN=MCM6 PE=1 SV=1	-0,009387647	0,06721
Q96GK7	Fumarylacetoacetate hydrolase domain-containing protein 2A OS=Homo sapiens OX=9606 GN=FAHD2A PE=1 SV=1	-0,023269114	0,067185
Q9BW62	Katanin p60 ATPase-containing subunit A-like 1 OS=Homo sapiens OX=9606 GN=KATNAL1 PE=1 SV=1	0,025555618	0,067005
O95210	Starch-binding domain-containing protein 1 OS=Homo sapiens OX=9606 GN=STBD1 PE=1 SV=1	0,019317935	0,066963
O95104	SR-related and CTD-associated factor 4 OS=Homo sapiens OX=9606 GN=SCAF4 PE=1 SV=3	0,031554563	0,066924
Q9NUQ8	ATP-binding cassette sub-family F member 3 OS=Homo sapiens OX=9606 GN=ABCF3 PE=1 SV=2	-0,014006916	0,066892

Q5JSZ5	Protein PRRC2B OS=Homo sapiens OX=9606 GN=PRRC2B PE=1 SV=2	-0,02311691	0,066736
P17980	26S proteasome regulatory subunit 6A OS=Homo sapiens OX=9606 GN=PSMC3 PE=1 SV=3	0,008025321	0,066646
Q86SZ2	Trafficking protein particle complex subunit 6B OS=Homo sapiens OX=9606 GN=TRAPPC6B PE=1 SV=1	0,022914569	0,06614
Q9Y333	U6 snRNA-associated Sm-like protein LSm2 OS=Homo sapiens OX=9606 GN=LSM2 PE=1 SV=1	0,022861932	0,065985
P50613	Cyclin-dependent kinase 7 OS=Homo sapiens OX=9606 GN=CDK7 PE=1 SV=1	-0,031050032	0,065833
Q9UKX7	Nuclear pore complex protein Nup50 OS=Homo sapiens OX=9606 GN=NUP50 PE=1 SV=2	-0,016465547	0,065072
Q99439	Calponin-2 OS=Homo sapiens OX=9606 GN=CNN2 PE=1 SV=4	-0,009069188	0,064881
Q96A49	Synapse-associated protein 1 OS=Homo sapiens OX=9606 GN=SYAP1 PE=1 SV=1	0,012559383	0,064449
Q7L0Y3	tRNA methyltransferase 10 homolog C OS=Homo sapiens OX=9606 GN=TRMT10C PE=1 SV=2	0,018427657	0,063813
P14317	Hematopoietic lineage cell-specific protein OS=Homo sapiens OX=9606 GN=HCLS1 PE=1 SV=3	-0,016612923	0,063713
Q9UBB5	Methyl-CpG-binding domain protein 2 OS=Homo sapiens OX=9606 GN=MBD2 PE=1 SV=1	-0,010515636	0,06334
O00483	Cytochrome c oxidase subunit NDUFA4 OS=Homo sapiens OX=9606 GN=NDUFA4 PE=1 SV=1	-0,029842496	0,063224
P14406	Cytochrome c oxidase subunit 7A2, mitochondrial OS=Homo sapiens OX=9606 GN=COX7A2 PE=1 SV=1	0,029643498	0,062794
Q96K17	Transcription factor BTF3 homolog 4 OS=Homo sapiens OX=9606 GN=BTF3L4 PE=1 SV=1	0,018137964	0,062789
Q9Y673	Dolichyl-phosphate beta-glucosyltransferase OS=Homo sapiens OX=9606 GN=ALG5 PE=1 SV=1	-0,02162783	0,062351
Q9Y4E1	#N/D	0,008697849	0,062169
Q5VTR2	E3 ubiquitin-protein ligase BRE1A OS=Homo sapiens OX=9606 GN=RNF20 PE=1 SV=2	-0,02930302	0,062059
Q9Y3D0	Cytosolic iron-sulfur assembly component 2B OS=Homo sapiens OX=9606 GN=CIAO2B PE=1 SV=1	-0,029249973	0,061945
P60842	Eukaryotic initiation factor 4A-I OS=Homo sapiens OX=9606 GN=EIF4A1 PE=1 SV=1	-0,006061797	0,061837
Q99584	Protein S100-A13 OS=Homo sapiens OX=9606 GN=S100A13 PE=1 SV=1	0,017853668	0,061785
Q9ULW0	Targeting protein for Xklp2 OS=Homo sapiens OX=9606 GN=TPX2 PE=1 SV=2	-0,029011225	0,06143
P49023	Paxillin OS=Homo sapiens OX=9606 GN=PXN PE=1 SV=3	0,008573409	0,061261
P55854	Small ubiquitin-related modifier 3 OS=Homo sapiens OX=9606 GN=SUMO3 PE=1 SV=2	0,028744303	0,060854
O95197	Reticulon-3 OS=Homo sapiens OX=9606 GN=RTN3 PE=1 SV=2	-0,021082308	0,060748
Q9BZK7	F-box-like/WD repeat-containing protein TBL1XR1 OS=Homo sapiens OX=9606 GN=TBL1XR1 PE=1 SV=1	0,0108884	0,05943
Q9UBC5	Unconventional myosin-Ia OS=Homo sapiens OX=9606 GN=MYO1A PE=1 SV=1	0,015064456	0,059426
Q92791	Endoplasmic reticulum protein SC65 OS=Homo sapiens OX=9606 GN=P3H4 PE=1 SV=1	0,008938276	0,059242
Q9Y2X7	ARF GTPase-activating protein GIT1 OS=Homo sapiens OX=9606 GN=GIT1 PE=1 SV=2	-0,017920732	0,059084
Q9NRW1	#N/D	0,02051528	0,059083
Q96KC8	DnaJ homolog subfamily C member 1 OS=Homo sapiens OX=9606 GN=DNAJC1 PE=1 SV=1	0,011515765	0,058989
P05109	Protein S100-A8 OS=Homo sapiens OX=9606 GN=S100A8 PE=1 SV=1	-0,027838577	0,058902
P35555	Fibrillin-1 OS=Homo sapiens OX=9606 GN=FBN1 PE=1 SV=4	-0,004201424	0,058736
Q9P2E3	NFX1-type zinc finger-containing protein 1 OS=Homo sapiens OX=9606 GN=ZNFX1 PE=2 SV=2	0,040328769	0,058623
Q9BU23	Lipase maturation factor 2 OS=Homo sapiens OX=9606 GN=LMF2 PE=1 SV=2	-0,009589719	0,058204
Q96FZ7	Charged multivesicular body protein 6 OS=Homo sapiens OX=9606 GN=CHMP6 PE=1 SV=3	-0,027464792	0,058097
Q9Y4I1	Unconventional myosin-Va OS=Homo sapiens OX=9606 GN=MYO5A PE=1 SV=2	-0,010044328	0,057961
Q9HCN8	Stromal cell-derived factor 2-like protein 1 OS=Homo sapiens OX=9606 GN=SDF2L1 PE=1 SV=2	0,027059447	0,057225
Q99661	Kinesin-like protein KIF2C OS=Homo sapiens OX=9606 GN=KIF2C PE=1 SV=2	-0,033121503	0,057197
O15031	Plexin-B2 OS=Homo sapiens OX=9606 GN=PLXNB2 PE=1 SV=3	-0,007494596	0,05706
O43818	U3 small nucleolar RNA-interacting protein 2 OS=Homo sapiens OX=9606 GN=RRP9 PE=1 SV=1	-0,019744347	0,056822
O15427	Monocarboxylate transporter 4 OS=Homo sapiens OX=9606 GN=SLC16A3 PE=1 SV=1	0,012950722	0,056601
Q5CZ79	Ankyrin repeat domain-containing protein 20B OS=Homo sapiens OX=9606 GN=ANKRD20A8P PE=2 SV=2	-0,110277957	0,056101
P61962	DDB1- and CUL4-associated factor 7 OS=Homo sapiens OX=9606 GN=DCAF7 PE=1 SV=1	-0,019277373	0,055454
O75534	Cold shock domain-containing protein E1 OS=Homo sapiens OX=9606 GN=CSDE1 PE=1 SV=2	0,005783994	0,054724
O75153	Clustered mitochondria protein homolog OS=Homo sapiens OX=9606 GN=CLUH PE=1 SV=2	-0,015827065	0,054646
Q8N3U4	Cohesin subunit SA-2 OS=Homo sapiens OX=9606 GN=STAG2 PE=1 SV=3	0,105501658	0,054551
Q86VI3	Ras GTPase-activating-like protein IQGAP3 OS=Homo sapiens OX=9606 GN=IQGAP3 PE=1 SV=2	-0,012464713	0,054439
Q8NBU5	ATPase family AAA domain-containing protein 1 OS=Homo sapiens OX=9606 GN=ATAD1 PE=1 SV=1	-0,018890653	0,054321
P62491	Ras-related protein Rab-11A OS=Homo sapiens OX=9606 GN=RAB11A PE=1 SV=3	-0,008193929	0,054219
P46736	Lys-63-specific deubiquitinase BRCC36 OS=Homo sapiens OX=9606 GN=BRCC3 PE=1 SV=2	0,015681893	0,054136
P31943	Heterogeneous nuclear ribonucleoprotein H OS=Homo sapiens OX=9606 GN=HNRNPH1 PE=1 SV=4	-0,007867263	0,054107
Q96CW1	AP-2 complex subunit mu OS=Homo sapiens OX=9606 GN=AP2M1 PE=1 SV=2	-0,006704669	0,054004
P00505	Aspartate aminotransferase, mitochondrial OS=Homo sapiens OX=9606 GN=GOT2 PE=1 SV=3	-0,010505445	0,05372
Q9H1E3	Nuclear ubiquitous casein and cyclin-dependent kinase substrate 1 OS=Homo sapiens OX=9606 GN=NUCKS1 PE=1 SV=1	0,018584572	0,053426
Q6ZW31	Rho GTPase-activating protein SYDE1 OS=Homo sapiens OX=9606 GN=SYDE1 PE=1 SV=1	0,025147334	0,053115
P13797	Plastin-3 OS=Homo sapiens OX=9606 GN=PLS3 PE=1 SV=4	0,004468966	0,052597
Q4V328	GRIP1-associated protein 1 OS=Homo sapiens OX=9606 GN=GRIPAP1 PE=1 SV=2	-0,012039347	0,052548
Q96SL4	Glutathione peroxidase 7 OS=Homo sapiens OX=9606 GN=GPX7 PE=1 SV=1	-0,011990269	0,05233
Q86SQ0	Pleckstrin homology-like domain family B member 2 OS=Homo sapiens OX=9606 GN=PHLDB2 PE=1 SV=2	0,018191087	0,052276
Q9H1K1	Iron-sulfur cluster assembly enzyme ISCU, mitochondrial OS=Homo sapiens OX=9606 GN=ISCU PE=1 SV=2	0,024715791	0,052189
O60437	Periplakin OS=Homo sapiens OX=9606 GN=PPL PE=1 SV=4	0,011909782	0,052028
Q9Y383	Putative RNA-binding protein Luc7-like 2 OS=Homo sapiens OX=9606 GN=LUC7L2 PE=1 SV=2	0,010916017	0,051876
P61457	Pterin-4-alpha-carbinolamine dehydratase OS=Homo sapiens OX=9606 GN=PCBD1 PE=1 SV=2	-0,024506047	0,051739
O60831	PRA1 family protein 2 OS=Homo sapiens OX=9606 GN=PRAF2 PE=1 SV=1	0,017970713	0,051632
Q96IU4	Protein ABHD14B OS=Homo sapiens OX=9606 GN=ABHD14B PE=1 SV=1	0,01176048	0,05131
O14773	Tripeptidyl-peptidase 1 OS=Homo sapiens OX=9606 GN=TPP1 PE=1 SV=2	-0,017820673	0,051193
P46060	Ran GTPase-activating protein 1 OS=Homo sapiens OX=9606 GN=RANGAP1 PE=1 SV=1	-0,006158671	0,05088
Q01658	Protein Dr1 OS=Homo sapiens OX=9606 GN=DR1 PE=1 SV=1	0,014483851	0,049932
O15145	Actin-related protein 2/3 complex subunit 3 OS=Homo sapiens OX=9606 GN=ARPC3 PE=1 SV=3	-0,010512595	0,049927
Q6YHK3	CD109 antigen OS=Homo sapiens OX=9606 GN=CD109 PE=1 SV=2	-0,006570353	0,049905
Q9Y3B9	RRP15-like protein OS=Homo sapiens OX=9606 GN=RRP15 PE=1 SV=2	-0,026136649	0,049699
Q9Y2E4	Disco-interacting protein 2 homolog C OS=Homo sapiens OX=9606 GN=DIP2C PE=1 SV=2	-0,034806013	0,049401
Q05932	Folylpolyglutamate synthase, mitochondrial OS=Homo sapiens OX=9606 GN=FPGS PE=1 SV=3	0,01302333	0,049228
Q9BRP8	Partner of Y14 and mago OS=Homo sapiens OX=9606 GN=PYM1 PE=1 SV=1	0,011266216	0,049119
P42356	Phosphatidylinositol 4-kinase alpha OS=Homo sapiens OX=9606 GN=PI4KA PE=1 SV=4	-0,016942704	0,048631
Q9NQ48	Leucine zipper transcription factor-like protein 1 OS=Homo sapiens OX=9606 GN=LZTFL1 PE=1 SV=1	0,014016286	0,048294
Q9NRW7	Vacuolar protein sorting-associated protein 45 OS=Homo sapiens OX=9606 GN=VPS45 PE=1 SV=1	-0,007948833	0,048084
O43670	BUB3-interacting and GLEBS motif-containing protein ZNF207 OS=Homo sapiens OX=9606 GN=ZNF207 PE=1 SV=1	-0,016087595	0,046139
Q8IWU6	Extracellular sulfatase Sulf-1 OS=Homo sapiens OX=9606 GN=SULF1 PE=1 SV=1	0,011615528	0,045614
Q96EY7	Pentatricopeptide repeat domain-containing protein 3, mitochondrial OS=Homo sapiens OX=9606 GN=PTCD3 PE=1 SV=3	0,015900408	0,045595
P33527	Multidrug resistance-associated protein 1 OS=Homo sapiens OX=9606 GN=ABCC1 PE=1 SV=3	0,005778254	0,045131
Q8IVM0	Coiled-coil domain-containing protein 50 OS=Homo sapiens OX=9606 GN=CCDC50 PE=1 SV=1	-0,007747228	0,044507
P18031	Tyrosine-protein phosphatase non-receptor type 1 OS=Homo sapiens OX=9606 GN=PTPN1 PE=1 SV=1	0,008729322	0,044502
P38159	RNA-binding motif protein, X chromosome OS=Homo sapiens OX=9606 GN=RBMX PE=1 SV=3	-0,006452791	0,044234
Q9Y676	28S ribosomal protein S18b, mitochondrial OS=Homo sapiens OX=9606 GN=MRPS18B PE=1 SV=1	0,020969898	0,04417
Q5VY43	Platelet endothelial aggregation receptor 1 OS=Homo sapiens OX=9606 GN=PEAR1 PE=1 SV=1	0,012455829	0,042841
P43155	Carnitine O-acetyltransferase OS=Homo sapiens OX=9606 GN=CRAT PE=1 SV=5	-0,024920057	0,042466
P22087	rRNA 2'-O-methyltransferase fibrillarin OS=Homo sapiens OX=9606 GN=FBL PE=1 SV=2	0,009716375	0,042266
P84022	Mothers against decapentaplegic homolog 3 OS=Homo sapiens OX=9606 GN=SMAD3 PE=1 SV=1	0,020041731	0,042188
O95671	Probable bifunctional dTTP/UTP pyrophosphatase/methyltransferase protein OS=Homo sapiens OX=9606 GN=ASMTL PE=1 SV=3	0,008213292	0,041835
Q9H0A8	COMM domain-containing protein 4 OS=Homo sapiens OX=9606 GN=COMMD4 PE=1 SV=1	0,010634513	0,041708
Q14435	Polypeptide N-acetylgalactosaminyltransferase 3 OS=Homo sapiens OX=9606 GN=GALNT3 PE=1 SV=2	-0,021591086	0,040742
Q9NVX2	Notchless protein homolog 1 OS=Homo sapiens OX=9606 GN=NLE1 PE=1 SV=4	0,019145384	0,040277
Q13618	Cullin-3 OS=Homo sapiens OX=9606 GN=CUL3 PE=1 SV=2	-0,007000491	0,040159
P08236	Beta-glucuronidase OS=Homo sapiens OX=9606 GN=GUSB PE=1 SV=2	0,008745144	0,039875
Q15233	Non-POU domain-containing octamer-binding protein OS=Homo sapiens OX=9606 GN=NONO PE=1 SV=4	0,005205037	0,039396
Q9UNX4	WD repeat-containing protein 3 OS=Homo sapiens OX=9606 GN=WDR3 PE=1 SV=1	-0,018610454	0,039138
Q6VY07	Phosphofurin acidic cluster sorting protein 1 OS=Homo sapiens OX=9606 GN=PACS1 PE=1 SV=2	0,008911152	0,038717

Q0ZGT2	Nexilin OS=Homo sapiens OX=9606 GN=NEXN PE=1 SV=1	-0,005447859	0,038637
Q04759	#N/D	-0,010856596	0,038493
Q7Z434	Mitochondrial antiviral-signaling protein OS=Homo sapiens OX=9606 GN=MAVS PE=1 SV=2	0,007000338	0,037938
P05166	Propionyl-CoA carboxylase beta chain, mitochondrial OS=Homo sapiens OX=9606 GN=PCCB PE=1 SV=3	-0,01792214	0,037673
Q92878	DNA repair protein RAD50 OS=Homo sapiens OX=9606 GN=RAD50 PE=1 SV=1	-0,010776931	0,036995
Q96EP5	DAZ-associated protein 1 OS=Homo sapiens OX=9606 GN=DAZAP1 PE=1 SV=1	0,009311298	0,036455
Q16698	2,4-dienoyl-CoA reductase, mitochondrial OS=Homo sapiens OX=9606 GN=DECR1 PE=1 SV=1	0,00621701	0,03561
P61020	Ras-related protein Rab-5B OS=Homo sapiens OX=9606 GN=RAB5B PE=1 SV=1	-0,012260066	0,035035
Q03519	Antigen peptide transporter 2 OS=Homo sapiens OX=9606 GN=TAP2 PE=1 SV=1	0,012256238	0,035024
Q06033	Inter-alpha-trypsin inhibitor heavy chain H3 OS=Homo sapiens OX=9606 GN=ITIH3 PE=1 SV=2	0,012105844	0,034589
Q9H9E3	Conserved oligomeric Golgi complex subunit 4 OS=Homo sapiens OX=9606 GN=COG4 PE=1 SV=3	-0,009908275	0,033979
Q13155	Aminoacyl tRNA synthase complex-interacting multifunctional protein 2 OS=Homo sapiens OX=9606 GN=AIMP2 PE=1 SV=2	0,007066057	0,033375
O15116	U6 snRNA-associated Sm-like protein LSm1 OS=Homo sapiens OX=9606 GN=LSM1 PE=1 SV=1	-0,011666832	0,033321
Q15041	ADP-ribosylation factor-like protein 6-interacting protein 1 OS=Homo sapiens OX=9606 GN=ARL6IP1 PE=1 SV=2	0,015797424	0,033159
P49643	DNA primase large subunit OS=Homo sapiens OX=9606 GN=PRIM2 PE=1 SV=2	0,015701341	0,032955
P42574	Caspase-3 OS=Homo sapiens OX=9606 GN=CASP3 PE=1 SV=2	-0,006971697	0,032924
Q32P44	Echinoderm microtubule-associated protein-like 3 OS=Homo sapiens OX=9606 GN=EML3 PE=1 SV=1	0,011512427	0,032875
Q9BRJ2	39S ribosomal protein L45, mitochondrial OS=Homo sapiens OX=9606 GN=MRPL45 PE=1 SV=2	-0,021117462	0,032605
Q8N1F7	Nuclear pore complex protein Nup93 OS=Homo sapiens OX=9606 GN=NUP93 PE=1 SV=2	-0,004592359	0,032503
O43837	Isocitrate dehydrogenase [NAD] subunit beta, mitochondrial OS=Homo sapiens OX=9606 GN=IDH3B PE=1 SV=2	0,007373648	0,031965
Q13619	Cullin-4A OS=Homo sapiens OX=9606 GN=CUL4A PE=1 SV=3	-0,005855476	0,031667
Q14919	Dr1-associated corepressor OS=Homo sapiens OX=9606 GN=DRAP1 PE=1 SV=3	-0,009087401	0,031134
Q9Y5K5	Ubiquitin carboxyl-terminal hydrolase isozyme L5 OS=Homo sapiens OX=9606 GN=UCHL5 PE=1 SV=3	-0,008745729	0,029952
P63104	14-3-3 protein zeta/delta OS=Homo sapiens OX=9606 GN=YWHAZ PE=1 SV=1	-0,003626286	0,029747
Q92759	General transcription factor IIH subunit 4 OS=Homo sapiens OX=9606 GN=GTF2H4 PE=1 SV=1	0,01418136	0,029734
Q96JC1	Vam6/Vps39-like protein OS=Homo sapiens OX=9606 GN=VPS39 PE=1 SV=2	0,010339552	0,029493
O00399	Dynactin subunit 6 OS=Homo sapiens OX=9606 GN=DCTN6 PE=1 SV=1	-0,010314198	0,02942
Q9Y6I9	Testis-expressed protein 264 OS=Homo sapiens OX=9606 GN=TEX264 PE=1 SV=1	0,010145993	0,028935
Q14376	UDP-glucose 4-epimerase OS=Homo sapiens OX=9606 GN=GALE PE=1 SV=2	0,010892072	0,028761
P09382	Galectin-1 OS=Homo sapiens OX=9606 GN=LGALS1 PE=1 SV=2	0,004784067	0,028753
A6NIH7	Protein unc-119 homolog B OS=Homo sapiens OX=9606 GN=UNC119B PE=1 SV=1	0,013673376	0,028659
Q00403	Transcription initiation factor IIB OS=Homo sapiens OX=9606 GN=GTF2B PE=1 SV=1	0,009936058	0,028331
P49792	E3 SUMO-protein ligase RanBP2 OS=Homo sapiens OX=9606 GN=RANBP2 PE=1 SV=2	-0,003953289	0,027937
P23368	NAD-dependent malic enzyme, mitochondrial OS=Homo sapiens OX=9606 GN=ME2 PE=1 SV=1	-0,005042216	0,027228
Q8WVC6	Dephospho-CoA kinase domain-containing protein OS=Homo sapiens OX=9606 GN=DCAKD PE=1 SV=1	0,012994146	0,027223
Q96RF0	Sorting nexin-18 OS=Homo sapiens OX=9606 GN=SNX18 PE=1 SV=2	0,007857544	0,026883
Q92530	Proteasome inhibitor PI31 subunit OS=Homo sapiens OX=9606 GN=PSMF1 PE=1 SV=2	0,009400061	0,026789
Q9Y6K9	NF-kappa-B essential modulator OS=Homo sapiens OX=9606 GN=IKBKG PE=1 SV=2	0,006839363	0,02669
Q13683	Integrin alpha-7 OS=Homo sapiens OX=9606 GN=ITGA7 PE=1 SV=3	-0,005599721	0,026387
P26373	60S ribosomal protein L13 OS=Homo sapiens OX=9606 GN=RPL13 PE=1 SV=4	0,004604434	0,026291
Q9UNN5	FAS-associated factor 1 OS=Homo sapiens OX=9606 GN=FAF1 PE=1 SV=2	0,007604792	0,02601
Q0VDG4	Secernin-3 OS=Homo sapiens OX=9606 GN=SCRN3 PE=1 SV=1	-0,012393896	0,025955
O15347	High mobility group protein B3 OS=Homo sapiens OX=9606 GN=HMGB3 PE=1 SV=4	-0,01347494	0,025709
P61313	60S ribosomal protein L15 OS=Homo sapiens OX=9606 GN=RPL15 PE=1 SV=2	-0,00474422	0,025605
Q9Y2R5	28S ribosomal protein S17, mitochondrial OS=Homo sapiens OX=9606 GN=MRPS17 PE=1 SV=1	-0,01201906	0,025163
Q4G0J3	La-related protein 7 OS=Homo sapiens OX=9606 GN=LARP7 PE=1 SV=1	0,002112398	0,024998
Q01629	Interferon-induced transmembrane protein 2 OS=Homo sapiens OX=9606 GN=IFITM2 PE=1 SV=2	0,011765247	0,024628
Q7L2H7	Eukaryotic translation initiation factor 3 subunit M OS=Homo sapiens OX=9606 GN=EIF3M PE=1 SV=1	0,004271656	0,024375
P05388	60S acidic ribosomal protein P0 OS=Homo sapiens OX=9606 GN=RPLP0 PE=1 SV=1	-0,003540286	0,024105
P50579	Methionine aminopeptidase 2 OS=Homo sapiens OX=9606 GN=METAP2 PE=1 SV=1	0,004760329	0,024103
P49720	Proteasome subunit beta type-3 OS=Homo sapiens OX=9606 GN=PSMB3 PE=1 SV=2	-0,00435043	0,023462
P49902	Cytosolic purine 5'-nucleotidase OS=Homo sapiens OX=9606 GN=NT5C2 PE=1 SV=1	0,003860691	0,02316
Q8TF05	Serine/threonine-protein phosphatase 4 regulatory subunit 1 OS=Homo sapiens OX=9606 GN=PPP4R1 PE=1 SV=1	0,003848299	0,023085
O00159	Unconventional myosin-Ic OS=Homo sapiens OX=9606 GN=MYO1C PE=1 SV=4	0,001747777	0,022566
Q15645	Pachytene checkpoint protein 2 homolog OS=Homo sapiens OX=9606 GN=TRIP13 PE=1 SV=2	-0,010435912	0,021825
Q9UBQ7	Glyoxylate reductase/hydroxypyruvate reductase OS=Homo sapiens OX=9606 GN=GRHPR PE=1 SV=1	0,003476782	0,021824
Q8TBQ9	Protein kish-A OS=Homo sapiens OX=9606 GN=TMEM167A PE=1 SV=1	0,010351394	0,021647
Q9BVG4	Protein PBDC1 OS=Homo sapiens OX=9606 GN=PBDC1 PE=1 SV=1	-0,00387975	0,020906
Q86W26	NACHT, LRR and PYD domains-containing protein 10 OS=Homo sapiens OX=9606 GN=NLRP10 PE=1 SV=1	0,009682338	0,020238
Q68E01	Integrator complex subunit 3 OS=Homo sapiens OX=9606 GN=INTS3 PE=1 SV=1	0,009591011	0,020046
P36957	Dihydrolipoyllysine-residue succinyltransferase component of 2-oxoglutarate dehydrogenase complex, mitochondrial OS=Homo sapiens	-0,003301633	0,019783
Q8NFU7	Methylcytosine dioxygenase TET1 OS=Homo sapiens OX=9606 GN=TET1 PE=1 SV=2	0,016629944	0,019655
Q5XKP0	MICOS complex subunit MIC13 OS=Homo sapiens OX=9606 GN=MICOS13 PE=1 SV=1	0,009262519	0,019355
P61019	Ras-related protein Rab-2A OS=Homo sapiens OX=9606 GN=RAB2A PE=1 SV=1	0,004411967	0,019043
Q8IVL6	Prolyl 3-hydroxylase 3 OS=Homo sapiens OX=9606 GN=P3H3 PE=1 SV=1	0,002895375	0,018933
Q5XPI4	E3 ubiquitin-protein ligase RNF123 OS=Homo sapiens OX=9606 GN=RNF123 PE=1 SV=1	0,008948272	0,018694
P62140	Serine/threonine-protein phosphatase PP1-beta catalytic subunit OS=Homo sapiens OX=9606 GN=PPP1CB PE=1 SV=3	-0,004663907	0,018148
Q8WU10	Pyridine nucleotide-disulfide oxidoreductase domain-containing protein 1 OS=Homo sapiens OX=9606 GN=PYROXD1 PE=1 SV=1	0,008397011	0,017536
P15151	Poliovirus receptor OS=Homo sapiens OX=9606 GN=PVR PE=1 SV=2	-0,005072448	0,017298
Q8TER5	Rho guanine nucleotide exchange factor 40 OS=Homo sapiens OX=9606 GN=ARHGEF40 PE=1 SV=3	-0,00439932	0,017112
P35914	Hydroxymethylglutaryl-CoA lyase, mitochondrial OS=Homo sapiens OX=9606 GN=HMGCL PE=1 SV=2	-0,004881569	0,016643
Q14392	Transforming growth factor beta activator LRRC32 OS=Homo sapiens OX=9606 GN=LRRC32 PE=1 SV=1	-0,006638499	0,016313
Q15208	Serine/threonine-protein kinase 38 OS=Homo sapiens OX=9606 GN=STK38 PE=1 SV=1	-0,004718444	0,016084
P46937	Transcriptional coactivator YAP1 OS=Homo sapiens OX=9606 GN=YAP1 PE=1 SV=2	-0,002360777	0,01603
Q5SW96	Low density lipoprotein receptor adapter protein 1 OS=Homo sapiens OX=9606 GN=LDLRAP1 PE=1 SV=3	-0,004500813	0,015338
P22681	E3 ubiquitin-protein ligase CBL OS=Homo sapiens OX=9606 GN=CBL PE=1 SV=2	0,007319948	0,015275
P46459	Vesicle-fusing ATPase OS=Homo sapiens OX=9606 GN=NSF PE=1 SV=3	-0,001527966	0,01454
P61587	Rho-related GTP-binding protein RhoE OS=Homo sapiens OX=9606 GN=RND3 PE=1 SV=1	0,005108244	0,014498
Q13190	Syntaxin-5 OS=Homo sapiens OX=9606 GN=STX5 PE=1 SV=2	0,006719562	0,014016
Q9H444	Charged multivesicular body protein 4b OS=Homo sapiens OX=9606 GN=CHMP4B PE=1 SV=1	-0,002736773	0,013809
O60716	Catenin delta-1 OS=Homo sapiens OX=9606 GN=CTNND1 PE=1 SV=1	0,001304998	0,013535
O95777	U6 snRNA-associated Sm-like protein LSm8 OS=Homo sapiens OX=9606 GN=LSM8 PE=1 SV=3	-0,003952893	0,013463
P04216	Thy-1 membrane glycoprotein OS=Homo sapiens OX=9606 GN=THY1 PE=1 SV=2	0,006414305	0,013377
Q9P2J5	Leucine--tRNA ligase, cytoplasmic OS=Homo sapiens OX=9606 GN=LARS PE=1 SV=2	0,001247051	0,013338
Q9H3U1	Protein unc-45 homolog A OS=Homo sapiens OX=9606 GN=UNC45A PE=1 SV=1	0,001890105	0,01329
P53611	Geranylgeranyl transferase type-2 subunit beta OS=Homo sapiens OX=9606 GN=RABGGTB PE=1 SV=2	-0,006273429	0,013082
P23470	Receptor-type tyrosine-protein phosphatase gamma OS=Homo sapiens OX=9606 GN=PTPRG PE=1 SV=4	0,008514407	0,013018
Q15814	Tubulin-specific chaperone C OS=Homo sapiens OX=9606 GN=TBCC PE=1 SV=2	0,004495457	0,012751
Q99873	Protein arginine N-methyltransferase 1 OS=Homo sapiens OX=9606 GN=PRMT1 PE=1 SV=3	0,001770383	0,012445
Q9Y5U8	Mitochondrial pyruvate carrier 1 OS=Homo sapiens OX=9606 GN=MPC1 PE=1 SV=1	-0,005918363	0,012338
Q8NFH3	Nucleoporin Nup43 OS=Homo sapiens OX=9606 GN=NUP43 PE=1 SV=1	0,005845721	0,012186
Q9UHY8	Fasciculation and elongation protein zeta-2 OS=Homo sapiens OX=9606 GN=FEZ2 PE=1 SV=2	0,005808775	0,012109
Q15185	Prostaglandin E synthase 3 OS=Homo sapiens OX=9606 GN=PTGES3 PE=1 SV=1	-0,002779267	0,011967
Q16352	Alpha-internexin OS=Homo sapiens OX=9606 GN=INA PE=1 SV=2	-0,005671019	0,01182
P61421	V-type proton ATPase subunit d 1 OS=Homo sapiens OX=9606 GN=ATP6V0D1 PE=1 SV=1	0,001839723	0,010991
O00244	Copper transport protein ATOX1 OS=Homo sapiens OX=9606 GN=ATOX1 PE=1 SV=1	0,003851646	0,010918
Q71DI3	Histone H3.2 OS=Homo sapiens OX=9606 GN=HIST2H3A PE=1 SV=3	-0,005128138	0,010685
Q99536	Synaptic vesicle membrane protein VAT-1 homolog OS=Homo sapiens OX=9606 GN=VAT1 PE=1 SV=2	0,001421527	0,010655

Q9BUQ8	Probable ATP-dependent RNA helicase DDX23 OS=Homo sapiens OX=9606 GN=DDX23 PE=1 SV=3	-0,003675292	0,010416
O43504	Ragulator complex protein LAMTOR5 OS=Homo sapiens OX=9606 GN=LAMTOR5 PE=1 SV=1	0,00267845	0,010395
Q96EQ0	Small glutamine-rich tetratricopeptide repeat-containing protein beta OS=Homo sapiens OX=9606 GN=SGTB PE=1 SV=1	0,002380789	0,010245
O15212	Prefoldin subunit 6 OS=Homo sapiens OX=9606 GN=PFDN6 PE=1 SV=1	-0,002897233	0,009855
O43150	Arf-GAP with SH3 domain, ANK repeat and PH domain-containing protein 2 OS=Homo sapiens OX=9606 GN=ASAP2 PE=1 SV=3	-0,004712742	0,009816
Q7Z6E9	E3 ubiquitin-protein ligase RBBP6 OS=Homo sapiens OX=9606 GN=RBBP6 PE=1 SV=1	-0,004702986	0,009796
O95297	Myelin protein zero-like protein 1 OS=Homo sapiens OX=9606 GN=MPZL1 PE=1 SV=1	0,003292078	0,009327
Q04656	Copper-transporting ATPase 1 OS=Homo sapiens OX=9606 GN=ATP7A PE=1 SV=4	0,003825248	0,009157
P29372	DNA-3-methyladenine glycosylase OS=Homo sapiens OX=9606 GN=MPG PE=1 SV=3	0,004308981	0,008973
Q9Y3S2	Zinc finger protein 330 OS=Homo sapiens OX=9606 GN=ZNF330 PE=1 SV=1	-0,004016768	0,008363
Q9ULV4	Coronin-1C OS=Homo sapiens OX=9606 GN=CORO1C PE=1 SV=1	0,000948446	0,00811
P15529	Membrane cofactor protein OS=Homo sapiens OX=9606 GN=CD46 PE=1 SV=3	0,002793926	0,007912
Q8N163	Cell cycle and apoptosis regulator protein 2 OS=Homo sapiens OX=9606 GN=CCAR2 PE=1 SV=2	0,001153917	0,007516
Q9NP97	Dynein light chain roadblock-type 1 OS=Homo sapiens OX=9606 GN=DYNLRB1 PE=1 SV=3	0,001538199	0,006611
Q12982	BCL2/adenovirus E1B 19 kDa protein-interacting protein 2 OS=Homo sapiens OX=9606 GN=BNIP2 PE=1 SV=1	-0,003168199	0,006592
Q8WWM7	Ataxin-2-like protein OS=Homo sapiens OX=9606 GN=ATXN2L PE=1 SV=2	-0,000738718	0,005693
Q13137	Calcium-binding and coiled-coil domain-containing protein 2 OS=Homo sapiens OX=9606 GN=CALCOCO2 PE=1 SV=1	0,001607937	0,005559
P14735	Insulin-degrading enzyme OS=Homo sapiens OX=9606 GN=IDE PE=1 SV=4	0,000963942	0,005465
Q9UQ16	Dynamin-3 OS=Homo sapiens OX=9606 GN=DNM3 PE=1 SV=4	0,002424273	0,005041
O95721	Synaptosomal-associated protein 29 OS=Homo sapiens OX=9606 GN=SNAP29 PE=1 SV=1	0,001263405	0,004894
O15118	NPC intracellular cholesterol transporter 1 OS=Homo sapiens OX=9606 GN=NPC1 PE=1 SV=2	0,001518402	0,004294
P06748	Nucleophosmin OS=Homo sapiens OX=9606 GN=NPM1 PE=1 SV=2	0,000561381	0,003796
Q96S97	Myeloid-associated differentiation marker OS=Homo sapiens OX=9606 GN=MYADM PE=1 SV=2	-0,001089924	0,0037
O14776	Transcription elongation regulator 1 OS=Homo sapiens OX=9606 GN=TCERG1 PE=1 SV=2	0,001768201	0,003675
P23919	Thymidylate kinase OS=Homo sapiens OX=9606 GN=DTYMK PE=1 SV=4	-0,000932821	0,003166
Q6FI13	Histone H2A type 2-A OS=Homo sapiens OX=9606 GN=HIST2H2AA3 PE=1 SV=3	-0,001258233	0,002614
P13073	Cytochrome c oxidase subunit 4 isoform 1, mitochondrial OS=Homo sapiens OX=9606 GN=COX4I1 PE=1 SV=1	0,00050514	0,002168
Q8IYS2	Uncharacterized protein KIAA2013 OS=Homo sapiens OX=9606 GN=KIAA2013 PE=1 SV=1	0,001269259	0,001858
Q8TED0	U3 small nucleolar RNA-associated protein 15 homolog OS=Homo sapiens OX=9606 GN=UTP15 PE=1 SV=3	0,000174614	0,001714
Q9Y3A3	MOB-like protein phocein OS=Homo sapiens OX=9606 GN=MOB4 PE=1 SV=1	-0,000405425	0,001375
Q8IUF8	Ribosomal oxygenase 2 OS=Homo sapiens OX=9606 GN=RIOX2 PE=1 SV=1	-0,000546357	0,001135
Q9UEW8	STE20/SPS1-related proline-alanine-rich protein kinase OS=Homo sapiens OX=9606 GN=STK39 PE=1 SV=3	-0,000227589	0,000772
P06865	Beta-hexosaminidase subunit alpha OS=Homo sapiens OX=9606 GN=HEXA PE=1 SV=2	0,000162585	0,000629
P35610	Sterol O-acyltransferase 1 OS=Homo sapiens OX=9606 GN=SOAT1 PE=1 SV=3	0,000145795	0,000564
P60033	CD81 antigen OS=Homo sapiens OX=9606 GN=CD81 PE=1 SV=1	0,00022074	0,000458
Q9BWM7	Sideroflexin-3 OS=Homo sapiens OX=9606 GN=SFXN3 PE=1 SV=3	-5,60117E-05	0,000333
